# LCN2 secreted by tissue-infiltrating neutrophils induces the ferroptosis and wasting of adipose and muscle tissues in lung cancer cachexia

**DOI:** 10.1186/s13045-023-01429-1

**Published:** 2023-03-27

**Authors:** Dong Wang, Xiaohui Li, Defeng Jiao, Ying Cai, Liting Qian, Yiqing Shen, Yichen Lu, Yonggang Zhou, Binqing Fu, Rui Sun, Zhigang Tian, Xiaohu Zheng, Haiming Wei

**Affiliations:** 1 Department of Geriatrics, First Affiliated Hospital of USTC, Division of Life Sciences and Medicine, University of Science and Technology of China, Hefei, 230036 China; 2 CAS Key Laboratory of Innate Immunity and Chronic Disease, School of Basic Medical Sciences, Division of Life Sciences and Medicine, University of Science and Technology of China, Hefei, 230027 China; 3 Institue of Immunology, University of Science and Technology of China, Hefei, 230027 Anhui China; 4 The First Affiliated Hospital of USTC, Division of Life Sciences and Medicine, University of Science and Technology of China, Hefei, 230031 Anhui China

**Keywords:** Cachexia, Lung cancer, LCN2, Ferroptosis, Neutrophils

## Abstract

**Background:**

Cancer cachexia is a deadly wasting syndrome that accompanies various diseases (including ~ 50% of cancers). Clinical studies have established that cachexia is not a nutritional deficiency and is linked to expression of certain proteins (*e.g.*, interleukin-6 and C-reactive protein), but much remains unknown about this often fatal syndrome.

**Methods:**

First, cachexia was created in experimental mouse models of lung cancer. Samples of human lung cancer were used to identify the association between the serum lipocalin 2 (LCN2) level and cachexia progression. Then, mouse models with LCN2 blockade or LCN2 overexpression were used to ascertain the role of LCN2 upon ferroptosis and cachexia. Furthermore, antibody depletion of tissue-infiltrating neutrophils (TI-Neu), as well as myeloid-specific-knockout of *Lcn2*, were undertaken to reveal if LCN2 secreted by TI-Neu caused cachexia. Finally, chemical inhibition of ferroptosis was conducted to illustrate the effect of ferroptosis upon tissue wasting.

**Results:**

Protein expression of LCN2 was higher in the wasting adipose tissue and muscle tissues of experimental mouse models of lung cancer cachexia. Moreover, evaluation of lung cancer patients revealed an association between the serum LCN2 level and cachexia progression. Inhibition of LCN2 expression reduced cachexia symptoms significantly and inhibited tissue wasting in vivo. Strikingly, we discovered a significant increase in the number of TI-Neu in wasting tissues, and that these innate immune cells secreted high levels of LCN2. Antibody depletion of TI-Neu, as well as myeloid-specific-knockout of *Lcn2*, prevented ferroptosis and tissue wasting in experimental models of lung cancer cachexia. Chemical inhibition of ferroptosis alleviated tissue wasting significantly and also prolonged the survival of cachectic mice.

**Conclusions:**

Our study provides new insights into how LCN2-induced ferroptosis functionally impacts tissue wasting. We identified LCN2 as a potential target in the treatment of cancer cachexia.

**Supplementary Information:**

The online version contains supplementary material available at 10.1186/s13045-023-01429-1.

## Background

Around 50% of all cancer-related deaths worldwide can be attributed to a wasting condition known as “cachexia”. Cachexia is a complex syndrome that causes ongoing loss of adipose tissue and muscle, and which cannot be reversed with nutritional supplementation [1–3]. The progressive wasting that occurs in cancer cachexia has been suggested to be mediated by circulating factors [3] (e.g., proinflammatory cytokines, hormones, and metal ions), which can originate from various tissues and have different functions [3]. It has also been suggested that interventions for cachexia could be developed by targeting inflammatory processes (*e.g.*, interleukin [IL]-6), although these efforts have not achieved the desired results [4, 5]. Thus, advances in the basic understanding of cachexia and effective targeting strategies are needed.

The protein lipocalin 2 (LCN2) (also known as neutrophil gelatinase-associated lipocalin, siderocalin, or 24p3) functions as a mediator in several diseases associated with cachexia, including cancer, pneumonia, and kidney disease [6–9]. LCN2 has been demonstrated in mechanistic studies to function as an iron-regulatory protein under physiological and inflammatory conditions. In prokaryotes, LCN2 inhibits bacterial siderophores from acquiring iron, thus inhibiting bacterial growth [10]. In mammals, a study using a mouse model of leptomeningeal metastasis showed that cancer cells use lipocalin 2 to collect iron [6]. However, LCN2 in the hypothalamus interacts with the melanocortin-4 receptor, which has been shown to mediate anorexia and promote the loss of lean (skeletal muscle) and fat (adipose tissue) mass in cancer cachexia [11, 12].

Ferroptosis is a form of cell death. It is driven by iron-dependent phospholipid peroxidation and regulated by multiple metabolic and signaling pathways [13, 14]. Since its discovery in 2012, diverse injuries to many organs and various malignant lesions have been pathogenically associated with ferroptosis [13]. In recent years, multiple studies have reported links between ferroptosis and cancer progression. Egolf et al*.* [15] reported that knocking out the gene of an epigenetic regulator, myeloid/lymphoid or mixed-lineage leukemia 4, leads to ferroptosis inhibition and the development of pre-cancerous skin lesions in mice. Ma et al*.* [16] showed that cholesterol in the tumor microenvironment leads to increased cluster of differentiation (CD)36 expression in CD8^+^ T cells and causes these cells to take up polyunsaturated fatty acids and to initiate ferroptosis. Liao et al. [17] reported that CD8^+^ T cells orchestrate tumor ferroptosis via acyl-coa synthetase long chain family member-4. Although a link between cachexia and ferroptosis has not been reported, many therapy-resistant cancer cells (especially those prone to metastasis) are highly susceptible to ferroptosis [18]. Accordingly, it has been proposed that ferroptosis could be regulated by pharmacologic means to treat drug-resistant cancers [19].

In the present study, we found that the ferroptosis of adipose tissue cells caused tissue wasting in experimental models of lung cancer cachexia. Mechanistically, we demonstrated that wasting tissue had an increased number of tissue-infiltrating neutrophils (TI-Neu), and showed that these cells promoted ferroptosis and tissue wasting through LCN2 secretion. Moreover, we showed that the chemical inhibition of ferroptosis inhibited tissue wasting in experimental models of lung cancer cachexia.

## Methods

### Mice

6–10 week-old male C57BL/6 (Cat# T002040), BALB/cJGpt-*Foxn1*^*nu*^/Gpt mice (Cat# D000521) were obtained from GemPharmatech (Nanjing, China). The *Lcn2*^*f/*+^ (Cat# NM-CKO-00134) and *LysM*^*cre*^ mice (Cat# NM-KI-18018) were obtained from ShangHai Model Organisms. *Lcn2*^*f/f*^ mice were crossed with *LysM*^*cre*^ mice to obtain *Lcn2*^*f/f*^;*LysM*^*cre*^ mice. All mice used were housed under specific pathogen-free conditions. All procedures involving experimental animals were approved by the Ethics Committee of the University of Science and Technology of China and were performed in accordance with the National Guidelines for Animal Usage in Research.

### Cell lines

The Lewis lung carcinoma (LLC) and 3T3-L1 cell lines were obtained from The Cell Bank of Type Culture Collection of the Chinese Academy of Sciences (Cat# TCM 7 and Cat#SCSP-5038, respectively). Both cell lines were cultured in DMEM supplemented with 10% FBS (HyClone), 1% streptomycin and penicillin, and were maintained at 37 °C and 5% CO_2_. The cell lines were routinely tested for mycoplasma using the TransDetect PCR mycoplasma detection kit (Transgen, Cat#FM311).

2 days after the 3T3-L1 cells had reached 70% confluence, the cells were treated with 1 μM dexamethasone (Selleck, Cat#S1322), 5 μg/mL insulin (Novoprotein, Cat#P05019), 0.5 μM isobutylmethylxanthine (IBMX) (Selleck, Cat#S5836), and 1 μM rosiglitazone (Selleck, Cat#S2556). Another 2 days later, the cells were treated with 5 μg/mL insulin and 1 μM rosiglitazone. Starting on day 6, the cells were cultured in DMEM supplemented with 10% FBS, 1% streptomycin and penicillin, and treated with 200 ng/mL recombinant mouse LCN2 protein (Novoprotein, Cat#P11672) for 24 h.

### Human samples

Serum samples from healthy donors and from lung cancer patients with and without cachexia were obtained from the First Affiliated Hospital of the University of Science and Technology of China. Patients were diagnosed with cachexia if they had lost > 5% of their body weight over the past 6 months or had a body mass index (BMI) < 20, plus had three of the following criteria: anorexia, decreased muscle strength, fatigue, low fat-free mass index, or abnormal biochemistry results, including increased levels of inflammatory markers (*e.g.,* CRP and IL-6), anemia, or low serum albumin. All samples were collected with the informed consent of the patients, and the experiments were approved by the Ethics Committee of the University of Science and Technology of China (2020-research-36). Details relating to the patients’ cancer type and cachexia are listed in Additional file 4: Table S5.

### Tumor models

LLC-induced cachexia model was established as previous study described [20]. In brief, the male C57BL/6 mice were subcutaneously inoculated with LLC cells (5 × 10^6^ per mouse). Mice were randomly divided into treatment groups while ensuring that the average body weight in each group was roughly the same. Mice which showed significant loss (> 20%) in body weight were defined as having cachexia. Then, they were euthanized and WAT and muscle tissues were harvested to further confirm the cachexia symptom. We comprehensively compared the cachexia phenotype of mice at 1,2,3 weeks and finally determined the analysis of samples on day 21.

For lung cancer patient-derived xenograft (PDX)-induced cachexia, whether or not cachexia occurs is dependent upon the source of the tissue from the tumor patient. We established based on the 3#-Ade PDX of a lung adenocarcinoma. For specific steps and methods, PDX tumors in cold Dulbecco’s Modified Eagle’s Medium (DMEM) were minced into 30–50 mm3 fragments, and each fragment was subcutaneously transplanted into the dorsal flank of 6- to 10-week-old male BALB/cJGpt-*Foxn1*^*nu*^/Gpt mice. Body weight of these tumor-bearing mice were monitored regularly. Mice who showed significant loss (> 20%) in body weight were defined as having cachexia. Then, they were euthanized and WAT and muscle tissues were harvested to further confirm the cachexia symptom.

### Animal treatment protocol

To deplete neutrophils, the male C57BL/6 mice were subcutaneously inoculated with LLC cells (5 × 10^6^ per mouse) on day 0 and then intraperitoneally injected on days − 1, 1, 4, 6, 8, 11, 13, 15, 17, and 20 with a 100 μg dose of an anti-Ly6G (Bio X Cell, Cat# BE0075-1; RRID:AB_1107721) or an isotype IgG (Bio X Cell, Cat# BP0090, RRID: AB_2891360).

To deplete LCN2, male C57BL/6 mice were subcutaneously inoculated with LLC cells (5 × 10^6^ per mouse) on day 0 and then were intraperitoneally injected on days 4, 7, 11, 14, 17, and 20 with a 50 μg dose of an anti-LCN2 (Novus Biologicals, Cat# AF1857, RRID:AB_355022) or an isotype IgG (Bio X Cell, Cat# BP0090, RRID: AB_2891360).

For deferoxamine (DFO) therapy, male C57BL/6 mice were subcutaneously inoculated with LLC cells (5 × 10^6^ per mouse) on day 0 and then intraperitoneally injected with 15 mg/kg DFO (Selleck, Cat#S5742) on days 4, 7, 10, 13, 16, and 19.

For liproxtatin-1 therapy, male C57BL/6 mice were subcutaneously inoculated with LLC cells (5 × 10^6^ per mouse) on day 0 and then intraperitoneally injected with 10 mg/kg liproxtatin-1 (Selleck, Cat#S7699) daily between days 1 and 20.

### Lentivirus production and delivery

The pCDH-CMV-MCS-EF1-Lcn2 (Sangon Biotech) or pCDH-CMV-MCS-EF1 vectors (YouBio, Cat# VT1479) were extracted using the Endo-Free Plasmid DNA Mini Kit II (Omega, Cat#D6950) and co-transfected with the pRSV-Rev (YouBio, Cat# VT1445), pLP/VSVG (YouBio, Cat# VT1491), and pNL-GFP-RRE (YouBio) plasmids into 293 T cells using the lipofectamine (Invitrogen, Cat#11668019) transfection method. Supernatants containing the LCN2-expressing or control lentiviruses were collected 48 and 72 h later and centrifuged at 50,000 × *g* for 2 h at 4 °C to purify the virus. For overexpression of *Lcn2*, 2 × 10^9^ plaque-forming units (PFUs) of LCN2*-*expressing lentivirus were injected intravenously into C57BL/6 mice once per week.

### Flow cytometry

Leukocytes were isolated from the epididymal and inguinal white adipose tissue (eWAT and iWAT, respectively), gastrocnemius skeletal muscle (Gast), bone marrow, and blood, as previously described [21, 22]. For intracellular staining, leukocytes were incubated with PMA (50 ng/mL), ionomycin (1 mg/mL), and monensin (10 ng/mL) for 4 h at 37 °C and 5% CO_2_, followed by staining for surface markers for 30 min at 4 °C. Cells were fixed and then permeabilized with the Foxp3/Transcription Factor Staining Buffer and incubated with fluorescent antibodies for 30 min at 4 °C. Cells were acquired by flow cytometry (LSR II).

For analysis of human blood samples, blood from cachectic cancer patients was centrifuged and lysed using red blood cell lysis buffer. Cells were then stained for surface markers for 30 min at 4 °C. Cells were fixed and then permeabilized with the Foxp3/Transcription Factor Staining Buffer and incubated with the fluorescent antibodies for 30 min at 4 °C. Cells were acquired by flow cytometry (LSR II). Antibodies and related materials used in this study are listed in Additional file 1: Table S1. Data analysis was performed using FlowJo 10 software.

### Isolation of adipocytes, neutrophils, and macrophages

eWAT samples from mice were cut into pieces and digested in DMEM with collagenase I (1 mg/mL) while shaking at 220 rpm for 30 min at 37 °C. The suspensions were filtered through sieves, and the filtrates were centrifuged at 500 × *g* for 5 min to separate the suspended adipocytes from pelleted leukocytes. Neutrophils were purified using the Neutrophil Isolation Kit (Miltenyi Biotec, Cat#130-097-658). Macrophages were purified using a PE-F4/80 antibody (eBioscience, Cat#12-4801-80; RRID:AB_465922) and anti-PE Microbeads (Miltenyi Biotec, Cat#130-048-801).

### ELISA

The concentrations of human IL-6, human CRP, human LCN2, and mouse LCN2 in cell culture supernatants and serum were measured by ELISA, according to the manufacturers’ instructions; the kits used are listed in Additional file 1: Table S1.

### Histological analysis

Tissues were fixed overnight with 10% neutral-buffered formalin, dehydrated, embedded in paraffin, and sectioned. The 4 μm slices were then stained with hematoxylin and eosin (H&E).

### Immunofluorescence

Tissues were collected as described above. Tissues were fixed overnight with 10% neutral-buffered formalin, dehydrated, embedded in paraffin, and sectioned into 4 μm slices. The slides were dewaxed, rehydrated, and subjected to heat-induced epitope retrieval, followed by incubation with 5% goat serum for 1 h at room temperature to block non-specific antibody binding. Next, the sections were incubated with a primary anti-LCN2/NGAL antibody (Abcam, Cat# ab216462) overnight at 4 °C and then with an Alexa-Fluor-546-conjugated goat anti-rabbit IgG (5 μg/mL; Invitrogen, Cat# A-11010, RRID:AB_2534077) secondary antibody. Nuclear staining was performed using DAPI (5 min incubation). The stained sections were imaged using the LSM 880 Confocal Microscope (Zeiss, Jena, Germany) and analyzed with Image J software.

### Iron assay

The concentrations of iron (Fe^2+^) in eWAT, iWAT, and Gast tissues were measured using the Iron Assay Kit (Sigma Aldrich, Cat#MAK025). eWAT, iWAT, and Gast tissues were homogenized in Iron Assay buffer by centrifugation at 16,000 × *g* for 10 min at 4 °C. The samples were then incubated with an iron reducer in a 96-well plate for 30 min at room temperature, and finally with an iron probe for 60 min at room temperature. Absorbance at 593 nm was measured using a microplate reader.

### Lipid reactive oxygen species (ROS) analysis

Adipocyte lipid ROS production was measured using the Lipid Peroxidation Assay Kit (Abcam, Cat#ab243377). Adipocytes purified from eWAT or iWAT were stained with the Lipid Peroxidation Sensor for 30 min at 37 °C and analyzed immediately by flow cytometry (LSR II). The oxidized and non-oxidized lipids were detected on the FITC and PE channels, respectively. The FITC to PE mean fluorescence intensity (MFI) ratio was calculated for each sample. Data analysis was performed using FlowJo 10 software.

### The malondialdehyde (MDA) lipid peroxidation assay

Lipid peroxidation was analyzed using the MDA Assay Kit (Sigma Aldrich, Cat#MAK085). Briefly, eWAT, iWAT, and Gast tissues were homogenized in MDA Lysis Buffer by centrifugation at 13,000 × *g* for 10 min. MDA in epididymal adipose tissue was mixed with TBA to generate the MDA-TBA adduct. Absorbance at 532 nm was measured using a microplate reader.

### Quantitative (q)PCR assays

RNA was extracted from purified cells or frozen tissue samples using the TRIzol reagent (Invitrogen, Cat#15596018), glycogen (Thermofisher, Cat#AM9515), and sodium acetate (Thermofisher, Cat#R1181). RNA was reverse transcribed into cDNA using the M-MLV Reverse Transcriptase (Invitrogen, Cat#28025013). qPCR was then performed using the SYBR Green Premix Pro Taq HS qPCR Kit (Accurate biology, Cat#AG11701) on a LightCycler 96 instrument (Roche). PCR was performed using the 2 × TransTaq-T PCR SuperMix (Transgen, Cat#AS122). Relative mRNA levels were calculated using the 2^−ΔΔCt^ method and normalized to actin mRNA levels. Target gene primers are shown in Additional file 1: Table S2.

### RNA sequencing (seq)

Total RNA was extracted from the eWAT of cachectic and control mice using the miRNeasy Mini Kit and treated with DEPC-treated water. A total amount of 1 µg RNA per sample was used as input material for the RNA sample preparations. RNA libraries were prepared for sequencing using the NEBNext UltraTM RNA Library Prep Kit (Illumina). Clustering of the index-coded samples was performed on a cBot Cluster Generation System using TruSeq PE Cluster Kit v3-cBot-HS (Illumina), according to the manufacturer’s instructions. After cluster generation, the library preparations were sequenced on an Illumina Novaseq platform and 150 bp paired-end reads were generated. Raw data (raw reads in fastq format) were firstly processed through in-house perl scripts. Clean data were obtained by removing reads containing adapter and poly-N sequences, and low-quality reads. The reference genome and gene model annotation files were downloaded from the genome website directly. The reference genome index was built using Hisat2 software, and paired-end clean reads were aligned to the reference genome. FeatureCounts software was used to count the read numbers mapped to each gene. Fragments per kilobase of exon model per million reads mapped (FPKM) were calculated for each gene based on the length of the gene and the read counts mapped to this gene. Differential expression analysis of two groups was performed using the DESeq2 R package (1.16.1). The resulting P-values were adjusted using the Benjamini and Hochberg’s approach to control for the false discovery rate. Genes with a DESeq2-derived adjusted *P*-value < 0.05 and log2fold change (FC) ≥ 2 were labelled as differentially expressed genes (DEGs; listed in Additional file 5: Table S6). The DEG heatmap was analyzed using MEV software Gene Ontology (GO) enrichment analysis of DEGs was implemented using the clusterProfiler R package. GO terms with a corrected *P*-value < 0.05 were considered significantly enriched DEGs. We used clusterProfiler R package to test the statistical enrichment of DEGs in various KEGG enrichment pathways. For the Gene Set Enrichment Analysis (GSEA), the genes were ranked according to the degree of differential expression in the two samples being compared. The predefined Gene Sets were then tested to see if they were enriched at the top or bottom of the list. The RNA-seq data generated in this study were deposited in the GEO database repository and can be accessed using with the accession number GSE188479.

### Serum protein array

The relative expression levels of human serum proteins in lung cancer patients with and without cachexia and in healthy donors were measured using the G-Series Human Cytokine Antibody Array 440 (RayBiotech). The relative expression levels of mouse serum proteins in cachectic and control mice were determined using the Quantibody® Mouse Cytokine Antibody Array 4000 (RayBiotech). Fluorescence signals were visualized in the Cy3 channel of a laser scanner. Data were extracted using the GAL file from www.RayBiotech.com/Gal-Files.html. Heatmaps of differentially expressed proteins (DEPs) were analyzed using MEV software.

### Western blotting

Purified adipocytes, neutrophils, and macrophages were lysed in RIPA buffer (Beyotime, Cat#P0013B) containing protease inhibitors on ice for 30 min, followed by centrifugation at 16,000 × *g* for 10 min at 4 °C. Protein concentrations in the supernatants were measured using the BCA Protein Assay Kit (Thermofisher, Cat#23225), and 50 μg aliquots of proteins were incubated at 95 °C for 10 min and separated on SDS-PAGE gels. The proteins were transferred to PVDF membranes, which were blocked in a solution of 5% milk in TBS buffer containing 0.1% Tween-20 for 1 h at room temperature. The membranes were then incubated with primary antibodies overnight at 4 °C and subsequently with HRP-conjugated secondary antibodies for 1 h at room temperature. The protein bands were detected by chemiluminescence autoradiography. The antibodies used are listed in Additional file 1: Table S1.

### Statistical analysis

All statistical analyses were performed using GraphPad Prism 5.0 (GraphPad Software, La Jolla, CA, USA). Results are presented as the mean ± standard error of the mean (SEM) and were compared using unpaired t-tests, one-way ANOVA, or two-way ANOVA. Mouse survival was estimated using the Kaplan–Meier method and compared using the log-rank test. Patient sample data were compared using the Wilcoxon signed rank test and Spearman’s rho was calculated as indicated.

## Results

### The LCN2 level is increased in wasting tissues in lung cancer cachexia

**Fig. 1:**
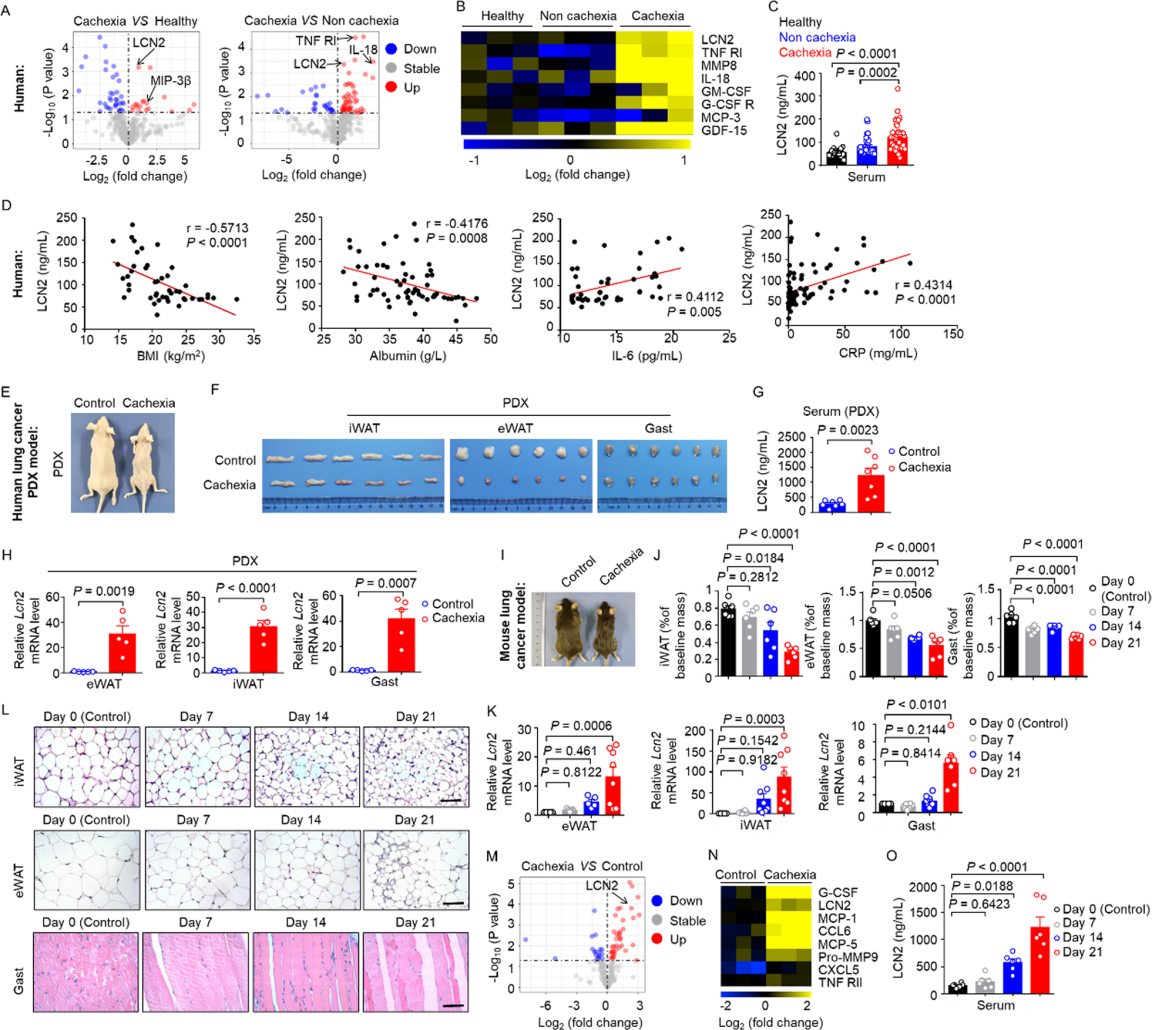
LCN2 is highly expressed in wasting tissues in lung cancer cachexia. **A**. Volcano plot of differentially expressed proteins (DEPs) between the sera of cancer patients with cachexia and healthy controls, and non-cachectic lung cancer patients. n = 3 for each group. **B**. Heatmap showing the normalized levels of serum inflammatory proteins in lung cancer patients with cachexia *vs*. the levels in lung cancer patients without cachexia *vs* healthy controls. n = 3 for each group. **C**. Serum LCN2 concentrations in lung cancer patients with (n = 33) or without (n = 37) cachexia and in healthy controls (n = 32). **D**. Negative correlation between the LCN2 level, the body mass index (BMI), and the serum albumin concentration; and positive correlation between the LCN2 level and the serum concentrations of IL-6 and CRP. The Spearman correlation coefficient (r) and P-value are shown. **E**. Representative images of PDX-induced cachectic model mice and control mice. **F**. Representative images of iWAT, eWAT, and Gast. **G**. Serum LCN2 concentrations in mice with PDX-induced cachexia (n = 7) and control mice (n = 7). **H**. The *Lnc2* mRNA levels in eWAT, iWAT, and Gast tissues for the indicated mice, assessed by qPCR. n = 5 per group. **I–O**. Experiments using a classic model of murine cachexia. Mice were inoculated with murine lung cancer cells (the LLC cell line) and monitored for up to 21 days. **I**. Representative images of cachectic and control mice. **J.** Weight of eWAT, iWAT, and Gast in these mouse groups. n = 6 per group. **K**. qPCR determination of *Lcn2* mRNA levels in eWAT, iWAT and Gast. n = 5–8 per group. **L**. Representative H&E staining of iWAT, eWAT, and Gast. Scale bars, 100 μm. **M**. Volcano plot of DEPs in the sera of cachectic and control mice. **N.** Heatmap showing the normalized expression of the indicated inflammatory proteins in mice with cachexia compared with control mice. **O**. Monitoring of serum LCN2 concentrations for up to 21 days in mice bearing LLC tumors. n = 6 per group. Data are shown as the mean ± SEM. Statistical analyses were performed using one-way ANOVA (**C, J, K, O)** or unpaired Student’s t-tests (**G, H)**

We wished to identify the drivers of lung cancer cachexia in humans. Hence, we used a proteomics approach to evaluate the profiles of serum protein factors in cachectic lung cancer patients *vs.* non-cachectic lung cancer patients or healthy controls. This approach revealed aberrantly higher LCN2 levels in lung cancer patients with cachexia than in non-cachectic lung cancer patients or healthy controls (Fig. 1A, B). Levels of proinflammatory cytokines (*e.g.,* IL-6 and CRP) were also higher in the serum of cachectic patients than in the other two groups, thereby indicating that cachexia was associated with a highly inflammatory environment (Fig. 1B and Additional file 1: Fig. S1A). Consistent with our data on the profiles of protein factors, ELISA results showed that serum concentrations of LCN2 were higher in cachectic lung cancer patients than in non-cachectic lung cancer patients or healthy controls (Fig. 1C). Moreover, we detected a negative correlation between the serum LCN2 concentration and the BMI and serum albumin level (Fig. 1D). We also detected a positive correlation between the serum LCN2 concentration and the IL-6 level and CRP level (Fig. 1D).

To pursue the basis of these clinical observations experimentally, we followed a previously reported [23] approach to establish a lung cancer PDX mouse model of cachexia (Fig. 1E, F). The tumor-tissue source of the PDX cachexia model was from a patient with lung adenocarcinoma (number: 3#-Ade) and all mice were able to develop cachexia. First, our ELISA data showed that the serum concentration of LCN2 was significantly higher in cachexia model mice than in control mice (*i.e.*, mice without PDX-based induction of cachexia) (Fig. 1G). In addition, after sacrificing the mice, a qPCR analysis showed significantly increased *Lcn2* expression in the tissues (eWAT, iWAT, and Gast) of cachexia model mice (Fig. 1H); eWAT, iWAT, and Gast undergo atrophy in cachexia [24, 25].

Subsequently, we induced a classic experimental model of murine cachexia [20] based on the subcutaneous inoculation of mice with LLC cells. As expected, 100% of LLC tumor-bearing mice developed cachexia symptoms. Over the 3-week observation period, tumor-bearing (*i.e.*, cachectic) mice experienced significant weight loss *vs.* control mice (Fig. 1I). Upon sacrificing the mice, we detected wasting of eWAT, iWAT, and Gast in cachectic mice (Fig. 1J and Additional file 1: Fig. S1B). We also detected dystrophic pathological changes in the iWAT (Fig. 1L, top), eWAT (Fig. 1L, middle), and Gast (Fig. 1L, bottom) of tumor-bearing mice during cachexia progression.

A proteomics-based analysis of the profiles of serum protein factors in lung cancer cachectic mice showed significantly higher LCN2 levels in cachectic mice than in control animals (Fig. 1M, N). ELISA results further supported the significantly increased serum LCN2 concentrations in cachectic mice compared with those in control mice (Fig. 1O). In addition, we observed aberrantly increased LCN2 (mRNA and protein) levels during cachexia progression in the eWAT, iWAT, and Gast of cachectic mice (Fig. 1K and Additional file 1: Fig. S1E), as well as stronger LCN2 protein signals (immunofluorescence staining) in the iWAT and Gast of cachectic mice (Additional file 1: Fig. S1C, D). Collectively, these findings implied that the pathological changes which affected the wasting tissues of lung cancer cachexia models were related to the increased LCN2 level observed in lung cancer patients suffering from cachexia.

### Ferroptosis occurs in wasting tissues in lung cancer cachexia

**Fig. 2:**
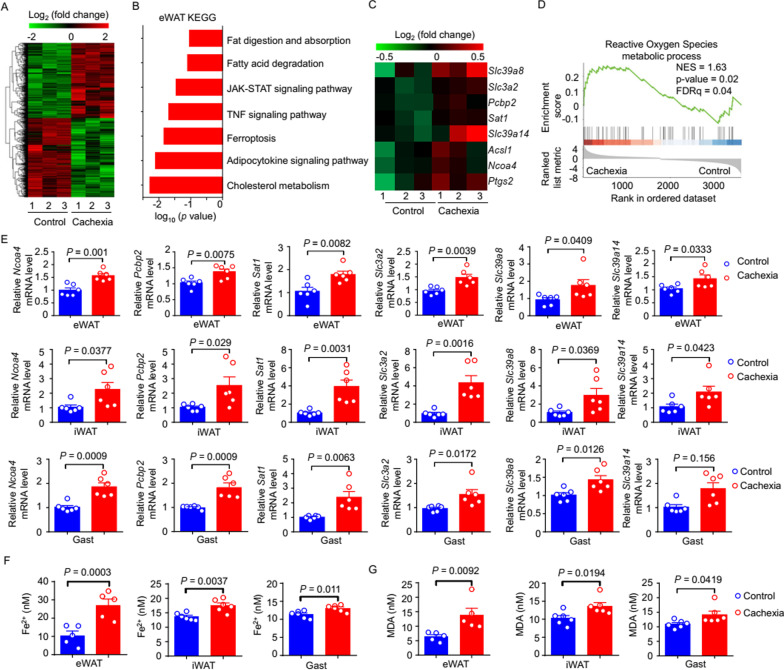
Ferroptosis occurs in the wasting tissues of mice with lung cancer cachexia. **A**. Mice were inoculated with LLC cells to induce the murine cachexia, and eWAT tissues from cachectic and control mice were harvested on day 21 and analyzed by RNA-seq; this panel shows a heatmap of differentially expressed genes (DEGs) in the eWAT tissue. **B**. KEGG pathway enrichment analysis. **C**. Heatmap analysis showing the normalized expression of ferroptosis-related genes. **D**. Gene set enrichment analysis (GSEA) showing that metabolic processes involving reactive oxygen species were enriched in the cachectic mice compared with controls. **E**. qPCR analysis of the indicated ferroptosis gene mRNA levels in the eWAT, iWAT, and Gast. n = 6 per group. **F**. Chemiluminescence analysis of Fe^2+^ concentrations in the eWAT, iWAT, and Gast. n = 5–6 per group. **G**. Chemiluminescence analysis of MDA concentration in the eWAT, iWAT, and Gast. n = 5–6 per group. Data are shown as the mean ± SEM. Statistical analyses were performed using unpaired Student’s t-tests (**E, F, G)**

To assess the pathological transformations that occur in the microenvironment of local wasting tissue during lung cancer cachexia, we assessed changes in the wasting tissues of LLC-implanted cancer cachectic mice at the transcriptomic level. Among the 3,624 DEGs identified in our comparison of the eWAT from cachectic mice and control mice, 1770 showed upregulated expression and 1,854 had downregulated expression in cachectic mice (Fig. 2A). Enrichment analysis using the KEGG database revealed that the DEGs with upregulated expression were enriched in pathways such as “fatty acid degradation”, “inflammation”, and “ferroptosis” (Fig. 2B).

Ferroptosis is a form of cell death that results from iron-dependent accumulation of lipid peroxide and ROS production [26, 27]. GSEA showed enrichment of the ROS signature (Fig. 2D). Pursuing ferroptosis-related genes specifically, RNA-seq data and follow-up qPCR analysis showed that expression of *Ncoa4*, *Slc39a8*, *Slc3a2*, *Pcbp2*, *Slc39a14*, and *Sat1* [28] was significantly higher in the wasting eWAT, iWAT, and Gast of cachectic mice than in the equivalent tissues in control mice (Fig. 2C, E). *Ptgs2* expression [29] was significantly higher in the wasting eWAT of tumor-bearing mice than in the eWAT of control animals (Fig. 2C).

Tissues were harvested 21 days after tumor inoculation. We also assessed the known biochemical features of ferroptosis [26]. Levels of Fe^2+^, MDA, and lipid ROS were significantly higher in wasting eWAT, iWAT, and Gast than in the equivalent normal tissues (Fig. 2F, G and Additional file 1: Fig. S2B). Finally, immunofluorescence and flow-cytometry analyses of fresh adipocytes purified from wasting eWAT and normal eWAT showed that wasting eWAT exhibited increased lipid peroxidation (Additional file 1: Fig. S2C) and had a higher proportion of adipocytes (Additional file 1: Fig. S2A) than those in the eWAT of controls.

We also examined a lung cancer PDX-induced mouse model of cachexia, and found that Fe^2+^ and MDA levels were significantly higher in wasting eWAT, iWAT, and Gast than in normal tissues (Additional file 1: Fig. S2D, E). We also detected significantly higher expression of ferroptosis-related genes in the wasting eWAT, iWAT, and Gast of cachectic mice than in the equivalent normal tissues of control animals (Additional file 1: Fig. S2F). These results suggested that, in lung cancer cachexia, wasting tissues undergo ferroptosis.

### Exogenous LCN2 induces ferroptosis and tissue wasting in mice

**Fig. 3:**
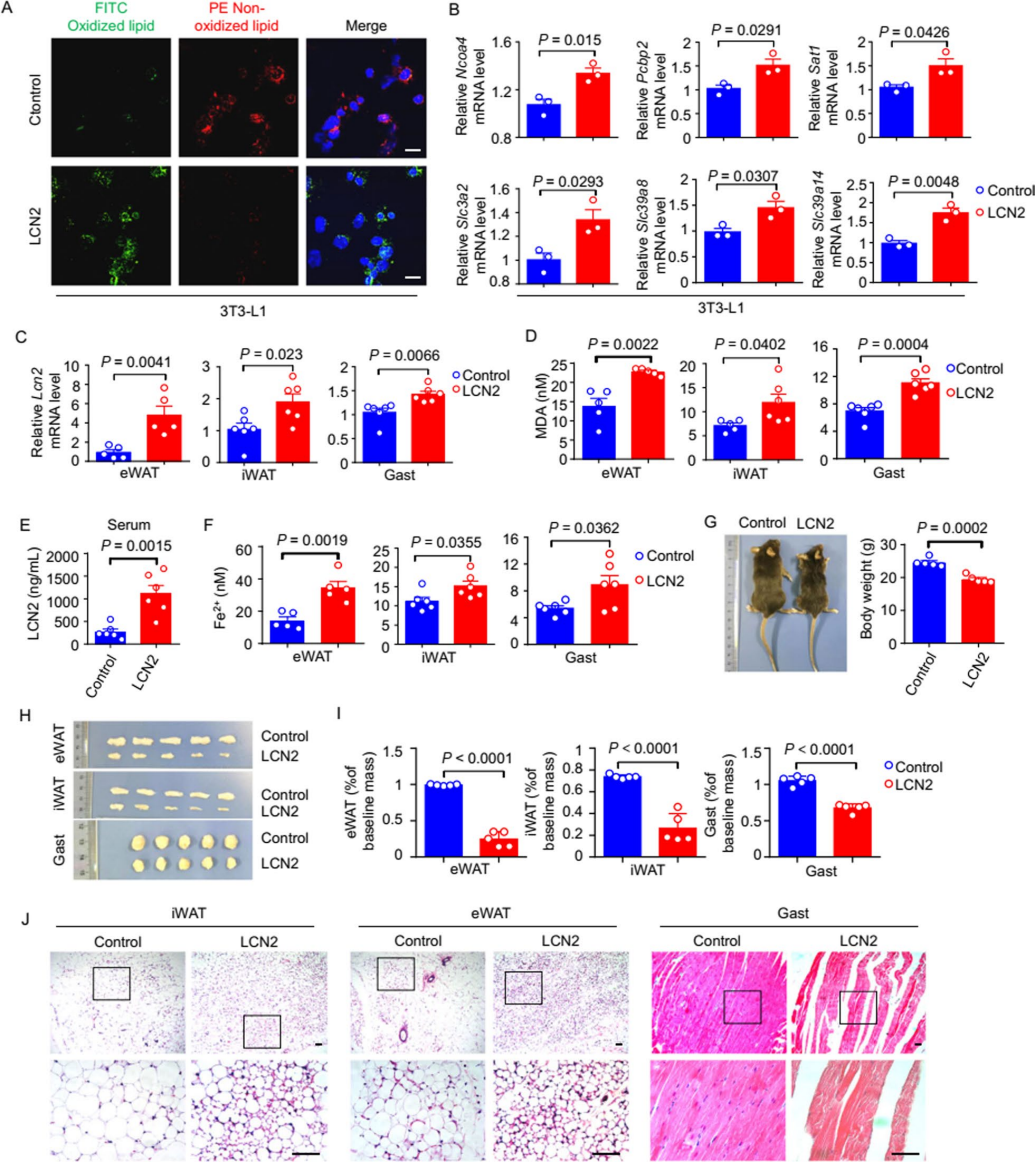
LCN2 overexpression promotes tissue ferroptosis and wasting. **A**, **B**. 3T3-L1 cells were treated with LCN2 for 24 h. **A**. Representative immunofluorescence staining of lipid peroxidation. Scale bars, 50 μm. **B**. qPCR analysis of mRNA levels of the indicated ferroptosis-related genes in 3T3-L1 cells. n = 3 per group. **C–J**. Mice were injected intravenously with the control or LCN2-overexpressing lentivirus, and tissues were harvested on day 21. **C**. qPCR measurements of *Lcn2* mRNA levels in the eWAT, iWAT, and Gast. n = 5–6 per group. **D**. MDA concentrations in the eWAT, iWAT, and Gast, assessed using chemiluminescence. n = 5–6 per group. **E**. ELISA evaluation of serum LCN2 concentrations. n = 6 per group. **F.** Fe^2+^ concentrations in eWAT, iWAT, and Gast, assessed by chemiluminescence. n = 5–6 per group. **G**. Representative images of mice injected with the control or LCN2-overexpressing lentivirus. Body weights of mice injected with the control or LCN2-overexpressing lentivirus. n = 5 per group. **H**. Representative image of the eWAT, iWAT, and Gast from mice injected with the control or LCN2-overexpressing lentivirus. **I**. Weights of the eWAT, iWAT, and Gast isolated from mice injected with the control or LCN2-overexpressing lentivirus. n = 5 per group. **J**. Representative H&E staining of the iWAT, eWAT, and Gast from mice injected with the control or LCN2-overexpressing lentivirus. Scale bars, 100 μm. Data are shown as the mean ± SEM. Statistical analyses were performed using the unpaired Student’s t-tests **(B–E, F, G, I)**

To investigate the potential impact of the LCN2 level on ferroptosis, we conducted in vitro experiments with 3T3-L1 adipocytes [30]. Briefly, addition of the recombinant murine Lcn2 protein to 3T3-L1 cells increased the extent of cell death, lipid peroxidation (Fig. 3A and Additional file 1: Fig. S3E), and expression of ferroptosis-related genes (*Ncoa4, Slc3a2, Slc39a8, Slc39a14, Pcbp2, Ptgs2,* and *Sat1*) significantly (Fig. 3B and Additional file 1: Fig. S3H). We also treated 3T3-L1 cells with a ferroptosis inhibitor, liproxstatin-1 (after treatment with recombinant Lcn2 protein), and found that it alleviated the increase in MDA or lipid ROS levels triggered by Lcn2 (Additional file 1: Fig. S3F, G). These results indicated that LCN2 could induce ferroptosis in cultured adipocytes.

Next, we evaluated the potential functional contributions of LCN2 to ferroptosis and tissue wasting in vivo by developing mice overexpressing LCN2. To achieve this, we injected (i.v.) wild-type C57BL/6 mice with 2 × 10^9^ PFUs of an LCN2-expressing lentivirus [31]. mRNA expression of *Lcn2* was increased significantly in the eWAT, iWAT, and Gast of mice injected with the LCN2-expressing lentivirus compared with that in the tissues of mice injected with the empty control lentivirus (Fig. 3C); protein expression of LCN2 was also increased significantly, considerably exceeding the serum concentration of endogenous LCN2 (Fig. 3E). In addition, the concentrations of MDA (Fig. 3D) and Fe^2+^ (Fig. 3F) were significantly higher in the eWAT, iWAT, and Gast of LCN2-overexpressing mice than in those of control mice. Moreover, expression of ferroptosis-related genes was increased significantly in the eWAT, iWAT, and Gast of LCN2-overexpressing mice than in those of control mice (Additional file 1: Fig. S3D).

LCN2-overexpressing mice also showed a significant reduction in body weight (Fig. 3G), as well as wasting of adipose tissues (eWAT and iWAT) and skeletal muscle (Gast) (Fig. 3H, I). Histology revealed the clearly aberrant morphology of the iWAT and eWAT of LCN2-overexpressing mice (Fig. 3J); the skeletal muscle of these mice was also pathologically altered markedly (Fig. 3J). In addition, the eWAT and iWAT of mice injected with the LCN2-expressing lentivirus expressed higher levels of thermogenic and lipolytic genes (Additional file 1: Fig. S3A, B), whereas the Gast of these mice expressed higher levels of genes involved in protein degradation (Additional file 1: Fig. S3C). Collectively, our results from these narrowly focused experimental models revealed that LCN2: (i) induced ferroptosis in adipocytes; (ii) contributed specifically and functionally to the observed wasting of WAT and muscle observed initially in cachexia model mice.

### TI-Neu cells are a major source of LCN2 in wasting tissues in lung cancer cachexia

**Fig. 4:**
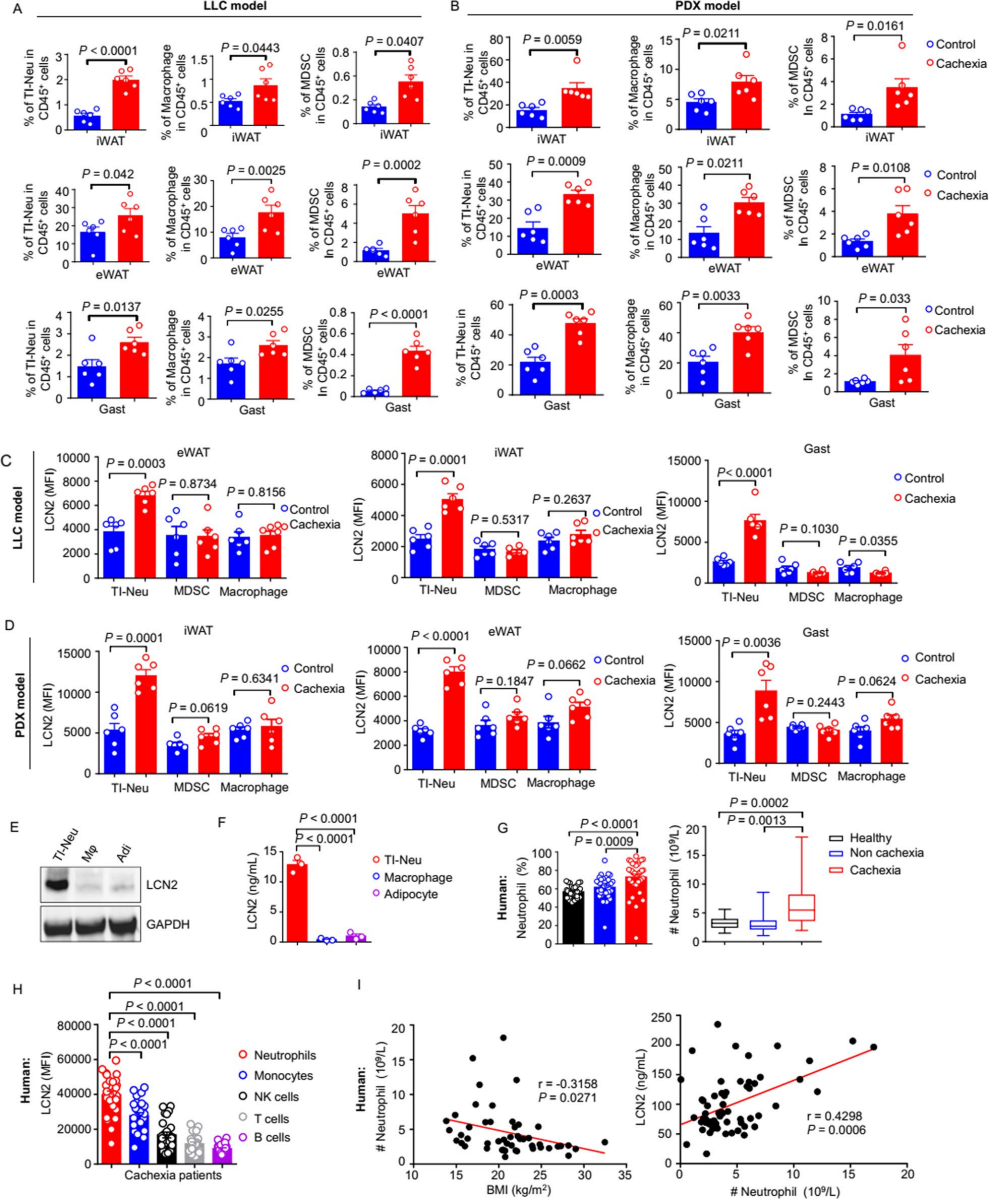
TI-Neu cells are a major source of LCN2 in wasting tissues in lung cancer cachexia.** A**. Mice were inoculated with LLC cells to induce cachexia. Proportions of neutrophils, macrophages, and MDSCs in the eWAT, iWAT, and Gast were assessed. n = 6 per group. **B**. Proportions of neutrophils, macrophages, and MDSCs in the eWAT, iWAT, and Gast of PDX-induced cachectic model mice and non-model control mice. n = 6 per group. **C**. MFI values for LCN2 in the TI-Neu cells, macrophages, and MDSCs of the eWAT, iWAT, and Gast. n = 6 per group. **D**. Flow cytometry LCN2 MFI values in the TI-Neu cells, macrophages, and MDSCs of the eWAT, iWAT, and Gast of PDX-induced cachectic model mice and control mice. n = 6 per group. **E–F**. Mice were inoculated with LLC cells and the tissues were harvested on day 21. Adipocytes, TI-Neu cells, and macrophages were purified from the eWAT. **E**. Western blotting for LCN2 in purified adipocytes, TI-Neu cells, and macrophages. **F**. LCN2 concentrations in the supernatants of purified adipocytes, TI-Neu cells, and macrophages (cultured in medium for 18 h). Adi, adipocyte. Mφ, macrophage. **G**. Proportions and absolute numbers of neutrophils in the peripheral blood of lung cancer patients with (n = 34) or without (n = 34) cachexia, and healthy controls (n = 32). **H**. MFI values for LCN2 in neutrophils, monocytes, NK cells, T cells, and B cells obtained from the peripheral blood of lung cancer patients with cachexia (n = 26). **I**. Negative correlation between the number of neutrophils and BMI, and positive correlation between the number of neutrophils and the LCN2 level. Data are shown as the mean ± SEM. Statistical analyses were performed using one-way ANOVA (**F–H)** or unpaired Student’s t-tests (**A–D)**

Next, we investigated the source of LCN2 in wasting tissues. Notably, LCN2 is a neutrophil gelatinase-associated lipocalin, and has been reported to act as a pleiotropic mediator in several inflammatory and metabolic diseases [32]. The eWAT, iWAT, and Gast of LLC lung cancer cachectic mice had significantly higher numbers of TI-Neu cells (Fig. 4A and Additional file 1:Fig. S4B, E), macrophages, and myeloid-derived suppressor cells (MDSCs) that the equivalent tissues of control mice (Fig. 4A and Additional file 1:sFig.4C, D, E).

We also conducted flow-cytometry analyses of the eWAT, iWAT, and Gast from LLC lung cancer cachectic model mice: TI-Neu cells, macrophages, and MDSCs all expressed LCN2 protein. Moreover, the LCN2 level was higher in the TI-Neu cells derived from the wasting eWAT, iWAT, and Gast of cachectic mice than from control animals. In contrast, the LCN2 levels in eWAT-, iWAT-, and Gast-derived MDSCs and macrophages did not differ between the experimental lung cancer cachexia mice and controls (Fig. 4C). Notably, the LCN2 level was very low in the lymphocytes (e.g., T, B, and natural killer [NK] cells) of cachectic mice and control mice (Additional file 1: Fig. S4G). Furthermore, neutrophils were the largest population of LCN2-positive immune cells (LCN2 + CD45 + cells) (Additional file 1: Fig. S4H). Co-localization of LCN2 and neutrophils (MPO +) in the adipose tissue of cachectic mice was observed (Additional file 1: Fig. S4I), indicating that TI-Neu cells were the major source of LCN2. To garner additional support for the conclusions inferred from the LLC cancer cachexia model, we examined a lung cancer PDX-induced mouse model of cachexia. The two models elicited similar results (Fig. 4B, D).

LCN2 secretion from bone-marrow neutrophils has been reported to mediate appetite suppression during pancreatic cancer cachexia [12]. Similarly, we found that the LCN2 levels in neutrophils derived from the bone marrow and blood were higher in cachectic mice than in control animals (Additional file 1: Fig. S4F). Our evaluation of cachectic mice also showed that the MFI of LCN2 was much higher in WAT neutrophils than in neutrophils originating from the bone marrow or blood (Additional file 1: Fig. S4F). Next, we measured the LCN2 levels in adipocytes, TI-Neu cells, and macrophages purified from the wasting adipose tissue of cachectic mice. The LCN2 level was markedly higher in the TI-Neu cells than in the adipocytes or macrophages of wasting eWAT (Fig. 4E). Then, we cultured these three cell types individually. ELISA of the culture supernatants showed that the LCN2 level was obviously increased in TI-Neu cells but not in the other two cell types (Fig. 4F).

Analysis of the peripheral blood from lung cancer cachectic patients revealed two main phenomena. First, the proportion and absolute number of circulating neutrophils were higher in cancer patients with cachexia than in cancer patients not suffering from cachexia or in healthy controls (Fig. 4G). Second, the neutrophils of cancer patients with cachexia had significantly higher LCN2 levels than other immune cells (monocytes, T cells, NK cells, and B cells) (Fig. 4H). Furthermore, the number of neutrophils in the blood of cancer patients suffering from cachexia correlated positively with their LCN2 level, but negatively with their BMI (Fig. 4I). Taken together, these results showed that TI-Neu cells from wasting tissues were a major source of LCN2.

### Neutrophil depletion prevents ferroptosis and tissue wasting in lung cancer cachexia

**Fig. 5:**
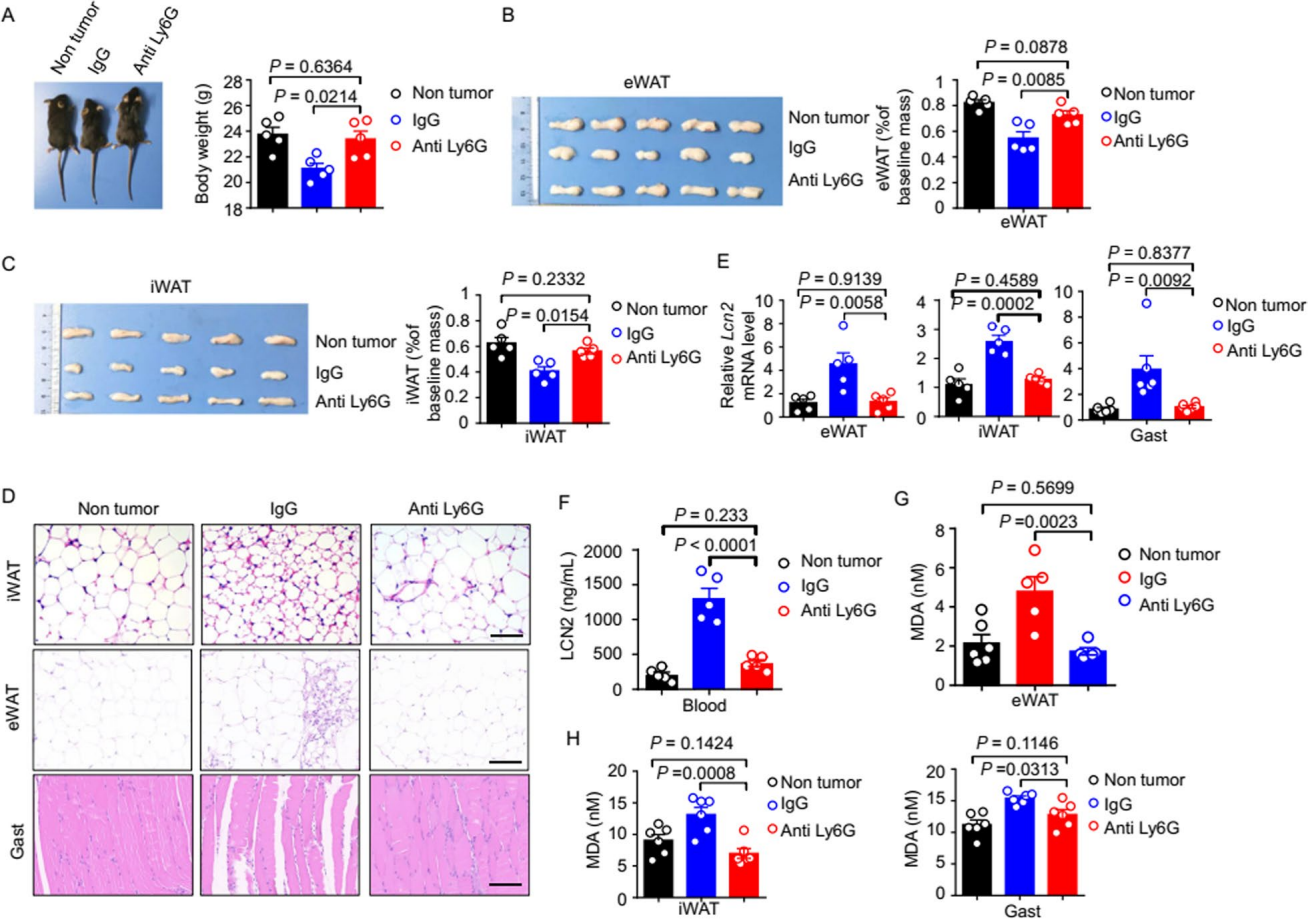
Depletion of TI-Neu cells prevents tissue ferroptosis and wasting in lung cancer cachexia. Mice were inoculated subcutaneously with LLC cells to induce cachexia and were treated with 100 μg anti-Ly6G antibody (or IgG control) three times per week between days 1 and 20; the tissues were harvested on day 21. **A**. Representative images (left) and body weights (right) of the lung cancer cachexia model mice. n = 5 per group. **B**. Representative images (left) and weights (right) of the eWAT. n = 5 per group. **C**. Representative images (left) and weights (right) of the iWAT. n = 5 per group. **D**. Representative H&E staining of the iWAT, eWAT, and Gast. Scale bars, 100 μm. **E**. qPCR analysis of *Lcn2* mRNA levels in the eWAT, iWAT, and Gast. n = 5 per group. **F**. Serum LCN2 concentrations. n = 5 per group. **G.** MDA concentrations in the eWAT. n = 5–6 per group. **H**. MDA concentrations in the iWAT and Gast. n = 6 per group. Data are shown as the mean ± SEM. Statistical analyses were performed using the one-way ANOVA (**A–C, E–H)**

To determine if elimination of TI-Neu cells from mice with lung cancer cachexia could alleviate tissue wasting and ferroptosis, we treated model mice with a neutrophil-depleting, anti-Ly6G antibody (Additional file 1: Fig. S5A). As expected, treatment with the anti-Ly6G antibody reduced the number of neutrophils in the blood and adipose tissue of model mice significantly compared with treatment with the control (IgG) (Additional file 1: Fig. S5B–E). Strikingly, the body weight of neutrophil-depleted lung cancer cachectic model mice was significantly higher than that of IgG control-treated animals; there was no significant difference between the body weight of control mice and neutrophil-depleted cachexia model animals (Fig. 5A).

Neutrophil depletion alleviated the wasting of eWAT and iWAT significantly (Fig. 5B, C). Moreover, histology showed that neutrophil depletion alleviated the pathological morphology of iWAT, eWAT, and Gast observed in cachexia model mice (Fig. 5D) and also reduced the LCN2 levels in the eWAT, iWAT, Gast, and serum significantly (Fig. 5E, F). Neutrophil depletion reduced the MDA concentrations in iWAT, eWAT, and Gast significantly (Fig. 5G, H) and alleviated the increase in Fe^2+^ concentrations in iWAT and Gast compared with those in IgG control-treated mice (Additional file 1: Fig. S5F). Next, we measured mRNA expression of ferroptosis-related genes. The increase in expression of these genes was alleviated significantly in the eWAT, iWAT, and Gast of anti-Ly6G-antibody-treated mice compared with those in IgG control-treated animals (Additional file 1: Fig. S5G). Collectively, these results showed that the targeted elimination of neutrophils alleviated tissue wasting in lung cancer cachexia model mice significantly.

### LCN2 knockout reduces ferroptosis and tissue wasting in lung cancer cachexia

**Fig. 6:**
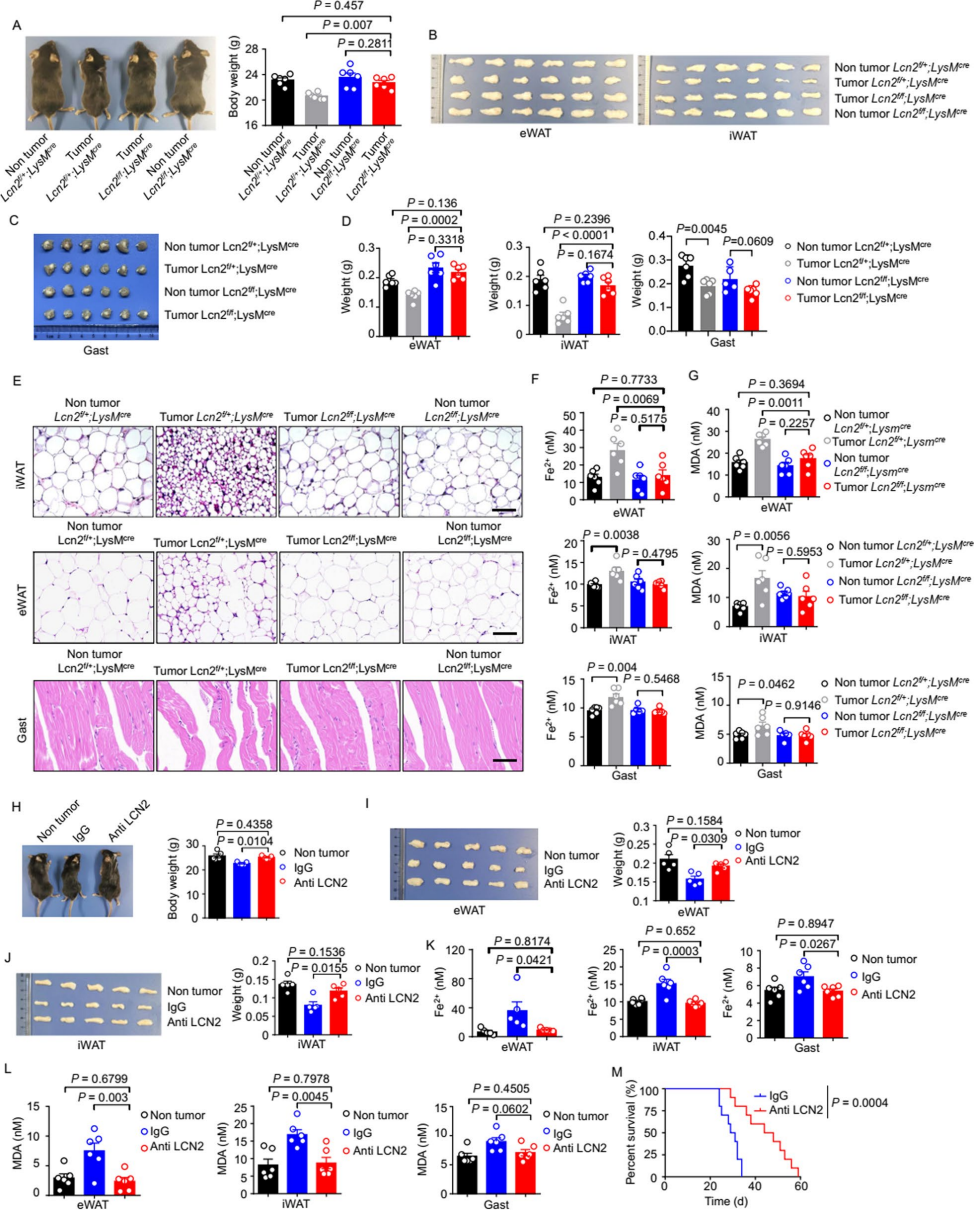
*LCN2* knockout reduces tissue ferroptosis and wasting in lung cancer cachexia. **A–G**. *Lcn2*^*f/*+^*;LysM*^*cre*^ and *Lcn2*^*f/f*^*;LysM*^*cre*^ mice were injected with LLC cells to induce the lung cancer cachexia model, and the tissues were harvested on day 21. **A**. Representative images and body weights of *Lcn2*^*f/*+^*;LysM*^*cre*^ and *Lcn2*^*f/f*^*;LysM*^*cre*^ lung cancer cachexia model mice and their respective controls. Representative images of eWAT, iWAT (**B**), and Gast (**C**) from the *Lcn2*^*f/*+^*;LysM*^*cre*^ and *Lcn2*^*f/f*^*;LysM*^*cre*^ lung cancer cachexia model mice and controls. n = 5–6 per group. **D**. Weights of the eWAT, iWAT, and Gast of *Lcn2*^*f/*+^*;LysM*^*cre*^ and *Lcn2*^*f/f*^*;LysM*^*cre*^ lung cancer cachexia model mice and controls. n = 5–6 per group. **E.** Representative images of the iWAT, eWAT, and Gast of *Lcn2*^*f/*+^*;LysM*^*cre*^ and *Lcn2*^*f/f*^*;LysM*^*cre*^ lung cancer cachexia model mice and controls. H&E staining; scale bars, 100 μm. Analysis of **(F)** Fe^2+^ concentration, **(G)** MDA concentration in the eWAT, iWAT, and Gast of *Lcn2*^*f/*+^*;LysM*^*cre*^ and *Lcn2*^*f/f*^*;LysM*^*cre*^ lung cancer cachexia model mice and controls. n = 5–6 per group. **H–M**. Lung cancer cachexia model mice were injected with 50 μg IgG or anti-LCN2 antibody every 3 days between days 4 and 20, and the tissue samples were harvested on day 21. **H**. Representative images (left) and body weights (right) of control mice, neutrophil-depleted lung cancer cachexia model mice, and control IgG-injected lung cancer cachexia model mice. n = 5 per group. **I**. Representative images (left) and weights (right) of the eWAT. n = 5 per group. **J**. Representative images (left) and weights (right) of the iWAT. n = 5 per group. **K**. Fe^2+^ (n = 5–6 per group) concentrations in the eWAT, iWAT, and Gast. **L**. MDA (n = 6 per group) concentrations in the eWAT, iWAT, and Gast. **M**. Kaplan–Meier analysis of mouse survival (n = 10 per group), with comparisons performed using the log-rank test. Statistical analyses were performed using the one-way ANOVA **(A, D, F, G, H–L)** or the log-rank test (**O)**. Data are shown as the mean ± SEM

Next, we examined the impact of knocking out *LCN2* by generating myeloid-specific *Lcn2* knockout (*Lcn2*^*f/f*^*LysM*^*cre*^) mice (Additional file 1: Fig. S6A) and inducing lung cancer cachexia in these mice and their control (*Lcn2*^*f/*+^*LysM*^*cre*^) littermates. Induction of lung cancer cachexia led to the expected increase in protein expression of LCN2 in the serum of *Lcn2*^*f/*+^*LysM*^*cre*^ mice, but not in *Lcn2*^*f/f*^*LysM*^*cre*^ mice (Additional file 1: Fig. S6B). As expected, *Lcn2*^*f/*+^*LysM*^*cre*^, but not *Lcn2*^*f/f*^*LysM*^*cre*^ mice, exhibited weight loss (Fig. 6A), significant wasting of eWAT, iWAT, and Gast (Fig. 6B–D), and pathological morphology of eWAT, iWAT, and Gast (Fig. 6E). Examination of the biochemical features of ferroptosis showed that Fe^2+^ and MDA concentrations were increased significantly in the eWAT, iWAT, and Gast of *Lcn2*^*f/*+^*LysM*^*cre*^, but not in those of *Lcn2*^*f/f*^*LysM*^*cre*^ mice (Fig. 6F, G). Moreover, analyses of the lipid ROS of fresh adipocytes purified from eWAT showed lipid ROS levels to be increased significantly in *Lcn2*^*f/*+^*LysM*^*cre*^ mice, but not in *Lcn2*^*f/f*^*LysM*^*cre*^ mice (Additional file 1: Fig. S6H). Taken together, these findings suggested that myeloid-derived LCN2 contributed specifically to the ferroptotic tissue wasting observed in lung cancer cachexia model mice.

Given our findings regarding *LCN2* knockout, next we explored if treating mice with an anti-LCN2 antibody to achieve targeted elimination of LCN2 conferred similarly protective effects in lung cancer cachexia model mice (Additional file 1: Fig. S6C). Upon administration of the anti-LCN2 antibody (or IgG control; both 50 μg per mouse), we observed the expected reductions in eWAT, iWAT, and serum LCN2 level (Additional file 1: Fig. S6D–E). Furthermore, treatment with the anti-LCN2 antibody alleviated the pathogenic phenotypes of lung cancer cachexia model mice, including the reduction in body weight (Fig. 6H) and eWAT/iWAT wasting (Fig. 6I, J). Histology showed that treatment with anti-LCN2 antibody also alleviated the pathological morphology of iWAT and Gast of cachexia model mice (Additional file 1: Fig. S6F, G). Biochemical analysis showed that lung cancer cachexia model mice treated with anti-LCN2 antibody had significantly lower Fe^2+^ and MDA concentrations in their eWAT, iWAT, and Gast than those in controls (Fig. 6K, L). Moreover, anti-LCN2 antibody-treated mice lived significantly longer (Fig. 6M). Collectively, the findings from studies on myeloid-specific knockout and therapy with anti-LCN2 antibody demonstrated that targeted elimination of LCN2 alleviated ferroptosis and tissue wasting significantly in mice with lung cancer cachexia.

### Chemical inhibition of ferroptosis reduces tissue wasting in lung cancer cachexia

**Fig. 7:**
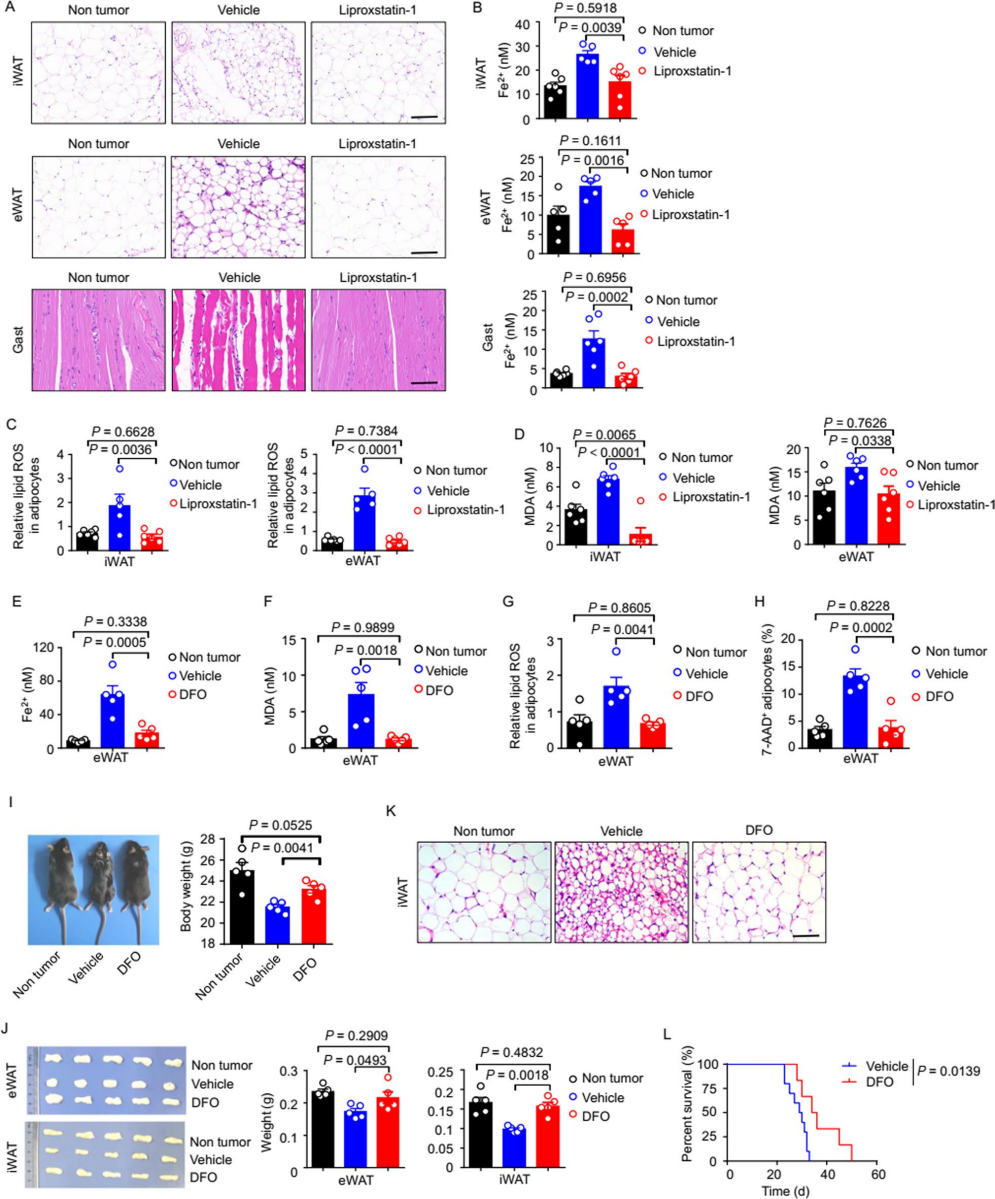
Chemical inhibition of ferroptosis reduces tissue wasting in lung cancer cachexia.** A–D**. Lung cancer cachexia model mice were treated with the ferroptosis inhibitor, liproxtatin-1 (10 mg per kg body weight, delivered by intraperitoneal injection daily between days 1 and 20), and the tissues were harvested on day 21. **A**. Representative H&E staining of the iWAT, eWAT, and Gast. Scale bars, 100 μm. **B**. Fe^2+^ concentration in the iWAT, eWAT, and Gast. n = 5–6 per group. **C**. Flow cytometry analyses of the relative lipid ROS levels in the adipocytes of the eWAT and iWAT. A PE channel was used to detect non-oxidized lipids and a FITC channel was used to detect oxidized lipids. The FITC to PE MFI ratio was calculated as the relative lipid ROS value. n = 5–6 per group. **D**. Chemiluminescence analysis of the MDA concentration in the iWAT, eWAT, and Gast. n = 6 per group. **E–L**. Lung cancer cachexia model mice were treated with the ferroptosis inhibitor, DFO (15 mg per kg body weight, delivered by intraperitoneal injection every 3 days between days 4 and 19), and the tissues were harvested on day 21. (**E, F)** Concentrations of (**E**) Fe^2+^ and (**F)** MDA in the eWAT. n = 5 per group. (**G, H)** Flow cytometry analyses of (**G)** relative lipid ROS and (**H)** the percentage of 7-AAD^+^ adipocytes in the eWAT. n = 5 per group. **I**. Representative images (left) and body weights (right) of the different groups of mice. n = 5 per group. **J**. Representative images (left) and weights (right) of the eWAT and iWAT. n = 5 per group. **K**. Representative H&E staining of the iWAT. Scale bars, 100 μm. **L**. Kaplan–Meier analysis of mouse survival (n = 10 per group), with comparisons performed using the log-rank test. Statistical analyses were performed using the one-way ANOVA **(B–I, J)**. Data are shown as the mean ± SEM

Until this point, we had focused on the nature of the aberrantly high LCN2 level in lung cancer cachexia model mice and LCN2-mediated induction of ferroptosis in wasting tissues. We were also interested in whether disrupting ferroptosis could protect mice directly from developing lung cancer cachexia. To this end, we first treated lung cancer cachectic model mice with a ferroptosis inhibitor: liproxstatin-1 (10 mg per kg body weight, delivered by intraperitoneal injection daily between days 1 and 20). Histology showed that liproxstatin-1 treatment alleviated the pathological morphology of the iWAT, eWAT, and Gast of cachexia model mice (Fig. 7A). Liproxstatin-1 treatment also alleviated the loss in body weight of cachectic mice (Additional file 1: Fig. S7D). As expected, liproxstatin-1 treatment reduced the Fe^2+^ concentration in eWAT, iWAT, and Gast (Fig. 7B). Liproxstatin-1 treatment also led to a significant reduction in the MDA and lipid ROS levels in eWAT and iWAT (Fig. 7C, D), as well as a significant reduction in expression of ferroptosis-related genes (*Ncoa4*, *Slc39a8*, *Slc3a2*, *Slc39a14*, *Pcbp2*, and *Sat1*) in eWAT, iWAT, and Gast (Additional file 1: Fig. S7C).

We also treated lung cancer cachexia model mice with an alternative ferroptosis inhibitor: DFO (15 mg per kg body weight, delivered by intraperitoneal injection) [33] (Additional file 1: Fig. S7A). Beyond the expected reduction in the Fe^2+^ concentration in eWAT (Fig. 7E), DFO treatment induced four phenotypes in lung cancer cachexia model mice: (i) reduced MDA and lipid ROS levels in eWAT (Fig. 7F, G); (ii) alleviation of weight loss and wasting of eWAT and iWAT (Fig. 7I, J); (iii) alleviation of the pathological morphology of iWAT (Fig. 7K); (iv) reduced expression of ferroptosis-related genes (*Ncoa4*, *Slc39a8*, *Slc3a2*, *Slc39a14*, *Pcbp2*, and *Sat1*) (Additional file 1: Fig. S7B). These four phenotypes were very similar to those of mice subjected to myeloid-specific *LCN2* knockout or treatment with anti-LCN2 antibody. DFO treatment prolonged the survival of lung cancer cachexia model mice significantly (Fig. 7L) and reduced the percentage of dead (7AAD^+^) adipocytes in their eWAT significantly (Fig. 7H). The results of experiments on DFO treatment showed that disrupting ferroptosis protected mice from cachexia directly. Thus, the antibody-based disruption of LCN2-induced ferroptosis or chemical inhibition of ferroptosis represent potentially promising strategies for treating the tissue wasting and multiple other deleterious aspects of cachexia.

## Discussion and conclusions

The incidence of cachexia among cancer patients is relatively high, especially those with cancer of the pancreas, gastrointestinal tract, colon, or lung. Cachexia symptoms can appear early, even if the primary tumor is localized. These systemic changes affect many peripheral tissues that are not proximal to the tumor (*e.g.,* the muscles essential for breathing, moving, chewing, and swallowing food) and are often detrimental to the host [34]. Furthermore, weakened muscle and adipose tissue reduce the tolerance of cancer cachexia patients to anti-tumor therapies. For instance, weakening of the heart muscles and diaphragm muscles often leads to premature death from heart failure or respiratory failure [2, 35]. Frustratingly, efficacious treatment for cancer cachexia is lacking, despite > 100 clinical trials of mediators developed to treat this condition [2]. In addition, many reported cachexia mediators target tumor metastasis-related cachexia but very few target cachexia arising from localized tumors or early tumors. This scenario is suboptimal given that the mediators of cachexia may differ between metastatic tumors and localized primary tumors [2, 36].

We established, by experimental means, that the LCN2-induced ferroptosis of tissue parenchymal (*e.g.*, adipose) cells is one of the causes of tissue wasting in mice with non-metastatic lung cancer cachexia. Ferroptosis has been demonstrated to induce organ injury and various degenerative changes in diverse diseases [19]. For example, there is strong evidence that ferroptosis contributes to ischemia–reperfusion injury, including stroke and ischemic disease of the heart, liver, and kidney [37]. DFO (Desferal®) is a drug approved for the treatment of acute iron overdose and chronic iron overload resulting from repeated blood transfusions. It is an iron-chelating agent that binds excess free iron and forms a stable complex that inhibits ferroptosis [38]. We found that the DFO-mediated inhibition of ferroptosis significantly and reduced wasting of adipose tissue in a mouse model of lung cancer cachexia. In addition, DFO treatment lengthened the survival of these mice significantly.

LCN2 is a mediator implicated in several diseases: cachexia, cancer, pneumonia, and kidney disease [6–9]. There are two forms of LCN2 under physiological conditions. It is now clear that the function of the iron-free form of LCN2 is distinct from that of the iron-loaded from [39]. Meier et al*.* [40] reported that iron-loaded LCN2 promoted the growth and progression of renal cell carcinoma, whereas iron-free LCN2 exerted anti-tumoral activity; however, the mechanistic details of cancer-related LCN2 signaling are not known. Iron-free LCN2 has been used as a marker for renal regeneration [41]. Incidentally, the exogenous LCN2 used in our study was the iron-free form. Therefore, further studies assessing the impact of the iron-loaded form of LCN2 on ferroptosis are needed to deepen our understanding of the diverse functions of LCN2.

LCN2-related signaling varies among diseases affecting different organs. Liu et al*. *[42] reported that the stress-responsive transcription factor nuclear protein-1 transactivates LCN2 in pancreatic cancer cells which, in turn, induce ferroptosis resistance . Yao et al*.* [43] reported that an leukemia inhibitory factor receptor (LIFR)/nuclear factor-kappa B (NF-κB)/LCN2 axis controls liver tumorigenesis and vulnerability to ferroptosis. They showed that loss of LIFR activates NF-κB signaling, thereby leading to upregulation of expression of the iron-sequestering cytokine LCN2 which, in turn, depletes iron and renders liver cells insensitive to ferroptosis inducers.

LCN2 has been reported to mediate appetite suppression during pancreatic cancer cachexia [12]. We found that LCN2 overexpression in healthy mice or LCN2 depletion in LLC tumor-bearing mice did not influence the food intake of animals significantly. We established that TI-Neu-derived LCN2 induced the ferroptosis of adipocytes directly, leading to the wasting of adipose tissues. A therapeutic antibody targeting LCN2 prolonged the survival of tumor-bearing mice effectively and prevented wasting of adipose tissue and muscle in these animals. In addition, LCN2 expression was upregulated in lung cancer patients and was associated with cachexia progression. Collectively, these findings indicate that LCN2 may represent a valuable target in the treatment of cachexia caused by lung cancer and other types of cancer.

Wang and colleagues suggested that LCN2 knockdown protected a lipopolysaccharide- induced model of acute respiratory distress syndrome via inhibition of ferroptosis-related inflammation and oxidative stress by inhibiting the mitogen-activated protein kinase/extracellular signal-regulated kinase (MAPK/ERK) pathway [44]. Huang et al*.* [45] found that signal transducer and activator of transcription-3 (STAT3)-mediated lysosomal cell death promoted ferroptosis in PDAC. They found the MEK-ERK pathway promotes STAT3 activation in ferroptosis, and that STAT3 contributes to erastin-induced ferroptosis. Wang and colleagues [46] reported the crosstalk of Lcn2/Janus kinase 2 (JAK2)-STAT3 in neurotoxic microglia and astrocytes. We also found the JAK-STAT pathway to be enriched in the eWAT of cachectic mice (Fig. 2B). Thus, LCN2 may induce ferroptosis by activating the STAT3 pathway.

## Supplementary Information

**Additional file 1.** **Fig. S1:** LCN2 levels are increased in the wasting tissues in murine lung cancer cachexia. **Fig. S2:** Ferroptosis occurs in wasting tissues of mice with PDX-induced lung cancer cachexia. **Fig. S3:** LCN2 promotes tissue ferroptosis and wasting. **Fig. S4:** Lung cancer cachectic mice have an increased number of myeloid cells and exhibit higher LCN2 expression in wasting adipose tissues. **Fig. S5:** Depletion of neutrophils alleviates tissue ferroptosis and wasting in lung cancer cachexia. **Fig. S6:** LCN2 knockout alleviates tissue ferroptosis and wasting in lung cancer cachexia. **Fig. S7:** Chemical inhibition of ferroptosis alleviates tissue wasting in lung cancer cachexia. **Table S1:** Materials. **Table S2:** List of qPCR primers used in this study.

**Additional file 2.** **Table S3:** Human serum proteomics.

**Additional file 3.** **Table S4:** Mice serum proteomics.

**Additional file 4.** **Table S5:** Clinical characteristics of patients in this study.

**Additional file 5.** **Table S6:** Differential expressed genes in eWAT.

**Additional file 6. Table S7:** KEGG gene signatures.

## Data Availability

Microarray data were deposited into the National Center for Biotechnology Information GEO repository (accession number: GSE188479). The main materials used in the study are listed in Additional file 1: Table S1. The primers used in the qPCR are listed in Additional file 1: Table S2. The DEPs between the sera of healthy individuals and those of non-cachectic or cachectic lung cancer patients are listed in Additional file 2: Table S3. The DEPs between the sera of cachectic mice and control mice are listed in Additional file 3: Table S4. The clinical characteristics of all patients included in the present study are shown in Additional file 4: Table S5. The DEGs between the eWAT of cachectic mice and control mice are listed in Additional file 5: Table S6. KEGG gene signature (Additional file 6: Table S7).

## Abbreviations

BMI: Body mass index
CRP: C-reactive protein
DEGs: Differentially expressed genes
DEPs: Differentially expressed proteins
DFO: Deferoxamine
eWAT: Epididymal adipose tissue
FPKM: Fragments per kilobase of exon model per million reads mapped
Gast: Gastrocnemius skeletal muscle
GSEA: Gene set enrichment analysis
H&E: Hematoxylin and eosin
IBMX: Isobutylmethylxanthine
IL-6: Interleukin-6
iWAT: Inguinal white adipose tissue
LCN2: Lipocalin 2
LIFR: Leukemia inhibitory factor receptor
LLC: Lewis lung carcinoma
MDA: Malondialdehyde
MDSCs: Myeloid-derived suppressor cells
MFI: Mean fluorescence intensity
NF-κB: Nuclear factor-kappa B
NK cells: Natural killer cells
PDX: Patient derived xenograft
PFUs: Plaque-forming units
PUFAs: Poly-unsaturated fatty acids
ROS: Reactive oxygen species
TI-Neu: Tissue-infiltrating neutrophils
