# Supplementary material for: LCN2 secreted by tissue-infiltrating neutrophils induces the ferroptosis and wasting of adipose and muscle tissues in lung cancer cachexia
Source: J Hematol Oncol. 2023 Mar 27;16:30. doi: 10.1186/s13045-023-01429-1 (PMC10044814; doi:10.1186/s13045-023-01429-1)
Supplement: Supplementary file 1 — Additional file 1. Fig. S1: LCN2 levels are increased in the wasting tissues in murine lung cancer cachexia. Fig. S2: Ferroptosis occurs in wasting tissues of mice with PDX-induced lung cancer cachexia. Fig. S3: LCN2 promotes tissue ferroptosis and wasting. Fig. S4: Lung cancer cachectic mice have an increased number of myeloid cells and exhibit higher LCN2 expression in wasting adipose tissues. Fig. S5: Depletion of neutrophils alleviates tissue ferroptosis and wasting in lung cancer cachexia. Fig. S6: LCN2 knockout alleviates tissue ferroptosis and wasting in lung cancer cachexia. Fig. S7: Chemical inhibition of ferroptosis alleviates tissue wasting in lung cancer cachexia. Table S1: Materials. Table S2: List of qPCR primers used in this study. [file 13045_2023_1429_MOESM1_ESM.docx]

**Supplementary information includes:**

Supplementary Figs. S1–7

Tables S1–S2

Other supplementary material for this manuscript includes:

Tables S3–S7 (Excel format)


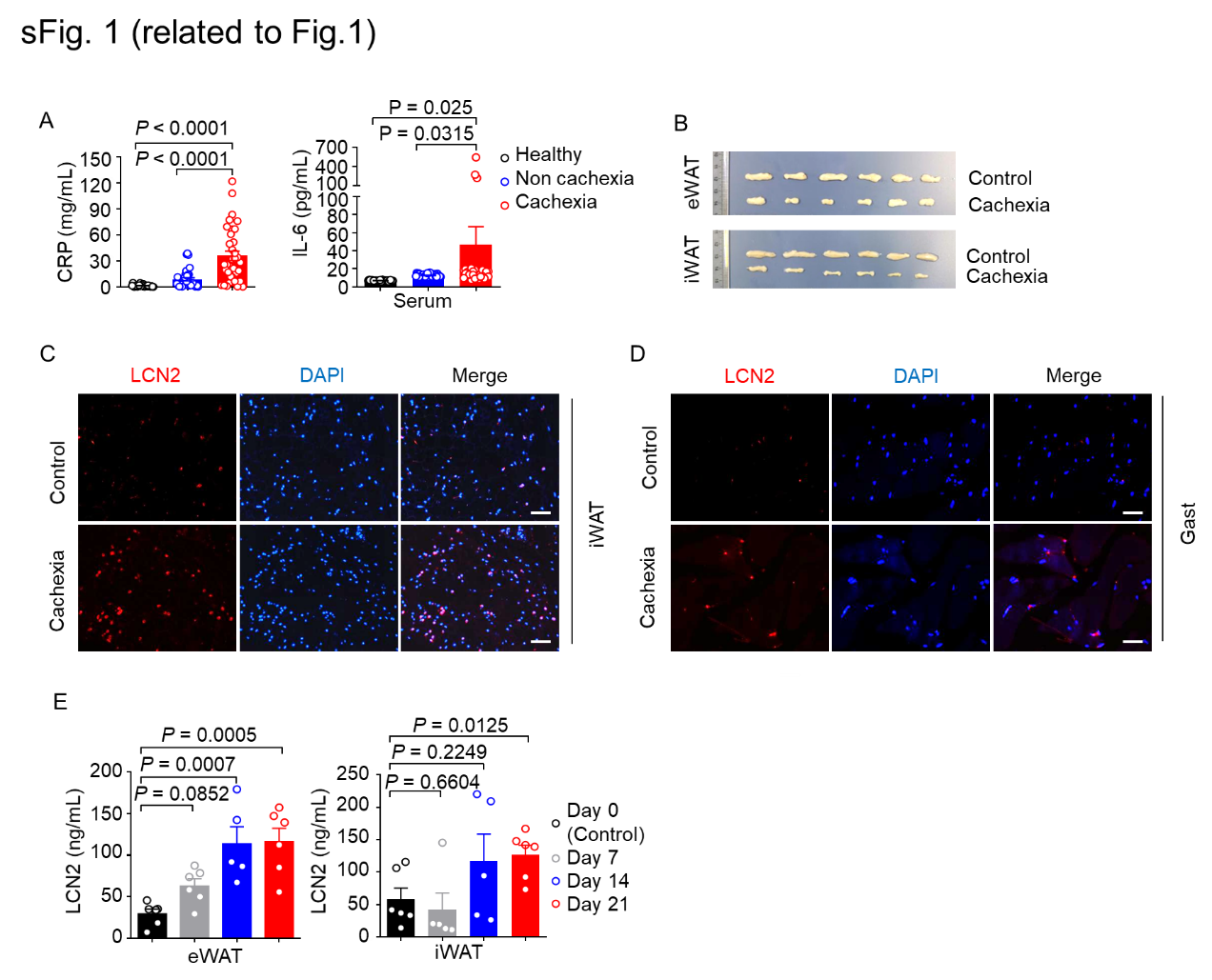


**sFig. 1 LCN2 levels are increased in the wasting tissues in murine lung cancer cachexia.**

**A.** Serum concentrations of IL-6 and CRP in lung cancer patients with (n = 31–35) and without (n = 33–34) cachexia and in healthy controls (n = 25–32). **B–E.** Mice were inoculated with LLC cells to induce cachexia. **B.** Representative images of the eWAT and iWAT in these mouse groups. **C.** LCN2 immunofluorescence staining in the iWAT of control and cachectic mice. Scale bars, 50 μm. **D.** LCN2 immunofluorescence staining in the Gast of control and cachectic mice. Scale bars, 50 μm. **E.** LCN2 concentrations in supernatants prepared from the eWAT and iWAT of mice bearing LLC tumors (up to 21 days). n = 5–6 per group. Data are shown as the mean ± SEM. Statistical analyses were performed using one-way ANOVA **(A, E**).


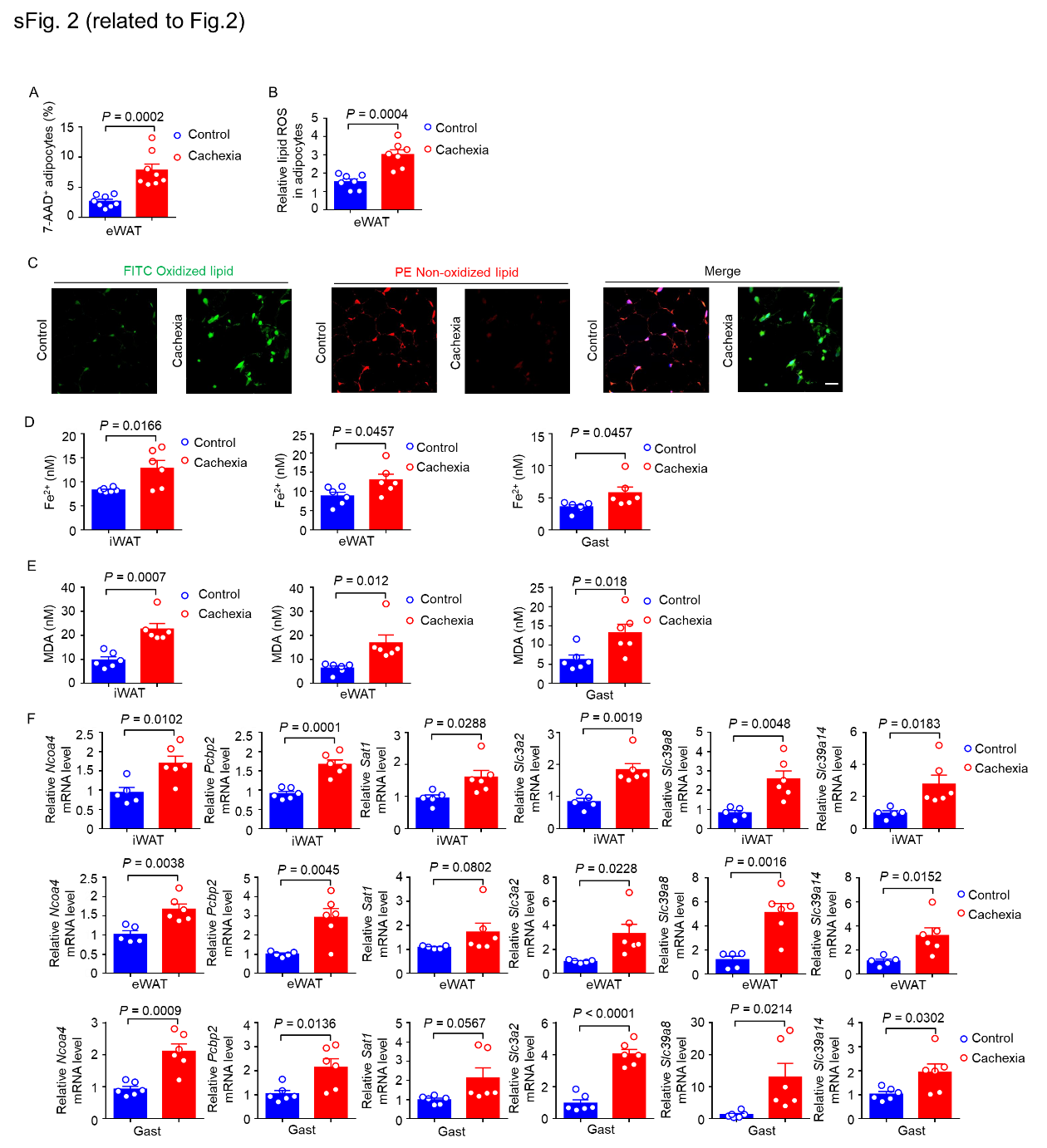


**sFig. 2 Ferroptosis occurs in wasting tissues of mice with PDX-induced lung cancer cachexia.**

**A–C.** Mice were inoculated with LLC cells to induce murine cachexia. **A.** Percentages of 7-AAD^+^ adipocytes purified from the eWAT, assessed using flow cytometry. n = 8 per group. **B.** Relative lipid ROS in adipocytes purified from the eWAT, determined by flow cytometry. n = 7 per group. **C.** Immunofluorescence staining of lipid peroxidation in the iWAT (using the Lipid Peroxidation Assay Kit, Abcam, Cat number ab243377). Scale bars, 50 μm. **D.** Chemiluminescence analysis of Fe^2+^ concentrations in the eWAT, iWAT, and Gast of PDX-induced cachectic model mice and control mice. n = 6 per group. **E.** Chemiluminescence analysis of MDA concentration in the eWAT, iWAT, and Gast of PDX-induced cachectic model mice and control mice. n = 6 per group. **F.** qPCR analysis of mRNA levels of the indicated ferroptosis-related genes in the eWAT, iWAT, and Gast of PDX-induced cachectic model mice and control mice. n = 6 per group. Data are shown as the mean ± SEM and were compared using the unpaired Student’s t-test.


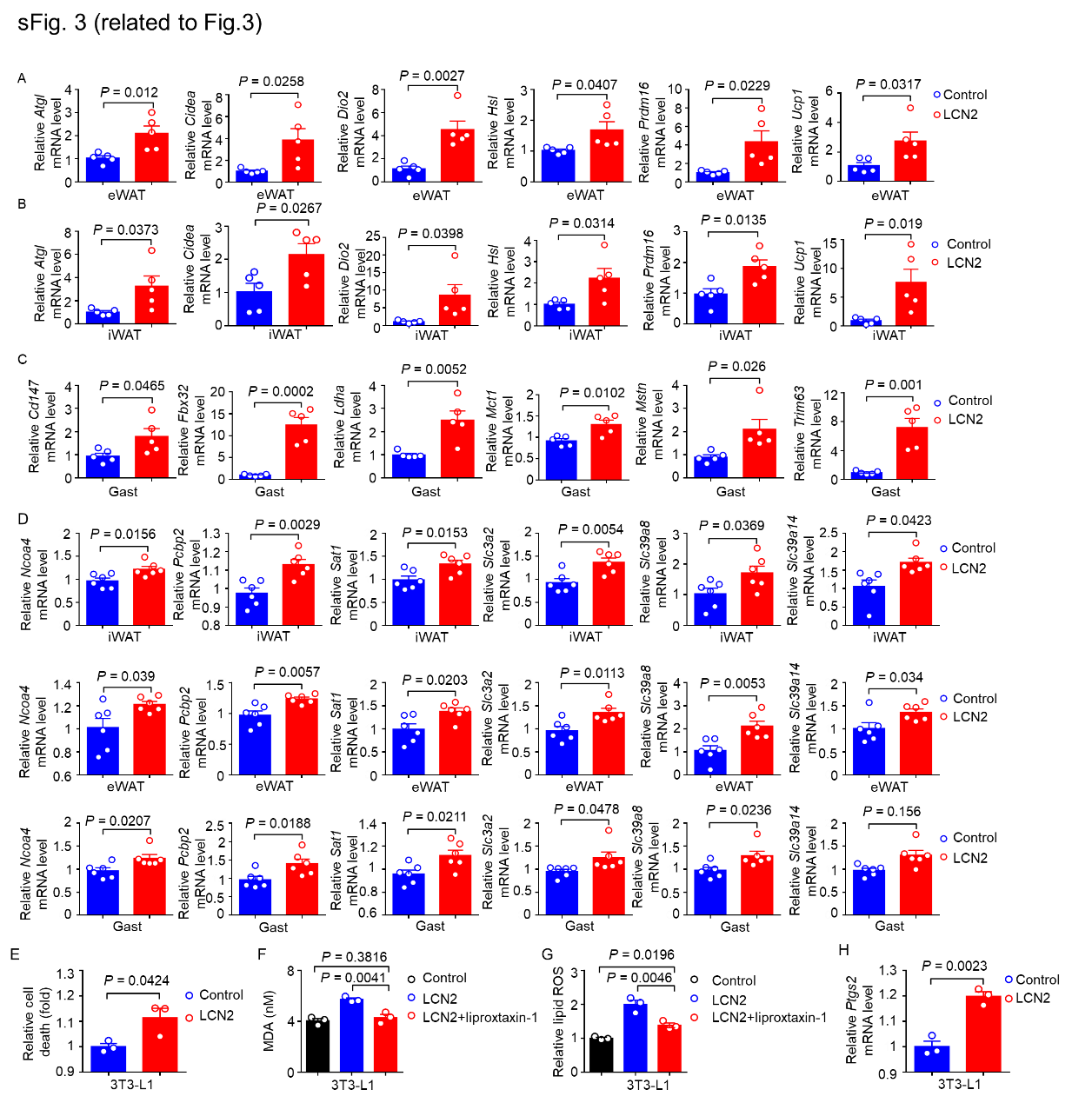


**sFig. 3 LCN2 promotes tissue ferroptosis and wasting.**

Mice were injected intravenously with the control or the LCN2-overexpressing lentivirus. **A–C.** qPCR determination of mRNA levels in the (**A**) eWAT, (**B)** iWAT, and (**C)** Gast. n = 5 per group. **D.** qPCR analysis of mRNA levels of the indicated ferroptosis-related genes in the eWAT, iWAT, and Gast from mice injected with the control or LCN2- overexpressing lentivirus. n = 6 per group. **E.** 3T3-L1 cells were treated with 200 ng/mL recombinant mouse Lcn2 protein for 24 h. Relative cell death was detected by flow cytometry. n = 3 per group. **F–G.** The 3T3-L1 cells were pretreated with 10 μM liproxstatin-1 or vehicle for 24 h. The 3T3-L1 cells were then treated with 200 ng/mL recombinant mouse Lcn2 protein for 24 h. The MDA concentration (**F**) and flow cytometry analysis of relative lipid ROS levels (**G**) in 3T3-L1 cells. n = 3 per group. **H.** 3T3-L1 cells were treated with 200 ng/mL recombinant mouse Lcn2 protein for 24 h. qPCR determination of *Ptgs2* mRNA levels. n = 3 per group. Data are shown as the mean ± SEM and were compared using the unpaired Student’s t-test.

**
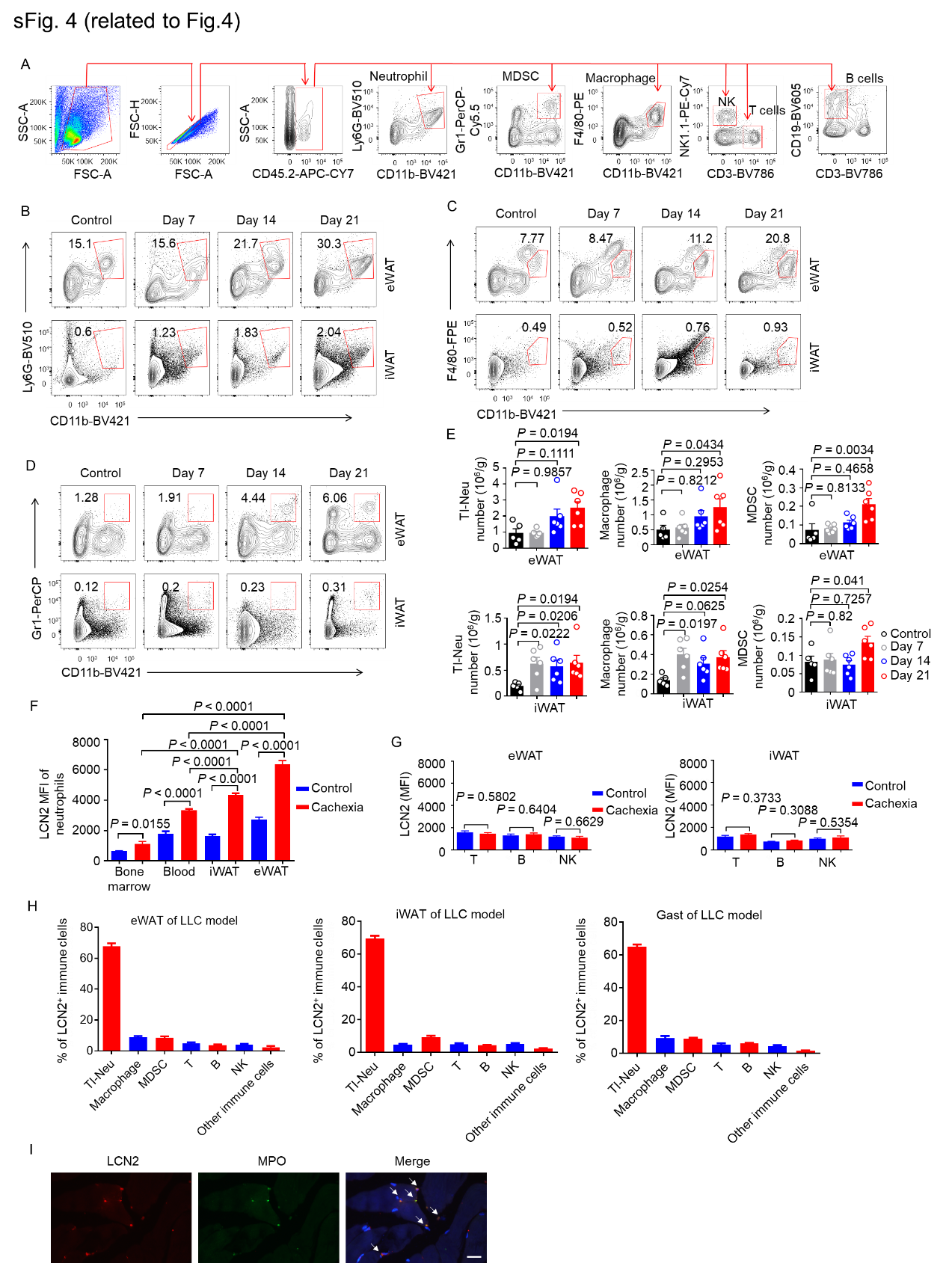
**

**sFig. 4 Lung cancer cachectic mice have an increased number of myeloid cells and exhibit higher LCN2 expression in wasting adipose tissues.**

Mice were inoculated with LLC cells to induce cachexia. **A.** The flow cytometry gating strategy. **B–D.** Representative flow cytometry plots showing (**B**) neutrophils, (**C)** macrophages, and (**D)** MDSCs in the eWAT and iWAT. **E**, Absolute numbers of neutrophils, macrophages, and MDSCs in the eWAT, iWAT, and Gast. n = 5–6 per group. **F.** Statistical analysis of the MFI values for LCN2 in the neutrophils of the iWAT, eWAT, blood, and bone marrow. n = 6 per group. **G.** Statistical analysis of the MFI values for LCN2 in the T cells, B cells, and NK cells of the iWAT and eWAT. n = 6 per group. H. Proportion of TI-Neu, Macrophage, MDSC, T cells , B cells, NK cells, and other immune cells in LCN2+ immune cells (LCN2+ CD45+) in eWAT, iWAT and Gast of LLC induced cachexia model, respectively. n = 6 per group. **I.** Representative immunofluorescence staining of LCN2 and MPO in the iWAT of LLC induced cachectic mice. Scale bar, 20 μm. Data are shown as the mean ± SEM. Statistical analyses were performed using one-way ANOVA (**E, F**) or unpaired Student’s t-test (**G)**.

**
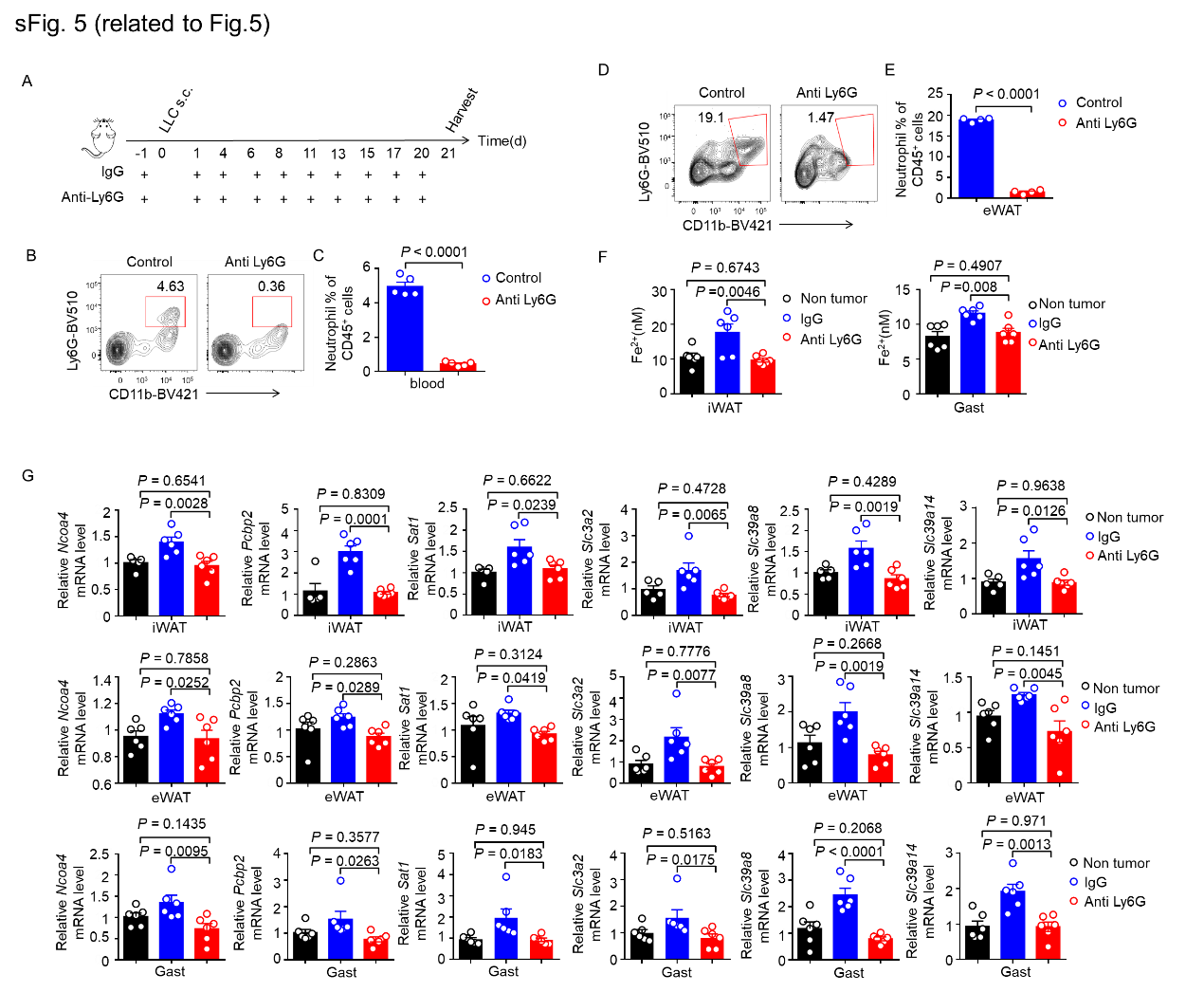
**

**sFig. 5 Depletion of neutrophils alleviates tissue ferroptosis and wasting in lung cancer cachexia.**

Mice inoculated subcutaneously with LLC cells were treated with 100 μg IgG or anti-Ly6G antibody. **A.** Schematic of the antibody therapy regimen. **B.** Representative flow cytometry plots showing neutrophils in the blood of mice 1 day after antibody treatment. **C.** Frequency of neutrophils in the blood of mice 1 day after antibody treatment. n = 5 per group. **D.** Representative flow cytometry plots showing neutrophils in the eWAT on day 21. **E.** Frequency of neutrophils in the eWAT on day 21. n = 4 per group. **F.** Fe^2+^ concentrations in the iWAT and Gast, assessed using chemiluminescence. n = 6 per group. **G.** qPCR of mRNA levels of the indicated ferroptosis-related genes in the eWAT, iWAT, and Gast. n = 6 per group. Data are shown as the mean ± SEM. Statistical analyses were performed using one-way ANOVA (**F, G**) or unpaired Student’s t-test (**C, E)**.

**
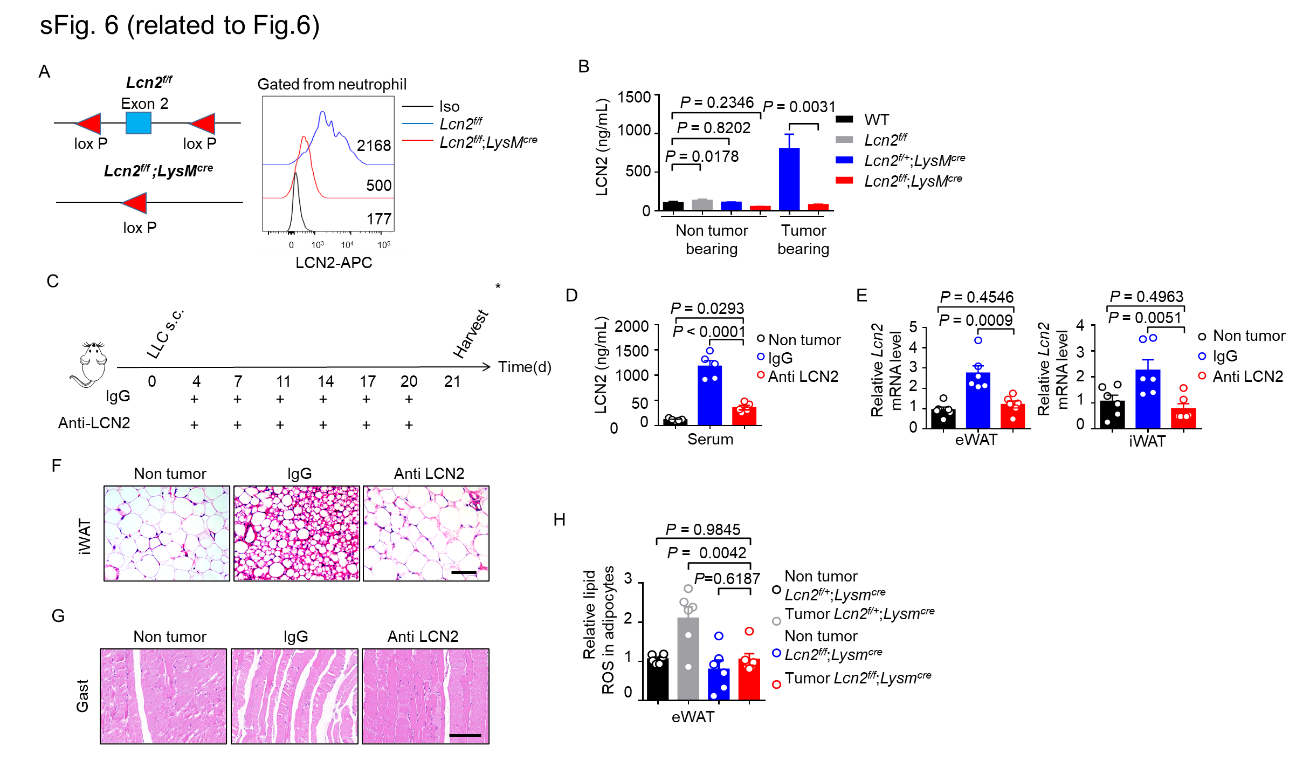
**

**sFig.6 *LCN2* knockout alleviates tissue ferroptosis and wasting in lung cancer cachexia.**

**A.** Left, diagram showing *Lcn2^f/f^;LysM^cre^* model construction. Right, representative flow cytometry plots of LCN2 expression in neutrophils of the *Lcn2^f/f^* and *Lcn2^f/f^;LysM^cre^* mice. **B.** LCN2 concentrations in the sera of *Lcn2^f/+^;LysM^cre^* and *Lcn2^f/f^;LysM^cre^* mice injected with LLC cells. n = 4 for each type of non-tumor-bearing mouse and n = 6 for each type of tumor-bearing mouse. **C–H.** Mice inoculated subcutaneously with LLC cells were administered 50 μg IgG or anti-LCN2 antibody. **C.** Schematic of antibody therapy. **D.** Serum LCN2 concentrations. n = 5 per group. **E.** qPCR determination of *Lcn2* mRNA levels in the eWAT and iWAT. n = 6 per group. Representative H&E staining of the (**F**) iWAT and (**G**) Gast. Scale bars, 100 μm. **H.** Analysis of relative lipid ROS levels in the eWAT of *Lcn2^f/+^;LysM^cre^* and *Lcn2^f/f^;LysM^cre^* lung cancer cachexia model mice and controls. n = 6 per group. Data are shown as the mean ± SEM. Statistical analyses were performed using one-way ANOVA (**B, D, E, H**).

**
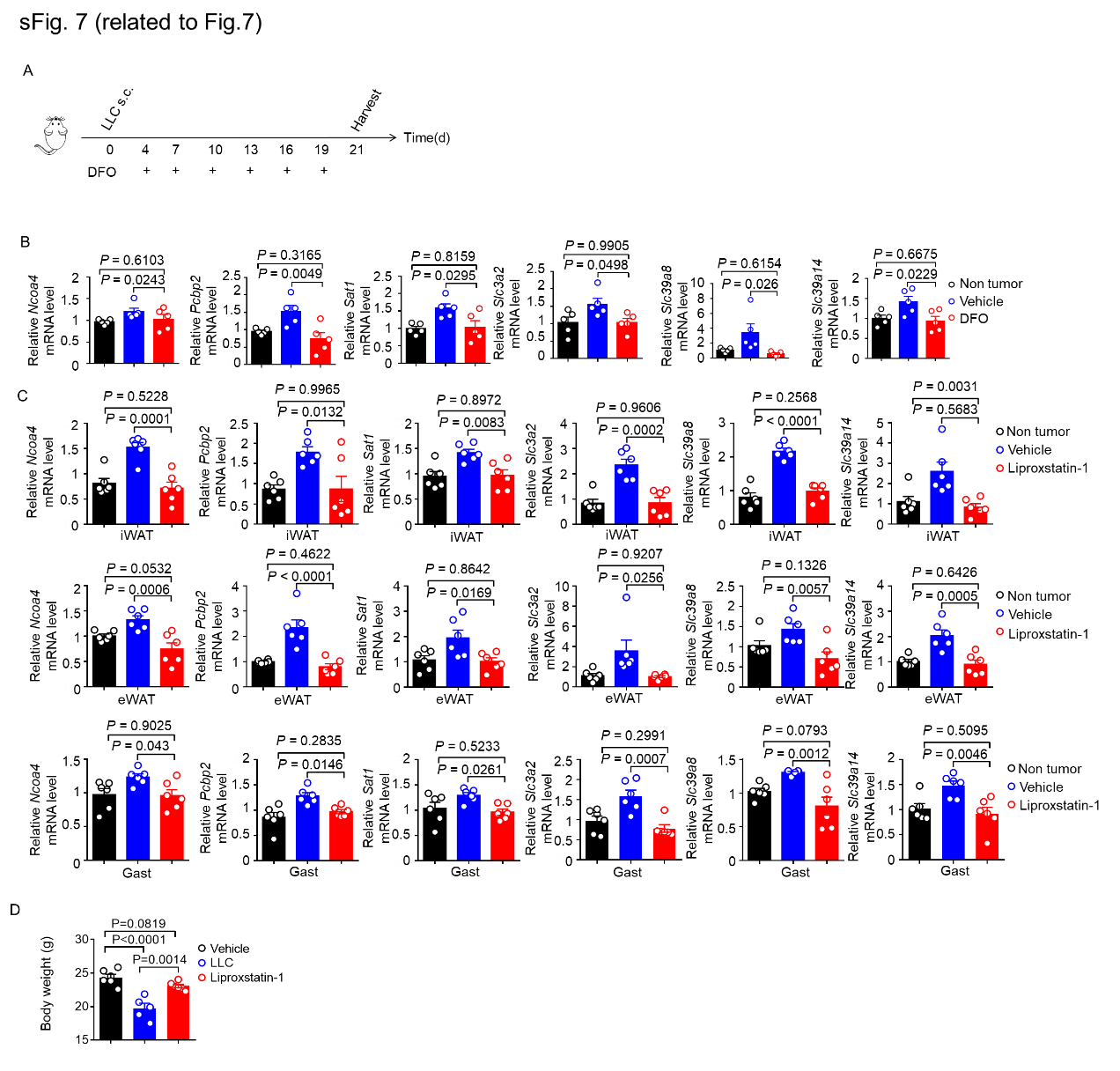
**

**sFig. 7 Chemical inhibition of ferroptosis alleviates tissue wasting in lung cancer cachexia.**

**A–B.** Mice inoculated subcutaneously with LLC cells were given 15 mg DFO per kg body weight. **A.** Schematic of DFO therapy. **B.** qPCR determination of mRNA levels in the eWAT. n = 5 per group. **C-D.** Lung cancer cachexia model mice were treated with the ferroptosis inhibitor, liproxtatin-1 (10 mg per kg body weight). **C.** qPCR analysis of mRNA levels of the indicated ferroptosis-related genes in the eWAT, iWAT, and Gast. n = 6 per group. **D.** Body weights of mice in each group. n = 5-6 per group. Data are shown as the mean ± SEM. Statistical analyses were performed using one-way ANOVA (**B-D**).

**Table S1 Materials**

| Reagent | Source | Cat# |
| --- | --- | --- |
| **Flow cytometry materials** | | |
| PE anti-mouse F4/80 | eBioscience | Cat#12-4801-80;  RRID:AB_465922 |
| PerCP-Cy 5.5 anti-mouse Gr-1 | BioLegend | Cat# 108428;RRID:AB_893558 |
| PE-CY7 anti-mouse NK1.1 | BioLegend | Cat# 108714, RRID:AB_389364 |
| APC-CY7 anti-mouse CD45.2 | BioLegend | Cat# 109824, RRID:AB_830789 |
| BV421 anti-mouseCD11b | BioLegend | Cat# 101236, RRID:AB_11203704 |
| BV510 anti-mouse Ly6G | BD Biosciences | Cat# 740157, RRID:AB_2739910 |
| BV605 anti-mouse Ly6C | BioLegend | Cat# 128036, RRID:AB_2562353 |
| BV786 anti-mouse CD3 | BD Biosciences | Cat# 564379, RRID:AB_2738780 |
| BV605 anti-mouse CD19 | BD Biosciences | Cat# 563148, RRID:AB_2732057 |
| FITC anti-human CD66 | BD Biosciences | Cat# 551479, RRID:AB_394217 |
| PE anti-human CD33 | BD Biosciences | Cat# 555450, RRID:AB_395843 |
| PerCP-Cy 5.5 anti-human CD16 | BioLegend | Cat# 302028, RRID:AB_893262 |
| PE-CY7 anti-human CD19 | BD Biosciences | Cat# 557835, RRID:AB_396893 |
| APC-CY7 anti-human CD14 | BD Biosciences | Cat# 557831, RRID:AB_396889 |
| BV421 anti-human CD56 | BD Biosciences | Cat# 318328, RRID:AB_11218798 |
| BV605 anti-human CD45 | BD Biosciences | Cat# 564047, RRID:AB_2744403 |
| BV786 anti-human CD3 | BD Biosciences | Cat# 563799, RRID:AB_2744384 |
| Collagenase I | Sigma | Cat#C0130 |
| RBC lysis buffer | Biolegend | Cat#420301 |
| Percoll | GE Healthcare | Cat#17-5445-02 |
| PMA | Sigma | Cat#P1585 |
| Intracellular fixation & permeabilization buffer set | eBioscience | Cat#88-8824-00 |
| **Western blot materials** | | |
| Lipocalin-2/NGAL antibody | Novus Biologicals | Cat# NBP2-66935 |
| Anti-GAPDH antibody | Proteintech | Cat# 60004-1-Ig, RRID:AB_2107436 |
| HRP conjugated goat anti-mouse IgG (H+L) | Proteintech | Cat# SA00001-1, RRID:AB_2722565 |
| HRP conjugated goat anti-rabbit IgG (H+L) | Proteintech | Cat# SA00001-2, RRID:AB_2722564 |
| PMSF | Beyotime | Cat#ST506 |
| SDS-PAGE protein loading buffer | Beyotime | Cat#P0015 |
| Blue plus II protein marker | Transgen | Cat#DM111 |
| Supersignal west femto maximum sensitivity substrate | Thermofisher | Cat#34094 |
| **RT-qPCR materials** | | |
| Random primers | Sangon Biotech | Cat#B300816 |
| Highly pure dNTPs | Transgen | Cat#AD101 |
| **ELISA materials** | | |
| Mouse lipocalin-2/NGAL quantikine ELISA kit | R＆D Systems | Cat#MLCN20 |
| Human lipocalin-2/NGAL quantikine ELISA kit | R＆D Systems | Cat#DLCN20 |
| Human IL-6 ELISA kit | Multiscience | Cat#70-EK106/2-96 |
| Human CRP ELISA kit | Multiscience | Cat# 70-EK194-96 |
| **Software** | | |
| Flow jo | FlowJo LLC | https://www.flowjo.com/solutions/flowjo |
| GraphPad Prism 5.0 | GraphPad Software | https://www.graphpad.com/ |
| MEV v.4.8.1 | Multiple Experiment Viewer | http://mev.tm4.org/ |
| R software | Ross Ihaka and Robert Gentleman | https://www.r-project.org/ |

**Table S2. List of qPCR primers used in this study.**

| **Gene name** | **Sequence (5'➡3’)** |
| --- | --- |
| *Atgl* F | CAGCACATTTATCCCGGTGTAC |
| *Atgl* R | AAATGCCGCCATCCACATAG |
| *Cd147* F | TTCTTATAGAGCCGCAGTGGG |
| *Cd147* R | ACAGTGGTGCCTTGAGGAAA |
| *Cidea* F | CCGATGCACAAGCTTCAAGG |
| *Cidea* R | GTATGTGCCCGCATAGACCA |
| *Dio2* F | GTCCGCAAATGACCCCTTT |
| *Dio2* R | CCCACCCACTCTCTGACTTTC |
| *Fbx32* F | TCAGAGAGGCAGATTCGCAA |
| *Fbx32* R | GGGTGACCCCATACTGCTCT |
| *Hsl* F | GCTGGAGGAGTGTTTTTTTGC |
| *Hsl* R | AGTTGAACCAAGCAGGTCACA |
| *Ldha* F | AAGCACGTTGCTATGCCTTG |
| *Ldha* R | GAACCCCAAAAGGGGATGGT |
| *Lipocalin2* F | AATGTCACCTCCATCCTGGTC |
| *Lipocalin2* R | GCCACTTGCACATTGTAGCTC |
| *Mct1* F | AGTGCAACGACCAGTGAAGT |
| *Mct1* R | GCGATCATTACTGGACGGCT |
| *Mstn* F | AGAAGATGGGCTGAATCCCTTT |
| *Mstn* R | ATCGCAGTCAAGCCCAAAGT |
| *Ncoa4* F | CTAAGGTCCGCTCGGATCAC |
| *Ncoa4* R | GCCCGAAGTACTCCACCAAT |
| *Pcbp2* F | AGTATGCCATTCCACAGCCA |
| *Pcbp2* R | ACCTGCCCAATAGCCTTTCA |
| *Prdm16* F | GCACGGTGAAGCCATTCATATG |
| *Prdm16* R | TCGGCGTGCATCCGCTTGTG |
| *Ptgs2* F | CCCATGGGTGTGAAGGGAAAT |
| *Ptgs2* R | TCCATCCTTGAAAAGGCGCA |
| *Sat1* F | GGACCCCTGAAGGACATAGC |
| *Sat1* R | ATACTGCTGCAGCGACACTT |
| *Slc3a2* F | TAAGCCGCGTGTTGATCCAT |
| *Slc3a2* R | GCTCGTTCAGCTCCACATCT |
| *Slc39a8* F | TCCACTTCGACACTGTCAGC |
| *Slc39a8* R | AAGCGTGATCATCCAGGCAA |
| *Slc39a14* F | CACTCAGTAGCTGTGTCGCC |
| *Slc39a14* R | CCACGGATACCAGGATGGTG |
| *Trim63* F | TCCTGATGGAAACGCTATGGAG |
| *Trim63* R | ATTCGCAGCCTGGAAGATGT |
| *Ucp1* F | AAGCTGTGCGATGTCCATGT |
| *Ucp1* R | AAGCCACAAACCCTTTGAAAA |
| *actin* F | CCACTGTCGAGTCGCGTCC |
| *actin* R | ATTCCCACCATCACACCCTGG |
