# Supplementary material for: LCN2 secreted by tissue-infiltrating neutrophils induces the ferroptosis and wasting of adipose and muscle tissues in lung cancer cachexia
Source: J Hematol Oncol. 2023 Mar 27;16:30. doi: 10.1186/s13045-023-01429-1 (PMC10044814; doi:10.1186/s13045-023-01429-1)
Supplement: Supplementary file 2 — Additional file 2. Table S3: Human serum proteomics. [file 13045_2023_1429_MOESM2_ESM.pdf]

Table S3 human serum proteomics (cachexia tumor patients VS non cachexia tumor patien

proteinID	AveExp.CA	AveExp.NC	P.Value	adj.P.Val	foldchange	entrezID	uniprotID	Cachexia1	Cachexia2	Cachexia3	non cachexia1	non cachexia2	non cachexia3
TNF RI	15.26443842	13.47364913	3.16E-05	0.0069472	3.460041378	7132	P19438	51683.8896	33454.153	37658.412	9576.438761	11005.99018	13962.67255
MMP-8	13.98572821	11.34876759	2.86E-05	0.0069472	6.220198526	4317	P22894	12674.4471	14095.58	21758.251	2990.2646	2743.709232	2159.763318
IL-18	15.03502206	11.41850492	0.000330424	0.0363467	12.26535542	3606	Q14116	50455.0063	15704.466	91400.22	3284.704761	1770.584485	3522.279166
Pentraxin 3	14.29749089	12.55276248	0.000279255	0.0363467	3.351317593	5806	P26022	13675.7639	21042.08	21764.035	5750.018149	7894.982384	4775.647556
Lipocalin-2	15.12720454	9.887061791	0.000431874	0.0671349	1.536469392	3934	P80188	64134.3917	61388.23	71543.11	39891.29675	35080.88296	32752.29631
LAP(TGFb1)	15.14955079	12.31533048	0.001149872	0.084324	7.131572931	7040	P01137	24050.8214	35513.402	42246.126	14417.50232	2219.828552	4133.437774
B7-H3	14.52852925	13.38832767	0.000998612	0.084324	2.20411818	80381	Q5ZPR3	21609.9965	24562.972	22251.249	7340.807063	11463.33454	14644.7914
BLC	13.89234015	10.3104877	0.001606654	0.1009897	11.9741592	10563	O43927	38878.2307	9962.2958	3491.2904	1284.558942	1311.843314	1212.37016
Flt-3L	10.15612848	11.6336628	0.003566764	0.1234871	0.359102023	2322	P36888	1139.70461	728.9301	1938.4534	4619.455422	2654.336113	2613.809764
TRAIL R3	13.85005404	13.06655887	0.003164851	0.1234871	1.721295955	8794	O14798	18048.5871	11472.655	13828.643	7971.07083	9145.896841	8657.318399
uPAR	13.72706431	12.15269213	0.003839063	0.1234871	2.978058676	5329	Q03405	19982.4543	6501.1672	18124.508	5041.045484	3787.734689	4940.668674
GDF-15	16.89084564	15.87914139	0.002996798	0.1234871	2.016291528	9518	Q99988	129361.845	121605.55	137109.82	72643.96258	50136.5258	60106.01867
GRO	12.36534626	11.1697951	0.002805019	0.1234871	2.290323137	2919	P09341	4168.31168	4199.4228	7093.1741	2789.267142	1591.573433	2750.597228
MIF	13.13562342	11.69503351	0.003024111	0.1234871	2.714318294	4283	Q07325	6677.85723	6299.5218	9483.1914	4052.781899	2541.604054	3535.132665
MIP-3b	10.34170119	12.59780463	0.003929134	0.1234871	0.209336612	6363	Q99731	953.20515	807.45709	3899.1626	8007.360702	7325.603972	4058.802197
IL-33	8.761153382	11.48161315	0.005089634	0.1492959	0.151726	90865	O95760	235.487664	391.2783	623.06777	4299.532827	6062.7561	895.7386285
HGF	12.46029132	10.82107277	0.006381033	0.1651561	3.114970595	3082	P14210	2686.88882	4236.5783	12189.073	1968.257209	1566.572995	1917.104443
TSLP	8.215403435	11.792055	0.006261377	0.1651561	0.083814777	85480	Q969D9	141.873467	101.5458	1675.1967	4076.108534	8545.338359	1278.836361
PDGF-AA	14.36274462	12.55488846	0.007243804	0.1766072	3.501216231	5154	P04085	11372.9699	15363.817	36565.897	6006.932326	3372.232781	10749.35268
VEGF R3	11.40403815	10.16314254	0.009687019	0.1766072	2.363452066	2324	P35916	3491.93341	3378.975	2455.3885	963.929975	1022.76016	1524.942758
I-TAC	9.267522896	8.457966595	0.010034501	0.1766072	1.752672328	6373	O14625	516.742385	616.25125	915.84899	403.4215801	327.2849839	326.5062578
Neprilysin	9.704433269	11.87233647	0.009297776	0.1766072	0.222533863	4311	P08473	1236.67402	435.59861	894.38202	4406.749713	9710.122015	1230.12416
Syndecan-1	14.47821993	13.4217909	0.007735655	0.1766072	2.079777269	6382	P18827	24517.7419	18352.147	23785.978	7258.729207	10774.95738	16892.07383
LRP-6	10.60449617	12.786993	0.008433324	0.1766072	0.220294162	4040	O75581	833.803231	970.25875	4276.9792	8472.781733	11143.48845	3737.246439
BMPR-IB	2.113807939	9.244433721	0.008827019	0.1766072	0.007136212	658	O00238	349.976774	0	0	587.9986206	915.5486469	412.3193428
CD48	1.899169963	10.10315011	0.009609777	0.1766072	0.003391219	962	P09326	0	192.56575	0	1687.32225	630.1220671	1247.777288
Follistatin	12.88956997	12.2587075	0.010940397	0.1782879	1.548490435	10468	P19883	8161.71704	8678.1773	8257.1638	4678.770709	4479.718103	5611.337727
TIMP-4	13.9480065	12.29788845	0.010929797	0.1782879	3.138593205	7079	Q99727	8879.34463	17305.617	18518.32	3318.016783	3437.192155	11187.20267
IP-10	11.82388221	10.78118182	0.013473398	0.2117248	2.060080029	3627	P02778	3799.32532	3276.5963	4697.3074	2057.096418	1087.85674	2430.872999
LOX-1	12.68297588	10.73321094	0.01454447	0.2206747	3.863115826	4973	P78380	2319.22609	4562.2598	15293.137	2061.549989	1642.801616	1453.793806
HVEM	12.95357896	12.13593868	0.017059538	0.2274605	1.762520795	8764	Q92956	11704.7927	5831.191	7913.9329	5653.476678	4052.058457	3977.042494
AgRP	11.44612125	10.59492774	0.016981913	0.2274605	1.803992711	181	O00253	4755.23874	1894.4504	2704.9048	1609.007283	1668.209831	1375.684367
MMP-3	13.32473957	11.5334218	0.016755281	0.2274605	3.461309072	4314	P08254	15630.9245	10525.03	13878.785	5154.848168	4445.026356	1135.175936
DLL1	9.1384091	7.885707911	0.015812916	0.2274605	2.382871557	28514	O00548	771.115034	369.52381	1061.2699	251.3185926	195.594874	265.6750661
IL-1ra	13.18148762	11.91198449	0.018023516	0.2332455	2.410785219	3557	P18510	4400.35726	9272.9468	19822.254	4108.56303	3542.626376	3928.618149
RAGE	13.98433187	14.77888557	0.0190772	0.2398277	0.576521491	177	Q15109	21121.0136	17258.132	13724.88	28429.57277	31566.41873	24752.63024
IL-31	8.805925201	11.090298	0.019627444	0.239891	0.205274624	386653	Q6EBC2	279.882901	354.37677	1236.2529	3750.079698	3397.410864	812.0268762
IL-17F	9.029556223	11.38597888	0.020274957	0.2411076	0.195274754	112744	Q96PD4	252.950011	320.59276	1202.589	3915.746416	6896.6001	708.4798249
4-1BB	9.881485444	12.18499383	0.031963485	0.2546096	0.202569884	3604	Q07011	319.199753	712.56789	1785.8356	3139.285461	17067.7957	1882.535955
GM-CSF	11.27616108	10.03187524	0.033616412	0.2546096	2.369012552	1437	P04141	1024.99508	2014.5675	4362.3129	1033.169286	862.2952477	1284.094577
IFNg	11.69554221	10.51322181	0.034816281	0.2546096	2.26941492	3458	P01579	1516.7875	2466.8408	6276.402	1423.094537	1298.13318	1686.30212
IL-1b	9.403960721	8.158302259	0.031234376	0.2546096	2.371267568	3553	P01584	306.606799	701.24757	1312.4485	373.1659402	171.2564062	360.7774115
IL-2	11.19604793	10.61202718	0.037220251	0.2546096	1.499021159	3558	P60568	1664.96043	2018.7895	3318.346	1515.33103	1527.116465	1653.493288
IL-5	11.21230848	10.51100741	0.036271824	0.2546096	1.625970486	3567	P05113	1552.09003	1922.8334	3308.3166	1471.68448	1332.28825	1581.52939
IL-10	11.19145214	10.68187995	0.037887243	0.2546096	1.423627983	3586	P22301	1647.85885	2313.6948	2950.8553	1694.61948	1570.531888	1662.593548
IL-17	10.09190846	9.641736986	0.031976345	0.2546096	1.366202629	3605	Q16552	1189.53666	1085.0722	1187.4656	765.7437435	789.535328	839.9779429
MIG	8.934310314	7.917514996	0.033226142	0.2546096	2.023419309	4283	Q07325	606.73943	633.18267	653.71687	261.5175846	229.5244742	232.5355902
PDGF-BB	16.72714625	14.68757748	0.038191442	0.2546096	4.111226273	5155	P01127	40366.312	79911.517	221408.92	32465.55737	8469.495192	66811.71386
EGF	15.03953989	12.62882274	0.032513	0.2546096	5.317385811	1950	P01133	8123.10991	25257.825	102416.62	13136.46479	2025.6609	9541.687773
NT-3	10.35426803	11.71626436	0.024126761	0.2546096	0.389043576	4908	P20783	771.722743	2712.0788	875.91855	3719.466717	2325.228653	4400.403733
Axl	13.94076135	13.33804539	0.029338172	0.2546096	1.518572683	558	P30530	21965.9345	14264.602	12471.399	8388.067715	10899.14912	12141.52758
Angiostatin	12.19751265	11.48238765	0.02407511	0.2546096	1.641625442	5340	P00747	3927.62421	5739.7543	3335.7259	2542.78138	3423.031289	2688.130061
DAN	10.22779978	9.260834904	0.024364601	0.2546096	1.954723945	4681	P41271	1651.4977	615.15148	1545.408	733.8778321	472.3703306	662.668327
IL-17B	11.81454651	10.96003455	0.036930921	0.2546096	1.80814698	27190	Q9UHF5	2951.62614	2357.514	5535.5077	2330.903951	1297.801603	2609.020913
SDF-1b	8.220550518	9.298134037	0.022140577	0.2546096	0.4738218	6387	P48061	266.916257	441.26737	457.57678	658.0181455	728.3363935	518.0706686
TREM-1	9.891192402	9.156206541	0.036125924	0.2546096	1.664381158	54210	Q9NP99	1653.86089	678.53661	756.07856	614.6967784	563.2251789	533.6371112
NSE	17.02907942	16.41488633	0.024340588	0.2546096	1.530701624	2026	P09104	122614.868	114341.44	138684.74	98671.51367	68897.78286	98108.65272
IL-24	7.198637649	8.31173791	0.030300578	0.2546096	0.46229951	11009	Q13007	271.501998	209.00654	87.907774	385.3022	275.5570079	299.28624
Kallikrein 14	11.56414041	12.84014908	0.025096426	0.2546096	0.412936351	43847	Q9P0G3	1932.52673	2630.8208	4643.318	10464.50375	11548.79999	3260.936183
PD-1	8.860830458	11.32182892	0.032893901	0.2546096	0.181620825	5133	Q15116	235.002623	283.74509	840.87826	4841.724546	6597.808505	523.8981486
CA9	6.607650847	7.170499869	0.037628173	0.2546096	0.67696398	768	Q16790	95.7122759	90.585316	105.40903	174.8492742	99.44703743	168.2500951
P-Cadherin	13.98612849	12.62314883	0.028683363	0.2546096	2.572158686	1001	P22223	41897.5397	10706.37	11058.377	9291.373695	4223.769638	6395.01711
TF	7.339865694	8.339653139	0.036620318	0.2546096	0.500073671	2152	P13726	158.077988	184.22407	202.77839	589.1329318	299.5278311	190.7002578
Aggrecan	12.1059895	12.81344141	0.035379093	0.2546096	0.612400807	176	P16112	6517.73297	3177.0602	5227.1081	8254.024405	6950.935756	6498.299085
IL-1 R3	12.79210292	13.44845709	0.035625541	0.2546096	0.634479666	3556	Q9NPH3	5615.3431	6065.8451	9532.4589	13175.5716	13146.395	8062.522469
Periostin	12.91082949	13.75578975	0.03064069	0.2546096	0.556726143	10631	Q15063	7006.47533	5366.2685	10897.219	12971.59517	20256.41633	10070.75297
ADAM8	4.216889819	9.939964446	0.035413881	0.2546096	0.018931406	101	P78325	626.674803	189.5051	0	1025.666683	962.4698652	957.108644
IL-16	14.12519718	11.52821995	0.039921231	0.2621693	6.050176467	3603	Q14005	10698.9893	4481.0244	163881.06	4185.366489	2360.548274	2605.069155
Eotaxin-2	12.74532544	11.53132119	0.048268767	0.2678232	2.319806145	6369	O00175	4760.83401	5158.7307	13430.77	1466.258804	3198.189334	5523.259098
ICAM-1	17.56134019	16.99880877	0.045552136	0.2678232	1.476858311	3383	P05362	144042.164	262117.28	222040.64	146734.6351	133411.6268	114740.7475
TNF RII	15.41030869	13.07814846	0.047943204	0.2678232	5.035587934	7133	P20333	47406.6637	40451.66	37083.002	1024.247064	29296.63137	21530.9756
GDNF	10.81897178	10.1561845	0.049760682	0.2678232	1.583138295	2668	P39905	1541.34537	1577.9952	3027.6315	1039.82078	1227.931187	1160.568977
MCP-3	9.588673774	8.422957586	0.047005898	0.2678232	2.243445579	6354	P80098	390.55574	686.14473	1420.2106	444.090175	181.6068804	496.4203081
ANG-1	14.47660506	12.73870455	0.044649041	0.2678232	3.335494156	284	Q15389	9887.21994	10129.6	51514.732	5653.46994	4288.398759	13163.61627
PDGF-AB	11.85446752	9.764927993	0.049042516	0.2678232	4.256122054	767790	Q08CD2	1025.25196	2353.0212	10967.736	1006.165552	296.7758643	2194.929216
Shh-N	9.164688605	9.645675056	0.049912501	0.2678232	0.716487554	6469	Q15465	513.185683	684.85084	520.17399	750.0208743	878.4715343	777.1060029
TPO	10.71354531	11.19376275	0.042698325	0.2678232	0.716869569	7066	P40225	1436.57165	1652.8705	1963.5624	2067.822811	2487.525873	2495.37372
FSH	11.95727089	14.33077797	0.041764383	0.2678232	0.192975943	2488	P01225	15198.0003	1612.9444	12665.562	15375.60242	21423.76536	26557.62784
IL-21	10.73763574	10.2505506	0.04506768	0.2678232	1.401610168	59067	Q9HBE4	1477.4709	1399.9027	1832.8048	1196.252649	1257.53078	1198.830661
Clusterin	11.55735107	9.97160677	0.046369711	0.2678232	3.001626139	1191	P10909	1028.6986	1766.0528	8784.3167	866.0090387	813.8768815	1431.658253
G-CSF R	10.13234175	9.415907396	0.048603826	0.2678232	1.643116019	1441	Q99062	1158.43906	1708.2007	1289.5162	642.9305286	696.5718046	708.5512243
CEACAM-5	2.896658623	9.229221379	0.042842185	0.2678232	0.012408196	1048	P06731	3074.56232	0	0	428.1152847	980.1465569	512.4634647
Pref-1	3.978826172	9.133892269	0.048447233	0.2678232	0.028065351	8788	P80370	733.416461	83.147593	0	516.4784806	950.3821275	359.1466403
OPN	14.37722016	12.74952762	0.051366388	0.2723037	3.090183553	6696	P10451	80499.6975	10154.968	16005.273	3210.64275	11773.21774	8634.976328
IL-1a	12.57667633	11.16490517	0.05230226	0.2739642	2.660636018	3552	P01583	2695.57459	3880.211	13899.327	2299.28093	1239.203659	4241.679066
IL-6	11.55207872	10.69679472	0.053522871	0.2770596	1.809114854	3569	P05231	1839.76295	1954.9467	5102.309	1555.481032	1510.760516	1942.306801
IL-7	11.43422689	10.79711303	0.054159329	0.2770942	1.555214811	3574	P13232	1790.53484	2251.3872	3890.1554	1810.728947	1615.270219	1922.789138
MCSF	9.207750677	8.28357037	0.056010818	0.2800541	1.897605777	1435	P09603	527.827879	295.73686	916.54089	215.2184825	341.6709622	407.3563737
MMP-9	14.32259657	13.15743719	0.055730239	0.2800541	2.24257988	4318	P14780	7817.7658	29596.347	37547.921	9742.767027	12801.00234	6113.275284
ErbB3	14.5242933	13.41525285	0.058447839	0.28057	2.157021338	2065	P21860	52461.8023	9266.5052	28448.174	12492.80331	8213.515698	12701.9296
IL-6R	16.8252121	16.44181717	0.058788751	0.28057	1.30440776	3570	P08887	94247.3875	126463.3	110924.33	90955.55066	96131.12761	80673.80459
IL-11	10.51203841	9.976940216	0.060982503	0.28057	1.449040778	3589	P20809	1524.97182	1186.6578	1982.2383	934.5425533	1011.182489	1079.817689
TNFb	11.69046712	11.17913717	0.061603645	0.28057	1.42536356	4050	Q06643	2330.70029	2912.2053	4601.0918	2331.352704	2171.372482	2459.584736
TRAIL R4	9.847399406	10.16099501	0.05767686	0.28057	0.804633882	8793	Q9UBN6	868.65943	1019.2621	898.27625	1162.67739	1024.238653	1256.868629
Procalcitonin	11.24976459	10.52785338	0.059832246	0.28057	1.649365585	796	P06881	3786.72595	2372.8813	2533.1902	1125.29473	2028.988905	1406.515735
BMP-9	10.32583363	11.32492583	0.058453756	0.28057	0.50031472	2658	Q9UK05	1939.80235	1805.3697	572.99218	2458.388183	1731.893362	3960.213006
ROBO3	11.29369346	13.07232836	0.061852932	0.28057	0.291459049	64221	Q96MS0	2016.29692	1043.0187	6967.9507	11711.27705	21091.31565	2585.56412
ESAM	13.46926045	12.54814186	0.061295686	0.28057	1.893582901	90952	Q96AP7	6640.75269	11306.032	13986.916	9328.476193	6971.375245	3301.655384
IL-8	12.38384157	11.11399928	0.063860699	0.2867215	2.411352037	3576	P10145	3277.39459	2594.7823	18392.345	1953.484511	2041.60727	2726.246301
BDNF	16.68937727	14.75653222	0.066416492	0.2893392	3.818073954	627	P23560	25934.4764	60060.908	290818.58	23017.0973	14881.02493	61906.75311
OPG	12.39715924	11.61983519	0.06574544	0.2893392	1.713948846	4982	O00300	7400.10987	4093.3229	5258.9494	5586.263758	2972.529703	1875.226897

Cadherin-11	10.06219782	10.68144148	0.066308335	0.2893392	0.651012134	1009	P55287	1254.83506	1241.1923	1083.0807	1146.997885	2126.119889	1812.718122
MICA	8.498539159	7.829901394	0.072912419	0.2944605	1.589571339	100507436	Q29983	263.880959	264.54317	462.45509	259.4006811	173.9508606	257.551671
VCAM-1	16.73192506	16.35039785	0.07345742	0.2944605	1.302720161	7412	P19320	121418.455	98746.422	124839.93	99109.73497	83940.35016	70109.64121
IL-13	10.67956981	10.18477246	0.071485573	0.2944605	1.409122807	3596	P35225	1169.992	1371.4053	2269.6217	1077.056977	1221.164009	1195.726263
TNFa	11.53445067	10.98518098	0.073600974	0.2944605	1.463344748	4049	P01374	1968.51337	2586.5941	4114.4045	2035.834217	1884.542055	2167.777716
TARC	10.52980356	9.485515574	0.074284353	0.2944605	2.06234827	6361	Q92583	726.438291	1544.6331	3860.1268	757.6055971	578.548483	836.853947
Fcg RIIBC	10.47036691	9.024341886	0.072745671	0.2944605	2.724563324	2213	P31994	1188.43655	2900.9019	572.82114	369.0198551	984.8016589	386.0599384
Ck beta 8-1	9.744517478	10.52232401	0.072994337	0.2944605	0.583252895	6368	P55773	1225.92354	1099.0653	728.62719	1389.035132	1745.382284	1309.510059
Cadherin-13	4.316733197	9.005573	0.072519167	0.2944605	0.038772033	1012	P55290	423.12972	370.84847	0	477.7645911	592.689073	476.7071313
Galectin-9	14.36317622	13.57714198	0.068536602	0.2944605	1.724328013	3965	O00182	26912.9875	15077.898	17355.208	21509.08507	9484.584876	8945.944345
SP-D	5.510696927	11.51223414	0.073754547	0.2944605	0.01560836	66421	P35247	1238.88256	3483.6546	0	1738.479889	3234.184694	4427.584059
PECAM-1	13.81056993	12.75819527	0.076723153	0.3014124	2.073940732	5175	P16284	7857.67401	18534.759	14314.044	8425.53366	3739.264006	10548.87175
RANTES	17.68016548	16.86550414	0.079386808	0.3091168	1.75888522	6352	P13501	168615.905	213639.08	232883.42	173957.1808	53753.8261	182055.2507
IFNab R2	11.57178194	11.28927407	0.080815067	0.3092055	1.216307378	3455	P48551	2718.29401	2834.1239	3364.7374	2498.696303	2405.728122	2604.650103
HAI-2	11.50266829	10.02744988	0.080175875	0.3092055	2.780257321	6691	P20155	8596.36894	965.11383	5326.3099	1875.818754	688.6650535	877.2719785
CA125	16.59195065	14.20363028	0.082546745	0.3129513	5.235474762	94025	Q8WXI7	70034.3265	9642.72	596038.35	34112.77511	15088.73571	13047.02769
IGF-2R	11.24584977	10.59130818	0.083762588	0.3129513	1.574115701	3482	P11717	4069.25594	1928.7061	1799.6607	1952.975928	1498.623564	1252.149554
CD99	13.49099447	13.91966458	0.083927856	0.3129513	0.742946329	4267	P14209	9829.03858	11185.647	13785.645	15958.92109	20464.14995	11392.68031
TLR2	10.12627102	11.48288534	0.084640941	0.3129581	0.390497625	7097	O60603	1165.5548	740.6127	1646.9852	3619.927063	7521.790303	859.7559657
IL-34	7.380805026	5.425180029	0.086836739	0.3168525	3.878839274	146433	Q6ZMJ4	91.7666901	178.75333	148.79773	91.1043388	8.069688002	93.96275928
JAM-B	4.209762591	8.556418076	0.087134433	0.3168525	0.049150316	58494	P57087	413.128586	282.08969	0	548.0277867	471.1618179	204.8402495
ANGPTL3	10.95071034	10.58457849	0.090658466	0.326965	1.288892421	27329	Q9Y5C1	1813.82399	1747.5841	2024.9764	1863.451699	1593.051349	1217.353762
IL-1 RII	15.53440767	15.20469853	0.093227743	0.3302535	1.256759976	7850	P27930	48146.3134	46363.956	40450.11	42158.79147	31382.72552	40700.27789
JAM-A	4.311653114	8.668827994	0.093804445	0.3302535	0.048793241	50848	Q9Y624	425.834429	363.32438	0	461.5500075	427.3600158	339.2233697
TLR4	7.092606209	11.34018819	0.093822021	0.3302535	0.052644186	7099	O00206	652.275189	617.74071	857.50734	4685.888965	2906.386044	1277.860107
IL-12p70	9.965662188	9.336633158	0.095345124	0.3303296	1.546523794	3592	P29459 & P	750.270501	662.48128	1771.8762	625.6407314	752.3736499	571.5202739
Ferritin	17.59746612	17.21127915	0.094647152	0.3303296	1.306934614	2512	P02792	230889.067	161361.93	212964.37	187448.4348	135610.7521	137451.4244
ANG-2	13.83988483	12.71144769	0.09619296	0.3306633	2.186217799	285	O15123	33581.9718	7397.4531	13027.369	10268.66328	3956.677402	7422.086743
CTACK	13.48031202	12.91159786	0.100640571	0.3406296	1.483201041	10850	Q9Y4X3	9811.41535	9850.9765	12105.633	12360.39652	5424.29355	6819.935792
TFPI	12.69220231	11.43584023	0.100373684	0.3406296	2.388925861	7035	P10646	3654.5854	6759.0543	10640.439	6019.310084	2448.500444	1440.771452
IGF-1	11.14632092	10.77597882	0.102619594	0.3420653	1.292659324	3479	P05019	1970.04912	2654.0822	2675.8889	1853.953982	1654.263943	1754.812648
2B4	10.33561017	11.53377399	0.10191665	0.3420653	0.435829628	51744	Q9BZW8	1377.15417	872.86073	1734.4032	2377.36479	8694.852547	1259.216526
CD58	5.161753203	10.15681519	0.105552356	0.3491958	0.031357145	965	P19256	2236.35411	732.89757	0	1146.83613	1193.95654	1083.641204
BCMA	14.19568971	14.45067799	0.111093522	0.3569925	0.83799394	608	Q02223	19041.9203	17399.397	19696.199	20587.179	24706.68466	22069.5224
MMP-2	11.30447451	10.3891712	0.110378467	0.3569925	1.885965533	4313	P08253	3248.39771	1020.5347	4613.5943	1667.878132	962.8460913	1498.42301
Galectin-1	12.87606756	13.48811532	0.111154494	0.3569925	0.654267378	3956	P09382	5118.08999	6110.5818	10912.614	14577.54002	14280.26962	7285.199089
Desmoglein 2	15.31051775	15.05392405	0.110620574	0.3569925	1.194654719	1829	Q14126	45788.0829	40385.245	40617.119	34005.87833	35902.27057	32235.45607
PF4	15.03623658	15.76451798	0.113120261	0.3571391	0.603622546	5196	P02776	65009.191	46002.187	18975.575	46979.84396	59420.60536	61788.28159
Adipsin	15.88177924	15.64472768	0.113175673	0.3571391	1.178581532	1675	P00746	62234.7097	55959.12	64845.182	53524.90442	51116.54645	49142.1399
LAG-3	8.848327822	9.176698538	0.113635169	0.3571391	0.796435417	3902	P18627	559.787903	490.70051	467.64863	594.4325711	624.1148565	519.7054889
TRAIL R2	13.85642162	13.07877728	0.115317116	0.3598548	1.714329398	8795	O14763	26753.753	10892.483	19661.384	7227.526032	11942.1753	7500.415943
bFGF	11.15269688	9.998182856	0.118081391	0.365886	2.226093241	2247	P09038	5907.90722	3807.8409	650.72391	968.7169335	885.1627462	1243.802229
G-CSF	9.55453776	8.91729809	0.127292371	0.3902134	1.555350435	1440	P09919	476.401002	1522.2484	612.82742	372.8042285	529.2833173	569.1254686
EG-VEGF	10.30532315	11.45510321	0.127706201	0.3902134	0.450693932	84432	P58294	669.517082	2212.8068	1128.6179	3873.233163	2789.345892	2046.129029
IGF-2	12.42637839	11.61518861	0.128841329	0.3909668	1.754657897	3481	P01344	11954.3336	3419.0808	5038.4133	4501.073429	3091.734571	2216.20224
IL-4	10.46421048	10.13349075	0.130327619	0.3927682	1.257640626	3565	P05112	1270.89129	1180.2607	1980.5667	1162.420947	1153.214663	1054.432753
FGF-4	10.20029317	9.449842493	0.133132532	0.3957994	1.682318285	2249	P08620	823.385605	1490.2931	2048.8348	758.5577819	736.7329209	609.3769889
Thyroglobulin	12.25733782	11.18896211	0.13304723	0.3957994	2.097071002	7038	P01266	3139.50351	2820.2209	16308.215	1189.279199	3192.647846	3346.370427
MMP-1	10.2040287	8.112718586	0.134471744	0.3962343	4.261348717	4312	P03956	1992.34501	152.99614	2112.3105	183.2671782	1346.564512	84.41170105
Cadherin-4	7.61007822	11.67746954	0.135079876	0.3962343	0.059647632	1002	P55283	2944.04438	526.17633	937.42486	4556.800768	7103.820457	1084.168973
MPIF-1	14.96836611	14.33706489	0.138467552	0.3967295	1.54896143	6368	P55773	49640.2575	26226.077	18875.896	23106.97071	14261.34502	26896.1952
EDA-A2	6.54475756	4.273964118	0.138855316	0.3967295	4.825884682	1896	Q92838	112.894666	83.333401	82.374731	81.96852767	0	86.26916423
IL-1 F5	5.137249772	7.831665079	0.138378594	0.3967295	0.154489928	26525	Q9UBH0	0	85.496253	174.3205	301.2279949	290.5087681	133.1874816
BMPR-IA	11.69638546	12.0211962	0.137036107	0.3967295	0.798403122	657	P36894	4257.37777	2888.0309	3323.9242	3630.43553	3812.894856	5184.311644
IL-1 RI	10.98238993	10.73335783	0.141872383	0.4003687	1.18840955	3554	P14778	2025.14892	2157.9379	1727.5244	1771.931773	1697.60638	1637.336882
Resistin	11.85547436	10.52956354	0.14285883	0.4003687	2.506911072	56729	Q9HD89	5389.81783	15581.085	2340.4051	1716.081055	1200.581924	1564.305871
FGF-6	6.578960276	5.324727718	0.142811389	0.4003687	2.385402233	2251	P10767	101.185185	84.86012	97.369357	86.91709203	7.120312943	89.17002794
SIGIRR	2.496790782	5.115902196	0.146757731	0.406122	0.162767953	59307	Q6IA17	11.2003727	82.188362	0	81.71475513	4.98421906	83.24217601
CD155	10.7396396	9.450232416	0.146755804	0.406122	2.444275978	5817	P15151	1033.07307	1619.3376	619.29658	940.3201541	519.6427442	697.4464147
Cystatin B	14.74599047	14.50747107	0.150607853	0.4141716	1.179781262	1476	P04080	29721.1045	23059.429	30779.04	23691.01497	24080.85305	22143.19798
IL-15	11.24605759	10.97642064	0.157019784	0.4238571	1.205504428	3600	P40933	2112.89952	2175.0061	3024.1477	2028.720552	2257.361478	1783.291732
Adiponectin	19.11775283	18.87643883	0.157014163	0.4238571	1.182068795	9370	Q15848	636031.747	537477.73	541637.99	482846.4983	508586.6907	453885.2914
uPA	11.45883998	11.83673148	0.155623048	0.4238571	0.769561482	5328	P00749	3343.15595	2584.8425	3038.0578	3245.734875	2827.506019	5327.803981
SCF	11.19239595	10.50058889	0.162346593	0.435564	1.615305508	4254	P21583	1949.32797	5060.4896	2310.6583	1928.32697	1046.617256	1503.416917
DR6	16.22341649	15.90483488	0.173357607	0.448867	1.247103848	27242	O75509	76277.1733	55029.102	100873.46	67832.63855	62309.74185	54636.28268
IGFBP-3	13.77668469	14.26755639	0.174294217	0.448867	0.711595011	3486	P17936	13332.3784	22395.418	8449.2288	22546.69106	17825.11955	19085.59336
TGFb3	10.48742954	11.23554319	0.174615117	0.448867	0.59538152	7043	P10600	1240.18869	3075.8754	1406.9706	1566.152699	3174.799191	2815.710458
Notch-1	10.38601959	10.08949184	0.173887171	0.448867	1.228184883	4851	P46531	1495.98935	1444.6285	1280.4171	1088.7264	1371.385492	863.8194743
MMP-7	7.84931855	7.226498134	0.172042062	0.448867	1.539882655	4316	P09237	194.479199	381.80693	122.50928	186.6496969	98.73500614	178.4661804
IL-32 alpha	6.334384561	9.277978969	0.175466188	0.448867	0.129983967	?9235	P24001	419.404777	283.75136	353.18126	887.839428	1090.923914	245.5098163
ICOS	4.725545659	8.534361774	0.17341633	0.448867	0.071356264	29851	Q9Y6W8	722.094423	676.53065	0	949.3701543	398.4404283	133.2616116
Testican 2	4.388068007	8.929368437	0.170448617	0.448867	0.042946952	9806	Q92563	423.570021	451.70058	0	456.899313	612.1854241	411.7256029
CEACAM-1	14.82871394	14.3499216	0.181950107	0.4523054	1.393576629	634	P13688	32405.4751	21806.701	32942.487	13800.55113	27686.04615	23825.8148
HCC-4	16.22811762	15.71659648	0.179162509	0.4523054	1.42555247	6360	O15467	67611.7041	58984.417	89576.824	31025.78541	56643.44178	88838.64685
LIF	10.22111512	9.939384254	0.181434527	0.4523054	1.215652482	3976	P15018	1195.88952	1051.7613	1164.3888	1159.939054	820.4133948	991.6295396
Tie-2	8.632963675	9.693599319	0.181324566	0.4523054	0.479420783	7010	Q02763	879.729116	565.2448	504.80808	785.7121316	1204.201141	597.848687
FGF-19	8.577368652	8.070930536	0.178066156	0.4523054	1.420538681	9965	O95750	287.144587	575.68884	399.13558	318.3934348	235.2774999	256.6488457
Eotaxin-3	8.538851386	8.022561985	0.183040697	0.4524601	1.430271866	10344	Q9Y258	280.517295	382.67006	699.3018	256.5249832	211.8846079	319.7242287
EMMPRIN	13.98922647	13.64401713	0.187175646	0.4600966	1.270335302	682	P35613	23500.8104	13627.354	12595.314	11071.87993	12260.74667	15450.20387
NrCAM	11.67461704	10.91342579	0.192930992	0.4716091	1.694889539	4897	Q92823	9306.99425	2354.3569	3594.8683	1866.287018	2044.733289	1877.21569
ADAM12	6.775228566	9.732520838	0.198795388	0.4832595	0.128755657	8038	O43184	798.203679	627.94287	285.36467	802.8104297	1187.197805	643.6140013
MIP-1b	13.65505762	12.95545206	0.20037652	0.4844268	1.624060712	6351	P13236	4713.56038	13943.325	18988.649	6701.071607	10458.54695	7147.535761
TGFb2	11.29494549	11.55765216	0.205030474	0.48764	0.833522666	7042	P61812	2650.94112	2475.3594	2772.7919	2909.783355	2453.829707	3832.263593
Serpin A4	17.53498954	17.77999336	0.204163961	0.48764	0.843813562	5267	P29622	195902.091	164536.98	201144.99	242186.3869	221924.7803	212115.6186
TGFb RIII	11.61665723	11.12740497	0.203001212	0.48764	1.403717153	7049	Q03167	4906.27236	2732.6994	1791.1878	2473.267092	2068.688254	2185.233245
TRAIL R1	9.984673659	10.34117903	0.208822475	0.4939887	0.781054229	8797	O00220	1319.91927	1184.3506	955.96643	1154.549424	1690.484522	1115.757006
MCP-2	9.881811366	8.594865205	0.210210949	0.494614	2.440109958	6355	P80075	499.325399	3287.7069	1649.4116	400.5652574	275.7712078	519.067441
NAP-2	16.03155961	16.26388262	0.216226576	0.5060622	0.851263095	5473	P02775	74201.2076	70254.709	60532.342	96795.06427	70270.6201	71631.79128
Fas	11.56248561	11.21128426	0.219956836	0.5093737	1.275622413	355	P25445	3682.30759	4085.0562	2792.0033	2654.891783	2493.975163	2010.508872
ACE-2	9.041256556	9.64000298	0.219223073	0.5093737	0.660327473	59272	Q9BYF1	507.708524	416.56121	1121.7537	619.4676763	923.8584922	884.1068778
HB-EGF	9.36497655	10.31352955	0.225671971	0.5176251	0.518151901	1839	Q99075	357.019774	1205.4541	651.78178	403.7390811	1438.002591	3537.413199
RBP4	18.53566189	18.34729724	0.225872772	0.5176251	1.139471351	5950	P02753	410102.476	374053.73	399520.52	341832.9551	332705.3253	326124.0544
Renin	15.30248134	14.16528261	0.230591707	0.5257013	2.199535275	5972	P00797	16657.7915	38141.938	25470.845	25291.22945	10241.22371	23940.06764
ADAMTS13	10.85365505	11.15044054	0.232906216	0.5257644	0.814064218	11093	Q76LX8	2097.14905	1565.5334	1927.7386	2023.135766	2839.425523	2041.810869
Angiotensinogen	12.56379035	11.77508819	0.233009212	0.5257644	1.727519694	183	P01019	6180.05643	4629.1182	3511.9652	2649.722777	6331.389831	2563.674693
MDC	8.377924516	9.216448487	0.234251834	0.5258715	0.559215413	6367	O00626	98.9653943	340.29471	734.51758	354.184017	757.8354589	780.0544534
TGFb1	12.55804511	12.91411784	0.238567542	0.5328412	0.781288493	7040	P01137	4844.61833	8422.2328	5427.5478	7772.269199	8773.608309	6740.849714
Follistatin-like 1	11.58958398	11.85548326	0.240306626	0.5340147	0.831680155	11167	Q12841	3123.32027	3341.3042	2755.0937	4694.106088	2951.481273	3669.588212
CEA	15.79022816	15.57575052	0.242197657	0.5355124	1.160283732	1048	P06731	64030.3471	47778.03	64904.845	52286.81106	47682.11892	46722.91438
TIM-3	5.389540435	9.92276112	0.244296013	0.5374512	0.04318815	84868	Q8TDQ0	3612.98812	853.41057	0	1970.260449	816.5071834	566.4278203
IL-23	11.66603687	11.42324363	0.247714984	0.5410924	1.183281424	51561	Q9NPF7	2497.02273	2964.1656	3902.5644	3043.08957	2722.400632	2497.319526
Prostasin	7.368146125	6.500323951	0.248410597	0.5410924	1.824906026	5652	Q16651	197.279292	86.895745	386.4147	93.13451905	85.44375531	90.17902401
GITR	9.869034382	9.437385122	0.252319165	0.5423368	1.348774587	8784	Q9Y5U5	1880.72449	785.76067	815.22209	710.0262232	805.6552226	580.0780082

ErbB2	7.95321307	7.515867441	0.255915091	0.5423368	1.354110647	2064	P04626	206.759369	191.90568	314.78433	122.096558	184.2250548	267.8803027
GROa	8.168280089	7.6503415	0.256724624	0.5423368	1.431907789	2919	P09341	278.09903	151.19478	472.24134	172.3176372	191.7649615	241.70345
IL-3	10.57155764	10.16320457	0.257329785	0.5423368	1.3271699	3562	P08700	1385.88809	907.52893	2701.0233	1266.721204	1064.509167	1115.11086
Galectin-3	12.68838527	10.55060994	0.254099177	0.5423368	4.400829028	3958	P17931	18411.6915	12412.303	23289.561	1232.315596	208.5499759	13054.34519
CA19-9	13.93171815	12.02616817	0.253391471	0.5423368	3.746516958	30815	Q969X2	30381.6445	2276.0373	154706.97	29870.38788	1366.442122	1775.428371
SLAM	2.310253645	5.716132303	0.257609957	0.5423368	0.094347058	6504	Q13291	604.093627	0	0	431.2576459	335.0772873	0
MIP-3a	10.0085189	8.843385317	0.272550169	0.5571127	2.242539786	6364	P78556	322.387269	9039.1268	720.07314	392.6763661	547.1405456	448.0983508
OSM	5.953094303	8.125381991	0.268563283	0.5571127	0.221858589	5008	P13725	368.425372	180.7372	218.41648	274.5337436	327.1062876	239.8583855
Cathepsin L	11.56890527	12.20550372	0.273491677	0.5571127	0.643227746	1514	P07711	5943.8202	2585.3122	2728.6916	5935.065343	8126.67307	2182.751908
CD163	14.91966722	14.43432456	0.270950016	0.5571127	1.399918329	9332	Q86VB7	42863.2921	43259.042	21079.948	32477.30427	22933.5962	14567.32207
CHI3L1	13.68759394	13.45615582	0.269713993	0.5571127	1.174004647	1116	P36222	12734.6965	13052.174	12443.37	11918.17317	11129.76116	10698.13311
CD84	11.56123454	11.90599762	0.271799823	0.5571127	0.787437277	8832	Q9UIB8	3834.27107	3840.8577	2442.3669	4851.051467	3454.23351	3370.199351
CF XIV	6.11544192	9.96218472	0.269971609	0.5571127	0.069504837	5624	P04070	6407.0155	3604.8052	0	748.3847343	2195.289132	602.0522115
ENA-78	12.22248826	10.94114598	0.27897952	0.5588232	2.430650193	6374	P42830	949.860166	3152.6355	16819.056	3650.78848	438.1645204	4738.216183
TWEAK	8.113986714	8.466876695	0.277629429	0.5588232	0.783014006	8742	O43508	252.899325	481.04365	209.22288	377.1059818	310.80166	374.7159166
CD229	8.146548501	11.08997798	0.276622145	0.5588232	0.129998828	4063	Q9HBG7	4966.22327	1158.7102	1118.3314	2711.480599	2059.724504	1851.940574
Siglec-10	2.321038581	6.218922968	0.279411622	0.5588232	0.067084144	89790	Q96LC7	622.460489	0	0	1150.355574	357.9530059	0
IL-12p40	8.593942595	9.023316769	0.282367107	0.5621789	0.74258384	3593	P29460	337.389633	301.5582	689.59158	776.7156662	346.6017997	520.1517012
IL-13 R1	11.62973628	11.43268912	0.284768605	0.5642517	1.146349654	3597	P78552	2910.33247	3128.5784	3224.449	3265.560871	2208.097295	2926.126375
GASP-1	16.29222027	15.97944889	0.287255432	0.5642517	1.242091441	124857	Q8TEU8	109458.294	69781.756	101816.3	57862.78003	78538.53578	59344.13948
Syndecan-4	14.59996253	14.01226476	0.286477505	0.5642517	1.502846615	6385	P31431	12329.6994	19690.039	46955.529	17617.33362	10962.92819	23355.35088
Prolactin	13.33731596	12.49304463	0.289051877	0.565257	1.795357729	5617	P01236	9543.25022	14738.428	9046.7744	19315.11271	5032.259427	1969.491041
Endoglin	15.88464957	15.609871	0.290463773	0.5655047	1.209808386	2022	P17813	74830.1803	49760.533	81992.985	50294.56874	47221.54318	52653.93774
IL-17R	11.33766983	11.6847091	0.293364837	0.5661427	0.786195893	23765	Q96F46	2407.45562	2173.051	4280.9876	2577.8902	3009.372537	4594.05477
Osteoactivin	14.7068535	15.03318804	0.292674905	0.5661427	0.797560279	10457	Q14956	30810.687	26867.262	26500.766	42413.37131	45170.89584	19675.15515
Siglec-5	17.60743492	15.25023149	0.295740121	0.5682343	5.123761893	8778	O15389	154598.804	227746.42	223603.02	1175.163024	224305.0203	224399.0265
E-Selectin	13.12264928	12.76685318	0.324182069	0.5858922	1.27969154	6401	P16581	13082.8377	5967.3346	8204.1021	4467.242991	9739.492828	7777.56636
IGFBP-4	11.62615684	10.98901166	0.321029675	0.5858922	1.555248576	3487	P22692	3967.18973	2837.0736	2528.9768	4196.527991	1155.457198	1728.59026
PIGF	10.50716307	9.946924321	0.321728384	0.5858922	1.474513216	5228	P49763	980.614313	1847.1456	2437.8675	543.2614067	2130.374208	827.8968896
CA15-3	13.05202276	12.7606069	0.325003228	0.5858922	1.223840765	4582	P15941	6439.55615	8512.6622	11447.41	9490.988754	6589.878495	5340.654523
MMP-10	9.99018966	10.6246134	0.30819864	0.5858922	0.644198077	4319	P09238	2040.09093	1346.7023	1247.983	1258.157317	1810.708605	1724.097462
BMP-2	6.553575037	8.695473343	0.317360859	0.5858922	0.226581455	650	P12643	339.771594	393.77354	0	328.8436882	538.8168452	399.1661028
Chemerin	13.04261497	14.27187748	0.315312715	0.5858922	0.42653543	5919	Q99969	17127.7866	23117.145	631.14843	21018.85253	16661.6624	22100.1864
MBL	17.49843925	17.66357871	0.3208326	0.5858922	0.891842305	4153	P11226	192956.07	200621.8	193616.75	232709.4999	201004.9089	191326.002
ANG-4	8.518298528	7.838936104	0.325512963	0.5858922	1.601431871	51378	Q9Y264	237.36682	286.26368	1467.7715	189.8797836	222.6852178	280.1042979
Cystatin C	16.84897329	16.67089357	0.316337767	0.5858922	1.131376978	1471	P01034	121208.082	100936.42	126239.68	111513.1315	114953.0129	88604.97612
VE-Cadherin	7.783122198	8.297420239	0.325818271	0.5858922	0.700133508	1003	P33151	331.842479	254.99227	265.33256	203.0625202	355.2853212	427.3077766
WISP-1	5.948327152	7.854354791	0.326072286	0.5858922	0.266826223	55515	Q96FT7	214.598448	183.19833	365.07348	242.0060172	270.9034372	186.5674356
Marapsin	9.040173176	8.63541257	0.32623545	0.5858922	1.323869216	83886	Q9BQR3	349.120709	481.55256	794.77897	696.6055989	291.9328307	306.7348061
CNTF	11.56854589	11.82706865	0.322186281	0.5858922	0.835943441	1270	P26441	4292.67235	2563.1871	2895.8064	2997.443463	4724.337611	3384.155067
S100A8	11.24343929	10.65167451	0.317019791	0.5858922	1.507089179	6279	P05109	1377.61516	2659.0694	6150.0104	1243.443261	1910.466558	1749.164163
Cystatin A	7.907538028	10.53703551	0.317936174	0.5858922	0.161600382	1475	P01040	3768.79195	925.20826	950.91576	963.8250156	1620.406726	2095.769741
IL-10 Rb	9.904093099	9.487837804	0.333400823	0.5869037	1.334459291	3588	Q08334	599.745069	991.84954	1036.4894	540.7378596	989.0057716	689.1514497
NRG1-b1	8.596582996	8.204707988	0.335428626	0.5869037	1.312097572	3084	Q02297	502.335887	485.62033	294.4558	522.3828311	238.8426855	203.5708172
Lymphotactin	6.602794552	7.42996658	0.333432801	0.5869037	0.563632994	6375	P47992	176.938129	229.70539	114.71988	262.5096593	102.3137354	187.3535537
MSP	11.59670518	11.38107969	0.333980966	0.5869037	1.16120726	4485	P26927	3287.54274	2961.3004	3080.3659	2674.470172	2477.301061	2860.441878
Siglec-9	7.936160174	10.40951842	0.336135749	0.5869037	0.180071499	27180	Q9Y336	2236.65892	1235.4979	1299.4743	1372.791059	1364.22046	1340.554679
BAFF	8.205016777	7.425238476	0.331251789	0.5869037	1.716867023	10673	Q9Y275	243.516898	114.40123	1332.9343	132.5530209	124.1100675	302.8872954
ULBP-1	5.962094845	8.132287286	0.332601168	0.5869037	0.222181031	80329	Q9BZM6	122.592511	85.443079	0	109.3541684	1086.856585	183.0038171
MCP-4	8.290305069	8.557039643	0.341281505	0.5911963	0.831198771	6357	Q99616	281.324705	448.17098	298.4388	482.582524	265.4208648	413.7037747
Activin A	10.17426684	9.91600508	0.340481458	0.5911963	1.196036782	3624	P08476	1591.54726	985.50522	1213.1515	776.8839054	1307.660852	884.7333601
Midkine	12.68325315	13.00894816	0.344387864	0.5942379	0.797913903	4282	P14174	6600.49655	5651.4858	8785.146	11135.89949	10309.4105	4876.649302
MCP-1	12.68145754	12.92869551	0.361736946	0.6156306	0.842507847	6347	P13500	7868.80086	4570.9673	8903.0992	8331.306398	6261.442089	9082.777887
MIP-1a	11.60614308	11.95498726	0.359797549	0.6156306	0.785212922	6348	P10147	4185.7325	2541.0469	3127.1429	3909.621576	6500.888371	2460.183438
FGF-7	10.2262405	9.893648356	0.362382554	0.6156306	1.25927393	2252	P21781	977.954166	1406.063	1841.4918	870.7594178	944.0962215	1043.677138
PAI-1	14.4828823	14.02150414	0.361732816	0.6156306	1.37685645	5054	P05121	9673.4133	23779.138	37559.711	15209.39421	13273.04469	22778.08362
SDF-1a	9.239153492	9.414094306	0.365617593	0.6187375	0.885803858	6387	?P48061	563.341475	581.75668	745.02262	762.6381657	607.9339396	681.8361392
ErbB4	2.652917417	6.082179424	0.367298348	0.6192003	0.092830197	2066	Q15303	1563.69925	0	0	246.612504	1254.980119	0
MMP-13	13.36391228	12.99446058	0.383057334	0.6433024	1.291861761	4322	P45452	11493.0148	7961.0835	7150.9349	11525.52358	9118.134892	5169.294157
TIMP-1	16.40675378	16.53905408	0.38469967	0.643604	0.912375554	7076	P01033	91132.3357	96316.948	81237.565	86326.00216	98648.98163	101392.582
IGFBP-6	15.22924437	15.08652983	0.392715624	0.654526	1.103980385	3489	P24592	40407.358	46070.49	33672.747	33075.31429	34480.72556	36929.73277
IL-1 F6	5.716642687	4.317943133	0.399208195	0.6620812	2.636638083	27179	Q9UHA7	92.9121827	81.933909	9.1881962	89.39137421	0	86.77366227
ULBP-2	12.04091145	11.75062019	0.400258207	0.6620812	1.222887138	80328	Q9BZM5	6556.28166	3664.7657	3222.1428	3650.020223	3909.473955	2864.654306
CD40L	11.79404901	11.57288426	0.406884553	0.6658314	1.165674306	959	P29965	4005.05783	3217.8592	3055.759	4175.864909	2372.232211	2851.103174
IL-1 F8	7.871124966	7.651410277	0.405652922	0.6658314	1.164503269	27177	Q9NZH7	154.896064	235.49638	322.60778	179.7343954	189.7563399	234.7177114
LRIG3	11.00886143	11.16965357	0.407065111	0.6658314	0.894533774	121227	Q6UXM1	2456.85341	1995.3074	1872.0133	1836.526897	2433.313409	2731.700915
MCSF R	15.75714068	15.61538784	0.416465693	0.6663451	1.103244721	1436	P07333	61683.4965	53493.408	56939.202	43864.30135	50896.98686	56659.27492
VEGF-D	10.44583633	9.764486289	0.410131516	0.6663451	1.603639701	2277	O43915	742.881146	1245.7045	5060.7305	1434.919985	592.9455575	770.494647
PARC	15.66249962	15.80719617	0.415870842	0.6663451	0.904569622	6362	P55774	48994.5602	63479.305	48935.846	62595.08823	52342.87426	57530.74172
ANGPTL4	13.27776302	12.99964609	0.41404363	0.6663451	1.212611097	51129	Q9BY76	7013.20521	7776.9837	16766.967	9414.280797	8325.313697	7006.517137
Decorin	16.97128028	16.78163541	0.413509166	0.6663451	1.140482941	1634	P07585	145005.611	117222.05	136830.31	112647.4515	149152.822	85106.3937
IL-5 Ra	6.760718066	6.996908028	0.413813889	0.6663451	0.848984452	3568	Q01344	102.330678	140.20368	107.83369	181.3204737	101.1677797	110.8634435
Thrombospondin-5	17.17128465	16.81381598	0.419679592	0.6690544	1.281176001	1311	P49747	210745.832	110904.81	197528.58	141447.3062	175459.749	61603.31317
Contactin-2	9.734782124	9.405096043	0.431942942	0.671349	1.256739888	6900	Q02246	551.584504	1212.0524	1159.4158	554.3602326	855.9378933	653.823019
MICB	8.93277693	9.26397873	0.437711736	0.671349	0.794874059	4277	Q29980	543.910882	454.64455	578.19494	343.4372922	965.3366767	697.1806384
Cripto-1	10.52846166	11.22436954	0.435312096	0.671349	0.617320722	6997	P13385	2335.82819	892.85607	1556.4916	799.963411	1846.799512	9253.39885
VEGF-C	9.928333382	10.26693926	0.426821466	0.671349	0.790805125	7424	P49767	903.485409	1005.6622	646.37601	1785.571807	657.699245	1588.506825
BCAM	11.69700349	11.39658613	0.432066341	0.671349	1.231500625	4059	P50895	2959.54886	2580.9993	6403.9869	3577.991919	2046.456356	2673.0372
hCGb	11.57167683	11.90249221	0.435626498	0.671349	0.795086994	1082	P01233	3129.73095	2566.7085	3796.3483	3176.834992	2464.167189	7161.156606
PSA-free	12.0777514	13.08817991	0.436587458	0.671349	0.496398784	354	P07288	2636.49608	3798.4835	32379.076	11524.78954	31766.7581	1802.62802
Transferrin	18.45729221	18.33095022	0.424158863	0.671349	1.091522593	7018	P02787	405790.112	333890.14	363914.92	326694.4646	376317.0128	291607.0333
AMICA	10.52895836	10.8493707	0.437902638	0.671349	0.800840953	120425	Q86YT9	1922.6189	1055.6706	1805.7087	1880.535513	2405.46698	1385.965685
Furin	10.88669366	10.64941446	0.436568848	0.671349	1.178767508	5045	P09958	1939.2372	1290.4668	2778.2731	1281.023356	1893.342826	1705.180796
Layilin	4.221830439	5.573178835	0.441118301	0.6739307	0.39192557	143903	Q6UX15	107.803587	84.732894	11.995701	94.91092677	11.15515694	91.56639361
PDGF Rb	11.29667731	11.11436019	0.448413702	0.675007	1.134704882	5159	P09619	2526.68308	2614.0188	1958.8781	2587.86715	1955.021944	2150.71086
IGFBP-1	15.28973734	14.87782539	0.448736818	0.675007	1.330447847	3484	P08833	46429.6516	28849.883	46165.166	35169.43348	20101.86027	38598.31159
DPPIV	13.22740527	12.89979382	0.449493268	0.675007	1.254933967	1803	P27487	17721.917	5498.7322	8191.0217	10415.83978	7341.279988	5834.898165
IL-17E	10.31528826	10.6107367	0.44868602	0.675007	0.814819015	64806	Q9H293	1762.97693	1437.8579	1397.0762	1223.858568	2672.421664	1166.606929
RGM-B	11.23545836	11.0852614	0.445023184	0.675007	1.109720967	285704	Q6NW40	2299.65484	1937.8785	2517.3071	2469.39579	1915.025571	2165.809494
IGFBP-2	15.44732069	15.32251607	0.454265966	0.6798538	1.090360059	3485	P18065	46184.638	48142.678	43054.623	35625.47884	41792.7321	46208.02252
TACE	6.019328501	7.610519152	0.4569858	0.6816059	0.331897427	6868	P78536	121.73813	111.20442	1284.2993	183.634202	244.0416483	163.9801078
BTC	9.962075618	9.815163771	0.458878637	0.6821169	1.10719693	685	P35070	968.661284	1091.2944	1181.6983	913.3431929	840.9951113	948.6363194
Leptin R	4.087592819	5.306915796	0.464719476	0.6884733	0.429484218	3953	P48357	0	88.549691	9.6986516	86.79020576	7.120312943	86.01691521
DcR3	10.46726015	10.61042547	0.467997315	0.6910027	0.905530212	8771	O95407	1584.50943	1478.6113	1362.4481	1522.072622	1989.130096	1259.569874
ALCAM	14.73015135	15.00561302	0.472758001	0.6933784	0.826185893	214	Q13740	34220.6874	32946.367	37118.829	35343.59861	29767.68076	33831.66634
CD23	9.604238927	10.13970916	0.471314013	0.6933784	0.689933762	2208	P06734	716.823854	463.61361	351.83135	1889.590269	1067.33491	709.8287373
IL-2 Ra	9.893497022	9.763898296	0.48181173	0.7043095	1.093989375	3559	P01589	856.719094	790.85701	1053.5727	818.250451	944.3662594	847.5198332
CD200	12.12217206	12.01169159	0.485448303	0.7072757	1.079587719	4345	P41217	4526.28632	4278.5147	5224.8806	4255.831768	4033.221015	4099.078994
BMPR-II	9.84475543	9.99668363	0.499052864	0.7246972	0.900046722	659	Q13873	1122.29264	1128.7222	864.6325	1001.652362	1028.777363	1031.787962
CRP	14.61935507	14.47529289	0.50624232	0.7327191	1.105012115	1401	P02741	28337.9003	22983.047	27094.539	19805.33406	25224.50668	23649.92149
ST2	10.42175773	10.12751906	0.510787307	0.7368735	1.226237704	9173	Q01638	2653.11774	1555.85	864.52162	948.6559637	802.405559	1833.678296
FABP2	14.2472548	14.11925685	0.527056823	0.7553909	1.092776187	2169	P12104	25073.0094	21187.607	16005.092	19749.2146	15999.24644	17833.5024
B7-H1	11.19590243	11.40199405	0.526145221	0.7553909	0.866882512	29126	Q9NZQ7	2830.20001	2413.2689	2148.6041	2075.115267	4321.224849	2207.349656

Troponin I	9.588238001	9.76725875	0.530411135	0.7577302	0.883302348	7135	P19237	986.760304	1023.4871	752.45901	846.5977615	832.1531421	936.1280556
Persephin	11.94154325	12.09939269	0.532565664	0.7583459	0.896360239	5623	O60542	5645.00557	3971.1367	3720.4897	4521.723835	4882.823314	3824.43103
Insulin	11.21318337	11.66394084	0.538501074	0.7618665	0.731658595	3630	P01308	852.087194	3489.6957	3259.8346	8725.924727	1860.982458	2101.574377
IGF-1R	9.208191134	8.779399327	0.537396062	0.7618665	1.3461058	3480	P08069	362.143014	346.70319	1574.8295	293.1296122	1096.679436	261.7685265
GH	12.93475407	12.45017752	0.544114062	0.7664287	1.399175134	2688	P01241	15271.6259	10204.952	9169.7723	4055.954869	2453.025314	17600.57125
TECK	8.183828321	8.412721958	0.545209488	0.7664287	0.853289004	6370	O15444	340.611666	231.90168	444.91257	507.8813823	208.4344936	370.3472316
IL-9	10.21204277	10.09305044	0.561411831	0.7866917	1.085976084	3578	P15248	1166.93811	1354.1378	1124.8755	1445.027265	1054.009929	853.0823738
L-Selectin	15.67120185	15.59896253	0.58715851	0.7948072	1.051347296	6402	P14151	52086.5965	52816.207	52507.186	49762.20896	48861.39244	50277.66843
TIM-1	12.34409935	11.74553706	0.574691986	0.7948072	1.514206835	26762	Q96D42	34580.4314	1661.7008	3833.788	3946.139256	7679.092449	1334.451176
SCF R	15.18176629	15.2683003	0.587208838	0.7948072	0.941782614	3815	P10721	36635.2691	42477.147	35268.284	42189.79993	37588.46697	38757.34189
VEGF	11.11698224	11.35635315	0.585252852	0.7948072	0.847114622	7422	P15692	1821.22087	2903.4289	3551.1462	3489.575956	1577.276449	3270.362312
IL-18 Rb	8.909548551	8.519261167	0.587974083	0.7948072	1.310654459	8807	O95256	447.722685	256.51473	1057.4523	282.6082655	1108.114961	156.0232671
APRIL	8.695303302	8.88139153	0.582080212	0.7948072	0.878985805	8741	O75888	500.07782	450.39186	311.5951	295.0500116	451.6073186	781.7248228
IL-1 R6	11.27335413	11.16651254	0.587633114	0.7948072	1.076868131	8808	Q9HB29	2340.44666	2169.3191	3336.7	2264.854919	2484.279437	2155.544069
SOST	8.475497292	7.934716162	0.571604246	0.7948072	1.454759967	50964	Q9BQB4	311.560308	255.31802	2289.0963	188.7308295	247.0535652	310.2284835
IGFBP-5	5.78234005	5.199707773	0.588879889	0.7948072	1.497579173	3488	P24593	11.9640345	86.514066	84.990815	91.61188386	5.458906589	81.98093092
IL-1 F9	4.623233733	5.370125809	0.575237023	0.7948072	0.595885862	56300	Q9NZH8	4.83652458	84.605667	7.6568302	78.54259849	9.96843812	80.08906328
IL-1 R5	7.15609905	6.97556138	0.583114848	0.7948072	1.133306172	8809	Q13478	103.603448	107.37922	179.8079	185.8883793	101.8204751	102.7284127
Nectin-4	11.92554023	12.04392888	0.576656643	0.7948072	0.921215986	81607	Q96NY8	4697.80911	4190.1707	3220.1072	4462.353884	4061.960956	4150.906849
IL-29	9.97395177	10.04868714	0.59430645	0.7964813	0.949516275	282618	Q8IU54	1052.51658	1014.1661	991.8912	1129.335597	1102.371015	951.664011
bIG-H3	13.0886227	12.99588566	0.593289144	0.7964813	1.066391386	7045	Q15582	8126.50679	8154.2051	9489.7477	9823.915352	6928.301837	8005.374842
TRAIL	9.300125608	9.494334246	0.595550785	0.7964813	0.874052209	8743	P50591	403.190353	512.65847	811.57403	629.477788	825.9954076	718.557165
FLRG	13.46597557	13.00336032	0.598619394	0.7981592	1.378037598	10272	O95633	55316.624	5025.0883	10079.915	5674.63408	19800.15507	4925.05875
E-Cadherin	10.02132979	9.830323811	0.601409336	0.7994565	1.141559435	999	P12830	1880.10538	804.21403	1172.4697	850.914888	1079.962128	818.3327532
Leptin	11.99215575	11.29238682	0.609630311	0.8079438	1.624244618	3952	P41159	383.782246	4309.4714	4639.2751	3290.734687	1707.537547	2804.382729
ADAM9	12.15793511	11.89452871	0.613518813	0.8096095	1.200309462	8754	Q13443	9833.00447	2865.0768	4901.7767	5694.302362	4696.138899	2061.906697
Cystatin E M	15.4368311	15.27421954	0.614567233	0.8096095	1.119311481	1474	Q15828	74304.1452	37825.268	38405.639	38108.67076	40539.93259	40276.80477
C5a	13.55518331	13.27715416	0.617405798	0.810921	1.212537316	727	P01031	19002.3509	10414.324	12024.877	8212.406279	9931.596568	11990.64821
TGFa	11.75709841	12.05934253	0.622200334	0.8147862	0.810989915	7039	P01135	1625.91005	2228.4312	6565.0254	4265.530246	6590.684011	2763.396142
Albumin	16.20640947	16.08383857	0.62563349	0.8168508	1.08867316	213	P02768	88585.1926	59022.046	80958.752	56329.71243	70984.99616	83797.38391
Cathepsin S	12.91367213	12.68796458	0.629559189	0.8195445	1.169350608	1520	P25774	14464.0999	6419.0195	7086.2686	4569.111527	4987.531808	12602.00821
CD97	12.29751236	12.37873645	0.638571248	0.8263863	0.945255278	976	P48960	6284.8044	4506.0574	4840.4371	5620.879154	5582.539037	4810.958583
DNAM-1	11.00414241	11.1271859	0.63846673	0.8263863	0.918248478	10666	Q15762	2237.70159	1901.5614	2085.3725	1827.535182	2690.537744	2272.769191
PGRP-S	17.79512009	17.86542546	0.645310359	0.8302239	0.952436378	8993	O75594	226576.602	231777.32	224115.61	271722.1959	242012.3341	207070.7432
Dkk-3	17.14672889	17.06733791	0.644896829	0.8302239	1.05657193	27122	Q9UBP4	160853.314	136456.05	150911.63	137819.7056	155558.4569	120816.8438
Syndecan-3	9.953575809	10.19056619	0.650995568	0.8350964	0.848513559	9672	O75056	648.819566	861.05595	667.68561	1747.225622	1206.578886	754.9364667
AR	10.45276366	10.14398034	0.667102753	0.8386435	1.238662652	374	P15514	794.684015	1938.4484	2889.0502	1826.283027	955.05759	828.1578089
IL-2 Rb	10.2787426	10.19611057	0.661353695	0.8386435	1.058948206	3560	P14784	1290.80005	1244.6314	1294.0115	1089.52923	1537.668387	961.1062192
AFP	14.67484024	14.43289188	0.658232863	0.8386435	1.18258867	174	P02771	65161.0319	19087.049	22376.017	33395.7331	24728.75782	13099.15076
Legumain	14.44609417	14.62744681	0.660019728	0.8386435	0.881875781	5641	Q99538	28969.9544	26877.124	19543.196	38844.94906	25147.0866	16596.7824
aFGF	12.032051	11.96366811	0.665976117	0.8386435	1.048540724	2246	P05230	4434.99098	4081.9611	4095.1668	3525.233079	4676.130335	3862.477521
IL-1 F10	7.540113428	7.413915893	0.664552625	0.8386435	1.091413304	84639	Q8WWZ1	157.696157	187.15028	197.54622	239.3074972	104.8466081	193.979495
DR3	3.779101249	5.123376742	0.666418497	0.8386435	0.393851727	8718	Q93038	214.61552	163.74465	0	201.4253505	208.2210296	0
BMP-7	10.95691182	10.80634131	0.67271463	0.8408933	1.110008332	655	P18075	1456.64068	2077.5887	3094.013	1552.84262	2522.275372	1463.626726
6Ckine	9.729246566	9.512996058	0.671283065	0.8408933	1.161710433	6366	O00585	438.76965	1001.6344	1185.6377	979.0386155	517.0412723	767.2170412
Fractalkine	11.35519371	11.4442797	0.678722468	0.8459997	0.940118167	6376	P78423	3541.22315	2518.8274	2493.3884	2607.104513	3100.609001	2673.868056
IL-17C	4.835148012	5.367031764	0.687243389	0.8542008	0.691651043	27189	Q9P0M4	6.61840206	77.608206	11.612859	85.52134311	9.019063061	80.08906328
VEGF R1	9.005909442	9.216302281	0.689442788	0.8545206	0.864301854	2321	P17948	518.40958	942.64133	673.58124	595.0224457	433.4325385	811.7656603
MIP-1d	14.86091353	14.52990544	0.701995885	0.8549403	1.257892032	6359	Q16663	69133.6033	55303.753	6261.1006	37627.54554	14183.73458	24800.24399
b-NGF	10.3764451	10.24510609	0.697266848	0.8549403	1.095309821	4803	P01138	1153.24387	1910.0288	1307.1339	988.6820529	1212.198399	1487.76176
VEGF R2	14.46364785	14.55046545	0.709028044	0.8549403	0.941597499	3791	P35968	20751.8094	24523.288	19980.702	28666.52555	17356.74672	27763.89922
gp130	12.74892426	12.68532398	0.711154896	0.8549403	1.045070508	3572	P40189	5393.67357	7807.1782	7546.1754	6941.003672	6303.429501	6528.176875
TSH	10.69288871	10.82666011	0.703085752	0.8549403	0.911445692	7252	P01222	1651.4224	1231.0545	3023.5897	1556.30292	1823.400781	2107.409613
FAP	17.50077219	17.44212392	0.695547757	0.8549403	1.041489481	2191	Q12884	203678.762	175941.08	191702.74	196503.0363	170108.393	168929.3562
WIF-1	13.28658118	13.34477199	0.706229682	0.8549403	0.960467825	11197	Q9Y5W5	10114.3286	9457.4567	10395.156	11152.86329	10743.08435	9394.831817
Fetuin A	12.67237101	12.58648811	0.709667401	0.8549403	1.061337063	197	P02765	6305.92306	4713.6436	7231.6388	5936.706331	6385.155129	6134.828384
RANK	9.408500915	9.188678888	0.705552865	0.8549403	1.164589912	8792	Q9Y6Q6	827.643406	541.38625	1844.6643	551.0746715	659.2415702	544.1339252
TRANCE	8.906315076	8.506351296	0.704521338	0.8549403	1.319474784	8600	O14788	437.309765	514.15407	2726.5935	246.4808911	655.8714796	294.7866741
CD27	10.82591237	10.66517768	0.70931366	0.8549403	1.117856264	970	P32970	1959.72546	1815.6066	1664.7611	1078.63622	2895.453398	1368.213521
CXCL14	8.868104509	8.650712386	0.713496375	0.855418	1.162630067	9547	O95715	342.310695	376.03868	1393.7799	253.7374434	392.8747934	646.0245532
CXCL16	14.48282936	14.54724227	0.716085511	0.8561892	0.956334409	58191	Q9H2A7	21259.3354	21953.143	24139.402	29483.77919	21518.3631	21628.2541
LIMPII	11.29816748	11.58815833	0.721308035	0.8600963	0.817907243	950	Q14108	2575.9317	1350.694	3242.7331	2111.084093	11126.06012	1240.818482
ICAM-3	6.885455867	6.653137117	0.727779656	0.8654677	1.174721486	3385	P32942	94.3740928	93.551859	193.65622	102.8393233	90.03316025	106.8499738
Cathepsin B	12.10800718	11.94813471	0.733618079	0.8700592	1.117188384	1508	P07858	4007.56972	3887.535	6212.2416	5727.392263	1651.675259	6515.59214
IL-13 R2	10.02665862	10.10522096	0.737272707	0.872043	0.947000875	3598	Q14627	1039.55549	1196.6676	794.49335	1239.230723	810.2055975	1327.282459
CCL28	13.17681543	13.0977283	0.752099727	0.8814372	1.05634942	56477	Q9NRJ3	9530.55202	10694.866	7644.4502	11174.88658	7761.448588	7764.091127
IL-18 BPa	9.369512796	9.429072669	0.753228188	0.8814372	0.95955681	10068	O95998	655.84759	822.70188	557.36431	759.3737969	602.6992834	712.5974854
Siglec-7	11.04663407	11.1292699	0.752188243	0.8814372	0.944330764	27036	?Q9Y286	1696.64314	1951.8273	3240.9724	2298.060517	2156.893229	2264.436285
FGF-21	13.21192108	13.44495905	0.751491111	0.8814372	0.850841333	26291	Q9NSA1	25665.6763	2558.5739	8445.0468	12358.27798	10877.27422	10313.65708
FAS L	10.20598592	10.1198582	0.757016128	0.8835201	1.061517186	356	P48023	1791.67226	1049.2546	1183.1754	1124.968253	882.2155528	1384.318878
IL-28A	10.30898531	10.24946906	0.766041568	0.8878812	1.04211627	282616	Q8IZJ0	1362.21598	1339.7328	1323.6358	1519.15564	1152.338188	1027.840731
IL-10 Ra	7.100663025	7.192073731	0.766806468	0.8878812	0.938604507	3587	Q13651	107.67631	169.84747	181.97733	171.5502313	103.8378971	171.8446437
PSMA	3.686948297	4.258491687	0.764774348	0.8878812	0.672896541	2346	Q04609	16.2914512	88.804144	16.717413	82.72984526	0	82.73767797
DKK-1	9.52124725	9.650985535	0.774836246	0.894824	0.913997241	22943	O94907	966.420929	791.03915	1098.5369	1165.704211	524.2873868	846.911769
B7-1	10.19080609	10.39299573	0.791490146	0.9037587	0.869230291	941	P33681	756.138059	861.32659	1119.9533	641.5945839	919.3574779	4109.709398
Eotaxin	11.09465238	10.94398092	0.788849678	0.9037587	1.110086003	6356	P51671	1553.55588	897.38194	5766.0554	2041.139322	2050.266302	1824.123162
TIMP-2	16.88783654	16.82767763	0.794553078	0.9037587	1.042580595	7077	P16035	152893.206	136185.45	87790.807	126163.3653	122264.4463	102014.6327
EGF R	16.4571087	16.49498081	0.789813288	0.9037587	0.974090611	1956	P00533	87489.4458	83044.65	101229.42	88642.79501	94332.24779	94218.08408
Dkk-4	7.108698524	7.194584433	0.794896871	0.9037587	0.942205782	27121	Q9UBT3	101.057908	173.02813	104.89857	178.2752034	100.3964125	171.9077059
IL-1 F7	4.783359408	5.23274201	0.790842541	0.9037587	0.732356191	27178	Q9NZH6	0	80.916097	80.396717	86.79020576	6.526953531	79.45844074
TACI	10.34928941	10.45237618	0.806298905	0.9143596	0.931038818	23495	O14836	2407.95161	863.61205	1267.4454	1707.103915	821.265157	1957.439208
Epo R	1.597872002	2.136457552	0.829158882	0.9378661	0.688445545	2057	P19235	0	82.951722	0	0	0	83.99892307
CD14	15.36284669	15.32418719	0.843398307	0.9401436	1.027158986	929	P08571	34309.9076	44306.11	47994.971	47035.04825	32435.72003	45252.50799
Trappin-2	16.03772735	16.06968943	0.838202571	0.9401436	0.978089182	5266	P19957	62177.7526	65304.443	69083.327	75655.71797	67597.91596	63618.22736
Galectin-7	11.58785782	11.63487165	0.839915825	0.9401436	0.967937749	3963	P47929	3514.19008	2558.8407	2735.2587	4194.416384	2894.999884	2646.05202
NCAM-1	16.71123766	16.6669348	0.843763635	0.9401436	1.031184774	4684	P13591	128756.919	86690.689	120541.39	119439.6733	115479.8294	81672.24153
GASP-2	7.196227829	7.087775195	0.84056783	0.9401436	1.078071328	117166	Q96NZ8	111.355658	105.28041	427.66784	116.588606	191.6414985	110.1178216
Tie-1	9.969554106	9.88159274	0.84399252	0.9401436	1.062867211	7075	P35590	1152.20543	811.41831	1629.8374	493.5589051	1525.469875	1110.888389
FGF-9	5.678069043	5.512765547	0.851796299	0.9440564	1.121401959	2254	P31371	12.7276963	79.134925	72.357045	81.71475513	12.22320389	86.01691521
L1CAM-2	13.70825089	13.52065588	0.850395196	0.9440564	1.138863631	10752	O00533	22394.4128	14883.563	3757.0038	56892.91806	4307.667783	6620.556506
IL-2 Rg	9.45068928	9.535281584	0.855259193	0.9455127	0.943050998	3561	P31785	481.605656	614.89426	1389.7608	748.6549242	888.7801781	611.4536074
BMP-4	10.43271494	10.49334421	0.869060061	0.9460652	0.958845802	652	P12644	1934.62715	1668.7836	1108.915	864.104378	1851.052747	1868.442996
BMP-5	11.37087439	11.43390003	0.870897952	0.9460652	0.957254445	653	P22003	2338.68953	3987.2317	2741.2125	1961.602169	4703.587704	2292.436833
B2M	15.12644634	15.16331855	0.870053954	0.9460652	0.974765968	567	P61769	34759.5866	32458.48	44039.196	39087.78165	36154.98383	34962.01197
Thrombomodulin	12.90540777	12.72373886	0.868506665	0.9460652	1.134195163	7056	P07204	33147.9809	5174.5456	18312.12	12283.66214	5830.615633	4319.45072
CRTAM	11.00968545	10.87839505	0.872960193	0.9460652	1.095272916	56253	O95727	6646.79438	1348.1886	1805.1056	2578.494896	4092.715804	630.709316
IL-17B R	8.29524008	8.41138685	0.861695813	0.9460652	0.922648624	55540	Q9NRM6	327.524339	300.85701	930.59364	306.7102747	424.4369881	300.4824235
Granulysin	13.06431819	13.13895707	0.872690837	0.9460652	0.949579785	10578	P22749	6676.30828	5328.4434	19293.926	10815.67765	8549.469819	7934.543948
IL-15 R	10.6863508	10.74660469	0.867027218	0.9460652	0.959095324	3601	Q13261	3036.83602	1621.957	1547.2036	2114.407828	1427.331113	1677.52604
LDL R	10.298109	10.38708733	0.884463574	0.9561768	0.940188329	3949	P01130	3271.97207	668.16575	1106.0749	2795.707694	700.2011329	1223.573542
IL-27	10.11978543	10.03760177	0.886783741	0.9563354	1.058619146	246778	Q8NEV9	929.446816	761.58908	1281.0738	1656.791819	1281.088096	545.2730751
Angiogenin	16.65858671	16.64118168	0.890383112	0.9578694	1.012137316	283	P03950	105057.542	100256.15	106526.97	101828.7967	102904.5973	101898.1766
XEDAR	10.11109494	10.15682588	0.918660328	0.9664994	0.968798854	60401	Q9HAV5	670.9265	2344.1673	890.36473	880.7855472	1217.203027	1383.985094

NT-4	10.89894171	10.85102114	0.919864328	0.9664994	1.033773816	4909	P34130	830.666008	3495.7397	2597.6063	2616.948457	1559.351388	1541.641592
GCP-2	7.274522243	7.222095281	0.901797799	0.9664994	1.037007956	6372	P80162	213.732955	244.95018	92.933843	162.8103949	124.2636008	161.1942986
ICAM-2	14.23267487	14.21621709	0.9116365	0.9664994	1.011472983	3384	P13598	21595.4655	19017.361	20338.929	19728.43708	18413.70685	18976.70969
HGF R	17.05599993	17.04310516	0.921650383	0.9664994	1.008978036	4233	P08581	137440.399	136190.75	133655.57	146419.5861	131136.4006	128270.125
Pepsinogen I	17.14068355	17.12712449	0.924764204	0.9664994	1.009442732	643834	P0DJD8	150003.007	155175.37	139070.68	140986.2231	151363.6014	137444.5083
TSP-1	14.96621839	14.98646601	0.924013883	0.9664994	0.986063441	7057	P07996	37480.9275	36562.139	29837.852	31291.82552	35168.4509	31081.57786
Gas 1	6.623023557	6.601275981	0.9221608	0.9664994	1.015188461	2619	P54826	101.82157	90.839769	95.965605	100.4939225	96.9549279	91.06189557
IL-20	5.448089996	5.564515006	0.914990648	0.9664994	0.922470698	50604	Q9NYY1	5.09107851	77.989886	84.225132	89.32793108	12.104532	88.53940539
Mer	7.146058222	7.184398835	0.920385141	0.9664994	0.973774338	10461	Q12866	197.661123	181.17063	99.538792	184.3657441	89.71594308	181.9976666
MEPE	11.20392577	11.15574472	0.904457988	0.9664994	1.033960489	56955	Q9NQ76	3398.2926	1900.3047	2064.1256	1597.076307	1774.775923	4183.616617
Kallikrein 5	11.12683037	11.2083687	0.906906691	0.9664994	0.945049411	25818	Q9Y337	5058.43498	3125.5588	563.72692	3581.411864	1606.109402	2300.148193
NGF R	11.5767694	11.52410934	0.928606665	0.9682155	1.037175516	4804	P08138	3114.33249	3148.2747	4043.8504	1934.865255	1651.426883	7984.651943
I-309	7.977056436	7.955502506	0.935609936	0.9732113	1.015052207	6346	P22362	189.461014	223.83372	311.55704	263.0850021	209.2599346	274.4446821
IL-21R	8.095983624	8.125975107	0.945726414	0.979105	0.97942608	50615	Q9HBE5	183.708792	260.5463	368.65286	354.1816991	290.5975965	209.5000643
CD40	8.816344633	8.847066042	0.945489925	0.979105	0.978930668	958	P25942	969.157256	337.56832	484.15226	346.4448195	487.5960058	574.2557974
HCC-1	15.74656825	15.73801245	0.957114429	0.9843252	1.005948047	6358	Q16627	50442.5923	58905.093	54283.511	65186.317	47522.8266	52693.70169
EpCAM	10.81590316	10.79337598	0.959643483	0.9843252	1.015737193	4072	P16422	2852.74523	2326.0643	2011.6753	1690.037788	1381.792418	2389.448943
sFRP-3	9.535571793	9.512921558	0.959717095	0.9843252	1.015823838	2487	Q92765	853.551837	588.878	894.50445	704.917093	1547.937728	355.6453371
GITR L	11.06827411	11.04410122	0.955020845	0.9843252	1.016896529	8995	Q9UNG2	1342.44573	1675.8672	4843.9013	1968.200086	2608.235555	1831.373883
Dtk	13.10563783	13.08091398	0.965440016	0.9861528	1.017284956	7301	Q06418	19567.3915	7429.6913	12179.413	10623.40442	9847.475995	6215.827379
CTLA4	8.333691183	8.341489208	0.968222767	0.9861528	0.994609403	1493	P16410	270.590823	345.67458	344.55736	348.1759132	256.8059535	378.1212783
Galectin-2	12.37065068	12.39845782	0.96624402	0.9861528	0.980910124	3957	P05162	2255.03547	3431.7511	9726.7466	5958.168152	9623.138944	2742.946181
Nidogen-1	16.51631841	16.51009827	0.973677971	0.9894187	1.004320779	4811	P14543	107363.397	94420.597	100568.21	91552.72156	106824.1421	83128.45869
LIGHT	9.425303784	9.430931459	0.976463321	0.9899628	0.996106791	8740	O43557	688.143989	849.96163	567.27247	793.9217003	623.8758472	661.0056213
CD30	9.990186236	9.984803989	0.981169954	0.9923883	1.003737657	943	P28908	844.671037	1219.6714	1021.8644	841.6452078	1281.188858	961.8968164
NOV	15.42680314	15.43095444	0.98336656	0.9923883	0.997126671	4856	P48745	48275.3349	36548.34	47800.526	55988.38619	38078.69089	40432.4992
LYVE-1	15.4338311	15.43202639	0.991007611	0.9953597	1.001251709	10894	Q9Y5Y7	40275.2894	46319.786	41524.382	48330.77255	38672.92293	46221.93989
FOLR1	7.940002262	7.936289089	0.99309754	0.9953597	1.00257709	2348	P15328	367.172711	180.59238	415.84848	323.5817587	216.5931309	207.0721334
CD6	11.06568858	11.06963837	0.990215391	0.9953597	0.997265962	923	P30203	2611.17334	1555.8572	1866.6698	1576.875743	2893.293866	2173.023055
Thrombospondin-2	10.68141933	10.68255101	0.997438087	0.9974381	0.999215891	7058	P35442	2638.58297	2113.3067	993.51313	1854.140803	1427.571061	1674.043631

Table S3 human serum proteomics (cachexia tumor patients VS health)

proteinID	AveExp.H	AveExp.CA	P.Value	adj.P.Val	foldchange	entrezID	uniprotID	healthy1	healthy2	healthy3	Cachexia1	Cachexia2	Cachexia3
MMP-8	11.447152	13.98572821	3.80E-05	0.0167235	0.17211245	4317	P22894	3575.445	1673.682	3633.329	12674.4471	14095.58	21758.251
Pentraxin 3	12.251072	14.29749089	8.97E-05	0.019728	0.24208421	5806	P26022	6407.319	3803.219	4750.28	13675.7639	21042.08	21764.035
IL-18	11.259644	15.03502206	0.000245202	0.035963	0.07302942	3606	Q14116	2661.096	2985.196	1853.084	50455.0063	15704.466	91400.22
Lipocalin-2	8.1881088	15.31176337	0.000682086	0.036293	1.87123554	3934	P80188	29864.82	38256.68	36525.57	64134.3917	61388.23	71543.11
Flt-3L	12.079889	10.15612848	0.000694191	0.0436348	3.79410636	2322	P36888	3202.142	4688.178	5400.987	1139.70461	728.9301	1938.4534
BLC	9.7555527	13.89234015	0.000636355	0.0436348	0.05684639	10563	O43927	831.644	946.1068	817.9995	38878.2307	9962.2958	3491.2904
GDF-15	15.586497	16.89084564	0.000607755	0.0436348	0.40490392	9518	Q99988	34434.73	55886.1	61898.71	129361.845	121605.55	137109.82
MIF	11.156653	13.13562342	0.000400471	0.0436348	0.25367079	4283	Q07325	2228.116	2709.079	1968.459	6677.85723	6299.5218	9483.1914
uPAR	11.713253	13.72706431	0.000848076	0.0466442	0.24761812	5329	Q03405	4690.896	2727.877	2955.578	19982.4543	6501.1672	18124.508
TNF RI	14.218683	15.26443842	0.001351632	0.0660798	0.48439112	7132	P19438	22957.84	18771.82	16078.43	51683.8896	33454.153	37658.412
Syndecan-1	13.105013	14.47821993	0.001720107	0.0756847	0.38603207	6382	P18827	8225.019	7213.098	11523.81	24517.7419	18352.147	23785.978
Follistatin	12.06037	12.88956997	0.002424575	0.096983	0.56284122	10468	P19883	4195.826	3933.592	4717.224	8161.71704	8678.1773	8257.1638
CA125	12.216685	16.59195065	0.006339037	0.2324314	0.0481852	94025	Q8WXI7	5116.145	4711.023	4471.294	70034.3265	9642.72	596038.35
MIG	7.5635896	8.934310314	0.008330945	0.2443744	0.38669801	4283	Q07325	233.1975	220.3632	129.5914	606.73943	633.18267	653.71687
HGF	10.884122	12.46029132	0.007862777	0.2443744	0.33537129	3082	P14210	1925.968	2012.536	1738.835	2686.88882	4236.5783	12189.073
AgRP	10.436458	11.44612125	0.007253539	0.2443744	0.49666221	181	O00253	1365.403	1411.988	1377.299	4755.23874	1894.4504	2704.9048
TRAIL R3	13.227357	13.85005404	0.01101442	0.3018331	0.64945576	8794	O14798	8763.721	9299.256	10819.84	18048.5871	11472.655	13828.643
FSH	15.131215	11.95727089	0.011661733	0.3018331	9.02510475	2488	P01225	37812.35	32481.29	37630.93	15198.0003	1612.9444	12665.562
IGFBP-1	13.65296	15.28973734	0.012636503	0.3088923	0.321574	3484	P08833	5775.989	12322.51	30019.34	46429.6516	28849.883	46165.166
CEACAM-1	13.825494	14.82871394	0.014954276	0.3144303	0.49888532	634	P13688	22138.47	12011.77	11503.16	32405.4751	21806.701	32942.487
HVEM	12.182294	12.95357896	0.022227804	0.3144303	0.58589529	8764	Q92956	5220.952	3606.988	5327.557	11704.7927	5831.191	7913.9329
RAGE	14.743397	13.98433187	0.02339352	0.3144303	1.69239384	177	Q15109	32113.63	35515.9	18090.57	21121.0136	17258.132	13724.88
OPG	11.271295	12.39715924	0.014635648	0.3144303	0.45822745	4982	O00300	2937.909	2085.145	2461.992	7400.10987	4093.3229	5258.9494
MIP-3b	11.964058	10.34170119	0.021116372	0.3144303	3.07877556	6363	Q99731	5393.357	4467.385	2644.639	953.20515	807.45709	3899.1626
BMP-9	11.685904	10.32583363	0.016670912	0.3144303	2.56697661	2658	Q9UK05	2694.033	3601.797	3682.144	1939.80235	1805.3697	572.99218
IL-24	8.4499662	7.198637649	0.018176941	0.3144303	2.38060548	11009	Q13007	416.0119	262.7747	387.7749	271.501998	209.00654	87.907774
DLL1	7.9991209	9.1384091	0.02429689	0.3144303	0.45398351	28514	O00548	313.3099	256.2809	206.0906	771.115034	369.52381	1061.2699
HAI-2	9.4282743	11.50266829	0.022219574	0.3144303	0.23743524	6691	P20155	754.4573	755.4386	571.2641	8596.36894	965.11383	5326.3099
CHI3L1	13.125348	13.68759394	0.019779924	0.3144303	0.67724685	1116	P36222	6554.481	10581.79	10283.08	12734.6965	13052.174	12443.37
P-Cadherin	12.415273	13.98612849	0.015216959	0.3144303	0.33660882	1001	P22223	6160.512	6923.5	3818.736	41897.5397	10706.37	11058.377
Follistatin-like 1	12.171917	11.58958398	0.023283053	0.3144303	1.49726884	11167	Q12841	4433.844	3865.454	5728.879	3123.32027	3341.3042	2755.0937
Renin	12.784578	15.30248134	0.020057358	0.3144303	0.17459651	5972	P00797	19188.27	6165.554	2967.407	16657.7915	38141.938	25470.845
BMPR-IB	7.9137425	2.113807939	0.023633619	0.3144303	55.7127099	658	O00238	804.0383	44.59347	381.0344	349.976774	0	0
Cystatin B	14.320219	14.74599047	0.021318656	0.3144303	0.74444031	1476	P04080	19948.18	19930.96	21525.38	29721.1045	23059.429	30779.04
Galectin-9	13.309701	14.36317622	0.022149492	0.3144303	0.4818063	3965	O00182	10737.51	12415.33	7849.922	26912.9875	15077.898	17355.208
IL-1ra	12.02864	13.18148762	0.027579259	0.3444461	0.44973658	3557	P18510	4503.935	3447.013	4694.552	4400.35726	9272.9468	19822.254
SDF-1b	9.23952	8.220550518	0.028181951	0.3444461	2.02647088	6387	P48061	595.9319	611.8136	602.7606	266.916257	441.26737	457.57678
TRAIL R4	10.21797	9.847399406	0.030543649	0.344595	1.29286426	8793	Q9UBN6	1245.66	1203.207	1124.375	868.65943	1019.2621	898.27625
Prolactin	11.388212	13.33731596	0.029764822	0.344595	0.25897706	5617	P01236	3927.965	4054.491	1207.534	9543.25022	14738.428	9046.7744
Aggrecan	12.842572	12.1059895	0.030024749	0.344595	1.6662239	176	P16112	5890.114	8639.768	7783.813	6517.73297	3177.0602	5227.1081
Prostasin	5.579057	7.368146125	0.032522672	0.3577494	0.28935469	5652	Q16651	82.04764	85.42413	14.22048	197.279292	86.895745	386.4147
LOX-1	11.09723	12.68297588	0.035931894	0.3764294	0.33315241	4973	P78380	3180.864	1347.034	2450.404	2319.22609	4562.2598	15293.137
CD23	11.383536	9.604238927	0.035248802	0.3764294	3.43258882	2208	P06734	2118.777	3071.877	2926.67	716.823854	463.61361	351.83135
I-309	7.3436833	7.977056436	0.038795646	0.3863376	0.64466735	6346	P22362	173.6605	118.6674	204.0388	189.461014	223.83372	311.55704
TIMP-1	16.754062	16.40675378	0.041267875	0.3863376	1.27218478	7076	P01033	105289.5	109951	116635.2	91132.3357	96316.948	81237.565
IP-10	11.015484	11.82388221	0.040717246	0.3863376	0.57101553	3627	P02778	1805.221	2955.854	1660.011	3799.32532	3276.5963	4697.3074
Neprilysin	11.271943	9.704433269	0.039993973	0.3863376	2.96392694	4311	P08473	2365.75	2538.855	2514.461	1236.67402	435.59861	894.38202
Pref-1	9.3786022	3.978826172	0.04069006	0.3863376	42.2176996	8788	P80370	438.9294	407.4961	1640.16	733.416461	83.147593	0
IL-10	10.701857	11.19145214	0.044184635	0.3878419	0.71222493	3586	P22301	1612.849	1699.867	1682.475	1647.85885	2313.6948	2950.8553
IL-16	11.607903	14.12519718	0.044954404	0.3878419	0.17467022	3603	Q14005	1814.605	4244.28	3944.053	10698.9893	4481.0244	163881.06
hCGb	12.524542	11.57167683	0.044655765	0.3878419	1.93571358	1082	P01233	4692.557	4641.697	9385.775	3129.73095	2566.7085	3796.3483
Midkine	11.912496	12.68325315	0.043733308	0.3878419	0.58610967	4282	P14174	4790.799	3802.987	3141.809	6600.49655	5651.4858	8785.146
IL-5	10.558418	11.21230848	0.047423528	0.3882864	0.63556429	3567	P05113	1640.41	1450.006	1438.845	1552.09003	1922.8334	3308.3166
DAN	9.409698	10.22779978	0.047653329	0.3882864	0.56718772	4681	P41271	726.5609	744.5434	579.045	1651.4977	615.15148	1545.408
SIGIRR	6.3216762	2.496790782	0.046337998	0.3882864	14.171155	59307	Q6IA17	73.70875	90.95157	73.49238	11.2003727	82.188362	0
BMP-5	12.226473	11.37087439	0.050983515	0.3946115	1.80950887	653	P22003	6588.032	3866.059	4318.192	2338.68953	3987.2317	2741.2125
b-NGF	11.095394	10.3764451	0.057042276	0.3946115	1.6459823	4803	P01138	1564.116	1898.215	3522.859	1153.24387	1910.0288	1307.1339
VEGF R3	10.583358	11.40403815	0.057212139	0.3946115	0.56617515	2324	P35916	1259.72	1072.256	2668.335	3491.93341	3378.975	2455.3885
GRO	11.71111	12.36534626	0.050431753	0.3946115	0.63541162	2919	P09341	3689.372	3597.314	2837.028	4168.31168	4199.4228	7093.1741
MDC	9.829288	8.377924516	0.055783909	0.3946115	2.73466387	6367	O00626	1170.747	610.5806	1049.616	98.9653943	340.29471	734.51758
MPIF-1	14.117942	14.96836611	0.05694713	0.3946115	0.55462148	6368	P55773	18493.06	13780.49	22050.76	49640.2575	26226.077	18875.896
TSLP	10.432021	8.215403435	0.053674232	0.3946115	4.64802253	85480	Q969D9	1878.475	1325.995	1056.176	141.873467	101.5458	1675.1967
G-CSF R	9.4483939	10.13234175	0.057398031	0.3946115	0.6224596	1441	Q99062	735.9227	630.3341	731.9404	1158.43906	1708.2007	1289.5162
B7-H3	14.007442	14.52852925	0.052876614	0.3946115	0.69684644	80381	Q5ZPR3	16502.47	16175.47	16729.99	21609.9965	24562.972	22251.249
FGF-21	11.630312	13.21192108	0.055527467	0.3946115	0.33410902	26291	Q9NSA1	5555.588	2608.718	2195.931	25665.6763	2558.5739	8445.0468
PF4	15.933813	15.03623658	0.059285748	0.3997104	1.86293365	5196	P02776	58880.15	62630.7	66510.44	65009.191	46002.187	18975.575
Cathepsin S	11.938174	12.91367213	0.060864989	0.3997104	0.50856419	1520	P25774	4485.438	4505.221	2988.037	14464.0999	6419.0195	7086.2686
Cadherin-13	9.2740848	4.316733197	0.060194732	0.3997104	31.0678738	1012	P55290	448.3994	658.6827	799.5078	423.12972	370.84847	0
ErbB3	13.447956	14.5242933	0.064753386	0.4189925	0.47423128	2065	P21860	11853.81	11025.51	10674.34	52461.8023	9266.5052	28448.174
Angiostatin	11.662653	12.19751265	0.072370148	0.4588165	0.69022585	5340	P00747	2882.953	3660.803	3225.577	3927.62421	5739.7543	3335.7259
Procalcitonin	10.575133	11.24976459	0.074968846	0.4588165	0.62649239	796	P06881	1897.755	1275.36	1464.059	3786.72595	2372.8813	2533.1902
TIMP-4	12.917335	13.9480065	0.075079056	0.4588165	0.48948233	7079	Q99727	9484.486	5123.21	9523.223	8879.34463	17305.617	18518.32
ADAM8	8.8745457	4.216889819	0.074299515	0.4588165	25.2402777	101	P78325	253.5225	361.8574	1118.57	626.674803	189.5051	0
IFNg	10.75417	11.69554221	0.078595491	0.461575	0.52073741	3458	P01579	2222.801	1473.173	1570.588	1516.7875	2466.8408	6276.402
IL-1a	11.380745	12.57667633	0.090307949	0.461575	0.43650457	3552	P01583	2821.114	3210.756	2090.798	2695.57459	3880.211	13899.327
IL-6	10.828226	11.55207872	0.092314996	0.461575	0.60547817	3569	P05231	1988.132	1784.053	1691.573	1839.76295	1954.9467	5102.309
IL-8	11.226441	12.38384157	0.086048945	0.461575	0.44831966	3576	P10145	2419.474	2383.047	2382.809	3277.39459	2594.7823	18392.345
MCSF	8.3915569	9.207750677	0.084366069	0.461575	0.56793833	1435	P09603	393.8803	268.7191	354.5935	527.827879	295.73686	916.54089
MIP-1b	12.681151	13.65505762	0.087371136	0.461575	0.50912567	6351	P13236	7239.691	7466.62	5238.123	4713.56038	13943.325	18988.649
TARC	9.5468679	10.52980356	0.089680774	0.461575	0.50594915	6361	Q92583	879.8424	853.723	554.8494	726.438291	1544.6331	3860.1268
ANG-1	13.036071	14.47660506	0.084657853	0.461575	0.36843077	284	Q15389	8628.481	11935.47	5751.865	9887.21994	10129.6	51514.732
CEA	15.44845	15.79022816	0.078209024	0.461575	0.7890682	1048	P06731	41153.52	42861.14	50680.47	64030.3471	47778.03	64904.845
MMP-3	12.121605	13.32473957	0.07892954	0.461575	0.4343305	4314	P08254	5954.99	4593.655	3232.65	15630.9245	10525.03	13878.785
ANG-2	12.65839	13.83988483	0.083793539	0.461575	0.44089436	285	O15123	3445.067	6556.55	11955.38	33581.9718	7397.4531	13027.369
IL-33	10.162154	8.761153382	0.087271268	0.461575	2.64084592	90865	O95760	2339.431	950.5034	674.5116	235.487664	391.2783	623.06777
CA19-9	10.849545	13.93171815	0.080906138	0.461575	0.11807925	30815	Q969X2	1330.044	2831.463	1665.324	30381.6445	2276.0373	154706.97

Clusterin	10.265805	11.55735107	0.0920432	0.461575	0.40851297	1191	P10909	1214.139	1403.817	1092.201	1028.6986	1766.0528	8784.3167
Siglec-7	11.533482	11.04663407	0.08903669	0.461575	1.40137947	27036	?Q9Y286	3697.038	2898.512	2428.269	1696.64314	1951.8273	3240.9724
JAM-B	8.6197313	4.209762591	0.08335858	0.461575	21.2585115	58494	P57087	353.1858	334.9155	510.6014	413.128586	282.08969	0
GM-CSF	10.349346	11.27616108	0.093809316	0.4637764	0.52601847	1437	P04141	1545.017	1064.774	1346.447	1024.99508	2014.5675	4362.3129
IL-1 RII	15.209581	15.53440767	0.097401939	0.4701717	0.7983944	7850	P27930	37752.92	36360.4	39628.42	48146.3134	46363.956	40450.11
B7-H1	11.775256	11.19590243	0.098308621	0.4701717	1.49417977	29126	Q9NZQ7	3833.611	4481.983	2504.114	2830.20001	2413.2689	2148.6041
DNAM-1	11.475362	11.00414241	0.097169784	0.4701717	1.38628048	10666	Q15762	4202.715	2617.746	2095.803	2237.70159	1901.5614	2085.3725
NT-4	11.753729	10.89894171	0.099816394	0.4718048	1.8084915	4909	P34130	3417.666	3985.456	3020.557	830.666008	3495.7397	2597.6063
CD163	14.160583	14.91966722	0.100794662	0.4718048	0.59087109	9332	Q86VB7	19262.34	26448.26	12053.11	42863.2921	43259.042	21079.948
IL-17F	10.534183	9.029556223	0.103986452	0.4816215	2.83751319	112744	Q96PD4	2161.329	1305.755	1152.983	252.950011	320.59276	1202.589
IL-7	10.918638	11.43422689	0.106091463	0.4862525	0.69950735	3574	P13232	2089.947	1875.781	1847.23	1790.53484	2251.3872	3890.1554
IL-28A	10.655082	10.30898531	0.109875405	0.4953485	1.27111722	282616	Q8IZJ0	1725.104	1368.941	1772.085	1362.21598	1339.7328	1323.6358
OPN	13.097404	14.37722016	0.110327628	0.4953485	0.41184798	6696	P10451	9488.201	7340.461	9662.227	80499.6975	10154.968	16005.273
IL-12p40	9.2341499	8.593942595	0.123330628	0.529906	1.55855313	3593	P29460	478.8375	723.5885	627.178	337.389633	301.5582	689.59158
LAP(TGFb1)	14.14214	15.14955079	0.123220493	0.529906	0.49743826	7040	P01137	24400.93	18736.1	12926.02	24050.8214	35513.402	42246.126
Siglec-5	13.950804	17.60743492	0.120146675	0.529906	0.07929475	8778	O15389	1922.222	8399.02	245764.9	154598.804	227746.42	223603.02
TGFb2	11.620511	11.29494549	0.125250517	0.529906	1.25315549	7042	P61812	3188.387	2828.82	3457.605	2650.94112	2475.3594	2772.7919
IGF-2R	10.676651	11.24584977	0.124681339	0.529906	0.67399103	3482	P11717	1189.677	1783.413	2062.894	4069.25594	1928.7061	1799.6607
Cadherin-11	10.562191	10.06219782	0.125153828	0.529906	1.41420696	1009	P55287	1944.158	1541.838	1150.685	1254.83506	1241.1923	1083.0807
ErbB2	7.3450038	7.95321307	0.126627518	0.5306296	0.65601045	2064	P04626	250.9428	139.8688	120.0829	206.759369	191.90568	314.78433
IL-2	10.800414	11.19604793	0.130715479	0.5336177	0.76015539	3558	P60568	1817.147	1737.852	1793.132	1664.96043	2018.7895	3318.346
IL-12p70	9.4133698	9.965662188	0.135857192	0.5336177	0.68193567	3592	P29459 & P2	688.8891	583.3697	785.4172	750.270501	662.48128	1771.8762
TNFb	11.301558	11.69046712	0.137919002	0.5336177	0.76370681	4050	Q06643	2664.177	2633.043	2289.735	2330.70029	2912.2053	4601.0918
PDGF-AA	13.505287	14.36274462	0.131649799	0.5336177	0.55192417	5154	P04085	15506.74	10274	9865.406	11372.9699	15363.817	36565.897
IL-31	10.125389	8.805925201	0.133493899	0.5336177	2.49573361	386653	Q6EBC2	2329.946	448.9182	1327.837	279.882901	354.37677	1236.2529
MIP-3a	8.3664709	10.0085189	0.134763485	0.5336177	0.32040132	6364	P78556	278.8939	344.2139	371.0441	322.387269	9039.1268	720.07314
Albumin	15.809917	16.20640947	0.138255491	0.5336177	0.75970323	213	P02768	56240.01	44804.02	75230.25	88585.1926	59022.046	80958.752
GASP-1	15.840375	16.29222027	0.137536928	0.5336177	0.73110726	124857	Q8TEU8	64805.38	51470.41	60547.09	109458.294	69781.756	101816.3
Ck beta 8-1	10.37006	9.744517478	0.136255502	0.5336177	1.54279042	6368	P55773	2271.5	1003.82	1014.09	1225.92354	1099.0653	728.62719
CTACK	12.983558	13.48031202	0.144114425	0.5419688	0.70869965	10850	Q9Y4X3	7201.218	7129.755	10343.74	9811.41535	9850.9765	12105.633
NAP-2	16.313012	16.03155961	0.142084421	0.5419688	1.21541764	5473	P02775	89407.88	82194.85	73431.29	74201.2076	70254.709	60532.342
SP-D	10.246931	5.510696927	0.143933872	0.5419688	26.6531565	66421	P35247	746.8759	1600.023	1497.56	1238.88256	3483.6546	0
Shh-N	9.4975215	9.164688605	0.150220112	0.5554873	1.25948407	6469	Q15465	570.9959	685.7705	960.4174	513.185683	684.85084	520.17399
TRAIL R2	13.155397	13.85642162	0.150234063	0.5554873	0.61513534	8795	O14763	12029.04	6060.235	10414.48	26753.753	10892.483	19661.384
IL-13	10.303232	10.67956981	0.153681279	0.5572712	0.77039063	3596	P35225	1316.502	1218.867	1254.11	1169.992	1371.4053	2269.6217
IL-34	5.8022627	7.380805026	0.154516108	0.5572712	0.33482002	146433	Q6ZMJ4	123.1589	90.95157	14.22048	91.7666901	178.75333	148.79773
CD58	9.4731088	5.161753203	0.154382006	0.5572712	19.8539691	965	P19256	432.9393	643.5279	1282.509	2236.35411	732.89757	0
Tie-2	9.7575191	8.632963675	0.159056678	0.5643947	2.18034343	7010	Q02763	1430.338	499.6347	904.0128	879.729116	565.2448	504.80808
TFPI	11.638204	12.69220231	0.158322706	0.5643947	0.4816316	7035	P10646	1323.442	7373.26	3314.846	3654.5854	6759.0543	10640.439
GDNF	10.378201	10.81897178	0.164506968	0.5707118	0.73674082	2668	P39905	1640.365	1379.246	1039.609	1541.34537	1577.9952	3027.6315
Insulin	12.262415	11.21318337	0.172132744	0.5707118	2.06942668	3630	P01308	6323.137	2847.432	6582.496	852.087194	3489.6957	3259.8346
gp130	12.997565	12.74892426	0.170792504	0.5707118	1.18808694	3572	P40189	7881.034	8304.84	8354.042	5393.67357	7807.1782	7546.1754
IL-21	10.429737	10.73763574	0.173598404	0.5707118	0.8078177	59067	Q9HBE4	1670.338	1210.655	1294.83	1477.4709	1399.9027	1832.8048
Leptin	14.011282	11.99215575	0.162711747	0.5707118	4.05338233	3952	P41159	31458.65	22408.43	6385.513	383.782246	4309.4714	4639.2751
DcR3	10.745336	10.46726015	0.176315443	0.5707118	1.2125769	8771	O95407	1849.041	1702.463	1604.062	1584.50943	1478.6113	1362.4481
ANG-4	7.5346986	8.518298528	0.167244735	0.5707118	0.50571626	51378	Q9Y264	207.9423	147.1009	205.0283	237.36682	286.26368	1467.7715
BAFF	7.0887882	8.205016777	0.176401824	0.5707118	0.46129814	10673	Q9Y275	143.4522	147.33	116.7227	243.516898	114.40123	1332.9343
TWEAK	8.5697607	8.113986714	0.170402391	0.5707118	1.37151844	8742	O43508	383.822	465.3102	304.7245	252.899325	481.04365	209.22288
LRP-6	11.549469	10.60449617	0.175086479	0.5707118	1.92515199	4040	O75581	4738.36	1868.735	3037.852	833.803231	970.25875	4276.9792
Nectin-4	12.232441	11.92554023	0.16882924	0.5707118	1.23704729	81607	Q96NY8	4785.414	6038.342	3853.739	4697.80911	4190.1707	3220.1072
ICOS	8.5441774	4.725545659	0.172429392	0.5707118	14.1098596	29851	Q9Y6W8	599.5773	231.7931	371.0677	722.094423	676.53065	0
ICAM-3	5.9513728	6.885455867	0.184100066	0.5779369	0.523375	3385	P32942	116.2576	115.3078	16.37302	94.3740928	93.551859	193.65622
TSP-1	15.263529	14.96621839	0.185202498	0.5779369	1.22885154	7057	P07996	34619.85	50137.57	35060.5	37480.9275	36562.139	29837.852
PSMA	6.3821794	3.686948297	0.181278292	0.5779369	6.47657494	2346	Q04609	78.37387	89.19284	80.06487	16.2914512	88.804144	16.717413
Periostin	13.384765	12.91082949	0.181952512	0.5779369	1.38889352	10631	Q15063	13808.8	8630.388	10264.49	7006.47533	5366.2685	10897.219
JAM-A	7.6445143	4.311653114	0.184714765	0.5779369	10.0760705	50848	Q9Y624	109.3045	170.361	422.8178	425.834429	363.32438	0
IL-17B	11.32058	11.81454651	0.188308354	0.5794103	0.71007015	27190	Q9UHF5	2589.544	2448.171	2635.892	2951.62614	2357.514	5535.5077
Leptin R	6.3712385	4.087592819	0.187606	0.5794103	4.86906807	3953	P48357	78.14061	87.68535	79.82587	0	88.549691	9.6986516
IGFBP-3	14.242455	13.77668469	0.194975809	0.5916507	1.38105413	3486	P17936	14256.83	22910.77	22288.78	13332.3784	22395.418	8449.2288
TLR4	10.266621	7.092606209	0.194042377	0.5916507	9.02554756	7099	O00206	3575.671	849.8308	613.2711	652.275189	617.74071	857.50734
MMP-1	8.4466739	10.2040287	0.199756422	0.6020057	0.29579	4312	P03956	685.6816	144.3689	424.4806	1992.34501	152.99614	2112.3105
TF	7.8977007	7.339865694	0.20171219	0.6037644	1.47205854	2152	P13726	304.9816	262.1767	167.4192	158.077988	184.22407	202.77839
PD-1	10.191389	8.860830458	0.203635273	0.6054022	2.51500035	5133	Q15116	2801.596	709.6033	801.7007	235.002623	283.74509	840.87826
PDGF-AB	10.612024	11.85446752	0.20707972	0.6074338	0.42265612	767790	Q08CD2	1936.842	1167.603	1691.883	1025.25196	2353.0212	10967.736
CD48	5.272374	1.899169963	0.205801902	0.6074338	10.3618095	962	P09326	386.5591	0	147.9671	0	192.56575	0
C5a	12.829331	13.55518331	0.211033525	0.6149321	0.60463971	727	P01031	13918.6	2729.04	10143.83	19002.3509	10414.324	12024.877
4-1BB	11.065716	9.881485444	0.221644222	0.6251504	2.27242122	3604	Q07011	3931.346	1944.799	1286.026	319.199753	712.56789	1785.8356
PECAM-1	13.12035	13.81056993	0.220748774	0.6251504	0.6197594	5175	P16284	6002.439	16994.63	6919.212	7857.67401	18534.759	14314.044
XEDAR	10.691711	10.11109494	0.216573298	0.6251504	1.49548789	60401	Q9HAV5	2085.018	1111.373	1948.885	670.9265	2344.1673	890.36473
MCSF R	15.536883	15.75714068	0.219142916	0.6251504	0.85841185	1436	P07333	40887.62	51407.39	51116.15	61683.4965	53493.408	56939.202
MMP-10	10.766834	9.99018966	0.219165749	0.6251504	1.71314096	4319	P09238	1411.553	1517.637	2464.817	2040.09093	1346.7023	1247.983
I-TAC	8.9459242	9.267522896	0.223705236	0.6269446	0.80018265	6373	O14625	454.1761	446.3233	588.0791	516.742385	616.25125	915.84899
IGF-2	11.802308	12.42637839	0.229561925	0.6384893	0.64883788	3481	P01344	2353.671	3461.214	5587.075	11954.3336	3419.0808	5038.4133
ULBP-1	8.6896185	5.962094845	0.230726818	0.6384893	6.62317793	80329	Q9BZM6	668.948	253.7415	411.4456	122.592511	85.443079	0
TNFa	11.189582	11.53445067	0.233778951	0.6415522	0.78737968	4049	P01374	2776.026	2245.253	2041.495	1968.51337	2586.5941	4114.4045
PIGF	9.8295473	10.50716307	0.236945665	0.6415522	0.62519764	5228	P49763	1342.193	870.76	642.3251	980.614313	1847.1456	2437.8675
Resistin	10.816118	11.85547436	0.239124007	0.6415522	0.48654447	56729	Q9HD89	1914.754	1233.968	2476.036	5389.81783	15581.085	2340.4051
S100A8	10.536519	11.24343929	0.237834452	0.6415522	0.61262663	6279	P05109	2640.923	1275.592	970.5222	1377.61516	2659.0694	6150.0104
ULBP-2	12.45937	12.04091145	0.235690876	0.6415522	1.3364989	80328	Q9BZM5	3637.614	6405.715	7661.332	6556.28166	3664.7657	3222.1428
IL-4	10.220421	10.46421048	0.249925393	0.6618301	0.84452401	3565	P05112	1165.586	1162.16	1250.422	1270.89129	1180.2607	1980.5667
IL-11	10.20769	10.51203841	0.252133389	0.6618301	0.80980813	3589	P20809	1622.883	962.5398	1055.912	1524.97182	1186.6578	1982.2383
Furin	10.528726	10.88669366	0.251200072	0.6618301	0.78026318	5045	P09958	1443.155	1184.368	1882.303	1939.2372	1290.4668	2778.2731
IL-1 R3	13.114584	12.79210292	0.252698773	0.6618301	1.25047936	3556	Q9NPH3	10029.77	9212.609	7547.933	5615.3431	6065.8451	9532.4589
EGF	13.911096	15.03953989	0.26401712	0.6873819	0.45740883	1950	P01133	20850.49	18430.29	9511.147	8123.10991	25257.825	102416.62
TRAIL	9.7199346	9.300125608	0.265658836	0.6875876	1.33775045	8743	P50591	609.9601	1245.598	786.4744	403.190353	512.65847	811.57403
TIM-1	11.153026	12.34409935	0.277293046	0.6929649	0.43797695	26762	Q96D42	3359.648	1359	2582.595	34580.4314	1661.7008	3833.788
VCAM-1	16.5216	16.73192506	0.290865231	0.6929649	0.86434249	7412	P19320	104323.3	85896.57	92922.46	121418.455	98746.422	124839.93
MIP-1a	11.199664	11.60614308	0.290246546	0.6929649	0.75446242	6348	P10147	2968.016	1659.593	2637.935	4185.7325	2541.0469	3127.1429

EG-VEGF	11.069558	10.30532315	0.292407876	0.6929649	1.69846938	84432	P58294	6206.19	2030.633	786.1672	669.517082	2212.8068	1128.6179
SCF	10.660143	11.19239595	0.270932155	0.6929649	0.69147426	4254	P21583	1832.722	1978.328	1166.394	1949.32797	5060.4896	2310.6583
TGFa	11.080511	11.75709841	0.283803937	0.6929649	0.62564335	7039	P01135	3544.368	1360.156	2103.405	1625.91005	2228.4312	6565.0254
CA15-3	12.739261	13.05202276	0.292935155	0.6929649	0.80509934	4582	P15941	5282.481	8093.779	7473.366	6439.55615	8512.6622	11447.41
CRP	14.385992	14.61935507	0.29182981	0.6929649	0.85064954	1401	P02741	19117.23	17922.9	28638.46	28337.9003	22983.047	27094.539
ADAM9	11.577521	12.15793511	0.279635477	0.6929649	0.66877163	8754	Q13443	2478.78	2884.392	3988.56	9833.00447	2865.0768	4901.7767
LDL R	9.6173548	10.298109	0.283372793	0.6929649	0.62383907	3949	P01130	798.7624	825.323	732.206	3271.97207	668.16575	1106.0749
CA9	6.8640845	6.607650847	0.292432531	0.6929649	1.19452221	768	Q16790	96.62611	133.9149	119.0218	95.7122759	90.585316	105.40903
IL-1 F5	7.0415042	5.137249772	0.279035346	0.6929649	3.74315394	26525	Q9UBH0	301.7161	91.07719	81.02087	0	85.496253	174.3205
ADAM12	9.1682117	6.775228566	0.290187121	0.6929649	5.25242319	8038	O43184	279.1372	689.8052	982.9972	798.203679	627.94287	285.36467
Cadherin-4	10.399961	7.61007822	0.288135674	0.6929649	6.91573814	1002	P55283	2825.487	587.2635	1482.482	2944.04438	526.17633	937.42486
Cystatin A	10.787671	7.907538028	0.276834676	0.6929649	7.36217813	1475	P01040	3296.762	1007.069	1660.605	3768.79195	925.20826	950.91576
Kallikrein 5	11.917337	11.12683037	0.274723911	0.6929649	1.72968151	25818	Q9Y337	3815.959	3493.852	4336.918	5058.43498	3125.5588	563.72692
LIF	10.005603	10.22111512	0.295896187	0.6962263	0.86124051	3976	P15018	1049.449	853.3987	1209.388	1195.88952	1051.7613	1164.3888
Eotaxin-2	12.161429	12.74532544	0.297532727	0.6963532	0.66715966	6369	O00175	6946.02	3710.075	3727.781	4760.83401	5158.7307	13430.77
MMP-9	13.751108	14.32259657	0.306724492	0.7065905	0.67292193	4318	P14780	16836.45	10827.06	14375.75	7817.7658	29596.347	37547.921
MEPE	11.629841	11.20392577	0.304513528	0.7065905	1.34342457	56955	Q9NQ76	2239.875	3455.291	4108.274	3398.2926	1900.3047	2064.1256
CEACAM-5	0	2.896658623	0.305811305	0.7065905	0.13428233	1048	P06731	0	0	0	3074.56232	0	0
Axl	13.692176	13.94076135	0.309639172	0.7072182	0.84172125	558	P30530	13077.13	12082.52	14672.3	21965.9345	14264.602	12471.399
TPO	10.496963	10.71354531	0.311818915	0.7072182	0.86060198	7066	P40225	1127.815	1792.114	1489.98	1436.57165	1652.8705	1963.5624
GASP-2	6.6323094	7.196227829	0.31063017	0.7072182	0.67646234	117166	Q96NZ8	83.27847	99.67116	114.0669	111.355658	105.28041	427.66784
GITR	9.4973745	9.869034382	0.31937792	0.7097287	0.77289276	8784	Q9Y5U5	782.013	696.7857	690.0235	1880.72449	785.76067	815.22209
APRIL	9.0405584	8.695303302	0.317854976	0.7097287	1.2703756	8741	O75888	609.6507	500.5399	475.8034	500.07782	450.39186	311.5951
LAG-3	9.0460585	8.848327822	0.317279154	0.7097287	1.14689288	3902	P18627	545.8802	512.5618	524.9203	559.787903	490.70051	467.64863
Epo R	4.1705152	1.597872002	0.317238133	0.7097287	5.94898375	2057	P19235	69.86004	81.40417	0	0	82.951722	0
Persephin	12.196988	11.94154325	0.321598934	0.711073	1.19370378	5623	O60542	3988.802	5297.812	4895.074	5645.00557	3971.1367	3720.4897
Chemerin	14.247026	13.04261497	0.324610348	0.7141428	2.30443107	5919	Q99969	17931.74	22425.58	18277.32	17127.7866	23117.145	631.14843
TIMP-2	16.65531	16.88783654	0.327758438	0.7150127	0.8511433	7077	P16035	89909.86	100031.1	122261.3	152893.206	136185.45	87790.807
Kallikrein 14	12.051053	11.56414041	0.328255818	0.7150127	1.40144209	43847	Q9P0G3	4147.278	4094.605	4496.788	1932.52673	2630.8208	4643.318
MICA	8.1787902	8.498539159	0.354478695	0.7189428	0.80120927	100507436	Q29983	378.7239	222.2471	286.0317	263.880959	264.54317	462.45509
IL-15	11.079568	11.24605759	0.36412889	0.7189428	0.89100806	3600	P40933	2207.148	2147.598	2135.307	2112.89952	2175.0061	3024.1477
PDGF-BB	15.917293	16.72714625	0.356711152	0.7189428	0.57043995	5155	P01127	104065.7	63063.46	36110.99	40366.312	79911.517	221408.92
Lymphotactin	7.3710254	6.602794552	0.367163537	0.7189428	1.70317992	6375	P47992	126.314	186.5735	188.9553	176.938129	229.70539	114.71988
MSP	11.392807	11.59670518	0.359504296	0.7189428	0.86820128	4485	P26927	2128.2	2391.462	3815.525	3287.54274	2961.3004	3080.3659
IL-13 R2	10.25145	10.02665862	0.348769383	0.7189428	1.16860794	3598	Q14627	1205.661	1263.813	1185.78	1039.55549	1196.6676	794.49335
IL-23	11.478575	11.66603687	0.364291025	0.7189428	0.87814946	51561	Q9NPF7	2702.073	2892.305	2970.218	2497.02273	2964.1656	3902.5644
ST2	10.013801	10.42175773	0.367641185	0.7189428	0.75369024	9173	Q01638	979.2118	970.0793	1159.885	2653.11774	1555.85	864.52162
TREM-1	9.5939421	9.891192402	0.341888459	0.7189428	0.81380196	54210	Q9NP99	685.9292	830.412	807.0993	1653.86089	678.53661	756.07856
B2M	15.34313	15.12644634	0.34929103	0.7189428	1.1620592	567	P61769	48136.42	29869.03	49945.9	34759.5866	32458.48	44039.196
GROa	7.7612752	8.168280089	0.365652734	0.7189428	0.75418748	2919	P09341	235.0751	131.8415	324.6462	278.09903	151.19478	472.24134
Nidogen-1	16.695906	16.51631841	0.35380814	0.7189428	1.13255994	4811	P14543	109198.5	92591.65	118332.7	107363.397	94420.597	100568.21
OSM	7.7478212	5.953094303	0.355024882	0.7189428	3.46949797	5008	P13725	291.623	198.6744	168.9608	368.425372	180.7372	218.41648
PSA-free	10.876374	12.0777514	0.358592277	0.7189428	0.43485997	354	P07288	1988.183	1713.331	1946.938	2636.49608	3798.4835	32379.076
Siglec-9	10.30509	7.936160174	0.355827162	0.7189428	5.16557679	27180	Q9Y336	1177.275	1172.708	1463.255	2236.65892	1235.4979	1299.4743
Thyroglobulin	11.616242	12.25733782	0.346163597	0.7189428	0.64122567	7038	P01266	3759.118	2515.205	3269.124	3139.50351	2820.2209	16308.215
Cathepsin L	11.027136	11.56890527	0.346559107	0.7189428	0.68692819	1514	P07711	3313.601	1462.817	1872.176	5943.8202	2585.3122	2728.6916
Cystatin C	16.680923	16.84897329	0.342767353	0.7189428	0.89004488	1471	P01034	104510.5	95416.57	116299.6	121208.082	100936.42	126239.68
WISP-1	7.7759087	5.948327152	0.345249067	0.7189428	3.54941575	55515	Q96FT7	219.6216	193.6141	244.2015	214.598448	183.19833	365.07348
ANGPTL3	11.137955	10.95071034	0.355760013	0.7189428	1.13858717	27329	Q9Y5C1	2160.996	2250.172	2350.322	1813.82399	1747.5841	2024.9764
MMP-7	7.4420231	7.84931855	0.356211455	0.7189428	0.75403562	4316	P09237	162.1126	222.6052	143.1608	194.479199	381.80693	122.50928
LRIG3	11.185988	11.00886143	0.363211155	0.7189428	1.13062955	121227	Q6UXM1	2508.431	2267.848	2220.125	2456.85341	1995.3074	1872.0133
CD229	10.721799	8.146548501	0.337131153	0.7189428	5.95974661	4063	Q9HBG7	1522.048	2351.725	1343.204	4966.22327	1158.7102	1118.3314
NT-3	10.820648	10.35426803	0.37350303	0.7262765	1.38163793	4908	P20783	1095.611	2395.114	2250.429	771.722743	2712.0788	875.91855
IL-32 alpha	8.1993961	6.334384561	0.374692628	0.7262765	3.6427085	?9235	P24001	235.9106	429.6947	247.9078	419.404777	283.75136	353.18126
IGFBP-2	15.298799	15.44732069	0.376539369	0.7266549	0.90217472	3485	P18065	35174.24	42610.19	43694.02	46184.638	48142.678	43054.623
CD30	9.7913311	9.990186236	0.394031911	0.7310642	0.87124167	943	P28908	712.6525	1058.692	918.9976	844.671037	1219.6714	1021.8644
Fas	11.804439	11.56248561	0.386654852	0.7310642	1.18259253	355	P25445	3521.095	3168.53	4097.968	3682.30759	4085.0562	2792.0033
IL-21R	8.4814548	8.095983624	0.392461386	0.7310642	1.30628631	50615	Q9HBE5	305.5539	703.851	210.3065	183.708792	260.5463	368.65286
SCF R	15.322771	15.18176629	0.383359515	0.7310642	1.10267275	3815	P10721	38165.41	38137.65	47291.73	36635.2691	42477.147	35268.284
CXCL16	14.637617	14.48282936	0.391150383	0.7310642	1.11325805	58191	Q9H2A7	27410.8	22455.64	26902.17	21259.3354	21953.143	24139.402
MMP-13	12.999915	13.36391228	0.389800535	0.7310642	0.77700883	4322	P45452	9046.316	6338.244	9582.762	11493.0148	7961.0835	7150.9349
2B4	10.920072	10.33561017	0.395439296	0.7310642	1.4994796	51744	Q9BZW8	3378.698	1067.122	2014.158	1377.15417	872.86073	1734.4032
TACI	10.716929	10.34928941	0.391918016	0.7310642	1.29024029	23495	O14836	1622.026	1956.419	1499.867	2407.95161	863.61205	1267.4454
IL-1 F7	6.296103	4.783359408	0.382376711	0.7310642	2.85352187	27178	Q9NZH6	74.17527	78.3892	80.30387	0	80.916097	80.396717
CD27	11.20784	10.82591237	0.384984163	0.7310642	1.30308161	970	P32970	2953.091	1292.682	3461.873	1959.72546	1815.6066	1664.7611
PARC	15.813076	15.66249962	0.397966495	0.732658	1.11001256	6362	P55774	55204.5	65699.57	52610.27	48994.5602	63479.305	48935.846
IL-2 Rg	9.8327916	9.45068928	0.419060132	0.7328199	1.3032396	3561	P31785	570.3848	812.9853	1629.61	481.605656	614.89426	1389.7608
IL-1b	8.9805423	9.403960721	0.404490752	0.7328199	0.74565573	3553	P01584	608.8822	443.1508	474.8405	306.606799	701.24757	1312.4485
BDNF	15.887232	16.68937727	0.405935973	0.7328199	0.5734957	627	P23560	95662.45	60700.61	38339.15	25934.4764	60060.908	290818.58
BTC	9.7979545	9.962075618	0.409908723	0.7328199	0.89247205	685	P35070	937.2579	746.1643	1005.228	968.661284	1091.2944	1181.6983
Eotaxin-3	8.238757	8.538851386	0.422341499	0.7328199	0.81219924	10344	Q9Y258	364.4232	254.2356	294.53	280.517295	382.67006	699.3018
Activin A	9.9565438	10.17426684	0.417910854	0.7328199	0.85992158	3624	P08476	951.4911	900.7038	1141.169	1591.54726	985.50522	1213.1515
Galectin-3	11.217772	12.68838527	0.423036966	0.7328199	0.36082884	3958	P17931	1246.764	2337.804	4628.468	18411.6915	12412.303	23289.561
MBL	17.634132	17.49843925	0.410099658	0.7328199	1.09862018	4153	P11226	186376.7	214276.4	210783.1	192956.07	200621.8	193616.75
TLR2	10.724087	10.12627102	0.412968106	0.7328199	1.51342367	7097	O60603	2829.949	1064.893	1602.873	1165.5548	740.6127	1646.9852
TRAIL R1	10.212056	9.984673659	0.409398598	0.7328199	1.17070856	8797	O00220	1378.357	1054.861	1144.834	1319.91927	1184.3506	955.96643
FOLR1	7.5884907	7.940002262	0.42223519	0.7328199	0.78376251	2348	P15328	227.6828	182.5013	168.9093	367.172711	180.59238	415.84848
bIG-H3	12.94345	13.0886227	0.408994507	0.7328199	0.90427135	7045	Q15582	7707.113	8614.898	7358.559	8126.50679	8154.2051	9489.7477
IL-20	6.3476369	5.448089996	0.418927977	0.7328199	1.86548002	50604	Q9NYY1	80.70642	83.66539	77.07738	5.09107851	77.989886	84.225132
ROBO3	11.995223	11.29369346	0.419299758	0.7328199	1.62622757	64221	Q96MS0	6725.11	3671.808	2753.249	2016.29692	1043.0187	6967.9507
CF XIV	8.9960692	6.11544192	0.401106854	0.7328199	7.3647026	5624	P04070	323.1214	548.0119	747.1187	6407.0155	3604.8052	0
BCMA	14.314704	14.19568971	0.42898217	0.7381697	1.08599242	608	Q02223	22671.68	19303.53	19332.19	19041.9203	17399.397	19696.199
IL-6R	16.680429	16.8252121	0.43208582	0.7381697	0.90451529	3570	P08887	96427.62	115270.3	104231.1	94247.3875	126463.3	110924.33
NSE	16.843761	17.02907942	0.43136109	0.7381697	0.87945522	2026	P09104	96746.41	146645.6	114687.8	122614.868	114341.44	138684.74
SLAM	0	2.310253645	0.432835864	0.7381697	0.20162499	6504	Q13291	0	0	0	604.093627	0	0
MMP-2	10.886281	11.30447451	0.437393303	0.7419347	0.74836133	4313	P08253	2425.372	1207.493	2311.545	3248.39771	1020.5347	4613.5943
Marapsin	8.7255641	9.040173176	0.44010219	0.7419347	0.80406884	83886	Q9BQR3	452.0493	516.6903	322.4105	349.120709	481.55256	794.77897
Galectin-2	11.85262	12.37065068	0.439084754	0.7419347	0.69832422	3957	P05162	4212.672	4523.798	2651.907	2255.03547	3431.7511	9726.7466

Ferritin	17.432015	17.59746612	0.441999293	0.7422889	0.89164992	2512	P02792	162798	159106.9	213468.1	230889.067	161361.93	212964.37
BMP-2	8.1698895	6.553575037	0.44454195	0.7437204	3.06590811	650	P12643	359.5604	335.5046	195.8686	339.771594	393.77354	0
MCP-1	12.882789	12.68145754	0.453793574	0.7472727	1.14975903	6347	P13500	7591.293	8025.206	7069.246	7868.80086	4570.9673	8903.0992
LIGHT	9.5704364	9.425303784	0.454678165	0.7472727	1.10583234	8740	O43557	766.1994	677.8535	842.8806	688.143989	849.96163	567.27247
IL-2 Ra	10.031376	9.893497022	0.455157028	0.7472727	1.10028627	3559	P01589	986.047	1002.018	1156.68	856.719094	790.85701	1053.5727
IL-3	10.306676	10.57155764	0.452217192	0.7472727	0.83226722	3562	P08700	1542.048	1110.68	1183.396	1385.88809	907.52893	2701.0233
BMPR-IA	11.851997	11.69638546	0.452636678	0.7472727	1.11389376	657	P36894	3603.184	3834.871	3652.835	4257.37777	2888.0309	3323.9242
SOST	7.7642774	8.475497292	0.460083663	0.7507386	0.61080345	50964	Q9BQB4	215.1149	181.9285	258.9412	311.560308	255.31802	2289.0963
Layilin	5.5140179	4.221830439	0.460680497	0.7507386	2.44899091	143903	Q6UX15	81.8727	86.30349	12.18898	107.803587	84.732894	11.995701
FGF-4	10.546128	10.20029317	0.464525207	0.7521917	1.27088615	2249	P08620	1798.967	2300.545	805.8978	823.385605	1490.2931	2048.8348
Adiponectin	18.998888	19.11775283	0.464991222	0.7521917	0.92091217	9370	Q15848	500734.3	465416.3	616956.2	636031.747	537477.73	541637.99
MICB	9.2371398	8.93277693	0.474472257	0.7606002	1.23487321	4277	Q29980	996.0058	518.9195	422.9696	543.910882	454.64455	578.19494
VEGF R2	14.632325	14.46364785	0.473660354	0.7606002	1.12402768	3791	P35968	24788.37	29958.73	22053.8	20751.8094	24523.288	19980.702
TACE	7.5441073	6.019328501	0.475375128	0.7606002	2.87742594	6868	P78536	524.1862	101.9218	119.2783	121.73813	111.20442	1284.2993
sFRP-3	9.2185264	9.535571793	0.486198163	0.7750985	0.80271214	2487	Q92765	655.2951	560.9391	572.2849	853.551837	588.878	894.50445
ADAMTS13	11.020897	10.85365505	0.488496569	0.7759512	1.1229101	11093	Q76LX8	2531.639	2328.37	1519.724	2097.14905	1565.5334	1927.7386
IL-9	10.352938	10.21204277	0.493471802	0.7810345	1.10258891	3578	P15248	1304.255	1257.23	1361.006	1166.93811	1354.1378	1124.8755
ALCAM	14.980392	14.73015135	0.513069216	0.8091414	1.18940557	214	Q13740	39178.42	33290.55	25896.04	34220.6874	32946.367	37118.829
IGFBP-6	15.121843	15.22924437	0.516004224	0.8108453	0.92825872	3489	P24592	37126.54	36160.32	33762.29	40407.358	46070.49	33672.747
AMICA	10.794524	10.52895836	0.517835274	0.8108453	1.20210691	120425	Q86YT9	3111.517	1062.128	1692.28	1922.6189	1055.6706	1805.7087
EpCAM	11.103365	10.81590316	0.523751192	0.8172004	1.22049095	4072	P16422	2101.204	1871.045	2705.116	2852.74523	2326.0643	2011.6753
CD14	15.242079	15.36284669	0.541404002	0.8214864	0.91969787	929	P08571	36928.54	39172.14	40234.21	34309.9076	44306.11	47994.971
IL-1 RI	10.883937	10.98238993	0.538717465	0.8214864	0.93403401	3554	P14778	1698.149	2108.741	1881.409	2025.14892	2157.9379	1727.5244
G-CSF	9.3132897	9.55453776	0.538885134	0.8214864	0.84601314	1440	P09919	735.4483	586.7371	593.858	476.401002	1522.2484	612.82742
IL-17	9.979798	10.09190846	0.539606438	0.8214864	0.92523358	3605	Q16552	1224.855	854.2439	981.0338	1189.53666	1085.0722	1187.4656
TGFb1	12.740059	12.55804511	0.533971635	0.8214864	1.13446665	7040	P01137	4867.469	6755.535	9733.275	4844.61833	8422.2328	5427.5478
Galectin-7	11.43999	11.58785782	0.529796647	0.8214864	0.90258364	3963	P47929	3264.714	2527.887	2595.768	3514.19008	2558.8407	2735.2587
NCAM-1	16.847992	16.71123766	0.547035243	0.8214864	1.09942928	4684	P13591	114409.3	104026.1	137923.5	128756.919	86690.689	120541.39
IL-1 F9	5.447879	4.623233733	0.53698135	0.8214864	1.77109944	56300	Q9NZH8	76.04131	85.54975	11.47198	4.83652458	84.605667	7.6568302
IL-10 Ra	7.289748	7.100663025	0.543167089	0.8214864	1.14004044	3587	Q13651	121.5261	180.5213	171.2432	107.67631	169.84747	181.97733
Mer	6.9110419	7.146058222	0.544624391	0.8214864	0.84967538	10461	Q12866	166.8943	97.23276	104.6818	197.661123	181.17063	99.538792
Syndecan-4	14.27442	14.59996253	0.545170301	0.8214864	0.79799803	6385	P31431	31877.55	19043.5	12816.91	12329.6994	19690.039	46955.529
TSH	10.904414	10.69288871	0.549383018	0.8222059	1.15791156	7252	P01222	2037.317	1483.46	2326.162	1651.4224	1231.0545	3023.5897
Galectin-1	13.084654	12.87606756	0.56013935	0.8354621	1.15555577	3956	P09382	9855.324	8626.019	7708.828	5118.08999	6110.5818	10912.614
IL-17R	11.519349	11.33766983	0.572781965	0.8408395	1.1342033	23765	Q96F46	3249.433	2561.192	3036.078	2407.45562	2173.051	4280.9876
ICAM-1	17.423959	17.56134019	0.581797714	0.8408395	0.9091678	3383	P05362	186466.2	197660.4	147528.3	144042.164	262117.28	222040.64
TNF RII	14.822987	15.41030869	0.575493116	0.8408395	0.66557739	7133	P20333	31207.25	28011.17	27852.19	47406.6637	40451.66	37083.002
bFGF	11.526517	11.15269688	0.5877086	0.8408395	1.29577923	2247	P09038	3673.763	2365.21	2951.564	5907.90722	3807.8409	650.72391
FGF-7	10.420973	10.2262405	0.587550838	0.8408395	1.14451204	2252	P21781	1300.178	2073.89	953.4315	977.954166	1406.063	1841.4918
IL-18 BPa	9.474983	9.369512796	0.580182191	0.8408395	1.07584501	10068	O95998	653.8763	678.0481	809.4057	655.84759	822.70188	557.36431
FABP2	14.358462	14.2472548	0.581496148	0.8408395	1.08013189	2169	P12104	21154.63	22276.22	19664.12	25073.0094	21187.607	16005.092
IL-1 R6	11.379918	11.27335413	0.588587619	0.8408395	1.07666068	8808	Q9HB29	2945.395	2599.185	2469.555	2340.44666	2169.3191	3336.7
Gas 1	6.7455338	6.623023557	0.585412231	0.8408395	1.08862742	2619	P54826	154.8817	89.06721	86.99586	101.82157	90.839769	95.965605
IGFBP-5	6.3663781	5.78234005	0.587997386	0.8408395	1.49903913	3488	P24593	74.87503	87.43411	82.69387	11.9640345	86.514066	84.990815
Angiotensinoge	12.200509	12.56379035	0.569451432	0.8408395	0.77739415	183	P01019	2931.285	10546.54	3370.319	6180.05643	4629.1182	3511.9652
FLRG	12.976668	13.46597557	0.578077999	0.8408395	0.71236705	10272	O95633	7380.814	7275.899	9748.667	55316.624	5025.0883	10079.915
Tie-1	9.7217327	9.969554106	0.582408608	0.8408395	0.84216718	7075	P35590	916.6083	700.8882	933.7042	1152.20543	811.41831	1629.8374
NrCAM	11.375439	11.67461704	0.592510188	0.8437038	0.81271521	4897	Q92823	2353.222	2902.253	2742.548	9306.99425	2354.3569	3594.8683
L1CAM-2	13.175389	13.70825089	0.594985385	0.8444954	0.69118239	10752	O00533	6961.785	13085.9	8687.45	22394.4128	14883.563	3757.0038
Contactin-2	9.9438952	9.734782124	0.614120096	0.8486096	1.15597728	6900	Q02246	888.0242	650.0041	1649.954	551.584504	1212.0524	1159.4158
EGF R	16.384866	16.4571087	0.613185764	0.8486096	0.95115816	1956	P00533	80326.64	85704.8	91018.21	87489.4458	83044.65	101229.42
VEGF	10.895398	11.11698224	0.612966053	0.8486096	0.85762289	7422	P15692	2414.822	1932.643	1478.392	1821.22087	2903.4289	3551.1462
ENA-78	12.800277	12.22248826	0.615241954	0.8486096	1.49256004	6374	P42830	8041.86	6905.685	6532.141	949.860166	3152.6355	16819.056
NOV	15.52984	15.42680314	0.607797739	0.8486096	1.07403155	4856	P48745	42259.79	47909.72	52295.45	48275.3349	36548.34	47800.526
aFGF	12.113227	12.032051	0.609190691	0.8486096	1.05787079	2246	P05230	4508.717	4018.237	4796.786	4434.99098	4081.9611	4095.1668
Cathepsin B	12.350029	12.10800718	0.608080451	0.8486096	1.18264926	1508	P07858	5027.94	4773.324	5925.517	4007.56972	3887.535	6212.2416
FGF-6	6.1712132	6.578960276	0.612785719	0.8486096	0.75379962	2251	P10767	48.05064	90.95157	81.97687	101.185185	84.86012	97.369357
CD99	13.607803	13.49099447	0.607133164	0.8486096	1.08433353	4267	P14209	12703.85	12360.87	12387.45	9829.03858	11185.647	13785.645
TGFb3	10.73853	10.48742954	0.631452521	0.85982	1.19011433	7043	P10600	1410.062	1227.296	2876.461	1240.18869	3075.8754	1406.9706
VEGF-D	10.825185	10.44583633	0.641711712	0.85982	1.30075491	2277	O43915	698.747	3338.263	2554.789	742.881146	1245.7045	5060.7305
Fcg RIIBC	10.81236	10.47036691	0.640074913	0.85982	1.2675068	2213	P31994	1126.676	1156.739	4452.891	1188.43655	2900.9019	572.82114
CD97	12.379605	12.29751236	0.635035669	0.85982	1.05855247	976	P48960	4967.096	5359.156	5681.377	6284.8044	4506.0574	4840.4371
FGF-19	8.4115818	8.577368652	0.64271808	0.85982	0.89144219	9965	O95750	547.4778	313.7692	227.6989	287.144587	575.68884	399.13558
Legumain	14.250065	14.44609417	0.634782627	0.85982	0.87294995	5641	Q99538	12321.17	19575.6	30665.65	28969.9544	26877.124	19543.196
Notch-1	10.28816	10.38601959	0.636474639	0.85982	0.93441857	4851	P46531	1185.481	1430.214	1150.249	1495.98935	1444.6285	1280.4171
Transferrin	18.382243	18.45729221	0.630593768	0.85982	0.94930942	7018	P02787	333527.9	342073.2	349594.1	405790.112	333890.14	363914.92
IL-1 F8	7.7504512	7.871124966	0.642910876	0.85982	0.919758	27177	Q9NZH7	231.856	190.4456	222.9866	154.896064	235.49638	322.60778
Fractalkine	11.456501	11.35519371	0.638142723	0.85982	1.07274494	6376	P78423	3229.807	2427.441	2827.867	3541.22315	2518.8274	2493.3884
BMP-7	11.108547	10.95691182	0.670560268	0.8629295	1.1108279	655	P18075	1911.604	1748.913	3215.444	1456.64068	2077.5887	3094.013
HB-EGF	9.0409576	9.36497655	0.666423169	0.8629295	0.79884145	1839	Q99075	890.087	443.932	367.6248	357.019774	1205.4541	651.78178
TECK	8.3504403	8.183828321	0.657979833	0.8629295	1.12241946	6370	O15444	306.8757	360.3023	311.574	340.611666	231.90168	444.91257
ANGPTL4	13.125621	13.27776302	0.65028301	0.8629295	0.89991353	51129	Q9BY76	10441.45	6721.484	10168.16	7013.20521	7776.9837	16766.967
IL-18 Rb	8.5851065	8.909548551	0.65152901	0.8629295	0.79860719	8807	O95256	545.7516	325.5901	316.1981	447.722685	256.51473	1057.4523
CD200	12.055326	12.12217206	0.670012696	0.8629295	0.95472269	4345	P41217	4615.673	3937.441	4239.24	4526.28632	4278.5147	5224.8806
ACE-2	9.2403211	9.041256556	0.670060081	0.8629295	1.14795379	59272	Q9BYF1	704.8203	376.6882	828.8774	507.708524	416.56121	1121.7537
TRANCE	8.4573479	8.906315076	0.670695458	0.8629295	0.73256709	8600	O14788	605.2923	208.5075	340.8728	437.309765	514.15407	2726.5935
IL-17E	10.479045	10.31528826	0.670731593	0.8629295	1.12020046	64806	Q9H293	1493.505	1426.833	1361.529	1762.97693	1437.8579	1397.0762
DR3	2.3712101	3.779101249	0.651833419	0.8629295	0.37686217	8718	Q93038	0	0	137.4893	214.61552	163.74465	0
ESAM	13.276931	13.46926045	0.66357731	0.8629295	0.87519174	90952	Q96AP7	7317.467	12388.86	10782.94	6640.75269	11306.032	13986.916
Testican 2	5.7381027	4.388068007	0.667116806	0.8629295	2.54918262	9806	Q92563	0	123.3288	1222.07	423.570021	451.70058	0
L-Selectin	15.725842	15.67120185	0.680126156	0.8649003	1.03859985	6402	P14151	55464.86	57901.95	49558.15	52086.5965	52816.207	52507.186
Osteoactivin	14.833118	14.7068535	0.674984583	0.8649003	1.09146395	10457	Q14956	27388.13	27867.08	32579.3	30810.687	26867.262	26500.766
CXCL14	8.6227694	8.868104509	0.678958144	0.8649003	0.84361983	9547	O95715	422.1051	317.6875	453.2799	342.310695	376.03868	1393.7799
CD155	10.395145	10.7396396	0.680034761	0.8649003	0.78758393	5817	P15151	1231.615	2280.19	867.4857	1033.07307	1619.3376	619.29658
MIP-1d	14.514545	14.86091353	0.689001178	0.8729465	0.78656146	6359	Q16663	15575.93	17602.11	46758.39	69133.6033	55303.753	6261.1006
HCC-4	16.370071	16.22811762	0.694389233	0.8729465	1.10339809	6360	O15467	90724.67	75155.26	89114.21	67611.7041	58984.417	89576.824
IGF-1R	8.9336417	9.208191134	0.690876785	0.8729465	0.82670849	3480	P08069	723.7886	411.3358	390.2191	362.143014	346.70319	1574.8295
FGF-9	6.0275179	5.678069043	0.693972497	0.8729465	1.27407377	2254	P31371	83.03898	38.6921	82.21587	12.7276963	79.134925	72.357045

VEGF R1	9.2110763	9.005909442	0.696699105	0.873355	1.1528197	2321	P17948	608.4631	837.0877	406.5622	518.40958	942.64133	673.58124
IL-10 Rb	10.06164	9.904093099	0.707358065	0.8841976	1.11538927	3588	Q08334	678.235	1835.047	977.7296	599.745069	991.84954	1036.4894
TIM-3	6.7644824	5.389540435	0.713562907	0.8894269	2.59357483	84868	Q8TDQ0	0	1137.366	1127.898	3612.98812	853.41057	0
CRTAM	10.712531	11.00968545	0.718305541	0.8928091	0.81385632	56253	O95727	2315.548	971.5074	2096.228	6646.79438	1348.1886	1805.1056
FAP	17.554333	17.50077219	0.720589066	0.8931245	1.03782311	2191	Q12884	176092.7	196842.7	205719.2	203678.762	175941.08	191702.74
BMPR-II	9.9226626	9.84475543	0.726117482	0.8974486	1.05548583	659	Q13873	1034.054	1001.636	879.9499	1122.29264	1128.7222	864.6325
IL-1 F6	6.2709938	5.716642687	0.733524639	0.904064	1.46850797	27179	Q9UHA7	72.77573	87.9366	69.19039	92.9121827	81.933909	9.1881962
DKK-1	9.6734605	9.52124725	0.737408031	0.9063115	1.111273	22943	O94907	873.7718	633.4117	980.159	966.420929	791.03915	1098.5369
Endoglin	15.962955	15.88464957	0.755704512	0.9105108	1.05577686	2022	P17813	63852.09	56970.15	71637.66	74830.1803	49760.533	81992.985
Eotaxin	11.258641	11.09465238	0.770779964	0.9105108	1.12038045	6356	P51671	2681.169	2124.969	2578.426	1553.55588	897.38194	5766.0554
CCL28	13.255265	13.17681543	0.754020621	0.9105108	1.05588291	56477	Q9NRJ3	12347.36	7140.524	10598.79	9530.55202	10694.866	7644.4502
GCP-2	7.1520268	7.274522243	0.773581325	0.9105108	0.91859739	6372	P80162	231.1137	120.663	100.8743	213.732955	244.95018	92.933843
HCC-1	15.695453	15.74656825	0.748764491	0.9105108	0.96519008	6358	Q16627	53473.46	52216.71	53509.92	50442.5923	58905.093	54283.511
ICAM-2	14.18606	14.23267487	0.75391615	0.9105108	0.96820544	3384	P13598	18595.78	18807.57	18512.78	21595.4655	19017.361	20338.929
IL-2 Rb	10.33274	10.2787426	0.773934192	0.9105108	1.03813766	3560	P14784	1253.761	1292.741	1320.253	1290.80005	1244.6314	1294.0115
BCAM	11.579145	11.69700349	0.754052417	0.9105108	0.92155483	4059	P50895	3367.51	3442.109	2468.572	2959.54886	2580.9993	6403.9869
Decorin	16.903682	16.97128028	0.766580016	0.9105108	0.95422544	1634	P07585	103847.7	128485.2	138129.2	145005.611	117222.05	136830.31
IL-17B R	8.1029459	8.29524008	0.773376972	0.9105108	0.87521282	55540	Q9NRM6	203.8799	440.157	228.9306	327.524339	300.85701	930.59364
RANK	9.2316465	9.408500915	0.76077863	0.9105108	0.8846297	8792	Q9Y6Q6	695.9338	562.3401	552.4079	827.643406	541.38625	1844.6643
RBP4	18.579019	18.53566189	0.770860977	0.9105108	1.03050931	5950	P02753	428400.3	402178.6	348541.3	410102.476	374053.73	399520.52
IL-17C	5.2200277	4.835148012	0.770264872	0.9105108	1.30575091	27189	Q9P0M4	74.64178	82.78603	7.169988	6.61840206	77.608206	11.612859
RGM-B	11.297949	11.23545836	0.747010523	0.9105108	1.04426689	285704	Q6NW40	2330.924	2534.736	2698.268	2299.65484	1937.8785	2517.3071
Cystatin E M	15.533916	15.4368311	0.762516785	0.9105108	1.06960967	1474	Q15828	68618.34	41644.03	37368.2	74304.1452	37825.268	38405.639
Desmoglein 2	15.262257	15.31051775	0.745573572	0.9105108	0.96710162	1829	Q14126	36945.6	38500.54	42670.61	45788.0829	40385.245	40617.119
AR	10.648275	10.45276366	0.784633746	0.9206369	1.14513022	374	P15514	1133.197	800.6887	4545.284	794.684015	1938.4484	2889.0502
IL-13 R1	11.584035	11.62973628	0.79741843	0.9261933	0.96881893	3597	P78552	3001.051	2889.294	3333.771	2910.33247	3128.5784	3224.449
IL-27	9.970482	10.11978543	0.796192077	0.9261933	0.90168569	246778	Q8NEV9	2291.681	632.0526	694.7591	929.446816	761.58908	1281.0738
IL-1 F10	7.6143907	7.540113428	0.797789219	0.9261933	1.05283346	84639	Q8WWZ1	175.7581	181.275	232.5466	157.696157	187.15028	197.54622
IL-1 R5	7.2409107	7.15609905	0.795074625	0.9261933	1.06054928	8809	Q13478	163.0456	142.9598	145.5508	103.603448	107.37922	179.8079
LYVE-1	15.47216	15.4338311	0.811149203	0.9303616	1.02692392	10894	Q9Y5Y7	46500.41	48450.82	41683.72	40275.2894	46319.786	41524.382
IGF-1	11.098009	11.14632092	0.816897036	0.9303616	0.96706692	3479	P05019	2552.117	2320.518	1775.877	1970.04912	2654.0822	2675.8889
FAS L	10.274258	10.20598592	0.806047634	0.9303616	1.04845984	356	P48023	1356.036	1102.809	1266.925	1791.67226	1049.2546	1183.1754
IFNab R2	11.536078	11.57178194	0.808038619	0.9303616	0.97555544	3455	P48551	3093.771	2940.541	2875.821	2718.29401	2834.1239	3364.7374
Thrombomoduli	13.159984	12.90540777	0.81668899	0.9303616	1.19298534	7056	P07204	12521.04	5704.274	10731.43	33147.9809	5174.5456	18312.12
Fetuin A	12.618688	12.67237101	0.815503976	0.9303616	0.96347331	197	P02765	7612.998	5485.465	5954.319	6305.92306	4713.6436	7231.6388
Pepsinogen I	17.173323	17.14068355	0.820409754	0.9303616	1.0228816	643834	P0DJD8	158856.5	133248.7	152538.4	150003.007	155175.37	139070.68
uPA	11.51569	11.45883998	0.820095698	0.9303616	1.04019213	5328	P00749	3193.272	2682.414	2927.496	3343.15595	2584.8425	3038.0578
Thrombospondi	10.766608	10.68141933	0.809349508	0.9303616	1.0608263	7058	P35442	1439.437	2165.885	1692.891	2638.58297	2113.3067	993.51313
Adipsin	15.912538	15.88177924	0.824008532	0.9315446	1.02154929	1675	P00746	61331.01	57908.83	66070.53	62234.7097	55959.12	64845.182
Dkk-3	17.184474	17.14672889	0.825687289	0.9315446	1.02650806	27122	Q9UBP4	158617.9	127415	163510.1	160853.314	136456.05	150911.63
E-Cadherin	10.097971	10.02132979	0.83280269	0.9371693	1.05456016	999	P12830	1230.913	882.7709	1208.085	1880.10538	804.21403	1172.4697
Cripto-1	10.347152	10.52846166	0.836087084	0.9384651	0.88190209	6997	P13385	738.3328	3324.659	897.8595	2335.82819	892.85607	1556.4916
IGFBP-4	11.7512	11.62615684	0.840976806	0.9415516	1.09054041	3487	P22692	2307.81	9118.317	1944.542	3967.18973	2837.0736	2528.9768
CTLA4	8.2951371	8.333691183	0.844037799	0.9425803	0.97363023	1493	P16410	351.8077	278.2565	313.5675	270.590823	345.67458	344.55736
E-Selectin	13.190517	13.12264928	0.846421364	0.9428491	1.04816647	6401	P16581	9278.802	7982.785	11026.48	13082.8377	5967.3346	8204.1021
B7-1	10.05522	10.19080609	0.859115763	0.950424	0.91030026	941	P33681	2163.836	945.6476	586.6998	756.138059	861.32659	1119.9533
RANTES	17.752261	17.68016548	0.864021772	0.950424	1.05124273	6352	P13501	253024.2	211210.1	201375.5	168615.905	213639.08	232883.42
GH	13.073082	12.93475407	0.861077216	0.950424	1.10062857	2688	P01241	10416.03	9643.024	6369.406	15271.6259	10204.952	9169.7723
MCP-4	8.2431125	8.290305069	0.862586476	0.950424	0.96781787	6357	Q99616	318.0948	298.8031	289.7479	281.324705	448.17098	298.4388
VE-Cadherin	7.6949694	7.783122198	0.862289942	0.950424	0.94072647	1003	P33151	116.1582	271.518	277.6646	331.842479	254.99227	265.33256
LIMPII	11.16437	11.29816748	0.868867932	0.9509997	0.91142932	950	Q14108	5160.524	1598.533	1463.4	2575.9317	1350.694	3242.7331
NGF R	11.478774	11.5767694	0.867660621	0.9509997	0.9343303	4804	P08138	4439.063	3048.708	1715.795	3114.33249	3148.2747	4043.8504
Dtk	13.194105	13.10563783	0.876877145	0.9518018	1.06323964	7301	Q06418	6622.874	11567.41	10740.82	19567.3915	7429.6913	12179.413
Trappin-2	16.062214	16.03772735	0.875623345	0.9518018	1.01711772	5266	P19957	70070.1	76275.61	59935.91	62177.7526	65304.443	69083.327
VEGF-C	9.9923708	9.928333382	0.878253451	0.9518018	1.04538725	7424	P49767	1085.917	1172.446	827.6101	903.485409	1005.6622	646.37601
Syndecan-3	10.036487	9.953575809	0.873545275	0.9518018	1.05915343	9672	O75056	1133.263	1158.274	879.9499	648.819566	861.05595	667.68561
PDGF Rb	11.262025	11.29667731	0.883441863	0.9532854	0.9762671	5159	P09619	1855.393	2821.989	2825.462	2526.68308	2614.0188	1958.8781
BMP-4	10.475808	10.43271494	0.906684611	0.9532854	1.03032069	652	P12644	1538.321	1692.36	1106.948	1934.62715	1668.7836	1108.915
AFP	14.74307	14.67484024	0.900167646	0.9532854	1.04842964	174	P02771	30444.46	18918.65	35799.05	65161.0319	19087.049	22376.017
HGF R	17.070829	17.05599993	0.909954203	0.9532854	1.01033159	4233	P08581	134418	142499.2	136212	137440.399	136190.75	133655.57
Serpin A4	17.511154	17.53498954	0.896607348	0.9532854	0.98361427	5267	P29622	148176.6	216905.6	202810.5	195902.091	164536.98	201144.99
Troponin I	9.6208434	9.588238001	0.907940799	0.9532854	1.02285765	7135	P19237	759.7891	789.1206	810.9507	986.760304	1023.4871	752.45901
Dkk-4	7.0687536	7.108698524	0.903608429	0.9532854	0.97269209	27121	Q9UBT3	112.6624	166.2002	126.3113	101.057908	173.02813	104.89857
EDA-A2	6.3584299	6.54475756	0.896431303	0.9532854	0.87883994	1896	Q92838	74.17527	91.83094	78.15287	112.894666	83.333401	82.374731
TGFb RIII	11.56752	11.61665723	0.892983708	0.9532854	0.96651392	7049	Q03167	2371.043	4109.152	2866.681	4906.27236	2732.6994	1791.1878
CNTF	11.600043	11.56854589	0.901047198	0.9532854	1.02207197	1270	P26441	2665.28	3178.122	3528.145	4292.67235	2563.1871	2895.8064
EMMPRIN	14.021622	13.98922647	0.895979992	0.9532854	1.02270869	682	P35613	17388.52	16918.59	15634.39	23500.8104	13627.354	12595.314
GITR L	11.006378	11.06827411	0.885229627	0.9532854	0.9580043	8995	Q9UNG2	2378.425	1647.592	2218.038	1342.44573	1675.8672	4843.9013
IL-15 R	10.643616	10.6863508	0.905429512	0.9532854	0.97081274	3601	Q13261	1654.052	1545.95	1598.023	3036.83602	1621.957	1547.2036
Thrombospondi	17.119682	17.17128465	0.905395401	0.9532854	0.96486396	1311	P49747	182785.6	123952.9	127469.3	210745.832	110904.81	197528.58
SDF-1a	9.2583899	9.239153492	0.918720195	0.9556428	1.01342296	6387	?P48061	608.7358	527.414	711.9239	563.341475	581.75668	745.02262
PAI-1	14.533656	14.4828823	0.917961555	0.9556428	1.03582045	5054	P05121	26474.54	31084.69	16209.42	9673.4133	23779.138	37559.711
CD6	11.099447	11.06568858	0.916552002	0.9556428	1.02367513	923	P30203	2119.497	2969.571	1675.955	2611.17334	1555.8572	1866.6698
PGRP-S	17.809451	17.79512009	0.924776221	0.9596734	1.00998264	8993	O75594	213859.8	250507.2	226246.3	226576.602	231777.32	224115.61
DR6	16.204032	16.22341649	0.929994151	0.9605573	0.98665357	27242	O75509	79921.78	71708.29	75066.69	76277.1733	55029.102	100873.46
6Ckine	9.7742034	9.729246566	0.929317378	0.9605573	1.03165229	6366	O00585	762.1077	508.8604	1724.631	438.76965	1001.6344	1185.6377
MCP-3	9.6309967	9.588673774	0.934688659	0.9614986	1.02977059	6354	P80098	974.6109	887.4568	574.1005	390.55574	686.14473	1420.2106
DPPIV	13.192845	13.22740527	0.93527591	0.9614986	0.97632924	1803	P27487	10407.63	11920.18	6615.27	17721.917	5498.7322	8191.0217
Siglec-10	2.5653352	2.321038581	0.943928534	0.9681318	1.1845151	89790	Q96LC7	0	0	206.3617	622.460489	0	0
IL-29	9.9659897	9.97395177	0.954351591	0.9760568	0.99449628	282618	Q8IU54	943.3294	1040.426	1016.263	1052.51658	1014.1661	991.8912
MCP-2	9.8333612	9.881811366	0.960459799	0.9760568	0.96697455	6355	P80075	2248.838	279.99	1200.069	499.325399	3287.7069	1649.4116
CD40	8.7927082	8.816344633	0.958045217	0.9760568	0.98374995	958	P25942	575.6793	390.5973	385.2181	969.157256	337.56832	484.15226
Granulysin	13.041283	13.06431819	0.960528655	0.9760568	0.98415994	10578	P22749	11858.14	9188.195	5495.944	6676.30828	5328.4434	19293.926
Angiogenin	16.66347	16.65858671	0.969140992	0.9825393	1.0033903	283	P03950	100667.4	107270.9	103568.8	105057.542	100256.15	106526.97
WIF-1	13.281195	13.28658118	0.97204571	0.9832186	0.99627352	11197	Q9Y5W5	11185.79	8659.306	10182	10114.3286	9457.4567	10395.156
CD40L	11.789572	11.79404901	0.98631299	0.989838	0.99690181	959	P29965	3205.557	2970.84	4654.65	4005.05783	3217.8592	3055.759
NRG1-b1	8.5915507	8.596582996	0.989838034	0.989838	0.99651797	3084	Q02297	532.289	354.3606	301.9085	502.335887	485.62033	294.4558
IL-5 Ra	6.7653827	6.760718066	0.986850282	0.989838	1.00323848	3568	Q01344	85.02165	108.162	136.1103	102.330678	140.20368	107.83369

CD84	11.56749	11.56123454	0.983472608	0.989838	1.00434552	8832	Q9UIB8	2353.697	4236.38	2800.897	3834.27107	3840.8577	2442.3669
ErbB4	2.7005724	2.652917417	0.98974501	0.989838	1.0335835	2066	Q15303	0	0	273.7008	1563.69925	0	0
