# Supplementary material for: LCN2 secreted by tissue-infiltrating neutrophils induces the ferroptosis and wasting of adipose and muscle tissues in lung cancer cachexia
Source: J Hematol Oncol. 2023 Mar 27;16:30. doi: 10.1186/s13045-023-01429-1 (PMC10044814; doi:10.1186/s13045-023-01429-1)
Supplement: Supplementary file 3 — Additional file 3. Table S4: Mice serum proteomics. [file 13045_2023_1429_MOESM3_ESM.pdf]

Table S4 mice serum proteomics

proteinID	AveExp.L3	AveExp.C	P.Value	adj.P.Val	foldchange	entrezID	uniprotID	cachexia 1	cachexia 2	cachexia 3	control 1	control 2	control 3
G-CSF	14.1266613	11.9299173	9.08E-06	0.000605346	4.584435189	12985	P09920	16721.83517	17847.05386	19174.45131	3122.59948	5300.921127	3585.742958
OPG	13.47170859	11.03514559	1.37E-05	0.000684667	5.413505075	18383	O08712	13629.39567	9902.379792	10860.08554	1804.275352	1846.172017	2770.269061
Lipocalin-2	18.11590256	15.9649594	0.000153357	0.003365305	2.396958788	16819	P11672	705153.4908	541465.5407	602937.3132	228682.6153	267883.9632	273394.6261
MCP-1	15.17824867	12.17513047	4.63E-05	0.001852884	8.017309702	20296	P10148	29987.66714	41214.7187	41237.6421	5013.167741	4527.272464	4355.191946
4-1BB	13.91659938	12.90035345	0.000171144	0.004889829	2.022648926	21942	P20334	13653.05706	15913.94818	17015.67147	6789.110909	7655.997275	8593.976059
IGFBP-5	14.45506969	12.09315536	0.00015815	0.004889829	5.140520114	16011	Q07079	27352.84661	15692.25301	26392.52026	4080.65784	3803.784332	5369.823416
Activin A	9.835875724	11.11273701	0.000207973	0.005199313	0.412692383	16323	Q04998	1226.977738	751.0301631	825.5233478	2333.016208	2284.216051	2034.965368
CCL6	14.56342105	11.43831064	0.000332438	0.006995716	8.724729535	20305	P27784	10480.81302	40620.93545	33332.63729	2219.372242	2337.843684	4114.25566
OPN	18.22612654	17.43876928	0.000349786	0.006995716	1.725910031	20750	P10923	326102.6503	293199.2285	301516.4272	167030.5784	174277.4106	192634.2446
MCP-5	15.13911217	12.58568301	0.000458046	0.00832811	5.870279369	20293	Q62401	26981.28354	45790.4834	38028.20094	5102.058697	7967.179881	5711.347396
Galectin-1	16.03807118	14.50239329	0.000588884	0.00981473	2.89924628	16852	P16045	73197.24735	56261.35525	73977.0536	18249.93933	24266.74558	28225.14129
P-selectin	17.03343527	15.47606408	0.001593118	0.024509505	2.943170635	20344	Q01102	161450.7853	120904.4105	123660.994	33974.34275	48951.78555	56928.48451
bFGF	12.25039721	11.60810613	0.002093993	0.028661158	1.560805838	14173	P15655	5092.285223	4628.021914	4904.957959	3323.598222	2937.802561	3112.54277
IL-1ra	15.07466256	13.86458475	0.002149587	0.028661158	2.313501152	16181	P25085	43221.59471	30807.7849	30858.67965	12818.43576	19634.97964	13182.96903
Pro-MMP-9	19.85661045	18.64180594	0.002552267	0.031903339	2.32109329	17395	P41245	998694.7988	1001334.348	855643.3779	324659.8039	618908.4489	340541.4923
MIG	12.27638585	11.36120691	0.002751964	0.032376053	1.885802956	17329	P18340	4376.954645	5175.253372	5386.587704	2345.541164	2957.104117	2621.711788
IFNg R1	10.29078311	11.44562796	0.002981902	0.033132248	0.44911448	15979	P15261	1803.735117	938.8466914	1157.864598	2359.001815	2687.96653	3418.27693
LIX	13.69874557	15.19163679	0.003498884	0.033653046	0.355299801	20311	P50228	23653.08919	11546.41766	8605.148896	37198.36394	31760.04912	44358.30303
TREM-1	12.00307104	10.85042937	0.003322976	0.033653046	2.223206051	58217	Q9JKE2	4581.20347	3680.746049	4098.445033	1535.514294	1602.506145	2553.505758
IL-20	12.18758582	10.90484988	0.004312658	0.039205986	2.432999364	58181	Q9JKV9	7752.172753	3565.675013	3669.656288	1957.398135	1839.232023	1954.632128
CD40	11.2842192	10.3953442	0.004664277	0.040558929	1.851731603	21939	P27512	2097.28466	2311.294357	3196.111984	1132.465224	1226.110195	1755.46497
CXCL16	13.95047272	12.69095245	0.004985425	0.040645746	2.394161149	66102	Q8BSU2	15330.14629	13619.21747	18999.87295	6310.460121	5448.696506	8404.623374
Gremlin	0	8.018197671	0.005080718	0.040645746	0.003857287	23892	O70326	0	0	0	263.4759521	327.4182425	199.6044527
PlGF-2	11.12072726	8.604073032	0.009207009	0.070823148	5.722534386	18654	P49764	4551.755736	3180.730732	761.2188108	250.2120827	344.8835861	677.0812342
E-selectin	17.43501617	16.03014213	0.009756568	0.071543599	2.647946609	20339	Q00690	274060.2969	115371.7833	175968.5185	50515.46359	74131.42046	80022.90335
Progranulin	18.44651757	17.55906619	0.010016104	0.071543599	1.849905247	14824	P28798	403262.1059	294112.7473	384379.6311	162839.4525	195234.7746	226513.0566
IL-15	12.69003242	12.05111043	0.010698719	0.073784267	1.557165188	16168	P48346	7458.216169	5545.909094	6973.2414	3447.122019	4684.989972	4728.913281
TNF RI	16.81305207	16.35329501	0.011587623	0.077250823	1.375310206	21937	P25118	123375.5704	121004.7194	102248.3094	85731.79991	81345.08506	84141.09355
Prolactin	15.93881939	14.75715937	0.012345087	0.079645726	2.268376341	19109	P06879	56984.31741	58192.1151	74739.09005	27596.58581	34719.45822	22159.84553
Epiregulin	9.0889321	10.55251397	0.014359601	0.088998518	0.362591781	13874	Q61521	1006.467167	459.3954532	347.1461883	856.3614851	2010.910045	1962.799641
H60	8.240152693	8.924926437	0.014684756	0.088998518	0.622103383	15101	Q3TDZ7	213.4567776	338.055651	379.1794389	535.0021738	458.4334276	465.2539864
Gas 6	14.63637379	13.79211789	0.015778118	0.090160675	1.795338515	14456	Q61592	23541.45827	34118.5632	20562.98794	11301.02023	12619.8992	20010.38699
TCK-1	16.66655996	17.17498037	0.015520236	0.090160675	0.702991713	57349	Q9EQI5	114201.3665	110358.1371	89313.09859	151029.768	147085.2933	145851.9331
BLC	14.19298551	12.40314782	0.017399004	0.094528026	3.45775991	55985	O55038	22423.82382	33963.23111	8624.658826	4928.253148	5266.9451	6118.786014
IL-28	11.77823991	11.17315682	0.017487685	0.094528026	1.521066351	338374	Q8CGK6	4275.082595	2958.819371	3422.727842	2062.52529	2221.865954	2683.373738
TWEAK	10.47395643	11.66818609	0.018360601	0.09663474	0.437019736	21944	O54907	2172.14467	944.0376005	1399.836506	2518.406058	3221.900071	4243.993386
Fas L	12.02543871	11.57946319	0.020007213	0.099548207	1.362234914	14103	P41047	4107.253305	4267.204348	4130.910155	2695.332849	3131.082524	3392.833771
Granzyme B	10.29881668	11.13961665	0.02001423	0.099548207	0.558333888	14939	P04187	1818.816921	993.9411128	1102.908668	1612.141866	2727.570413	2607.96664
PF4	18.51653515	18.15513998	0.020905123	0.099548207	1.284667641	56744	Q9Z126	366507.4555	379190.131	379449.6627	299955.1565	296016.1959	280123.0581
Testican 3	8.147250732	9.381723921	0.020621008	0.099548207	0.424997662	72902	Q8BKV0	330.2309075	332.6419484	205.2001866	797.2081045	378.2428616	979.6349904
IL-13	12.06791924	11.48024556	0.023308713	0.108412617	1.502821524	16163	P20109	4677.992042	4280.23671	3949.894949	2642.229161	2988.832702	2949.618799
CD30	12.04018548	11.32046486	0.025113864	0.111617176	1.646863075	21941	Q60846	5224.991519	3330.233643	4290.381747	2054.161441	2627.188329	3095.677951
TROY	11.62921196	12.14228683	0.02493454	0.111617176	0.700727359	29820	Q9JLL3	3628.403822	2876.108895	3042.920341	4545.40908	4265.07546	4762.00029
ANG-3	9.567234611	10.36063226	0.028572395	0.121584659	0.576983654	11602	Q9WVH6	1015.832481	540.6803703	791.6496494	1257.393086	1236.477674	1458.58555
TGFb1	12.24937235	13.36145496	0.028277587	0.121584659	0.462625723	21803	P04202	7751.925668	3864.867757	3849.99454	7732.20472	9636.029978	15641.04275
TRANCE	11.43139508	12.27659552	0.032939954	0.137249808	0.556633467	21943	O35235	3953.74359	2353.369415	2261.483903	3772.825667	4788.410106	6756.820192
CTLA4	10.62289492	10.0323183	0.033862454	0.1382141	1.505848491	12477	P09793	1702.065278	1327.434875	1732.239069	786.65779	1227.682231	1185.612854
Decorin	17.59508455	17.12328231	0.037868585	0.14889423	1.386840856	13179	P28654	210571.7366	178007.6941	207061.4371	137344.4541	134006.2775	158095.4509
HGF	12.40016537	13.3008955	0.0387125	0.14889423	0.535615595	15234	Q08048	9247.20826	4040.967683	4223.883553	9058.435047	9678.756658	11719.33393
TNF RII	16.87079175	16.51395386	0.03853506	0.14889423	1.280615962	21938	P25119	139508.2408	110596.9725	111555.0077	91916.96862	97542.26327	91407.94018
CD48	13.64046999	13.0803136	0.04029491	0.152056264	1.474429037	12506	P18181	15079.81418	12321.58711	11204.85967	7845.635265	7798.885379	10614.2091
Tryptase	0	5.117972809	0.041067819	0.152103035	0.028796299	70835	Q9ER10	0	0	0	183.7355426	0	225.6937447
IL-4	11.81510747	12.27881824	0.042167266	0.153335513	0.725118775	16189	P07750	4509.223318	3580.322182	2895.435195	5183.125197	5080.936271	4656.717114
Marapsin	7.599040709	8.386658558	0.047466729	0.169524032	0.579299832	213171	Q8BJR6	158.6618262	303.4079543	148.9548393	298.0598803	406.4699408	306.6476743
EGF	15.64884149	12.80449609	0.049965928	0.169646423	7.181799604	13645	P01132	120477.8993	29486.52028	38172.57313	2153.112214	3551.275299	47844.07825
IL-17B R	10.22947181	11.21191877	0.050045695	0.169646423	0.506120578	50905	Q9JIP3	2096.978904	970.6999971	847.7899747	1597.274479	2394.270406	3485.307268
SLAM	9.044608335	10.16804729	0.048913184	0.169646423	0.458998405	27218	Q9QUM4	913.3005389	300.836776	532.6224527	722.4257215	1156.747084	1817.251913
TIM-1	12.72986605	12.2315348	0.053779197	0.17926399	1.412578701	171283	Q5QNS5	7246.206149	7421.825345	5826.398331	3712.095932	5657.86142	5292.115526
IL-22	8.096191803	9.335042069	0.056305334	0.183025883	0.423710191	50929	Q9JJY9	658.9775362	160.6822309	191.0429211	406.978556	723.4570749	910.4503707
PDGF-AA	14.7626318	15.20438268	0.056738024	0.183025883	0.736240553	18590	P20033	33469.44231	28564.74682	22462.52583	32555.69885	39351.35946	42005.3178
Meteorin	7.936568866	8.933773147	0.05774029	0.183147864	0.500969862	70083	Q8C1Q4	124.20189	346.9581841	336.518773	415.1912887	528.3596011	529.8322434
VEGF R3	11.33443455	11.84901232	0.058607316	0.183147864	0.699997769	14257	P35917	2761.334439	2150.588783	2896.202288	2724.429904	4211.641169	4371.551137
Fcg RIIB	15.62373755	15.06464163	0.061871854	0.185419152	1.473345633	14130	P08101	49194.82312	41266.39119	63401.39899	24406.72598	41852.11445	39396.79503
LOX-1	10.86559691	12.65865539	0.062115416	0.185419152	0.288559656	108078	Q9EQ09	2894.025804	1210.5039	1850.972415	8732.286852	1844.511322	16771.99021
MMP-10	11.98310721	12.73189053	0.061837622	0.185419152	0.595105224	17384	O55123	6941.765209	2670.132843	3576.636163	5414.573025	7221.50016	8047.424473
IL-10	12.83050631	13.20804791	0.064466545	0.189607487	0.769748148	16153	P18893	7991.515946	8322.441152	5808.186896	9834.828703	9367.07149	9194.90897
ADAMTS1	10.36442671	10.99407588	0.065982819	0.191254548	0.646333568	11504	P97857	1911.983549	1083.956195	1102.790228	1505.743088	2286.307345	2460.932351
TWEAK R	16.92432156	16.37291469	0.067096144	0.191703269	1.465514122	27279	Q9CR75	131468.7471	100662.3685	145373.4393	69420.62732	89248.98378	98652.47328
MCSF	12.56784472	12.22742379	0.07372243	0.207476656	1.26612595	12977	P07141	6876.679989	5405.491776	6018.178389	4156.325622	5361.86645	4944.987014
MIP-1a	12.28674245	11.89330281	0.075728979	0.207476656	1.313521346	20302	P10855	4849.694853	4565.15993	5631.212208	3308.334119	4944.899963	3362.076402
Shh-N	10.13907046	10.74334377	0.075510917	0.207476656	0.65780263	20423	Q62226	1493.828368	888.7072288	1077.088856	1377.624998	1549.648976	2355.386598
Lungkine	10.31779114	10.5821206	0.082525914	0.22304301	0.832585613	20309	Q9WVL7	1196.356441	1304.942935	1328.677167	1579.232508	1387.617554	1640.736738
TPO	10.63289929	11.1423307	0.088867324	0.23697953	0.702499252	21832	P40226	1552.281185	1243.845818	2069.614428	2074.106005	2102.951816	2644.155494
Axl	13.85995105	12.67967236	0.093788846	0.238449071	2.266205507	26362	Q00993	30945.16678	7482.674607	14191.57695	4599.345185	7368.806158	8328.878335
IL-33	10.54377689	10.91955368	0.090951779	0.238449071	0.770690339	77125	Q8BVZ5	1307.894608	1570.815884	1615.862097	2162.210073	1715.436587	1956.100496
SCF	10.65925056	9.816905925	0.095193451	0.238449071	1.792961653	17311	P20826	1165.103429	1557.073488	2326.755907	887.3400961	637.5452704	1292.5359
TACI	10.26088117	10.93310637	0.095270774	0.238449071	0.62753803	57916	Q9ET35	1437.639863	982.1094253	1305.005759	2182.959074	1372.568853	2490.63235
TCA-3	13.51343967	13.14412142	0.096571874	0.238449071	1.291742262	20290	P10146	13493.51717	10192.20187	11623.76217	8612.289447	9889.006712	8707.810054
TSLP	12.47702925	12.84585719	0.09644069	0.238449071	0.774411377	53603	Q9JIE6	6978.515871	4403.500671	6026.791194	6690.17582	7234.402026	8240.379177
IL-1b	11.94583209	12.31623786	0.097923033	0.238836665	0.773564897	16176	P10749	4185.886855	4881.769678	3002.335757	5116.937991	5570.746301	4650.41121
GITR L	8.842018397	9.449479202	0.108748919	0.252904462	0.656350887	240873	Q7TS55	269.7308039	695.9413998	511.1584509	681.7266349	576.6722747	865.5660685
Kremen-1	9.627716409	10.15850469	0.107451741	0.252904462	0.692176428	84035	Q99N43	542.348253	1136.99785	799.7087413	1133.673817	1244.300974	1055.570569
VCAM-1	18.43467378	18.19225344	0.105170978	0.252904462	1.182975622	22329	P29533	355531.2375	357159.0446	350288.767	267679.5994	323006.0319	310750.3661
VEGF	13.99470677	13.64636764	0.10792894	0.252904462	1.273094164	22339	Q00731	17975.63526	16147.21099	14983.65441	9994.671494	13912.55373	15157.21028
IL-2	13.29097812	13.63932418	0.112697969	0.259075791	0.785484075	16183	P04351	11709.12241	10810.60025	7951.393084	12476.41651	12780.40622	13025.68134
Prostasin	11.45788586	11.03220021	0.119223478	0.27096245	1.343210723	76560	Q9ESD1	3567.88194	2346.419009	2655.924367	1675.438479	2439.717779	2243.73126
Fas	9.770381455	10.2410283	0.120931274	0.271755672	0.721640971	14102	P25446	1173.218404	735.3394606	769.3830226	869.0310844	1526.252622	1332.903667
IGFBP-2	16.01374008	15.29822847	0.123046734	0.273437186	1.642065429	16008	P47877	41148.56252	79713.07322	88296.43764	37213.59733	44446.39446	39546.34381
Galectin-3	16.45436235	16.03728559	0.131565818	0.286012647	1.335219338	16854	P16110	109207.0934	71874.10557	92242.66627	56559.71748	83231.2897	64609.82453
IL-17B	10.49871039	11.20188755	0.131290268	0.286012647	0.614218057	56069	Q9QXT6	2144.048728	1208.066112	1166.866	1241.620728	2891.867711	3635.179534
ICAM-1	13.13704025	12.80615449	0.134623977	0.28951393	1.25778537	15894	P13597	10136.56877	8862.389819	8134.717158	5911.056188	8965.440485	6929.287724
CD6	8.043826897	9.378641587	0.139748454	0.294374964	0.396442987	12511	Q61003	931.8794747	128.7258173	150.8614613	364.153638	1121.384875	718.6807249
IL-1a	12.10738147	11.7012836	0.139828108	0.294374964	1.325096907	16175	P01582	5015.177398	4347.698352	3937.427065	2889.918102	3926.412387	3251.143967
IL-17	12.52357202	12.8145798	0.143534459	0.299030122	0.817330918	16171	Q62386	5808.789436	6952.126563	5052.263701	6411.180053	7930.030997	7350.625069
CCL28	8.338708631	9.076921525	0.149317155	0.307870422	0.599481487	56838	Q9JIL2	357.8279645	272.7586769	344.4220797	406.0735847	348.592596	1105.730285
Pentraxin 3	13.66004379	12.60235914	0.156128007	0.318628587	2.081588144	19288	P48759	26917.49438	4525.903634	17798.04699	4844.560634	5233.163715	9480.411872
TNFa	12.58401325	12.82499773	0.168555079	0.339276898	0.8461677	21926	P06804	6342.089213	6997.867601	5212.960874	6707.99632	8526.131786	6677.308945
VEGF R1	11.77210411	11.24561586	0.169638449	0.339276898	1.44041871	14254	P35969	4319.671903	2524.290558	3920.089013	1778.746015	2297.41322	3498.588197
IL-12p40	12.26459101	12.54128202	0.183023646	0.362423062	0.825482186	16159	P43431	5020.92821	5860.47455	4046.17472	5519.320285	6891.977459	5564.895991
ACE	15.65287873	15.19491522	0.185721179	0.364159174	1.373601492	11421	P09470	75980.76796	37055.87357	48570.31706	29287.263	34121.91014	52799.266
6Ckine	7.719207613	8.223563569	0.190902568	0.368729235	0.70497502	100042493	P86792	308.8121012	153.1318177	194.9514247	197.5423233	300.4928302	445.1571206
IL-21	11.10125539	10.74811338	0.193582848	0.368729235	1.27733948	60505	Q9ES17	1829.34499	2527.639488	2289.934713	1330.28587	2090.120533	1826.524416
Leptin	12.38308273	12.14189159	0.192270122	0.368729235	1.181968132	16846	P41160	5698.233012	5828.021412	4587.026915	4406.964769	4653.922399	4497.525209
TremL1	15.85315669	16.04950979	0.195609364	0.369074272	0.872753958	71326	Q8K558	68746.51181	50161.05212	60143.34361	66484.62712	66877.75748	70166.43835
DLL4	11.45588631	11.89813715	0.19960834	0.3730997	0.735985451	54485	Q9JI71	3490.073109	2235.979476	2837.410943	3171.607211	4512.611833	3881.82676

GM-CSF	12.01301651	12.24696616	0.2063273	0.378582202	0.850303833	12983	P26955	4288.345197	4687.81746	3509.593931	5399.772852	4635.678462	4585.164407
MadCAM-1	10.68098274	10.23679564	0.205931336	0.378582202	1.360547286	17123	Q61826	1921.033155	1010.245273	2275.518509	915.4428599	1328.279795	1441.201186
Periostin	17.00539786	17.52856933	0.209588844	0.381070626	0.695840491	50706	Q62009	239987.6408	84787.48518	111911.0816	197162.5278	167180.2655	205049.7003
Dkk-1	14.57093018	15.01221323	0.221436935	0.398985468	0.736479332	13380	O54908	40354.11568	18590.22042	19214.63093	28532.41043	29851.87013	42367.16734
Renin 1	15.47916744	14.97416737	0.243285735	0.434438813	1.419123417	19701	P06281	62114.33932	30459.41567	50366.04023	31355.74629	22576.9161	47097.35862
TARC	12.60875157	12.263173	0.26230654	0.464259364	1.270660462	20295	Q9WUZ6	9232.165583	4821.974133	5471.323139	3862.7161	6343.469547	4844.607317
DAN	9.437977416	9.901513238	0.273672401	0.479361256	0.72520671	74108	Q8VDG3	1251.760225	418.6596674	633.7134684	1021.390985	832.5705322	1025.592523
EDAR	3.443106208	6.481876702	0.280426335	0.479361256	0.121685528	13608	Q9R187	0	1285.57391	0	55.6310393	44.77841204	274.4245767
IL-5	12.57659149	12.77667597	0.278249795	0.479361256	0.870499587	16191	P04401	6456.166542	7093.949332	4974.108539	7235.826457	7353.09956	6491.477813
VEGF-B	8.415508045	7.940341516	0.276587699	0.479361256	1.390078666	22340	P49766	364.5174744	291.5167347	371.3078472	185.5191321	235.2705934	335.2868294
ANGPTL3	12.06635994	11.6893126	0.300991828	0.501964563	1.298681216	30924	Q9R182	3692.974332	4236.843583	5038.298091	2217.955561	3540.430314	4582.253335
OX40 Ligand	9.425052423	8.670570319	0.301178738	0.501964563	1.687025879	22164	P43488	378.2069317	2213.36223	385.8526157	329.0652531	325.3812405	627.0297835
RAGE	9.507940867	10.12735774	0.299385638	0.501964563	0.650933976	26448	Q9WVS4	1639.365225	329.3337067	711.2504786	1299.321433	1072.605205	1001.351498
MFG-E8	10.06414353	9.678801887	0.315098675	0.520824255	1.306169066	17304	P21956	1618.867131	807.2824047	936.0974041	670.6469477	690.0731661	1185.216593
MDC	14.56100333	14.16277097	0.321573389	0.522285268	1.317892197	20299	O88430	42618.6069	23597.79109	14039.97015	15321.67159	17621.71391	22847.06195
RANTES	12.54299517	12.33372667	0.321688828	0.522285268	1.156101851	20304	P30882	6094.68694	5882.706227	5925.246475	4262.917808	6608.006624	4880.126287
VEGF R2	7.298348267	4.775195237	0.323816866	0.522285268	5.748370417	16542	P35918	129.5332366	89.66115676	328.551186	139.7534368	0	144.8720198
IL-6	13.74035206	13.87868147	0.341843271	0.542608366	0.908570636	16193	P08505	14541.45593	14555.87125	12106.89268	14132.76416	16097.23317	15018.7331
Persephin	7.722824441	7.36022367	0.34144846	0.542608366	1.285741637	19197	O70300	215.7671003	198.1983465	217.3364911	140.3998449	124.170591	249.6069222
Leptin R	13.00095469	13.26249881	0.350178209	0.551461746	0.834194599	16847	P48356	9631.692719	5846.003949	9779.271436	9308.240727	10099.08099	10091.31422
SDF-1a	12.09077669	12.32349579	0.361583288	0.564973887	0.851029416	20315	P40224	5921.542154	4260.606796	3287.348414	4865.444274	4537.154912	6096.258069
Gas 1	14.97411804	15.2108123	0.371616799	0.576150077	0.848687739	14451	Q01721	43118.34397	24426.24456	31653.06141	35062.64771	34103.48903	45609.37468
MIP-3b	7.894151841	7.226943205	0.376051681	0.578541047	1.587997494	24047	O70460	124.3149123	284.4846842	375.3084019	217.6832658	47.68585869	314.7633055
VEGF-D	9.490618512	10.01064625	0.381065335	0.581779137	0.697358427	14205	P97946	739.8849967	367.739329	1361.737753	845.3921739	1342.622026	964.3073984
Clusterin	13.26049537	13.05630549	0.387356041	0.582490288	1.152039263	12759	Q06890	12509.07859	8148.464538	9267.970102	7765.123371	8552.548395	9302.966738
Limitin	11.35500843	11.51512486	0.384706071	0.582490288	0.894952844	15964	Q61716	2898.165867	2309.966745	2681.425435	3021.428923	2575.207759	3219.008208
IL-12p70	11.07807861	10.92867016	0.390434712	0.582738375	1.109114613	16159	P43431	2327.990897	2016.196408	2149.729999	1903.869499	1629.831033	2382.956866
C5a	17.04978017	16.92181578	0.41783272	0.608597674	1.09275077	15139	P06684	157890.3132	116902.0502	135300.2088	130288.4044	127846.0872	114899.6422
Chordin	12.6526329	12.91331574	0.417660141	0.608597674	0.834692756	12667	Q9Z0E2	8178.594771	4776.344201	6830.998434	6877.782459	7023.088626	9500.334067
Fetuin A	13.68341725	13.44972935	0.419932395	0.608597674	1.175836848	11625	P29699	11878.57135	11480.29772	16693.1901	8880.296039	12627.6405	12486.71443
TECK	2.351549634	4.964029838	0.418543915	0.608597674	0.163517823	20300	O35903	0	0	131.9416082	130.3158782	0	230.551927
IFNg	12.70050667	12.81889711	0.427676004	0.615361157	0.921214841	15978	P01580	6973.974337	7258.898156	5823.079091	6894.372307	7943.515645	6885.403779
MIP-2	8.356279117	8.871458809	0.443190401	0.633129144	0.699705777	20310	P10889	452.7948449	144.9152652	530.5103329	810.7787044	238.6290441	527.1393314
Dtk	8.212569817	8.569592685	0.450481632	0.638981038	0.780774117	22174	P55144	450.2346096	377.9101278	151.6690324	329.9139795	339.1575046	486.2122231
CD27	12.8026904	12.94918439	0.464389648	0.654069927	0.90344333	21940	P41272	7782.885202	6074.457415	7711.694068	6997.669726	7108.067162	9940.47087
gp130	10.22774417	9.54045906	0.475296708	0.661055013	1.610250472	16195	Q00560	3347.065858	432.7330566	1186.290079	591.0755903	547.1348357	1271.414144
Osteoactivin	12.28697869	11.59328905	0.475959609	0.661055013	1.617414715	93695	Q99P91	12201.73979	1985.264894	5148.355902	1989.958002	3099.846518	4776.94215
HAI-1	12.67825662	12.82382951	0.484517556	0.668300078	0.904020323	20732	Q9R097	7420.068935	5271.022648	7196.188416	6642.728689	7044.050529	8141.84228
E-Cadherin	16.31810903	16.41610851	0.493374846	0.675855953	0.93432768	12550	P09803	89319.91698	75100.06693	81304.81148	86208.9954	87700.38135	88441.12469
IGF-1	17.9685882	18.07624281	0.499867329	0.680091604	0.928095641	16000	P05017	304222.3674	218643.9382	253698.779	269791.793	266548.3753	293538.043
Flt-3L	15.70654191	15.86315844	0.518724637	0.684551708	0.897126587	14256	P49772	56383.14301	53523.52082	50664.33991	54633.02506	62790.64839	61728.74302
Galectin-7	10.27421785	10.51486518	0.523663834	0.684551708	0.846365467	16858	O54974	2048.327923	937.430989	986.4826347	1224.814117	1911.181454	1335.33622
HGF R	12.47910495	12.29295093	0.5192651	0.684551708	1.137726681	17295	P16056	6569.880335	4311.157376	6567.051745	3674.676016	5794.942324	5930.706008
IL-3	12.24541841	12.38194441	0.523682057	0.684551708	0.90970709	16187	P01586	4790.191555	6198.293829	3853.153404	5675.681145	4723.989691	5668.106911
MBL-2	18.66562786	18.57359932	0.511121868	0.684551708	1.065867824	17195	P41317	428974.6978	381729.0725	439088.0028	357748.4128	398041.3349	416986.8237
P-Cadherin	12.34386747	11.80116355	0.518186703	0.684551708	1.45670013	12560	P10287	11923.74674	4073.029224	2890.677194	2170.886474	5153.494837	4058.657611
IGFBP-6	18.09592016	17.83444774	0.53074171	0.689274949	1.198701486	16012	P47880	143536.4583	360741.7469	424698.203	217011.013	242588.311	242523.567
Adiponectin	15.61749298	15.69711264	0.53847903	0.690357731	0.94630709	11450	Q60994	52695.21531	49210.52589	48994.39615	60438.64264	48787.40943	50846.15984
I-TAC	5.561577543	7.357241993	0.53684244	0.690357731	0.288038897	56066	Q9JHH5	181.7088611	0	575.5611569	80.55030455	295.2166921	181.4804992
CRP	15.91587241	15.98937475	0.557599196	0.710317447	0.950328144	12944	P14847	66631.7753	57206.29549	61989.57861	67232.39204	60754.70958	67400.89636
B7-1	12.02861912	11.75241501	0.566651202	0.717280002	1.211004399	12519	Q00609	5160.344997	3673.858886	3844.226493	1583.060332	4531.472654	5718.807444
IL-7	10.90010408	10.73956038	0.588247867	0.738580928	1.117708281	16196	P10168	1655.060181	2517.034722	1672.543563	1493.445027	2162.831874	1544.56042
JAM-A	15.44101759	15.25893077	0.590864743	0.738580928	1.134523758	16456	O88792	67063.37354	32839.22379	39968.65423	31981.45188	43002.0622	43829.50245
BAFF R	11.35441439	11.26954108	0.631692603	0.74756521	1.060594604	72049	Q9D8D0	3047.208135	2291.199243	2567.98143	2493.061644	2266.377852	2659.594105
BTC	12.69475421	12.82741435	0.621556003	0.74756521	0.912148018	12223	Q05928	8446.946186	5528.232481	6237.632851	5921.771021	6836.213673	9481.118207
GITR	9.351440607	9.177311189	0.625060747	0.74756521	1.128283346	21936	O35714	561.9925142	533.3567749	925.5277594	675.7990407	425.0746674	671.9648711
IL-17E	9.181117748	9.304328386	0.630143613	0.74756521	0.918142101	140806	Q8VHH8	575.1651755	475.1701723	711.9618885	535.8010673	631.272583	743.6086557
IL-17F	9.408679079	9.597385735	0.611190872	0.74756521	0.877391932	257630	Q7TNI7	423.7411246	807.5075472	913.2721196	608.3735462	651.558721	1167.960303
KC	14.31959702	14.18190467	0.63043278	0.74756521	1.100143981	14825	P12850	29658.22778	18134.66022	15891.47272	19273.17552	17982.96873	18520.44024
Lymphotactin	11.70873014	11.82366101	0.626008572	0.74756521	0.923426556	16963	P47993	3826.029565	2789.897771	3510.002998	3829.27024	3518.824433	3531.477858
MMP-2	14.33193314	14.51270951	0.61406592	0.74756521	0.882228112	17390	P33434	32989.37875	13599.70988	19545.83458	21247.82294	21510.13683	27942.73139
TRAIL	3.024097713	4.72859308	0.617413567	0.74756521	0.30682855	22035	P50592	0	0	537.3098964	114.70712	0	160.0589928
Chemerin	13.31367856	13.39821791	0.642714461	0.75613466	0.943085617	71660	Q9DD06	12951.8218	8684.902313	9380.460796	11224.32774	11299.29981	9918.903324
Endoglin	11.58786742	11.77215139	0.668825162	0.777703677	0.880085763	13805	Q63961	5700.278834	2079.768209	2457.617952	3663.726678	2539.892545	4593.985634
Nope	13.76565974	14.07589819	0.666472878	0.777703677	0.806508449	56741	Q9EQS9	28553.04038	6635.394814	14256.0506	14908.48056	13178.58176	26207.28295
IL-1 R4	9.671088637	9.859763801	0.681499181	0.787860324	0.877411083	17082	P14719	654.250935	500.9142833	1646.502593	689.4172413	994.740077	1165.799805
MIP-3a	10.154317	9.927090429	0.687495778	0.790225032	1.170582465	20297	O89093	1147.129517	1193.376597	1078.269344	907.5420095	1555.226091	651.5867642
CD36	11.00938997	11.18984069	0.703009537	0.803439471	0.882427267	12491	Q08857	3827.980804	1338.31074	1707.068581	2614.074461	2216.24899	2197.540965
Neprilysin	12.35950748	12.26878184	0.713342733	0.810616742	1.064905668	17380	Q61391	6072.379532	4313.922865	5536.885729	4717.339755	4056.302851	6276.509757
NOV	9.325276824	9.09203301	0.723936195	0.818007	1.175474966	18133	Q64299	945.1520296	262.2638146	1058.770998	570.4733411	373.8804948	757.6389781
Cystatin C	15.03625954	14.96318979	0.741423486	0.833060096	1.051952636	13010	P21460	43745.00156	26056.15059	33282.58846	29590.86433	31773.94003	34660.69565
sFRP-3	7.216123529	7.139046176	0.747591718	0.835298009	1.054878875	20378	P97401	177.5760803	119.1549583	152.1947635	151.1302198	127.568944	142.1691836
Eotaxin-2	13.34409266	13.1980586	0.754725565	0.838583961	1.106523478	56221	Q9JKC0	8966.454602	17367.02851	7218.327677	8535.32758	8395.647981	11577.63986
IL-9	12.64078928	12.6072436	0.76931653	0.850073514	1.023524534	16198	P15247	6851.548264	6191.20916	6137.644834	6142.317876	6272.323496	6302.398808
Eotaxin	15.03329282	14.95997298	0.787097	0.855546087	1.052135009	20292	P48298	43385.18097	36782.31	23626.29404	28270.14603	34704.0652	32995.32129
IL-23	9.760909763	9.839212272	0.779564941	0.855546087	0.947171444	16160	P43432	1153.777403	599.628253	940.6203529	1002.246521	705.7440632	1082.987442
IL-7 Ra	10.53985417	10.42318093	0.7871024	0.855546087	1.084231809	16197	P16872	2011.340019	1285.934823	1273.027639	783.9383153	2148.337339	1533.370433
Epigen	15.35199654	15.40315614	0.797315371	0.857328356	0.965160246	71920	Q924X1	51006.53976	35341.12469	40578.55871	44521.48895	41823.92269	43692.96872
Resistin	18.55873931	18.52854643	0.796662988	0.857328356	1.021148642	57264	Q99P87	411639.7576	361799.0281	386565.9146	380118.1739	360373.8988	394702.0147
CD40L	10.81341152	10.87954147	0.831408101	0.877090506	0.95519689	21947	P27548	2149.647193	1168.238881	2316.454657	1769.882266	1576.115614	2393.152148
IGFBP-3	17.86895373	17.89667752	0.827345125	0.877090506	0.9809668	16009	P47878	255592.595	230567.6593	232766.8272	232162.9628	246530.6283	253887.3735
IL-3 Rb	12.76662704	12.80629014	0.833235981	0.877090506	0.972882115	12984	P26954	7730.072465	5936.543665	7370.680257	6526.978545	6639.439285	8476.286547
L-Selectin	16.96213646	17.01783662	0.822421661	0.877090506	0.962127393	20343	P18337	181595.3895	97108.95516	118021.4508	123355.1107	130616.6622	145034.821
CD27L	8.907323979	8.823598289	0.8536046	0.893826806	1.059751266	21948	O55237	244.7406611	465.6153196	964.3372854	413.817539	409.860064	544.6978261
IL-2 Ra	13.3001659	13.35497415	0.863109365	0.899072255	0.962722393	16184	P01590	16008.23058	7887.77586	8124.808303	8784.615482	10277.46465	12735.2915
MMP-3	15.55408523	15.50903141	0.871014542	0.902605743	1.031721671	17392	P28862	71407.47721	34709.13151	44928.60484	41451.56748	48328.37036	50615.06734
MIP-1g	18.46689644	18.44887821	0.881782544	0.909054169	1.012567604	20308	P51670	373696.5571	363903.042	349752.5481	344351.6355	373539.0441	356168.4004
AR	10.19776814	10.16394276	0.895530389	0.918492707	1.023722986	11839	P31955	865.3330519	1360.954012	1371.950786	1193.971687	863.2285554	1461.071816
Endocan	11.55892798	11.61450218	0.917744771	0.936474256	0.9622114	71690	Q9QYY7	3894.338085	2018.589241	3489.952303	2159.147814	4520.951652	3154.958199
CD30L	12.19657669	12.21068658	0.931857164	0.946047882	0.990267435	21949	P32972	4690.784664	5438.96706	4051.023342	5275.992516	4737.474339	4258.158239
ALK-1	10.9939178	11.00364908	0.964752217	0.974497189	0.993277484	11482	Q61288	2397.674133	1633.081564	2162.974291	1833.623054	1870.722769	2519.559401
CT-1	11.90699877	11.90427393	0.988568333	0.993536013	1.001890497	13019	Q60753	4277.349368	3336.502748	3965.372363	3336.550033	3743.797169	4504.826914
Fractalkine	0	0	1	1	1	20312	O35188	0	0	0	0	0	0
