# Supplementary material for: LCN2 secreted by tissue-infiltrating neutrophils induces the ferroptosis and wasting of adipose and muscle tissues in lung cancer cachexia
Source: J Hematol Oncol. 2023 Mar 27;16:30. doi: 10.1186/s13045-023-01429-1 (PMC10044814; doi:10.1186/s13045-023-01429-1)
Supplement: Supplementary file 4 — Additional file 4. Table S5: Clinical characteristics of patients in this study. [file 13045_2023_1429_MOESM4_ESM.pdf]

Table S5 Clinical characteristics of patients in this study

Case NO	Age	Gender	Height (m)	Body weight (kg)	BMI (kg/m^2)	ALB (g/L)	HGB (g/L)	CRP (mg/mL)	IL-6 (pg/mL)	Tumor type	LCN2 (ng/mL)	Cancer stage	Main tumor site	Cachexia (Yes or No)
1	67	M	1.78	61	19.25	40.6	146	32.02	18.79	LUAD	97.23	IV	lung,bone,liver,adrenal gland	Yes
2	70	M	1.68	42	14.88	37	123	83.42	224.59	ESCA	145.75	IV	esophagus	Yes
3	49	M	1.78	70	22.09	37.4	88	23.85	18.49	SCLC	121.41	IV	lung,liver,lymph gland	Yes
4	69	M	1.68	61	21.61	28.2	112	75.9	18.36	STAD	126.87	IV	stomach,liver,lymph gland	Yes
5	82	M	1.65	44.5	16.34	40.6	108	4.72	18.14	COAD	118.70	IV	colon,abdominal cavity	Yes
6	55	M	1.65	47	17.26	34.1	73	107.93	276.23	STAD	141.85	IV	stomach,liver,lymph gland	Yes
7	43	M	1.73	45	15.03	32.6	53	15.89	17.02	ESCA	85.13	IV	esophagus	Yes
8	42	F	1.65	45	16.53	32.2	86	77.31	18.17	BRCA	74.89	IV	breast,liver,lung,bone,lymph gland	Yes
9	53	M	1.7	65	22.49	33.2	83	28.34	17.04	COAD	86.70	IV	rectum,pelvic cavity,mesentery,peritoneum,lymph gland	Yes
10	56	M	1.7	41	14.19	29.1	78	66.54	18.59	STAD	198.21	IV	stomach,abdominal cavity,pelvic cavity	Yes
11	49	M	1.7	40	13.84	34.2	91	69.53	545.54	ESCA	76.12	IV	esophagus,lymph gland	Yes
12	64	M	1.7	49	16.95	36	97	25.78	19.63	SCLC	206.81	Extensive	lung,bone	Yes
13	54	M	1.69	62.5	21.88	30.4	115	11.56	16.98	CHOL	138.68	IV	bile duct,bone,lung,abdominal cavity，peritoneum	Yes
14	52	F	1.61	57	21.99	39.9	100	21.45	18.87	READ	71.53	pT3N2aM1,IV	rectum,liver	Yes
15	51	F	1.56	50	20.54	46.3	80	121.78	16.53	READ	1139.10	IV	colon,abdominal cavity,pelvic cavity	Yes
16	47	F	1.6	40	15.62	32.8	72	11.80	16.50	OV	113.99	IIIC	ovary,liver,abdominal cavity，peritoneum	Yes
17	58	F	1.5	41.5	18.44	29.2	90	66.84	20.77	CESC	182.10	IV	cervix,abdominal cavity,pelvic cavity	Yes
18	68	M	1.61	46	17.75	30.5	99	42.97	17.08	PAAD	173.46	IV	pancreas,abdominal cavity，peritoneum	Yes
19	53	M	1.72	50	16.90	41.1	92	34.10	13.55	ESCA	130.24	pT2N1M0,IIB	esophagus	Yes
20	56	F	1.63	55	20.70	38.8	101	1.98	7.66	OV	32.196	III	ovary,peritoneum, abdominal cavity	Yes
21	74	F	1.74	50	16.51	29.9	91	51.6	13.01	STAD	99.93	IV	stomach,abdominal cavity	Yes
22	58	M	1.6	45	17.58	44.9	114	0.18	14.85	ESCA	16.57	IV	esophagus.lung,brain,lymph gland	Yes
23	53	M	1.72	60	20.28	38	126	0.97	9.74	READ	57.03	T3N1cM1,IV	rectum,liver	Yes
24	69	F	1.65	45	16.53	38.8	113	0.5	9.80	STAD	87.99	IV	stomach,abdominal cavity	Yes
25	54	M	1.6	50.5	19.73	45.2	109	19.7	9.74	COAD	70.05	IV	colon,adrenal gland,pelvic cavity,abdominal cavity	Yes
26	68	F	1.5	44	19.55	33.2	115	60.78	9.32	LUAD	142.02	IV	lung,bone,liver,brain,peritoneum,abdominal cavity	Yes
27	57	F	1.62	38	14.48	30.2	83	16.99	13.34	STAD	62.23	IV	stomach,abdominal cavity,pelvic cavity	Yes
28	52	M	1.65	62	22.77	37.5	106	49.58	11.76	ESCA	80.69	T3N2M0G2,IV	esophagus,lymph gland	Yes
29	70	M	1.73	54	18.04	35	128	6.51	11.31	ESCA	48.09	IV	esophagus,lymph gland，lung	Yes
30	64	F	1.55	65	27.05	38.7	83	1.78	9.07	STAD	234.80	IV	stomach,abdominal cavity	Yes
31	66	M	1.78	58	18.31	25.1	76	18.67305388	14.41828909	LUSC	332.91	IV	lung	Yes
32	63	M	1.7	57.5	19.90	41	110	6.559848015	2.530500107	SCLC	135.06	IV	lung,stomach	Yes
33	76	M	1.62	52	19.81	35.1	109	25.87247587	1.947404	LUSC	196.58	IV	liver,lung,bone	Yes

Table S5 Clinical characteristics of patients in this study (Continue)

Case NO	Age	Gender	Height (m)	Body weight (kg)	BMI (kg/m^2)	ALB (g/L)	HGB (g/L)	CRP (mg/mL)	IL-6 (pg/mL)	Tumor type	LCN2 (ng/mL)	Cancer stage	Main tumor site	Cachexia (Yes or No)
1	60	M	1.75	76	24.82	44.2	144	2.46	13.20	SCLC	66.10	ED	lung, liver	No
2	58	M	1.7	54	18.69	41.7	125	3.07	13.94	LUAD	72.45	cT1N1M1,IVB	lung,brain	No
3	75	F	1.58	40	16.02	43.1	126	5.00	11.80	LUAD	69.42	cT4N2M1C,IVB	lung,bone,brain	No
4	56	M	1.7	64	22.15	36.3	108	7.22	12.24	SCLC	70.02	ED	lung,bone	No
5	72	M	1.61	62	23.92	46.2	140	14.07	11.88	LUSC	51.98	cT3N3M0,IIIC	lung,mediastinum	No
6	66	M	1.7	60	20.76	34.2	85	1.91	13.90	LUSC	190.49	T1N2M1,IV	lung	No
7	56	M	1.56	60	24.65	37.7	114	1.33	15.34	SCLC	68.81	ED	lung	No
8	60	M	1.7	59	20.42	36.1	71	11.40	14.17	LUAD	106.03	cT4N2M0,IIIb	lung	No
9	54	F	1.52	43	18.61	41.7	97	1.56	14.07	LUSC	74.89	cT2N1M1a,IVa	lung,bone	No
10	64	M	1.68	63	22.32	34.8	103	37.51	11.12	LUSC	62.52	cT3N3M1a,IVa	lung,adrenal gland	No
11	76	F	1.6	69	26.95	43.7	146	0.28	11.98	LUAD	66.70	T1N2M1C,IVB	lung,bone,brain	No
12	65	M	1.79	64	19.97	38.1	116	2.61	13.73	LUAD	59.57	cT4N2M1,IVa	lung	No
13	81	M	1.7	55	19.03	34.7	135	36.95	11.05	LUSC	137.63	IV	lung	No
14	74	M	1.73	64	21.38	38.4	122	0.13	14.25	LUAD	114.67	IV	lung,bone	No
15	69	M	1.67	67	24.02	39.2	126	10.51	13.50	LUAD	84.19	cT1N1M1a,IVA	lung,pleura,peritoneum	No
16	67	F	1.52	75	32.46	47.9	109	2.06	12.43	LUSC	67.30	T4N3M1,IV	lung	No
17	48	F	1.53	66	28.19	41.5	110	2.08	11.55	LUAD	70.63	T3N1M1b,IV	lung,bone	No
18	49	M	1.62	74	28.20	42	44.6	38.67	11.13	SCLC	77.66	ED	lung,bone	No
19	64	M	1.7	50	17.30	39	99	2.53	11.25	SCLC	198.60	ED	lung	No
20	70	M	1.61	61	23.53	37.8	145	1.44	13.52	LCNEC	85.13	pT2bN0M0	lung	No
21	68	M	1.78	79	24.93	36.6	128	0.30	11.59	LUSC	97.56	IV	lung	No
22	67	F	1.62	68	25.91	44.8	130	1.69	13.39	LUAD	69.11	pT2bN0M0,IIa	lung	No
23	75	M	1.77	65	20.75	37.2	120	4.75	13.63	LUSC	107.68	T3N2M1,IV	lung,bone,liver	No
24	45	M	1.71	65	22.23	41.3	132	1.66	11.35	LUAD	120.06	T4N2M1a,IV	lung	No
25	74	M	1.7	60.6	20.97	40.6	104	18.49	11.06	LUSC	64.91	cT4N3M1,IVB	lung	No
26	77	F	1.55	55	22.89	31.6	91	22.20	11.50	LUAD	138.68	cT1N3M1a,IVA	lung,brain	No
27	63	M	1.78	92	29.04	37.7	124	14.36	11.00	LUAD	70.02	IV	lung,brain	No
28	77	M	1.78	65	20.52	39.4	137	6.33	11.02	LUAD	76.12	T4N2M1,IV	lung	No
29	70	M	1.83	69	20.60	38.5	106	14.18	11.82	LUAD	79.83	cT4N3M1,IVB	lung,peritoneum	No
30	63	F	1.59	69	27.29	45.9	107	1.13	15.06	LUAD	67.30	T1bN2M0,IIIA	lung	No
31	79	F	1.55	58	24.14	38.7	135	0.39	3.68	LUAD	58.90	CT1N3M1,IV	lung,brain	No
32	66	F	1.48	48	21.91	39.9	123	12.87	3.07	LUAD	50.00	IV	lung	No
33	81	M	1.73	65	21.72	40.5	98	3.61	2.87	LUAD	51.49	IV	lung	No
34	71	M	1.6	52	20.31	36.9	106	10.97	3.33	LUSC	55.45	T3N3Mx	lung	No
