# Supplementary material for: LCN2 secreted by tissue-infiltrating neutrophils induces the ferroptosis and wasting of adipose and muscle tissues in lung cancer cachexia
Source: J Hematol Oncol. 2023 Mar 27;16:30. doi: 10.1186/s13045-023-01429-1 (PMC10044814; doi:10.1186/s13045-023-01429-1)
Supplement: Supplementary file 5 — Additional file 5. Table S6: Differential expressed genes in eWAT. [file 13045_2023_1429_MOESM5_ESM.pdf]

Table S6 Differential expressed genes in eWAT

gene_id	eWAT_cachexia_1	eWAT_cachexia_2	eWAT_cachexia_3	eWAT_control_1	eWAT_control_2	eWAT_control_3	eWAT_cachexia	eWAT_control	log2FoldChange	p value	padj	gene_name	gene_chr	gene_start	gene_end	gene_strand	gene_length	gene_biotype	gene_description	tf_fa
ENSMUSG0000000226	1890.967	1987.514822	2753.796036	34.45324342	30.6170999	19.46050868	2210.759286	28.17695067	6.288043226	1.95E-113	3.64E-109	Zim1	7	6671269	6696450	-	8086	protein_coding	zinc finger, imprinted 1 [Source:MGI Symbol;Acc:MGI:1341879]	zf-C2
ENSMUSG0000003180	37679.04568	32689.19449	34690.00994	5156.789209	5438.979649	5964.645909	35019.4167	5520.138256	2.665425713	4.25E-79	3.98E-75	Slc27a1	8	71568882	71587302	+	5835	protein_coding	solute carrier family 27 (fatty acid transporter), member 1 [Source:MGI Symbol;Acc:MGI:1347098]	-
ENSMUSG0000000226	14379.1656	11859.78638	21681.81479	832.0458287	1460.73196	1010.0004	15973.58892	1100.926063	3.859127255	8.11E-43	5.06E-39	Peg3	7	6703892	6730431	-	13324	protein_coding	paternally expressed 3 [Source:MGI Symbol;Acc:MGI:104748]	zf-C2
ENSMUSG0000005902	36.07568203	54.7698838	49.15497995	516.7986514	787.1557618	788.1506014	46.66684859	697.3683382	-3.903266524	1.47E-42	6.87E-39	9630013D21Ri	4	109651529	109657853	-	3142	processed_transcrip	RIKEN cDNA 9630013D21 gene [Source:MGI Symbol;Acc:MGI:2442649]	-
ENSMUSG0000009491	45.09460254	12.63920395	23.46033134	742.4673958	688.3909235	857.2354072	27.06471261	762.6979088	-4.809798959	5.65E-41	2.11E-37	D430019H16R	12	105453856	105493095	-	5786	protein_coding	RIKEN cDNA D430019H16 gene [Source:MGI Symbol;Acc:MGI:2443127]	-
ENSMUSG0000003800	3041.380416	3397.839329	4464.165906	409.1322657	586.6631399	435.9153943	3634.461884	477.2369333	2.929403059	5.61E-39	1.75E-35	Acer2	4	86874396	86934822	+	7159	protein_coding	alkaline ceramidase 2 [Source:MGI Symbol;Acc:MGI:1920932]	-
ENSMUSG0000003896	4752.971107	5503.320055	6378.975807	1218.783486	1274.066415	1234.769275	5545.08899	1242.539726	2.157891644	1.10E-38	2.95E-35	Pdk2	11	95026258	95041354	-	3163	protein_coding	pyruvate dehydrogenase kinase, isoenzyme 2 [Source:MGI Symbol;Acc:MGI:1343087]	-
ENSMUSG0000002474	501.0511393	353.8977107	299.3985142	2640.841108	2793.069629	3121.465592	384.7824547	2851.79211	-2.888717677	2.56E-38	5.97E-35	Aldh1a7	19	20692953	20727562	-	2066	protein_coding	aldehyde dehydrogenase family 1, subfamily A7 [Source:MGI Symbol;Acc:MGI:1347050]	-
ENSMUSG0000004911	107.2249438	34.75781087	98.3099599	2754.536812	1546.657369	2279.798591	80.09757153	2193.664257	-4.775345397	2.87E-38	5.97E-35	Oxtr	6	112473683	112489943	-	4790	protein_coding	oxytocin receptor [Source:MGI Symbol;Acc:MGI:109147]	-
ENSMUSG0000003837	2176.566149	2284.536115	2358.321879	9570.249692	9253.277707	12333.09737	2273.141381	10385.54159	-2.191847207	3.81E-38	7.13E-35	Trp53inp2	2	155381059	155389850	+	5257	protein_coding	transformation related protein 53 inducible nuclear protein 2 [Source:MGI Symbol;Acc:MGI:1915978]	-
ENSMUSG0000002135	4759.985823	2615.261951	5996.907554	149.0102778	307.1586473	204.3353411	4457.38511	220.1680887	4.340580653	1.02E-37	1.74E-34	Irf4	13	30749226	30766976	+	4903	protein_coding	interferon regulatory factor 4 [Source:MGI Symbol;Acc:MGI:1096873]	IRF
ENSMUSG0000004165	990.0770513	524.5269641	498.2527513	6764.894346	10845.3669	9967.672544	670.9522555	9192.644597	-3.775719258	7.59E-37	1.18E-33	Pnpla3	15	84167837	84187236	+	1548	protein_coding	patatin-like phospholipase domain containing 3 [Source:MGI Symbol;Acc:MGI:2151796]	-
ENSMUSG0000000014	243.5108537	219.0795352	197.7370784	1402.247007	1061.722013	1219.200869	220.1091558	1227.723296	-2.479094674	2.16E-36	3.11E-33	Axin2	11	108920349	108950783	+	5506	protein_coding	axin 2 [Source:MGI Symbol;Acc:MGI:1270862]	-
ENSMUSG0000002937	323.679036	403.4012595	358.6079219	15.50395954	24.69120959	31.13681388	361.8960725	23.77732767	3.936988064	4.27E-36	5.50E-33	Rassf6	5	90603076	90640657	-	4260	protein_coding	Ras association (RalGDS/AF-6) domain family member 6 [Source:MGI Symbol;Acc:MGI:1920496]	-
ENSMUSG0000005010	1362.859099	1578.847227	1173.016567	243.7566972	237.0356121	180.9827307	1371.574298	220.59168	2.635790149	4.41E-36	5.50E-33	Grrp1	4	134251110	134254106	-	1275	protein_coding	glycine/arginine rich protein 1 [Source:MGI Symbol;Acc:MGI:1919940]	-
ENSMUSG0000002718	709.4884133	705.6888874	774.1909342	148.1489467	162.9619833	158.6031457	729.7894116	156.5713586	2.221295394	3.95E-35	4.62E-32	Pamr1	2	102550012	102643041	+	6046	protein_coding	peptidase domain containing associated with muscle regeneration 1 [Source:MGI Symbol;Acc:MGI:2445082]	-
ENSMUSG0000003134	245.5150583	204.3337972	217.8459339	1049.962593	1005.426055	931.1853401	222.5649298	995.5246627	-2.160781696	2.82E-34	3.10E-31	Nsdhl	X	72918557	72958514	+	2853	protein_coding	NAD(P) dependent steroid dehydrogenase-like [Source:MGI Symbol;Acc:MGI:1099438]	-
ENSMUSG0000007086	263.5528993	218.0262682	135.1761949	1794.152651	2010.852109	2778.960639	205.5851208	2194.655133	-3.414669976	3.42E-34	3.55E-31	Trabd2b	4	114406724	114615098	+	6617	protein_coding	TraB domain containing 2B [Source:MGI Symbol;Acc:MGI:3650152]	-
ENSMUSG0000004425	1551.254327	2162.357143	1978.487943	376.4016844	366.4175504	294.8267064	1897.366471	345.8819804	2.455134075	1.70E-33	1.62E-30	Ctla2a	13	60934155	60936625	-	2108	protein_coding	cytotoxic T lymphocyte-associated protein 2 alpha [Source:MGI Symbol;Acc:MGI:88554]	-
ENSMUSG0000002699	4556.559061	4240.452926	5088.657583	1397.940352	1397.522463	1371.965862	4628.556524	1389.142892	1.736302561	1.73E-33	1.62E-30	Pkp4	2	59160850	59355208	+	5707	protein_coding	plakophilin 4 [Source:MGI Symbol;Acc:MGI:109281]	-
ENSMUSG0000009134	353.7421044	279.115754	188.7998094	11981.97673	3291.832063	7253.904609	273.8858892	7509.237801	-4.776535121	2.87E-33	2.45E-30	Col6a5	9	105856078	105960643	-	9413	protein_coding	collagen, type VI, alpha 5 [Source:MGI Symbol;Acc:MGI:3648134]	-
ENSMUSG0000004848	4687.834459	8303.956997	7787.712846	651.1663007	975.7966031	800.799932	6926.501434	809.2542786	3.097730576	2.89E-33	2.45E-30	Depp1	6	116650609	116652794	+	1358	protein_coding	DEPP1 autophagy regulator [Source:MGI Symbol;Acc:MGI:1918730]	-
ENSMUSG0000003219	131.2753985	210.6533992	186.5654921	1305.777926	1045.919638	1180.279851	176.1647633	1177.325805	-2.74182495	3.60E-33	2.93E-30	Ldlr	9	21723483	21749919	+	9622	protein_coding	low density lipoprotein receptor [Source:MGI Symbol;Acc:MGI:96765]	-
ENSMUSG0000002549	462.9712527	408.6675945	385.4197292	55.98652056	61.23419979	43.78614452	419.0195255	53.66895496	2.964682393	4.50E-33	3.51E-30	Ifitm1	7	140967221	140969825	+	1094	protein_coding	interferon induced transmembrane protein 1 [Source:MGI Symbol;Acc:MGI:1915963]	-
ENSMUSG0000009069	1554.260634	2191.848619	1799.742561	304.0498732	390.1211116	321.0983931	1848.617271	338.423126	2.450104071	7.29E-33	5.46E-30	Apold1	6	134981718	134986836	+	3567	protein_coding	apolipoprotein L domain containing 1 [Source:MGI Symbol;Acc:MGI:2685921]	-
ENSMUSG0000004180	804.6881297	826.8145919	508.307179	4012.941528	5024.167328	5330.233326	713.2699669	4789.114061	-2.746761221	1.67E-32	1.20E-29	Phlda3	1	135766119	135769136	+	1559	protein_coding	pleckstrin homology like domain, family A, member 3 [Source:MGI Symbol;Acc:MGI:1351485]	-
ENSMUSG0000002808	1785.74626	1968.556016	2008.651226	601.2090977	566.9101723	585.7613111	1920.984501	584.6268604	1.715962417	1.16E-31	8.05E-29	Fmo5	3	97628804	97655282	+	5675	protein_coding	flavin containing monooxygenase 5 [Source:MGI Symbol;Acc:MGI:1310004]	-
ENSMUSG0000002662	3353.034224	3072.379828	2338.213024	15597.84463	14162.87782	20808.1489	2921.209025	16856.29045	-2.528545973	1.49E-31	9.95E-29	Lpgat1	1	191717834	191784255	+	7610	protein_coding	lysophosphatidylglycerol acyltransferase 1 [Source:MGI Symbol;Acc:MGI:2446186]	-
ENSMUSG0000005239	823.728073	931.0880246	1047.8948	106.8050546	163.9496317	165.4143237	934.2369658	145.38967	2.686127611	5.44E-30	3.51E-27	Acot4	12	84038379	84048601	+	6674	protein_coding	acyl-CoA thioesterase 4 [Source:MGI Symbol;Acc:MGI:2159621]	-
ENSMUSG0000002927	281.5907403	140.0845105	192.1512853	3812.251385	1693.816978	3131.195846	204.6088453	2879.08807	-3.814110025	6.79E-30	4.23E-27	Sult1e1	5	87575969	87591594	-	1242	protein_coding	sulfotransferase family 1E, member 1 [Source:MGI Symbol;Acc:MGI:98431]	-
ENSMUSG0000005605	883.8542097	826.8145919	3170.496207	38.75989885	46.41947404	43.78614452	1627.055003	42.9885058	5.24281804	1.50E-29	9.03E-27	S100a8	3	90668978	90670035	+	694	protein_coding	S100 calcium binding protein A8 (calgranulin A) [Source:MGI Symbol;Acc:MGI:88244]	-
ENSMUSG0000008684	852.7890391	845.7733979	1137.267491	175.7115415	117.5301577	156.6570948	945.2766425	149.9662647	2.654182036	3.28E-29	1.92E-26	2410006H16Ri	11	62602877	62604807	+	462	processed_transcrip	RIKEN cDNA 2410006H16 gene [Source:MGI Symbol;Acc:MGI:1916471]	-
ENSMUSG0000005455	92.19340963	156.9367824	118.4188153	1030.151978	737.7733427	1032.379985	122.5163358	933.4351021	-2.930528635	4.25E-29	2.41E-26	Adam12	7	133883199	134232146	-	10573	protein_coding	a disintegrin and metallopeptidase domain 12 (meltrin alpha) [Source:MGI Symbol;Acc:MGI:105378]	-
ENSMUSG0000002604	6840.350154	2989.171735	4160.298758	45505.84391	36503.48426	45594.02578	4663.273549	42534.45132	-3.189164954	4.90E-29	2.69E-26	Col3a1	1	45311538	45349706	+	9816	protein_coding	collagen, type III, alpha 1 [Source:MGI Symbol;Acc:MGI:88453]	-
ENSMUSG0000002265	331.6958542	376.0163176	472.5581027	5.167986514	24.69120959	8.757228904	393.4234248	12.87214167	4.950852794	8.72E-29	4.66E-26	Retnlg	16	48872621	48874496	+	593	protein_coding	resistin like gamma [Source:MGI Symbol;Acc:MGI:2667763]	-
ENSMUSG0000003629	129.2711939	133.7649085	113.9501808	604.6544221	822.7111037	615.9250996	125.6620944	681.0968751	-2.437268711	2.07E-27	1.08E-24	Slc2a13	15	91267696	91573261	-	8367	protein_coding	solute carrier family 2 (facilitated glucose transporter), member 13 [Source:MGI Symbol;Acc:MGI:2146030]	-
ENSMUSG0000001997	9403.727783	5216.831432	7049.270988	1232.564783	1384.683034	1212.38969	7223.276734	1276.545836	2.500486666	2.77E-27	1.40E-24	Sgk1	10	21882184	21999903	+	4903	protein_coding	serum/glucocorticoid regulated kinase 1 [Source:MGI Symbol;Acc:MGI:1340062]	-
ENSMUSG0000002579	704.4779019	954.2598985	432.3403918	4755.408924	5539.719784	5813.826967	697.0260641	5369.651892	-2.945207747	3.99E-27	1.96E-24	Slc25a10	11	120491840	120499187	+	3455	protein_coding	solute carrier family 25 (mitochondrial carrier, dicarboxylate transporter), member 10 [Source:MGI Symbol;Acc:MGI:1353497]	-
ENSMUSG0000003770	21625.36717	19227.38901	29224.8699	1167.103621	2687.391252	2801.340224	23359.20869	2218.611699	3.396422325	1.07E-26	5.15E-24	Fam13a	6	58932090	59024549	-	10505	protein_coding	family with sequence similarity 13, member A [Source:MGI Symbol;Acc:MGI:1889842]	-
ENSMUSG0000003706	1316.762394	1497.745668	1660.097732	4595.201342	5570.336884	5106.437476	1491.535265	5090.658567	-1.771207679	1.10E-26	5.15E-24	Cavin3	7	105480083	105482300	-	1662	protein_coding	caveolae associated 3 [Source:MGI Symbol;Acc:MGI:1923422]	-
ENSMUSG0000007501	282.5928426	357.0575117	289.3440865	47.37320971	36.5429902	35.02891562	309.6648136	39.64837184	2.961080737	1.38E-26	6.29E-24	Fjx1	2	102449366	102452499	-	3134	protein_coding	four jointed box 1 [Source:MGI Symbol;Acc:MGI:1341907]	-
ENSMUSG0000003074	3466.271782	3841.264735	1084.761035	35901.14098	48926.12563	49496.83079	2797.432517	44774.69913	-4.000421315	5.16E-26	2.30E-23	Dgat2	7	99153658	99182719	-	3083	protein_coding	diacylglycerol O-acyltransferase 2 [Source:MGI Symbol;Acc:MGI:1915050]	-
ENSMUSG0000007223	1030.161142	559.2847749	1661.214891	14091.37656	10389.07335	12931.50801	1083.553603	12470.65264	-3.524903335	5.32E-26	2.32E-23	Tuba1a	15	98949837	98953703	-	1803	protein_coding	tubulin, alpha 1A [Source:MGI Symbol;Acc:MGI:98869]	-
ENSMUSG0000003074	3332.992179	4146.712164	4273.13178	1097.335803	1222.708699	1019.730655	3917.612041	1113.258386	1.815168166	6.59E-26	2.80E-23	Il4ra	7	125552120	125579474	+	6002	protein_coding	interleukin 4 receptor, alpha [Source:MGI Symbol;Acc:MGI:105367]	-
ENSMUSG0000003485	5225.963383	5434.8577	6709.654763	1107.671776	1532.830292	1511.108499	5790.158615	1383.870189	2.065142055	1.24E-25	5.18E-23	Fam214a	9	74952884	75032468	+	6637	protein_coding	family with sequence similarity 214, member A [Source:MGI Symbol;Acc:MGI:2387648]	-
ENSMUSG0000004268	860.8058573	1190.191706	1091.463987	4567.638747	3599.978359	4340.66646	1047.487183	4169.427855	-1.993177263	1.51E-25	6.13E-23	Mapk6	9	75369062	75410005	-	6675	protein_coding	mitogen-activated protein kinase 6 [Source:MGI Symbol;Acc:MGI:1354946]	-
ENSMUSG0000002855	1461.065122	1495.639134	1051.246276	5479.788367	5362.930724	7061.245573	1335.983511	5967.988221	-2.159109735	1.60E-25	6.37E-23	Cdkn2c	4	109660876	109667189	-	2686	protein_coding	cyclin dependent kinase inhibitor 2C [Source:MGI Symbol;Acc:MGI:105388]	-
ENSMUSG0000002190	3551.450475	3012.343609	3400.630886	1013.786688	1169.375686	1119.952274	3321.47499	1101.038216	1.593226583	2.00E-25	7.81E-23	Sema3g	14	31217860	31230352	+	4540	protein_coding	sema domain, immunoglobulin domain (Ig), short basic domain, secreted, (semaphorin) 3G [Source:MGI Symbol;Acc:MGI:30412	-
ENSMUSG0000002222	1699.565465	1826.364971	1617.645704	483.206739	585.6754916	536.137014	1714.52538	535.0064149	1.680798678	2.13E-25	8.12E-23	Adcy4	14	55769057	55784095	-	4672	protein_coding	adenylate cyclase 4 [Source:MGI Symbol;Acc:MGI:99674]	-
ENSMUSG0000003512	334.7021611	344.4183077	427.8717573	49.09587188	59.25890302	66.1657295	368.9974087	58.17350147	2.667451892	2.43E-25	9.10E-23	Neil2	14	63182443	63193856	-	5017	protein_coding	nei like 2 (E. coli) [Source:MGI Symbol;Acc:MGI:2686058]	-
ENSMUSG0000005613	363.7631271	455.0113423	307.2186247	41.34389211	49.38241919	59.35455146	375.3310314	50.02695425	2.910516808	6.03E-25	2.21E-22	Unc93a2	17	7362815	7385464	-	2945	protein_coding	unc-93 homolog A2 [Source:MGI Symbol;Acc:MGI:3712668]	-
ENSMUSG0000002232	407.8556274	199.0674623	280.4068174	3265.306145	1841.964236	2496.783263	295.7766357	2534.684548	-3.098782751	8.85E-25	3.18E-22	Matn2	15	34306677	34436273	+	6263	protein_coding	matrilin 2 [Source:MGI Symbol;Acc:MGI:109613]	-
ENSMUSG0000009052	392.8240932	403.4012595	357.4907633	1200.695533	1339.251208	1538.353211	384.5720387	1359.433317	-1.821177636	1.23E-24	4.35E-22	Gypc	18	32528322	32560034	-	2331	protein_coding	glycophorin C [Source:MGI Symbol;Acc:MGI:1098566]	-
ENSMUSG0000002636	2783.84013	3012.343609	2152.76469	651.1663007	521.4783466	408.6706822	2649.649476	527.1051098	2.329084557	3.47E-24	1.20E-21	Rgs2	1	143999338	144004161	-	3627	protein_coding	regulator of G-protein signaling 2 [Source:MGI Symbol;Acc:MGI:1098271]	-
ENSMUSG0000006208	722.5157429	567.7109109	548.5248899	144.7036224	135.3078286	139.142637	612.9171812	139.7180293	2.133148914	5.12E-24	1.74E-21	Gm6055	14	48079375	48079752	-	378	processed_pseudog	predicted gene 6055 [Source:MGI Symbol;Acc:MGI:3648620]	-
ENSMUSG0000001769	199.4183534	182.2151903	130.7075603	803.6219029	774.3163329	972.0524083	170.780368	849.9968814	-2.313598459	5.31E-24	1.78E-21	Rhbdl3	11	80300912	80355955	+	4734	protein_coding	rhomboid like 3 [Source:MGI Symbol;Acc:MGI:2179276]	-
ENSMUSG0000002235	115.241762	147.4573795	124.0046085	732.1314228	536.2930724	613.9790487	128.90125	627.4678479	-2.284035211	6.13E-24	2.01E-21	Sqle	15	59315077	59331192	+	5546	protein_coding	squalene epoxidase [Source:MGI Symbol;Acc:MGI:109296]	-
ENSMUSG0000005054	124.2606825	174.8423214	151.9335744	658.0569494	847.4023133	1021.676705	150.3455261	842.378656	-2.486500955	8.25E-24	2.66E-21	Fam241a	3	127869058	127896324	-	2265	protein_coding	family with sequence similarity 241, member A [Source:MGI Symbol;Acc:MGI:1917867]	-
ENSMUSG0000002896	2742.753937	2861.726428	2277.886457	7600.385499	9378.709052	8318.394433	2627.455607	8432.496328	-1.682161032	9.93E-24	3.15E-21	Pgd	4	149149991	149166771	-	6447	protein_coding	phosphogluconate dehydrogenase [Source:MGI Symbol;Acc:MGI:97553]	-
ENSMUSG0000002658	915.9214827	1099.610744	1805.328355	175.7115415	187.6531929	126.4933064	1273.620194	163.2860136	2.962919408	1.31E-23	4.08E-21	Selp	1	164115264	164150026	+	4413	protein_coding	selectin, platelet [Source:MGI Symbol;Acc:MGI:98280]	-
ENSMUSG0000002731	565.1856851	630.9069307	414.4658537	37.03723668	80.98716747	56.43547516	536.8528232	58.1532931	3.211570935	1.64E-23	5.02E-21	Chac1	2	119351229	119354381	+	1637	protein_coding	ChaC, cation transport regulator 1 [Source:MGI Symbol;Acc:MGI:1916315]	-
ENSMUSG0000005825	119.2501712	102.1668986	97.19280126	421.1909009	537.2807208	513.757429	106.2032903	490.7430169	-2.206307233	1.00E-22	3.03E-20	Idi1	13	8885501	8892451	+	3505	protein_coding	isopentenyl-diphosphate delta isomerase [Source:MGI Symbol;Acc:MGI:2442264]	-
ENSMUSG0000000803	270.5676152	254.8906131	262.5322793	1752.808759	988.6360321	1316.503412	262.6635025	1352.649401	-2.364758389	1.62E-22	4.81E-20	Mid1ip1	X	10715013	10719690	+	2128	protein_coding	Mid1 interacting protein 1 (gastrulation specific G12-like (zebrafish)) [Source:MGI Symbol;Acc:MGI:1915291]	-
ENSMUSG0000000133	4.008409114	6.319601977	3.351475906	403.1029481	116.5425093	291.9076301	4.559828999	270.5176958	-5.88857023	3.40E-22	9.94E-20	Fndc5	4	129136999	129144593	+	2763	protein_coding	fibronectin type III domain containing 5 [Source:MGI Symbol;Acc:MGI:1917614]	-
ENSMUSG0000003492	101.2123301	45.29048083	55.85793176	507.3240094	564.9348755	516.6765053	67.45358091	529.6451301	-2.969536508	8.29E-22	2.39E-19	Dhcr24	4	106561038	106589113	+	4028	protein_coding	24-dehydrocholesterol reductase [Source:MGI Symbol;Acc:MGI:1922004]	-
ENSMUSG0000010097	394.8282978	492.9289542	530.6503517	109.3890479	83.95011262	77.8420347	472.8025346	90.39373173	2.383831948	8.65E-22	2.45E-19	Gm28875	12	104925501	104926689	-	1189	lincRNA	predicted gene 28875 [Source:MGI Symbol;Acc:MGI:5579581]	-
ENSMUSG0000007370	1578.311089	1529.343678	1226.640181	383.2923331	454.3182565	441.7535469	1444.764983	426.4547122	1.76112434	1.06E-21	2.97E-19	Klhl21	4	152008803	152017680	+	3986	protein_coding	kelch-like 21 [Source:MGI Symbol;Acc:MGI:1919288]	-
ENSMUSG0000000465	30666.33393	30989.22156	30422.46395	13235.21346	12741.6518	11038.00052	30692.67315	12338.28859	1.314730844	1.96E-21	5.40E-19	Aqp1	6	55336432	55348555	+	2627	protein_coding	aquaporin 1 [Source:MGI Symbol;Acc:MGI:103201]	-
ENSMUSG0000005294	95.19971647	67.40908775	98.3099599	776.0593081	425.6764534	825.1255678	86.97292137	675.6204431	-2.958064326	2.41E-21	6.53E-19	Glis3	19	28258851	28680077	-	8842	protein_coding	GLIS family zinc finger 3 [Source:MGI Symbol;Acc:MGI:2444289]	zf-C2
ENSMUSG0000002604	1450.041997	960.5795004	1135.033173	3976.765622	4172.814421	4534.298521	1181.88489	4227.959522	-1.838637327	4.06E-21	1.09E-18	Col5a2	1	45374321	45503282	-	9591	protein_coding	collagen, type V, alpha 2 [Source:MGI Symbol;Acc:MGI:88458]	-
ENSMUSG0000000090	1314.75819	1711.558869	1103.752732	243.7566972	313.0845376	180.0097052	1376.68993	245.61698	2.48697832	4.50E-21	1.19E-18	Mmp11	10	75923222	75936496	-	2778	protein_coding	matrix metallopeptidase 11 [Source:MGI Symbol;Acc:MGI:97008]	-
ENSMUSG0000001957	3385.101497	13892.59168	9972.875137	583.982476	774.3163329	728.7960499	9083.522771	695.6982863	3.706817904	7.58E-21	1.97E-18	Pdk4	6	5483351	5496309	-	3511	protein_coding	pyruvate dehydrogenase kinase, isoenzyme 4 [Source:MGI Symbol;Acc:MGI:1351481]	-
ENSMUSG0000008759	452.9502299	512.9410271	699.3413056	119.7250209	90.8636513	106.0597723	555.0775209	105.5494815	2.392719681	9.55E-21	2.45E-18	Epb41l4aos	18	33794892	33795986	+	430	lincRNA	erythrocyte membrane protein band 4.1 like 4a, opposite strand [Source:MGI Symbol;Acc:MGI:1916999]	-
ENSMUSG0000004141	3244.807178	3772.80238	2325.924279	12750.28406	10954.00822	14332.66644	3114.511279	12678.98564	-2.025282356	1.43E-20	3.60E-18	Pik3r1	13	101680563	101768217	-	10079	protein_coding	phosphoinositide-3-kinase regulatory subunit 1 [Source:MGI Symbol;Acc:MGI:97583]	-
ENSMUSG0000004267	3207.729394	2650.019762	3288.915022	1136.957033	1061.722013	1116.060173	3048.888059	1104.913073	1.464237575	1.53E-20	3.81E-18	Ypel3	7	126776955	126780514	+	1848	protein_coding	yippee like 3 [Source:MGI Symbol;Acc:MGI:1913340]	-
ENSMUSG0000003938	247.5192628	191.6945933	188.7998094	827.7391733	1389.621276	992.4859425	209.3378885	1069.948797	-2.352592948	1.61E-20	3.98E-18	Dusp10	1	184013302	184075636	+	3042	protein_coding	dual specificity phosphatase 10 [Source:MGI Symbol;Acc:MGI:1927070]	-
ENSMUSG0000002590	1972.137284	1571.474358	1914.809901	602.9317599	595.5519754	483.5936406	1819.473848	560.6924586	1.69798954	2.13E-20	5.17E-18	Sox17	1	4490931	4497354	-	4772	protein_coding	SRY (sex determining region Y)-box 17 [Source:MGI Symbol;Acc:MGI:107543]	HMC
ENSMUSG0000002833	2082.368535	2290.855717	1199.828374	8086.176232	11939.68131	13051.19014	1857.684209	11025.68256	-2.569133863	2.80E-20	6.71E-18	Col15a1	4	47208161	47313167	+	6349	protein_coding	collagen, type XV, alpha 1 [Source:MGI Symbol;Acc:MGI:88449]	-
ENSMUSG0000003192	3085.472916	3548.45651	2222.028525	10097.38432	12698.19527	14070.9208	2951.985984	12288.83346	-2.057486197	2.99E-20	7.08E-18	Cyb5b	8	107150640	107187471	+	4374	protein_coding	cytochrome b5 type B [Source:MGI Symbol;Acc:MGI:1913677]	-
ENSMUSG0000003196	286.6012517	239.0916081	288.2269279	1976.754841	987.6483837	1734.904348	271.3065959	1566.435858	-2.529725727	3.21E-20	7.50E-18	Bmper	9	23223076	23485200	+	4833	protein_coding	BMP-binding endothelial regulator [Source:MGI Symbol;Acc:MGI:1920480]	-
ENSMUSG0000004605	954.0013692	654.0788046	964.1079022	186.0475145	216.294996	208.2274428	857.3960253	203.5233178	2.075384528	3.25E-20	7.50E-18	Sbsn	7	30751471	30756134	+	3625	protein_coding	suprabasin [Source:MGI Symbol;Acc:MGI:2446326]	-
ENSMUSG0000004113	2527.301947	2971.266196	2900.143817	592.5957869	834.5628843	886.4261702	2799.570653	771.1949471	1.860560524	3.64E-20	8.30E-18	Cyyr1	16	85421533	85553397	-	8894	protein_coding	cysteine and tyrosine-rich protein 1 [Source:MGI Symbol;Acc:MGI:2152187]	-
ENSMUSG0000003821	72.15136406	70.56888874	50.27213858	311.801853	554.0707433	496.2429712	64.33079713	454.0385225	-2.816565331	4.26E-20	9.60E-18	Tlcd2	11	75461715	75470899	+	1936	protein_coding	TLC domain containing 2 [Source:MGI Symbol;Acc:MGI:1917141]	-
ENSMUSG0000002403	1433.006258	2401.448751	3224.119821	357.4524005	428.6393985	336.6668001	2352.858277	374.2528664	2.652327536	4.92E-20	1.10E-17	Abcg1	17	31057698	31115777	+	4066	protein_coding	ATP binding cassette subfamily G member 1 [Source:MGI Symbol;Acc:MGI:107704]	-
ENSMUSG0000002010	2698.661436	4951.408149	5258.465696	792.4245987	452.3429598	388.2371481	4302.845094	544.3349022	2.982062612	5.17E-20	1.13E-17	Ddit4	10	59949669	59951834	-	1755	protein_coding	DNA-damage-inducible transcript 4 [Source:MGI Symbol;Acc:MGI:1921997]	-
ENSMUSG0000002787	3056.41195	3764.376244	3838.557071	1000.866721	577.7743045	581.8692094	3553.115088	720.1700784	2.301963343	5.17E-20	1.13E-17	Hmgcs2	3	98280435	98310738	+	3290	protein_coding	3-hydroxy-3-methylglutaryl-Coenzyme A synthase 2 [Source:MGI Symbol;Acc:MGI:101939]	-
ENSMUSG0000005230	28433.65005	13605.04979	25327.10342	4209.325015	2172.826444	2362.505753	22455.26775	2914.885738	2.945427303	1.06E-19	2.29E-17	Hbb-bs	7	103826534	103828096	-	768	protein_coding	hemoglobin, beta adult s chain [Source:MGI Symbol;Acc:MGI:5474852]	-
ENSMUSG0000003180	1283.693019	1770.54182	1389.745342	444.4468402	473.0835758	434.9423689	1481.326727	450.8242616	1.716308021	1.12E-19	2.38E-17	B3gnt3	8	71690756	71701789	-	3055	protein_coding	UDP-GlcNAc:betaGal beta-1,3-N-acetylglucosaminyltransferase 3 [Source:MGI Symbol;Acc:MGI:2152535]	-
ENSMUSG0000005293	3105.514961	8090.143797	4798.196338	811.3738826	787.1557618	693.7671343	5331.285032	764.0989263	2.802573252	1.73E-19	3.64E-17	Fbxo31	8	121549440	121578806	-	5482	protein_coding	F-box protein 31 [Source:MGI Symbol;Acc:MGI:1354708]	-
ENSMUSG0000005981	3364.057349	4134.07296	4675.308888	1206.724851	1167.40039	1423.53621	4057.813066	1265.88715	1.680526671	2.25E-19	4.67E-17	Rgs3	4	62559847	62704001	+	10801	protein_coding	regulator of G-protein signaling 3 [Source:MGI Symbol;Acc:MGI:1354734]	-
ENSMUSG0000002683	453.9523322	361.2705797	331.7961147	1187.775567	1526.904401	1672.630721	382.3396755	1462.436896	-1.934571849	2.83E-19	5.81E-17	Olfm1	2	28192992	28230736	+	3553	protein_coding	olfactomedin 1 [Source:MGI Symbol;Acc:MGI:1860437]	-
ENSMUSG0000003092	619.2992082	677.2506785	895.9612254	161.068913	161.9743349	201.4162648	730.8370374	174.8198376	2.063809833	3.17E-19	6.45E-17	Rexo5	7	119794006	119848943	+	4782	protein_coding	RNA exonuclease 5 [Source:MGI Symbol;Acc:MGI:1919402]	-
ENSMUSG0000003144	3043.38462	2121.27973	3004.03957	804.4832339	865.1799842	791.0696777	2722.901307	820.2442986	1.731077864	4.94E-19	9.94E-17	Mcf2l	8	12873806	13020905	+	12760	protein_coding	mcf.2 transforming sequence-like [Source:MGI Symbol;Acc:MGI:103263]	-
ENSMUSG0000003839	62807.76241	65077.15462	62862.5164	12307.55988	21131.72482	17580.62354	63582.47781	17006.63608	1.90256059	5.20E-19	1.04E-16	Txnip	3	96557957	96561883	+	3123	protein_coding	thioredoxin interacting protein [Source:MGI Symbol;Acc:MGI:1889549]	-
ENSMUSG0000001821	1226.573189	981.6448404	1547.26471	4167.981123	4204.41917	4810.637745	1251.82758	4394.346012	-1.811797002	7.95E-19	1.57E-16	Pmp22	11	63128982	63159547	+	2354	protein_coding	peripheral myelin protein 22 [Source:MGI Symbol;Acc:MGI:97631]	-
ENSMUSG0000005862	23.04835241	23.17187391	21.22601407	181.7408591	154.0731479	241.3103076	22.48208013	192.3747715	-3.096014242	1.12E-18	2.18E-16	Gm17383	18	82338774	82339816	+	1041	pseudogene	predicted gene, 17383 [Source:MGI Symbol;Acc:MGI:4937017]	-
ENSMUSG0000002835	6.012613672	4.213067984	18.9916968	188.6315077	175.8014123	177.0906289	9.739126152	180.5078497	-4.230506348	1.21E-18	2.34E-16	Orm3	4	63356162	63359511	+	773	protein_coding	orosomucoid 3 [Source:MGI Symbol;Acc:MGI:97445]	-
ENSMUSG0000002865	1413.966315	1315.530478	1890.232411	470.2867727	443.4541243	471.9173354	1539.909735	461.8860775	1.736930138	1.68E-18	3.19E-16	Trit1	4	123016597	123054949	+	2087	protein_coding	tRNA isopentenyltransferase 1 [Source:MGI Symbol;Acc:MGI:1914216]	-
ENSMUSG0000002404	1785.74626	2659.499165	3583.844902	458.2281375	544.1942594	594.51854	2676.363442	532.3136457	2.330065322	1.69E-18	3.19E-16	Sik1	17	31844248	31855792	-	4511	protein_coding	salt inducible kinase 1 [Source:MGI Symbol;Acc:MGI:104754]	-
ENSMUSG0000002444	178.3742056	203.2805302	170.9252712	542.6385839	648.8849881	640.2507354	184.1933357	610.5914358	-1.728328389	2.07E-18	3.87E-16	Pcdh12	18	38267092	38284402	-	6055	protein_coding	protocadherin 12 [Source:MGI Symbol;Acc:MGI:1855700]	-
ENSMUSG0000002707	2538.325072	3351.495582	3545.861508	331.612468	722.9586169	608.1408961	3145.227387	554.237327	2.505251976	3.04E-18	5.64E-16	Slc43a1	2	84838850	84863594	+	5716	protein_coding	solute carrier family 43, member 1 [Source:MGI Symbol;Acc:MGI:1931352]	-
ENSMUSG0000002109	3985.360762	4664.919526	2899.026658	1180.023587	935.3030194	987.6208153	3849.768982	1034.315807	1.895929455	3.98E-18	7.30E-16	Clmn	12	104763117	104865076	-	12409	protein_coding	calmin [Source:MGI Symbol;Acc:MGI:2136957]	-
ENSMUSG0000003771	2270.763763	2354.051736	2223.145684	968.1361402	1055.796122	983.7287136	2282.653728	1002.553659	1.187215255	4.81E-18	8.73E-16	Cisd1	10	71330480	71344954	-	1084	protein_coding	CDGSH iron sulfur domain 1 [Source:MGI Symbol;Acc:MGI:1261855]	-
ENSMUSG0000006991	11183.46143	6105.788776	10509.11128	2254.103451	1353.078286	1386.561243	9266.120496	1664.580993	2.476601678	5.49E-18	9.87E-16	Hba-a1	11	32283511	32284465	+	812	protein_coding	hemoglobin alpha, adult chain 1 [Source:MGI Symbol;Acc:MGI:96015]	-
ENSMUSG0000003275	1597.351032	1262.867128	1333.88741	476.3160903	467.1576855	529.325836	1398.03519	490.9332039	1.510078964	6.78E-18	1.21E-15	Slc8b1	5	120511168	120534024	+	3463	protein_coding	solute carrier family 8 (sodium/lithium/calcium exchanger), member B1 [Source:MGI Symbol;Acc:MGI:2180781]	-
ENSMUSG0000003201	1219.558473	874.2116068	775.3080928	3114.573205	3864.668126	4544.028776	956.3593909	3841.090036	-2.005489488	7.28E-18	1.28E-15	Sc5d	9	42251595	42264256	-	4866	protein_coding	sterol-C5-desaturase [Source:MGI Symbol;Acc:MGI:1353611]	-
ENSMUSG0000004930	260.5465924	261.210215	132.9418776	1037.903958	1091.351464	1317.476437	218.232895	1148.91062	-2.394925498	8.24E-18	1.44E-15	Syt12	19	4445908	4477447	-	5539	protein_coding	synaptotagmin XII [Source:MGI Symbol;Acc:MGI:2159601]	-
ENSMUSG0000003654	1185.486996	1069.066001	1150.673394	3973.320298	2914.55038	3049.461709	1135.075464	3312.444129	-1.545234056	1.06E-17	1.83E-15	Adamts2	11	50602084	50807573	+	9336	protein_coding	a disintegrin-like and metallopeptidase (reprolysin type) with thrombospondin type 1 motif, 2 [Source:MGI Symbol;Acc:MGI:1347	-
ENSMUSG0000000409	1715.599101	1005.869981	2335.978706	7713.219871	9926.853905	10390.93861	1685.81593	9343.670795	-2.470636857	1.37E-17	2.34E-15	Col5a3	9	20770050	20815067	-	6809	protein_coding	collagen, type V, alpha 3 [Source:MGI Symbol;Acc:MGI:1858212]	-
ENSMUSG0000002618	178.3742056	189.5880593	189.916968	4.306655428	14.81472576	20.43353411	185.9597443	13.18497176	3.834108806	1.97E-17	3.35E-15	Cxcr2	1	74153989	74161246	+	3048	protein_coding	chemokine (C-X-C motif) receptor 2 [Source:MGI Symbol;Acc:MGI:105303]	-
ENSMUSG0000003135	650.3643788	512.9410271	637.8975807	1741.611455	1599.990382	2063.786945	600.4009955	1801.796261	-1.585406054	3.47E-17	5.85E-15	Zfp185	X	72987339	73031543	+	3707	protein_coding	zinc finger protein 185 [Source:MGI Symbol;Acc:MGI:108095]	-
ENSMUSG0000000580	89.1871028	145.3508455	450.21493	1.722662171	1.975296767	4.865127169	228.2509594	2.854362036	6.332835903	3.59E-17	6.00E-15	Mmp8	9	7558456	7568485	+	2425	protein_coding	matrix metallopeptidase 8 [Source:MGI Symbol;Acc:MGI:1202395]	-
ENSMUSG0000002089	2128.46524	1892.720792	2714.695484	757.1100242	639.9961527	750.2026094	2245.293838	715.7695954	1.649020479	4.37E-17	7.24E-15	Per1	11	69095217	69109960	+	5631	protein_coding	period circadian clock 1 [Source:MGI Symbol;Acc:MGI:1098283]	-
ENSMUSG0000003331	1831.842965	1855.856447	1300.372651	557.2812124	503.7006757	505.0002001	1662.690688	521.9940294	1.671356656	5.39E-17	8.84E-15	Gstt2	10	75831114	75837424	-	4156	protein_coding	glutathione S-transferase, theta 2 [Source:MGI Symbol;Acc:MGI:106188]	-
ENSMUSG0000007031	354.7442066	299.1278269	458.0350404	71.4904801	89.87600292	69.0848058	370.6356913	76.81709627	2.271154306	5.65E-17	9.20E-15	4930581F22Ri	9	35116728	35130922	+	5954	processed_transcrip	RIKEN cDNA 4930581F22 gene [Source:MGI Symbol;Acc:MGI:1926184]	-
ENSMUSG0000011034	207.4351717	115.8593696	222.3145684	747.6353823	1103.203245	1177.360775	181.8697032	1009.399801	-2.472561384	5.74E-17	9.26E-15	Gm45716	11	90187683	90251255	+	2187	protein_coding	predicted gene 45716 [Source:MGI Symbol;Acc:MGI:5804831]	-
ENSMUSG0000000208	85.17869368	97.95383064	62.56088357	322.9991571	388.1458148	414.5088348	81.89780263	375.2179356	-2.19355199	6.21E-17	9.89E-15	Bbc3	7	16308393	16318205	+	2124	protein_coding	BCL2 binding component 3 [Source:MGI Symbol;Acc:MGI:2181667]	-
ENSMUSG0000004782	67.14085267	101.1136316	26.81180725	684.758213	504.6883241	711.2815921	65.02209718	633.5760431	-3.282177293	6.24E-17	9.89E-15	Angptl8	9	21835510	21837347	+	865	protein_coding	angiopoietin-like 8 [Source:MGI Symbol;Acc:MGI:3643534]	-
ENSMUSG0000000083	2279.782684	1889.560991	1702.54976	4938.011114	6506.627552	6597.112441	1957.297812	6013.917036	-1.619228898	7.74E-17	1.22E-14	Fmr1	X	68678541	68717963	+	8966	protein_coding	fragile X mental retardation 1 [Source:MGI Symbol;Acc:MGI:95564]	-
ENSMUSG0000009073	4446.32781	4034.012595	4840.648366	1571.0679	911.5994582	1082.977308	4440.329591	1188.548222	1.901028639	8.45E-17	1.32E-14	Rps27	3	90212522	90213651	-	1043	protein_coding	ribosomal protein S27 [Source:MGI Symbol;Acc:MGI:1888676]	-
ENSMUSG0000004529	912.9151758	1113.303215	537.3533035	3895.8005	3539.731807	4742.525964	854.5238981	4059.352757	-2.24776378	9.47E-17	1.46E-14	Insig1	5	28071363	28078662	+	3093	protein_coding	insulin induced gene 1 [Source:MGI Symbol;Acc:MGI:1916289]	-
ENSMUSG0000004122	129.2711939	201.1739963	187.6826507	758.8326864	3303.683844	1943.131791	172.7092803	2001.882774	-3.535176133	1.05E-16	1.62E-14	Elovl6	3	129532355	129638495	+	6431	protein_coding	ELOVL family member 6, elongation of long chain fatty acids (yeast) [Source:MGI Symbol;Acc:MGI:2156528]	-
ENSMUSG0000002596	271.5697175	236.9850741	224.5488857	1264.434034	742.7115846	973.0254338	244.3678924	993.3903507	-2.023227563	1.09E-16	1.66E-14	Adam23	1	63445891	63596276	+	17907	protein_coding	a disintegrin and metallopeptidase domain 23 [Source:MGI Symbol;Acc:MGI:1345162]	-
ENSMUSG0000002966	4891.261222	2796.423875	3441.965755	23029.40924	12933.25559	17194.33244	3709.883617	17718.99909	-2.255819974	1.17E-16	1.77E-14	Col1a2	6	4504814	4541544	+	12426	protein_coding	collagen, type I, alpha 2 [Source:MGI Symbol;Acc:MGI:88468]	-
ENSMUSG0000003952	896.8815394	1111.196681	821.1115969	214.4714403	313.0845376	252.0135873	943.0632724	259.8565218	1.860829009	1.39E-16	2.07E-14	Atp8b1	18	64528979	64661000	-	6440	protein_coding	ATPase, class I, type 8B, member 1 [Source:MGI Symbol;Acc:MGI:1859665]	-
ENSMUSG0000004573	1026.152733	739.3934313	1528.273013	204.1354673	225.1838315	235.472155	1097.939726	221.5971513	2.308899173	1.61E-16	2.38E-14	Adrb2	18	62177817	62179959	-	2143	protein_coding	adrenergic receptor, beta 2 [Source:MGI Symbol;Acc:MGI:87938]	-
ENSMUSG0000004942	2046.292853	3652.729942	1666.800684	468.5641106	442.4664759	283.1504012	2455.274493	398.0603292	2.624560343	1.68E-16	2.48E-14	Chchd10	10	75933130	75937747	+	1575	protein_coding	coiled-coil-helix-coiled-coil-helix domain containing 10 [Source:MGI Symbol;Acc:MGI:2143558]	-
ENSMUSG0000002118	4632.718834	4433.200787	5655.057011	1968.141531	1642.459262	1460.511176	4906.992211	1690.370656	1.537237893	1.72E-16	2.51E-14	Fbln5	12	101746565	101819055	-	6911	protein_coding	fibulin 5 [Source:MGI Symbol;Acc:MGI:1346091]	-
ENSMUSG0000009130	3.006306836	3.159800988	0	85.27177747	147.1596092	211.1465191	2.055369275	147.8593019	-6.137871421	2.73E-16	3.96E-14	Gm8895	12	103867846	103881511	-	1223	unprocessed_pseud	predicted gene 8895 [Source:MGI Symbol;Acc:MGI:3643891]	-
ENSMUSG0000001730	8749.354995	6930.496834	10114.75428	2808.80067	3057.759396	3198.334601	8598.202037	3021.631556	1.508735109	3.51E-16	5.05E-14	Cd300lg	11	102041509	102055620	+	2737	protein_coding	CD300 molecule like family member G [Source:MGI Symbol;Acc:MGI:1289168]	-
ENSMUSG0000002683	1594.344725	764.6718392	1064.652179	4861.352647	4893.797741	4999.404679	1141.222915	4918.185022	-2.107329708	3.78E-16	5.40E-14	Col5a1	2	27886425	28039514	+	8490	protein_coding	collagen, type V, alpha 1 [Source:MGI Symbol;Acc:MGI:88457]	-
ENSMUSG0000002679	3203.720985	3807.560191	4342.395615	1520.249366	1212.832215	1299.96198	3784.55893	1344.347854	1.4928958	5.75E-16	8.15E-14	Fam129b	2	32876114	32925254	+	4672	protein_coding	family with sequence similarity 129, member B [Source:MGI Symbol;Acc:MGI:2442910]	-
ENSMUSG0000007380	94.19761419	42.13067984	53.62361449	484.0680701	324.9363183	529.325836	63.31730284	446.1100748	-2.814114156	6.05E-16	8.49E-14	Cdkn2b	4	89306299	89311039	-	1390	protein_coding	cyclin dependent kinase inhibitor 2B [Source:MGI Symbol;Acc:MGI:104737]	-
ENSMUSG0000002190	6341.303219	12509.65211	6890.634462	1936.27228	1957.519097	1974.268605	8580.529931	1956.019994	2.133149733	6.08E-16	8.49E-14	Galnt15	14	32028989	32062197	+	6326	protein_coding	polypeptide N-acetylgalactosaminyltransferase 15 [Source:MGI Symbol;Acc:MGI:1926004]	-
ENSMUSG0000002024	2424.085412	1110.143414	1769.579278	9805.393078	6886.87218	9108.491086	1767.936035	8600.252115	-2.282241988	6.33E-16	8.77E-14	Col6a2	10	76595762	76623630	-	8326	protein_coding	collagen, type VI, alpha 2 [Source:MGI Symbol;Acc:MGI:88460]	-
ENSMUSG0000001983	422.8871616	322.2997008	374.2481428	960.3841604	1086.413222	1128.709503	373.1450017	1058.502295	-1.503680834	6.47E-16	8.91E-14	Rab32	10	10545002	10558265	-	2339	protein_coding	RAB32, member RAS oncogene family [Source:MGI Symbol;Acc:MGI:1915094]	-
ENSMUSG0000007394	4530.504402	1565.154756	2849.871678	504.7400162	263.7021185	227.6879515	2981.843612	332.043362	3.166012284	6.60E-16	9.01E-14	Hbb-bt	7	103812524	103813996	-	703	protein_coding	hemoglobin, beta adult t chain [Source:MGI Symbol;Acc:MGI:5474850]	-
ENSMUSG0000003056	2627.512175	2034.911836	2235.434429	5627.937313	5445.893188	6350.937006	2299.286147	5808.255836	-1.336812882	6.88E-16	9.31E-14	Ctsc	7	88278085	88310888	+	5808	protein_coding	cathepsin C [Source:MGI Symbol;Acc:MGI:109553]	-
ENSMUSG0000005648	323.679036	124.2855055	205.5571889	1252.375398	1470.608443	1147.196986	217.8405768	1290.060276	-2.565224973	6.92E-16	9.31E-14	Cd248	19	5068078	5070637	+	2560	protein_coding	CD248 antigen, endosialin [Source:MGI Symbol;Acc:MGI:1917695]	-
ENSMUSG0000005705	610.2802877	757.2989702	578.688173	173.1275482	168.8878736	222.8228243	648.7558103	188.2794154	1.785407424	7.04E-16	9.41E-14	Inca1	11	70688361	70700155	-	1377	protein_coding	inhibitor of CDK, cyclin A1 interacting protein 1 [Source:MGI Symbol;Acc:MGI:2144284]	-
ENSMUSG0000002847	1782.739954	1802.13983	2103.60971	797.5925853	790.118707	829.990695	1896.163165	805.9006624	1.234285443	7.10E-16	9.42E-14	Gne	4	44034075	44084177	-	6358	protein_coding	glucosamine (UDP-N-acetyl)-2-epimerase/N-acetylmannosamine kinase [Source:MGI Symbol;Acc:MGI:1354951]	-
ENSMUSG0000008693	279.5865357	420.2535314	354.1392874	83.5491153	77.03657393	55.46244973	351.3264515	72.01604632	2.284324402	7.18E-16	9.47E-14	4930481A15Ri	19	5406740	5422847	+	2240	antisense	RIKEN cDNA 4930481A15 gene [Source:MGI Symbol;Acc:MGI:1922181]	-
ENSMUSG0000000654	5.010511393	7.372868973	11.17158635	160.2075819	107.6536738	101.1946451	7.851655573	123.0186336	-3.982689489	7.54E-16	9.85E-14	Prkag3	1	74738922	74749221	-	3682	protein_coding	protein kinase, AMP-activated, gamma 3 non-catalytic subunit [Source:MGI Symbol;Acc:MGI:1891343]	-
ENSMUSG0000002688	62.13034127	22.11860692	65.91235948	543.499915	341.7263408	413.5358094	50.05376922	432.9206884	-3.113533301	7.58E-16	9.85E-14	Grb14	2	64912476	65024987	-	7185	protein_coding	growth factor receptor bound protein 14 [Source:MGI Symbol;Acc:MGI:1355324]	-
ENSMUSG0000003845	1472.088247	2055.977176	1909.224108	619.2970505	679.502088	587.707362	1812.429844	628.8355002	1.527118111	9.54E-16	1.23E-13	Dennd2a	6	39462378	39557867	-	7229	protein_coding	DENN/MADD domain containing 2A [Source:MGI Symbol;Acc:MGI:2444961]	-
ENSMUSG0000003683	1326.783417	1628.350776	3081.123516	348.8390897	390.1211116	372.6687411	2012.085903	370.5429808	2.440932037	1.14E-15	1.46E-13	Pnpla7	2	24976033	25054057	+	7496	protein_coding	patatin-like phospholipase domain containing 7 [Source:MGI Symbol;Acc:MGI:2385325]	-
ENSMUSG0000002102	2714.695073	4319.447951	3994.95928	1097.335803	1202.955731	1149.143037	3676.367434	1149.811524	1.676880082	1.19E-15	1.51E-13	Nfkbia	12	55489410	55492647	-	1610	protein_coding	nuclear factor of kappa light polypeptide gene enhancer in B cells inhibitor, alpha [Source:MGI Symbol;Acc:MGI:104741]	-
ENSMUSG0000004408	32.06727292	36.86434486	25.69464861	255.8153324	153.0854995	246.1754347	31.5420888	218.3587555	-2.790198795	1.27E-15	1.60E-13	Efcab8	2	153779931	153844751	+	5693	protein_coding	EF-hand calcium binding domain 8 [Source:MGI Symbol;Acc:MGI:3644206]	-
ENSMUSG0000003826	1754.68109	3178.759794	2806.302492	662.3636048	736.7856943	647.0619135	2579.914459	682.0704042	1.919304449	1.57E-15	1.97E-13	Sema7a	9	57940112	57962865	+	3298	protein_coding	sema domain, immunoglobulin domain (Ig), and GPI membrane anchor, (semaphorin) 7A [Source:MGI Symbol;Acc:MGI:1306826]	-
ENSMUSG0000003568	6764.190381	6064.711364	3467.660404	18675.3806	32156.84373	32006.69862	5432.187383	27612.97431	-2.345680241	1.61E-15	2.00E-13	Thrsp	7	97412938	97417730	-	2189	protein_coding	thyroid hormone responsive [Source:MGI Symbol;Acc:MGI:109126]	-
ENSMUSG0000009078	174.3657965	129.5518405	766.3708238	9.474641942	14.81472576	7.78420347	356.7628203	10.69119039	5.062410446	1.74E-15	2.16E-13	Gm17116	14	64111200	64114641	-	1013	antisense	predicted gene 17116 [Source:MGI Symbol;Acc:MGI:4937943]	-
ENSMUSG0000008990	143.3006258	137.9779765	157.5193676	24.1172704	10.86413222	16.54143237	146.26599	17.17427833	3.079254541	1.82E-15	2.25E-13	Gm8113	14	43925390	43933411	+	2721	protein_coding	predicted gene 8113 [Source:MGI Symbol;Acc:MGI:3648791]	-
ENSMUSG0000003707	64481.27322	127382.1105	124310.71	375525.7107	603872.913	631134.4601	105391.3646	536844.3613	-2.34874952	1.90E-15	2.32E-13	Scd1	19	44394451	44407709	-	4836	protein_coding	stearoyl-Coenzyme A desaturase 1 [Source:MGI Symbol;Acc:MGI:98239]	-
ENSMUSG0000003350	104.218637	102.1668986	78.20110447	6.890648685	3.950593535	2.919076301	94.86221335	4.58677284	4.351248957	1.96E-15	2.38E-13	Asprv1	6	86628164	86629710	+	1547	protein_coding	aspartic peptidase, retroviral-like 1 [Source:MGI Symbol;Acc:MGI:1915105]	-
ENSMUSG0000004812	1391.920065	703.5823534	776.4252515	4156.783819	4109.604925	3979.674024	957.3092233	4082.020923	-2.091881384	2.29E-15	2.75E-13	Col6a3	1	90765923	90843971	-	11963	protein_coding	collagen, type VI, alpha 3 [Source:MGI Symbol;Acc:MGI:88461]	-
ENSMUSG0000000765	21438.97615	11753.40641	16852.33801	54132.07474	59674.70299	68116.64549	16681.57352	60641.14107	-1.862029368	2.29E-15	2.75E-13	Cav1	6	17306335	17341452	+	3444	protein_coding	caveolin 1, caveolae protein [Source:MGI Symbol;Acc:MGI:102709]	-
ENSMUSG0000002527	1394.926372	582.4566488	998.7398199	201.551474	166.9125769	159.5761711	992.0409469	176.0134073	2.494225212	2.44E-15	2.90E-13	Alas2	X	150547375	150570638	+	2403	protein_coding	aminolevulinic acid synthase 2, erythroid [Source:MGI Symbol;Acc:MGI:87990]	-
ENSMUSG0000004577	2.004204557	5.26633498	0	93.02375724	102.7154319	167.3603746	2.423513179	121.0331879	-5.623904586	2.63E-15	3.12E-13	Lrtm1	14	29018208	29033645	+	7939	protein_coding	leucine-rich repeats and transmembrane domains 1 [Source:MGI Symbol;Acc:MGI:2442106]	-
ENSMUSG0000003015	4000.392296	3139.788915	4351.332884	1516.804042	1436.04075	1298.015929	3830.504699	1416.953573	1.434598863	2.85E-15	3.35E-13	Clec2d	6	129180615	129186534	+	1213	protein_coding	C-type lectin domain family 2, member d [Source:MGI Symbol;Acc:MGI:2135589]	-
ENSMUSG0000003263	1917.021659	2220.286828	1927.098646	851.8564436	928.3894807	818.3143898	2021.469044	866.1867714	1.222750151	2.87E-15	3.36E-13	Flcn	11	59791408	59810016	-	4322	protein_coding	folliculin [Source:MGI Symbol;Acc:MGI:2442184]	-
ENSMUSG0000003701	41.08619342	10.53266996	4.468634541	435.8335293	309.1339441	393.1022752	18.69583264	379.3565829	-4.331424744	3.02E-15	3.51E-13	Apln	X	48025146	48034853	-	3149	protein_coding	apelin [Source:MGI Symbol;Acc:MGI:1353624]	-
ENSMUSG0000011061	95.19971647	123.2322385	180.9796989	13.78129737	10.86413222	9.730254338	133.137218	11.45856131	3.531667454	3.19E-15	3.68E-13	Gm39822	11	51898966	51909624	+	2284	lincRNA	predicted gene, 39822 [Source:MGI Symbol;Acc:MGI:5622707]	-
ENSMUSG0000002865	1432.004156	2978.639065	1972.90215	467.7027795	312.0968893	491.3778441	2127.848457	423.7258376	2.32787603	4.58E-15	5.26E-13	Mycl	4	122995652	123002485	+	4038	protein_coding	v-myc avian myelocytomatosis viral oncogene lung carcinoma derived [Source:MGI Symbol;Acc:MGI:96799]	bHLI
ENSMUSG0000008643	24.05045469	2.106533992	17.87453816	211.8874471	297.2821635	232.5530787	14.67717561	247.2408964	-4.071250918	4.99E-15	5.69E-13	B430119L08Ri	12	103895897	103898784	+	1994	antisense	RIKEN cDNA B430119L08 gene [Source:MGI Symbol;Acc:MGI:2441857]	-
ENSMUSG0000005415	1109.327222	1302.891274	786.4796792	226.5300755	322.9610215	273.4201469	1066.232725	274.303748	1.959703567	5.15E-15	5.84E-13	Syne3	12	104929933	105009809	-	5548	protein_coding	spectrin repeat containing, nuclear envelope family member 3 [Source:MGI Symbol;Acc:MGI:2442408]	-
ENSMUSG0000005461	9401.723578	8354.513813	9732.68603	4421.212462	4435.528891	4394.182859	9162.974474	4416.974738	1.052746269	5.35E-15	6.02E-13	Mettl7a1	15	100304140	100328662	+	2595	protein_coding	methyltransferase like 7A1 [Source:MGI Symbol;Acc:MGI:1916523]	-
ENSMUSG0000002720	1623.405691	1133.315288	1192.008264	3489.252228	4877.007719	4158.710704	1316.243081	4174.990217	-1.66507394	5.38E-15	6.02E-13	Fbn1	2	125300594	125507993	-	12128	protein_coding	fibrillin 1 [Source:MGI Symbol;Acc:MGI:95489]	-
ENSMUSG0000004857	883.8542097	1955.916812	3279.977753	294.5752313	219.2579412	158.6031457	2039.916258	224.1454394	3.185242592	6.10E-15	6.79E-13	Tmem252	19	24674008	24679661	+	2462	protein_coding	transmembrane protein 252 [Source:MGI Symbol;Acc:MGI:3583948]	-
ENSMUSG0000002015	149.3132395	86.36789368	83.78689764	441.0015158	491.8488951	586.7343366	106.4893436	506.5282492	-2.247283753	6.71E-15	7.42E-13	Gamt	10	80258151	80261012	-	1444	protein_coding	guanidinoacetate methyltransferase [Source:MGI Symbol;Acc:MGI:1098221]	-
ENSMUSG0000002810	1421.983133	1168.073099	1970.667833	460.8121308	425.6764534	444.6726232	1520.241355	443.7204025	1.776247492	7.21E-15	7.93E-13	Polr3gl	3	96577872	96594181	-	1597	protein_coding	polymerase (RNA) III (DNA directed) polypeptide G like [Source:MGI Symbol;Acc:MGI:1917120]	-
ENSMUSG0000000537	444.9334117	460.2776773	435.6918677	1060.298566	993.574274	1188.064055	446.9676522	1080.645632	-1.273555577	7.37E-15	8.06E-13	Tbl2	5	135149657	135165760	+	7855	protein_coding	transducin (beta)-like 2 [Source:MGI Symbol;Acc:MGI:1351652]	-
ENSMUSG0000003998	709.4884133	737.2868973	774.1909342	1840.66453	3353.066263	2760.473156	740.3220816	2651.401316	-1.840437392	7.76E-15	8.44E-13	Dtx4	19	12466341	12501996	-	5765	protein_coding	deltex 4, E3 ubiquitin ligase [Source:MGI Symbol;Acc:MGI:2672905]	-
ENSMUSG0000002944	187.3931261	133.7649085	135.1761949	464.2574551	559.9966336	570.1929042	152.1114098	531.482331	-1.803070753	8.12E-15	8.78E-13	Psph	5	129765558	129787449	-	4329	protein_coding	phosphoserine phosphatase [Source:MGI Symbol;Acc:MGI:97788]	-
ENSMUSG0000002300	2242.7049	1560.941688	3702.263717	11482.4047	12623.13399	11097.35507	2501.970102	11734.29792	-2.229708845	8.23E-15	8.85E-13	Tuba1b	15	98931425	98934565	-	1875	protein_coding	tubulin, alpha 1B [Source:MGI Symbol;Acc:MGI:107804]	-
ENSMUSG0000003734	146.3069327	148.5106465	83.78689764	659.7796116	461.2317952	631.4935065	126.2014923	584.1683044	-2.209104008	9.33E-15	9.97E-13	Paqr7	4	134496697	134510235	+	4259	protein_coding	progestin and adipoQ receptor family member VII [Source:MGI Symbol;Acc:MGI:1919154]	-
ENSMUSG0000003815	9085.059258	7561.403765	5954.455526	2302.337992	2569.861095	2833.450063	7533.639516	2568.549716	1.552520307	9.52E-15	1.01E-12	Ormdl3	11	98581256	98587368	-	2184	protein_coding	ORM1-like 3 (S. cerevisiae) [Source:MGI Symbol;Acc:MGI:1913862]	-
ENSMUSG0000008514	344.7231838	317.0333658	377.5996187	880.2803695	871.1058745	813.4492626	346.4520561	854.9451689	-1.303646201	1.32E-14	1.40E-12	Mir22hg	11	75461539	75466676	+	1924	processed_transcrip	Mir22 host gene (non-protein coding) [Source:MGI Symbol;Acc:MGI:1914348]	-
ENSMUSG0000000150	2212.641831	1057.480064	2056.689047	16001.80891	7270.079753	9326.448783	1775.603648	10866.11248	-2.613477458	1.35E-14	1.42E-12	Col1a1	11	94936224	94953042	+	6563	protein_coding	collagen, type I, alpha 1 [Source:MGI Symbol;Acc:MGI:88467]	-
ENSMUSG0000006174	2631.520584	4652.280322	5031.682493	1051.685256	1146.659774	1018.757629	4105.161133	1072.367553	1.936585174	1.51E-14	1.58E-12	Cyp2d22	15	82370527	82380260	-	3909	protein_coding	cytochrome P450, family 2, subfamily d, polypeptide 22 [Source:MGI Symbol;Acc:MGI:1929474]	-
ENSMUSG0000007171	2044.288648	3265.127688	1324.950141	421.1909009	484.9353564	397.9674024	2211.455493	434.6978866	2.347065013	1.71E-14	1.78E-12	Csf2rb2	15	78282507	78305721	-	4740	protein_coding	colony stimulating factor 2 receptor, beta 2, low-affinity (granulocyte-macrophage) [Source:MGI Symbol;Acc:MGI:1339760]	-
ENSMUSG0000002136	678.4232426	691.9964164	906.0156532	201.551474	240.9862056	141.0886879	758.8117707	194.5421225	1.963381984	1.79E-14	1.85E-12	Edn1	13	42301476	42307990	+	2139	protein_coding	endothelin 1 [Source:MGI Symbol;Acc:MGI:95283]	-
ENSMUSG0000000117	5462.459521	5732.93226	5280.808869	10313.57842	11186.10559	10808.36652	5492.066883	10769.35018	-0.971473373	1.96E-14	2.02E-12	Calm1	12	100199435	100209814	+	4706	protein_coding	calmodulin 1 [Source:MGI Symbol;Acc:MGI:88251]	-
ENSMUSG0000002941	244.512956	220.1328022	174.2767471	611.5450708	756.5386619	928.2662638	212.9741684	765.4499988	-1.844291492	2.02E-14	2.07E-12	Cxcl9	5	92321347	92328079	-	3016	protein_coding	chemokine (C-X-C motif) ligand 9 [Source:MGI Symbol;Acc:MGI:1352449]	-
ENSMUSG0000001531	529.1100031	834.1874609	574.2195385	184.3248523	180.7396542	179.0366798	645.8390008	181.3670621	1.832105116	2.24E-14	2.27E-12	Gadd45b	10	80930073	80932204	+	1589	protein_coding	growth arrest and DNA-damage-inducible 45 beta [Source:MGI Symbol;Acc:MGI:107776]	-
ENSMUSG0000002003	1340.812849	1551.462285	1767.344961	556.4198813	634.0702624	626.6283794	1553.206698	605.7061743	1.358675347	2.44E-14	2.47E-12	Tcp11l2	10	84576626	84614359	+	3438	protein_coding	t-complex 11 (mouse) like 2 [Source:MGI Symbol;Acc:MGI:2444679]	-
ENSMUSG0000002263	546.1457418	505.5681581	350.7878115	1346.260487	2245.912425	1840.964121	467.5005705	1811.045677	-1.953077546	2.73E-14	2.74E-12	Alcam	16	52248996	52454074	-	7121	protein_coding	activated leukocyte cell adhesion molecule [Source:MGI Symbol;Acc:MGI:1313266]	-
ENSMUSG0000000122	1034.169552	1609.39197	1126.095904	419.4682387	383.2075729	354.1812579	1256.552475	385.6190232	1.703861371	2.86E-14	2.87E-12	Sema6b	17	56123085	56140343	-	3968	protein_coding	sema domain, transmembrane domain (TM), and cytoplasmic domain, (semaphorin) 6B [Source:MGI Symbol;Acc:MGI:1202889]	-
ENSMUSG0000011690	62.13034127	30.54474289	42.45202814	204.1354673	472.0959274	410.6167331	45.04237077	362.2827093	-3.005066876	3.32E-14	3.30E-12	AC161607.1	16	42884370	42912065	-	2525	antisense	novel transcript, antisense to Zbtb20	-
ENSMUSG0000007171	4034.463774	5529.65173	3018.562632	956.9388361	1339.251208	973.0254338	4194.226045	1089.738493	1.944617764	3.48E-14	3.45E-12	Csf2rb	15	78325752	78353847	+	7277	protein_coding	colony stimulating factor 2 receptor, beta, low-affinity (granulocyte-macrophage) [Source:MGI Symbol;Acc:MGI:1339759]	-
ENSMUSG0000009888	5.010511393	11.58593696	1.117158635	99.05307484	120.4931028	131.3584336	5.904535662	116.9682037	-4.296908879	3.65E-14	3.60E-12	Mir6392	15	84168270	84168377	+	108	miRNA	microRNA 6392 [Source:MGI Symbol;Acc:MGI:5530813]	-
ENSMUSG0000002868	220.4625013	99.00709763	90.48984945	1063.743891	1151.598015	635.3856083	136.6531495	950.2425048	-2.796002578	3.85E-14	3.78E-12	Ptch2	4	117096075	117116101	+	5974	protein_coding	patched 2 [Source:MGI Symbol;Acc:MGI:1095405]	-
ENSMUSG0000008629	563.1814806	562.4445759	1042.309007	169.6822239	113.5795641	148.8728914	722.6450211	144.0448931	2.325286394	4.53E-14	4.42E-12	Snhg12	4	132308623	132311024	+	2402	lincRNA	small nucleolar RNA host gene 12 [Source:MGI Symbol;Acc:MGI:1916721]	-
ENSMUSG0000007031	1577.308987	1480.893397	1715.955664	701.9848348	623.2061301	737.5532788	1591.386016	687.5814146	1.210453726	4.63E-14	4.47E-12	Eif3g	9	20894349	20898623	-	1984	protein_coding	eukaryotic translation initiation factor 3, subunit G [Source:MGI Symbol;Acc:MGI:1858258]	-
ENSMUSG0000005711	4866.208665	5695.014648	6008.07914	2635.673122	2283.443063	2249.634803	5523.100818	2389.583663	1.208532117	4.63E-14	4.47E-12	Npm1	11	33152287	33163206	-	2665	protein_coding	nucleophosmin 1 [Source:MGI Symbol;Acc:MGI:106184]	-
ENSMUSG0000004544	891.871028	1703.132733	2948.181638	87.85577073	253.8256346	212.1195446	1847.728466	184.6003166	3.324325913	4.83E-14	4.64E-12	Gprin3	6	59347226	59426294	-	8339	protein_coding	GPRIN family member 3 [Source:MGI Symbol;Acc:MGI:1924785]	-
ENSMUSG0000011212	308.6475018	410.7741285	364.1937151	62.87716925	108.6413222	72.97690753	361.2051151	81.49846633	2.150643596	5.01E-14	4.79E-12	Pbld1	10	63060162	63077962	+	3928	protein_coding	phenazine biosynthesis-like protein domain containing 1 [Source:MGI Symbol;Acc:MGI:1915621]	-
ENSMUSG0000004371	256.5381833	266.47655	90.48984945	1630.499745	1006.413703	1775.771417	204.5015276	1470.894955	-2.84548188	5.33E-14	5.06E-12	Col6a6	9	105687809	105828160	-	9166	protein_coding	collagen, type VI, alpha 6 [Source:MGI Symbol;Acc:MGI:2444259]	-
ENSMUSG0000005844	2215.648138	1839.004175	1710.369871	4377.284577	4436.51654	5136.601265	1921.674061	4650.134127	-1.274702362	6.47E-14	6.12E-12	Znrf2	6	54816916	54893500	+	6176	protein_coding	zinc and ring finger 2 [Source:MGI Symbol;Acc:MGI:1196246]	-
ENSMUSG0000004509	4793.055199	5916.200717	6521.972112	1755.392752	2319.986053	2247.688752	5743.742676	2107.689186	1.446445346	6.96E-14	6.54E-12	S1pr1	3	115710433	115715072	-	3046	protein_coding	sphingosine-1-phosphate receptor 1 [Source:MGI Symbol;Acc:MGI:1096355]	-
ENSMUSG0000002607	53.11142077	123.2322385	210.0258234	2.583993257	4.938241919	4.865127169	128.7898276	4.129120781	4.974263424	8.96E-14	8.38E-12	Il1r2	1	40074079	40125231	+	1908	protein_coding	interleukin 1 receptor, type II [Source:MGI Symbol;Acc:MGI:96546]	-
ENSMUSG0000003188	1234.590007	1434.549649	1407.61988	495.2653742	624.1937785	542.948192	1358.919845	554.1357816	1.294569629	9.62E-14	8.95E-12	D230025D16R	8	105225145	105253053	+	4704	protein_coding	RIKEN cDNA D230025D16 gene [Source:MGI Symbol;Acc:MGI:2443049]	-
ENSMUSG0000004204	38.07988659	60.03621878	18.9916968	254.9540013	353.5781214	267.5819943	39.03593405	292.038039	-2.899846872	1.09E-13	1.00E-11	2010003K11Ri	19	4496790	4498583	-	1165	protein_coding	RIKEN cDNA 2010003K11 gene [Source:MGI Symbol;Acc:MGI:1917111]	-
ENSMUSG0000002388	4755.977414	1280.772667	5385.82178	405.6869413	537.2807208	397.9674024	3807.523954	446.9783548	3.090674403	1.09E-13	1.00E-11	Thbs2	17	14665500	14694235	-	7003	protein_coding	thrombospondin 2 [Source:MGI Symbol;Acc:MGI:98738]	-
ENSMUSG0000005073	335.7042633	523.4736971	312.8044179	1197.250209	2691.341846	2199.03748	390.6607928	2029.209845	-2.376676072	1.30E-13	1.19E-11	Ptges	2	30889471	30929863	-	3931	protein_coding	prostaglandin E synthase [Source:MGI Symbol;Acc:MGI:1927593]	-
ENSMUSG0000001125	3027.350984	2820.649016	2491.263757	979.3334443	1282.95525	967.1872812	2779.754585	1076.491992	1.368930715	1.31E-13	1.19E-11	Pabpc4	4	123262351	123298925	+	4027	protein_coding	poly(A) binding protein, cytoplasmic 4 [Source:MGI Symbol;Acc:MGI:2385206]	-
ENSMUSG0000006068	2946.180699	3944.4849	4654.082874	1010.341363	1410.361892	1236.715326	3848.249491	1219.139527	1.658482511	1.38E-13	1.26E-11	Slc9a6	X	56609757	56664230	+	5098	protein_coding	solute carrier family 9 (sodium/hydrogen exchanger), member 6 [Source:MGI Symbol;Acc:MGI:2443511]	-
ENSMUSG0000009163	227.4772172	84.26135969	188.7998094	1054.269249	921.475942	790.0966522	166.8461288	921.947281	-2.465916261	1.49E-13	1.35E-11	Gm3756	9	31935446	31936800	-	1355	processed_pseudog	predicted gene 3756 [Source:MGI Symbol;Acc:MGI:3781931]	-
ENSMUSG0000004262	419.8808547	586.6697168	785.3625206	118.8636898	161.9743349	140.1156625	597.304364	140.3178957	2.090356002	1.71E-13	1.54E-11	Maff	15	79346621	79359076	+	2594	protein_coding	v-maf musculoaponeurotic fibrosarcoma oncogene family, protein F (avian) [Source:MGI Symbol;Acc:MGI:96910]	TF_b
ENSMUSG0000003577	2265.753252	1827.418238	1900.286839	852.7177747	918.5129969	863.0735598	1997.819443	878.1014438	1.186209222	1.90E-13	1.70E-11	Ggta1	2	35400179	35463231	-	4709	protein_coding	glycoprotein galactosyltransferase alpha 1, 3 [Source:MGI Symbol;Acc:MGI:95704]	-
ENSMUSG0000002043	6303.223333	7560.350498	5105.414963	2571.07329	2377.26966	2409.210974	6322.996264	2452.517975	1.366335755	1.98E-13	1.76E-11	Tcn2	11	3917192	3932159	-	2178	protein_coding	transcobalamin 2 [Source:MGI Symbol;Acc:MGI:98534]	-
ENSMUSG0000006434	37748.19073	37995.55362	37727.56427	75115.82264	110788.4698	103799.4342	37823.76954	96567.90888	-1.352245379	2.10E-13	1.86E-11	mt-Nd1	MT	2751	3707	+	957	protein_coding	mitochondrially encoded NADH dehydrogenase 1 [Source:MGI Symbol;Acc:MGI:101787]	-
ENSMUSG0000002866	75.1576709	47.39701482	37.9833936	380.7083398	230.1220734	311.3681388	53.51269311	307.3995174	-2.519009509	2.13E-13	1.88E-11	Ephb2	4	136647539	136835988	-	12608	protein_coding	Eph receptor B2 [Source:MGI Symbol;Acc:MGI:99611]	-
ENSMUSG0000004113	2303.833139	2166.570211	2066.743475	1061.159897	1049.870232	906.8597043	2179.048942	1005.963278	1.115048318	2.19E-13	1.92E-11	Zfp598	17	24669752	24682015	+	3286	protein_coding	zinc finger protein 598 [Source:MGI Symbol;Acc:MGI:2670965]	-

ENSMUSG0000007262	659.3832993	313.8735648	1099.284097	50.81853405	55.30830949	108.9788486	690.8469871	71.70189737	3.26939803	2.44E-13	2.13E-11	Gm5460	14	34016758	34048193	+	4336	protein_coding	predicted gene 5460 [Source:MGI Symbol;Acc:MGI:3643519]	-
ENSMUSG0000004910	421.8850593	268.583084	401.05995	1019.816005	1392.584221	1195.848258	363.8426978	1202.749495	-1.724697458	2.57E-13	2.23E-11	Ccr2	9	124101950	124113557	+	6760	protein_coding	chemokine (C-C motif) receptor 2 [Source:MGI Symbol;Acc:MGI:106185]	-
ENSMUSG0000003582	7560.861692	6118.42798	8103.86874	2732.142203	3186.153686	3193.469474	7261.052804	3037.255121	1.257482302	2.62E-13	2.27E-11	Pim3	15	88862186	88865726	+	2427	protein_coding	proviral integration site 3 [Source:MGI Symbol;Acc:MGI:1355297]	-
ENSMUSG0000005341	1961.114159	1645.203048	1568.490724	715.7661321	546.1695562	453.4298521	1724.935977	571.7885135	1.592404137	3.80E-13	3.28E-11	Cbx7	15	79915807	79971119	-	6487	protein_coding	chromobox 7 [Source:MGI Symbol;Acc:MGI:1196439]	-
ENSMUSG0000002667	103.2165347	89.52769467	94.95848399	369.5110357	273.5786023	330.8286475	95.90090445	324.6394285	-1.759415639	3.85E-13	3.30E-11	Hsd17b7	1	169949535	169969241	-	5104	protein_coding	hydroxysteroid (17-beta) dehydrogenase 7 [Source:MGI Symbol;Acc:MGI:1330808]	-
ENSMUSG0000006106	41.08619342	29.49147589	23.46033134	235.1433864	168.8878736	158.6031457	31.34600022	187.5448019	-2.577237224	3.89E-13	3.32E-11	Mcpt4	14	56059629	56062313	-	1108	protein_coding	mast cell protease 4 [Source:MGI Symbol;Acc:MGI:96940]	-
ENSMUSG0000002257	5444.42168	4445.839991	7457.03389	2001.733443	1789.618871	2024.865928	5782.431853	1938.739414	1.576469751	3.93E-13	3.34E-11	Gpihbp1	15	75596628	75599481	+	2554	protein_coding	GPI-anchored HDL-binding protein 1 [Source:MGI Symbol;Acc:MGI:1915703]	-
ENSMUSG0000001242	5745.052363	4011.893988	6688.428749	14452.27429	17133.72416	17060.05493	5481.7917	16215.35113	-1.564659801	4.42E-13	3.74E-11	Steap4	5	7960457	7982213	+	3155	protein_coding	STEAP family member 4 [Source:MGI Symbol;Acc:MGI:1923560]	-
ENSMUSG0000002496	53.11142077	46.34374783	40.21771087	206.7194605	185.6778961	184.8748324	46.55762649	192.424063	-2.045190772	4.56E-13	3.84E-11	Rcor2	19	7267325	7275225	+	3135	protein_coding	REST corepressor 2 [Source:MGI Symbol;Acc:MGI:1859854]	MYB
ENSMUSG0000002532	158.33216	104.2734326	189.916968	570.2011787	551.1077981	517.6495308	150.8408535	546.3195025	-1.857691221	4.99E-13	4.18E-11	Atp10a	7	58656166	58829420	+	5587	protein_coding	ATPase, class V, type 10A [Source:MGI Symbol;Acc:MGI:1330809]	-
ENSMUSG0000000005	6862.396404	4331.033888	5724.320847	14089.6539	16415.70379	18383.36952	5639.25038	16296.2424	-1.530923687	5.07E-13	4.23E-11	Cav2	6	17281185	17289115	+	3335	protein_coding	caveolin 2 [Source:MGI Symbol;Acc:MGI:107571]	-
ENSMUSG0000002180	1352.838076	732.0205623	654.6549602	3265.306145	3774.792123	4828.152202	913.1711996	3956.08349	-2.114719555	6.01E-13	5.00E-11	Nid2	14	19751265	19811787	+	5174	protein_coding	nidogen 2 [Source:MGI Symbol;Acc:MGI:1298229]	-
ENSMUSG0000009776	102.2144324	109.5397676	141.8791467	8.613310856	19.75296767	10.70327977	117.8777822	13.0231861	3.187231065	7.50E-13	6.21E-11	4732463B04Ri	12	84039469	84053807	-	2932	antisense	RIKEN cDNA 4732463B04 gene [Source:MGI Symbol;Acc:MGI:3642483]	-
ENSMUSG0000009008	275.5781266	301.2343609	230.1346789	806.2058961	649.8726365	906.8597043	268.9823888	787.646079	-1.549602952	8.15E-13	6.72E-11	Srpx	X	10037977	10117709	-	2652	protein_coding	sushi-repeat-containing protein [Source:MGI Symbol;Acc:MGI:1858306]	-
ENSMUSG0000003545	186.3910238	136.9247095	367.545191	20.67194605	2.962945151	2.919076301	230.2869748	8.851322502	4.677206142	8.54E-13	7.01E-11	Stab2	10	86841198	87008025	-	8757	protein_coding	stabilin 2 [Source:MGI Symbol;Acc:MGI:2178743]	-
ENSMUSG0000002619	3432.200304	1604.125635	2599.628144	8311.844976	15340.1547	11166.43988	2545.318028	11606.14652	-2.188903251	8.87E-13	7.25E-11	Fn1	1	71585520	71653200	-	11685	protein_coding	fibronectin 1 [Source:MGI Symbol;Acc:MGI:95566]	-
ENSMUSG0000002289	1870.924954	993.2307773	2794.013747	7681.350621	9876.483837	12314.60989	1886.056493	9957.48145	-2.40049042	9.53E-13	7.76E-11	Adamts5	16	85856173	85901828	-	10791	protein_coding	a disintegrin-like and metallopeptidase (reprolysin type) with thrombospondin type 1 motif, 5 (aggrecanase-2) [Source:MGI Symbo	-
ENSMUSG0000007527	24.05045469	18.95880593	10.05442772	143.8422913	112.5919157	193.6320613	17.68789611	150.0220895	-3.076614448	9.67E-13	7.83E-11	Pde11a	2	75989141	76338774	-	5208	protein_coding	phosphodiesterase 11A [Source:MGI Symbol;Acc:MGI:3036251]	-
ENSMUSG0000005839	724.5199474	1394.525503	880.3210046	287.6845826	204.4432154	247.1484602	999.7888183	246.4254194	2.019718608	1.06E-12	8.55E-11	Gpr182	10	127747276	127751732	-	3954	protein_coding	G protein-coupled receptor 182 [Source:MGI Symbol;Acc:MGI:109545]	-
ENSMUSG0000005607	1750.672681	1356.607891	5790.233206	6.029317599	43.45652888	33.08286475	2965.837926	27.52290374	6.754155997	1.07E-12	8.59E-11	S100a9	3	90692632	90695721	-	522	protein_coding	S100 calcium binding protein A9 (calgranulin B) [Source:MGI Symbol;Acc:MGI:1338947]	-
ENSMUSG0000004528	385.8093773	242.2514091	210.0258234	1169.687614	871.1058745	1086.86941	279.3622033	1042.554299	-1.898916153	1.33E-12	1.06E-10	Rtn4rl1	11	75193783	75267769	+	3525	protein_coding	reticulon 4 receptor-like 1 [Source:MGI Symbol;Acc:MGI:2661375]	-
ENSMUSG0000003127	368.7736385	267.529817	215.6116166	1458.233528	806.9087295	1249.364657	283.9716907	1171.502305	-2.04391993	1.37E-12	1.09E-10	Col4a5	X	141475385	141689234	+	6718	protein_coding	collagen, type IV, alpha 5 [Source:MGI Symbol;Acc:MGI:88456]	-
ENSMUSG0000002871	3385.101497	4479.544534	2802.951016	1277.354	1359.991824	1165.68447	3555.865682	1267.676765	1.488063382	1.44E-12	1.14E-10	Cyp4b1	4	115624725	115647723	-	2256	protein_coding	cytochrome P450, family 4, subfamily b, polypeptide 1 [Source:MGI Symbol;Acc:MGI:103225]	-
ENSMUSG0000003783	244.512956	163.2563844	464.7379923	40.48256102	41.48123212	32.10983931	290.8357775	38.02454415	2.933962496	1.47E-12	1.16E-10	Sh2d4b	14	40813789	40893266	-	4236	protein_coding	SH2 domain containing 4B [Source:MGI Symbol;Acc:MGI:1925182]	-
ENSMUSG0000003482	144.3027281	208.5468652	155.2850503	32.73058125	32.59239666	38.92101735	169.3782145	34.74799842	2.286064931	1.58E-12	1.24E-10	Nxnl1	8	71560555	71566601	-	2730	protein_coding	nucleoredoxin-like 1 [Source:MGI Symbol;Acc:MGI:1924446]	-
ENSMUSG0000002855	3042.382518	2575.237805	3238.642884	5909.592578	6571.812345	6955.185801	2952.087736	6478.863575	-1.134005366	1.78E-12	1.39E-10	Rnf11	4	109451098	109484728	-	4263	protein_coding	ring finger protein 11 [Source:MGI Symbol;Acc:MGI:1352759]	-
ENSMUSG0000003700	21.04414785	12.63920395	4.468634541	106.8050546	163.9496317	166.3873492	12.71732878	145.7140118	-3.504602717	1.79E-12	1.39E-10	Xpnpep2	X	48108725	48136981	+	4878	protein_coding	X-prolyl aminopeptidase (aminopeptidase P) 2, membrane-bound [Source:MGI Symbol;Acc:MGI:2180001]	-
ENSMUSG0000002515	7925.626922	12328.49019	4441.822734	66468.91988	253475.0194	180943.8097	8231.979948	166962.583	-4.342135738	2.01E-12	1.56E-10	Fasn	11	120805846	120824547	-	10947	protein_coding	fatty acid synthase [Source:MGI Symbol;Acc:MGI:95485]	-
ENSMUSG0000005022	66.13875039	53.7166168	46.92066268	250.6473459	201.4802703	208.2274428	55.59200996	220.118353	-1.983447803	2.36E-12	1.83E-10	Il17d	14	57524777	57543166	+	1274	protein_coding	interleukin 17D [Source:MGI Symbol;Acc:MGI:2446510]	-
ENSMUSG0000004583	458.9628436	589.8295178	454.6835645	1342.815162	1129.869751	1242.553479	501.158642	1238.412797	-1.305264194	3.06E-12	2.36E-10	Ccdc9b	2	118754158	118762661	-	4779	protein_coding	coiled-coil domain containing 9B [Source:MGI Symbol;Acc:MGI:2685199]	-
ENSMUSG0000003900	4526.495993	4113.00762	4045.231418	2248.935464	1849.865423	2012.216597	4228.24501	2037.005828	1.053468409	3.31E-12	2.54E-10	Rps21	2	180257377	180258445	+	810	protein_coding	ribosomal protein S21 [Source:MGI Symbol;Acc:MGI:1913731]	-
ENSMUSG0000001997	4178.766502	3142.948716	2244.371698	8507.367132	13006.34157	13628.19423	3188.695639	11713.96764	-1.877066994	3.65E-12	2.79E-10	Epb41l2	10	25359798	25523519	+	11843	protein_coding	erythrocyte membrane protein band 4.1 like 2 [Source:MGI Symbol;Acc:MGI:103009]	-
ENSMUSG0000004999	189.3973307	192.7478603	148.5820985	441.8628469	597.5272722	541.9751666	176.9090965	527.1217619	-1.573644896	3.73E-12	2.84E-10	Ppp1r3d	2	178411206	178414472	-	3267	protein_coding	protein phosphatase 1, regulatory subunit 3D [Source:MGI Symbol;Acc:MGI:1917664]	-
ENSMUSG0000001943	262.550797	420.2535314	255.8293275	950.9095185	1126.906806	955.510976	312.8778853	1011.1091	-1.692055906	4.05E-12	3.06E-10	Tlcd1	11	78176711	78181909	+	5009	protein_coding	TLC domain containing 1 [Source:MGI Symbol;Acc:MGI:1915572]	-
ENSMUSG0000003787	448.9418208	642.4928676	1101.518414	143.8422913	168.8878736	136.2235607	730.9843676	149.6512419	2.288031868	4.19E-12	3.16E-10	Ackr1	1	173331886	173333750	-	1405	protein_coding	atypical chemokine receptor 1 (Duffy blood group) [Source:MGI Symbol;Acc:MGI:1097689]	-
ENSMUSG0000004307	1741.65376	1331.329483	1265.740734	540.0545907	506.6636209	343.4779781	1446.241326	463.3987299	1.641702802	4.26E-12	3.20E-10	Synpo	18	60593973	60660142	-	9533	protein_coding	synaptopodin [Source:MGI Symbol;Acc:MGI:1099446]	-
ENSMUSG0000003010	384.807275	337.0454388	306.1014661	1104.226452	871.1058745	804.6920337	342.6513933	926.6747866	-1.435133617	4.31E-12	3.23E-10	Bhlhe40	6	108660629	108666925	+	4705	protein_coding	basic helix-loop-helix family, member e40 [Source:MGI Symbol;Acc:MGI:1097714]	bHLH
ENSMUSG0000002804	216.4540922	166.4161854	176.5110644	571.0625097	476.046521	494.2969204	186.4604473	513.8019837	-1.461879057	4.63E-12	3.45E-10	Cks1b	3	89415472	89418383	-	1126	protein_coding	CDC28 protein kinase 1b [Source:MGI Symbol;Acc:MGI:1889208]	-
ENSMUSG0000002662	5800.167989	5227.364102	4536.781218	10737.35331	16854.21967	16592.0297	5188.104436	14727.86756	-1.505200293	4.75E-12	3.53E-10	Ppp2r5a	1	191351975	191403272	-	9607	protein_coding	protein phosphatase 2, regulatory subunit B', alpha [Source:MGI Symbol;Acc:MGI:2388479]	-
ENSMUSG0000000248	141.2964213	127.4453065	121.7702912	333.3351301	530.3671821	453.4298521	130.170673	439.0440548	-1.752568879	4.97E-12	3.67E-10	Tiam1	16	89787111	90143769	-	10048	protein_coding	T cell lymphoma invasion and metastasis 1 [Source:MGI Symbol;Acc:MGI:103306]	-
ENSMUSG0000002719	81.17028457	43.18394684	44.68634541	456.5054754	210.3691057	403.805555	56.34685894	356.8933787	-2.660939824	4.99E-12	3.67E-10	Gatm	2	122594467	122611303	-	3001	protein_coding	glycine amidinotransferase (L-arginine:glycine amidinotransferase) [Source:MGI Symbol;Acc:MGI:1914342]	-
ENSMUSG0000002735	3983.356558	2897.537506	5742.195385	853.5791058	1320.485889	1176.387749	4207.696483	1116.817581	1.91380663	5.26E-12	3.86E-10	Crls1	2	132846666	132866785	+	2082	protein_coding	cardiolipin synthase 1 [Source:MGI Symbol;Acc:MGI:1913836]	-
ENSMUSG0000002648	2572.396549	3112.403973	1465.712129	717.4887943	589.6260851	608.1408961	2383.504217	638.4185918	1.900411604	5.34E-12	3.90E-10	Coq8a	1	180165238	180199602	-	5293	protein_coding	coenzyme Q8A [Source:MGI Symbol;Acc:MGI:1914676]	-
ENSMUSG0000005195	395.8304001	323.3529678	380.9510946	792.4245987	872.0935228	889.3452465	366.7114875	851.2877894	-1.214697843	5.83E-12	4.24E-10	B3glct	5	149678230	149762599	+	4798	protein_coding	beta-3-glucosyltransferase [Source:MGI Symbol;Acc:MGI:2685903]	-
ENSMUSG0000010717	2702.669845	2426.727159	2590.690875	1255.820723	896.7847324	1118.006223	2573.362627	1090.203893	1.23870789	6.03E-12	4.37E-10	Gm9794	6	66875321	66875743	-	423	processed_pseudog	predicted pseudogene 9794 [Source:MGI Symbol;Acc:MGI:3642653]	-
ENSMUSG0000002280	3554.456782	3482.100689	4556.890073	1557.286603	1727.397023	1784.528646	3864.482515	1689.737424	1.193504614	6.41E-12	4.63E-10	Osbpl11	16	33185071	33243312	+	5091	protein_coding	oxysterol binding protein-like 11 [Source:MGI Symbol;Acc:MGI:2146553]	-
ENSMUSG0000003040	920.931994	910.0226846	510.5414963	2239.460823	3023.191703	2696.253477	780.498725	2652.968667	-1.764664588	6.72E-12	4.84E-10	Dmpk	7	19083849	19093821	+	4330	protein_coding	dystrophia myotonica-protein kinase [Source:MGI Symbol;Acc:MGI:94906]	-
ENSMUSG0000004631	357.7505135	353.8977107	250.2435343	788.9792744	938.2659646	904.9136534	320.6305861	877.3862975	-1.451188792	6.75E-12	4.84E-10	AI464131	4	41495604	41503076	-	4701	protein_coding	expressed sequence AI464131 [Source:MGI Symbol;Acc:MGI:2140300]	-
ENSMUSG0000003138	460.9670482	421.3067984	389.8883637	877.6963762	1071.598496	1001.243171	424.0540701	983.5126813	-1.212928247	6.83E-12	4.88E-10	Vegfd	X	164373378	164402650	+	2408	protein_coding	vascular endothelial growth factor D [Source:MGI Symbol;Acc:MGI:108037]	-
ENSMUSG0000003020	3411.156156	1729.464408	3453.137341	9029.33377	10598.45481	10922.21049	2864.585968	10183.33302	-1.829810294	7.07E-12	5.03E-10	Emp1	6	135362545	135383173	+	5935	protein_coding	epithelial membrane protein 1 [Source:MGI Symbol;Acc:MGI:107941]	-
ENSMUSG0000003016	25731.98231	21219.1169	22256.03433	9834.678335	12032.52026	10776.25668	23069.04451	10881.15176	1.084166303	7.19E-12	5.09E-10	Adipor2	6	119353150	119417704	-	5979	protein_coding	adiponectin receptor 2 [Source:MGI Symbol;Acc:MGI:93830]	-
ENSMUSG0000001597	1177.470177	1134.368555	1157.376346	583.982476	566.9101723	598.4106418	1156.405026	583.1010967	0.987823021	8.20E-12	5.79E-10	Actr8	14	29978337	30006354	+	9009	protein_coding	ARP8 actin-related protein 8 [Source:MGI Symbol;Acc:MGI:1860775]	-
ENSMUSG0000007856	9000.882667	10967.66923	12280.92488	4854.461998	5066.636209	4713.335201	10749.82559	4878.144469	1.139875124	8.36E-12	5.88E-10	Bnip3	7	138890836	138909519	-	2265	protein_coding	BCL2/adenovirus E1B interacting protein 3 [Source:MGI Symbol;Acc:MGI:109326]	-
ENSMUSG0000005396	866.818471	614.0546587	1746.118947	212.7487781	213.3320509	170.2794509	1075.664026	198.78676	2.435510499	9.05E-12	6.34E-10	Lgals4	7	28833854	28841708	+	2315	protein_coding	lectin, galactose binding, soluble 4 [Source:MGI Symbol;Acc:MGI:107536]	-
ENSMUSG0000003995	1438.01677	1377.673231	1969.550674	3828.616675	3798.495684	4640.358294	1595.080225	4089.156884	-1.358358355	9.91E-12	6.92E-10	Mrap	16	90738207	90749785	+	1581	protein_coding	melanocortin 2 receptor accessory protein [Source:MGI Symbol;Acc:MGI:1924287]	-
ENSMUSG0000003909	81.17028457	82.1548257	73.73246992	238.5887107	236.0479637	292.8806556	79.0191934	255.83911	-1.693967455	1.04E-11	7.24E-10	Wdr93	7	79743163	79785950	+	2285	protein_coding	WD repeat domain 93 [Source:MGI Symbol;Acc:MGI:3646885]	-
ENSMUSG0000003087	498.0448325	283.328822	681.4667675	1816.54726	1919.00081	2908.373022	487.613474	2214.640364	-2.183527355	1.07E-11	7.40E-10	Cdr2	7	120957036	120982312	-	5684	protein_coding	cerebellar degeneration-related 2 [Source:MGI Symbol;Acc:MGI:1100885]	-
ENSMUSG0000003191	21.04414785	15.79900494	33.51475906	129.1996628	198.5173251	137.1965862	23.45263728	154.9711914	-2.728333107	1.21E-11	8.36E-10	Has3	8	106870242	106884566	+	6380	protein_coding	hyaluronan synthase 3 [Source:MGI Symbol;Acc:MGI:109599]	-
ENSMUSG0000003925	92.19340963	41.07741285	100.5442772	407.4096035	347.6522311	368.7766394	77.93836655	374.6128247	-2.265820628	1.25E-11	8.61E-10	Lgi2	5	52533517	52566462	-	6528	protein_coding	leucine-rich repeat LGI family, member 2 [Source:MGI Symbol;Acc:MGI:2180196]	-
ENSMUSG0000004579	1270.665689	1151.220827	1352.879107	617.5743884	604.4408109	609.1139215	1258.255208	610.3763736	1.043489684	1.29E-11	8.81E-10	Whamm	7	81571266	81596836	+	3176	protein_coding	WAS protein homolog associated with actin, golgi membranes and microtubules [Source:MGI Symbol;Acc:MGI:2142282]	-
ENSMUSG0000005786	4540.525424	3862.330075	4217.273848	2235.154167	1843.939532	2021.946851	4206.709782	2033.680184	1.048457135	1.52E-11	1.04E-09	Rpl36	17	56613416	56614243	+	383	protein_coding	ribosomal protein L36 [Source:MGI Symbol;Acc:MGI:1860603]	-
ENSMUSG0000001844	2081.366433	1881.134855	1823.202893	937.1282211	1024.191374	921.4550858	1928.56806	960.9248936	1.005268604	1.53E-11	1.04E-09	C1qbp	11	70977836	70983026	-	2826	protein_coding	complement component 1, q subcomponent binding protein [Source:MGI Symbol;Acc:MGI:1194505]	-
ENSMUSG0000001884	4594.638947	6159.505393	2903.495293	13225.73882	17895.20107	21873.61175	4552.546545	17664.85055	-1.956082532	1.61E-11	1.09E-09	Pank3	11	35769484	35791285	+	7376	protein_coding	pantothenate kinase 3 [Source:MGI Symbol;Acc:MGI:2387464]	-
ENSMUSG0000000194	3493.328543	6204.795874	4928.903899	1633.945069	1828.137158	1549.056491	4875.676105	1670.379573	1.545415044	1.68E-11	1.14E-09	Esam	9	37528078	37538319	+	2867	protein_coding	endothelial cell-specific adhesion molecule [Source:MGI Symbol;Acc:MGI:1916774]	-
ENSMUSG0000007895	178.3742056	223.2926032	98.3099599	12.0586352	28.64180313	17.51445781	166.6589229	19.40496538	3.110126978	1.70E-11	1.14E-09	Gm14221	2	160568379	160619971	-	1667	lincRNA	predicted gene 14221 [Source:MGI Symbol;Acc:MGI:3650696]	-
ENSMUSG0000002526	314.6601155	205.3870642	255.8293275	987.9467552	650.8602849	837.7748985	258.6255024	825.5273128	-1.674313384	1.74E-11	1.17E-09	Maged2	X	150806370	150814345	-	3239	protein_coding	melanoma antigen, family D, 2 [Source:MGI Symbol;Acc:MGI:1933391]	-
ENSMUSG0000003835	233.4898309	163.2563844	193.2684439	629.6330236	525.4289402	512.7844036	196.6715531	555.9487891	-1.498864949	1.91E-11	1.27E-09	Sgsm2	11	74849261	74897060	-	6884	protein_coding	small G protein signaling modulator 2 [Source:MGI Symbol;Acc:MGI:2144695]	-
ENSMUSG0000004004	4384.197469	3634.824404	4194.930675	1745.91811	1996.037384	2091.031657	4071.317516	1944.32905	1.066394845	2.15E-11	1.43E-09	Rbms2	10	128131558	128180297	-	4484	protein_coding	RNA binding motif, single stranded interacting protein 2 [Source:MGI Symbol;Acc:MGI:1861776]	-
ENSMUSG0000002618	3458.254964	2308.761255	1844.428907	8507.367132	6828.600925	9040.379305	2537.148375	8125.449121	-1.679100135	2.29E-11	1.52E-09	Igfbp5	1	72857932	72874884	-	6006	protein_coding	insulin-like growth factor binding protein 5 [Source:MGI Symbol;Acc:MGI:96440]	-
ENSMUSG0000003180	1795.767283	2121.27973	2008.651226	870.8057275	1008.389	777.4473216	1975.232747	885.5473496	1.157412043	2.50E-11	1.65E-09	Jak3	8	71676296	71690575	+	5352	protein_coding	Janus kinase 3 [Source:MGI Symbol;Acc:MGI:99928]	-
ENSMUSG0000009578	227.4772172	136.9247095	328.4446388	38.75989885	36.5429902	43.78614452	230.9488552	39.69634452	2.540048585	2.54E-11	1.67E-09	Nupr1l	5	129908540	129913789	+	2489	protein_coding	nuclear protein transcriptional regulator 1 like [Source:MGI Symbol;Acc:MGI:1923099]	-
ENSMUSG0000004259	2892.067176	3103.977838	2867.746217	887.1710182	1276.041712	1350.559302	2954.597077	1171.257344	1.335308797	2.56E-11	1.68E-09	Kdm7a	6	39136623	39206789	-	10057	protein_coding	lysine (K)-specific demethylase 7A [Source:MGI Symbol;Acc:MGI:2443388]	-
ENSMUSG0000002687	3068.437177	2701.629845	2744.858767	5749.384996	9093.278669	10664.35875	2838.308596	8502.340807	-1.582750907	2.61E-11	1.70E-09	Zeb2	2	44983632	45117395	-	19942	protein_coding	zinc finger E-box binding homeobox 2 [Source:MGI Symbol;Acc:MGI:1344407]	zf-C2
ENSMUSG0000006424	87.18289824	53.7166168	64.79520084	5.167986514	3.950593535	4.865127169	68.56490529	4.661235739	3.875032091	2.63E-11	1.72E-09	Chil1	1	134182176	134190181	+	2804	protein_coding	chitinase-like 1 [Source:MGI Symbol;Acc:MGI:1340899]	-
ENSMUSG0000002595	2269.761661	2687.937374	1433.314529	5787.283564	6693.293097	7686.900927	2130.337855	6722.492529	-1.657767189	2.70E-11	1.75E-09	Idh1	1	65158616	65186500	-	2771	protein_coding	isocitrate dehydrogenase 1 (NADP+), soluble [Source:MGI Symbol;Acc:MGI:96413]	-
ENSMUSG0000002020	71.14926178	47.39701482	88.25553218	318.6925017	235.0603153	291.9076301	68.93393626	281.8868157	-2.033835996	2.74E-11	1.77E-09	Phlda1	10	111506286	111508645	+	2205	protein_coding	pleckstrin homology like domain, family A, member 1 [Source:MGI Symbol;Acc:MGI:1096880]	-
ENSMUSG0000002136	1359.852792	1145.954492	1837.725955	473.7320971	555.0583917	536.137014	1447.844413	521.6425009	1.472867657	2.78E-11	1.80E-09	Gcnt2	13	40859754	40960892	+	7051	protein_coding	glucosaminyl (N-acetyl) transferase 2, I-branching enzyme [Source:MGI Symbol;Acc:MGI:1100870]	-
ENSMUSG0000002139	2584.421777	1727.357874	1097.04978	6607.270758	6448.356297	6769.337943	1802.943143	6608.321666	-1.873711279	2.83E-11	1.82E-09	Ogn	13	49608046	49624501	+	2566	protein_coding	osteoglycin [Source:MGI Symbol;Acc:MGI:109278]	-
ENSMUSG0000011023	79.16608001	110.5930346	218.9630925	8.613310856	15.80237414	13.62235607	136.2407357	12.67934702	3.431033239	2.89E-11	1.85E-09	Gm18258	7	138899388	138900239	+	542	processed_pseudog	predicted gene, 18258 [Source:MGI Symbol;Acc:MGI:5010443]	-
ENSMUSG0000003657	2032.263421	1719.985005	2122.601407	920.7629305	959.994229	966.2142557	1958.283278	948.9904717	1.045174317	3.15E-11	2.01E-09	Fxyd1	7	31051678	31057199	-	2186	protein_coding	FXYD domain-containing ion transport regulator 1 [Source:MGI Symbol;Acc:MGI:1889273]	-
ENSMUSG0000006636	11.02312506	6.319601977	15.64022089	68.90648685	153.0854995	129.4123827	10.99431598	117.1347897	-3.41682198	3.67E-11	2.34E-09	Serpina1a	12	103853589	103863562	-	1832	protein_coding	serine (or cysteine) peptidase inhibitor, clade A, member 1A [Source:MGI Symbol;Acc:MGI:891971]	-
ENSMUSG0000001850	687.4421631	544.539037	611.0857735	242.8953661	244.9367992	275.3661978	614.3556579	254.3994544	1.272485051	3.82E-11	2.43E-09	Cenpv	11	62524946	62539261	-	1811	protein_coding	centromere protein V [Source:MGI Symbol;Acc:MGI:1920389]	-
ENSMUSG0000002944	72.15136406	319.1398998	80.43542174	6.890648685	7.90118707	9.730254338	157.2422285	8.174030031	4.268720906	3.86E-11	2.44E-09	Hpd	5	123171807	123182727	-	2128	protein_coding	4-hydroxyphenylpyruvic acid dioxygenase [Source:MGI Symbol;Acc:MGI:96213]	-
ENSMUSG0000007965	8.016818229	13.69247095	6.702951811	88.71710182	89.87600292	76.86900927	9.470746997	85.154038	-3.166349352	4.03E-11	2.54E-09	Prrt4	6	29169232	29179584	-	3411	protein_coding	proline-rich transmembrane protein 4 [Source:MGI Symbol;Acc:MGI:2141677]	-
ENSMUSG0000003251	370.7778431	294.9147589	392.122681	130.0609939	125.4313447	110.9248995	352.6050943	122.1390794	1.528696943	4.27E-11	2.68E-09	Csrnp1	9	119971163	119984658	-	3473	protein_coding	cysteine-serine-rich nuclear protein 1 [Source:MGI Symbol;Acc:MGI:2387989]	-
ENSMUSG0000005865	7581.90584	9145.517327	6578.947203	3760.57152	3374.794527	3271.311508	7768.790123	3468.892518	1.163178014	4.66E-11	2.91E-09	Eif4b	15	102073773	102097173	+	4468	protein_coding	eukaryotic translation initiation factor 4B [Source:MGI Symbol;Acc:MGI:95304]	-
ENSMUSG0000003016	4533.510708	5444.337103	7796.650115	2320.425945	1929.864942	2057.948792	5924.832642	2102.74656	1.49435021	4.73E-11	2.95E-09	Gabarapl1	6	129533160	129542346	+	2013	protein_coding	gamma-aminobutyric acid (GABA) A receptor-associated protein-like 1 [Source:MGI Symbol;Acc:MGI:1914980]	-
ENSMUSG0000002212	11.02312506	7.372868973	4.468634541	78.38112879	68.14773848	116.7630521	7.621542859	87.76397311	-3.513800626	5.62E-11	3.49E-09	Dct	14	118012790	118052244	-	5895	protein_coding	dopachrome tautomerase [Source:MGI Symbol;Acc:MGI:102563]	-
ENSMUSG0000002026	1937.063705	1887.454457	2597.393827	913.8722818	932.3400743	937.0234927	2140.637329	927.7452829	1.206104355	5.79E-11	3.59E-09	Appl2	10	83600033	83648738	-	5949	protein_coding	adaptor protein, phosphotyrosine interaction, PH domain and leucine zipper containing 2 [Source:MGI Symbol;Acc:MGI:2384914]	-
ENSMUSG0000002933	1477.098759	1070.119268	1055.71491	2610.69452	3005.414032	3232.390491	1200.977646	2949.499681	-1.295911303	6.02E-11	3.71E-09	Antxr2	5	97882783	98031043	-	6655	protein_coding	anthrax toxin receptor 2 [Source:MGI Symbol;Acc:MGI:1919164]	-
ENSMUSG0000003766	701.471595	1140.688157	1052.363434	345.3937653	332.8375053	346.3970544	964.8410621	341.542775	1.4978982	6.03E-11	3.71E-09	Gpr160	3	30855950	30897192	+	4227	protein_coding	G protein-coupled receptor 160 [Source:MGI Symbol;Acc:MGI:1919112]	-
ENSMUSG0000001479	139.2922167	140.0845105	107.247229	364.3430492	329.8745602	384.3450463	128.8746521	359.5208852	-1.478825091	6.39E-11	3.92E-09	Elmo3	8	105305601	105310760	+	3485	protein_coding	engulfment and cell motility 3 [Source:MGI Symbol;Acc:MGI:2679007]	-
ENSMUSG0000002821	1874.933363	7787.856169	4526.72679	829.4618354	775.3039812	794.9617794	4729.838774	799.9091987	2.563830939	6.41E-11	3.92E-09	Trp53inp1	4	11156431	11174379	+	6475	protein_coding	transformation related protein 53 inducible nuclear protein 1 [Source:MGI Symbol;Acc:MGI:1926609]	-
ENSMUSG0000000198	86.18079596	109.5397676	71.49815265	312.6631841	259.7515249	301.6378845	89.0729054	291.3508645	-1.708974867	6.69E-11	4.08E-09	Gria3	X	41400854	41678601	+	9859	protein_coding	glutamate receptor, ionotropic, AMPA3 (alpha 3) [Source:MGI Symbol;Acc:MGI:95810]	-
ENSMUSG0000007436	12056.29251	7897.395937	10257.75059	21837.32701	27326.25548	31010.32057	10070.47968	26724.63436	-1.408014248	6.78E-11	4.11E-09	Ehd2	7	15946958	15967567	-	5119	protein_coding	EH-domain containing 2 [Source:MGI Symbol;Acc:MGI:2154274]	-
ENSMUSG0000003241	4264.947298	4156.191567	2962.704701	9239.498555	28626.9884	17202.11664	3794.614522	18356.2012	-2.274194236	6.79E-11	4.11E-09	Me1	9	86581371	86695953	-	3848	protein_coding	malic enzyme 1, NADP(+)-dependent, cytosolic [Source:MGI Symbol;Acc:MGI:97043]	-
ENSMUSG0000004849	1959.109955	2161.303876	1748.353264	3902.691149	6306.13493	6967.835131	1956.255698	5725.553737	-1.549202776	7.54E-11	4.55E-09	Nrip1	16	76287400	76373827	-	11457	protein_coding	nuclear receptor interacting protein 1 [Source:MGI Symbol;Acc:MGI:1315213]	-
ENSMUSG0000005037	989.074949	840.5070629	835.6346591	385.8763263	426.6641018	418.4009365	888.405557	410.3137882	1.115134955	7.64E-11	4.60E-09	Snx21	2	164785823	164793816	+	4760	protein_coding	sorting nexin family member 21 [Source:MGI Symbol;Acc:MGI:1917729]	-
ENSMUSG0000000056	52.10931849	23.17187391	10.05442772	561.5878678	205.4308638	244.2293839	28.44520671	337.0827052	-3.561370189	7.69E-11	4.61E-09	Sox9	11	112782224	112787760	+	4135	protein_coding	SRY (sex determining region Y)-box 9 [Source:MGI Symbol;Acc:MGI:98371]	HMG
ENSMUSG0000001477	124.2606825	51.61008281	99.42711853	327.3058125	465.1823887	516.6765053	91.7659613	436.3882355	-2.248445	8.09E-11	4.84E-09	Nol3	8	105276140	105281939	+	3176	protein_coding	nucleolar protein 3 (apoptosis repressor with CARD domain) [Source:MGI Symbol;Acc:MGI:1925938]	-
ENSMUSG0000002593	569.1940943	634.0667317	656.8892775	1197.250209	1578.262117	1398.237548	620.0500345	1391.249958	-1.16591923	8.93E-11	5.32E-09	Lactb2	1	13623330	13660546	-	4566	protein_coding	lactamase, beta 2 [Source:MGI Symbol;Acc:MGI:2442551]	-
ENSMUSG0000007163	2358.948764	3001.810939	6237.09666	892.3390047	959.0065806	812.4762372	3865.952121	887.9406075	2.122196521	9.30E-11	5.53E-09	Cebpd	16	15887286	15891031	+	3746	protein_coding	CCAAT/enhancer binding protein (C/EBP), delta [Source:MGI Symbol;Acc:MGI:103573]	-
ENSMUSG0000000062	753.5809135	1035.361457	888.141115	1850.139172	2062.209825	2238.931523	892.3611619	2050.42684	-1.200283671	9.41E-11	5.57E-09	Hk2	6	82725025	82774454	-	5969	protein_coding	hexokinase 2 [Source:MGI Symbol;Acc:MGI:1315197]	-
ENSMUSG0000002890	2139.488365	2648.966495	2438.757301	1236.010108	1198.017489	1198.767334	2409.07072	1210.931644	0.992224164	9.73E-11	5.75E-09	Epb41	4	131923413	132075321	-	15942	protein_coding	erythrocyte membrane protein band 4.1 [Source:MGI Symbol;Acc:MGI:95401]	-
ENSMUSG0000002013	2123.454728	2697.416777	3119.10691	884.5870249	1150.610367	1109.248995	2646.659472	1048.148795	1.336479844	1.01E-10	5.96E-09	Tbc1d15	10	115197872	115251467	-	4261	protein_coding	TBC1 domain family, member 15 [Source:MGI Symbol;Acc:MGI:1913937]	-
ENSMUSG0000005616	6.012613672	0	3.351475906	197.2448186	49.38241919	86.59926361	3.121363192	111.0755005	-5.143751605	1.03E-10	6.06E-09	Cndp1	18	84610509	84650084	-	5555	protein_coding	carnosine dipeptidase 1 (metallopeptidase M20 family) [Source:MGI Symbol;Acc:MGI:2451097]	-
ENSMUSG0000002945	749.5725044	761.5120382	609.9686148	306.6338665	323.9486699	294.8267064	707.0177191	308.4697476	1.197036938	1.11E-10	6.46E-09	Acad10	5	121621026	121660514	-	6068	protein_coding	acyl-Coenzyme A dehydrogenase family, member 10 [Source:MGI Symbol;Acc:MGI:1919235]	-
ENSMUSG0000011344	183.384717	148.5106465	226.7832029	45.65054754	35.55534181	51.57034799	186.2261888	44.25874578	2.07161319	1.16E-10	6.76E-09	Gm46404	13	23441822	23445935	-	4114	lincRNA	predicted gene, 46404 [Source:MGI Symbol;Acc:MGI:5826041]	-
ENSMUSG0000001649	233.4898309	329.6725698	243.5405825	835.491153	661.7244171	679.1717528	268.9009944	725.462441	-1.432216654	1.18E-10	6.83E-09	Cd274	19	29367455	29388095	+	3622	protein_coding	CD274 antigen [Source:MGI Symbol;Acc:MGI:1926446]	-
ENSMUSG0000000536	11772.69757	8989.633812	14051.62131	3190.370341	4890.834796	4294.934265	11604.6509	4125.379801	1.492174285	1.27E-10	7.37E-09	Slc1a3	15	8634124	8710764	-	8537	protein_coding	solute carrier family 1 (glial high affinity glutamate transporter), member 3 [Source:MGI Symbol;Acc:MGI:99917]	-
ENSMUSG0000006645	178.3742056	145.3508455	194.3856025	540.9159218	409.8740793	468.9982591	172.7035512	473.2627534	-1.455312271	1.34E-10	7.72E-09	Hmgn3	9	83109948	83146685	-	1869	protein_coding	high mobility group nucleosomal binding domain 3 [Source:MGI Symbol;Acc:MGI:2138069]	-
ENSMUSG0000002025	79.16608001	24.22514091	32.39760042	280.7939339	258.7638765	340.5589018	45.26294045	293.3722374	-2.69189955	1.34E-10	7.72E-09	Glt8d2	10	82650433	82690650	-	5773	protein_coding	glycosyltransferase 8 domain containing 2 [Source:MGI Symbol;Acc:MGI:1922032]	-
ENSMUSG0000003408	961.0160852	786.7904461	971.9280126	422.913563	407.8987825	422.2930383	906.5781813	417.7017946	1.117830802	1.36E-10	7.82E-09	Ccdc174	6	91878053	91899843	+	9188	protein_coding	coiled-coil domain containing 174 [Source:MGI Symbol;Acc:MGI:2444652]	-
ENSMUSG0000002315	8647.140562	9851.206215	11859.75607	4585.7267	2894.797413	2603.816061	10119.36762	3361.446724	1.589806629	1.37E-10	7.85E-09	Ivns1abp	1	151344477	151364422	+	5435	protein_coding	influenza virus NS1A binding protein [Source:MGI Symbol;Acc:MGI:2152389]	-
ENSMUSG0000002815	589.2361398	463.4374783	538.4704622	1374.684413	1058.759067	1277.582395	530.3813601	1237.008625	-1.22181491	1.45E-10	8.25E-09	Tspan5	3	138742195	138907905	+	7389	protein_coding	tetraspanin 5 [Source:MGI Symbol;Acc:MGI:1928096]	-
ENSMUSG0000003722	559.1730715	408.6675945	376.4824601	1023.26133	1570.36093	1336.936946	448.1077087	1310.186402	-1.547037865	1.50E-10	8.55E-09	Fgf2	3	37348346	37410108	+	6782	protein_coding	fibroblast growth factor 2 [Source:MGI Symbol;Acc:MGI:95516]	-
ENSMUSG0000011015	9.018920508	9.479402965	8.937269082	66.32249359	101.7277835	72.97690753	9.145197518	80.34239488	-3.133751538	1.54E-10	8.74E-09	Gm42067	19	5912822	5914826	+	880	antisense	predicted gene, 42067 [Source:MGI Symbol;Acc:MGI:5624952]	-
ENSMUSG0000001836	190.3994329	201.1739963	244.6577411	522.827969	488.88595	515.7034799	212.0770568	509.1391329	-1.264744237	1.60E-10	9.01E-09	Kpna2	11	106988629	106999541	-	2873	protein_coding	karyopherin (importin) alpha 2 [Source:MGI Symbol;Acc:MGI:103561]	-
ENSMUSG0000002288	14348.10043	12589.7004	17671.21529	5621.046665	7331.313953	5566.678507	14869.67204	6173.013041	1.268344905	1.60E-10	9.01E-09	Eif4a2	16	23107444	23114136	+	5338	protein_coding	eukaryotic translation initiation factor 4A2 [Source:MGI Symbol;Acc:MGI:106906]	-
ENSMUSG0000003219	588.2340375	538.219435	377.5996187	1404.831001	1170.363335	1339.856022	501.3510304	1305.016786	-1.379603028	1.86E-10	1.05E-08	Gnb5	9	75306288	75345876	+	3115	protein_coding	guanine nucleotide binding protein (G protein), beta 5 [Source:MGI Symbol;Acc:MGI:101848]	-
ENSMUSG0000002262	3739.845704	3784.388317	3925.695444	1404.831001	2004.926219	1789.393773	3816.643155	1733.050331	1.139255451	1.94E-10	1.09E-08	Shank3	15	89499623	89560261	+	9189	protein_coding	SH3 and multiple ankyrin repeat domains 3 [Source:MGI Symbol;Acc:MGI:1930016]	-
ENSMUSG0000003181	6764.190381	7215.93219	9250.0735	3467.718951	3655.286668	3740.309767	7743.39869	3621.105129	1.096511851	2.09E-10	1.17E-08	Map1lc3b	8	121590361	121598760	+	7517	protein_coding	microtubule-associated protein 1 light chain 3 beta [Source:MGI Symbol;Acc:MGI:1914693]	-
ENSMUSG0000003206	398.8367069	242.2514091	618.9058839	1618.44111	1594.064491	1602.572889	419.998	1605.026164	-1.934701103	2.13E-10	1.19E-08	Cryab	9	50751325	50756636	+	1479	protein_coding	crystallin, alpha B [Source:MGI Symbol;Acc:MGI:88516]	-
ENSMUSG0000002658	137.2880122	65.30255376	81.55258037	11.19730411	11.8517806	7.78420347	94.7143821	10.27776273	3.203506957	2.14E-10	1.19E-08	Sele	1	164048234	164057677	+	2912	protein_coding	selectin, endothelial cell [Source:MGI Symbol;Acc:MGI:98278]	-
ENSMUSG0000000111	3806.986556	1854.80318	2722.515594	12152.52029	7532.794223	9467.537471	2794.768444	9717.617327	-1.797841066	2.17E-10	1.20E-08	Col6a1	10	76708792	76726168	-	4380	protein_coding	collagen, type VI, alpha 1 [Source:MGI Symbol;Acc:MGI:88459]	-
ENSMUSG0000007000	1280.686712	1276.559599	1890.232411	521.9666379	606.4161076	594.51854	1482.492907	574.3004285	1.36817815	2.30E-10	1.27E-08	Ell	8	70539457	70592858	+	3108	protein_coding	elongation factor RNA polymerase II [Source:MGI Symbol;Acc:MGI:109377]	-
ENSMUSG0000002692	2661.583652	2853.300292	1105.987049	8079.285583	8445.381329	9888.857483	2206.956998	8804.508132	-1.99604942	2.58E-10	1.42E-08	Agpat2	2	26593057	26604417	-	2130	protein_coding	1-acylglycerol-3-phosphate O-acyltransferase 2 (lysophosphatidic acid acyltransferase, beta) [Source:MGI Symbol;Acc:MGI:19147	-
ENSMUSG0000004309	232.4877286	227.5056712	319.5073697	689.0648685	623.2061301	647.0619135	259.8335898	653.1109707	-1.331016672	2.67E-10	1.46E-08	Tuba1c	15	99029891	99038110	+	2287	protein_coding	tubulin, alpha 1C [Source:MGI Symbol;Acc:MGI:1095409]	-
ENSMUSG0000006422	212.4456831	347.5781087	153.050733	689.9261996	1554.558556	1222.119945	237.6915083	1155.5349	-2.280808405	2.67E-10	1.46E-08	Paqr9	9	95559657	95568023	+	8367	protein_coding	progestin and adipoQ receptor family member IX [Source:MGI Symbol;Acc:MGI:1922802]	-
ENSMUSG0000004980	700.4694928	546.645571	863.563625	275.6259474	222.2208863	257.85174	703.5595629	251.8995246	1.480987732	2.71E-10	1.48E-08	Armcx4	X	134686519	134696757	+	7996	protein_coding	armadillo repeat containing, X-linked 4 [Source:MGI Symbol;Acc:MGI:2147887]	-
ENSMUSG0000003674	243.5108537	498.1952892	389.8883637	102.4983992	99.75248676	93.41044164	377.1981689	98.55377586	1.935642441	2.95E-10	1.60E-08	Ttll7	3	146852367	146984009	+	8266	protein_coding	tubulin tyrosine ligase-like family, member 7 [Source:MGI Symbol;Acc:MGI:1918142]	-
ENSMUSG0000002526	3493.328543	5244.216374	4054.168687	1505.606738	1773.816497	1932.428511	4263.904535	1737.283915	1.295456155	2.97E-10	1.61E-08	Hsd17b10	X	152001845	152004442	+	1298	protein_coding	hydroxysteroid (17-beta) dehydrogenase 10 [Source:MGI Symbol;Acc:MGI:1333871]	-
ENSMUSG0000007375	389.8177864	447.6384733	454.6835645	178.2955347	108.6413222	147.8998659	430.7132748	144.9455743	1.56890908	3.08E-10	1.67E-08	Sh3d21	4	126150602	126163491	-	2717	protein_coding	SH3 domain containing 21 [Source:MGI Symbol;Acc:MGI:1914188]	-
ENSMUSG0000002656	1580.315293	1216.52338	1139.501808	2919.91238	2739.736617	2987.188082	1312.113494	2882.279026	-1.135041412	3.11E-10	1.68E-08	Mpc2	1	165460637	165481214	+	7344	protein_coding	mitochondrial pyruvate carrier 2 [Source:MGI Symbol;Acc:MGI:1917706]	-
ENSMUSG0000004406	1113.335632	938.4608935	1246.749037	503.8786851	446.4170695	517.6495308	1099.515187	489.3150951	1.167710832	3.16E-10	1.70E-08	Zrsr1	11	22972005	22976496	+	4492	protein_coding	zinc finger (CCCH type), RNA binding motif and serine/arginine rich 1 [Source:MGI Symbol;Acc:MGI:98885]	-
ENSMUSG0000000353	584.2256284	554.0184399	380.9510946	1840.66453	1651.348098	1128.709503	506.3983877	1540.24071	-1.604480544	3.31E-10	1.78E-08	Ddr1	17	35681567	35704621	-	4774	protein_coding	discoidin domain receptor family, member 1 [Source:MGI Symbol;Acc:MGI:99216]	-
ENSMUSG0000004715	982.060233	1058.533331	1226.640181	440.1401847	507.6512692	536.137014	1089.077915	494.6428227	1.13891166	3.41E-10	1.82E-08	Khnyn	14	55884947	55898775	+	6502	protein_coding	KH and NYN domain containing [Source:MGI Symbol;Acc:MGI:2451333]	-
ENSMUSG0000002758	1875.935466	2042.284705	2077.915062	916.4562751	1122.956212	972.0524083	1998.711744	1003.821632	0.993791863	3.44E-10	1.83E-08	Pcmtd2	2	181837854	181857461	+	4183	protein_coding	protein-L-isoaspartate (D-aspartate) O-methyltransferase domain containing 2 [Source:MGI Symbol;Acc:MGI:1923927]	-
ENSMUSG0000002745	137.2880122	130.6051075	136.2933535	29.28525691	34.56769343	38.92101735	134.7288244	34.25798923	1.978432248	3.46E-10	1.84E-08	Sdcbp2	2	151572622	151590005	+	1399	protein_coding	syndecan binding protein (syntenin) 2 [Source:MGI Symbol;Acc:MGI:2385156]	-
ENSMUSG0000002002	4814.099346	5194.712825	6681.725797	1751.086097	2544.182237	2347.910372	5563.512656	2214.392902	1.329202525	3.54E-10	1.88E-08	Tmcc3	10	94311949	94590956	+	7153	protein_coding	transmembrane and coiled coil domains 3 [Source:MGI Symbol;Acc:MGI:2442900]	-
ENSMUSG0000001585	253.5318765	188.5347923	93.84132536	640.8303277	928.3894807	774.5282453	178.6359981	781.2493512	-2.126956999	3.69E-10	1.95E-08	Fcrls	3	87250758	87263738	-	2334	protein_coding	Fc receptor-like S, scavenger receptor [Source:MGI Symbol;Acc:MGI:1933397]	-
ENSMUSG0000007261	134.2817053	77.94175771	86.02121491	16.36529063	14.81472576	9.730254338	99.41489265	13.63675691	2.862804967	3.76E-10	1.98E-08	Gm10384	15	36870501	36879816	-	2046	lincRNA	predicted gene 10384 [Source:MGI Symbol;Acc:MGI:3641812]	-
ENSMUSG0000004979	6168.941627	5868.803702	5695.274722	10273.95719	14727.8127	14059.24449	5911.006684	13020.33813	-1.139236453	4.08E-10	2.14E-08	Fzd4	7	89404355	89413134	+	6720	protein_coding	frizzled class receptor 4 [Source:MGI Symbol;Acc:MGI:108520]	-
ENSMUSG0000004201	11139.36893	18861.90537	11932.37138	3931.976406	4861.205345	5635.763312	13977.88189	4809.648354	1.53918805	4.08E-10	2.14E-08	Acacb	5	114146535	114250761	+	9632	protein_coding	acetyl-Coenzyme A carboxylase beta [Source:MGI Symbol;Acc:MGI:2140940]	-
ENSMUSG0000000337	438.920798	387.6022546	536.2361449	186.9088456	140.2460705	121.6281792	454.2530658	149.5943651	1.600244599	4.13E-10	2.16E-08	Grik5	7	25009849	25072346	-	5168	protein_coding	glutamate receptor, ionotropic, kainate 5 (gamma 2) [Source:MGI Symbol;Acc:MGI:95818]	-
ENSMUSG0000006966	2243.707002	1678.907592	1540.561758	3793.302101	4779.230529	4431.157825	1821.058784	4334.563485	-1.250852602	4.23E-10	2.20E-08	Marcks	10	37133375	37138920	-	4048	protein_coding	myristoylated alanine rich protein kinase C substrate [Source:MGI Symbol;Acc:MGI:96907]	-
ENSMUSG0000003964	312.6559109	301.2343609	175.3939057	1109.394438	860.2417422	708.3625158	263.0947258	892.6662321	-1.761846538	4.23E-10	2.20E-08	Vasn	16	4639941	4650802	+	3007	protein_coding	vasorin [Source:MGI Symbol;Acc:MGI:2177651]	-
ENSMUSG0000002294	11.02312506	8.426135969	10.05442772	608.0997464	48.3947708	113.8439758	9.834562917	256.7794977	-4.706869013	4.69E-10	2.43E-08	Clic6	16	92485736	92541243	+	3914	protein_coding	chloride intracellular channel 6 [Source:MGI Symbol;Acc:MGI:2146607]	-
ENSMUSG0000002740	266.5592061	279.115754	75.96678719	2097.341193	786.1681135	1523.757829	207.2139158	1469.089045	-2.825004599	4.93E-10	2.55E-08	Cpxm1	2	130390775	130397574	-	2432	protein_coding	carboxypeptidase X 1 (M14 family) [Source:MGI Symbol;Acc:MGI:1934569]	-
ENSMUSG0000005753	7153.006065	9099.173579	5715.383578	3034.469415	3230.597863	2972.5927	7322.521074	3079.219993	1.249816715	5.11E-10	2.63E-08	Ece1	4	137862237	137965229	+	5300	protein_coding	endothelin converting enzyme 1 [Source:MGI Symbol;Acc:MGI:1101357]	-
ENSMUSG0000002281	2522.291435	1822.151903	2037.697351	4429.825773	4725.897516	4348.450664	2127.38023	4501.391318	-1.081155541	5.35E-10	2.75E-08	Fstl1	16	37776873	37836514	+	4914	protein_coding	follistatin-like 1 [Source:MGI Symbol;Acc:MGI:102793]	-
ENSMUSG0000002841	2075.353819	1761.062417	2277.886457	4236.026279	3841.952213	4117.843636	2038.100898	4065.274042	-0.996245297	5.50E-10	2.82E-08	Dnaja1	4	40722150	40737149	+	8629	protein_coding	DnaJ heat shock protein family (Hsp40) member A1 [Source:MGI Symbol;Acc:MGI:1270129]	-
ENSMUSG0000002932	403.8472183	829.9743929	1682.440905	122.3090142	183.7025994	154.711044	972.0875053	153.5742192	2.662451506	5.66E-10	2.88E-08	Slc10a6	5	103605711	103629403	-	2124	protein_coding	solute carrier family 10 (sodium/bile acid cotransporter family), member 6 [Source:MGI Symbol;Acc:MGI:1923000]	-
ENSMUSG0000004456	1169.453359	1427.17678	1919.278535	602.0704288	582.7125464	459.2680047	1505.302891	548.0169933	1.457305222	5.66E-10	2.88E-08	Rasip1	7	45627488	45639093	+	3202	protein_coding	Ras interacting protein 1 [Source:MGI Symbol;Acc:MGI:1917153]	-
ENSMUSG0000005122	15.03153418	3.159800988	34.63191769	236.8660485	153.0854995	225.7419006	17.60775095	205.2311496	-3.550340365	5.78E-10	2.93E-08	Fam83a	15	57985419	58011009	+	3171	protein_coding	family with sequence similarity 83, member A [Source:MGI Symbol;Acc:MGI:2447773]	-
ENSMUSG0000002837	41.08619342	35.81107787	24.57748997	144.7036224	136.295477	144.9807896	33.82492042	141.9932963	-2.06545564	5.78E-10	2.93E-08	Ptgr1	4	58965439	58987119	-	2296	protein_coding	prostaglandin reductase 1 [Source:MGI Symbol;Acc:MGI:1914353]	-
ENSMUSG0000003844	1379.894838	1440.869251	1327.184459	651.1663007	724.9339137	765.7710164	1382.649516	713.9570769	0.953967152	6.10E-10	3.08E-08	Cdc40	10	40831621	40883311	-	8196	protein_coding	cell division cycle 40 [Source:MGI Symbol;Acc:MGI:1918963]	-
ENSMUSG0000007406	1350.83372	1481.946664	1299.255493	710.5981456	679.502088	756.040762	1377.345343	715.3803319	0.945164011	6.25E-10	3.15E-08	Mlycd	8	119394878	119411102	+	2968	protein_coding	malonyl-CoA decarboxylase [Source:MGI Symbol;Acc:MGI:1928485]	-
ENSMUSG0000002256	14052.48025	13939.98869	15882.64432	6798.486259	8178.716266	8010.918396	14625.03775	7662.706974	0.932551	6.29E-10	3.16E-08	Grina	15	76246764	76249904	+	2067	protein_coding	glutamate receptor, ionotropic, N-methyl D-aspartate-associated protein 1 (glutamate binding) [Source:MGI Symbol;Acc:MGI:191	-
ENSMUSG0000005209	144.3027281	21.06533992	184.3311748	4.306655428	4.938241919	1.946050868	116.5664143	3.730316071	4.962541434	6.42E-10	3.22E-08	Prss51	14	64086234	64097678	+	1244	protein_coding	protease, serine 51 [Source:MGI Symbol;Acc:MGI:1921465]	-
ENSMUSG0000002031	21.04414785	137.9779765	103.8957531	0.861331086	0.987648384	1.946050868	87.63929247	1.265010112	6.125626095	6.56E-10	3.28E-08	Madcam1	10	79664559	79668537	+	1436	protein_coding	mucosal vascular addressin cell adhesion molecule 1 [Source:MGI Symbol;Acc:MGI:103579]	-
ENSMUSG0000006829	3029.355188	2754.293195	2344.915975	1410.860318	1164.437444	1311.638285	2709.521453	1295.645349	1.064293232	7.52E-10	3.75E-08	Ddrgk1	2	130653960	130664659	-	2084	protein_coding	DDRGK domain containing 1 [Source:MGI Symbol;Acc:MGI:1924256]	-
ENSMUSG0000004680	1128.367166	1042.734326	1346.176155	470.2867727	560.984282	563.3817262	1172.425882	531.550927	1.141516746	7.91E-10	3.94E-08	Atp10d	5	72203329	72298775	+	7848	polymorphic_pseuc	ATPase, class V, type 10D [Source:MGI Symbol;Acc:MGI:2450125]	-
ENSMUSG0000002751	71062.07888	28019.00863	65998.38069	6507.356352	16428.54322	9581.381446	55026.4894	10839.09367	2.343902865	8.43E-10	4.18E-08	Pck1	2	173153048	173159273	+	3429	protein_coding	phosphoenolpyruvate carboxykinase 1, cytosolic [Source:MGI Symbol;Acc:MGI:97501]	-
ENSMUSG0000003949	656.3769925	509.7812261	569.750904	1120.591742	1295.794679	1401.156625	578.6363742	1272.514349	-1.136482127	8.84E-10	4.37E-08	Dse	10	34151393	34207715	-	4980	protein_coding	dermatan sulfate epimerase [Source:MGI Symbol;Acc:MGI:2443455]	-
ENSMUSG0000007046	2.004204557	0	3.351475906	52.54119622	48.3947708	86.59926361	1.785226821	62.51174354	-5.14561334	9.16E-10	4.52E-08	Gm9696	3	59952309	59973594	+	3050	transcribed_unproc	predicted gene 9696 [Source:MGI Symbol;Acc:MGI:3819177]	-
ENSMUSG0000007319	399.8388092	377.0695846	397.7084741	112.8343722	154.0731479	169.3064255	391.538956	145.4046485	1.431178934	9.23E-10	4.54E-08	Bnip3l-ps	12	18216542	18217673	+	1132	transcribed_process	BCL2/adenovirus E1B interacting protein 3-like, pseudogene [Source:MGI Symbol;Acc:MGI:3642435]	-
ENSMUSG0000010496	508.0658553	469.7570803	460.2693577	229.9753999	166.9125769	191.6860105	479.3640977	196.1913291	1.287458862	9.63E-10	4.73E-08	Snhg8	3	123507552	123508404	-	853	processed_transcrip	small nucleolar RNA host gene 8 [Source:MGI Symbol;Acc:MGI:1917145]	-
ENSMUSG0000002189	2616.489049	3088.178833	2573.933496	1101.642458	1437.028398	1366.127709	2759.533793	1301.599522	1.084453746	9.87E-10	4.84E-08	Sh3bp5	14	31359880	31436078	-	3194	protein_coding	SH3-domain binding protein 5 (BTK-associated) [Source:MGI Symbol;Acc:MGI:1344391]	-
ENSMUSG0000002209	808.6965388	710.9552224	713.8643679	1782.955347	1414.312486	1461.484202	744.5053764	1552.917345	-1.060680857	1.00E-09	4.91E-08	Bmp1	14	70474558	70520234	-	8870	protein_coding	bone morphogenetic protein 1 [Source:MGI Symbol;Acc:MGI:88176]	-
ENSMUSG0000003846	288.6054562	303.3408949	223.431727	1079.24785	611.3543495	789.1236268	271.7926927	826.5752755	-1.604594722	1.02E-09	4.98E-08	Olfml2b	1	170644532	170682789	+	3095	protein_coding	olfactomedin-like 2B [Source:MGI Symbol;Acc:MGI:2443310]	-
ENSMUSG0000002243	1012.123301	875.2648738	1057.949228	443.5855091	476.046521	507.9192764	981.7791342	475.8504355	1.045167396	1.03E-09	5.00E-08	Parvb	15	84232043	84315688	+	4416	protein_coding	parvin, beta [Source:MGI Symbol;Acc:MGI:2153063]	-
ENSMUSG0000004440	5897.37191	4892.425197	5394.759049	2457.377587	3026.154648	2744.904749	5394.852052	2742.812328	0.976075051	1.03E-09	5.00E-08	Adig	2	158502612	158508198	+	810	protein_coding	adipogenin [Source:MGI Symbol;Acc:MGI:2675492]	-
ENSMUSG0000003160	259.5444902	303.3408949	353.0221287	1017.232012	645.922043	843.6130511	305.3025046	835.5890354	-1.453612502	1.11E-09	5.35E-08	Msmo1	8	64718139	64733792	-	6144	protein_coding	methylsterol monoxygenase 1 [Source:MGI Symbol;Acc:MGI:1913484]	-
ENSMUSG0000005896	88.18500052	86.36789368	79.3182631	220.5007579	269.6280088	227.6879515	84.6237191	239.2722394	-1.498247098	1.12E-09	5.39E-08	Fam57b	7	126797668	126830219	+	3935	protein_coding	family with sequence similarity 57, member B [Source:MGI Symbol;Acc:MGI:1916202]	-
ENSMUSG0000003153	997.0917672	1054.320263	1124.978746	566.7558543	554.0707433	551.705421	1058.796925	557.5106729	0.925060677	1.12E-09	5.39E-08	Smim19	8	22462614	22476882	-	1462	protein_coding	small integral membrane protein 19 [Source:MGI Symbol;Acc:MGI:2142501]	-
ENSMUSG0000002077	331.6958542	357.0575117	245.7748997	705.4301591	1333.325318	973.9984592	311.5094219	1004.251312	-1.687979775	1.15E-09	5.52E-08	Aspa	11	73304992	73329596	-	5954	protein_coding	aspartoacylase [Source:MGI Symbol;Acc:MGI:87914]	-
ENSMUSG0000000680	2680.623595	1392.418969	2017.588495	5069.79477	6014.778657	6153.412843	2030.210353	5745.995423	-1.500811025	1.18E-09	5.63E-08	Sulf2	2	166073089	166155663	-	5871	protein_coding	sulfatase 2 [Source:MGI Symbol;Acc:MGI:1919293]	-
ENSMUSG0000004264	923.9383009	797.323116	673.646657	301.46588	380.2446277	288.9885538	798.3026913	323.5663538	1.303656978	1.20E-09	5.72E-08	Acad12	5	121596775	121618938	-	6516	protein_coding	acyl-Coenzyme A dehydrogenase family, member 12 [Source:MGI Symbol;Acc:MGI:2443320]	-
ENSMUSG0000002169	341.716877	480.2897502	437.926185	89.5784329	155.0607962	141.0886879	419.9776041	128.5759723	1.709769717	1.24E-09	5.90E-08	Pde4d	13	108449948	109953461	+	12006	protein_coding	phosphodiesterase 4D, cAMP specific [Source:MGI Symbol;Acc:MGI:99555]	-
ENSMUSG0000002779	133.2796031	64.24928676	83.78689764	310.0791908	337.7757472	399.9134533	93.77192915	349.2561304	-1.89484887	1.28E-09	6.09E-08	Dclk1	3	55242364	55539068	+	15296	protein_coding	doublecortin-like kinase 1 [Source:MGI Symbol;Acc:MGI:1330861]	-
ENSMUSG0000002908	182.3826147	267.529817	327.3274801	72.35181119	78.02422232	63.2466532	259.0799706	71.20756223	1.862315987	1.29E-09	6.13E-08	Bst1	5	43818885	43843986	+	3317	protein_coding	bone marrow stromal cell antigen 1 [Source:MGI Symbol;Acc:MGI:105370]	-
ENSMUSG0000003897	1141.394495	1270.239997	1429.963053	574.5078341	677.5267912	587.707362	1280.532515	613.2473291	1.06227857	1.30E-09	6.16E-08	Rabggtb	3	153907287	153913009	-	2421	protein_coding	Rab geranylgeranyl transferase, b subunit [Source:MGI Symbol;Acc:MGI:99537]	-
ENSMUSG0000003610	2108.423194	2138.132002	2753.796036	1031.874641	1134.807993	1170.549597	2333.450411	1112.410743	1.068783063	1.40E-09	6.60E-08	Rab3gap1	1	127868773	127943868	+	11739	protein_coding	RAB3 GTPase activating protein subunit 1 [Source:MGI Symbol;Acc:MGI:2445001]	-
ENSMUSG0000004469	230.4835241	531.899833	687.0525607	98.19174376	111.6042674	81.73413644	483.1453059	97.17671585	2.313291717	1.45E-09	6.82E-08	2010007H06Ri	9	51278513	51284210	+	4934	antisense	RIKEN cDNA 2010007H06 gene [Source:MGI Symbol;Acc:MGI:1917099]	-
ENSMUSG0000007457	358.7526157	374.9630506	489.3154822	169.6822239	148.1472576	126.4933064	407.6770495	148.1075959	1.459011166	1.47E-09	6.88E-08	Zfas1	2	167062934	167065862	+	2929	processed_transcrip	zinc finger, NFX1-type containing 1, antisense RNA 1 [Source:MGI Symbol;Acc:MGI:1916199]	-
ENSMUSG0000002407	3242.802974	3041.835085	2947.06448	1614.995785	1834.063049	1587.977508	3077.234179	1679.012114	0.874184322	1.50E-09	7.01E-08	Crim1	17	78200248	78376592	+	5995	protein_coding	cysteine rich transmembrane BMP regulator 1 (chordin like) [Source:MGI Symbol;Acc:MGI:1354756]	-
ENSMUSG0000001625	3776.923488	5049.361979	4477.57181	2221.37287	2073.073957	2311.908431	4434.619092	2202.118419	1.009854028	1.67E-09	7.79E-08	Ctsz	2	174427493	174439039	-	1441	protein_coding	cathepsin Z [Source:MGI Symbol;Acc:MGI:1891190]	-
ENSMUSG0000000080	2643.545811	1780.021223	5212.662192	728.6860984	807.8963779	795.9348048	3212.076409	777.5057604	2.046547761	1.68E-09	7.80E-08	Car4	11	84957786	84966044	+	1966	protein_coding	carbonic anhydrase 4 [Source:MGI Symbol;Acc:MGI:1096574]	-
ENSMUSG0000002304	23.04835241	9.479402965	14.52306226	139.5356359	76.04892555	126.4933064	15.68360588	114.0259559	-2.858342454	1.68E-09	7.82E-08	Amhr2	15	102445367	102454633	+	2005	protein_coding	anti-Mullerian hormone type 2 receptor [Source:MGI Symbol;Acc:MGI:105062]	-
ENSMUSG0000002885	524.0994917	472.9168812	210.0258234	73.21314228	104.6907287	85.62623817	402.3473988	87.84336971	2.197208736	1.77E-09	8.18E-08	Csf3r	4	126024550	126044440	+	3609	protein_coding	colony stimulating factor 3 receptor (granulocyte) [Source:MGI Symbol;Acc:MGI:1339755]	-
ENSMUSG0000002227	369.7757408	863.6789368	1443.368957	199.8288119	85.92540939	73.94993297	892.2745448	119.9013847	2.893554648	1.79E-09	8.25E-08	Retreg1	15	25843180	25973687	+	4142	protein_coding	reticulophagy regulator 1 [Source:MGI Symbol;Acc:MGI:1913520]	-
ENSMUSG0000009749	102.2144324	168.5227194	192.1512853	28.42392582	26.66650636	38.92101735	154.2961457	31.33714985	2.299760195	1.79E-09	8.25E-08	4933406C10Ri	12	32919384	32953789	-	2569	lincRNA	RIKEN cDNA 4933406C10 gene [Source:MGI Symbol;Acc:MGI:1921326]	-
ENSMUSG0000002512	97.20392103	121.1257046	208.9086648	17.22662171	30.6170999	20.43353411	142.4127635	22.75908524	2.648684696	1.85E-09	8.50E-08	Gcgr	11	120530699	120538986	+	2621	protein_coding	glucagon receptor [Source:MGI Symbol;Acc:MGI:99572]	-
ENSMUSG0000009149	697.4631859	623.5340617	569.750904	1191.220891	2065.17277	1872.100935	630.2493839	1709.498199	-1.439113323	1.92E-09	8.83E-08	Mpc1-ps	12	110858565	110858894	-	330	processed_pseudog	mitochondrial pyruvate carrier 1, pseudogene [Source:MGI Symbol;Acc:MGI:3781628]	-
ENSMUSG0000000657	84.1765914	36.86434486	58.09224903	4.306655428	3.950593535	1.946050868	59.71106177	3.401099943	4.126331468	2.08E-09	9.50E-08	Slc4a1	11	102348824	102366203	-	4891	protein_coding	solute carrier family 4 (anion exchanger), member 1 [Source:MGI Symbol;Acc:MGI:109393]	-
ENSMUSG0000002210	86.18079596	46.34374783	34.63191769	221.362089	275.5538991	257.85174	55.71882049	251.5892427	-2.170119836	2.12E-09	9.65E-08	Gfra2	14	70890120	70979838	+	3539	protein_coding	glial cell line derived neurotrophic factor family receptor alpha 2 [Source:MGI Symbol;Acc:MGI:1195462]	-
ENSMUSG0000003563	2004.204557	1450.348654	1616.528545	838.9364774	633.082614	724.9039482	1690.360585	732.3076798	1.206525101	2.21E-09	1.01E-07	Grhpr	4	44981395	44990734	+	1603	protein_coding	glyoxylate reductase/hydroxypyruvate reductase [Source:MGI Symbol;Acc:MGI:1923488]	-
ENSMUSG0000009988	1076.257847	781.5241111	2165.053435	279.0712717	324.9363183	354.1812579	1340.945131	319.3962826	2.069869994	2.24E-09	1.02E-07	2810013P06Ril	8	123042466	123044602	+	2137	bidirectional_prom	RIKEN cDNA 2810013P06 gene [Source:MGI Symbol;Acc:MGI:1914456]	-
ENSMUSG0000002964	7112.921974	5639.191497	6629.219341	2207.591572	3335.288592	2860.694775	6460.444271	2801.191647	1.205755923	2.24E-09	1.02E-07	Flt1	5	147561604	147726011	-	13504	protein_coding	FMS-like tyrosine kinase 1 [Source:MGI Symbol;Acc:MGI:95558]	-
ENSMUSG0000007491	395.8304001	413.9339295	344.0848596	852.7177747	791.1063554	743.3914314	384.6163964	795.7385205	-1.048657478	2.31E-09	1.04E-07	Inafm2	2	118745758	118748810	+	3053	protein_coding	InaF motif containing 2 [Source:MGI Symbol;Acc:MGI:1915354]	-
ENSMUSG0000011058	6.012613672	5.26633498	5.585793176	49.95720296	55.30830949	86.59926361	5.621580609	63.95492535	-3.505920877	2.38E-09	1.07E-07	D830024N08R	8	83576044	83585919	+	2899	antisense	RIKEN cDNA D830024N08 gene [Source:MGI Symbol;Acc:MGI:2442966]	-
ENSMUSG0000000641	1631.42251	1647.309582	1698.081126	961.2454915	925.4265356	955.510976	1658.937739	947.3943344	0.808095501	2.43E-09	1.09E-07	Rnf114	2	167492645	167516173	+	3356	protein_coding	ring finger protein 114 [Source:MGI Symbol;Acc:MGI:1933159]	-
ENSMUSG0000003703	1383.903247	1382.939566	1169.665091	653.750294	712.0944847	663.6033458	1312.169301	676.4827082	0.956195144	2.50E-09	1.12E-07	Tspan15	10	62185396	62231251	-	8183	protein_coding	tetraspanin 15 [Source:MGI Symbol;Acc:MGI:1917673]	-
ENSMUSG0000002886	1748.668476	2430.940227	4131.252633	831.1844976	721.9709685	877.6689413	2770.287112	810.2748025	1.773372682	2.52E-09	1.13E-07	Map3k6	4	133240818	133252929	+	4604	protein_coding	mitogen-activated protein kinase kinase kinase 6 [Source:MGI Symbol;Acc:MGI:1855691]	-
ENSMUSG0000002205	4820.11196	4865.040255	4795.962021	2712.331589	2478.997443	2951.186141	4827.038079	2714.171724	0.830613528	2.60E-09	1.16E-07	Bnip3l	14	66985239	67008877	-	3859	protein_coding	BCL2/adenovirus E1B interacting protein 3-like [Source:MGI Symbol;Acc:MGI:1332659]	-
ENSMUSG0000002509	949.9929601	476.0766822	632.3117875	1625.331759	2773.316662	2693.334401	686.1271433	2363.994274	-1.784295664	2.69E-09	1.20E-07	Hspa12a	19	58795751	58860984	-	5733	protein_coding	heat shock protein 12A [Source:MGI Symbol;Acc:MGI:1920692]	-
ENSMUSG0000010311	16.03363646	5.26633498	16.75737953	68.90648685	123.456048	134.2775099	12.68578366	108.8800149	-3.1012855	2.70E-09	1.20E-07	Gm32200	1	191436340	191465048	-	2385	lincRNA	predicted gene, 32200 [Source:MGI Symbol;Acc:MGI:5591359]	-
ENSMUSG0000000661	809.6986411	767.8316402	507.1900204	1472.014825	2186.653522	2240.877574	694.9067672	1966.515307	-1.500207104	2.77E-09	1.23E-07	Hfe	13	23702034	23710854	-	3572	protein_coding	hemochromatosis [Source:MGI Symbol;Acc:MGI:109191]	-
ENSMUSG0000002230	421.8850593	261.210215	191.0341266	985.3627619	863.2046874	1046.002341	291.376467	964.8565969	-1.726274083	2.93E-09	1.30E-07	Angpt1	15	42424723	42676977	-	5205	protein_coding	angiopoietin 1 [Source:MGI Symbol;Acc:MGI:108448]	-
ENSMUSG0000002274	1414.968417	1926.425336	979.7481231	498.7106986	533.3301272	447.5916995	1440.380625	493.2108418	1.546306648	2.98E-09	1.32E-07	St3gal6	16	58468125	58524243	-	6172	protein_coding	ST3 beta-galactoside alpha-2,3-sialyltransferase 6 [Source:MGI Symbol;Acc:MGI:1888707]	-
ENSMUSG0000006362	77.16187545	30.54474289	87.13837355	249.7860148	327.8992634	351.2621816	64.94833063	309.6491533	-2.253712236	3.04E-09	1.34E-07	Gm7665	18	16274648	16274944	-	297	processed_pseudog	predicted pseudogene 7665 [Source:MGI Symbol;Acc:MGI:3645651]	-
ENSMUSG0000002790	1622.403589	2051.764108	1752.821899	581.3984828	692.341517	923.4011367	1808.996532	732.3803788	1.304912996	3.17E-09	1.39E-07	Dennd2d	3	106482405	106503030	+	6868	protein_coding	DENN/MADD domain containing 2D [Source:MGI Symbol;Acc:MGI:2181193]	-
ENSMUSG0000004248	300.6306836	322.2997008	325.0931628	602.9317599	643.9467462	725.8769736	316.0078491	657.5851599	-1.05719361	3.20E-09	1.40E-07	Leo1	9	75441524	75466432	+	4846	protein_coding	Leo1, Paf1/RNA polymerase II complex component [Source:MGI Symbol;Acc:MGI:2685031]	-
ENSMUSG0000002245	6.012613672	4.213067984	3.351475906	220.5007579	54.32066111	41.84009365	4.525719187	105.5538376	-4.538986503	3.39E-09	1.48E-07	Sept3	15	82274893	82294574	+	6620	protein_coding	septin 3 [Source:MGI Symbol;Acc:MGI:1345148]	-

ENSMUSG0000001833	90322.48468	110165.4082	56563.97602	28092.31336	35291.6397	23939.34475	85683.9563	29107.76593	1.557625361	3.41E-09	1.49E-07	Gpx3	11	54902453	54910377	+	1813	protein_coding	glutathione peroxidase 3 [Source:MGI Symbol;Acc:MGI:105102]	-
ENSMUSG0000003856	264.5550016	218.0262682	268.1180725	60.29317599	90.8636513	90.49136534	250.2331141	80.54939755	1.638326095	3.44E-09	1.50E-07	Rad9b	5	122323223	122354233	-	4322	protein_coding	RAD9 checkpoint clamp component B [Source:MGI Symbol;Acc:MGI:2385231]	-
ENSMUSG0000003486	399.8388092	426.5731334	264.7665965	972.4427956	1050.85788	828.0446441	363.7261797	950.44844	-1.385039627	3.49E-09	1.51E-07	Ano8	8	71476019	71485963	-	6847	protein_coding	anoctamin 8 [Source:MGI Symbol;Acc:MGI:2687327]	-
ENSMUSG0000002722	157.3300577	94.79402965	74.84962856	342.8097721	496.787137	382.3989955	108.9912387	407.3319682	-1.899244169	3.62E-09	1.57E-07	Mapk8ip1	2	92383676	92401263	-	4644	protein_coding	mitogen-activated protein kinase 8 interacting protein 1 [Source:MGI Symbol;Acc:MGI:1309464]	-
ENSMUSG0000000620	2175.564047	1250.227924	1676.855111	4775.219539	4009.852438	4093.518	1700.882361	4292.863325	-1.335582626	3.63E-09	1.57E-07	Htra1	7	130936111	130985660	+	2879	protein_coding	HtrA serine peptidase 1 [Source:MGI Symbol;Acc:MGI:1929076]	-
ENSMUSG0000003097	570.1961965	357.0575117	494.9012754	131.7836561	182.714951	172.2255018	474.0516612	162.2413696	1.548384712	3.69E-09	1.59E-07	Acsm5	7	119519463	119545551	+	3799	protein_coding	acyl-CoA synthetase medium-chain family member 5 [Source:MGI Symbol;Acc:MGI:2444086]	-
ENSMUSG0000008521	53.11142077	61.08948577	43.56918677	152.4556021	187.6531929	244.2293839	52.5900311	194.779393	-1.886826284	3.94E-09	1.70E-07	B430219N15R	10	7780185	7781357	+	1173	antisense	RIKEN cDNA B430219N15 gene [Source:MGI Symbol;Acc:MGI:3697426]	-
ENSMUSG0000009157	2448.135867	1574.634159	3389.459299	486.6520634	603.4531625	921.4550858	2470.743108	670.5201039	1.881809688	4.00E-09	1.72E-07	2010016I18Rik	3	106481982	106485913	-	3697	processed_transcrip	RIKEN cDNA 2010016I18 gene [Source:MGI Symbol;Acc:MGI:1916456]	-
ENSMUSG0000002195	370.7778431	338.0987057	508.307179	164.5142373	130.3695867	111.8979249	405.7279093	135.5939163	1.579123953	4.01E-09	1.72E-07	Anxa8	14	34085981	34100571	+	1880	protein_coding	annexin A8 [Source:MGI Symbol;Acc:MGI:1201374]	-
ENSMUSG0000005629	19.03994329	23.17187391	17.87453816	99.91440593	81.97481585	129.4123827	20.02878512	103.7672015	-2.372432216	4.02E-09	1.72E-07	Ms4a4b	19	11443553	11463549	+	5274	protein_coding	membrane-spanning 4-domains, subfamily A, member 4B [Source:MGI Symbol;Acc:MGI:1913083]	-
ENSMUSG0000002667	1231.5837	1559.888421	1557.319137	606.3770843	642.9590978	767.7170673	1449.597086	672.3510831	1.108487116	4.12E-09	1.76E-07	Optn	2	5020642	5064051	-	3753	protein_coding	optineurin [Source:MGI Symbol;Acc:MGI:1918898]	-
ENSMUSG0000002012	155.3258532	124.2855055	103.8957531	429.8042117	322.9610215	339.5858764	127.8357039	364.1170365	-1.509043967	4.14E-09	1.76E-07	Avpr1a	10	122448499	122453452	+	2670	protein_coding	arginine vasopressin receptor 1A [Source:MGI Symbol;Acc:MGI:1859216]	-
ENSMUSG0000006991	3421.177179	973.2187044	3797.222201	565.0331921	387.1581664	582.8422348	2730.539362	511.6778645	2.415742701	4.18E-09	1.77E-07	Hba-a2	11	32296489	32297298	+	667	protein_coding	hemoglobin alpha, adult chain 2 [Source:MGI Symbol;Acc:MGI:96016]	-
ENSMUSG0000002490	72.15136406	124.2855055	54.74077313	12.0586352	6.913538686	3.892101735	83.72588091	7.621425207	3.444089546	4.34E-09	1.84E-07	Lao1	4	118961578	118968912	+	2538	protein_coding	L-amino acid oxidase 1 [Source:MGI Symbol;Acc:MGI:2140628]	-
ENSMUSG0000001918	875.8373915	897.3834807	624.4916771	1663.230326	1789.618871	1827.341765	799.2375164	1760.063654	-1.138454025	4.39E-09	1.85E-07	Rnf145	11	44518964	44565520	+	7786	protein_coding	ring finger protein 145 [Source:MGI Symbol;Acc:MGI:1921565]	-
ENSMUSG0000003782	684.4358563	768.8849072	795.4169483	248.0633527	377.2816826	241.3103076	749.5792372	288.8851143	1.376279191	4.47E-09	1.88E-07	Ppm1k	6	57506502	57535468	-	6022	protein_coding	protein phosphatase 1K (PP2C domain containing) [Source:MGI Symbol;Acc:MGI:2442111]	-
ENSMUSG0000005461	5.010511393	4.213067984	6.702951811	102.4983992	40.49358373	65.19270406	5.30884373	69.39489566	-3.715049349	4.47E-09	1.88E-07	Gm9951	8	34049262	34057046	+	1854	lincRNA	predicted gene 9951 [Source:MGI Symbol;Acc:MGI:3641827]	-
ENSMUSG0000003259	2633.524788	2755.346462	2547.121688	1587.433191	1531.842643	1432.293439	2645.330979	1517.189757	0.801958224	4.65E-09	1.95E-07	Ip6k1	9	108002501	108048782	+	6487	protein_coding	inositol hexaphosphate kinase 1 [Source:MGI Symbol;Acc:MGI:1351633]	-
ENSMUSG0000003916	4119.642467	4701.783871	3840.791388	1754.531421	2344.677263	2071.571149	4220.739242	2056.926611	1.037218339	4.73E-09	1.98E-07	Adgrl4	3	151437887	151545086	+	4585	protein_coding	adhesion G protein-coupled receptor L4 [Source:MGI Symbol;Acc:MGI:2655562]	-
ENSMUSG0000003203	1342.817053	1083.811739	878.0866873	504.7400162	442.4664759	457.3219539	1101.571827	468.1761487	1.234473399	4.93E-09	2.06E-07	St3gal4	9	35046576	35119268	-	2923	protein_coding	ST3 beta-galactoside alpha-2,3-sialyltransferase 4 [Source:MGI Symbol;Acc:MGI:1316743]	-
ENSMUSG0000010371	89.1871028	110.5930346	246.8920584	14.64262846	27.65415474	13.62235607	148.8907319	18.63971309	3.000415102	4.97E-09	2.07E-07	4933431K14Ri	9	85749078	85750429	+	1352	TEC	RIKEN cDNA 4933431K14 gene [Source:MGI Symbol;Acc:MGI:1918545]	-
ENSMUSG0000004676	312.6559109	182.2151903	309.452942	710.5981456	707.1562428	954.5379505	268.1080144	790.764113	-1.560357403	4.97E-09	2.07E-07	A530053G22R	6	60279043	60403707	-	5085	processed_transcrip	RIKEN cDNA A530053G22 gene [Source:MGI Symbol;Acc:MGI:2443347]	-
ENSMUSG0000002262	1254.632053	1065.9062	1164.079298	566.7558543	634.0702624	617.8711504	1161.539184	606.2324224	0.938519632	5.02E-09	2.08E-07	Arsa	15	89472476	89477425	-	3680	protein_coding	arylsulfatase A [Source:MGI Symbol;Acc:MGI:88077]	-
ENSMUSG0000004786	1717.603306	2110.74706	1700.315443	983.6400997	947.1548	923.4011367	1842.888603	951.3986788	0.953766225	5.16E-09	2.13E-07	Gimap6	6	48701582	48708225	-	3496	protein_coding	GTPase, IMAP family member 6 [Source:MGI Symbol;Acc:MGI:1918876]	-
ENSMUSG0000002878	1.002102279	11.58593696	1.117158635	548.6679015	177.7767091	330.8286475	4.56839929	352.4244194	-6.268811468	5.24E-09	2.17E-07	Hpca	4	129111570	129122036	-	2922	protein_coding	hippocalcin [Source:MGI Symbol;Acc:MGI:1336200]	-
ENSMUSG0000002908	1628.416203	2113.906861	1941.621708	824.2938489	1012.339593	919.5090349	1894.648257	918.7141591	1.044436191	5.26E-09	2.17E-07	Cd38	5	43868553	43912375	+	3890	protein_coding	CD38 antigen [Source:MGI Symbol;Acc:MGI:107474]	-
ENSMUSG0000008623	35.07357975	15.79900494	5.585793176	206.7194605	113.5795641	193.6320613	18.81945929	171.310362	-3.177290474	5.41E-09	2.22E-07	Gm11816	4	9283972	9287391	-	984	antisense	predicted gene 11816 [Source:MGI Symbol;Acc:MGI:3650294]	-
ENSMUSG0000002508	6493.622765	7194.86685	7528.532043	3208.458294	4111.580222	3736.417666	7072.340553	3685.485394	0.940414871	5.48E-09	2.25E-07	Ablim1	19	57032733	57314919	-	11949	protein_coding	actin-binding LIM protein 1 [Source:MGI Symbol;Acc:MGI:1194500]	-
ENSMUSG0000002877	4973.433609	3018.663211	6243.799612	1849.277841	1477.521982	1540.299262	4745.298811	1622.366362	1.548264835	5.49E-09	2.25E-07	Tinagl1	4	130164454	130175122	-	4358	protein_coding	tubulointerstitial nephritis antigen-like 1 [Source:MGI Symbol;Acc:MGI:2137617]	-
ENSMUSG0000003498	303.6369904	304.3941619	226.7832029	776.0593081	1131.845048	612.0329978	278.2714517	839.9791179	-1.593197241	5.56E-09	2.27E-07	Parm1	5	91517615	91626894	+	5068	protein_coding	prostate androgen-regulated mucin-like protein 1 [Source:MGI Symbol;Acc:MGI:2443349]	-
ENSMUSG0000003694	819.7196639	890.0106117	937.2960949	467.7027795	474.0712242	450.5107758	882.3421235	464.0949265	0.926653731	5.56E-09	2.27E-07	Elac1	18	73735038	73754479	-	4971	protein_coding	elaC ribonuclease Z 1 [Source:MGI Symbol;Acc:MGI:1890495]	-
ENSMUSG0000003182	580.2172193	610.8948577	485.9640063	1099.058465	1074.561442	1131.628579	559.0253611	1101.749495	-0.978389117	5.62E-09	2.29E-07	Cotl1	8	119809222	119840544	-	1596	protein_coding	coactosin-like 1 (Dictyostelium) [Source:MGI Symbol;Acc:MGI:1919292]	-
ENSMUSG0000000173	1607.372055	1527.237144	2915.784038	603.793091	751.60042	651.9270406	2016.797746	669.1068506	1.591752223	5.83E-09	2.37E-07	Cldn15	5	136966616	136975858	+	2124	protein_coding	claudin 15 [Source:MGI Symbol;Acc:MGI:1913103]	-
ENSMUSG0000003102	590.2382421	604.5752558	456.9178818	1204.140858	1217.770457	1066.435875	550.5771266	1162.782397	-1.078119966	6.01E-09	2.43E-07	St5	7	109523911	109703605	-	5738	protein_coding	suppression of tumorigenicity 5 [Source:MGI Symbol;Acc:MGI:108517]	-
ENSMUSG0000003833	1945.080523	2356.15827	2648.783124	1198.11154	1149.622719	1065.46285	2316.673972	1137.73237	1.025623562	6.18E-09	2.50E-07	Sesn1	10	41809935	41908424	+	3982	protein_coding	sestrin 1 [Source:MGI Symbol;Acc:MGI:2155278]	-
ENSMUSG0000003208	178.3742056	1620.977907	645.7176912	27.56259474	6.913538686	11.67630521	815.0232679	15.38414621	5.723162693	6.32E-09	2.55E-07	Apoc3	9	46232933	46235636	-	1014	protein_coding	apolipoprotein C-III [Source:MGI Symbol;Acc:MGI:88055]	-
ENSMUSG0000003515	707.4842087	1026.935321	679.2324502	325.5831504	314.072186	341.5319273	804.55066	327.0624212	1.298677662	6.38E-09	2.57E-07	Mitf	6	97807052	98021349	+	6196	protein_coding	melanogenesis associated transcription factor [Source:MGI Symbol;Acc:MGI:104554]	bHLI
ENSMUSG0000003491	3221.758826	2796.423875	3395.045092	1703.712887	1783.692981	1688.199128	3137.742598	1725.201665	0.86295727	6.47E-09	2.60E-07	Ushbp1	8	71384272	71395802	-	6461	protein_coding	USH1 protein network component harmonin binding protein 1 [Source:MGI Symbol;Acc:MGI:1922920]	-
ENSMUSG0000002194	791.6608001	495.0354882	229.0175202	3608.977249	1616.780404	2561.002942	505.2379362	2595.586865	-2.360597817	6.78E-09	2.72E-07	Gdf10	14	33923587	33937983	+	5210	protein_coding	growth differentiation factor 10 [Source:MGI Symbol;Acc:MGI:95684]	-
ENSMUSG0000007141	5927.434978	5644.457832	5943.283939	3459.10564	2481.960388	2586.301603	5838.39225	2842.455877	1.038241452	6.85E-09	2.74E-07	Rpl23	11	97777527	97782437	-	1770	protein_coding	ribosomal protein L23 [Source:MGI Symbol;Acc:MGI:1929455]	-
ENSMUSG0000009015	3835.04542	2859.619894	5262.934331	1191.220891	1499.250247	1662.900466	3985.866548	1451.123868	1.457810885	7.17E-09	2.86E-07	Acad11	9	104063377	104127725	+	9798	protein_coding	acyl-Coenzyme A dehydrogenase family, member 11 [Source:MGI Symbol;Acc:MGI:2143169]	-
ENSMUSG0000006338	1051.20529	1090.131341	684.8182434	2395.361749	2228.134754	2094.923759	942.0516249	2239.473421	-1.248981213	7.21E-09	2.87E-07	Bcl9l	9	44482825	44511896	+	7998	protein_coding	B cell CLL/lymphoma 9-like [Source:MGI Symbol;Acc:MGI:1933114]	-
ENSMUSG0000003842	38.07988659	32.65127688	23.46033134	155.0395954	123.456048	114.8170012	31.39716494	131.1042149	-2.059075476	7.40E-09	2.94E-07	Hdhd3	4	62499008	62503467	-	1366	protein_coding	haloacid dehalogenase-like hydrolase domain containing 3 [Source:MGI Symbol;Acc:MGI:1919998]	-
ENSMUSG0000003165	356.7484112	326.5127688	225.6660443	770.0299905	740.7362878	707.3894904	302.9757414	739.3852562	-1.286118373	7.47E-09	2.96E-07	Adcy7	8	88272403	88329962	+	6733	protein_coding	adenylate cyclase 7 [Source:MGI Symbol;Acc:MGI:102891]	-
ENSMUSG0000003675	1843.868193	1230.215851	2128.1872	5026.728216	3894.297577	4284.230985	1734.090415	4401.752259	-1.34402824	7.51E-09	2.97E-07	Tubb4b	2	25222160	25224702	-	2088	protein_coding	tubulin, beta 4B class IVB [Source:MGI Symbol;Acc:MGI:1915472]	-
ENSMUSG0000005442	10.02102279	4.213067984	11.17158635	66.32249359	60.24655141	92.43741621	8.468559041	73.00215374	-3.109617574	8.05E-09	3.18E-07	Cadps	14	12372563	12823079	-	15054	protein_coding	Ca2+-dependent secretion activator [Source:MGI Symbol;Acc:MGI:1350922]	-
ENSMUSG0000003764	232.4877286	103.2201656	113.9501808	573.646503	508.6389176	549.7593701	149.886025	544.0149302	-1.858069834	8.48E-09	3.34E-07	H2-DMa	17	34135182	34139101	+	1641	protein_coding	histocompatibility 2, class II, locus DMa [Source:MGI Symbol;Acc:MGI:95921]	-
ENSMUSG0000003388	2669.60047	2329.826595	1982.956578	5963.856437	4406.887088	4757.121346	2327.461214	5042.621624	-1.115366838	8.54E-09	3.36E-07	Lgals3bp	11	118392751	118402092	-	2847	protein_coding	lectin, galactoside-binding, soluble, 3 binding protein [Source:MGI Symbol;Acc:MGI:99554]	-
ENSMUSG0000002040	176.370001	302.2876279	394.3569982	80.96512205	74.07362878	50.59732256	291.0048757	68.54535779	2.08376644	8.62E-09	3.38E-07	Upp1	11	9118103	9136170	+	1961	protein_coding	uridine phosphorylase 1 [Source:MGI Symbol;Acc:MGI:1097668]	-
ENSMUSG0000005713	501.0511393	557.1782409	776.4252515	174.8502104	237.0356121	250.0675365	611.5515439	220.6511197	1.471348076	9.54E-09	3.74E-07	Tmem140	6	34863146	34874946	+	5853	protein_coding	transmembrane protein 140 [Source:MGI Symbol;Acc:MGI:1915737]	-
ENSMUSG0000006345	10161.31711	8801.099019	9682.413891	5644.302604	4244.912753	4876.803474	9548.276672	4922.006277	0.955910041	9.75E-09	3.80E-07	Rps15	10	80292453	80294114	+	630	protein_coding	ribosomal protein S15 [Source:MGI Symbol;Acc:MGI:98117]	-
ENSMUSG0000000553	6858.387995	7641.452057	6981.124311	2319.564613	3497.262927	3639.115122	7160.321454	3151.980888	1.18391822	9.76E-09	3.80E-07	Insr	8	3122061	3279617	-	13746	protein_coding	insulin receptor [Source:MGI Symbol;Acc:MGI:96575]	-
ENSMUSG0000003927	738.5493793	647.7592026	506.0728618	1207.586182	2248.87537	2138.709903	630.7938146	1865.057152	-1.563473079	9.95E-09	3.87E-07	Megf9	4	70427065	70534995	-	8226	protein_coding	multiple EGF-like-domains 9 [Source:MGI Symbol;Acc:MGI:1918264]	-
ENSMUSG0000002283	1432.004156	1739.997078	1872.357873	4373.839253	2983.685767	4035.136474	1681.453035	3797.553831	-1.175585055	1.05E-08	4.06E-07	Mylk	16	34745210	35002420	+	11263	protein_coding	myosin, light polypeptide kinase [Source:MGI Symbol;Acc:MGI:894806]	-
ENSMUSG0000001989	1224.568984	657.2386056	691.5211952	2140.407748	2593.564656	2584.355552	857.7762617	2439.442652	-1.507439633	1.06E-08	4.09E-07	Lama2	10	26980036	27619758	-	16338	protein_coding	laminin, alpha 2 [Source:MGI Symbol;Acc:MGI:99912]	-
ENSMUSG0000003139	49.10301165	38.97087886	39.10055223	129.1996628	170.8631704	144.0077642	42.39148091	148.0235325	-1.801276451	1.06E-08	4.10E-07	Plxna3	X	74329066	74344689	+	7880	protein_coding	plexin A3 [Source:MGI Symbol;Acc:MGI:107683]	-
ENSMUSG0000002241	1106.320916	1022.722253	1183.070995	1915.600334	3261.214963	3033.893303	1104.038055	2736.902867	-1.309635396	1.10E-08	4.24E-07	Deptor	15	55112317	55259271	+	8874	protein_coding	DEP domain containing MTOR-interacting protein [Source:MGI Symbol;Acc:MGI:2146322]	-
ENSMUSG0000003998	244.512956	259.103681	224.5488857	529.7186176	468.1453339	525.4337342	242.7218409	507.7658953	-1.06471174	1.10E-08	4.24E-07	Cbx4	11	119077573	119086221	-	5210	protein_coding	chromobox 4 [Source:MGI Symbol;Acc:MGI:1195985]	-
ENSMUSG0000003107	98.2060233	85.31462668	72.61531129	223.0847512	225.1838315	246.1754347	85.37865376	231.4813391	-1.436818555	1.11E-08	4.28E-07	Mrgprf	7	145300828	145309557	+	2214	protein_coding	MAS-related GPR, member F [Source:MGI Symbol;Acc:MGI:2384823]	-
ENSMUSG0000003098	769.61455	845.7733979	490.4326409	1514.220048	2141.221696	2036.542233	701.9401962	1897.327992	-1.434051537	1.13E-08	4.35E-07	Dhx32	7	133720942	133782726	-	4172	protein_coding	DEAH (Asp-Glu-Ala-His) box polypeptide 32 [Source:MGI Symbol;Acc:MGI:2141813]	-
ENSMUSG0000002534	2641.541606	2137.078735	2600.745303	4595.201342	4326.887569	4561.543234	2459.788548	4494.544048	-0.869658604	1.24E-08	4.76E-07	Itga7	10	128933818	128958282	+	8092	protein_coding	integrin alpha 7 [Source:MGI Symbol;Acc:MGI:102700]	-
ENSMUSG0000007359	775.6271636	857.3593348	816.6429623	453.9214821	353.5781214	338.612851	816.5431536	382.0374848	1.094731667	1.25E-08	4.76E-07	Ecscr	18	35713086	35722356	-	1680	protein_coding	endothelial cell surface expressed chemotaxis and apoptosis regulator [Source:MGI Symbol;Acc:MGI:1915795]	-
ENSMUSG0000004159	328.6895474	180.1086563	453.5664059	87.85577073	49.38241919	72.0038821	320.7882032	69.74735734	2.199001844	1.25E-08	4.78E-07	Sdk2	11	113776374	114067046	-	15469	protein_coding	sidekick cell adhesion molecule 2 [Source:MGI Symbol;Acc:MGI:2443847]	-
ENSMUSG0000002755	234.4919332	262.263482	291.5784038	506.4626783	673.5761977	612.0329978	262.7779397	597.3572913	-1.184948057	1.29E-08	4.89E-07	Car13	3	14641727	14663002	+	2299	protein_coding	carbonic anhydrase 13 [Source:MGI Symbol;Acc:MGI:1931322]	-
ENSMUSG0000001731	379.7967636	379.1761186	461.3865163	177.4342036	191.6037864	153.7380185	406.7864662	174.2586695	1.222549167	1.29E-08	4.90E-07	Mpp2	11	102057015	102088515	-	4807	protein_coding	membrane protein, palmitoylated 2 (MAGUK p55 subfamily member 2) [Source:MGI Symbol;Acc:MGI:1858257]	-
ENSMUSG0000001218	253.5318765	113.7528356	215.6116166	510.7693338	752.5880684	851.3972546	194.2987762	704.9182189	-1.858620195	1.37E-08	5.19E-07	Mogat1	1	78510991	78538173	+	1558	protein_coding	monoacylglycerol O-acyltransferase 1 [Source:MGI Symbol;Acc:MGI:1915643]	-
ENSMUSG0000007835	356.7484112	253.8373461	230.1346789	1146.431675	627.1567237	804.6920337	280.2401454	859.4268108	-1.616387239	1.39E-08	5.27E-07	Smim1	4	154020470	154026230	-	5761	protein_coding	small integral membrane protein 1 [Source:MGI Symbol;Acc:MGI:1916109]	-
ENSMUSG0000005771	6.012613672	38.97087886	13.40590362	135.2289804	210.3691057	169.3064255	19.46313205	171.6348372	-3.14176882	1.41E-08	5.32E-07	A830018L16Ri	1	11414105	11975901	+	9183	protein_coding	RIKEN cDNA A830018L16 gene [Source:MGI Symbol;Acc:MGI:2444149]	-
ENSMUSG0000002746	15.03153418	14.74573795	10.05442772	101.6370681	64.19714494	78.81506014	13.27723328	81.54975773	-2.615988737	1.42E-08	5.34E-07	Tpx2	2	152847964	152895321	+	4677	protein_coding	TPX2, microtubule-associated [Source:MGI Symbol;Acc:MGI:1919369]	-
ENSMUSG0000003413	2113.433706	2346.678867	2375.079258	1273.047345	1298.757625	1363.208633	2278.397277	1311.671201	0.796597633	1.53E-08	5.74E-07	Sik3	9	46012820	46224194	+	9318	protein_coding	SIK family kinase 3 [Source:MGI Symbol;Acc:MGI:2446296]	-
ENSMUSG0000003484	4979.446222	5895.135377	3713.435303	2272.191404	1284.930547	1172.495648	4862.672301	1576.5392	1.624748921	1.53E-08	5.75E-07	Plvap	8	71497765	71511752	-	1987	protein_coding	plasmalemma vesicle associated protein [Source:MGI Symbol;Acc:MGI:1890497]	-
ENSMUSG0000007078	659.3832993	651.9722706	549.6420485	1096.474472	1385.670682	1532.515058	620.3325395	1338.220071	-1.108642576	1.70E-08	6.34E-07	Rbm47	5	66016549	66173118	-	7163	protein_coding	RNA binding motif protein 47 [Source:MGI Symbol;Acc:MGI:2384294]	-
ENSMUSG0000002461	6016.622081	3507.379097	2348.267451	16081.05137	11152.52555	13429.69704	3957.422876	13554.42465	-1.776053506	1.71E-08	6.38E-07	Cd74	18	60803848	60812646	+	1410	protein_coding	CD74 antigen (invariant polypeptide of major histocompatibility complex, class II antigen-associated) [Source:MGI Symbol;Acc:M	-
ENSMUSG0000002072	2.004204557	1.053266996	3.351475906	107.6663857	22.71591283	68.11178036	2.13631582	66.16469296	-4.965609694	1.71E-08	6.38E-07	Cacng4	11	107732357	107794557	-	3575	protein_coding	calcium channel, voltage-dependent, gamma subunit 4 [Source:MGI Symbol;Acc:MGI:1859167]	-
ENSMUSG0000011339	18.03784102	109.5397676	108.3643876	0	2.962945151	0	78.64733208	0.987648384	6.366650798	1.74E-08	6.48E-07	Gm48199	13	52180411	52181637	+	496	lincRNA	predicted gene, 48199 [Source:MGI Symbol;Acc:MGI:6097585]	-
ENSMUSG0000005227	24.05045469	75.83522372	108.3643876	0	3.950593535	2.919076301	69.41668867	2.289889945	4.956293146	1.84E-08	6.82E-07	Fpr2	17	17887824	17893952	+	1296	protein_coding	formyl peptide receptor 2 [Source:MGI Symbol;Acc:MGI:1278319]	-
ENSMUSG0000001419	2348.927741	2502.562383	3154.855986	1366.071102	1253.325799	1410.886879	2668.782037	1343.427927	0.990072921	1.94E-08	7.20E-07	Dnajc7	11	100582818	100620168	-	4863	protein_coding	DnaJ heat shock protein family (Hsp40) member C7 [Source:MGI Symbol;Acc:MGI:1928373]	-
ENSMUSG0000006811	213.4477853	203.2805302	224.5488857	73.21314228	88.88835454	56.43547516	213.7590671	72.84565732	1.553215418	1.95E-08	7.21E-07	Ninl	2	150934519	151039382	-	5952	protein_coding	ninein-like [Source:MGI Symbol;Acc:MGI:1925427]	-
ENSMUSG0000008555	265.5571038	349.6846427	325.0931628	125.7543385	138.2707737	116.7630521	313.4449698	126.9293881	1.303976951	2.09E-08	7.71E-07	4930412C18Ri	4	9770585	9823411	+	6148	processed_transcrip	RIKEN cDNA 4930412C18 gene [Source:MGI Symbol;Acc:MGI:2443490]	-
ENSMUSG0000002484	108.2270461	99.00709763	65.91235948	6.890648685	9.876483837	20.43353411	91.0488344	12.40022221	2.885504024	2.12E-08	7.81E-07	Cabp4	19	4135425	4139609	-	1431	protein_coding	calcium binding protein 4 [Source:MGI Symbol;Acc:MGI:1920910]	-
ENSMUSG0000002222	62.13034127	48.45028182	31.28044179	236.0047175	148.1472576	186.8208833	47.28702163	190.3242861	-2.006081765	2.14E-08	7.87E-07	Cma1	14	55941451	55944675	-	1090	protein_coding	chymase 1, mast cell [Source:MGI Symbol;Acc:MGI:96941]	-
ENSMUSG0000010908	48.10090937	48.45028182	150.8164158	1.722662171	5.925890302	7.78420347	82.45586898	5.144251981	4.017240307	2.27E-08	8.31E-07	4833411C07Ri	8	10899922	10903893	+	2257	processed_transcrip	RIKEN cDNA 4833411C07 gene [Source:MGI Symbol;Acc:MGI:1918874]	-
ENSMUSG0000002624	717.5052315	734.1270963	539.5876208	1544.366636	1233.572831	1557.813719	663.7399829	1445.251062	-1.122350035	2.27E-08	8.31E-07	Serpine2	1	79794197	79861180	-	8287	protein_coding	serine (or cysteine) peptidase inhibitor, clade E, member 2 [Source:MGI Symbol;Acc:MGI:101780]	-
ENSMUSG0000008160	193.4057398	183.2684573	220.0802511	68.04515576	30.6170999	57.40850059	198.9181494	52.02358542	1.930647445	2.39E-08	8.74E-07	Gm14681	X	66778442	66778852	-	411	processed_pseudog	predicted gene 14681 [Source:MGI Symbol;Acc:MGI:3705734]	-
ENSMUSG0000004283	448.9418208	491.8756872	170.9252712	1743.334117	1173.32628	1458.565125	370.5809264	1458.408507	-1.975985142	2.42E-08	8.81E-07	Nrep	18	33437019	33464029	-	2513	protein_coding	neuronal regeneration related protein [Source:MGI Symbol;Acc:MGI:99444]	-
ENSMUSG0000008736	63.13244355	68.46235475	101.6614358	12.91996628	14.81472576	7.78420347	77.75207803	11.83963184	2.712577889	2.45E-08	8.92E-07	0610043K17Ri	4	101353783	101399181	+	770	lincRNA	RIKEN cDNA 0610043K17 gene [Source:MGI Symbol;Acc:MGI:1915650]	-
ENSMUSG0000003465	117.2459666	122.1789715	110.5987049	293.7139002	251.8503379	282.1773758	116.6745477	275.9138713	-1.241800268	2.47E-08	8.97E-07	Cacna1a	8	84388440	84640246	+	11418	protein_coding	calcium channel, voltage-dependent, P/Q type, alpha 1A subunit [Source:MGI Symbol;Acc:MGI:109482]	-
ENSMUSG0000002660	3.006306836	8.426135969	6.702951811	69.76781793	47.40712242	60.32757689	6.045131539	59.16750575	-3.300050935	2.52E-08	9.15E-07	Cenpf	1	189640606	189688086	-	15433	protein_coding	centromere protein F [Source:MGI Symbol;Acc:MGI:1313302]	-
ENSMUSG0000000147	374.7862522	328.6193028	367.545191	673.5609089	826.6616972	685.0099054	356.983582	728.4108372	-1.028568987	2.55E-08	9.21E-07	Tubb6	18	67390717	67402748	+	1757	protein_coding	tubulin, beta 6 class V [Source:MGI Symbol;Acc:MGI:1915201]	-
ENSMUSG0000001989	234.4919332	369.6967156	278.1725002	114.5570344	101.7277835	87.57228904	294.120383	101.2857023	1.53655284	2.65E-08	9.58E-07	Dcbld1	10	52233619	52321378	+	7282	protein_coding	discoidin, CUB and LCCL domain containing 1 [Source:MGI Symbol;Acc:MGI:1913936]	-
ENSMUSG0000008594	113.2375575	148.5106465	275.9381829	41.34389211	31.60474828	36.97496648	179.2287956	36.64120229	2.287350968	2.70E-08	9.72E-07	2310014F06Ri	7	112612560	112680081	-	2810	bidirectional_prom	RIKEN cDNA 2310014F06 gene [Source:MGI Symbol;Acc:MGI:1916875]	-
ENSMUSG0000004807	2016.229785	1161.753497	1227.75734	2890.627123	6450.331594	6228.335802	1468.580207	5189.76484	-1.82105918	2.76E-08	9.91E-07	Tenm4	7	96171246	96911093	+	21021	protein_coding	teneurin transmembrane protein 4 [Source:MGI Symbol;Acc:MGI:2447063]	-
ENSMUSG0000011454	2.004204557	2.106533992	4.468634541	59.43184491	38.51828697	44.75916995	2.85979103	47.56976728	-4.072850519	2.76E-08	9.91E-07	Gm3226	13	112593743	112595084	-	1342	processed_pseudog	predicted gene 3226 [Source:MGI Symbol;Acc:MGI:3804968]	-
ENSMUSG0000002638	587.2319353	626.6938627	307.2186247	1366.071102	1665.175175	1378.77704	507.0481409	1470.077772	-1.535005282	2.80E-08	1.00E-06	Steap3	1	120190757	120272705	-	5715	protein_coding	STEAP family member 3 [Source:MGI Symbol;Acc:MGI:1915678]	-
ENSMUSG0000003527	175.3678988	168.5227194	137.4105121	608.0997464	350.6151762	422.2930383	160.4337101	460.335987	-1.520764779	2.80E-08	1.00E-06	Arx	X	93286445	93298357	+	4391	protein_coding	aristaless related homeobox [Source:MGI Symbol;Acc:MGI:1097716]	Hom
ENSMUSG0000002395	615.2907991	595.0958528	566.3994281	1078.386519	1007.401351	1175.414724	592.2620266	1087.067532	-0.875928451	2.97E-08	1.06E-06	Clic5	17	44188584	44280172	+	8782	protein_coding	chloride intracellular channel 5 [Source:MGI Symbol;Acc:MGI:1917912]	-
ENSMUSG0000002948	2212.641831	4005.574386	2599.628144	6466.873791	8125.383253	7836.746844	2939.281454	7476.334629	-1.346863342	3.07E-08	1.10E-06	Aacs	5	125475814	125517410	+	3854	protein_coding	acetoacetyl-CoA synthetase [Source:MGI Symbol;Acc:MGI:1926144]	-
ENSMUSG0000005295	1671.506601	1224.949516	1396.448294	3244.634199	2713.07011	2715.713986	1430.968137	2891.139432	-1.014608074	3.24E-08	1.15E-06	Gas1	13	60174405	60177365	-	2961	protein_coding	growth arrest specific 1 [Source:MGI Symbol;Acc:MGI:95655]	-
ENSMUSG0000000177	156.3279555	174.8423214	135.1761949	377.2630155	355.5534181	332.7746984	155.4488239	355.197044	-1.19173727	3.24E-08	1.15E-06	Folh1	7	86718977	86775943	-	3210	protein_coding	folate hydrolase 1 [Source:MGI Symbol;Acc:MGI:1858193]	-
ENSMUSG0000002246	958.0097784	925.8216896	892.6097495	1856.168489	1590.113898	1570.46305	925.4804058	1672.248479	-0.853615666	3.24E-08	1.15E-06	Srebf2	15	82147181	82205379	+	7184	protein_coding	sterol regulatory element binding factor 2 [Source:MGI Symbol;Acc:MGI:107585]	bHLI
ENSMUSG0000003988	134.2817053	76.88849072	21.22601407	594.3184491	441.4788275	455.375903	77.46540337	497.0577265	-2.678964517	3.25E-08	1.15E-06	Lrrc17	5	21543559	21575904	+	3608	protein_coding	leucine rich repeat containing 17 [Source:MGI Symbol;Acc:MGI:1921761]	-
ENSMUSG0000005742	1350.833872	1254.440992	1730.478726	742.4673958	684.4403299	700.5783123	1445.251197	709.1620127	1.026762732	3.40E-08	1.20E-06	Las1l	X	95935335	95956962	-	8143	protein_coding	LAS1-like (S. cerevisiae) [Source:MGI Symbol;Acc:MGI:1923380]	-
ENSMUSG0000002026	1600.357339	1814.779034	1561.787772	969.8588024	876.0441164	932.1583656	1658.974715	926.0204281	0.84102291	3.47E-08	1.22E-06	Adarb1	10	77290726	77418270	-	11272	protein_coding	adenosine deaminase, RNA-specific, B1 [Source:MGI Symbol;Acc:MGI:891999]	-
ENSMUSG0000002782	591.2403444	572.9772459	369.7795083	1008.618701	1926.901997	1707.659636	511.3323662	1547.726778	-1.597202117	3.48E-08	1.22E-06	Mme	3	63241537	63386030	+	11542	protein_coding	membrane metallo endopeptidase [Source:MGI Symbol;Acc:MGI:97004]	-
ENSMUSG0000004989	1314.75819	584.5631828	268.1180725	3491.836221	4046.395428	3577.81452	722.4798149	3705.348723	-2.358160887	3.60E-08	1.26E-06	Rasd1	11	59963181	59964944	-	1611	protein_coding	RAS, dexamethasone-induced 1 [Source:MGI Symbol;Acc:MGI:1270848]	-
ENSMUSG0000003468	1846.874499	1683.12066	2065.626317	990.5307484	746.6621781	573.1119805	1865.207159	770.1016357	1.275645346	3.62E-08	1.27E-06	Sema3f	9	107681500	107710475	-	5197	protein_coding	sema domain, immunoglobulin domain (Ig), short basic domain, secreted, (semaphorin) 3F [Source:MGI Symbol;Acc:MGI:109634	-
ENSMUSG0000007406	160.3363646	127.4453065	62.56088357	459.0894686	364.4422536	445.6456487	116.7808516	423.0591236	-1.854874737	3.66E-08	1.28E-06	Osgin1	8	119434124	119446256	+	2674	protein_coding	oxidative stress induced growth inhibitor 1 [Source:MGI Symbol;Acc:MGI:1919089]	-
ENSMUSG0000002132	1729.628533	2182.369216	2520.309881	940.5735455	1084.437925	1055.732596	2144.102543	1026.914689	1.062056597	3.67E-08	1.28E-06	Zkscan3	13	21387003	21402755	-	7974	protein_coding	zinc finger with KRAB and SCAN domains 3 [Source:MGI Symbol;Acc:MGI:1919989]	zf-C2
ENSMUSG0000003982	401.8430137	278.062487	392.122681	710.5981456	870.1182261	946.7537471	357.3427272	842.4900396	-1.237054679	3.77E-08	1.31E-06	Trub2	2	29761224	29788502	-	6316	protein_coding	TruB pseudouridine (psi) synthase family member 2 [Source:MGI Symbol;Acc:MGI:2442186]	-
ENSMUSG0000002173	771.6187545	457.1178763	479.2610545	1286.828642	1447.892531	1610.357093	569.3325618	1448.359422	-1.346433675	3.81E-08	1.33E-06	Fgf10	13	118669791	118792115	+	4601	protein_coding	fibroblast growth factor 10 [Source:MGI Symbol;Acc:MGI:1099809]	-
ENSMUSG0000002550	57614.86841	41929.50585	62077.15388	22771.87124	26030.4608	27961.83189	53873.84271	25588.05464	1.074120737	3.83E-08	1.33E-06	Pnpla2	7	141455198	141460743	+	3020	protein_coding	patatin-like phospholipase domain containing 2 [Source:MGI Symbol;Acc:MGI:1914103]	-
ENSMUSG0000001083	56.1177276	50.55681581	78.20110447	323.8604882	171.8508188	204.3353411	61.62521596	233.3488827	-1.923868942	3.90E-08	1.35E-06	Kdelr3	15	79516411	79527739	+	1411	protein_coding	KDEL (Lys-Asp-Glu-Leu) endoplasmic reticulum protein retention receptor 3 [Source:MGI Symbol;Acc:MGI:2145953]	-
ENSMUSG0000004531	1069.243131	761.5120382	539.5876208	1730.414151	2531.342808	2606.735137	790.1142634	2289.497365	-1.53437851	4.01E-08	1.39E-06	Lhfpl2	13	94057796	94195409	+	8415	protein_coding	lipoma HMGIC fusion partner-like 2 [Source:MGI Symbol;Acc:MGI:2145236]	-
ENSMUSG0000003881	325.6832406	324.4062348	590.976918	153.3169332	97.77718999	90.49136534	413.6887978	113.8618295	1.858684298	4.02E-08	1.39E-06	Gngt2	11	95837216	95845734	+	1877	protein_coding	guanine nucleotide binding protein (G protein), gamma transducing activity polypeptide 2 [Source:MGI Symbol;Acc:MGI:893584	-
ENSMUSG0000002853	1348.829667	597.2023868	593.2112353	2903.54709	2428.627376	3293.691093	846.4144297	2875.288519	-1.763903454	4.09E-08	1.41E-06	Cachd1	4	100776675	101029220	+	6020	protein_coding	cache domain containing 1 [Source:MGI Symbol;Acc:MGI:2444177]	-
ENSMUSG0000009788	304.6390927	225.3991372	401.05995	95.6077505	99.75248676	115.7900266	310.36606	103.7167546	1.581399644	4.32E-08	1.49E-06	5031434O11Ri	3	51560035	51567117	+	3731	processed_transcrip	RIKEN cDNA 5031434O11 gene [Source:MGI Symbol;Acc:MGI:1923230]	-
ENSMUSG0000000571	231.4856264	269.636351	233.4861548	98.19174376	93.82659646	108.9788486	244.8693774	100.3323963	1.287399215	4.33E-08	1.49E-06	Tfap4	16	4544661	4559854	-	2541	protein_coding	transcription factor AP4 [Source:MGI Symbol;Acc:MGI:103239]	bHLI
ENSMUSG0000007047	204.4288648	269.636351	198.8542371	453.060151	651.8479333	552.6784464	224.3064843	552.528436	-1.299939641	4.63E-08	1.59E-06	Fam217b	2	178414524	178424428	+	4559	protein_coding	family with sequence similarity 217, member B [Source:MGI Symbol;Acc:MGI:1918782]	-
ENSMUSG0000002306	2394.022344	2546.799597	2866.629058	1469.430832	1520.978511	1498.459168	2602.483666	1496.289504	0.798418538	4.88E-08	1.67E-06	Nus1	10	52417547	52440183	+	4867	protein_coding	NUS1 dehydrodolichyl diphosphate synthase subunit [Source:MGI Symbol;Acc:MGI:1196365]	-
ENSMUSG0000000703	1207.533246	1453.508455	1278.029479	755.3873621	568.885469	628.5744302	1313.023726	650.9490871	1.011657739	4.92E-08	1.68E-06	Neu1	17	34931253	34935953	+	3733	protein_coding	neuraminidase 1 [Source:MGI Symbol;Acc:MGI:97305]	-
ENSMUSG0000003063	3076.453995	3392.572994	3416.271107	1741.611455	1988.136196	1990.810038	3295.099365	1906.852563	0.789243643	5.23E-08	1.78E-06	Fah	7	84585159	84606722	-	2218	protein_coding	fumarylacetoacetate hydrolase [Source:MGI Symbol;Acc:MGI:95482]	-
ENSMUSG0000003299	612.2844922	501.3550901	516.1272895	1105.949114	1048.882584	965.2412303	543.255624	1040.024309	-0.936676163	5.27E-08	1.79E-06	Chpf	1	75474569	75479307	-	3856	protein_coding	chondroitin polymerizing factor [Source:MGI Symbol;Acc:MGI:106576]	-
ENSMUSG0000002804	1188.493302	1184.925371	821.1115969	2207.591572	2201.468247	2212.659836	1064.843423	2207.239885	-1.051261377	5.27E-08	1.79E-06	Zbtb7b	3	89377644	89394776	-	5591	protein_coding	zinc finger and BTB domain containing 7B [Source:MGI Symbol;Acc:MGI:102755]	ZBTI
ENSMUSG0000002585	328.6895474	238.0383411	210.0258234	869.0830654	613.3296463	591.5994637	258.917904	691.3373918	-1.41638473	5.31E-08	1.80E-06	Fam20c	5	138754514	138810077	+	6590	protein_coding	family with sequence similarity 20, member C [Source:MGI Symbol;Acc:MGI:2136853]	-
ENSMUSG0000002268	757.5893226	653.0255376	692.6383538	1668.398313	1215.79516	1373.911912	701.0844047	1419.368462	-1.017754608	5.34E-08	1.81E-06	Boc	16	44485049	44558897	-	4451	protein_coding	biregional cell adhesion molecule-related/down-regulated by oncogenes (Cdon) binding protein [Source:MGI Symbol;Acc:MGI:21	-
ENSMUSG0000005139	6559.761516	5794.021746	4614.982322	9980.243289	12530.29504	13769.28291	5656.255194	12093.27375	-1.096200915	5.40E-08	1.82E-06	Ywhag	5	135908409	135934616	-	5500	protein_coding	tyrosine 3-monooxygenase/tryptophan 5-monooxygenase activation protein, gamma polypeptide [Source:MGI Symbol;Acc:MGI:1	-
ENSMUSG0000004193	301.6327859	211.7066662	203.3228716	548.6679015	528.3918853	627.6014048	238.8874412	568.2203972	-1.248886003	5.70E-08	1.92E-06	Mvk	5	114444269	114460591	+	2675	protein_coding	mevalonate kinase [Source:MGI Symbol;Acc:MGI:107624]	-
ENSMUSG0000006175	214.4498876	194.8543943	157.5193676	410.8549278	453.3306081	403.805555	188.9412165	422.663697	-1.160136561	5.83E-08	1.96E-06	Akr1b10	6	34384218	34396950	+	3535	protein_coding	aldo-keto reductase family 1, member B10 (aldose reductase) [Source:MGI Symbol;Acc:MGI:1915111]	-
ENSMUSG0000003863	1848.878704	1669.428189	1586.365262	2902.685758	2823.686729	2933.671683	1701.557385	2886.68139	-0.762395348	5.87E-08	1.97E-06	Degs1	1	182275772	182282804	-	2591	protein_coding	delta(4)-desaturase, sphingolipid 1 [Source:MGI Symbol;Acc:MGI:1097711]	-
ENSMUSG0000006299	4483.405595	4120.380489	4960.18434	2660.651723	1910.111974	2278.825566	4521.323475	2283.196421	0.985476338	5.90E-08	1.98E-06	Rpl35	2	39001580	39005624	-	1241	protein_coding	ribosomal protein L35 [Source:MGI Symbol;Acc:MGI:1913739]	-
ENSMUSG0000000461	449.9439231	354.9509777	289.3440865	158.4849197	123.456048	131.3584336	364.7463291	137.7664671	1.404106973	5.92E-08	1.98E-06	Etfb	7	43444083	43457800	+	1164	protein_coding	electron transferring flavoprotein, beta polypeptide [Source:MGI Symbol;Acc:MGI:106098]	-
ENSMUSG0000003007	4.008409114	2.106533992	13.40590362	64.59983142	67.16009009	75.89598383	6.50694891	69.21863512	-3.428725727	5.98E-08	2.00E-06	Chl1	6	103510586	103750211	+	12293	protein_coding	cell adhesion molecule L1-like [Source:MGI Symbol;Acc:MGI:1098266]	-
ENSMUSG0000003145	4937.357927	4756.553754	4606.045053	1912.15501	2825.662026	2401.426771	4766.652245	2379.747935	1.002385384	6.02E-08	2.01E-06	Rasa3	8	13566948	13677603	-	5095	protein_coding	RAS p21 protein activator 3 [Source:MGI Symbol;Acc:MGI:1197013]	-
ENSMUSG0000003439	24.05045469	11.58593696	6.702951811	84.41044639	92.83894807	102.1676705	14.11311449	93.13902167	-2.710958411	6.16E-08	2.05E-06	Lif	11	4257557	4272514	+	4350	protein_coding	leukemia inhibitory factor [Source:MGI Symbol;Acc:MGI:96787]	-
ENSMUSG0000001648	4967.420995	6167.931529	5518.763658	2275.636728	3196.03017	2812.043504	5551.372061	2761.236801	1.007664621	6.30E-08	2.09E-06	Ppfibp1	6	146888487	147032025	+	11135	protein_coding	PTPRF interacting protein, binding protein 1 (liprin beta 1) [Source:MGI Symbol;Acc:MGI:1914783]	-
ENSMUSG0000011502	62.13034127	57.92968479	87.13837355	11.19730411	11.8517806	12.64933064	69.0661332	11.89947179	2.537298823	6.30E-08	2.09E-06	Gm49041	6	39207068	39207611	+	544	bidirectional_prom	predicted gene, 49041 [Source:MGI Symbol;Acc:MGI:6118414]	-
ENSMUSG0000002942	674.4148335	678.3039455	535.1189863	1155.044986	1190.116302	1315.530386	629.2792551	1220.230558	-0.954841415	6.48E-08	2.15E-06	Stx2	5	128984557	129008574	-	4586	protein_coding	syntaxin 2 [Source:MGI Symbol;Acc:MGI:108059]	-
ENSMUSG0000008741	42.0882957	31.59800988	25.69464861	146.4262846	133.3325318	104.1137214	33.12698473	127.9575126	-1.946453656	6.56E-08	2.17E-06	Gm11266	4	82508016	82547292	+	1271	antisense	predicted gene 11266 [Source:MGI Symbol;Acc:MGI:3649705]	-
ENSMUSG0000002691	740.5535839	786.7904461	872.5008941	1352.289804	1443.941937	1561.705821	799.948308	1452.645854	-0.860872935	6.56E-08	2.17E-06	Psmd14	2	61711694	61800376	+	2820	protein_coding	proteasome (prosome, macropain) 26S subunit, non-ATPase, 14 [Source:MGI Symbol;Acc:MGI:1913284]	-
ENSMUSG0000002967	188.3952284	205.3870642	193.2684439	387.5989885	387.1581664	428.1311909	195.6835788	400.9627819	-1.034890486	6.70E-08	2.21E-06	Limk1	5	134656039	134688598	-	7162	protein_coding	LIM-domain containing, protein kinase [Source:MGI Symbol;Acc:MGI:104572]	-
ENSMUSG0000007954	189.3973307	112.6995686	113.9501808	493.542712	341.7263408	399.9134533	138.68236	411.727502	-1.568758425	6.76E-08	2.22E-06	H2-DMb1	17	34153072	34160230	+	1485	protein_coding	histocompatibility 2, class II, locus Mb1 [Source:MGI Symbol;Acc:MGI:95922]	-
ENSMUSG0000002503	840.7638118	741.4999653	850.1577214	1399.663014	1623.693943	1406.994777	810.8071661	1476.783911	-0.864935199	7.06E-08	2.32E-06	Trim8	19	46501648	46516444	+	3230	protein_coding	tripartite motif-containing 8 [Source:MGI Symbol;Acc:MGI:1933302]	-
ENSMUSG0000002149	2118.444217	1791.60716	2440.991618	1034.458634	1146.659774	848.4781783	2117.014332	1009.865529	1.067776434	7.23E-08	2.37E-06	Fam193b	13	55539316	55571120	-	11540	protein_coding	family with sequence similarity 193, member B [Source:MGI Symbol;Acc:MGI:2385851]	-
ENSMUSG0000002012	1063.230518	832.0809269	1361.816376	2283.388708	2325.911944	2836.369139	1085.709274	2481.88993	-1.19297189	7.25E-08	2.37E-06	Egfr	11	16752203	16918158	+	11326	protein_coding	epidermal growth factor receptor [Source:MGI Symbol;Acc:MGI:95294]	-
ENSMUSG0000003538	111.2333529	113.7528356	80.43542174	292.8525691	513.5771595	254.9326637	101.8072034	353.7874641	-1.795568636	7.46E-08	2.44E-06	Ccl2	11	82035571	82037453	+	1138	protein_coding	chemokine (C-C motif) ligand 2 [Source:MGI Symbol;Acc:MGI:98259]	-
ENSMUSG0000008031	293.6159676	271.742885	291.5784038	590.8731247	534.3177756	527.3797851	285.6457521	550.8568951	-0.947791823	7.54E-08	2.46E-06	Spaca6	17	17827158	17843009	+	7067	protein_coding	sperm acrosome associated 6 [Source:MGI Symbol;Acc:MGI:1922452]	-
ENSMUSG0000004011	264.5550016	239.0916081	263.6494379	480.6227458	495.7994886	573.1119805	255.7653492	516.511405	-1.013718252	7.80E-08	2.54E-06	Cacna2d1	5	15934691	16374511	+	12416	protein_coding	calcium channel, voltage-dependent, alpha2/delta subunit 1 [Source:MGI Symbol;Acc:MGI:88295]	-
ENSMUSG0000003777	484.0154006	427.6264004	474.79242	233.4207242	238.0232605	232.5530787	462.1447403	234.6656878	0.977835562	7.95E-08	2.58E-06	Pced1a	2	130417247	130424701	-	5072	protein_coding	PC-esterase domain containing 1A [Source:MGI Symbol;Acc:MGI:2442177]	-
ENSMUSG0000001874	298.626479	329.6725698	141.8791467	666.6702602	988.6360321	887.3991956	256.7260652	847.568496	-1.721985228	8.20E-08	2.66E-06	Slc25a35	11	68968131	68974365	+	3677	protein_coding	solute carrier family 25, member 35 [Source:MGI Symbol;Acc:MGI:1919248]	-
ENSMUSG0000002441	2592.438595	4016.107056	1501.461206	815.6805381	837.5258294	885.4531447	2703.335619	846.2198374	1.675733227	8.58E-08	2.78E-06	Npc1	18	12189693	12236400	-	10725	protein_coding	NPC intracellular cholesterol transporter 1 [Source:MGI Symbol;Acc:MGI:1097712]	-
ENSMUSG0000003071	5111.723723	5406.419491	10037.67034	2113.706484	2843.439697	2388.77744	6851.937851	2448.641207	1.484538256	8.76E-08	2.83E-06	Sult1a1	7	126672865	126676432	-	1507	protein_coding	sulfotransferase family 1A, phenol-preferring, member 1 [Source:MGI Symbol;Acc:MGI:102896]	-
ENSMUSG0000002593	2212.641831	2317.187391	1876.826507	3516.814822	5506.139739	4970.213916	2135.55191	4664.389493	-1.126914749	8.82E-08	2.84E-06	Gsta3	1	21240589	21265661	+	4344	protein_coding	glutathione S-transferase, alpha 3 [Source:MGI Symbol;Acc:MGI:95856]	-
ENSMUSG0000003390	1774.723135	1459.828057	1369.636487	841.5204706	523.4536434	571.1659296	1534.729226	645.3800145	1.249193208	8.88E-08	2.86E-06	Mapkbp1	2	119972699	120027408	+	7602	protein_coding	mitogen-activated protein kinase binding protein 1 [Source:MGI Symbol;Acc:MGI:1347004]	-
ENSMUSG0000002535	566.1877874	493.9822212	503.8385445	873.3897208	1178.264522	1226.985072	521.3361844	1092.879772	-1.067274294	9.20E-08	2.96E-06	Mmp19	10	128790910	128800824	+	3533	protein_coding	matrix metallopeptidase 19 [Source:MGI Symbol;Acc:MGI:1927899]	-
ENSMUSG0000007661	2.004204557	2.106533992	1.117158635	189.4928388	87.90070615	6.811178036	1.742632395	94.73490767	-5.758316492	9.22E-08	2.96E-06	Ighg2b	12	113302965	113307933	-	2678	IG_C_gene	immunoglobulin heavy constant gamma 2B [Source:MGI Symbol;Acc:MGI:96445]	-
ENSMUSG0000002534	21.04414785	17.90553893	29.04612452	106.8050546	87.90070615	92.43741621	22.66527043	95.71439233	-2.082590131	9.23E-08	2.96E-06	Mettl7b	10	128958274	128960988	-	1230	protein_coding	methyltransferase like 7B [Source:MGI Symbol;Acc:MGI:1918914]	-
ENSMUSG0000002878	1044.190574	694.1029504	1102.635573	1905.264361	2136.283454	2408.237949	946.9763659	2149.928588	-1.182882285	9.77E-08	3.13E-06	Sema3c	5	17574281	17730268	+	7140	protein_coding	sema domain, immunoglobulin domain (Ig), short basic domain, secreted, (semaphorin) 3C [Source:MGI Symbol;Acc:MGI:107557]	-
ENSMUSG0000003310	1606.369953	3019.716478	1488.055302	269.5966298	676.5391429	612.0329978	2038.047244	519.3895902	1.972855207	9.83E-08	3.14E-06	Rnf125	18	20944625	20983848	+	1334	protein_coding	ring finger protein 125 [Source:MGI Symbol;Acc:MGI:1914914]	-
ENSMUSG0000004403	1309.747678	1151.220827	1910.341266	627.9103614	668.6379558	524.4607088	1457.103257	607.0030087	1.263075129	9.97E-08	3.18E-06	Als2cl	9	110879870	110900530	+	5883	protein_coding	ALS2 C-terminal like [Source:MGI Symbol;Acc:MGI:2447532]	-
ENSMUSG0000011586	283.5949448	181.1619233	227.9003616	80.96512205	69.13538686	89.51833991	230.8857433	79.87294961	1.531535518	1.00E-07	3.18E-06	Gm2999	15	71794570	71795107	+	428	processed_pseudog	predicted gene 2999 [Source:MGI Symbol;Acc:MGI:3781177]	-
ENSMUSG0000005358	485.0175029	499.2485562	342.967701	819.9871935	1238.511073	1312.61131	442.4112533	1123.703192	-1.344051852	1.00E-07	3.19E-06	Tanc2	11	105589986	105929304	+	16605	protein_coding	tetratricopeptide repeat, ankyrin repeat and coiled-coil containing 2 [Source:MGI Symbol;Acc:MGI:2444121]	-
ENSMUSG0000003201	205.4309671	128.4985735	140.761988	451.3374888	375.3063858	390.1831989	158.2305096	405.6090245	-1.357040691	1.03E-07	3.26E-06	Thy1	9	44043384	44048579	+	2995	protein_coding	thymus cell antigen 1, theta [Source:MGI Symbol;Acc:MGI:98747]	-
ENSMUSG0000000937	290.6096608	135.8714425	83.78689764	732.1314228	619.2555366	808.5841355	170.0893336	719.9903649	-2.079987456	1.03E-07	3.27E-06	Slc16a12	19	34668403	34747289	-	4051	protein_coding	solute carrier family 16 (monocarboxylic acid transporters), member 12 [Source:MGI Symbol;Acc:MGI:2147716]	-
ENSMUSG0000003021	870.8268801	609.8415907	585.3911249	1385.881717	1471.596092	1789.393773	688.6865319	1548.957194	-1.168821882	1.08E-07	3.42E-06	Plbd1	6	136612070	136661928	-	2633	protein_coding	phospholipase B domain containing 1 [Source:MGI Symbol;Acc:MGI:1914107]	-
ENSMUSG0000002345	3165.641098	2994.43807	1863.420604	6226.562418	5840.952541	6072.651732	2674.499924	6046.72223	-1.176747463	1.11E-07	3.50E-06	Tpi1	6	124810586	124814296	-	2753	protein_coding	triosephosphate isomerase 1 [Source:MGI Symbol;Acc:MGI:98797]	-
ENSMUSG0000006286	1016.131711	995.3373113	983.099599	583.1211449	492.8365435	410.6167331	998.1895403	495.5248072	1.009664257	1.12E-07	3.54E-06	Impdh2	9	108560286	108565584	+	3077	protein_coding	inosine monophosphate dehydrogenase 2 [Source:MGI Symbol;Acc:MGI:109367]	-
ENSMUSG0000002092	252.5297742	248.5710111	180.9796989	70.62914902	91.85129969	85.62623817	227.3601614	82.70222896	1.461556477	1.15E-07	3.63E-06	Higd1b	11	102835849	102838040	+	847	protein_coding	HIG1 domain family, member 1B [Source:MGI Symbol;Acc:MGI:1922939]	-
ENSMUSG0000003027	75062.47118	73472.74585	77712.90614	34876.15699	41979.0069	47400.93401	75416.04106	41418.6993	0.864599002	1.16E-07	3.64E-06	Cidec	6	113424634	113435760	-	1856	protein_coding	cell death-inducing DFFA-like effector c [Source:MGI Symbol;Acc:MGI:95585]	-
ENSMUSG0000001937	1940.070011	1539.876348	1443.368597	4051.701427	2993.562251	3180.820143	1641.105105	3408.694607	-1.054492051	1.18E-07	3.71E-06	Calm3	7	16915379	16924114	-	2423	protein_coding	calmodulin 3 [Source:MGI Symbol;Acc:MGI:103249]	-
ENSMUSG0000005732	2610.476436	1908.519797	2827.528506	1283.383318	810.8593231	982.7556881	2448.841579	1025.66611	1.255147857	1.19E-07	3.73E-06	Rpl38	11	114668524	114672331	+	542	protein_coding	ribosomal protein L38 [Source:MGI Symbol;Acc:MGI:1914921]	-
ENSMUSG0000002471	1122.354552	999.5503793	2068.977792	484.9294012	526.4165885	446.6186741	1396.960908	485.9882213	1.523084001	1.19E-07	3.73E-06	Pcsk5	19	17432832	17837632	-	9346	protein_coding	proprotein convertase subtilisin/kexin type 5 [Source:MGI Symbol;Acc:MGI:97515]	-
ENSMUSG0000000595	24.05045469	12.63920395	18.9916968	60.29317599	167.9002252	125.520281	18.56045181	117.9045607	-2.664007055	1.21E-07	3.78E-06	Trpv1	11	73234292	73261242	+	3681	protein_coding	transient receptor potential cation channel, subfamily V, member 1 [Source:MGI Symbol;Acc:MGI:1341787]	-
ENSMUSG0000009746	253.5318765	310.7137638	307.2186247	104.2210614	130.3695867	131.3584336	290.4880883	121.9830272	1.252974035	1.26E-07	3.91E-06	9530026P05Ri	6	92940329	93272979	+	5750	antisense	RIKEN cDNA 9530026P05 gene [Source:MGI Symbol;Acc:MGI:1924659]	-
ENSMUSG0000002796	210.4414785	164.3096514	89.37269082	379.8470087	634.0702624	637.3316591	154.7079402	550.4163101	-1.829006773	1.26E-07	3.91E-06	Lrrc39	3	116562973	116583134	+	5230	protein_coding	leucine rich repeat containing 39 [Source:MGI Symbol;Acc:MGI:1924557]	-
ENSMUSG0000001693	3515.374793	4373.164568	3395.045092	2055.997301	2046.407451	2132.871751	3761.194818	2078.425501	0.855731221	1.30E-07	4.02E-06	Plcg1	2	160731300	160775760	+	7857	protein_coding	phospholipase C, gamma 1 [Source:MGI Symbol;Acc:MGI:97615]	-
ENSMUSG0000003207	766.6082431	1030.095122	1011.028565	502.1560229	436.5405856	409.6437076	935.9106434	449.446772	1.057468162	1.33E-07	4.13E-06	Zpr1	9	46273064	46282643	+	3583	protein_coding	ZPR1 zinc finger [Source:MGI Symbol;Acc:MGI:1330262]	-
ENSMUSG0000003073	3966.320819	4833.442245	3789.402091	1895.789719	2403.936166	2275.90649	4196.388385	2191.877458	0.93715628	1.37E-07	4.24E-06	Slco2b1	7	99657804	99711340	-	5958	protein_coding	solute carrier organic anion transporter family, member 2b1 [Source:MGI Symbol;Acc:MGI:1351872]	-
ENSMUSG0000007341	29802.52177	38654.89876	29923.09404	14552.18869	18270.50745	17968.86069	32793.50486	16930.51894	0.953804459	1.37E-07	4.24E-06	C4b	17	34728380	34743882	-	5695	protein_coding	complement component 4B (Chido blood group) [Source:MGI Symbol;Acc:MGI:88228]	-
ENSMUSG0000002235	639.3412538	1103.823812	602.1485044	292.8525691	279.5044926	300.664859	781.77119	291.0073069	1.425714215	1.39E-07	4.27E-06	Fbxo32	15	58175879	58214932	-	6976	protein_coding	F-box protein 32 [Source:MGI Symbol;Acc:MGI:1914981]	-
ENSMUSG0000003177	838.7596072	765.7251062	690.4040366	1328.172534	1332.33767	1459.538151	764.9629166	1373.349451	-0.843802444	1.39E-07	4.27E-06	Arl2bp	8	94666600	94674425	+	2802	protein_coding	ADP-ribosylation factor-like 2 binding protein [Source:MGI Symbol;Acc:MGI:1349429]	-
ENSMUSG0000002005	4971.429404	3201.931668	5536.638196	10601.263	9377.721404	11342.55748	4569.999756	10440.51396	-1.191956551	1.46E-07	4.48E-06	Igf1	10	87858265	87937042	+	9904	protein_coding	insulin-like growth factor 1 [Source:MGI Symbol;Acc:MGI:96432]	-
ENSMUSG0000008544	71.14926178	53.7166168	164.2223194	9.474641942	13.82707737	14.59538151	96.36273265	12.63236694	2.934263671	1.46E-07	4.48E-06	Gm16348	8	41218139	41220894	+	2385	processed_transcrip	predicted gene 16348 [Source:MGI Symbol;Acc:MGI:3840113]	-
ENSMUSG0000002246	1825.830352	1414.537576	1913.692742	812.2352137	951.1053935	866.9656615	1718.020223	876.7687563	0.970641495	1.47E-07	4.49E-06	Rapgef3	15	97744770	97767972	-	6336	protein_coding	Rap guanine nucleotide exchange factor (GEF) 3 [Source:MGI Symbol;Acc:MGI:2441741]	-
ENSMUSG0000008678	234.4919332	199.0674623	242.4234238	57.70918273	83.95011262	95.35649251	225.3276064	79.00526262	1.514908118	1.48E-07	4.51E-06	E130102H24Ri	4	101346531	101356735	-	918	lincRNA	RIKEN cDNA E130102H24 gene [Source:MGI Symbol;Acc:MGI:1925116]	-
ENSMUSG0000001995	2194.60399	2366.69094	2277.886457	1273.047345	1410.361892	1422.563184	2279.727129	1368.657474	0.73627519	1.49E-07	4.56E-06	Uhrf1bp1l	10	89744991	89819871	+	9235	protein_coding	UHRF1 (ICBP90) binding protein 1-like [Source:MGI Symbol;Acc:MGI:2442888]	-
ENSMUSG0000002971	671.4085267	456.0646093	515.0101308	1953.498902	1232.585183	1132.601605	547.4944223	1439.561897	-1.394730809	1.51E-07	4.60E-06	Pcolce	5	137605103	137613784	-	3823	protein_coding	procollagen C-endopeptidase enhancer protein [Source:MGI Symbol;Acc:MGI:105099]	-
ENSMUSG0000005506	287.603354	286.4886229	313.9215765	148.1489467	131.357235	128.4393573	296.0045178	135.9818463	1.120845063	1.52E-07	4.61E-06	Smyd3	1	178951960	179518041	-	9184	protein_coding	SET and MYND domain containing 3 [Source:MGI Symbol;Acc:MGI:1916976]	-
ENSMUSG0000002867	1346.825462	1352.394823	1203.17985	746.7740512	784.1928167	685.9829308	1300.800045	738.9832662	0.815916602	1.53E-07	4.65E-06	Hmgcl	4	135946448	135962617	+	2434	protein_coding	3-hydroxy-3-methylglutaryl-Coenzyme A lyase [Source:MGI Symbol;Acc:MGI:96158]	-
ENSMUSG0000003368	2767.806494	3374.667456	3567.087522	1869.088456	1707.644055	1802.043103	3236.52049	1792.925205	0.851942399	1.58E-07	4.79E-06	Qsox1	1	155776029	155812889	-	8291	protein_coding	quiescin Q6 sulfhydryl oxidase 1 [Source:MGI Symbol;Acc:MGI:1330818]	-
ENSMUSG0000003300	3247.813485	3102.924571	3544.74435	5178.322487	6403.91212	7218.875693	3298.494135	6267.036767	-0.925944393	1.61E-07	4.88E-06	Mycbp2	14	103113411	103346814	-	19248	protein_coding	MYC binding protein 2, E3 ubiquitin protein ligase [Source:MGI Symbol;Acc:MGI:2179432]	-
ENSMUSG0000003756	5958.500149	5553.87687	5156.80426	3465.134957	2505.66395	2544.461509	5556.39376	2838.420139	0.968909216	1.67E-07	5.06E-06	Rps16	7	28350652	28353155	+	1266	protein_coding	ribosomal protein S16 [Source:MGI Symbol;Acc:MGI:98118]	-
ENSMUSG0000002113	11000.07671	10054.48674	14362.19141	6576.262838	5597.991039	5852.747984	11805.58496	6009.000621	0.974207071	1.68E-07	5.06E-06	Srsf5	12	80945504	80950507	+	3988	protein_coding	serine/arginine-rich splicing factor 5 [Source:MGI Symbol;Acc:MGI:98287]	-
ENSMUSG0000006316	240.5045469	225.3991372	207.7915062	559.8652056	411.849376	480.6745643	224.5650634	484.1297153	-1.108358651	1.72E-07	5.20E-06	Numbl	7	27258433	27282144	+	8673	protein_coding	numb-like [Source:MGI Symbol;Acc:MGI:894702]	-
ENSMUSG0000005879	5339.20094	7313.886021	5142.281198	2580.547932	3194.054873	2974.538751	5931.789386	2916.380519	1.024382677	1.76E-07	5.29E-06	Cds2	2	132263148	132312050	+	9125	protein_coding	CDP-diacylglycerol synthase (phosphatide cytidylyltransferase) 2 [Source:MGI Symbol;Acc:MGI:1332236]	-
ENSMUSG0000003358	332.6979565	159.0433164	345.2020183	1015.50935	717.0327266	824.1525424	278.9810971	852.2315396	-1.611406563	1.76E-07	5.29E-06	Ndn	7	62346569	62350262	+	3694	protein_coding	necdin [Source:MGI Symbol;Acc:MGI:97290]	-
ENSMUSG0000003018	5759.081795	6390.170865	6720.826349	3299.759389	3989.111822	3581.706622	6290.026337	3623.525944	0.795722507	1.76E-07	5.29E-06	Ybx3	6	131364855	131388476	-	3075	protein_coding	Y box protein 3 [Source:MGI Symbol;Acc:MGI:2137670]	CSD
ENSMUSG0000004085	202.4246603	166.4161854	135.1761949	396.2122994	357.5287149	439.8074961	168.0056802	397.8495035	-1.242209472	1.77E-07	5.30E-06	Reps2	X	162411954	162643649	-	8296	protein_coding	RALBP1 associated Eps domain containing protein 2 [Source:MGI Symbol;Acc:MGI:2663511]	-
ENSMUSG0000002422	1053.209495	1247.068123	1003.208454	441.0015158	578.7619529	592.5724892	1101.162024	537.4453193	1.035550674	1.81E-07	5.40E-06	Nudt12	17	58999618	59013372	-	4401	protein_coding	nudix (nucleoside diphosphate linked moiety X)-type motif 12 [Source:MGI Symbol;Acc:MGI:1915243]	-
ENSMUSG0000007043	3809.992863	2851.193758	5112.117915	8107.709509	10155.98833	9078.327297	3924.434846	9114.008379	-1.215647199	1.81E-07	5.41E-06	Serpinh1	7	99345376	99353239	-	2443	protein_coding	serine (or cysteine) peptidase inhibitor, clade H, member 1 [Source:MGI Symbol;Acc:MGI:88283]	-
ENSMUSG0000004643	4557.561163	4527.994816	4045.231418	2749.368825	2318.998405	2061.840894	4376.929133	2376.736041	0.880799242	1.86E-07	5.55E-06	Hnrnpa1	15	103240432	103246692	+	3153	protein_coding	heterogeneous nuclear ribonucleoprotein A1 [Source:MGI Symbol;Acc:MGI:104820]	-
ENSMUSG0000003527	161.3384669	60.03621878	93.84132536	402.241617	361.4793085	349.3161307	105.0720037	371.012352	-1.818527129	1.91E-07	5.69E-06	Ssc5d	7	4925785	4944826	+	4590	protein_coding	scavenger receptor cysteine rich family, 5 domains [Source:MGI Symbol;Acc:MGI:3606211]	-
ENSMUSG0000007560	3589.530362	3238.796013	4274.248938	2157.634369	1887.396061	1647.332059	3700.858438	1897.454163	0.963576964	1.95E-07	5.80E-06	Ly6a	15	74994877	74998031	-	1205	protein_coding	lymphocyte antigen 6 complex, locus A [Source:MGI Symbol;Acc:MGI:107527]	-
ENSMUSG0000005049	119.2501712	205.3870642	106.1300703	42.20522319	21.72826444	16.54143237	143.5891019	26.82497334	2.413376779	1.96E-07	5.82E-06	Fam167b	4	129576815	129578549	-	959	protein_coding	family with sequence similarity 167, member B [Source:MGI Symbol;Acc:MGI:2668032]	-
ENSMUSG0000001281	113.2375575	69.51562174	75.96678719	199.8288119	391.10876	293.853681	86.23998881	294.9304176	-1.771605363	1.97E-07	5.83E-06	Cdh23	10	60302748	60696490	-	16939	protein_coding	cadherin 23 (otocadherin) [Source:MGI Symbol;Acc:MGI:1890219]	-
ENSMUSG0000002053	2465.171605	4525.888282	1427.728756	8820.891648	9978.211621	10337.42221	2806.262874	9712.175159	-1.791088229	1.99E-07	5.88E-06	Srebf1	11	60199089	60222581	-	5634	protein_coding	sterol regulatory element binding transcription factor 1 [Source:MGI Symbol;Acc:MGI:107606]	bHLI
ENSMUSG0000003010	1778.731545	1304.997808	1600.888324	2604.665203	3931.828216	3768.527505	1561.539226	3435.006975	-1.137176652	2.03E-07	6.00E-06	Edem1	6	108828641	108859356	+	6453	protein_coding	ER degradation enhancer, mannosidase alpha-like 1 [Source:MGI Symbol;Acc:MGI:2180139]	-
ENSMUSG0000003055	26.05465924	21.06533992	29.04612452	93.02375724	87.90070615	108.9788486	25.38870789	96.63443733	-1.929437175	2.05E-07	6.05E-06	Rab38	7	88430273	88491572	+	1577	protein_coding	RAB38, member RAS oncogene family [Source:MGI Symbol;Acc:MGI:1919683]	-
ENSMUSG0000000276	2206.629218	2475.177441	1894.701045	1003.450715	1296.782328	992.4859425	2192.169235	1097.572995	0.99831681	2.06E-07	6.05E-06	Pex6	17	46711463	46725541	+	3796	protein_coding	peroxisomal biogenesis factor 6 [Source:MGI Symbol;Acc:MGI:2385054]	-
ENSMUSG0000003232	2436.110639	1900.093661	1566.256407	4358.335293	3989.111822	3800.637344	1967.486902	4049.361486	-1.041165409	2.13E-07	6.27E-06	Tspan3	9	56131725	56165477	-	4274	protein_coding	tetraspanin 3 [Source:MGI Symbol;Acc:MGI:1928098]	-
ENSMUSG0000003594	3269.859735	3017.609944	2825.294188	875.112383	1487.398466	1528.622956	3037.587956	1297.044602	1.228082489	2.15E-07	6.29E-06	Acss3	10	106933517	107123668	-	7196	protein_coding	acyl-CoA synthetase short-chain family member 3 [Source:MGI Symbol;Acc:MGI:2685720]	-
ENSMUSG0000000605	2607.470129	2490.976446	3559.267412	1572.790562	1365.917715	1335.963921	2885.904662	1424.890733	1.017904778	2.15E-07	6.30E-06	Sra1	18	36666681	36679366	-	4684	protein_coding	steroid receptor RNA activator 1 [Source:MGI Symbol;Acc:MGI:1344414]	-
ENSMUSG0000003289	5036.566052	2534.160393	2260.011919	1108.533107	1156.536257	1140.385808	3276.912788	1135.151724	1.529582057	2.17E-07	6.35E-06	Fbxo21	5	117976730	118010201	+	5545	protein_coding	F-box protein 21 [Source:MGI Symbol;Acc:MGI:1924223]	-
ENSMUSG0000004256	1693.552851	1453.508455	1998.596798	786.3952811	898.7600292	943.8346708	1715.219368	876.3299937	0.968976926	2.24E-07	6.54E-06	Dhrs7b	11	60830631	60860195	+	3325	protein_coding	dehydrogenase/reductase (SDR family) member 7B [Source:MGI Symbol;Acc:MGI:2384931]	-
ENSMUSG0000004500	180.3784102	150.6171804	141.8791467	308.3565286	381.2322761	373.6417666	157.6249124	354.4101904	-1.167223973	2.26E-07	6.59E-06	Fzd5	1	64730558	64737751	-	6915	protein_coding	frizzled class receptor 5 [Source:MGI Symbol;Acc:MGI:108571]	-
ENSMUSG0000002920	1573.300577	1689.440262	1508.164158	961.2454915	974.8089548	895.1833991	1590.301666	943.7459485	0.752832031	2.26E-07	6.59E-06	Apbb2	5	66298703	66618784	-	10966	protein_coding	amyloid beta (A4) precursor protein-binding, family B, member 2 [Source:MGI Symbol;Acc:MGI:108405]	-

ENSMUSG0000003310	359.754718	406.5610605	281.5239761	696.8168482	771.3533877	707.3894904	349.2799182	725.1865754	-1.053225811	2.28E-07	6.62E-06	Lss	10	76531588	76557138	+	10787	protein_coding	lanosterol synthase [Source:MGI Symbol;Acc:MGI:1336155]	-
ENSMUSG0000005316	0	0	0	42.20522319	13.82707737	40.86706822	0	32.2997896	-7.378204657	2.30E-07	6.67E-06	9030619P08Ri	15	75427606	75431829	-	935	transcribed_unproc	RIKEN cDNA 9030619P08 gene [Source:MGI Symbol;Acc:MGI:3612405]	-
ENSMUSG0000003977	3614.582919	3094.498435	4099.972191	1739.888793	1877.519578	2129.952675	3603.017848	1915.787015	0.911328026	2.33E-07	6.75E-06	Ypel5	17	72836453	72851195	+	4185	protein_coding	yippee like 5 [Source:MGI Symbol;Acc:MGI:1916937]	-
ENSMUSG0000000055	492.0322188	270.689618	330.678956	917.3176062	827.6493456	937.0234927	364.4669309	893.9968148	-1.293847149	2.34E-07	6.76E-06	Zfp385a	15	103313895	103340093	-	2515	protein_coding	zinc finger protein 385A [Source:MGI Symbol;Acc:MGI:1352495]	-
ENSMUSG0000004012	2576.404958	2558.385534	2974.993446	1472.876156	1683.940494	1348.613251	2703.261312	1501.809967	0.84798155	2.38E-07	6.87E-06	Pnrc1	4	33245423	33290163	-	2095	protein_coding	proline-rich nuclear receptor coactivator 1 [Source:MGI Symbol;Acc:MGI:1917838]	-
ENSMUSG0000004453	11708.56302	12817.20608	9635.493229	6418.63925	6371.319724	5116.167731	11387.08744	5968.708901	0.931902554	2.43E-07	7.01E-06	Rps2	17	24718116	24721929	+	1830	protein_coding	ribosomal protein S2 [Source:MGI Symbol;Acc:MGI:105110]	-
ENSMUSG0000003793	7726.208568	13973.69324	6821.370627	3584.859978	3746.15032	3588.5178	9507.090811	3639.842699	1.385140892	2.46E-07	7.08E-06	Scarb1	5	125277087	125341094	-	8500	protein_coding	scavenger receptor class B, member 1 [Source:MGI Symbol;Acc:MGI:893578]	-
ENSMUSG0000008750	5.010511393	4.213067984	1.117158635	36.17590559	67.16009009	39.89404278	3.446912671	47.74334616	-3.773433962	2.47E-07	7.10E-06	Gm12426	4	47197487	47208459	-	371	antisense	predicted gene 12426 [Source:MGI Symbol;Acc:MGI:3650989]	-
ENSMUSG0000004779	256.5381833	471.8636143	204.4400302	898.3683223	1043.944342	860.1544835	310.9472759	934.1557158	-1.586661597	2.57E-07	7.38E-06	Sned1	1	93235841	93301065	+	9491	protein_coding	sushi, nidogen and EGF-like domains 1 [Source:MGI Symbol;Acc:MGI:3045960]	-
ENSMUSG0000004435	216.4540922	186.4282583	198.8542371	360.8977249	489.8735983	468.0252336	200.5788625	439.5988523	-1.130995569	2.58E-07	7.39E-06	Lacc1	14	77024200	77036904	-	3644	protein_coding	laccase domain containing 1 [Source:MGI Symbol;Acc:MGI:2445077]	-
ENSMUSG0000003171	810.7007434	828.9211259	903.7813359	481.4840768	460.2441468	501.1080984	847.8010684	480.9454407	0.817606324	2.59E-07	7.41E-06	Zfp330	8	82763607	82774160	-	5686	protein_coding	zinc finger protein 330 [Source:MGI Symbol;Acc:MGI:1353574]	-
ENSMUSG0000002808	13441.19786	15270.26491	15642.45521	9458.276651	7461.683539	7175.089549	14784.63933	8031.683246	0.880252331	2.62E-07	7.48E-06	Rps3a1	3	86137940	86142702	-	977	protein_coding	ribosomal protein S3A1 [Source:MGI Symbol;Acc:MGI:1202063]	-
ENSMUSG0000003425	148.3111372	140.0845105	107.247229	38.75989885	43.45652888	22.37958498	131.8809589	34.86533757	1.918960954	2.62E-07	7.48E-06	Flvcr2	12	85746539	85813585	+	3504	protein_coding	feline leukemia virus subgroup C cellular receptor 2 [Source:MGI Symbol;Acc:MGI:2384974]	-
ENSMUSG0000002723	222.4667059	156.9367824	308.3357833	680.4515576	626.1690753	532.2449123	229.2464239	612.9551817	-1.419931486	2.70E-07	7.69E-06	Creb3l1	2	91982328	92024502	-	2603	protein_coding	cAMP responsive element binding protein 3-like 1 [Source:MGI Symbol;Acc:MGI:1347062]	TF_b
ENSMUSG0000005239	3.006306836	1.053266996	11.17158635	46.51187862	90.8636513	60.32757689	5.077053395	65.90103561	-3.716828361	2.71E-07	7.71E-06	Mogat2	7	99219084	99238619	-	1835	protein_coding	monoacylglycerol O-acyltransferase 2 [Source:MGI Symbol;Acc:MGI:2663253]	-
ENSMUSG0000002523	1022.144324	1227.05605	1132.798856	469.4254416	623.2061301	617.8711504	1127.333077	570.1675741	0.984039141	2.74E-07	7.79E-06	Parp6	9	59617284	59650285	+	7763	protein_coding	poly (ADP-ribose) polymerase family, member 6 [Source:MGI Symbol;Acc:MGI:1914537]	-
ENSMUSG0000003817	770.6166523	770.9914411	949.5848399	1336.785845	2873.069148	2959.94337	830.3976444	2389.932788	-1.525045871	2.75E-07	7.81E-06	Fam126b	1	58522806	58586323	-	12041	protein_coding	family with sequence similarity 126, member B [Source:MGI Symbol;Acc:MGI:1098784]	-
ENSMUSG0000003535	1015.129608	1160.70023	1063.535021	385.8763263	614.3172947	357.1003342	1079.788286	452.4313184	1.255484413	2.78E-07	7.86E-06	Nfkbiz	16	55811375	55838899	-	5018	protein_coding	nuclear factor of kappa light polypeptide gene enhancer in B cells inhibitor, zeta [Source:MGI Symbol;Acc:MGI:1931595]	-
ENSMUSG0000002248	286.6012517	193.8011273	182.0968575	483.206739	550.1201497	525.4337342	220.8330788	519.5868743	-1.232869042	2.80E-07	7.91E-06	Hoxc5	15	102967443	103017429	+	3089	protein_coding	homeobox C5 [Source:MGI Symbol;Acc:MGI:96196]	Hom
ENSMUSG0000002647	32563.31354	45868.72441	36780.21375	21620.27158	13993.0023	17618.57153	38404.0839	17743.94847	1.113908435	2.87E-07	8.11E-06	Glul	1	153899944	153909723	+	3323	protein_coding	glutamate-ammonia ligase (glutamine synthetase) [Source:MGI Symbol;Acc:MGI:95739]	-
ENSMUSG0000006660	42.0882957	27.3849419	37.9833936	155.9009265	130.3695867	101.1946451	35.81887707	129.1550528	-1.850674119	2.90E-07	8.17E-06	Insyn1	9	58488603	58499780	+	2331	protein_coding	inhibitory synaptic factor 1 [Source:MGI Symbol;Acc:MGI:2442108]	-
ENSMUSG0000001884	753.5809135	674.0908775	741.7933338	410.8549278	415.7999696	380.4529446	723.1550416	402.3692807	0.845690286	2.92E-07	8.21E-06	Unc45b	11	82910550	82943403	+	4493	protein_coding	unc-45 myosin chaperone B [Source:MGI Symbol;Acc:MGI:2443377]	-
ENSMUSG0000002867	210.4414785	271.742885	397.7084741	683.0355509	717.0327266	926.320213	293.2976125	775.4628301	-1.403653039	2.99E-07	8.39E-06	Gale	4	135963727	135968178	+	2370	protein_coding	galactose-4-epimerase, UDP [Source:MGI Symbol;Acc:MGI:1921496]	-
ENSMUSG0000010062	56.1177276	84.26135969	153.050733	17.22662171	10.86413222	18.48748324	97.80994011	15.52607906	2.651223285	2.99E-07	8.39E-06	A830008E24Ri	1	154037597	154117951	-	5637	lincRNA	RIKEN cDNA A830008E24 gene [Source:MGI Symbol;Acc:MGI:3041183]	-
ENSMUSG0000005929	8170.139878	6162.665194	6435.950898	4060.314737	3164.425422	3582.679647	6922.918656	3602.473269	0.942344332	3.03E-07	8.50E-06	Rpl11	4	136028265	136053428	-	2697	protein_coding	ribosomal protein L11 [Source:MGI Symbol;Acc:MGI:1914275]	-
ENSMUSG0000005870	395.8304001	401.2947255	409.9972191	671.8382468	797.0322457	725.8769736	402.3741149	731.5824887	-0.862251229	3.09E-07	8.65E-06	Memo1	17	74199055	74295521	-	7060	protein_coding	mediator of cell motility 1 [Source:MGI Symbol;Acc:MGI:1924140]	-
ENSMUSG0000004272	2870.020926	3234.582945	3764.824601	1834.635212	1796.53241	1912.968003	3289.809491	1848.045208	0.831887813	3.10E-07	8.66E-06	Trafd1	5	121371725	121385632	-	4486	protein_coding	TRAF type zinc finger domain containing 1 [Source:MGI Symbol;Acc:MGI:1923551]	-
ENSMUSG0000002250	280.588638	234.8785401	740.6761752	1356.59646	2264.677744	1556.840694	418.7144511	1726.038299	-2.044009822	3.14E-07	8.76E-06	Bcl6	16	23965052	23988852	-	6700	protein_coding	B cell leukemia/lymphoma 6 [Source:MGI Symbol;Acc:MGI:107187]	ZBTI
ENSMUSG0000003691	41.08619342	123.2322385	73.73246992	233.4207242	345.6769343	419.373962	79.35030063	332.8238735	-2.069016485	3.17E-07	8.82E-06	Trim67	8	124793092	124834713	+	8744	protein_coding	tripartite motif-containing 67 [Source:MGI Symbol;Acc:MGI:3045323]	-
ENSMUSG0000006131	6190.987877	6739.855508	8036.839222	4081.848015	3883.433445	4032.217398	6989.227536	3999.166286	0.805355783	3.19E-07	8.87E-06	Naca	10	128035575	128048637	+	6805	protein_coding	nascent polypeptide-associated complex alpha polypeptide [Source:MGI Symbol;Acc:MGI:106095]	-
ENSMUSG0000006824	1399.936883	1175.445968	885.9067977	602.0704288	408.8864309	424.2390891	1153.763216	478.3986496	1.269612348	3.20E-07	8.88E-06	Gm11808	4	3973092	3973595	-	504	protein_coding	predicted gene 11808 [Source:MGI Symbol;Acc:MGI:3649356]	-
ENSMUSG0000002929	37.07778431	28.43820889	22.3431727	112.8343722	108.6413222	98.27556881	29.28638864	106.5837544	-1.859724422	3.24E-07	8.99E-06	Abcg3	5	104935057	104982718	-	3550	protein_coding	ATP binding cassette subfamily G member 3 [Source:MGI Symbol;Acc:MGI:1351624]	-
ENSMUSG0000006721	34.07147747	41.07741285	58.09224903	6.029317599	1.975296767	2.919076301	44.41371312	3.641230223	3.584681228	3.26E-07	9.01E-06	Nipal1	5	72647795	72671078	+	4393	protein_coding	NIPA-like domain containing 1 [Source:MGI Symbol;Acc:MGI:1917951]	-
ENSMUSG0000007943	3354.036327	3351.495582	3619.593978	2012.069416	1125.919157	1157.900266	3441.708629	1431.962947	1.264749495	3.26E-07	9.02E-06	Rpl36a	X	134585654	134588062	+	1655	protein_coding	ribosomal protein L36A [Source:MGI Symbol;Acc:MGI:1201789]	-
ENSMUSG0000007972	40.08409114	40.02414585	36.86623496	0	3.950593535	0.973025434	38.99149065	1.641206323	4.618111105	3.32E-07	9.16E-06	Ttll2	17	7350885	7352696	-	1642	protein_coding	tubulin tyrosine ligase-like family, member 2 [Source:MGI Symbol;Acc:MGI:3644030]	-
ENSMUSG0000008341	365.7673317	664.6114745	433.4575505	130.0609939	123.456048	30.16378845	487.9454522	94.56027678	2.366173402	3.39E-07	9.33E-06	Rpl30-ps10	X	71803688	71804034	+	347	processed_pseudog	ribosomal protein L30, pseudogene 10 [Source:MGI Symbol;Acc:MGI:3646270]	-
ENSMUSG0000001509	2229.67757	1824.258437	1982.956578	1222.22881	924.4388872	1049.894443	2012.297528	1065.520714	0.916999297	3.40E-07	9.36E-06	Edf1	2	25557847	25562082	+	1317	protein_coding	endothelial differentiation-related factor 1 [Source:MGI Symbol;Acc:MGI:1891227]	-
ENSMUSG0000011134	17.03573874	11.58593696	26.81180725	117.1410276	71.11068363	93.41044164	18.47782765	93.8873843	-2.351156233	3.45E-07	9.48E-06	Gm19531	9	75310335	75314871	-	1377	antisense	predicted gene, 19531 [Source:MGI Symbol;Acc:MGI:5011716]	-
ENSMUSG0000002760	685.4379586	1026.935321	586.5082835	1403.108338	4612.317952	2777.014588	766.2938544	2930.813626	-1.935168898	3.51E-07	9.63E-06	Acss2	2	155517948	155585724	+	10163	protein_coding	acyl-CoA synthetase short-chain family member 2 [Source:MGI Symbol;Acc:MGI:1890410]	-
ENSMUSG0000002420	161.3384669	145.3508455	128.4732431	329.0284747	284.4427345	362.9384868	145.0541851	325.4698987	-1.164977537	3.52E-07	9.63E-06	Rfx2	17	56775897	56831013	-	7311	protein_coding	regulatory factor X, 2 (influences HLA class II expression) [Source:MGI Symbol;Acc:MGI:106583]	RFX
ENSMUSG0000001814	1074.253643	835.2407279	1226.640181	526.2732933	534.3177756	470.9443099	1045.378184	510.511793	1.033770302	3.58E-07	9.80E-06	Mafk	5	139791513	139802653	+	3901	protein_coding	v-maf musculoaponeurotic fibrosarcoma oncogene family, protein K (avian) [Source:MGI Symbol;Acc:MGI:99951]	TF_b
ENSMUSG0000003120	1.002102279	0	0	18.94928388	113.5795641	36.00194105	0.334034093	56.17692969	-7.211872693	3.65E-07	9.98E-06	Asb12	X	95470201	95557987	-	1594	protein_coding	ankyrin repeat and SOCS box-containing 12 [Source:MGI Symbol;Acc:MGI:1917642]	-
ENSMUSG0000005576	2318.864673	2400.395484	2377.313576	1500.438751	1091.351464	1264.933064	2365.524578	1285.574426	0.879373831	3.66E-07	9.98E-06	Eef1d	15	75894205	75909556	-	6488	protein_coding	eukaryotic translation elongation factor 1 delta (guanine nucleotide exchange protein) [Source:MGI Symbol;Acc:MGI:1913906]	-
ENSMUSG0000003084	183.384717	195.9076613	189.916968	821.7098557	372.3434407	492.3508695	189.7364487	562.1347219	-1.567709928	3.68E-07	1.00E-05	Fgfr2	7	130162451	133123350	-	10328	protein_coding	fibroblast growth factor receptor 2 [Source:MGI Symbol;Acc:MGI:95523]	-
ENSMUSG0000007955	336.7063656	361.2705797	720.5673197	140.396967	160.9866866	147.8998659	472.8480883	149.7611731	1.658387635	3.87E-07	1.05E-05	Colca2	9	51270258	51278569	-	1278	protein_coding	COLCA2 homolog [Source:MGI Symbol;Acc:MGI:2685530]	-
ENSMUSG0000004176	196.4120466	238.0383411	157.5193676	430.6655428	435.5529372	436.8884198	197.3232518	434.3689666	-1.137519663	3.87E-07	1.05E-05	Gpr155	2	73341506	73386572	-	6679	protein_coding	G protein-coupled receptor 155 [Source:MGI Symbol;Acc:MGI:1915776]	-
ENSMUSG0000011031	114.2396598	143.2443115	175.3939057	33.59191234	49.38241919	47.67824626	144.2926257	43.55085926	1.730540241	3.96E-07	1.07E-05	Gm45250	8	26151888	26155479	-	3592	lincRNA	predicted gene 45250 [Source:MGI Symbol;Acc:MGI:5791086]	-
ENSMUSG0000002113	189.3973307	99.00709763	141.8791467	357.4524005	401.9728922	382.3989955	143.4278583	380.6080961	-1.406805063	3.98E-07	1.08E-05	Galnt16	12	80518277	80630972	+	8047	protein_coding	polypeptide N-acetylgalactosaminyltransferase 16 [Source:MGI Symbol;Acc:MGI:1917754]	-
ENSMUSG0000005118	89.1871028	94.79402965	134.0590362	31.86925017	27.65415474	27.24471215	106.0133896	28.92270569	1.870820558	4.00E-07	1.08E-05	Zfp524	7	5014415	5018488	+	1436	protein_coding	zinc finger protein 524 [Source:MGI Symbol;Acc:MGI:1916740]	zf-C2
ENSMUSG0000002175	11197.49086	12998.368	11677.65921	6772.646326	7718.472119	7315.205211	11957.83936	7268.774552	0.718206557	4.04E-07	1.09E-05	Il6st	13	112464070	112510086	+	11099	protein_coding	interleukin 6 signal transducer [Source:MGI Symbol;Acc:MGI:96560]	-
ENSMUSG0000004509	310.6517064	280.169021	290.4612452	28.42392582	110.616619	60.32757689	293.7606575	66.45604057	2.148193228	4.11E-07	1.11E-05	Arhgef37	18	61493794	61536536	-	3234	protein_coding	Rho guanine nucleotide exchange factor (GEF) 37 [Source:MGI Symbol;Acc:MGI:3045339]	-
ENSMUSG0000005883	644.3517652	543.48577	628.9603116	1034.458634	1052.833177	1209.470614	605.5992823	1098.920808	-0.859492585	4.11E-07	1.11E-05	Rex1bd	8	70504292	70506752	-	1822	protein_coding	required for excision 1-B domain containing [Source:MGI Symbol;Acc:MGI:1913712]	-
ENSMUSG0000000640	564.1835829	417.0937305	2070.094951	167.0982306	183.7025994	198.4971885	1017.124088	183.0993395	2.47369628	4.12E-07	1.11E-05	Adamts4	1	171250421	171260637	+	4810	protein_coding	a disintegrin-like and metallopeptidase (reprolysin type) with thrombospondin type 1 motif, 4 [Source:MGI Symbol;Acc:MGI:1339	-
ENSMUSG0000002588	133.2796031	58.98295178	201.0885543	16.36529063	22.71591283	27.24471215	131.1170364	22.10863853	2.570642507	4.13E-07	1.11E-05	Snca	6	60731575	60829855	-	2703	protein_coding	synuclein, alpha [Source:MGI Symbol;Acc:MGI:1277151]	-
ENSMUSG0000002171	165.346876	183.2684573	34.63191769	524.5506311	676.5391429	785.2315251	127.7490837	662.1070997	-2.372213954	4.27E-07	1.14E-05	Rgs7bp	13	104945904	105054930	-	7077	protein_coding	regulator of G-protein signalling 7 binding protein [Source:MGI Symbol;Acc:MGI:106334]	-
ENSMUSG0000000645	29.06096608	48.45028182	21.22601407	175.7115415	95.80189322	174.1715526	32.91242066	148.5616624	-2.173413068	4.28E-07	1.15E-05	Actn3	19	4861223	4877909	-	2894	protein_coding	actinin alpha 3 [Source:MGI Symbol;Acc:MGI:99678]	-
ENSMUSG0000002059	20.04204557	13.69247095	11.17158635	85.27177747	79.99951908	60.32757689	14.96870096	75.19962448	-2.323586314	4.30E-07	1.15E-05	Nrcam	12	44328885	44601964	+	13358	protein_coding	neuronal cell adhesion molecule [Source:MGI Symbol;Acc:MGI:104750]	-
ENSMUSG0000003335	255.536081	346.5248417	454.6835645	151.5942711	79.99951908	81.73413644	352.2481624	104.4426422	1.750572109	4.33E-07	1.16E-05	Chst2	9	95399292	95406722	-	7328	protein_coding	carbohydrate sulfotransferase 2 [Source:MGI Symbol;Acc:MGI:1891160]	-
ENSMUSG0000002919	1522.193361	1478.786863	992.0368681	2342.820553	3538.744159	3446.456086	1331.005697	3109.340266	-1.223782719	4.38E-07	1.17E-05	D5Ertd579e	5	36600485	36696024	-	18420	protein_coding	DNA segment, Chr 5, ERATO Doi 579, expressed [Source:MGI Symbol;Acc:MGI:1261849]	-
ENSMUSG0000002517	296.6222745	707.7954214	592.0940767	190.3541699	153.0854995	162.4952474	532.1705908	168.6449723	1.656976094	4.39E-07	1.17E-05	Rab40b	11	121356125	121388251	-	2008	protein_coding	Rab40B, member RAS oncogene family [Source:MGI Symbol;Acc:MGI:2183451]	-
ENSMUSG0000002434	618.2971059	758.3522372	682.5839261	361.7590559	383.2075729	302.6109099	686.4110897	349.1925129	0.974791802	4.42E-07	1.17E-05	Tmem173	18	35733678	35740554	-	2302	protein_coding	transmembrane protein 173 [Source:MGI Symbol;Acc:MGI:1919762]	-
ENSMUSG0000004285	459.9649459	624.5873287	479.2610545	242.0340351	245.9244476	262.7168671	521.2711097	250.2251166	1.058982247	4.44E-07	1.18E-05	Zc3h6	2	128967402	129018563	+	5668	protein_coding	zinc finger CCCH type containing 6 [Source:MGI Symbol;Acc:MGI:1926001]	-
ENSMUSG0000003976	42.0882957	129.5518405	73.73246992	14.64262846	5.925890302	9.730254338	81.79086872	10.09959103	3.008051959	4.44E-07	1.18E-05	Il22ra2	10	19621998	19634681	+	2498	protein_coding	interleukin 22 receptor, alpha 2 [Source:MGI Symbol;Acc:MGI:2665114]	-
ENSMUSG0000005731	470.988071	606.6817898	315.0387351	1108.533107	1046.907287	1254.229784	464.2361986	1136.556726	-1.291265302	4.47E-07	1.18E-05	Arhgap24	5	102481391	102897937	+	8792	protein_coding	Rho GTPase activating protein 24 [Source:MGI Symbol;Acc:MGI:1922647]	-
ENSMUSG0000002068	1677.519214	1528.290411	2715.812642	803.6219029	837.5258294	879.6149921	1973.874089	840.2542415	1.232014614	4.48E-07	1.18E-05	Taf15	11	83473086	83506743	+	2919	protein_coding	TATA-box binding protein associated factor 15 [Source:MGI Symbol;Acc:MGI:1917689]	-
ENSMUSG0000007229	29.06096608	24.22514091	17.87453816	85.27177747	87.90070615	107.0327977	23.72021505	93.40176045	-1.972525198	4.50E-07	1.19E-05	Klf12	14	99870632	100284679	-	6985	protein_coding	Kruppel-like factor 12 [Source:MGI Symbol;Acc:MGI:1333796]	zf-C2
ENSMUSG0000003402	14.0294319	8.426135969	12.28874499	54.26385839	60.24655141	83.6801873	11.58143762	66.06353237	-2.509673612	4.52E-07	1.19E-05	Cd226	18	89197431	89270201	+	2357	protein_coding	CD226 antigen [Source:MGI Symbol;Acc:MGI:3039602]	-
ENSMUSG0000003903	740.5535839	288.5951569	662.4750707	124.8930074	167.9002252	190.712985	563.8746038	161.1687392	1.8076633	4.57E-07	1.20E-05	St6galnac5	3	152817081	152982246	-	6002	protein_coding	ST6 (alpha-N-acetyl-neuraminyl-2,3-beta-galactosyl-1,3)-N-acetylgalactosaminide alpha-2,6-sialyltransferase 5 [Source:MGI Sym	-
ENSMUSG0000002526	131.2753985	175.8955883	134.0590362	327.3058125	304.1957022	385.3180718	147.0766744	338.9398622	-1.204370666	4.58E-07	1.20E-05	Apex2	X	150519519	150589868	-	8744	protein_coding	apurinic/apyrimidinic endonuclease 2 [Source:MGI Symbol;Acc:MGI:1924872]	-
ENSMUSG0000003672	300.6306836	372.8565166	452.4492473	161.9302441	168.8878736	161.522222	375.3121492	164.1134466	1.19276275	4.58E-07	1.20E-05	Zscan12	13	21362820	21372289	+	5003	protein_coding	zinc finger and SCAN domain containing 12 [Source:MGI Symbol;Acc:MGI:1099444]	zf-C2
ENSMUSG0000006063	3096.496041	2614.208684	2974.993446	1708.880874	1609.866866	1821.503612	2895.232724	1713.417117	0.756811246	4.72E-07	1.24E-05	Rpl35a	16	33056453	33060189	+	3526	protein_coding	ribosomal protein L35A [Source:MGI Symbol;Acc:MGI:1928894]	-
ENSMUSG0000002215	2106.41899	1544.089416	1997.47964	4461.695023	3333.313295	3511.648791	1882.662682	3768.885703	-1.001444103	4.82E-07	1.26E-05	Dab2	15	6299788	6440712	+	7342	protein_coding	disabled 2, mitogen-responsive phosphoprotein [Source:MGI Symbol;Acc:MGI:109175]	-
ENSMUSG0000005802	92.19340963	191.6945933	159.7536848	24.97860148	47.40712242	31.13681388	147.8805626	34.50751259	2.102521382	4.87E-07	1.27E-05	Adtrp	13	41763148	41847684	-	2866	protein_coding	androgen dependent TFPI regulating protein [Source:MGI Symbol;Acc:MGI:1924596]	-
ENSMUSG0000007541	992.0812558	942.6739615	916.0700809	1621.886434	1469.620795	1690.145178	950.2750994	1593.884136	-0.7460469	4.94E-07	1.29E-05	Dolk	2	30284231	30286334	-	2104	protein_coding	dolichol kinase [Source:MGI Symbol;Acc:MGI:2677836]	-
ENSMUSG0000003740	889.8668234	603.5219888	943.9990468	383.2923331	343.7016375	234.4991295	812.4626196	320.4977001	1.34132381	5.15E-07	1.34E-05	Icam1	9	21015985	21028817	+	4360	protein_coding	intercellular adhesion molecule 1 [Source:MGI Symbol;Acc:MGI:96392]	-
ENSMUSG0000002620	1206.531143	1280.772667	1335.004569	793.2859298	763.4522006	744.3644568	1274.102793	767.0341958	0.731817064	5.20E-07	1.35E-05	Dnajb2	1	75236406	75245692	+	4249	protein_coding	DnaJ heat shock protein family (Hsp40) member B2 [Source:MGI Symbol;Acc:MGI:1928739]	-
ENSMUSG0000002014	290.6096608	362.3238467	237.9547893	585.7051382	659.7491203	656.7921678	296.9627656	634.0821421	-1.093650893	5.29E-07	1.37E-05	Dock2	11	34226815	34783892	-	11425	protein_coding	dedicator of cyto-kinesis 2 [Source:MGI Symbol;Acc:MGI:2149010]	-
ENSMUSG0000007058	153.3216486	125.3387725	196.6199198	50.81853405	58.27125464	50.59732256	158.4267803	53.22903708	1.573508343	5.44E-07	1.41E-05	Fv1	4	147868979	147870358	+	1380	protein_coding	Friend virus susceptibility 1 [Source:MGI Symbol;Acc:MGI:95595]	-
ENSMUSG0000006728	2796.86746	2270.843644	2625.322793	1514.220048	890.8588421	949.6728234	2564.344632	1118.250571	1.196918144	5.44E-07	1.41E-05	Rps28	17	33819027	33824562	-	1464	protein_coding	ribosomal protein S28 [Source:MGI Symbol;Acc:MGI:1859516]	-
ENSMUSG0000007910	650.3643788	1157.540429	285.9926106	12807.99324	4067.136044	6952.266724	697.9658061	7942.465337	-3.508305648	5.47E-07	1.42E-05	C7	15	4988762	5063740	-	2676	protein_coding	complement component 7 [Source:MGI Symbol;Acc:MGI:88235]	-
ENSMUSG0000002391	635.3328446	1043.787593	909.3671291	397.9349615	207.4061606	327.9095712	862.8291889	311.0835644	1.470733431	5.50E-07	1.42E-05	Pla2g7	17	43568098	43612201	+	2905	protein_coding	phospholipase A2, group VII (platelet-activating factor acetylhydrolase, plasma) [Source:MGI Symbol;Acc:MGI:1351327]	-
ENSMUSG0000000194	305.641195	532.9531	671.4123398	142.9809602	197.5296767	162.4952474	503.3355449	167.6686281	1.586085823	5.50E-07	1.42E-05	Vsig2	9	37539255	37544205	+	1979	protein_coding	V-set and immunoglobulin domain containing 2 [Source:MGI Symbol;Acc:MGI:1928009]	-
ENSMUSG0000002754	141.2964213	126.3920395	75.96678719	265.2899744	336.7880989	378.5068937	114.5517493	326.8616557	-1.510137643	5.56E-07	1.43E-05	Nfatc2	2	168476410	168601657	-	10578	protein_coding	nuclear factor of activated T cells, cytoplasmic, calcineurin dependent 2 [Source:MGI Symbol;Acc:MGI:102463]	RHD
ENSMUSG0000004570	322.6769337	160.0965834	676.9981329	80.10379096	63.20949656	91.46439078	386.59055	78.2592261	2.303786267	5.64E-07	1.45E-05	Smkr-ps	6	29985329	29993776	+	907	lincRNA	smal lysine rich protein 1, pseudogene [Source:MGI Symbol;Acc:MGI:1923669]	-
ENSMUSG0000002666	5367.259804	4928.236275	6850.416751	2511.641446	3187.141334	3102.978108	5715.304277	2933.920296	0.962056779	5.64E-07	1.45E-05	Phyh	2	4919019	4938730	+	1893	protein_coding	phytanoyl-CoA hydroxylase [Source:MGI Symbol;Acc:MGI:891978]	-
ENSMUSG0000007342	2405.045469	1009.029782	913.8357636	5459.11642	3720.471462	5490.782523	1442.637005	4890.123468	-1.760967164	5.70E-07	1.46E-05	H2-Ab1	17	34263209	34269418	+	1637	protein_coding	histocompatibility 2, class II antigen A, beta 1 [Source:MGI Symbol;Acc:MGI:103070]	-
ENSMUSG0000009095	1752.676885	1294.465138	1292.552541	710.5981456	800.9828392	724.9039482	1446.564855	745.4949777	0.956737454	5.79E-07	1.48E-05	Lrrc32	7	98489283	98502181	+	4688	protein_coding	leucine rich repeat containing 32 [Source:MGI Symbol;Acc:MGI:93882]	-
ENSMUSG0000003130	3308.941724	3092.391901	2609.682572	4633.099909	9016.242095	9127.951594	3003.672065	7592.4312	-1.337736012	5.79E-07	1.48E-05	Rps6ka3	X	159210307	159368244	+	8322	protein_coding	ribosomal protein S6 kinase polypeptide 3 [Source:MGI Symbol;Acc:MGI:104557]	-
ENSMUSG0000003914	1282.690917	1271.293264	1438.900322	825.15518	685.4279783	763.8249655	1330.961501	758.1360413	0.811432043	5.82E-07	1.49E-05	Sart1	19	5377523	5388703	-	5091	protein_coding	squamous cell carcinoma antigen recognized by T cells 1 [Source:MGI Symbol;Acc:MGI:1309453]	-
ENSMUSG0000002527	7.01471595	1.053266996	1.117158635	49.09587188	41.48123212	46.70522082	3.061713861	45.76077494	-3.874364436	5.86E-07	1.49E-05	Dnase1l3	14	7964533	7994573	-	5151	protein_coding	deoxyribonuclease 1-like 3 [Source:MGI Symbol;Acc:MGI:1314633]	-
ENSMUSG0000004028	6316.250662	3299.885499	7499.485918	1396.21769	2354.553747	2071.571149	5705.20736	1940.780862	1.555745594	5.89E-07	1.50E-05	Btnl9	11	49165585	49187159	-	6054	protein_coding	butyrophilin-like 9 [Source:MGI Symbol;Acc:MGI:2442439]	-
ENSMUSG0000002028	804.6881297	688.8366154	587.6254421	1433.254926	1398.510111	1188.064055	693.7167291	1339.943031	-0.949383507	5.99E-07	1.52E-05	Rhbdf1	11	32209585	32222300	-	5433	protein_coding	rhomboid 5 homolog 1 [Source:MGI Symbol;Acc:MGI:104328]	-
ENSMUSG0000003009	63.13244355	45.29048083	70.38099402	186.0475145	162.9619833	155.6840694	59.60130613	168.2311891	-1.498575108	5.99E-07	1.52E-05	Nup210	6	91013068	91116829	-	10474	protein_coding	nucleoporin 210 [Source:MGI Symbol;Acc:MGI:1859555]	-
ENSMUSG0000003150	38.07988659	45.29048083	61.44372494	133.5063183	153.0854995	148.8728914	48.27136412	145.154903	-1.591333075	6.01E-07	1.52E-05	Ptpn7	1	135132700	135145317	+	6102	protein_coding	protein tyrosine phosphatase, non-receptor type 7 [Source:MGI Symbol;Acc:MGI:2156893]	-
ENSMUSG0000008676	42.0882957	203.2805302	391.0055223	32.73058125	8.888835454	16.54143237	212.1247828	19.38694969	3.44532843	6.01E-07	1.52E-05	Gm11827	4	11122805	11126998	+	1368	lincRNA	predicted gene 11827 [Source:MGI Symbol;Acc:MGI:3649801]	-
ENSMUSG0000002408	136.2859099	70.56888874	65.91235948	233.4207242	349.6275278	325.9635203	90.92238604	303.0039241	-1.733596243	6.02E-07	1.52E-05	Qpct	17	79051906	79090378	+	2539	protein_coding	glutaminyl-peptide cyclotransferase (glutaminyl cyclase) [Source:MGI Symbol;Acc:MGI:1917786]	-
ENSMUSG0000006657	2216.65024	2533.107126	2119.249931	809.6512205	1268.140525	1197.794309	2289.669099	1091.862018	1.068782413	6.03E-07	1.52E-05	4931406P16Ri	7	34236707	34313551	-	8239	protein_coding	RIKEN cDNA 4931406P16 gene [Source:MGI Symbol;Acc:MGI:1924311]	-
ENSMUSG0000010807	20.04204557	30.54474289	90.48984945	0.861331086	2.962945151	0.973025434	47.02554597	1.599100557	4.896213681	6.05E-07	1.53E-05	Gm45061	6	58973197	58975291	+	2095	TEC	predicted gene 45061 [Source:MGI Symbol;Acc:MGI:5753637]	-
ENSMUSG0000005388	169.3552851	123.2322385	121.7702912	47.37320971	46.41947404	45.73219539	138.1192716	46.50829304	1.571093063	6.08E-07	1.53E-05	Sh2d4a	8	68276567	68347699	+	2723	protein_coding	SH2 domain containing 4A [Source:MGI Symbol;Acc:MGI:1919531]	-
ENSMUSG0000006410	634.3307424	646.7059356	540.7047794	1016.370681	1163.449796	1426.455286	607.2471525	1202.091921	-0.984691316	6.14E-07	1.55E-05	Ccnm2	19	46761596	46878795	+	3558	protein_coding	cyclin M2 [Source:MGI Symbol;Acc:MGI:2151054]	-
ENSMUSG0000003013	84.1765914	63.19601977	55.85793176	5.167986514	16.79002252	7.78420347	67.74351431	9.914070836	2.785349589	6.17E-07	1.55E-05	Rasgef1a	6	118011438	118091546	+	3355	protein_coding	RasGEF domain family, member 1A [Source:MGI Symbol;Acc:MGI:1917977]	-
ENSMUSG0000003025	71.14926178	57.92968479	88.25553218	293.7139002	195.55438	176.1176035	72.44482625	221.7952946	-1.616650643	6.20E-07	1.56E-05	Bhlhe41	6	145858243	145865558	-	6720	protein_coding	basic helix-loop-helix family, member e41 [Source:MGI Symbol;Acc:MGI:1930704]	bHLI
ENSMUSG0000002656	2.004204557	5.26633498	0	41.34389211	43.45652888	39.89404278	2.423513179	41.56482126	-4.085206353	6.20E-07	1.56E-05	Adcy10	1	165485183	165576774	+	8739	protein_coding	adenylate cyclase 10 [Source:MGI Symbol;Acc:MGI:2660854]	-
ENSMUSG0000004177	712.4947201	676.1974115	1228.874499	347.9777586	367.4051988	321.0983931	872.5222101	345.4937835	1.336151701	6.22E-07	1.56E-05	Mapk1ip1	7	138835823	138846273	-	1883	protein_coding	mitogen-activated protein kinase 1 interacting protein 1 [Source:MGI Symbol;Acc:MGI:1916796]	-
ENSMUSG0000002281	701.471595	664.6114745	592.0940767	355.7297383	375.3063858	343.4779781	652.7257154	358.1713674	0.866251604	6.34E-07	1.58E-05	Umps	16	33954782	33967038	-	3484	protein_coding	uridine monophosphate synthetase [Source:MGI Symbol;Acc:MGI:1298388]	-
ENSMUSG0000002537	282.5928426	270.689618	173.1595885	643.4143209	479.0094661	669.4414984	242.1473497	597.2884285	-1.301690119	6.34E-07	1.58E-05	Aatk	11	120007313	120047167	-	7159	protein_coding	apoptosis-associated tyrosine kinase [Source:MGI Symbol;Acc:MGI:1197518]	-
ENSMUSG0000002519	84.1765914	106.3799666	82.66973901	208.4421227	213.3320509	225.7419006	91.07543234	215.8386914	-1.244416005	6.38E-07	1.59E-05	Entpd7	19	43689672	43733697	+	8963	protein_coding	ectonucleoside triphosphate diphosphohydrolase 7 [Source:MGI Symbol;Acc:MGI:2135885]	-
ENSMUSG0000003043	5974.533785	5646.564366	4899.857774	3374.695193	2121.468728	2208.767735	5506.985308	2568.310552	1.100271448	6.41E-07	1.60E-05	Rpl28	7	4792874	4795066	+	1840	protein_coding	ribosomal protein L28 [Source:MGI Symbol;Acc:MGI:101839]	-
ENSMUSG0000002109	846.7764254	869.9985388	551.8763658	1370.377757	2176.777038	1889.615392	756.21711	1812.256729	-1.260421955	6.50E-07	1.62E-05	Dhrs7	12	72650353	72664909	-	3541	protein_coding	dehydrogenase/reductase (SDR family) member 7 [Source:MGI Symbol;Acc:MGI:1913625]	-
ENSMUSG0000003464	41.08619342	61.08948577	18.9916968	338.5031166	96.78954161	312.3411642	40.38912533	249.2112742	-2.623891728	6.52E-07	1.62E-05	Lrrn1	6	107529768	107570214	+	3700	protein_coding	leucine rich repeat protein 1, neuronal [Source:MGI Symbol;Acc:MGI:106038]	-
ENSMUSG0000002459	14.0294319	6.319601977	13.40590362	56.84785165	64.19714494	71.03085667	11.25164583	64.02528442	-2.50765869	6.55E-07	1.62E-05	Megf10	18	57133090	57297467	+	7718	protein_coding	multiple EGF-like-domains 10 [Source:MGI Symbol;Acc:MGI:2685177]	-
ENSMUSG0000002304	38.07988659	63.19601977	80.43542174	7.75197977	9.876483837	1.946050868	60.5704427	6.524838158	3.210398732	6.63E-07	1.64E-05	Soat2	15	102150526	102163469	+	2213	protein_coding	sterol O-acyltransferase 2 [Source:MGI Symbol;Acc:MGI:1332226]	-
ENSMUSG0000004579	300.6306836	440.2656044	358.6079219	180.0181969	148.1472576	144.9807896	366.5014033	157.7154147	1.215100475	6.65E-07	1.65E-05	Gm9800	14	8800000	8800335	-	336	processed_pseudog	predicted gene 9800 [Source:MGI Symbol;Acc:MGI:3710580]	-
ENSMUSG0000003285	57.11982988	26.3316749	41.3348695	131.7836561	155.0607962	148.8728914	41.5954581	145.2391146	-1.801301457	6.68E-07	1.65E-05	Rnft2	5	118190736	118245116	-	5593	protein_coding	ring finger protein, transmembrane 2 [Source:MGI Symbol;Acc:MGI:2442859]	-
ENSMUSG0000003430	346.7273884	512.9410271	555.2278417	155.9009265	181.7273026	225.7419006	471.6320857	187.7900432	1.328935045	6.72E-07	1.66E-05	Sdr42e1	8	117661399	117673689	-	2957	protein_coding	short chain dehydrogenase/reductase family 42E, member 1 [Source:MGI Symbol;Acc:MGI:1921282]	-
ENSMUSG0000006689	400.8409114	476.0766822	620.0230425	222.2234201	219.2579412	233.5261041	498.9802121	225.0024885	1.148438879	6.75E-07	1.66E-05	Fbxl12	9	20618118	20644767	-	5090	protein_coding	F-box and leucine-rich repeat protein 12 [Source:MGI Symbol;Acc:MGI:1354738]	-
ENSMUSG0000002078	1916.019557	1348.181755	1887.998094	956.9388361	786.1681135	823.179517	1717.399802	855.4288222	1.005228936	6.80E-07	1.68E-05	Atp2a3	11	72961169	72993044	+	5281	protein_coding	ATPase, Ca++ transporting, ubiquitous [Source:MGI Symbol;Acc:MGI:1194503]	-
ENSMUSG0000002657	2752.774959	934.2478255	1800.85972	5373.844643	5294.782985	6390.831049	1829.294168	5686.486226	-1.636155321	6.86E-07	1.69E-05	Dpt	1	164796644	164824266	+	1724	protein_coding	dermatopontin [Source:MGI Symbol;Acc:MGI:1928392]	-
ENSMUSG0000003127	179.3763079	159.0433164	121.7702912	558.1425435	297.2821635	428.1311909	153.3966385	427.8519659	-1.479517127	6.92E-07	1.70E-05	Col4a6	X	141165403	141474076	-	7973	protein_coding	collagen, type IV, alpha 6 [Source:MGI Symbol;Acc:MGI:2152695]	-
ENSMUSG0000009158	985.0665399	1095.397676	767.4879824	355.7297383	510.6142144	446.6186741	949.3173994	437.654209	1.117989581	7.09E-07	1.74E-05	Cyp4f17	17	32506462	32528894	+	1693	protein_coding	cytochrome P450, family 4, subfamily f, polypeptide 17 [Source:MGI Symbol;Acc:MGI:3646233]	-
ENSMUSG0000003026	771.6187545	891.0638787	726.1531129	447.8921645	391.10876	450.5107758	796.278582	429.8372334	0.889304184	7.14E-07	1.75E-05	Mtmr14	6	113237843	113281392	+	3006	protein_coding	myotubularin related protein 14 [Source:MGI Symbol;Acc:MGI:1916075]	-
ENSMUSG0000000394	6700.055835	3657.996277	5382.470304	9248.111866	16515.45627	16459.69824	5246.840806	14074.42213	-1.423512431	7.30E-07	1.79E-05	Mmd	11	90249456	90278589	+	3581	protein_coding	monocyte to macrophage differentiation-associated [Source:MGI Symbol;Acc:MGI:1914718]	-
ENSMUSG0000002078	1626.411998	1695.759864	1494.758254	995.6987349	985.673087	965.2412303	1605.643372	982.2043507	0.709111149	7.34E-07	1.79E-05	Srp68	11	116245166	116274217	-	2737	protein_coding	signal recognition particle 68 [Source:MGI Symbol;Acc:MGI:1917447]	-
ENSMUSG0000010760	38.07988659	42.13067984	29.04612452	3.445324342	2.962945151	2.919076301	36.41889698	3.109115265	3.546983697	7.42E-07	1.81E-05	Gm44117	6	90459711	90461468	-	1758	lincRNA	predicted gene, 44117 [Source:MGI Symbol;Acc:MGI:5690509]	-
ENSMUSG0000000306	2838.955755	2477.283975	3034.202853	1764.867394	1514.064972	1482.890761	2783.480861	1587.274376	0.81009024	7.49E-07	1.82E-05	Stk11	10	80115803	80130682	+	4580	protein_coding	serine/threonine kinase 11 [Source:MGI Symbol;Acc:MGI:1341870]	-
ENSMUSG0000006096	1034.169552	1273.399798	1692.495332	665.8089292	555.0583917	627.6014048	1333.354894	616.1562419	1.113184224	7.51E-07	1.83E-05	Slc4a4	5	88886818	89239653	+	10815	protein_coding	solute carrier family 4 (anion exchanger), member 4 [Source:MGI Symbol;Acc:MGI:1927555]	-
ENSMUSG0000000190	931.9551191	910.0226846	1038.957531	586.5664693	532.3424788	475.8094371	960.3117782	531.5727951	0.852593543	7.51E-07	1.83E-05	Trmt1	8	84686307	84699808	+	6306	protein_coding	tRNA methyltransferase 1 [Source:MGI Symbol;Acc:MGI:1289155]	-
ENSMUSG0000006264	2523.293538	1837.950908	1797.508244	1083.554506	492.8365435	581.8692094	2052.917563	719.4200862	1.51223405	7.52E-07	1.83E-05	Rpl7a	2	26910764	26913318	+	1781	protein_coding	ribosomal protein L7A [Source:MGI Symbol;Acc:MGI:1353472]	-
ENSMUSG0000008588	243.5108537	153.7769814	309.452942	24.1172704	81.97481585	39.89404278	235.580259	48.66204301	2.279210498	7.56E-07	1.83E-05	Gm15912	13	74008009	74009354	-	1297	processed_transcrip	predicted gene 15912 [Source:MGI Symbol;Acc:MGI:3801895]	-
ENSMUSG0000008672	32.06727292	12.63920395	31.28044179	96.46908159	105.6783771	138.1696116	25.32897289	113.4390234	-2.162625127	7.66E-07	1.86E-05	4931428L18Ri	1	30997842	31222684	-	3751	protein_coding	RIKEN cDNA 4931428L18 gene [Source:MGI Symbol;Acc:MGI:1918238]	-
ENSMUSG0000006758	229.4814218	362.3238467	90.48984945	865.637741	868.1429293	907.8327297	227.431706	880.5378	-1.952225025	7.72E-07	1.87E-05	S1pr3	13	51408639	51422797	+	4463	protein_coding	sphingosine-1-phosphate receptor 3 [Source:MGI Symbol;Acc:MGI:1339365]	-
ENSMUSG0000002090	363.7631271	707.7954214	729.5045888	178.2955347	225.1838315	236.4451804	600.3543791	213.3081822	1.49309831	7.76E-07	1.87E-05	Pik3r5	11	68432121	68497849	+	5550	protein_coding	phosphoinositide-3-kinase regulatory subunit 5 [Source:MGI Symbol;Acc:MGI:2443588]	-
ENSMUSG0000010799	55.11562532	49.50354882	103.8957531	12.91996628	9.876483837	11.67630521	69.50497574	11.49091844	2.591948036	7.79E-07	1.88E-05	Gm44243	6	129540221	129540912	+	692	TEC	predicted gene, 44243 [Source:MGI Symbol;Acc:MGI:5690635]	-
ENSMUSG0000002210	189.3973307	112.6995686	195.5027612	374.6790222	428.6393985	435.9153943	165.8665535	413.0779384	-1.316351575	8.04E-07	1.94E-05	Dok2	14	70766036	70778495	+	2902	protein_coding	docking protein 2 [Source:MGI Symbol;Acc:MGI:1332623]	-
ENSMUSG0000004297	264.5550016	272.796152	232.3689961	451.3374888	519.5030499	499.1620475	256.5733832	490.0008621	-0.932496482	8.08E-07	1.94E-05	Sbk1	7	126272619	126295016	+	4227	protein_coding	SH3-binding kinase 1 [Source:MGI Symbol;Acc:MGI:2135937]	-
ENSMUSG0000002071	12437.09138	18381.61562	9943.829012	6284.2716	6086.876989	5945.1854	13587.512	6105.446663	1.154117473	8.11E-07	1.95E-05	Pecam1	11	106654217	106750628	-	6228	protein_coding	platelet/endothelial cell adhesion molecule 1 [Source:MGI Symbol;Acc:MGI:97537]	-
ENSMUSG0000004621	58.12193216	10.53266996	50.27213858	344.5324342	163.9496317	215.0386209	39.6422469	241.1735623	-2.605114147	8.15E-07	1.95E-05	Rprml	11	103649570	103650579	+	1010	protein_coding	reprimo-like [Source:MGI Symbol;Acc:MGI:2144486]	-
ENSMUSG0000002121	7202.109076	9074.948438	5259.582855	3751.096878	2688.378901	3159.413583	7178.880123	3199.629787	1.165801729	8.22E-07	1.97E-05	Net1	13	3882018	3918220	-	5217	protein_coding	neuroepithelial cell transforming gene 1 [Source:MGI Symbol;Acc:MGI:1927138]	-
ENSMUSG0000002931	1106.320916	785.7371791	407.7629019	1912.15501	2657.761801	2407.264923	766.6069988	2325.727245	-1.600632045	8.26E-07	1.98E-05	Gpat3	5	100845713	100899102	+	3502	protein_coding	glycerol-3-phosphate acyltransferase 3 [Source:MGI Symbol;Acc:MGI:3603816]	-
ENSMUSG0000003184	2556.362913	1828.471505	3305.672402	1282.521986	1048.882584	1048.921418	2563.502273	1126.775329	1.185664223	8.34E-07	1.99E-05	Cdh13	8	118283733	119324921	+	11233	protein_coding	cadherin 13 [Source:MGI Symbol;Acc:MGI:99551]	-
ENSMUSG0000003806	1002.102279	598.2556538	1059.066386	2016.376071	1809.371839	2477.322754	886.4747729	2101.023555	-1.244995748	8.46E-07	2.02E-05	Mturn	6	54681624	54703851	+	5695	protein_coding	maturin, neural progenitor differentiation regulator homolog (Xenopus) [Source:MGI Symbol;Acc:MGI:1915485]	-
ENSMUSG0000002647	5424.379634	5503.320055	5182.498909	7830.360899	10376.23392	10280.98673	5370.066199	9495.860517	-0.822923898	8.50E-07	2.03E-05	Lamc1	1	153218922	153332786	-	8272	protein_coding	laminin, gamma 1 [Source:MGI Symbol;Acc:MGI:99914]	-
ENSMUSG0000000789	12350.91058	12097.82472	10404.09837	7581.436215	5984.161557	5874.154544	11617.61122	6479.917439	0.842214761	8.67E-07	2.06E-05	Rplp1	9	61913284	61914542	-	499	protein_coding	ribosomal protein, large, P1 [Source:MGI Symbol;Acc:MGI:1927099]	-
ENSMUSG0000006715	129.2711939	52.6633498	67.02951811	857.0244302	236.0479637	288.9885538	82.98802062	460.6869826	-2.472159462	8.80E-07	2.09E-05	Col4a4	1	82448423	82586849	-	10612	protein_coding	collagen, type IV, alpha 4 [Source:MGI Symbol;Acc:MGI:104687]	-
ENSMUSG0000004209	2608.472291	3964.496973	4114.495254	1806.211286	1790.60652	1475.106558	3562.488153	1690.641455	1.075165672	8.85E-07	2.10E-05	Kank3	17	33810520	33822918	+	3118	protein_coding	KN motif and ankyrin repeat domains 3 [Source:MGI Symbol;Acc:MGI:1098615]	-
ENSMUSG0000003817	1885.956488	4380.537437	2644.31449	1097.335803	1030.117264	1192.929182	2970.269472	1106.794083	1.424174617	8.90E-07	2.11E-05	Mylip	13	45389742	45412022	+	3233	protein_coding	myosin regulatory light chain interacting protein [Source:MGI Symbol;Acc:MGI:2388271]	-
ENSMUSG0000003208	42.0882957	51.61008281	130.7075603	11.19730411	2.962945151	0.973025434	74.80197961	5.044424899	3.863753309	8.90E-07	2.11E-05	Apoa1	9	46228580	46230466	+	1236	protein_coding	apolipoprotein A-I [Source:MGI Symbol;Acc:MGI:88049]	-
ENSMUSG0000009704	311.6538087	372.8565166	632.3117875	141.258298	153.0854995	170.2794509	438.9407043	154.8744161	1.502643815	9.12E-07	2.16E-05	Gm17491	8	22480291	22483332	+	2311	sense_intronic	predicted gene, 17491 [Source:MGI Symbol;Acc:MGI:4937125]	-
ENSMUSG0000003100	42.0882957	65.30255376	119.535974	349.7004207	199.5049735	368.7766394	75.64227448	305.9940112	-2.019510367	9.15E-07	2.16E-05	Mki67	7	135689784	135716361	-	10061	protein_coding	antigen identified by monoclonal antibody Ki 67 [Source:MGI Symbol;Acc:MGI:106035]	-
ENSMUSG0000001481	110.2312506	154.8302484	385.4197292	43.06655428	48.3947708	39.89404278	216.8270761	43.78512262	2.307154971	9.61E-07	2.27E-05	Stc1	14	69029238	69041804	+	4197	protein_coding	stanniocalcin 1 [Source:MGI Symbol;Acc:MGI:109131]	-
ENSMUSG0000004953	4.008409114	0	0	39.62122994	35.55534181	43.78614452	1.336136371	39.65423876	-4.8380539	9.62E-07	2.27E-05	Adamts16	13	70727802	70841811	-	6836	protein_coding	a disintegrin-like and metallopeptidase (reprolysin type) with thrombospondin type 1 motif, 16 [Source:MGI Symbol;Acc:MGI:242	-
ENSMUSG0000007851	1532.214384	2897.537506	3704.498034	898.3683223	1083.450277	987.6208153	2711.416667	989.8131382	1.453779206	9.74E-07	2.29E-05	Ddit2	4	141677549	141723419	-	9553	protein_coding	DNA-damage inducible protein 2 [Source:MGI Symbol;Acc:MGI:1917244]	-
ENSMUSG0000002849	11446.01223	15992.80607	13126.61396	8171.448009	6837.489761	6565.975627	13521.81075	7191.637799	0.910844587	9.78E-07	2.30E-05	Rps6	4	86854660	86857412	-	1586	protein_coding	ribosomal protein S6 [Source:MGI Symbol;Acc:MGI:98159]	-
ENSMUSG0000003815	468.9838664	396.0283905	786.4796792	1819.131253	1177.276873	1433.266464	550.497312	1476.558197	-1.424082388	9.81E-07	2.30E-05	Spon1	7	113765998	114043370	+	10382	protein_coding	spondin 1, (f-spondin) extracellular matrix protein [Source:MGI Symbol;Acc:MGI:2385287]	-
ENSMUSG0000001713	1419.978929	1489.319532	1184.188153	765.7233351	812.8346198	750.2026094	1364.495538	776.2535215	0.814023182	9.93E-07	2.33E-05	Cyth1	11	118132019	118248592	-	3905	protein_coding	cytohesin 1 [Source:MGI Symbol;Acc:MGI:1334257]	-
ENSMUSG0000002043	149.3132395	194.8543943	217.8459339	409.1322657	420.7382115	376.5608429	187.3378559	402.1437733	-1.103506891	1.00E-06	2.35E-05	Myo1g	11	6506548	6520965	-	6957	protein_coding	myosin IG [Source:MGI Symbol;Acc:MGI:1927091]	-
ENSMUSG0000000186	95.19971647	77.94175771	64.79520084	217.9167647	191.6037864	190.712985	79.31222501	200.0778454	-1.333171698	1.01E-06	2.36E-05	Cpa3	3	20215620	20242181	-	1517	protein_coding	carboxypeptidase A3, mast cell [Source:MGI Symbol;Acc:MGI:88479]	-
ENSMUSG0000001030	845.7743232	679.3572125	585.3911249	372.095029	244.9367992	313.3141897	703.5075535	310.1153393	1.181189215	1.03E-06	2.40E-05	Tmem86a	7	47050601	47054777	+	1887	protein_coding	transmembrane protein 86A [Source:MGI Symbol;Acc:MGI:1915143]	-
ENSMUSG0000002038	1891.969102	1818.992102	2537.067261	1058.575904	1132.832696	1129.682529	2082.676155	1107.030376	0.911661177	1.05E-06	2.44E-05	Clk4	11	51261730	51281766	+	5797	protein_coding	CDC like kinase 4 [Source:MGI Symbol;Acc:MGI:1098551]	-
ENSMUSG0000005815	11.02312506	13.69247095	5.585793176	125.7543385	31.60474828	104.1137214	10.10046306	87.15760273	-3.105929506	1.05E-06	2.44E-05	Sez6l	5	112419151	112577185	-	6070	protein_coding	seizure related 6 homolog like [Source:MGI Symbol;Acc:MGI:1935121]	-
ENSMUSG0000001036	622.305515	417.0937305	767.4879824	267.0126365	250.8626895	212.1195446	602.2957426	243.3316235	1.30690698	1.06E-06	2.47E-05	Rdm1	11	101627195	101636100	+	1575	protein_coding	RAD52 motif 1 [Source:MGI Symbol;Acc:MGI:1913849]	-
ENSMUSG0000003466	441.9271049	469.7570803	499.3699099	790.7019366	786.1681135	848.4781783	470.351365	808.4494094	-0.781727097	1.11E-06	2.58E-05	Bmp2k	5	96997689	97091867	+	8480	protein_coding	BMP2 inducible kinase [Source:MGI Symbol;Acc:MGI:2155456]	-
ENSMUSG0000002886	176.370001	360.2173127	278.1725002	37.03723668	83.95011262	88.54531447	271.5866046	69.84422126	1.962046751	1.13E-06	2.62E-05	Cd164l2	4	133212131	133224555	+	1827	protein_coding	CD164 sialomucin-like 2 [Source:MGI Symbol;Acc:MGI:1916905]	-
ENSMUSG0000003747	9.01892050 8	6.319601977	7.820110447	43.06655428	59.25890302	47.67824626	7.71954431	50.00123452	-2.692501274	1.14E-06	2.64E-05	Dtl	1	191537356	191575544	-	13709	protein_coding	denticleless E3 ubiquitin protein ligase [Source:MGI Symbol;Acc:MGI:1924093]	-
ENSMUSG0000004820	461.9691504	597.2023868	675.8809743	298.0205556	259.7515249	201.4162648	578.3508372	253.0627818	1.19119609	1.17E-06	2.70E-05	Cracr2b	7	141461094	141466613	+	2764	protein_coding	calcium release activated channel regulator 2B [Source:MGI Symbol;Acc:MGI:2446129]	-
ENSMUSG0000011177	5.010511393	3.159800988	4.468634541	29.28525691	46.41947404	49.62429712	4.212982307	41.77634269	-3.306340059	1.18E-06	2.73E-05	Gm38398	9	83098224	83106330	+	584	lincRNA	predicted gene, 38398 [Source:MGI Symbol;Acc:MGI:5618692]	-
ENSMUSG0000004142	4368.163832	3478.940888	4599.342101	1621.025103	2242.949479	2344.01827	4148.815607	2069.330951	1.003710474	1.18E-06	2.73E-05	Hibch	1	52844929	52920986	+	6059	protein_coding	3-hydroxyisobutyryl-Coenzyme A hydrolase [Source:MGI Symbol;Acc:MGI:1923792]	-
ENSMUSG0000006178	5880.336171	5318.99833	5417.102222	3636.539843	2715.045407	3143.845177	5538.812241	3165.143476	0.807182892	1.18E-06	2.73E-05	Rps17	7	81342732	81345254	-	1274	protein_coding	ribosomal protein S17 [Source:MGI Symbol;Acc:MGI:1309526]	-
ENSMUSG0000002587	458.9628436	328.6193028	369.7795083	755.3873621	699.2550557	842.6400257	385.7872182	765.7608145	-0.988579627	1.21E-06	2.79E-05	Tspan17	13	54789377	54796776	+	1657	protein_coding	tetraspanin 17 [Source:MGI Symbol;Acc:MGI:1921507]	-
ENSMUSG0000003968	71.14926178	43.18394684	42.45202814	137.8129737	173.8261155	171.2524763	52.26174559	160.9638552	-1.618992024	1.21E-06	2.79E-05	Sdk1	5	141241490	142215586	+	11087	protein_coding	sidekick cell adhesion molecule 1 [Source:MGI Symbol;Acc:MGI:2444413]	-
ENSMUSG0000007474	5618.787476	3684.327952	5140.046881	8546.988362	9430.066768	11112.92348	4814.387436	9696.659537	-1.01010192	1.25E-06	2.86E-05	Thbd	2	148404466	148408188	-	3723	protein_coding	thrombomodulin [Source:MGI Symbol;Acc:MGI:98736]	-
ENSMUSG0000006856	2387.007628	2184.47575	2934.775735	3960.400332	5510.090333	5230.011707	2502.086371	4900.167457	-0.969709147	1.25E-06	2.86E-05	Myadm	7	3289080	3300442	+	4480	protein_coding	myeloid-associated differentiation marker [Source:MGI Symbol;Acc:MGI:1355332]	-
ENSMUSG0000003178	4193.798036	5494.893919	5562.332845	2932.832346	1621.718646	1520.838753	5083.674933	2025.129915	1.327598256	1.25E-06	2.86E-05	Adgrg1	8	94974751	95014217	+	5421	protein_coding	adhesion G protein-coupled receptor G1 [Source:MGI Symbol;Acc:MGI:1340051]	-
ENSMUSG0000008528	122.256478	121.1257046	144.1134639	47.37320971	39.50593535	50.59732256	129.1652155	45.8254892	1.493576399	1.25E-06	2.87E-05	4833418N02Ri	17	87274886	87282814	-	2762	lincRNA	RIKEN cDNA 4833418N02 gene [Source:MGI Symbol;Acc:MGI:1921847]	-
ENSMUSG0000002940	253.5318765	174.8423214	280.4068174	465.9801173	554.0707433	583.8152603	236.2603384	534.6220403	-1.178192714	1.26E-06	2.88E-05	Cdkl2	5	92006074	92043883	-	6064	protein_coding	cyclin-dependent kinase-like 2 (CDC2-related kinase) [Source:MGI Symbol;Acc:MGI:1858227]	-
ENSMUSG0000005965	30.06306836	18.95880593	17.87453816	0	0	0	22.29880415	0	7.013716384	1.27E-06	2.91E-05	Stfa2l1	16	36156811	36161953	+	419	protein_coding	stefin A2 like 1 [Source:MGI Symbol;Acc:MGI:3524944]	-
ENSMUSG0000004671	508.0658553	723.5944263	408.8800605	222.2234201	195.55438	252.9866128	546.8467807	223.5881376	1.290464047	1.33E-06	3.04E-05	Hmga1	17	27556497	27563674	+	2125	protein_coding	high mobility group AT-hook 1 [Source:MGI Symbol;Acc:MGI:96160]	HMC
ENSMUSG0000003321	55.11562532	91.63422866	65.91235948	14.64262846	17.77767091	8.757228904	70.88740449	13.72584276	2.367714919	1.37E-06	3.11E-05	AA467197	2	122636986	122641191	+	1203	protein_coding	expressed sequence AA467197 [Source:MGI Symbol;Acc:MGI:3034182]	-
ENSMUSG0000005612	201.422558	298.0745599	196.6199198	453.060151	582.7125464	545.8672683	232.0390126	527.2133219	-1.18350833	1.37E-06	3.12E-05	B4galt6	18	20684599	20746404	-	5808	protein_coding	UDP-Gal:betaGlcNAc beta 1,4-galactosyltransferase, polypeptide 6 [Source:MGI Symbol;Acc:MGI:1928380]	-
ENSMUSG0000004235	5.010511393	8.426135969	13.40590362	68.90648685	45.43182565	59.35455146	8.947516995	57.89762132	-2.705704527	1.38E-06	3.13E-05	Grap2	15	80572594	80652854	+	6243	protein_coding	GRB2-related adaptor protein 2 [Source:MGI Symbol;Acc:MGI:1333842]	-
ENSMUSG0000010281	165.346876	85.31462668	199.9713957	40.48256102	43.45652888	32.10983931	150.2109661	38.68297641	1.956414197	1.38E-06	3.14E-05	Gm37795	4	150068539	150070997	+	2459	TEC	predicted gene, 37795 [Source:MGI Symbol;Acc:MGI:5611023]	-
ENSMUSG0000003644	1081.268359	649.8657366	227.9003616	3051.696036	2287.393657	2650.521282	653.0114856	2663.203658	-2.027576522	1.39E-06	3.16E-05	Lum	10	97565128	97572703	+	2448	protein_coding	lumican [Source:MGI Symbol;Acc:MGI:109347]	-
ENSMUSG0000002593	84.1765914	78.99502471	177.628223	8.613310856	25.67885798	23.35261041	113.5999464	19.21492641	2.570076548	1.40E-06	3.17E-05	Msc	1	14753346	14755992	-	2546	protein_coding	musculin [Source:MGI Symbol;Acc:MGI:1333884]	bHLI
ENSMUSG0000004965	1032.165347	794.1633151	636.7804221	340.2257788	405.9234857	392.1292498	821.0363614	379.4261714	1.114460326	1.41E-06	3.19E-05	Zbtb5	4	44991242	45012412	-	5606	protein_coding	zinc finger and BTB domain containing 5 [Source:MGI Symbol;Acc:MGI:1924601]	-
ENSMUSG0000001860	1459.060918	493.9822212	796.5341069	286.8232515	288.3933281	275.3661978	916.5257486	283.5275924	1.692896642	1.43E-06	3.24E-05	Tbx3	5	119670669	119684724	+	6624	protein_coding	T-box 3 [Source:MGI Symbol;Acc:MGI:98495]	T-box
ENSMUSG0000008754	141.2964213	132.7116415	103.8957531	28.42392582	45.43182565	43.78614452	125.9679386	39.21396533	1.688708096	1.44E-06	3.25E-05	Gm16576	15	79742698	79757394	+	5404	bidirectional_prom	predicted gene 16576 [Source:MGI Symbol;Acc:MGI:4414996]	-
ENSMUSG0000004074	158.33216	1185.978638	249.1263757	28.42392582	9.876483837	4.865127169	531.1457244	14.38851228	5.201755101	1.44E-06	3.25E-05	Slc25a34	4	141618824	141623821	-	2670	protein_coding	solute carrier family 25, member 34 [Source:MGI Symbol;Acc:MGI:2686215]	-
ENSMUSG0000003223	4678.815539	2093.894788	6613.579121	13287.75466	13053.74869	15344.61109	4462.096483	13895.37148	-1.638846209	1.45E-06	3.26E-05	Anxa2	9	69453620	69491795	+	3731	protein_coding	annexin A2 [Source:MGI Symbol;Acc:MGI:88246]	-
ENSMUSG0000004099	598.2550603	579.2968479	414.4658537	921.6242616	1165.425093	1227.958097	530.6725873	1105.002484	-1.05738419	1.47E-06	3.31E-05	Sh3kbp1	X	159627272	159978069	+	10100	protein_coding	SH3-domain kinase binding protein 1 [Source:MGI Symbol;Acc:MGI:1889583]	-
ENSMUSG0000004698	1631.42251	2144.451604	1765.110644	948.3255252	1078.512055	1063.516799	1846.994919	1030.11812	0.842533409	1.48E-06	3.31E-05	Tapt1	5	44175154	44226626	-	4443	protein_coding	transmembrane anterior posterior transformation 1 [Source:MGI Symbol;Acc:MGI:2683537]	-
ENSMUSG0000002209	162.3405691	163.2563844	285.9926106	710.5981456	854.3158519	386.2910972	203.863188	650.4016983	-1.674737018	1.48E-06	3.33E-05	Hr	14	70552212	70573548	+	6919	protein_coding	hairless [Source:MGI Symbol;Acc:MGI:96223]	-
ENSMUSG0000000587	5037.568155	3572.681651	4320.052442	7001.760395	8565.874432	8585.976428	4310.100749	8051.203752	-0.901409062	1.51E-06	3.37E-05	Reep5	18	34344889	34373415	-	2894	protein_coding	receptor accessory protein 5 [Source:MGI Symbol;Acc:MGI:1270152]	-
ENSMUSG0000009757	266.5592061	212.7599332	359.7250805	98.19174376	117.5301577	112.8709503	279.6814066	109.5309506	1.352734467	1.54E-06	3.45E-05	C920006O11R	9	78175914	78178879	+	1444	lincRNA	RIKEN cDNA C920006O11 gene [Source:MGI Symbol;Acc:MGI:2443759]	-
ENSMUSG0000004145	3022.340472	2207.647624	3211.831076	1621.886434	954.0683387	1060.597723	2813.939724	1212.184165	1.214611957	1.55E-06	3.46E-05	Rpl21	5	146832890	146837032	+	3400	protein_coding	ribosomal protein L21 [Source:MGI Symbol;Acc:MGI:1278340]	-
ENSMUSG0000002137	108.2270461	50.55681581	68.14667675	291.1299069	227.1591283	199.4702139	75.64351288	239.253083	-1.659882807	1.56E-06	3.47E-05	Id4	13	48261228	48266026	+	3849	protein_coding	inhibitor of DNA binding 4 [Source:MGI Symbol;Acc:MGI:99414]	bHLI
ENSMUSG0000002739	305.641195	309.6604969	482.6125304	987.0854241	749.6251233	773.5552199	365.9714074	836.7552557	-1.194192188	1.56E-06	3.47E-05	Slc20a1	2	129198764	129211616	+	5580	protein_coding	solute carrier family 20, member 1 [Source:MGI Symbol;Acc:MGI:108392]	-
ENSMUSG0000000455	6351.324242	6763.027382	5652.822694	3757.126195	3707.632033	4103.248254	6255.724773	3856.002161	0.698124934	1.58E-06	3.51E-05	Ndrg2	14	51905271	51914158	-	3397	protein_coding	N-myc downstream regulated gene 2 [Source:MGI Symbol;Acc:MGI:1352498]	-
ENSMUSG0000003576	1151.415518	984.8046414	628.9603116	1900.957706	1980.235009	2430.617534	921.7268237	2103.93675	-1.190243039	1.58E-06	3.53E-05	Fbxo45	16	32230112	32247158	-	4179	protein_coding	F-box protein 45 [Source:MGI Symbol;Acc:MGI:2447775]	-
ENSMUSG0000002010	445.935514	397.0816575	317.2730524	843.2431328	664.6873623	848.4781783	386.763408	785.4695578	-1.021590635	1.70E-06	3.77E-05	Slc29a3	10	60712072	60752794	-	5834	protein_coding	solute carrier family 29 (nucleoside transporters), member 3 [Source:MGI Symbol;Acc:MGI:1918529]	-
ENSMUSG0000007356	289.6075585	235.9318071	283.7582933	469.4254416	522.465995	577.9771077	269.7658863	523.2895148	-0.955489465	1.72E-06	3.82E-05	Prr16	18	51117898	51304641	+	2224	protein_coding	proline rich 16 [Source:MGI Symbol;Acc:MGI:1918623]	-
ENSMUSG0000006855	444.9334117	417.0937305	256.9464861	810.5125515	824.6864004	848.4781783	372.9912094	827.8923767	-1.149367545	1.72E-06	3.82E-05	Zfp467	6	48427697	48445825	-	4255	protein_coding	zinc finger protein 467 [Source:MGI Symbol;Acc:MGI:1916160]	zf-C2
ENSMUSG0000008339	219.460399	247.5177441	183.2140162	92.16242616	48.3947708	32.10983931	216.7307198	57.55567876	1.908262842	1.74E-06	3.86E-05	Gm11703	11	115724326	115724808	-	483	processed_pseudog	predicted gene 11703 [Source:MGI Symbol;Acc:MGI:3649808]	-
ENSMUSG0000002841	1645.491541	1410.324508	1348.410473	2342.820553	2426.652079	2592.139756	1468.062307	2453.870796	-0.740877034	1.77E-06	3.92E-05	Slc44a1	4	53440413	53622478	+	6945	protein_coding	solute carrier family 44, member 1 [Source:MGI Symbol;Acc:MGI:2140592]	-
ENSMUSG0000011498	24.05045469	37.91761186	45.80350404	3.445324342	1.975296767	0	35.92385686	1.806673703	4.279568338	1.78E-06	3.92E-05	Gm49303	14	47960019	47973713	+	442	lincRNA	predicted gene, 49303 [Source:MGI Symbol;Acc:MGI:6118800]	-
ENSMUSG0000000075	1367.86961	717.2748243	725.0359543	3404.841781	1988.136196	2347.910372	936.7267963	2580.296117	-1.461657898	1.79E-06	3.95E-05	Serpinf1	11	75409769	75422701	-	2359	protein_coding	serine (or cysteine) peptidase inhibitor, clade F, member 1 [Source:MGI Symbol;Acc:MGI:108080]	-
ENSMUSG0000009797	4508.458151	1272.346531	8804.327204	521.1053068	384.1952213	334.7207492	4861.710629	413.3404258	3.555954998	1.81E-06	3.98E-05	Gm26917	17	39843013	39846341	+	3329	lincRNA	predicted gene, 26917 [Source:MGI Symbol;Acc:MGI:5504032]	-
ENSMUSG0000004253	1.002102279	0	3.351475906	34.45324342	42.4688805	28.21773758	1.451192728	35.0466205	-4.625811356	1.82E-06	4.01E-05	Golga7b	19	42247574	42270348	+	2988	protein_coding	golgi autoantigen, golgin subfamily a, 7B [Source:MGI Symbol;Acc:MGI:1918396]	-
ENSMUSG0000003359	8.016818229	20.01207293	4.468634541	89.5784329	52.34536434	88.54531447	10.83250857	76.82303724	-2.823363404	1.82E-06	4.01E-05	Lgi3	14	70530685	70538324	+	3899	protein_coding	leucine-rich repeat LGI family, member 3 [Source:MGI Symbol;Acc:MGI:2182619]	-
ENSMUSG0000010304	397.8346046	347.5781087	450.21493	136.0903115	192.5914348	202.3892902	398.5425478	177.0236789	1.172206423	1.83E-06	4.02E-05	Gm37305	3	65957758	65962036	+	4279	antisense	predicted gene, 37305 [Source:MGI Symbol;Acc:MGI:5610533]	-
ENSMUSG0000009232	33.06937519	44.23721384	169.8081126	1.722662171	4.938241919	10.70327977	82.37156719	5.788061287	3.841538709	1.84E-06	4.04E-05	BB365896	6	58965655	59002083	+	3000	antisense	expressed sequence BB365896 [Source:MGI Symbol;Acc:MGI:3034340]	-
ENSMUSG0000005272	254.5339788	341.2585067	443.5119782	1487.518785	642.9590978	962.322154	346.4348212	1030.933346	-1.574176011	1.85E-06	4.04E-05	Map1b	13	99421446	99516540	-	15846	protein_coding	microtubule-associated protein 1B [Source:MGI Symbol;Acc:MGI:1306778]	-
ENSMUSG0000008999	80.16818229	47.39701482	41.3348695	8.613310856	7.90118707	10.70327977	56.30002221	9.072592566	2.636196578	1.87E-06	4.09E-05	Gm6485	3	104752727	104753165	-	439	transcribed_process	predicted gene 6485 [Source:MGI Symbol;Acc:MGI:3644007]	-
ENSMUSG0000001536	1060.224211	1199.671109	1205.414167	715.7661321	703.2056492	633.4395574	1155.103162	684.1371129	0.755317993	1.87E-06	4.09E-05	Trabd	15	89075124	89087077	+	5591	protein_coding	TraB domain containing [Source:MGI Symbol;Acc:MGI:1915226]	-
ENSMUSG0000003233	2187.589274	2071.776181	2122.601407	3147.303787	3257.26437	3483.431053	2127.322287	3295.999736	-0.631592857	1.92E-06	4.19E-05	Nptn	9	58582240	58657955	+	6168	protein_coding	neuroplastin [Source:MGI Symbol;Acc:MGI:108077]	-
ENSMUSG0000000146	368.7736385	689.8898824	399.9427914	1323.004547	969.8707128	1422.563184	486.2021041	1238.479481	-1.349033548	1.94E-06	4.22E-05	Cyp51	5	4081145	4104746	-	4415	protein_coding	cytochrome P450, family 51 [Source:MGI Symbol;Acc:MGI:106040]	-

ENSMUSG0000004144	3457.252861	4169.884038	2337.095865	1550.395954	1616.780404	1535.434135	3321.410921	1567.536831	1.083391956	1.97E-06	4.30E-05	Mmrn2	14	34375465	34404287	+	3974	protein_coding	multimerin 2 [Source:MGI Symbol;Acc:MGI:2385618]	-
ENSMUSG0000005267	380.7988659	413.9339295	378.7167773	216.1941025	212.3444025	204.3353411	391.1498576	210.9579487	0.890563481	1.98E-06	4.30E-05	Zmat1	X	134971372	135009209	-	5391	protein_coding	zinc finger, matrin type 1 [Source:MGI Symbol;Acc:MGI:2442284]	-
ENSMUSG0000005195	2.004204557	2.106533992	3.351475906	25.83993257	35.55534181	42.81311909	2.487404818	34.73613116	-3.811529204	1.98E-06	4.30E-05	Xkr4	1	3205901	3671498	-	6094	protein_coding	X-linked Kx blood group related 4 [Source:MGI Symbol;Acc:MGI:3528744]	-
ENSMUSG0000003910	4137.680308	3889.715017	3369.350444	1843.248523	2402.948518	2182.496048	3798.915256	2142.897696	0.826271908	2.00E-06	4.33E-05	6-Mar	15	31455891	31531053	-	9174	protein_coding	membrane-associated ring finger (C3HC4) 6 [Source:MGI Symbol;Acc:MGI:2442773]	-
ENSMUSG0000004926	1051.20529	3063.953692	832.2831832	219.6394268	480.9847629	410.6167331	1649.147388	370.4136409	2.154969954	2.01E-06	4.35E-05	Kcnk3	5	30588170	30625271	+	3976	protein_coding	potassium channel, subfamily K, member 3 [Source:MGI Symbol;Acc:MGI:1100509]	-
ENSMUSG0000000150	693.4547768	683.5702805	731.7389061	413.4389211	360.4916601	271.474096	702.9213211	348.4682257	1.011418802	2.01E-06	4.36E-05	Itga3	11	95044474	95076801	-	5710	protein_coding	integrin alpha 3 [Source:MGI Symbol;Acc:MGI:96602]	-
ENSMUSG0000003988	1982.158307	881.5844757	1246.749037	2823.443299	3937.754106	4222.930383	1370.16394	3661.375929	-1.417827009	2.02E-06	4.36E-05	Tmem120a	5	135735485	135744448	-	1730	protein_coding	transmembrane protein 120A [Source:MGI Symbol;Acc:MGI:2686991]	-
ENSMUSG0000002839	860.8058573	821.548257	542.9390967	1237.73277	2108.629299	2187.361175	741.7644037	1844.574415	-1.313774632	2.03E-06	4.38E-05	Ptprd	4	75941238	78211961	-	22822	protein_coding	protein tyrosine phosphatase, receptor type, D [Source:MGI Symbol;Acc:MGI:97812]	-
ENSMUSG0000002896	46.09670482	70.56888874	58.09224903	12.0586352	12.83942899	9.730254338	58.2526142	11.54277284	2.334009496	2.04E-06	4.40E-05	Tnfrsf9	4	150914562	150946102	+	2722	protein_coding	tumor necrosis factor receptor superfamily, member 9 [Source:MGI Symbol;Acc:MGI:1101059]	-
ENSMUSG0000003329	527.1057986	511.8877601	396.5913155	1894.067057	930.3647775	1000.270146	478.5282914	1274.90066	-1.41380596	2.05E-06	4.41E-05	Ptprf	4	118208213	118291405	-	9148	protein_coding	protein tyrosine phosphatase, receptor type, F [Source:MGI Symbol;Acc:MGI:102695]	-
ENSMUSG0000000404	4966.418893	6158.452126	8221.170397	2701.995615	3485.411146	3133.141897	6448.680472	3106.849553	1.053560648	2.05E-06	4.42E-05	Stat3	11	100885098	100939540	-	9527	protein_coding	signal transducer and activator of transcription 3 [Source:MGI Symbol;Acc:MGI:103038]	STAT
ENSMUSG0000000992	8421.66755	9259.270163	7957.520959	5707.179773	4404.911792	4859.289016	8546.15289	4990.460194	0.776024037	2.06E-06	4.43E-05	Rps25	9	44407139	44410427	+	2691	protein_coding	ribosomal protein S25 [Source:MGI Symbol;Acc:MGI:1922867]	-
ENSMUSG0000002847	687.4421631	682.5170135	929.4759845	408.2709346	401.9728922	378.5068937	766.478387	396.2502402	0.951287331	2.14E-06	4.59E-05	Spaar	4	43730034	43759462	+	5195	protein_coding	small regulatory polypeptide of amino acid response [Source:MGI Symbol;Acc:MGI:1918656]	-
ENSMUSG0000002725	183.384717	173.7890544	119.535974	399.6576237	313.0845376	385.3180718	158.9032484	366.0200777	-1.202666776	2.17E-06	4.65E-05	Map1a	2	121289600	121310832	+	12166	protein_coding	microtubule-associated protein 1 A [Source:MGI Symbol;Acc:MGI:1306776]	-
ENSMUSG0000003423	510.0700598	1086.97154	1145.087601	387.5989885	295.3068667	289.9615793	914.043067	324.2891448	1.494220129	2.18E-06	4.66E-05	Usp54	14	20548912	20641063	-	9068	protein_coding	ubiquitin specific peptidase 54 [Source:MGI Symbol;Acc:MGI:1926037]	-
ENSMUSG0000003239	17205.09402	18186.76122	15389.97736	11364.40234	10213.27194	10244.98479	16927.27753	10607.55302	0.674244436	2.18E-06	4.66E-05	Rpl4	9	64173375	64178666	+	2007	protein_coding	ribosomal protein L4 [Source:MGI Symbol;Acc:MGI:1915141]	-
ENSMUSG0000007867	1637.435123	1615.711572	1433.314529	988.8080863	906.6612163	973.9984592	1562.153741	956.4892539	0.707724311	2.19E-06	4.69E-05	Chd2	7	73426638	73541830	-	14300	protein_coding	chromodomain helicase DNA binding protein 2 [Source:MGI Symbol;Acc:MGI:2448567]	-
ENSMUSG0000002893	4617.6873	3797.027521	3977.084741	2677.017014	2247.887721	2430.617534	4130.599854	2451.840756	0.75241663	2.23E-06	4.75E-05	Rpl22	4	152325742	152334071	-	2911	protein_coding	ribosomal protein L22 [Source:MGI Symbol;Acc:MGI:99262]	-
ENSMUSG0000004694	256.5381833	270.689618	233.4861548	424.6362252	506.6636209	514.7304545	253.5713187	482.0101002	-0.925769557	2.24E-06	4.77E-05	Nqo2	13	33964687	33988443	+	4042	protein_coding	N-ribosyldihydronicotinamide quinone reductase 2 [Source:MGI Symbol;Acc:MGI:104513]	-
ENSMUSG0000004962	66.13875039	73.72868973	81.55258037	178.2955347	173.8261155	168.3334	73.8066735	173.4850168	-1.234642757	2.24E-06	4.78E-05	Tifab	13	56173704	56178885	-	3497	protein_coding	TRAF-interacting protein with forkhead-associated domain, family member B [Source:MGI Symbol;Acc:MGI:2385852]	-
ENSMUSG0000003537	68.14295495	56.87641779	121.7702912	251.508677	602.4655141	215.0386209	82.26322132	356.337604	-2.116051004	2.27E-06	4.83E-05	Ccl7	11	82045712	82047525	+	801	protein_coding	chemokine (C-C motif) ligand 7 [Source:MGI Symbol;Acc:MGI:99512]	-
ENSMUSG0000008185	139.2922167	143.2443115	189.916968	65.4611625	42.4688805	57.40850059	157.4844987	55.11284787	1.511194648	2.27E-06	4.83E-05	Rpl17-ps5	5	22612628	22613182	-	555	processed_pseudog	ribosomal protein L17, pseudogene 5 [Source:MGI Symbol;Acc:MGI:3704246]	-
ENSMUSG0000009220	229.4814218	267.529817	348.5534942	134.3676494	96.78954161	112.8709503	281.854911	114.6760471	1.294974354	2.28E-06	4.84E-05	1110038B12Ri	17	34950238	34952471	-	1811	processed_transcrip	RIKEN cDNA 1110038B12 gene [Source:MGI Symbol;Acc:MGI:1916013]	-
ENSMUSG0000000127	794.6671069	743.6064992	498.2527513	3776.075479	1527.89205	1443.969744	678.8421192	2249.312424	-1.72832822	2.30E-06	4.87E-05	Ckb	12	111669361	111672338	-	2895	protein_coding	creatine kinase, brain [Source:MGI Symbol;Acc:MGI:88407]	-
ENSMUSG0000003656	19.03994329	16.85227194	13.40590362	64.59983142	71.11068363	67.13875493	16.43270628	67.61642333	-2.036803723	2.38E-06	5.04E-05	Lgi4	7	31059342	31070935	+	4584	protein_coding	leucine-rich repeat LGI family, member 4 [Source:MGI Symbol;Acc:MGI:2180197]	-
ENSMUSG0000002603	5822.214239	6062.60483	8566.372415	3520.260147	3635.533701	3730.579513	6817.063828	3628.79112	0.909614932	2.41E-06	5.10E-05	Clk1	1	58410189	58424066	-	8132	protein_coding	CDC-like kinase 1 [Source:MGI Symbol;Acc:MGI:107403]	-
ENSMUSG0000007877	140.294319	124.2855055	107.247229	279.0712717	238.0232605	311.3681388	123.9423512	276.1542237	-1.154643077	2.42E-06	5.11E-05	Evi2a	11	79526560	79530609	-	2297	protein_coding	ecotropic viral integration site 2a [Source:MGI Symbol;Acc:MGI:95458]	-
ENSMUSG0000002795	460.9670482	303.3408949	575.3366971	1003.450715	959.0065806	1082.977308	446.5482134	1015.144868	-1.185182295	2.42E-06	5.11E-05	Pmvk	3	89454541	89469013	+	1713	protein_coding	phosphomevalonate kinase [Source:MGI Symbol;Acc:MGI:1915853]	-
ENSMUSG0000004225	12.02522734	8.426135969	24.57748997	54.26385839	93.82659646	110.9248995	15.00961776	86.33845143	-2.529620802	2.44E-06	5.15E-05	Pm20d1	1	131797381	131821473	+	7028	protein_coding	peptidase M20 domain containing 1 [Source:MGI Symbol;Acc:MGI:2442939]	-
ENSMUSG0000002110	2618.493254	2370.904008	2764.967622	1521.972028	1652.335746	1654.143237	2584.788295	1609.483671	0.68352822	2.46E-06	5.17E-05	Glrx5	12	105032688	105042906	+	3682	protein_coding	glutaredoxin 5 [Source:MGI Symbol;Acc:MGI:1920296]	-
ENSMUSG0000007952	1738.647453	1722.091539	1953.910453	1099.919796	587.6507883	684.0368799	1804.883148	790.5358215	1.190336828	2.46E-06	5.18E-05	Tmsb10	6	72957347	72958748	-	1276	protein_coding	thymosin, beta 10 [Source:MGI Symbol;Acc:MGI:109146]	-
ENSMUSG0000003120	1574.30268	1216.52338	1098.166938	2065.471943	3109.117112	3080.598523	1296.331	2751.729193	-1.085568086	2.47E-06	5.20E-05	Heph	X	96455359	96574485	+	7662	protein_coding	hephaestin [Source:MGI Symbol;Acc:MGI:1332240]	-
ENSMUSG0000010830	12.02522734	10.53266996	14.52306226	50.81853405	61.23419979	64.21967863	12.36031985	58.75747082	-2.250474098	2.49E-06	5.24E-05	Gm45218	6	91599232	91605514	+	1678	lincRNA	predicted gene 45218 [Source:MGI Symbol;Acc:MGI:5753794]	-
ENSMUSG0000004706	455.9565368	396.0283905	493.7841168	242.0340351	183.7025994	236.4451804	448.5896814	220.7272716	1.022123468	2.52E-06	5.28E-05	Dusp28	1	92906981	92908435	+	1314	protein_coding	dual specificity phosphatase 28 [Source:MGI Symbol;Acc:MGI:1914696]	-
ENSMUSG0000005371	390.8198887	382.3359196	292.6955624	694.232855	641.9714494	668.468473	355.2837902	668.2242591	-0.910700135	2.52E-06	5.28E-05	Dusp7	9	106368632	106375724	+	3356	protein_coding	dual specificity phosphatase 7 [Source:MGI Symbol;Acc:MGI:2387100]	-
ENSMUSG0000002053	237.49824	191.6945933	221.1974098	64.59983142	107.6536738	57.40850059	216.7967477	76.55400195	1.503816239	2.52E-06	5.28E-05	Shmt1	11	60788104	60811718	-	3883	protein_coding	serine hydroxymethyltransferase 1 (soluble) [Source:MGI Symbol;Acc:MGI:98299]	-
ENSMUSG0000002611	759.5935272	1257.600793	854.6263559	429.8042117	448.3923662	464.1331319	957.2735588	447.4432366	1.097298087	2.53E-06	5.29E-05	Mgat4a	1	37439340	37541016	-	12881	protein_coding	mannoside acetylglucosaminyltransferase 4, isoenzyme A [Source:MGI Symbol;Acc:MGI:2662992]	-
ENSMUSG0000010435	482.011196	456.0646093	549.6420485	122.3090142	175.8014123	264.662918	495.9059513	187.5911148	1.403834026	2.60E-06	5.42E-05	Gm38244	3	106478283	106480868	+	2586	TEC	predicted gene, 38244 [Source:MGI Symbol;Acc:MGI:5611472]	-
ENSMUSG0000003661	153.3216486	119.0191706	142.9963053	291.1299069	278.5168442	287.042503	138.4457082	285.5630847	-1.044297277	2.60E-06	5.42E-05	Eipr1	12	28751803	28869865	+	4998	protein_coding	EARP complex and GARP complex interacting protein 1 [Source:MGI Symbol;Acc:MGI:1289332]	-
ENSMUSG0000003982	75.1576709	32.65127688	44.68634541	155.9009265	161.9743349	185.8478579	50.83176439	167.9077064	-1.720692377	2.61E-06	5.44E-05	Myl6b	10	128494157	128498685	-	1847	protein_coding	myosin, light polypeptide 6B [Source:MGI Symbol;Acc:MGI:1917789]	-
ENSMUSG0000002654	48.10090937	18.95880593	58.09224903	161.068913	140.2460705	184.8748324	41.71732144	162.063272	-1.959317016	2.63E-06	5.47E-05	Slamf9	1	172475358	172478575	+	1313	protein_coding	SLAM family member 9 [Source:MGI Symbol;Acc:MGI:1923692]	-
ENSMUSG0000006181	328.6895474	95.84729665	303.8671488	608.9610775	1141.721532	1068.381926	242.8013309	939.6881785	-1.952196183	2.63E-06	5.47E-05	Myl1	1	66924295	66945404	-	2074	protein_coding	myosin, light polypeptide 1 [Source:MGI Symbol;Acc:MGI:97269]	-
ENSMUSG0000003866	232.4877286	167.4694524	159.7536848	782.0886257	471.108279	360.0194105	186.5702886	537.7387718	-1.527101778	2.64E-06	5.48E-05	Lpar1	4	58435255	58553898	-	4289	protein_coding	lysophosphatidic acid receptor 1 [Source:MGI Symbol;Acc:MGI:108429]	-
ENSMUSG0000008359	972.0392103	1063.799666	1019.965834	637.3850033	432.5899921	396.0213515	1018.60157	488.665449	1.058692424	2.71E-06	5.62E-05	Rpl21-ps15	X	151215167	151215649	-	483	processed_pseudog	ribosomal protein L21, pseudogene 15 [Source:MGI Symbol;Acc:MGI:3705426]	-
ENSMUSG0000003025	932.9572214	750.9793682	718.3330024	1285.967311	1544.682072	1561.705821	800.7565307	1464.118401	-0.870044094	2.72E-06	5.63E-05	Sspn	6	145931641	145965223	+	4996	protein_coding	sarcospan [Source:MGI Symbol;Acc:MGI:1353511]	-
ENSMUSG0000000234	1.002102279	0	0	28.42392582	21.72826444	49.62429712	0.334034093	33.25882913	-6.456876403	2.74E-06	5.68E-05	Ncan	8	70093085	70120873	-	7195	protein_coding	neurocan [Source:MGI Symbol;Acc:MGI:104694]	-
ENSMUSG0000002970	854.7932437	734.1270963	1185.305312	2313.535296	1642.459262	1987.890961	924.741884	1981.295173	-1.099735693	2.84E-06	5.87E-05	Rbm28	6	29123576	29165006	-	5663	protein_coding	RNA binding motif protein 28 [Source:MGI Symbol;Acc:MGI:2655711]	-
ENSMUSG0000006951	619.2992082	477.1299492	554.1106831	867.3604032	1975.296767	1443.969744	550.1799468	1428.875638	-1.376552225	2.88E-06	5.94E-05	Lyz1	10	117287797	117292868	-	1263	protein_coding	lysozyme 1 [Source:MGI Symbol;Acc:MGI:96902]	-
ENSMUSG0000000525	170.3573874	142.1910445	317.2730524	62.01583816	9.876483837	28.21773758	209.9404947	33.37001986	2.646612065	2.92E-06	6.01E-05	Ripk4	16	97741933	97763787	-	3591	protein_coding	receptor-interacting serine-threonine kinase 4 [Source:MGI Symbol;Acc:MGI:1919638]	-
ENSMUSG0000004779	94.19761419	127.4453065	212.2601407	4.306655428	37.53063858	14.59538151	144.6343538	18.81089184	2.950625167	2.94E-06	6.07E-05	Cd300lf	11	115116214	115133992	-	5149	protein_coding	CD300 molecule like family member F [Source:MGI Symbol;Acc:MGI:2442359]	-
ENSMUSG0000002869	670.4064244	443.4254054	587.6254421	272.180623	244.9367992	296.7727573	567.152424	271.2967265	1.063945438	2.96E-06	6.09E-05	Pik3r3	4	116221618	116303056	+	5300	protein_coding	phosphoinositide-3-kinase regulatory subunit 3 [Source:MGI Symbol;Acc:MGI:109277]	-
ENSMUSG0000004346	10.02102279	8.426135969	4.468634541	43.92788537	47.40712242	54.48942429	7.638597765	48.60814403	-2.660227539	2.99E-06	6.15E-05	Rab9b	X	136858147	136868755	-	3980	protein_coding	RAB9B, member RAS oncogene family [Source:MGI Symbol;Acc:MGI:2442454]	-
ENSMUSG0000000815	139.2922167	168.5227194	87.13837355	259.2606568	481.9724113	435.9153943	131.6511032	392.3828208	-1.57384638	3.00E-06	6.16E-05	Clstn3	6	124430759	124464794	-	4968	protein_coding	calsyntenin 3 [Source:MGI Symbol;Acc:MGI:2178323]	-
ENSMUSG0000005914	59.12403444	43.18394684	69.26383538	243.7566972	159.9990382	136.2235607	57.19060555	179.9930987	-1.656405405	3.04E-06	6.23E-05	Ntrk3	7	78175959	78738012	-	30170	protein_coding	neurotrophic tyrosine kinase, receptor, type 3 [Source:MGI Symbol;Acc:MGI:97385]	-
ENSMUSG0000006644	980.0560285	1533.556746	1185.305312	523.6893	618.2678882	663.6033458	1232.972696	601.8535114	1.034900434	3.05E-06	6.25E-05	Zfyve26	12	79232346	79296304	-	18957	protein_coding	zinc finger, FYVE domain containing 26 [Source:MGI Symbol;Acc:MGI:1924767]	-
ENSMUSG0000006352	6566.776232	7100.072821	6364.452745	10671.89215	13036.95867	10055.24483	6677.100599	11254.69855	-0.753207223	3.09E-06	6.33E-05	Eno1	4	150236721	150248879	+	5379	protein_coding	enolase 1, alpha non-neuron [Source:MGI Symbol;Acc:MGI:95393]	-
ENSMUSG0000003196	1206.531143	1303.944541	1422.142943	2042.216004	2091.839277	2157.197387	1310.872876	2097.084222	-0.678038913	3.14E-06	6.43E-05	Aars	8	111033144	111057664	+	9282	protein_coding	alanyl-tRNA synthetase [Source:MGI Symbol;Acc:MGI:2384560]	-
ENSMUSG0000003012	1905.998534	2630.007689	832.2831832	4879.4406	5877.495532	5479.106218	1789.429802	5412.014116	-1.596534439	3.18E-06	6.49E-05	Plxnd1	6	115954811	115995005	-	7301	protein_coding	plexin D1 [Source:MGI Symbol;Acc:MGI:2154244]	-
ENSMUSG0000008434	898.8857439	1218.629914	1073.589448	623.603706	561.9719304	572.1389551	1063.701702	585.9048638	0.859938343	3.19E-06	6.50E-05	Rpl3-ps1	X	13202571	13203780	+	1210	processed_pseudog	ribosomal protein L3, pseudogene 1 [Source:MGI Symbol;Acc:MGI:3644217]	-
ENSMUSG0000004262	1739.649556	1765.275485	2446.577411	1149.015668	988.6360321	1008.054349	1983.834151	1048.568683	0.919498241	3.19E-06	6.50E-05	Safb2	17	56560965	56584585	-	8165	protein_coding	scaffold attachment factor B2 [Source:MGI Symbol;Acc:MGI:2146808]	-
ENSMUSG0000006869	7.01471595	1.053266996	1.117158635	89.5784329	36.5429902	28.21773758	3.061713861	51.44638689	-4.049444856	3.22E-06	6.56E-05	Gpr88	3	116249654	116253503	-	3504	protein_coding	G-protein coupled receptor 88 [Source:MGI Symbol;Acc:MGI:1927653]	-
ENSMUSG0000002555	4801.072017	7263.329205	4994.816258	2511.641446	3190.104279	2891.831589	5686.405827	2864.525771	0.989299269	3.25E-06	6.62E-05	Dock9	14	121542046	121797837	-	13245	protein_coding	dedicator of cytokinesis 9 [Source:MGI Symbol;Acc:MGI:106321]	-
ENSMUSG0000006426	612.2844922	844.7201309	782.0110447	416.8842454	389.1334632	380.4529446	746.3385559	395.4902177	0.915629937	3.27E-06	6.63E-05	Gimap8	6	48647234	48660875	+	3700	protein_coding	GTPase, IMAP family member 8 [Source:MGI Symbol;Acc:MGI:2685303]	-
ENSMUSG0000002218	201.422558	562.4445759	661.3579121	2225.679525	1051.845529	2034.596182	475.0750153	1770.707079	-1.898634654	3.28E-06	6.65E-05	C6	15	4727175	4814967	+	3556	protein_coding	complement component 6 [Source:MGI Symbol;Acc:MGI:88233]	-
ENSMUSG0000002770	1733.636942	1110.143414	1266.857892	2339.375228	3198.005467	2920.049327	1370.212749	2819.143341	-1.040579629	3.33E-06	6.76E-05	Mccc1	3	35959312	36000678	-	10952	protein_coding	methylcrotonoyl-Coenzyme A carboxylase 1 (alpha) [Source:MGI Symbol;Acc:MGI:1919289]	-
ENSMUSG0000003111	1089.285177	1062.746399	588.7426008	1958.666889	2006.901516	2173.738819	913.5913922	2046.435741	-1.16309243	3.35E-06	6.78E-05	Gpc4	X	52053021	52165252	-	2651	protein_coding	glypican 4 [Source:MGI Symbol;Acc:MGI:104902]	-
ENSMUSG0000002786	834.7511981	631.9601977	558.5793176	1422.918953	1385.670682	1170.549597	675.0969045	1326.379744	-0.973900926	3.35E-06	6.78E-05	Ptgfrn	3	101040232	101110278	-	10464	protein_coding	prostaglandin F2 receptor negative regulator [Source:MGI Symbol;Acc:MGI:1277114]	-
ENSMUSG0000001912	140.294319	83.20809269	103.8957531	349.7004207	266.6650636	236.4451804	109.1327216	284.2702216	-1.380754886	3.36E-06	6.79E-05	Scrn1	6	54501173	54566489	-	8613	protein_coding	secernin 1 [Source:MGI Symbol;Acc:MGI:1917188]	-
ENSMUSG0000003001	485.0175029	361.2705797	340.7333837	736.4380782	754.5633652	793.988754	395.6738221	761.6633991	-0.943951118	3.45E-06	6.96E-05	Fbxl14	6	119479668	119482630	+	2963	protein_coding	F-box and leucine-rich repeat protein 14 [Source:MGI Symbol;Acc:MGI:2141676]	-
ENSMUSG0000002897	1021.142222	1126.995686	1341.707521	471.1481038	639.9961527	661.657295	1163.28181	590.9338505	0.977524235	3.49E-06	7.03E-05	Abcb1b	5	8798147	8866315	+	7990	protein_coding	ATP-binding cassette, sub-family B (MDR/TAP), member 1B [Source:MGI Symbol;Acc:MGI:97568]	-
ENSMUSG0000000601	268.5634107	317.0333658	373.1309842	90.43976399	166.9125769	107.0327977	319.5759202	121.4617129	1.397209211	3.49E-06	7.03E-05	Prg4	1	150449412	150466165	-	4280	protein_coding	proteoglycan 4 (megakaryocyte stimulating factor, articular superficial zone protein) [Source:MGI Symbol;Acc:MGI:1891344]	-
ENSMUSG0000004436	192.4036375	141.1377775	242.4234238	81.82645313	63.20949656	60.32757689	191.9882796	68.45450886	1.485127279	3.53E-06	7.10E-05	Cxxc4	3	134236484	134262161	+	13401	protein_coding	CXXC finger 4 [Source:MGI Symbol;Acc:MGI:2442112]	-
ENSMUSG0000007570	3.006306836	1.053266996	0	31.86925017	33.58004505	30.16378845	1.353191277	31.87102789	-4.51669442	3.54E-06	7.12E-05	Dio3	12	110279068	110281097	+	2030	protein_coding	deiodinase, iodothyronine type III [Source:MGI Symbol;Acc:MGI:1306782]	-
ENSMUSG0000000178	2302.831036	2124.439531	2162.819118	1494.409434	1359.004176	1346.6672	2196.696562	1400.026937	0.649742794	3.61E-06	7.25E-05	Rtcb	10	85938637	85957823	-	2615	protein_coding	RNA 2',3'-cyclic phosphate and 5'-OH ligase [Source:MGI Symbol;Acc:MGI:106379]	-
ENSMUSG0000003074	11180.45512	12301.10525	9804.184183	7349.738153	5680.953503	5514.135133	11095.24818	6181.60893	0.843829154	3.62E-06	7.25E-05	Rps3	7	99477896	99483738	-	4483	protein_coding	ribosomal protein S3 [Source:MGI Symbol;Acc:MGI:1350917]	-
ENSMUSG0000007110	255.536081	147.4573795	384.3025705	95.6077505	50.37006757	34.05589018	262.4320103	60.01123608	2.124755909	3.64E-06	7.30E-05	1700029J07Rik	8	45953606	45975252	-	2740	protein_coding	RIKEN cDNA 1700029J07 gene [Source:MGI Symbol;Acc:MGI:1916729]	-
ENSMUSG0000007441	308.6475018	232.7720061	292.6955624	552.9745569	700.2427041	488.4587678	278.0383568	580.5586763	-1.061868349	3.66E-06	7.33E-05	3110039I08Rik	9	41327077	41716994	+	23898	processed_transcrip	RIKEN cDNA 3110039I08 gene [Source:MGI Symbol;Acc:MGI:1920394]	-
ENSMUSG0000002231	5466.46793	4345.779626	5071.900204	2566.766635	2905.661545	3197.361575	4961.382587	2889.929918	0.779827494	3.71E-06	7.41E-05	Rad21	15	51962240	51991747	-	4232	protein_coding	RAD21 cohesin complex component [Source:MGI Symbol;Acc:MGI:108016]	-
ENSMUSG0000000630	4455.346731	3564.255515	7869.265426	1972.448186	2187.64117	2269.095312	5296.289224	2143.061556	1.305281816	3.71E-06	7.41E-05	Tmbim1	1	74288247	74305622	-	12659	protein_coding	transmembrane BAX inhibitor motif containing 1 [Source:MGI Symbol;Acc:MGI:1916910]	-
ENSMUSG0000002059	10249.50211	14871.07672	15062.64988	4693.393085	6292.307853	7709.280512	13394.40957	6231.660483	1.103976524	3.80E-06	7.58E-05	Lpin1	12	16535669	16610966	-	9665	protein_coding	lipin 1 [Source:MGI Symbol;Acc:MGI:1891340]	-
ENSMUSG0000004371	12228.65411	12590.75367	12837.26988	8907.024756	7720.447416	7592.51746	12552.22588	8073.329877	0.636646683	3.88E-06	7.72E-05	Rpl7	1	16101295	16104662	-	2687	protein_coding	ribosomal protein L7 [Source:MGI Symbol;Acc:MGI:98073]	-
ENSMUSG0000001125	245.5150583	270.689618	205.5571889	419.4682387	498.7624338	486.5127169	240.5872884	468.2477965	-0.959643056	3.90E-06	7.77E-05	Adam19	11	46055992	46147343	+	7115	protein_coding	a disintegrin and metallopeptidase domain 19 (meltrin beta) [Source:MGI Symbol;Acc:MGI:105377]	-
ENSMUSG0000003084	1274.674098	1095.397676	815.5258037	2034.464024	1965.420284	2068.652072	1061.865859	2022.84546	-0.929408614	3.93E-06	7.81E-05	Lamtor1	7	101905904	101926675	+	3215	protein_coding	late endosomal/lysosomal adaptor, MAPK and MTOR activator 1 [Source:MGI Symbol;Acc:MGI:1913758]	-
ENSMUSG0000002131	26.05465924	14.74573795	12.28874499	72.35181119	72.09833201	83.6801873	17.69638073	76.0434435	-2.096270202	3.93E-06	7.81E-05	Amph	13	18948205	19150921	+	4528	protein_coding	amphiphysin [Source:MGI Symbol;Acc:MGI:103574]	-
ENSMUSG0000003834	2177.568251	2316.134124	2607.448255	788.9792744	1390.608924	1194.875233	2367.05021	1124.821144	1.073738964	4.16E-06	8.26E-05	Txlng	X	162778919	162829454	-	7541	protein_coding	taxilin gamma [Source:MGI Symbol;Acc:MGI:3590652]	-
ENSMUSG0000003159	98.2060233	176.9488553	48.03782131	608.9610775	321.9733731	370.7226903	107.7309	433.8857136	-2.009318521	4.22E-06	8.36E-05	Slc7a2	8	40862396	40922308	+	9135	protein_coding	solute carrier family 7 (cationic amino acid transporter, y+ system), member 2 [Source:MGI Symbol;Acc:MGI:99828]	-
ENSMUSG0000003659	2578.409163	1215.470113	798.7684242	6010.368315	3828.125135	5193.03674	1530.882567	5010.510064	-1.710413098	4.23E-06	8.38E-05	H2-Aa	17	34282744	34287827	-	1874	protein_coding	histocompatibility 2, class II antigen A, alpha [Source:MGI Symbol;Acc:MGI:95895]	-
ENSMUSG0000011358	13.02732962	9.479402965	10.05442772	138.6743048	34.56769343	63.2466532	10.8537201	78.82955047	-2.86087671	4.28E-06	8.47E-05	Dio3os	12	110275385	110278172	-	1683	bidirectional_prom	deiodinase, iodothyronine type III, opposite strand [Source:MGI Symbol;Acc:MGI:2664395]	-
ENSMUSG0000005712	680.4274472	489.7691532	471.4409441	279.0712717	277.5291958	251.0405619	547.2125148	269.2136765	1.023705607	4.30E-06	8.50E-05	Gja5	3	96904693	97077416	+	5746	protein_coding	gap junction protein, alpha 5 [Source:MGI Symbol;Acc:MGI:95716]	-
ENSMUSG0000006822	48.10090937	69.51562174	70.38099402	144.7036224	163.9496317	179.0366798	62.66584171	162.5633113	-1.376797046	4.33E-06	8.54E-05	Il2rb	15	78479256	78495271	-	4287	protein_coding	interleukin 2 receptor, beta chain [Source:MGI Symbol;Acc:MGI:96550]	-
ENSMUSG0000005578	8655.15738	6896.79229	4239.617021	12265.35466	16673.48001	19023.62026	6597.188897	15987.48498	-1.276954916	4.33E-06	8.54E-05	Abcd2	15	91145871	91191799	-	5946	protein_coding	ATP-binding cassette, sub-family D (ALD), member 2 [Source:MGI Symbol;Acc:MGI:1349467]	-
ENSMUSG0000002041	655.3748902	769.9381741	1079.175242	434.9721982	373.3310891	370.7226903	834.8294353	393.0086592	1.086109049	4.33E-06	8.54E-05	Pttg1	11	43420250	43426251	-	2000	protein_coding	pituitary tumor-transforming gene 1 [Source:MGI Symbol;Acc:MGI:1353578]	-
ENSMUSG0000004252	5199.908724	6811.477664	6497.394622	3004.322827	3314.547976	3953.402337	6169.59367	3424.091047	0.849493167	4.36E-06	8.58E-05	Sun2	15	79724070	79742536	-	5420	protein_coding	Sad1 and UNC84 domain containing 2 [Source:MGI Symbol;Acc:MGI:2443011]	-
ENSMUSG0000010138	152.3195463	354.9509777	382.0682532	73.21314228	99.75248676	96.32951794	296.4462591	89.76504899	1.723966395	4.37E-06	8.59E-05	Ms4a4a	19	11375523	11392790	+	836	protein_coding	membrane-spanning 4-domains, subfamily A, member 4A [Source:MGI Symbol;Acc:MGI:3643932]	-
ENSMUSG0000002460	1956.103648	1311.31741	989.8025508	2596.051892	3848.865751	3888.209633	1419.074536	3444.375759	-1.278985622	4.38E-06	8.60E-05	Isoc1	18	58659482	58679570	+	2563	protein_coding	isochorismatase domain containing 1 [Source:MGI Symbol;Acc:MGI:1913557]	-
ENSMUSG0000004767	8497.827323	9206.606813	8859.067977	6111.144052	5070.586802	4556.678106	8854.500704	5246.13632	0.755060778	4.39E-06	8.61E-05	Rps8	4	117153827	117156243	-	1726	protein_coding	ribosomal protein S8 [Source:MGI Symbol;Acc:MGI:98166]	-
ENSMUSG0000007460	573.2025034	545.592304	498.2527513	155.0395954	234.0726669	298.7188082	539.0158529	229.2770235	1.234821419	4.42E-06	8.67E-05	Mgst2	3	51660360	51682677	+	3703	protein_coding	microsomal glutathione S-transferase 2 [Source:MGI Symbol;Acc:MGI:2448481]	-
ENSMUSG0000005145	1990.175125	1979.088686	2864.394741	1128.343722	1311.597054	1112.168071	2277.886184	1184.036282	0.943913109	4.43E-06	8.67E-05	Crebzf	7	90442729	90447994	+	4174	protein_coding	CREB/ATF bZIP transcription factor [Source:MGI Symbol;Acc:MGI:2675296]	TF_b
ENSMUSG0000006073	1523.195463	1670.481456	1504.812682	1048.239931	909.6241614	938.9695436	1566.1632	965.6112121	0.697431107	4.43E-06	8.67E-05	Nsa2	13	97129424	97137937	-	4275	protein_coding	NSA2 ribosome biogenesis homolog [Source:MGI Symbol;Acc:MGI:1913883]	-
ENSMUSG0000011184	681.4295495	459.2244103	1513.749951	264.4286433	273.5786023	245.2024093	884.8013035	261.069885	1.760607407	4.44E-06	8.68E-05	Gm48832	9	85332352	85348065	-	3422	lincRNA	predicted gene, 48832 [Source:MGI Symbol;Acc:MGI:6098557]	-
ENSMUSG0000000494	1049.201086	705.6888874	692.6383538	1521.972028	1863.6925	1552.948592	815.8427756	1646.204374	-1.012289654	4.49E-06	8.76E-05	Dtx2	5	135994800	136032872	+	7032	protein_coding	deltex 2, E3 ubiquitin ligase [Source:MGI Symbol;Acc:MGI:1921448]	-
ENSMUSG0000004602	679.4253449	1171.2329	786.4796792	419.4682387	365.429902	421.3200128	879.0459746	402.0727178	1.128252198	4.50E-06	8.77E-05	Stard5	7	83631959	83653127	+	5460	protein_coding	StAR-related lipid transfer (START) domain containing 5 [Source:MGI Symbol;Acc:MGI:2156765]	-
ENSMUSG0000000584	1634.428816	1531.450212	1780.750865	1050.823924	1042.956693	1015.838553	1648.876631	1036.539724	0.669558107	4.51E-06	8.77E-05	Rsl1d1	16	11192970	11203331	-	2258	protein_coding	ribosomal L1 domain containing 1 [Source:MGI Symbol;Acc:MGI:1913659]	-
ENSMUSG0000005505	1586.327907	1620.977907	920.5387154	2782.099406	2879.982687	3119.519541	1375.948176	2927.200545	-1.088826757	4.58E-06	8.91E-05	Nfic	10	81396186	81455635	-	6630	protein_coding	nuclear factor I/C [Source:MGI Symbol;Acc:MGI:109591]	CTF/
ENSMUSG0000004812	2262.746945	2668.978568	1422.142943	934.5442279	1053.820825	918.5360095	2117.956152	968.9670209	1.128339957	4.62E-06	8.97E-05	Entpd1	19	40612366	40741602	+	5580	protein_coding	ectonucleoside triphosphate diphosphohydrolase 1 [Source:MGI Symbol;Acc:MGI:102805]	-
ENSMUSG0000003278	2533.31456	3524.231369	2093.555282	8020.715069	4597.503226	6276.987073	2717.033737	6298.40179	-1.212966761	4.62E-06	8.97E-05	Alas1	9	106233455	106248654	-	6174	protein_coding	aminolevulinic acid synthase 1 [Source:MGI Symbol;Acc:MGI:87989]	-
ENSMUSG0000007959	16.03363646	36.86434486	11.17158635	0	0	0	21.35652256	0	6.950772701	4.64E-06	9.00E-05	Gm5483	16	36184212	36188111	+	1862	protein_coding	predicted gene 5483 [Source:MGI Symbol;Acc:MGI:3645124]	-
ENSMUSG0000003523	192.4036375	142.1910445	158.6365262	316.1085084	324.9363183	378.5068937	164.4104027	339.8505735	-1.04649884	4.66E-06	9.02E-05	Pdk3	X	93764607	93832201	-	2561	protein_coding	pyruvate dehydrogenase kinase, isoenzyme 3 [Source:MGI Symbol;Acc:MGI:2384308]	-
ENSMUSG0000004498	317.6664223	640.3863336	232.3689961	1319.559223	1154.560961	1041.137214	396.8072507	1171.752466	-1.561997311	4.76E-06	9.21E-05	Tst	15	78399556	78405907	-	3163	protein_coding	thiosulfate sulfurtransferase, mitochondrial [Source:MGI Symbol;Acc:MGI:98852]	-
ENSMUSG0000004555	23.04835241	131.6583745	46.92066268	0.861331086	8.888835454	0.973025434	67.20912987	3.574397324	4.252362171	4.81E-06	9.31E-05	Fpr1	17	17876471	17883940	-	1324	protein_coding	formyl peptide receptor 1 [Source:MGI Symbol;Acc:MGI:107443]	-
ENSMUSG0000003283	345.7252861	149.5639134	99.42711853	543.499915	818.7605101	803.7190083	198.2387727	721.9931445	-1.863189628	4.93E-06	9.51E-05	Ank2	3	126921612	127499350	-	35004	protein_coding	ankyrin 2, brain [Source:MGI Symbol;Acc:MGI:88025]	-
ENSMUSG0000004428	71.14926178	87.42116068	125.1217671	202.4128051	251.8503379	299.6918336	94.5640632	251.3183255	-1.411845865	4.95E-06	9.54E-05	Cnr1	4	33924593	33948831	+	6444	protein_coding	cannabinoid receptor 1 (brain) [Source:MGI Symbol;Acc:MGI:104615]	-
ENSMUSG0000004887	1458.058815	1189.138439	1049.011958	2465.129567	2073.073957	2132.871751	1232.069737	2223.691758	-0.851696029	4.98E-06	9.60E-05	Hexim1	11	103116231	103119725	+	3495	protein_coding	hexamethylene bis-acetamide inducible 1 [Source:MGI Symbol;Acc:MGI:2385923]	-
ENSMUSG0000003320	4.008409114	1.053266996	4.468634541	52.54119622	25.67885798	35.02891562	3.176770217	37.7496566	-3.575864904	5.12E-06	9.85E-05	S100b	10	76253853	76261159	+	1484	protein_coding	S100 protein, beta polypeptide, neural [Source:MGI Symbol;Acc:MGI:98217]	-
ENSMUSG0000006434	34144.63094	28859.51569	31563.08292	44442.96135	80307.66538	74781.86971	31522.40985	66510.83215	-1.077203066	5.19E-06	9.98E-05	mt-Nd2	MT	3914	4951	+	1038	protein_coding	mitochondrially encoded NADH dehydrogenase 2 [Source:MGI Symbol;Acc:MGI:102500]	-
ENSMUSG0000005311	1864.912341	2300.335119	1594.185372	820.8485246	1158.511554	709.3355412	1919.810944	896.2318733	1.099250154	5.20E-06	9.99E-05	Socs3	11	117966079	117970047	-	2891	protein_coding	suppressor of cytokine signaling 3 [Source:MGI Symbol;Acc:MGI:1201791]	-
ENSMUSG0000002864	249.5234674	215.9197342	258.0636447	411.7162589	463.207092	455.375903	241.1689488	443.4330846	-0.878495254	5.23E-06	0.0001004	Mycbp	4	123904832	123912269	+	2934	protein_coding	MYC binding protein [Source:MGI Symbol;Acc:MGI:1891750]	-
ENSMUSG0000004173	1058.220006	888.9573447	959.6392677	579.6758206	576.7866561	598.4106418	968.9388729	584.9577062	0.72825478	5.24E-06	0.0001005	Coq5	5	115279666	115296972	+	3034	protein_coding	coenzyme Q5 methyltransferase [Source:MGI Symbol;Acc:MGI:1098643]	-
ENSMUSG0000010924	34.07147747	22.11860692	29.04612452	85.27177747	89.87600292	102.1676705	28.41206964	92.43848365	-1.700160417	5.25E-06	0.0001005	Gm44751	7	88311478	88315864	+	4387	lincRNA	predicted gene 44751 [Source:MGI Symbol;Acc:MGI:5753327]	-
ENSMUSG0000003174	146.3069327	94.79402965	73.73246992	408.2709346	231.1097218	290.9346047	104.9444774	310.105087	-1.561849633	5.35E-06	0.0001023	Gnao1	8	93809966	93969388	+	11053	protein_coding	guanine nucleotide binding protein, alpha O [Source:MGI Symbol;Acc:MGI:95775]	-
ENSMUSG0000003567	1792.760976	2219.233561	3466.543245	957.8001672	1275.054063	999.2971205	2492.845927	1077.383784	1.210243041	5.35E-06	0.0001023	Sbno2	10	80056992	80105571	-	10715	protein_coding	strawberry notch 2 [Source:MGI Symbol;Acc:MGI:2448490]	-
ENSMUSG0000003257	161.3384669	210.6533992	161.9880021	312.6631841	422.7135082	399.9134533	177.9932894	378.4300485	-1.087465809	5.36E-06	0.0001023	Mapkapk3	9	107254927	107289877	+	5542	protein_coding	mitogen-activated protein kinase-activated protein kinase 3 [Source:MGI Symbol;Acc:MGI:2143163]	-
ENSMUSG0000001711	3602.557692	3835.9984	3955.858727	2473.742878	2517.51573	2580.46345	3798.138273	2523.907353	0.589612113	5.36E-06	0.0001023	Nbr1	11	101552149	101581951	+	6871	protein_coding	NBR1, autophagy cargo receptor [Source:MGI Symbol;Acc:MGI:108498]	-
ENSMUSG0000003998	14.0294319	10.53266996	5.585793176	46.51187862	58.27125464	61.30060233	10.04929835	55.3612452	-2.451718954	5.41E-06	0.000103	Zc3h12d	10	7832470	7870396	+	4092	protein_coding	zinc finger CCCH type containing 12D [Source:MGI Symbol;Acc:MGI:3045313]	-
ENSMUSG0000007100	803.6860275	878.4246747	1052.363434	477.1774214	501.7253789	548.7863447	911.4913789	509.229715	0.839824986	5.47E-06	0.0001042	Hrct1	4	43727188	43728639	+	1452	protein_coding	histidine rich carboxyl terminus 1 [Source:MGI Symbol;Acc:MGI:1917945]	-
ENSMUSG0000002759	789.6565956	2262.417508	1226.640181	572.7851719	437.528234	411.5897585	1426.238095	473.9677215	1.58902027	5.49E-06	0.0001044	Ahcy	2	155059310	155074497	-	2805	protein_coding	S-adenosylhomocysteine hydrolase [Source:MGI Symbol;Acc:MGI:87968]	-
ENSMUSG0000005464	561.177276	589.8295178	637.8975807	311.801853	329.8745602	372.6687411	596.3014582	338.1150514	0.818763583	5.60E-06	0.0001064	Zfp869	8	69706332	69710724	-	1590	protein_coding	zinc finger protein 869 [Source:MGI Symbol;Acc:MGI:1914119]	zf-C2
ENSMUSG0000005744	649.3622765	490.8224202	541.8219381	931.0989035	1030.117264	1310.665259	560.6688783	1090.627142	-0.959451544	5.61E-06	0.0001065	Mpp7	18	7347962	7626863	-	4975	protein_coding	membrane protein, palmitoylated 7 (MAGUK p55 subfamily member 7) [Source:MGI Symbol;Acc:MGI:1922989]	-
ENSMUSG0000002585	149.3132395	168.5227194	168.6909539	400.5189548	295.3068667	310.3951134	162.1756376	335.4069783	-1.049759186	5.72E-06	0.0001085	Pdgfa	5	138976014	138997370	-	3119	protein_coding	platelet derived growth factor, alpha [Source:MGI Symbol;Acc:MGI:97527]	-
ENSMUSG0000000205	536.1247191	337.0454388	357.4907633	721.7954497	1016.290187	980.8096372	410.220307	906.2984246	-1.142695356	5.78E-06	0.0001094	Unc119	11	78343482	78349164	+	2448	protein_coding	unc-119 lipid binding chaperone [Source:MGI Symbol;Acc:MGI:1328357]	-
ENSMUSG0000003383	6.012613672	25.27840791	37.9833936	0	0	0	23.09147173	0	7.06074169	5.85E-06	0.0001107	Fgb	3	83040141	83049863	-	3810	protein_coding	fibrinogen beta chain [Source:MGI Symbol;Acc:MGI:99501]	-
ENSMUSG0000003667	288.6054562	303.3408949	313.9215765	172.2662171	140.2460705	150.8189422	301.9559759	154.4437433	0.965705707	5.92E-06	0.0001119	Aaas	15	102338252	102350771	-	2798	protein_coding	achalasia, adrenocortical insufficiency, alacrimia [Source:MGI Symbol;Acc:MGI:2443767]	-
ENSMUSG0000001359	6175.956343	6680.872556	6412.490566	3737.31558	4384.171175	4278.392832	6423.106489	4133.293196	0.636064207	5.95E-06	0.0001123	Ndufs2	1	171234853	171251388	-	2313	protein_coding	NADH:ubiquinone oxidoreductase core subunit S2 [Source:MGI Symbol;Acc:MGI:2385112]	-
ENSMUSG0000004721	7941.660558	7982.710563	8538.443449	5764.027625	4810.835277	4689.982591	8154.271523	5088.281831	0.680270781	5.96E-06	0.0001123	Rpl9	5	65388364	65391444	-	3081	protein_coding	ribosomal protein L9 [Source:MGI Symbol;Acc:MGI:1298373]	-
ENSMUSG0000000747	355.7463089	557.1782409	498.2527513	248.0633527	195.55438	205.3083665	470.3924337	216.3086997	1.119506072	5.97E-06	0.0001125	Lrrc8a	2	30237715	30263790	+	4308	protein_coding	leucine rich repeat containing 8A [Source:MGI Symbol;Acc:MGI:2652847]	-
ENSMUSG0000004241	236.4961378	231.7187391	244.6577411	126.6156696	110.616619	116.7630521	237.624206	117.9984469	1.00879607	5.98E-06	0.0001125	Nfkbil1	17	35220175	35235792	-	1746	protein_coding	nuclear factor of kappa light polypeptide gene enhancer in B cells inhibitor like 1 [Source:MGI Symbol;Acc:MGI:1340031]	-
ENSMUSG0000008626	0	1.053266996	0	28.42392582	17.77767091	43.78614452	0.351088999	29.99591375	-6.308334487	6.06E-06	0.0001139	Gm11670	11	108939310	108942015	-	511	antisense	predicted gene 11670 [Source:MGI Symbol;Acc:MGI:3649868]	-
ENSMUSG0000003768	881.8500052	1010.083049	1129.44738	505.6013472	622.2184818	566.3008025	1007.126812	564.7068772	0.834845368	6.17E-06	0.000116	Atp8a1	5	67618140	67847434	-	14278	protein_coding	ATPase, aminophospholipid transporter (APLT), class I, type 8A, member 1 [Source:MGI Symbol;Acc:MGI:1330848]	-
ENSMUSG0000006787	404.8493206	476.0766822	340.7333837	205.8581295	168.8878736	216.9846717	407.2197955	197.2435583	1.045717732	6.22E-06	0.0001167	Rpl31-ps8	15	90764279	90764656	-	378	processed_pseudog	ribosomal protein L31, pseudogene 8 [Source:MGI Symbol;Acc:MGI:3647726]	-
ENSMUSG0000002766	490.0280142	768.8849072	482.6125304	1535.753326	966.9077677	1661.927441	580.5084839	1388.196178	-1.25788424	6.25E-06	0.0001171	Gnb4	3	32580332	32616585	-	12692	protein_coding	guanine nucleotide binding protein (G protein), beta 4 [Source:MGI Symbol;Acc:MGI:104581]	-
ENSMUSG0000005772	347.7294907	608.7883237	367.545191	186.9088456	201.4802703	153.7380185	441.3543351	180.7090448	1.288167646	6.45E-06	0.0001207	Lepr	4	101717404	101815352	+	7049	protein_coding	leptin receptor [Source:MGI Symbol;Acc:MGI:104993]	-
ENSMUSG0000002386	331.6958542	320.1931668	369.7795083	521.9666379	775.3039812	747.2835331	340.5561764	681.5180508	-1.000547988	6.49E-06	0.0001214	Mpc1	17	8282904	8297661	+	2082	protein_coding	mitochondrial pyruvate carrier 1 [Source:MGI Symbol;Acc:MGI:1915240]	-
ENSMUSG0000005099	10.02102279	7.372868973	0	139.5356359	45.43182565	41.84009365	5.79796392	75.60251839	-3.691900626	6.53E-06	0.000122	Adgb	10	10335703	10472326	-	6192	protein_coding	androglobin [Source:MGI Symbol;Acc:MGI:3605549]	-
ENSMUSG0000006342	381.8009682	446.5852063	477.0267372	224.8074133	221.233238	139.142637	435.1376372	195.0610961	1.156479625	6.54E-06	0.0001221	Ddo	10	40630011	40681847	+	8925	protein_coding	D-aspartate oxidase [Source:MGI Symbol;Acc:MGI:1925528]	-
ENSMUSG0000002017	811.7028457	654.0788046	1300.372651	355.7297383	414.8123212	402.8325296	922.0514339	391.124863	1.237149971	6.60E-06	0.0001231	Adora2a	10	75316877	75334784	+	3062	protein_coding	adenosine A2a receptor [Source:MGI Symbol;Acc:MGI:99402]	-
ENSMUSG0000002769	537.1268213	712.0084894	578.688173	329.0284747	323.9486699	344.4510036	609.2744946	332.4760494	0.873797513	6.73E-06	0.0001253	Pld1	3	27938695	28133362	+	12961	protein_coding	phospholipase D1 [Source:MGI Symbol;Acc:MGI:109585]	-
ENSMUSG0000005029	288.6054562	403.4012595	484.8468477	173.1275482	190.6161381	135.2505353	392.2845211	166.3314072	1.23704903	6.74E-06	0.0001255	Foxc1	13	31806633	31812476	+	5844	protein_coding	forkhead box C1 [Source:MGI Symbol;Acc:MGI:1347466]	Fork
ENSMUSG0000004121	6.012613672	5.26633498	6.702951811	48.23454079	28.64180313	50.59732256	5.993966821	42.49122216	-2.828266182	6.75E-06	0.0001255	Clvs1	4	9269293	9451691	+	7566	protein_coding	clavesin 1 [Source:MGI Symbol;Acc:MGI:1921688]	-
ENSMUSG0000003014	20.04204557	16.85227194	31.28044179	99.05307484	66.17244171	96.32951794	22.72491977	87.1850115	-1.944844673	6.80E-06	0.0001263	Klrk1	6	129610323	129623864	-	7183	protein_coding	killer cell lectin-like receptor subfamily K, member 1 [Source:MGI Symbol;Acc:MGI:1196250]	-
ENSMUSG0000003786	32.06727292	29.49147589	2.23431727	99.91440593	193.5790832	172.2255018	21.26435536	155.2396636	-2.861544048	6.88E-06	0.0001278	Egr2	10	67535475	67542188	+	3398	protein_coding	early growth response 2 [Source:MGI Symbol;Acc:MGI:95296]	zf-C2
ENSMUSG0000003188	66.13875039	50.55681581	63.67804221	172.2662171	129.3819383	155.6840694	60.12453614	152.4440749	-1.342989573	7.00E-06	0.0001298	Car7	8	104534689	104550343	+	3637	protein_coding	carbonic anhydrase 7 [Source:MGI Symbol;Acc:MGI:103100]	-
ENSMUSG0000004512	9125.143349	9211.873148	8367.518178	6252.40235	4886.884203	5151.196646	8901.511558	5430.161067	0.712980847	7.01E-06	0.0001298	Rpl18a	8	70894722	70897412	-	1739	protein_coding	ribosomal protein L18A [Source:MGI Symbol;Acc:MGI:1924058]	-
ENSMUSG0000003272	91.19130735	81.1015587	26.81180725	215.3327714	262.7144701	243.2563584	66.36822443	240.4345333	-1.853650602	7.01E-06	0.0001298	Folr2	7	101839988	101857194	-	1546	protein_coding	folate receptor 2 (fetal) [Source:MGI Symbol;Acc:MGI:95569]	-
ENSMUSG0000003273	2906.096608	4166.724237	3169.379048	1813.963266	1998.01268	1922.698257	3414.066631	1911.558068	0.836788587	7.08E-06	0.0001308	Ablim3	18	61799395	61911852	-	4560	protein_coding	actin binding LIM protein family, member 3 [Source:MGI Symbol;Acc:MGI:2442582]	-
ENSMUSG0000002596	4155.718149	5400.099889	4612.748005	3096.485253	2630.107646	2577.544374	4722.855348	2768.045758	0.770654891	7.18E-06	0.0001326	Eef1b2	1	63176825	63180486	+	2532	protein_coding	eukaryotic translation elongation factor 1 beta 2 [Source:MGI Symbol;Acc:MGI:1929520]	-
ENSMUSG0000006181	200.4204557	223.2926032	280.4068174	119.7250209	88.88835454	93.41044164	234.7066254	100.6746057	1.218379584	7.20E-06	0.0001328	Rufy4	1	74125541	74148223	+	2329	protein_coding	RUN and FYVE domain containing 4 [Source:MGI Symbol;Acc:MGI:3588214]	-
ENSMUSG0000011400	1064.23262	867.8920048	726.1531129	493.542712	454.3182565	470.9443099	886.0925792	472.9350928	0.906036788	7.32E-06	0.0001349	Gm9616	13	3298887	3299667	+	781	processed_pseudog	predicted gene 9616 [Source:MGI Symbol;Acc:MGI:3780024]	-
ENSMUSG0000002125	351.7378998	312.8202978	388.7712051	768.3073283	548.144853	725.8769736	351.1098009	680.776385	-0.955913763	7.41E-06	0.0001364	Tgfb3	12	86056745	86079041	-	3383	protein_coding	transforming growth factor, beta 3 [Source:MGI Symbol;Acc:MGI:98727]	-
ENSMUSG0000003328	1113.335632	795.2165821	1063.535021	1761.42207	1724.434078	1764.095111	990.6957448	1749.983753	-0.820805546	7.44E-06	0.0001369	Kctd17	15	78428564	78439303	+	2541	protein_coding	potassium channel tetramerisation domain containing 17 [Source:MGI Symbol;Acc:MGI:1920094]	-
ENSMUSG0000005308	974.0434148	840.5070629	760.7850306	1391.911034	1537.768533	1410.886879	858.4451694	1446.855482	-0.752653525	7.53E-06	0.0001384	2700081O15Ri	19	7417625	7425904	+	7032	protein_coding	RIKEN cDNA 2700081O15 gene [Source:MGI Symbol;Acc:MGI:1919667]	zf-BE
ENSMUSG0000007895	31.06517064	88.47442767	43.56918677	6.890648685	7.90118707	8.757228904	54.36959503	7.84968822	2.794325413	7.57E-06	0.0001389	Lncenc1	13	97434970	97497007	+	3301	lincRNA	long non-coding RNA, embryonic stem cells expressed 1 [Source:MGI Symbol;Acc:MGI:3780541]	-
ENSMUSG0000002951	1494.134497	1889.560991	1797.508244	954.3548428	1074.561442	1056.705621	1727.067911	1028.540635	0.747794361	7.76E-06	0.0001424	Ulk1	5	110784488	110810097	-	6488	protein_coding	unc-51 like kinase 1 [Source:MGI Symbol;Acc:MGI:1270126]	-
ENSMUSG0000002171	723.5178452	701.4758194	783.1282033	1089.583823	1264.189931	1367.100734	736.0406226	1240.291496	-0.752709725	7.77E-06	0.0001424	Nln	13	104023057	104109614	-	8484	protein_coding	neurolysin (metallopeptidase M3 family) [Source:MGI Symbol;Acc:MGI:1923055]	-
ENSMUSG0000005225	727.5262543	756.2457032	959.6392677	488.3747255	371.3557923	428.1311909	814.4704084	429.2872362	0.922966214	7.86E-06	0.0001438	Zfp622	15	25984366	25998481	+	7130	protein_coding	zinc finger protein 622 [Source:MGI Symbol;Acc:MGI:1289282]	-
ENSMUSG0000002445	2030.259216	2147.611405	2271.183505	1270.463351	1481.472576	1210.44364	2149.684709	1320.793189	0.702761414	7.91E-06	0.0001446	Arap3	18	37972624	37997574	-	5224	protein_coding	ArfGAP with RhoGAP domain, ankyrin repeat and PH domain 3 [Source:MGI Symbol;Acc:MGI:2147274]	-
ENSMUSG0000004510	46.09670482	51.61008281	29.04612452	167.0982306	93.82659646	163.4682729	42.25097071	141.4643666	-1.741897448	8.01E-06	0.0001464	Poln	5	34007179	34169448	-	5423	protein_coding	DNA polymerase N [Source:MGI Symbol;Acc:MGI:2675617]	-
ENSMUSG0000002749	934.961426	979.5383064	850.1577214	555.5585502	477.0341693	585.7613111	921.5524846	539.4513436	0.772507774	8.03E-06	0.0001466	Fam210b	2	172345565	172355749	+	3617	protein_coding	family with sequence similarity 210, member B [Source:MGI Symbol;Acc:MGI:1914267]	-
ENSMUSG0000002009	24.05045469	7.372868973	18.9916968	200.6901429	42.4688805	109.951874	16.80500682	117.7036325	-2.808303663	8.10E-06	0.0001477	Pcbd1	10	61089331	61094324	+	817	protein_coding	pterin 4 alpha carbinolamine dehydratase/dimerization cofactor of hepatocyte nuclear factor 1 alpha (TCF1) 1 [Source:MGI Symbo	-
ENSMUSG0000002734	32.06727292	22.11860692	35.74907633	80.96512205	100.7401351	108.9788486	29.97831872	96.89470192	-1.692627412	8.38E-06	0.0001526	Rasgrp1	2	117279993	117343001	-	5600	protein_coding	RAS guanyl releasing protein 1 [Source:MGI Symbol;Acc:MGI:1314635]	-
ENSMUSG0000002237	547.1478441	625.6405957	615.554408	908.7042953	1048.882584	1231.850199	596.1142826	1063.145693	-0.834583604	8.44E-06	0.0001536	Fam49b	15	63929097	64060478	-	11412	protein_coding	family with sequence similarity 49, member B [Source:MGI Symbol;Acc:MGI:1923520]	-
ENSMUSG0000000020	248.5213651	319.1398998	851.27488	7.75197977	56.29595787	15.56840694	472.978715	26.53878153	4.158162225	8.45E-06	0.0001537	Slfn4	11	83175186	83190216	+	3964	protein_coding	schlafen 4 [Source:MGI Symbol;Acc:MGI:1329010]	-
ENSMUSG0000005904	172.3615919	160.0965834	98.3099599	281.655265	363.4546052	449.5377504	143.5893784	364.8825402	-1.343459598	8.48E-06	0.0001541	Eno1b	18	48045335	48048378	+	1757	protein_coding	enolase 1B, retrotransposed [Source:MGI Symbol;Acc:MGI:3648653]	-
ENSMUSG0000007165	656.3769925	642.4928676	606.6171389	383.2923331	341.7263408	398.9404278	635.162333	374.6530339	0.761507225	8.52E-06	0.0001545	Uqcc3	19	8880016	8880933	-	829	protein_coding	ubiquinol-cytochrome c reductase complex assembly factor 3 [Source:MGI Symbol;Acc:MGI:2147553]	-
ENSMUSG0000001093	557.1688669	693.0496834	526.1817172	932.8215657	1284.930547	1139.412783	592.1334225	1119.054965	-0.917885014	8.58E-06	0.0001556	Vac14	8	110618585	110720398	+	6960	protein_coding	Vac14 homolog (S. cerevisiae) [Source:MGI Symbol;Acc:MGI:2157980]	-
ENSMUSG0000000152	2674.610982	2681.617772	3691.092131	5139.562588	5395.52312	5269.905749	3015.773628	5268.330486	-0.804941237	8.60E-06	0.0001558	Tubb5	17	35833921	35838306	-	3327	protein_coding	tubulin, beta 5 class I [Source:MGI Symbol;Acc:MGI:107812]	-
ENSMUSG0000005389	1189.495405	880.5312087	2552.707481	207.5807916	612.3419979	396.0213515	1540.911365	405.3147137	1.927084661	8.61E-06	0.0001558	Slc39a8	3	135825279	135888572	+	7664	protein_coding	solute carrier family 39 (metal ion transporter), member 8 [Source:MGI Symbol;Acc:MGI:1914797]	-
ENSMUSG0000003965	1119.348245	1088.024807	916.0700809	660.6409426	606.4161076	558.516599	1041.147711	608.5245498	0.774688742	8.62E-06	0.0001559	Rxrb	17	34031812	34038393	+	3698	protein_coding	retinoid X receptor beta [Source:MGI Symbol;Acc:MGI:98215]	RXR
ENSMUSG0000003723	3432.200304	3899.19442	4234.031227	2403.97506	2514.552785	2391.696516	3855.141984	2436.741454	0.661761591	8.63E-06	0.0001559	Matr3	18	35562146	35593835	+	9869	protein_coding	matrin 3 [Source:MGI Symbol;Acc:MGI:1298379]	-
ENSMUSG0000004280	171.3594896	109.5397676	191.0341266	342.8097721	381.2322761	342.5049527	157.311128	355.515667	-1.176675633	8.76E-06	0.0001581	Gpr153	4	152274232	152285337	+	3909	protein_coding	G protein-coupled receptor 153 [Source:MGI Symbol;Acc:MGI:1916157]	-
ENSMUSG0000003624	0	0	0	13.78129737	17.77767091	25.29866128	0	18.95254319	-6.6065292	8.84E-06	0.0001593	Tbc1d21	9	58359704	58370455	-	1332	protein_coding	TBC1 domain family, member 21 [Source:MGI Symbol;Acc:MGI:1921536]	-
ENSMUSG0000007905	17.03573874	28.43820889	43.56918677	2.583993257	0.987648384	0.973025434	29.6810448	1.514889025	4.262058769	8.87E-06	0.0001598	Gm14025	2	129025073	129048172	-	4242	protein_coding	predicted gene 14025 [Source:MGI Symbol;Acc:MGI:3649276]	-
ENSMUSG0000002091	3806.986556	4642.800919	2117.015614	8426.40201	40846.17421	24195.25044	3522.267696	24489.27555	-2.797555029	8.89E-06	0.0001599	Acly	11	100476353	100528000	-	5279	protein_coding	ATP citrate lyase [Source:MGI Symbol;Acc:MGI:103251]	-
ENSMUSG0000009260	12.02522734	25.27840791	72.61531129	0.861331086	1.975296767	0	36.63964885	0.945542618	5.282350538	8.91E-06	0.0001602	4931413I07Rik	17	33783340	33785724	-	764	lincRNA	RIKEN cDNA 4931413I07 gene [Source:MGI Symbol;Acc:MGI:2148502]	-
ENSMUSG0000003371	36.07568203	28.43820889	26.81180725	84.41044639	104.6907287	90.49136534	30.44189939	93.19751347	-1.610940075	8.98E-06	0.0001613	Adra2a	19	54045182	54048982	+	3801	protein_coding	adrenergic receptor, alpha 2a [Source:MGI Symbol;Acc:MGI:87934]	-
ENSMUSG0000002419	506.0616507	458.1711433	547.4077313	854.4404369	872.0935228	793.988754	503.8801751	840.1742379	-0.73789368	9.19E-06	0.0001648	Cuta	17	26937973	26939478	-	649	protein_coding	cutA divalent cation tolerance homolog [Source:MGI Symbol;Acc:MGI:1914925]	-
ENSMUSG0000003227	1359.852792	523.4736971	1758.407692	3790.718108	3547.632994	3513.594841	1213.911394	3617.315314	-1.575362524	9.29E-06	0.0001665	Nnmt	9	48591877	48605153	-	1576	protein_coding	nicotinamide N-methyltransferase [Source:MGI Symbol;Acc:MGI:1099443]	-
ENSMUSG0000003829	1086.27887	829.9743929	1045.660483	604.6544221	345.6769343	347.3700799	987.3045818	432.5671454	1.189578831	9.30E-06	0.0001665	Galnt18	7	111471661	111779977	-	2575	protein_coding	polypeptide N-acetylgalactosaminyltransferase 18 [Source:MGI Symbol;Acc:MGI:2446239]	-
ENSMUSG0000004589	1262.648871	1059.586598	1135.033173	686.4808752	731.8474524	714.2006684	1152.422881	710.8429987	0.697366122	9.36E-06	0.0001675	Paip2b	6	83805401	83831741	-	6335	protein_coding	poly(A) binding protein interacting protein 2B [Source:MGI Symbol;Acc:MGI:2386865]	-
ENSMUSG0000008696	56.1177276	44.23721384	32.39760042	205.8581295	119.5054544	114.8170012	44.25084729	146.7268617	-1.728087338	9.41E-06	0.0001682	4933431E20Ri	3	107888850	107896213	-	6821	antisense	RIKEN cDNA 4933431E20 gene [Source:MGI Symbol;Acc:MGI:3584041]	-
ENSMUSG0000000245	1958.107852	2374.063809	1863.420604	1325.588541	1106.16619	1207.524563	2065.197422	1213.093098	0.767415609	9.44E-06	0.0001685	Prpf6	2	181591868	181655660	+	3859	protein_coding	pre-mRNA splicing factor 6 [Source:MGI Symbol;Acc:MGI:1922946]	-
ENSMUSG0000000866	9432.788749	8764.234675	8613.293078	6289.439587	4632.07092	4774.635804	8936.772167	5232.04877	0.772288698	9.46E-06	0.0001687	Rps18	17	33951999	33956001	-	2491	protein_coding	ribosomal protein S18 [Source:MGI Symbol;Acc:MGI:98146]	-
ENSMUSG0000002794	1309.747678	1580.953761	2411.945493	584.8438071	903.6982711	801.7729574	1767.548978	763.4383452	1.211315718	9.49E-06	0.0001691	Il6ra	3	89864059	89913196	-	8642	protein_coding	interleukin 6 receptor, alpha [Source:MGI Symbol;Acc:MGI:105304]	-
ENSMUSG0000004911	611.28239	834.1874609	650.1863257	329.8898058	394.0717051	389.2101735	698.5520589	371.0572281	0.91323524	9.51E-06	0.0001694	Agtr1a	13	30336441	30382867	+	2786	protein_coding	angiotensin II receptor, type 1a [Source:MGI Symbol;Acc:MGI:87964]	-
ENSMUSG0000002081	166.3489783	169.5759864	246.8920584	82.68778422	48.3947708	26.27168671	194.272341	52.45141391	1.884138856	9.62E-06	0.0001711	1810032O08Ri	11	116671660	116675799	+	2776	protein_coding	RIKEN cDNA 1810032O08 gene [Source:MGI Symbol;Acc:MGI:1913543]	-
ENSMUSG0000005845	184.3868193	186.4282583	102.7785944	330.7511369	411.849376	391.1562244	157.8645573	377.9189124	-1.257488493	9.63E-06	0.0001711	Dhcr7	7	143823145	143848410	+	5817	protein_coding	7-dehydrocholesterol reductase [Source:MGI Symbol;Acc:MGI:1298378]	-
ENSMUSG0000002242	5041.576564	4127.753358	3311.258195	6633.972021	8176.740969	9321.583656	4160.196039	8044.098882	-0.951161238	9.71E-06	0.0001724	Enpp2	15	54838901	54952892	-	5779	protein_coding	ectonucleotide pyrophosphatase/phosphodiesterase 2 [Source:MGI Symbol;Acc:MGI:1321390]	-
ENSMUSG0000005931	564.1835829	605.6285228	485.9640063	860.4697545	980.7348451	1007.081324	551.9253706	949.4286412	-0.782003635	9.78E-06	0.0001735	Slc27a4	2	29802634	29817522	+	4412	protein_coding	solute carrier family 27 (fatty acid transporter), member 4 [Source:MGI Symbol;Acc:MGI:1347347]	-
ENSMUSG0000002084	54.11352305	448.6917403	252.4778516	47.37320971	37.53063858	39.89404278	251.7610383	41.59929702	2.596338035	9.83E-06	0.0001743	Doc2b	11	75768966	75796049	-	4361	protein_coding	double C2, beta [Source:MGI Symbol;Acc:MGI:1100497]	-
ENSMUSG0000002127	295.6201722	307.5539629	250.2435343	565.8945232	524.4412918	467.0522082	284.4725564	519.1293411	-0.86758025	9.90E-06	0.0001753	Traf3	12	111166370	111267153	+	7350	protein_coding	TNF receptor-associated factor 3 [Source:MGI Symbol;Acc:MGI:108041]	-
ENSMUSG0000001932	16864.37925	9498.361771	7531.883519	20645.24479	33790.41415	34244.65712	11298.20818	29560.10535	-1.387521929	9.98E-06	0.0001765	Aoc3	11	101330605	101341938	+	4621	protein_coding	amine oxidase, copper containing 3 [Source:MGI Symbol;Acc:MGI:1306797]	-
ENSMUSG0000003075	1574.30268	1711.558869	1741.650312	2505.612128	2492.824521	2590.193705	1675.837287	2529.543451	-0.594104835	1.00E-05	0.0001772	Psma1	7	114264606	114276118	-	2952	protein_coding	proteasome (prosome, macropain) subunit, alpha type 1 [Source:MGI Symbol;Acc:MGI:1347005]	-
ENSMUSG0000004017	5554.65293	8307.116798	5981.267333	3810.528723	3706.644384	3120.492566	6614.345687	3545.888558	0.899411101	1.00E-05	0.0001774	Fmo2	1	162874317	162898726	-	5609	protein_coding	flavin containing monooxygenase 2 [Source:MGI Symbol;Acc:MGI:1916776]	-
ENSMUSG0000001997	446.9376163	357.0575117	440.1605023	742.4673958	692.341517	717.1197447	414.7185434	717.3095525	-0.790590511	1.02E-05	0.00018	Ikbip	10	91082940	91102607	+	4933	protein_coding	IKBKB interacting protein [Source:MGI Symbol;Acc:MGI:1914704]	-
ENSMUSG0000004339	649.3622765	544.539037	468.0894682	874.2510519	1162.462148	1120.9253	553.9969272	1052.546166	-0.925129649	1.03E-05	0.0001809	2510009E07Ri	16	21649045	21694665	-	6564	protein_coding	RIKEN cDNA 2510009E07 gene [Source:MGI Symbol;Acc:MGI:1919440]	-
ENSMUSG0000003123	112.2354552	64.24928676	61.44372494	488.3747255	186.6655445	212.1195446	79.30948897	295.7199382	-1.898097631	1.04E-05	0.0001828	Itm2a	X	107397099	107403376	-	1744	protein_coding	integral membrane protein 2A [Source:MGI Symbol;Acc:MGI:107706]	-
ENSMUSG0000004540	768.6124477	837.3472619	1190.891105	532.3026109	417.7752663	410.6167331	932.2836049	453.5648701	1.038572018	1.04E-05	0.0001829	Trim39	17	36258873	36272247	-	7223	protein_coding	tripartite motif-containing 39 [Source:MGI Symbol;Acc:MGI:1890659]	-
ENSMUSG0000005990	7.01471595	25.27840791	26.81180725	0	0	0	19.7016437	0	6.832027737	1.05E-05	0.0001839	Mug1	6	121838541	121889057	+	5770	protein_coding	murinoglobulin 1 [Source:MGI Symbol;Acc:MGI:99837]	-
ENSMUSG0000001988	27.05676152	16.85227194	35.74907633	171.404886	68.14773848	106.0597723	26.55270326	115.2041323	-2.121004477	1.06E-05	0.0001854	Rspo3	10	29452416	29535867	-	3090	protein_coding	R-spondin 3 [Source:MGI Symbol;Acc:MGI:1920030]	-
ENSMUSG0000006060	312.6559109	289.6484239	310.5701006	180.0181969	126.4189931	137.1965862	304.2914785	147.8779254	1.03897282	1.07E-05	0.000188	Eno3	11	70657202	70662513	+	1739	protein_coding	enolase 3, beta muscle [Source:MGI Symbol;Acc:MGI:95395]	-
ENSMUSG0000002142	149.3132395	136.9247095	98.3099599	282.5165961	248.8873927	362.9683158	128.1826363	298.1141585	-1.216042593	1.07E-05	0.0001882	Ly86	13	37345208	37419036	+	3632	protein_coding	lymphocyte antigen 86 [Source:MGI Symbol;Acc:MGI:1321404]	-
ENSMUSG0000002017	2768.808596	2731.121321	2381.78221	1338.508507	1800.483004	1345.694175	2627.237376	1494.895228	0.813757675	1.09E-05	0.0001903	Grb10	11	11930508	12038683	-	9089	protein_coding	growth factor receptor bound protein 10 [Source:MGI Symbol;Acc:MGI:103232]	-
ENSMUSG0000002043	1868.92075	2206.594357	6878.345717	1024.983992	1070.610848	1021.676705	3651.286941	1039.090515	1.813001992	1.09E-05	0.0001906	Igfbp3	11	7206086	7213923	-	3130	protein_coding	insulin-like growth factor binding protein 3 [Source:MGI Symbol;Acc:MGI:96438]	-
ENSMUSG0000002401	360.7568203	117.9659036	185.4483334	964.6908159	715.0574298	518.6225562	221.3903524	732.7902673	-1.72620226	1.09E-05	0.0001908	Pi16	17	29318877	29329413	+	3987	protein_coding	peptidase inhibitor 16 [Source:MGI Symbol;Acc:MGI:1921366]	-
ENSMUSG0000007450	14.0294319	14.74573795	63.67804221	111.11171	262.7144701	175.1445781	30.81773735	182.9902527	-2.575106329	1.09E-05	0.0001909	Gm10705	3	87207545	87208005	-	461	processed_pseudog	predicted gene 10705 [Source:MGI Symbol;Acc:MGI:3708678]	-
ENSMUSG0000010556	5.010511393	1.053266996	4.468634541	29.28525691	43.45652888	33.08286475	3.51080431	35.27488351	-3.326261304	1.09E-05	0.0001909	Gm43566	5	23674739	23675546	-	808	sense_intronic	predicted gene 43566 [Source:MGI Symbol;Acc:MGI:5663703]	-

ENSMUSG0000000566	71.14926178	69.51562174	138.5276708	522.827969	208.393809	266.6089689	93.06418476	332.6102489	-1.840069932	1.10E-05	0.0001913	Mthfd2	6	83305691	83325908	-	3487	protein_coding	methylenetetrahydrofolate dehydrogenase (NAD+ dependent), methenyltetrahydrofolate cyclohydrolase [Source:MGI Symbol;Acc	-
ENSMUSG0000004835	123.2585803	44.23721384	151.9335744	451.3374888	279.5044926	392.1292498	106.4764562	374.3237438	-1.814721559	1.10E-05	0.0001914	Arxes1	X	136033367	136034946	+	1580	protein_coding	adipocyte-related X-chromosome expressed sequence 1 [Source:MGI Symbol;Acc:MGI:1923469]	-
ENSMUSG0000009971	36.07568203	29.49147589	18.9916968	80.10379096	108.6413222	97.30254338	28.18628491	95.34921885	-1.75258932	1.11E-05	0.0001929	Gm28802	1	58850085	58892792	-	944	unprocessed_pseud	predicted gene 28802 [Source:MGI Symbol;Acc:MGI:5579508]	-
ENSMUSG0000001842	1009.116995	1540.929615	1049.011958	434.1108671	654.8108784	598.4106418	1199.686189	562.4441291	1.093404668	1.11E-05	0.0001929	Ypel2	11	86936425	86993707	-	4847	protein_coding	yippee like 2 [Source:MGI Symbol;Acc:MGI:1925114]	-
ENSMUSG0000002938	439.9229003	314.9268318	394.3569982	218.7780957	140.2460705	147.8998659	383.0689101	168.9746774	1.179044567	1.12E-05	0.0001938	Shroom3	5	92683435	92965318	+	13247	protein_coding	shroom family member 3 [Source:MGI Symbol;Acc:MGI:1351655]	-
ENSMUSG0000002148	1288.70353	1658.895519	1410.971356	897.5069912	884.9329518	765.7710164	1452.856802	849.4036531	0.77416749	1.12E-05	0.0001948	Aaed1	13	64275280	64312710	-	3850	protein_coding	AhpC/TSA antioxidant enzyme domain containing 1 [Source:MGI Symbol;Acc:MGI:1913379]	-
ENSMUSG0000004940	39.08198887	14.74573795	13.40590362	131.7836561	79.0118707	101.1946451	22.41121014	103.996724	-2.208429306	1.14E-05	0.0001976	Prokr1	6	87578591	87590743	-	4230	protein_coding	prokineticin receptor 1 [Source:MGI Symbol;Acc:MGI:1929676]	-
ENSMUSG0000003015	31.06517064	94.79402965	55.85793176	6.029317599	12.83942899	8.757228904	60.57237735	9.208658497	2.724274483	1.14E-05	0.0001977	Clec12b	6	129375515	129385874	-	1619	protein_coding	C-type lectin domain family 12, member B [Source:MGI Symbol;Acc:MGI:1918433]	-
ENSMUSG0000007313	717.5052315	724.6476933	824.4630728	1124.037067	1274.066415	1343.748124	755.5386659	1247.283869	-0.723253223	1.14E-05	0.0001978	Vma21	X	71815924	71839757	+	7303	protein_coding	VMA21 vacuolar H+-ATPase homolog (S. cerevisiae) [Source:MGI Symbol;Acc:MGI:1914298]	-
ENSMUSG0000003137	15850.25174	12533.87725	12463.02173	5006.917601	7993.03837	7747.228504	13615.71691	6915.728158	0.977397472	1.15E-05	0.000198	Car5b	X	163976822	164027997	-	6354	protein_coding	carbonic anhydrase 5b, mitochondrial [Source:MGI Symbol;Acc:MGI:1926249]	-
ENSMUSG0000000027	1779.733647	1891.667525	2324.80712	1012.064026	1302.708218	1044.05629	1998.736097	1119.609511	0.836158119	1.16E-05	0.0001995	Trim25	11	88999376	89020293	+	5754	protein_coding	tripartite motif-containing 25 [Source:MGI Symbol;Acc:MGI:102749]	-
ENSMUSG0000003551	1116.341938	1104.877079	1062.417862	1618.44111	1654.311043	1888.642367	1094.545626	1720.46484	-0.652280063	1.17E-05	0.000202	Tdrd7	4	45965334	46034761	+	3958	protein_coding	tudor domain containing 7 [Source:MGI Symbol;Acc:MGI:2140279]	-
ENSMUSG0000006771	5.010511393	3.159800988	1.117158635	24.1172704	35.55534181	43.78614452	3.095823672	34.48625224	-3.457751884	1.18E-05	0.0002026	Lpar5	6	125067920	125082472	+	1515	protein_coding	lysophosphatidic acid receptor 5 [Source:MGI Symbol;Acc:MGI:2685918]	-
ENSMUSG0000004363	521.0931849	536.112901	205.5571889	1136.095702	1273.078767	1047.948392	420.9210916	1152.374287	-1.45233176	1.18E-05	0.0002027	Ecm2	13	49504810	49532789	+	5677	protein_coding	extracellular matrix protein 2, female organ and adipocyte specific [Source:MGI Symbol;Acc:MGI:3039578]	-
ENSMUSG0000003736	181.3805124	213.8132002	279.2896588	100.775737	102.7154319	89.51833991	224.8277905	97.66983628	1.201540255	1.18E-05	0.0002035	Letm2	8	25578490	25597582	-	7238	protein_coding	leucine zipper-EF-hand containing transmembrane protein 2 [Source:MGI Symbol;Acc:MGI:2444979]	-
ENSMUSG0000000633	10819.6983	11476.39719	9990.749675	7515.975053	5716.508845	6389.858024	10762.28172	6540.780641	0.718390154	1.18E-05	0.0002035	Rps9	7	3703993	3706897	+	2522	protein_coding	ribosomal protein S9 [Source:MGI Symbol;Acc:MGI:1924096]	-
ENSMUSG0000002906	6430.490322	6237.447151	9589.689725	3497.004207	4400.961198	3589.469825	7419.209066	3829.152077	0.954235212	1.19E-05	0.0002049	Ccnl2	4	155812489	155824543	+	5278	protein_coding	cyclin L2 [Source:MGI Symbol;Acc:MGI:1927119]	-
ENSMUSG0000002628	618.2971059	591.9360518	649.0691671	378.1243466	370.3681439	384.3450463	619.7674416	377.6125123	0.714663275	1.20E-05	0.0002055	Ing5	1	93803965	93822101	+	4729	protein_coding	inhibitor of growth family, member 5 [Source:MGI Symbol;Acc:MGI:1922816]	-
ENSMUSG0000003372	68.14295495	27.3849419	59.20940767	302.327211	149.1349059	144.0077642	51.5791015	198.4899604	-1.944881852	1.20E-05	0.0002057	BC034090	1	155212471	155244444	-	8222	protein_coding	cDNA sequence BC034090 [Source:MGI Symbol;Acc:MGI:2672904]	-
ENSMUSG0000005485	169.3552851	104.2734326	165.339478	47.37320971	62.22184818	46.70522082	146.3227319	52.1000929	1.491140731	1.20E-05	0.0002057	Rnd1	15	98663421	98677461	-	2967	protein_coding	Rho family GTPase 1 [Source:MGI Symbol;Acc:MGI:2444878]	-
ENSMUSG0000005221	497.0427302	504.5148911	518.3616067	788.9792744	792.0940038	918.5360095	506.6397427	833.2030959	-0.717665238	1.20E-05	0.0002058	Opa3	7	19228334	19256543	+	5082	protein_coding	optic atrophy 3 [Source:MGI Symbol;Acc:MGI:2686271]	-
ENSMUSG0000004882	34.07147747	20.01207293	13.40590362	161.9302441	75.06127716	79.78808557	22.49648467	105.5932023	-2.227086273	1.21E-05	0.0002068	Dact2	17	14195052	14204304	-	3379	protein_coding	disheveled-binding antagonist of beta-catenin 2 [Source:MGI Symbol;Acc:MGI:1920347]	-
ENSMUSG0000008332	1177.470177	1284.985735	1223.288706	1747.640773	2020.728593	2090.058632	1228.581539	1952.809333	-0.668418826	1.21E-05	0.000207	Vcp-rs	X	104345829	104348234	-	2406	processed_pseudog	valosin containing protein, related sequence [Source:MGI Symbol;Acc:MGI:894298]	-
ENSMUSG0000004752	221.4646036	127.4453065	191.0341266	339.3644477	550.1201497	424.2390891	179.9813456	437.9078955	-1.281917855	1.22E-05	0.0002074	Als2cr12	1	58646903	58695989	-	3396	protein_coding	amyotrophic lateral sclerosis 2 chromosome region 12 [Source:MGI Symbol;Acc:MGI:1918359]	-
ENSMUSG0000003889	3054.407745	2200.274755	4459.697272	778.6433014	1667.150472	1200.713385	3238.126591	1215.502386	1.413791432	1.22E-05	0.0002076	Irs2	8	10984681	11008458	-	6323	protein_coding	insulin receptor substrate 2 [Source:MGI Symbol;Acc:MGI:109334]	-
ENSMUSG0000003233	821.7238685	323.3529678	958.522109	1928.520301	2078.012199	1884.750265	701.1996484	1963.760922	-1.485833753	1.24E-05	0.0002103	Loxl1	9	58287738	58313186	-	3297	protein_coding	lysyl oxidase-like 1 [Source:MGI Symbol;Acc:MGI:106096]	-
ENSMUSG0000011592	9.018920508	11.58593696	0	37.89856777	88.88835454	79.78808557	6.868285822	68.85833596	-3.313180695	1.24E-05	0.000211	Gm33432	15	84127304	84136259	+	1578	lincRNA	predicted gene, 33432 [Source:MGI Symbol;Acc:MGI:5592591]	-
ENSMUSG0000002257	17.03573874	9.479402965	7.820110447	73.21314228	59.25890302	43.78614452	11.44508405	58.75272994	-2.353592775	1.26E-05	0.000214	Ly6h	15	75551268	75567255	-	4227	protein_coding	lymphocyte antigen 6 complex, locus H [Source:MGI Symbol;Acc:MGI:1346030]	-
ENSMUSG0000001242	761.5977317	702.5290864	856.8606732	1177.439594	1316.535296	1311.638285	773.6624971	1268.537725	-0.713461357	1.27E-05	0.0002154	Tmem167	13	90089123	90143907	+	10807	protein_coding	transmembrane protein 167 [Source:MGI Symbol;Acc:MGI:1913324]	-
ENSMUSG0000002796	277.5823312	274.902686	239.0719479	503.8786851	436.5405856	463.1601065	263.8523217	467.8597924	-0.826156542	1.27E-05	0.0002154	Extl2	3	116007462	116029017	+	3447	protein_coding	exostoses (multiple)-like 2 [Source:MGI Symbol;Acc:MGI:1889574]	-
ENSMUSG0000004137	5710.980886	5095.705727	6675.022845	3097.346584	3630.595459	3703.334801	5827.236486	3477.092281	0.744974025	1.31E-05	0.0002227	Cldn5	16	18776847	18778262	+	1416	protein_coding	claudin 5 [Source:MGI Symbol;Acc:MGI:1276112]	-
ENSMUSG0000005443	901.8920508	864.7322038	1097.04978	574.5078341	578.7619529	517.6495308	954.5580114	556.9731059	0.776840205	1.33E-05	0.0002252	Gimap4	6	48684549	48692060	+	1837	protein_coding	GTPase, IMAP family member 4 [Source:MGI Symbol;Acc:MGI:1349656]	-
ENSMUSG0000006093	412.8661388	340.2052397	435.6918677	678.7288954	667.6503074	709.3355412	396.2544154	685.238248	-0.790364997	1.34E-05	0.0002262	Tmem263	10	85102627	85120618	+	4014	protein_coding	transmembrane protein 263 [Source:MGI Symbol;Acc:MGI:2143652]	-
ENSMUSG0000003726	10716.48177	10471.58048	15227.98936	6939.744557	7004.402338	6499.809898	12138.68387	6814.652264	0.832861953	1.35E-05	0.0002279	Rsrp1	4	134923592	134927671	+	3349	protein_coding	arginine/serine rich protein 1 [Source:MGI Symbol;Acc:MGI:106498]	-
ENSMUSG0000003669	695.4589814	817.335189	812.1743278	476.3160903	472.0959274	459.2680047	774.9894994	469.2266742	0.72354674	1.36E-05	0.000229	Nop14	5	34638536	34660148	-	2730	protein_coding	NOP14 nucleolar protein [Source:MGI Symbol;Acc:MGI:1922666]	-
ENSMUSG0000001663	194.4078421	186.4282583	207.7915062	413.4389211	324.9363183	365.8575631	196.2092022	368.0776008	-0.908606368	1.37E-05	0.0002307	Ift27	15	78159463	78174107	-	2864	protein_coding	intraflagellar transport 27 [Source:MGI Symbol;Acc:MGI:1914292]	-
ENSMUSG0000005736	287.603354	342.3117737	307.2186247	537.4705974	508.6389176	585.7613111	312.3779175	543.9569421	-0.800370819	1.37E-05	0.0002314	Uxs1	1	43746966	43827800	-	5097	protein_coding	UDP-glucuronate decarboxylase 1 [Source:MGI Symbol;Acc:MGI:1915133]	-
ENSMUSG0000003568	234.4919332	370.7499826	227.9003616	129.1996628	109.6289706	113.8439758	277.7140925	117.5575364	1.239522233	1.38E-05	0.0002318	Kcnc2	10	112271121	112467024	+	7138	protein_coding	potassium voltage gated channel, Shaw-related subfamily, member 2 [Source:MGI Symbol;Acc:MGI:96668]	-
ENSMUSG0000003947	6.012613672	5.26633498	6.702951811	60.29317599	29.62945151	36.00194105	5.993966821	41.97485618	-2.812179303	1.38E-05	0.0002328	Prrx2	2	30834972	30881251	+	1961	protein_coding	paired related homeobox 2 [Source:MGI Symbol;Acc:MGI:98218]	Hom
ENSMUSG0000003345	226.475115	473.9701482	604.3828217	1960.389551	873.0811712	1251.310708	434.942695	1361.59381	-1.647110362	1.39E-05	0.0002329	Adamts15	9	30899155	30922452	-	10363	protein_coding	a disintegrin-like and metallopeptidase (reprolysin type) with thrombospondin type 1 motif, 15 [Source:MGI Symbol;Acc:MGI:244	-
ENSMUSG0000003378	1811.80092	1218.629914	2016.471337	807.0672272	827.6493456	935.0774419	1682.300724	856.5980049	0.973767711	1.39E-05	0.0002341	Dysf	6	84008590	84211060	+	8328	protein_coding	dysferlin [Source:MGI Symbol;Acc:MGI:1349385]	-
ENSMUSG0000002619	520.0910826	609.8415907	699.3413056	352.284414	322.9610215	342.5049527	609.7579993	339.2501294	0.84513533	1.44E-05	0.000241	Ankzf1	1	75192151	75199387	+	5142	protein_coding	ankyrin repeat and zinc finger domain containing 1 [Source:MGI Symbol;Acc:MGI:1098746]	-
ENSMUSG0000005464	102.2144324	76.88849072	29.04612452	238.5887107	203.4555671	298.7188082	69.38301588	246.9210286	-1.828211095	1.45E-05	0.0002423	Slc8a1	17	81388691	81649607	-	2934	protein_coding	solute carrier family 8 (sodium/calcium exchanger), member 1 [Source:MGI Symbol;Acc:MGI:107956]	-
ENSMUSG0000002494	4257.932582	2948.094322	3859.783085	6077.55214	6626.133007	6393.750125	3688.60333	6365.811757	-0.787224932	1.45E-05	0.0002425	Ltbp3	19	5740904	5758532	+	5190	protein_coding	latent transforming growth factor beta binding protein 3 [Source:MGI Symbol;Acc:MGI:1101355]	-
ENSMUSG0000003823	1843.868193	1941.171074	1908.106949	1336.785845	1070.610848	1043.083265	1897.715405	1150.159986	0.722007928	1.46E-05	0.0002447	F11r	1	171437535	171464603	+	2663	protein_coding	F11 receptor [Source:MGI Symbol;Acc:MGI:1321398]	-
ENSMUSG0000004402	70.1471595	64.24928676	33.51475906	464.2574551	151.1102027	144.0077642	55.97040177	253.1251407	-2.176450032	1.47E-05	0.0002463	Slc35g1	19	38395980	38405607	+	3471	protein_coding	solute carrier family 35, member G1 [Source:MGI Symbol;Acc:MGI:2444789]	-
ENSMUSG0000002167	337.7084679	225.3991372	274.8210243	1260.988709	483.947708	656.7921678	279.3095431	800.576195	-1.519430952	1.47E-05	0.0002463	Iqgap2	13	95627177	95891922	-	5771	protein_coding	IQ motif containing GTPase activating protein 2 [Source:MGI Symbol;Acc:MGI:2449975]	-
ENSMUSG0000000151	14.0294319	20.01207293	23.46033134	136.9516426	53.33301272	73.94993297	19.16727872	88.0781961	-2.205756692	1.49E-05	0.000249	Foxm1	6	128362967	128376146	+	5754	protein_coding	forkhead box M1 [Source:MGI Symbol;Acc:MGI:1347487]	Fork
ENSMUSG0000003666	2035.269728	1394.525503	1806.445513	2788.990055	3272.079095	4289.096112	1745.413581	3450.055088	-0.982926522	1.49E-05	0.0002493	Tcaf1	6	42668002	42710088	-	9638	protein_coding	TRPM8 channel-associated factor 1 [Source:MGI Symbol;Acc:MGI:1914665]	-
ENSMUSG0000001315	693.4547768	690.9431494	666.9437052	441.8628469	423.7011566	376.5608429	683.7805438	414.0416155	0.723430729	1.50E-05	0.0002507	Gfod2	8	105713854	105758664	-	4608	protein_coding	glucose-fructose oxidoreductase domain containing 2 [Source:MGI Symbol;Acc:MGI:1917825]	-
ENSMUSG0000003970	730.5325611	817.335189	811.0571692	517.6599824	415.7999696	455.375903	786.3083064	462.945285	0.763425164	1.51E-05	0.0002515	Ldb2	5	44472132	44799680	-	3336	protein_coding	LIM domain binding 2 [Source:MGI Symbol;Acc:MGI:894670]	-
ENSMUSG0000002750	336.7063656	227.5056712	644.6005325	124.0316763	130.3695867	142.0617133	402.9375231	132.1543254	1.607998031	1.52E-05	0.000252	Stmn2	3	8509360	8561606	+	5602	protein_coding	stathmin-like 2 [Source:MGI Symbol;Acc:MGI:98241]	-
ENSMUSG0000003776	66.13875039	81.1015587	106.1300703	249.7860148	159.9990382	243.2563584	84.45679314	217.6804705	-1.368642952	1.52E-05	0.0002523	Slc16a9	10	70245100	70285968	+	3869	protein_coding	solute carrier family 16 (monocarboxylic acid transporters), member 9 [Source:MGI Symbol;Acc:MGI:1914109]	-
ENSMUSG0000000352	3934.253546	3496.846427	1669.035001	5619.324002	9954.50806	8481.862706	3033.378325	8018.564923	-1.402298051	1.53E-05	0.0002541	Slc25a1	16	17925223	17928219	-	2164	protein_coding	solute carrier family 25 (mitochondrial carrier, citrate transporter), member 1 [Source:MGI Symbol;Acc:MGI:1345283]	-
ENSMUSG0000007540	25.05255697	11.58593696	41.3348695	186.9088456	84.937761	100.2216197	25.99112114	124.0227421	-2.259243149	1.54E-05	0.0002551	6030408B16Ri	15	101293212	101297426	+	1967	protein_coding	RIKEN cDNA 6030408B16 gene [Source:MGI Symbol;Acc:MGI:1924967]	-
ENSMUSG0000003253	5214.940258	1865.33585	2777.256367	8092.205549	9362.906678	8560.677766	3285.844158	8671.929998	-1.400005995	1.54E-05	0.0002557	Amotl2	9	102716672	102733418	+	6289	protein_coding	angiomotin-like 2 [Source:MGI Symbol;Acc:MGI:1929286]	-
ENSMUSG0000002161	345.7252861	437.1058034	576.4538558	813.0965448	1022.216077	911.7248315	453.0949818	915.6791511	-1.015612859	1.54E-05	0.0002557	Vcan	13	89655312	89742509	-	14462	protein_coding	versican [Source:MGI Symbol;Acc:MGI:102889]	-
ENSMUSG0000003010	460.9670482	117.9659036	539.5876208	1110.255769	1551.595611	1411.859904	372.8401908	1357.903762	-1.864849355	1.55E-05	0.0002557	Slc6a13	6	121300227	121337733	+	2904	protein_coding	solute carrier family 6 (neurotransmitter transporter, GABA), member 13 [Source:MGI Symbol;Acc:MGI:95629]	-
ENSMUSG0000003914	144.3027281	116.9126366	88.25553218	270.4579609	240.9862056	262.7168671	116.490299	258.0536779	-1.145572431	1.56E-05	0.0002578	Camk1d	2	5293457	5714515	-	7427	protein_coding	calcium/calmodulin-dependent protein kinase ID [Source:MGI Symbol;Acc:MGI:2442190]	-
ENSMUSG0000000634	163.3426714	89.52769467	108.3643876	700.2621726	301.232757	252.0135873	120.4115846	417.8361723	-1.794948033	1.56E-05	0.0002582	Susd2	10	75636706	75644008	-	3435	protein_coding	sushi domain containing 2 [Source:MGI Symbol;Acc:MGI:1918983]	-
ENSMUSG0000002494	4525.49389	4100.368416	6104.154783	2848.4219	2758.501936	2705.983731	4910.005696	2770.969189	0.825239876	1.57E-05	0.0002589	Sf1	19	6363690	6378030	+	6211	protein_coding	splicing factor 1 [Source:MGI Symbol;Acc:MGI:1095403]	-
ENSMUSG0000006160	14.0294319	23.17187391	13.40590362	42.20522319	96.78954161	106.0597723	16.86906981	81.68484569	-2.273238894	1.60E-05	0.0002638	Pclo	5	14514918	14863459	+	22457	protein_coding	piccolo (presynaptic cytomatrix protein) [Source:MGI Symbol;Acc:MGI:1349390]	-
ENSMUSG0000009086	3323.973258	3214.570872	3378.287713	2090.450545	1749.125288	1161.792368	3305.610614	1667.122733	0.987341143	1.61E-05	0.0002644	Rps13	7	116331505	116334195	-	2691	protein_coding	ribosomal protein S13 [Source:MGI Symbol;Acc:MGI:1915302]	-
ENSMUSG0000001588	1994.183534	1645.203048	2198.568194	1175.716932	1183.202764	1159.846317	1945.984925	1172.922004	0.730306153	1.61E-05	0.0002655	Lta4h	10	93453411	93484875	+	6102	protein_coding	leukotriene A4 hydrolase [Source:MGI Symbol;Acc:MGI:96836]	-
ENSMUSG0000011453	13.02732962	6.319601977	24.57748997	52.54119622	82.96246423	97.30254338	14.64147386	77.60206794	-2.41111814	1.62E-05	0.0002665	Gm31517	14	15015189	15065231	-	4170	lincRNA	predicted gene, 31517 [Source:MGI Symbol;Acc:MGI:5590676]	-
ENSMUSG0000004958	712.4947201	683.5702805	774.1909342	328.1671436	475.0588726	367.803614	723.4186449	390.3432101	0.890800276	1.65E-05	0.0002711	Ccdc69	11	55049731	55078131	-	1848	protein_coding	coiled-coil domain containing 69 [Source:MGI Symbol;Acc:MGI:1196234]	-
ENSMUSG0000001574	840.7638118	766.7783732	794.2997896	529.7186176	418.7629147	472.8903608	800.6139915	473.7906311	0.756270045	1.66E-05	0.0002718	Plekho1	3	95988429	95996001	-	2172	protein_coding	pleckstrin homology domain containing, family O member 1 [Source:MGI Symbol;Acc:MGI:1914470]	-
ENSMUSG0000003174	868.8226756	642.4928676	449.0977714	1271.324682	1359.004176	1607.438017	653.4711048	1412.588958	-1.111512204	1.67E-05	0.0002742	St3gal2	8	110919922	110972480	+	5041	protein_coding	ST3 beta-galactoside alpha-2,3-sialyltransferase 2 [Source:MGI Symbol;Acc:MGI:99427]	-
ENSMUSG0000005321	58.12193216	168.5227194	65.91235948	15.50395954	25.67885798	17.51445781	97.51900367	19.56575844	2.320365598	1.68E-05	0.0002751	Gm9899	5	30573987	30588619	-	6195	processed_transcrip	predicted gene 9899 [Source:MGI Symbol;Acc:MGI:3708711]	-
ENSMUSG0000001655	105.2207393	74.78195672	42.45202814	412.57759	213.3320509	176.1176035	74.15157471	267.3424148	-1.848758704	1.69E-05	0.0002763	Foxred2	15	77940522	77956722	-	5939	protein_coding	FAD-dependent oxidoreductase domain containing 2 [Source:MGI Symbol;Acc:MGI:106315]	-
ENSMUSG0000002883	946.9866533	1060.639865	978.6309645	1699.406232	1469.620795	1532.515058	995.4191609	1567.180695	-0.655028624	1.71E-05	0.0002797	Psmb2	4	126677630	126709714	+	1204	protein_coding	proteasome (prosome, macropain) subunit, beta type 2 [Source:MGI Symbol;Acc:MGI:1347045]	-
ENSMUSG0000002416	434.9123889	394.9751235	470.3237854	755.3873621	669.6256042	754.0947112	433.403766	726.3692258	-0.745428663	1.72E-05	0.0002806	Jpt2	17	24937419	24960689	-	4840	protein_coding	Jupiter microtubule associated homolog 2 [Source:MGI Symbol;Acc:MGI:1196260]	-
ENSMUSG0000004633	6485.605947	6156.345592	6444.888167	4586.588031	3494.299982	3632.303944	6362.279902	3904.397319	0.704310779	1.74E-05	0.0002838	Rpl37a	1	72711290	72713813	+	1410	protein_coding	ribosomal protein L37a [Source:MGI Symbol;Acc:MGI:98068]	-
ENSMUSG0000002250	87.18289824	52.6633498	22.3431727	195.5221564	188.6408413	211.1465191	54.06314025	198.4365056	-1.871717338	1.74E-05	0.0002838	Ciita	16	10480059	10528418	+	9535	protein_coding	class II transactivator [Source:MGI Symbol;Acc:MGI:108445]	-
ENSMUSG0000003471	646.3559697	462.3842113	586.5082835	888.0323492	1117.030322	1127.736478	565.0828215	1044.266383	-0.885548662	1.75E-05	0.0002848	Ttyh2	11	114675431	114720977	+	3739	protein_coding	tweety family member 2 [Source:MGI Symbol;Acc:MGI:2157091]	-
ENSMUSG0000004015	25067.5885	5010.3911	20469.69767	678.7288954	2896.77271	1262.987013	16849.22576	1612.829539	3.385061443	1.77E-05	0.0002885	Thbs1	2	118111876	118127133	+	6073	protein_coding	thrombospondin 1 [Source:MGI Symbol;Acc:MGI:98737]	-
ENSMUSG0000008518	393.8261955	302.2876279	530.6503517	192.9381632	194.5667316	170.2794509	408.9213917	185.9281152	1.136402871	1.78E-05	0.0002899	AI838599	4	3242055	3258092	+	4243	lincRNA	expressed sequence AI838599 [Source:MGI Symbol;Acc:MGI:3510989]	-
ENSMUSG0000009048	121.2543757	35.81107787	135.1761949	315.2471773	363.4546052	337.6398255	97.41388281	338.780536	-1.798506178	1.79E-05	0.0002902	BC035947	1	78497026	78512158	-	3310	protein_coding	cDNA sequence BC035947 [Source:MGI Symbol;Acc:MGI:2652858]	-
ENSMUSG0000002971	3607.568203	2823.808817	1901.403997	1409.998987	1279.004657	1368.07376	2777.593672	1352.359135	1.038456054	1.79E-05	0.0002904	Ephb4	5	137350109	137378669	+	4640	protein_coding	Eph receptor B4 [Source:MGI Symbol;Acc:MGI:104757]	-
ENSMUSG0000001546	1767.708419	2339.305998	1828.788686	1193.804885	1221.721051	1087.842435	1978.601035	1167.789457	0.760660742	1.81E-05	0.0002935	Notch4	17	34564268	34588503	+	8061	protein_coding	notch 4 [Source:MGI Symbol;Acc:MGI:107471]	-
ENSMUSG0000002519	272.5718198	314.9268318	272.586707	168.8208928	125.4313447	130.3854081	286.6951195	141.5458819	1.016388193	1.81E-05	0.0002935	Cutc	19	43752996	43768638	+	1422	protein_coding	cutC copper transporter [Source:MGI Symbol;Acc:MGI:1913638]	-
ENSMUSG0000000237	369.7757408	423.4133324	389.8883637	745.9127201	650.8602849	617.8711504	394.3591456	671.5480518	-0.768516525	1.82E-05	0.000295	Ndufa11	17	56717767	56722141	+	1092	protein_coding	NADH:ubiquinone oxidoreductase subunit A11 [Source:MGI Symbol;Acc:MGI:1917125]	-
ENSMUSG0000000230	672.410629	740.4466983	752.9649201	1116.285087	1115.055025	1127.736478	721.9407491	1119.692197	-0.633401659	1.84E-05	0.0002985	Cd320	17	33843091	33849774	+	2592	protein_coding	CD320 antigen [Source:MGI Symbol;Acc:MGI:1860083]	-
ENSMUSG0000001602	939.9719373	1938.011273	1368.519328	4138.695866	2648.872965	3089.355752	1415.500846	3292.308195	-1.217953433	1.92E-05	0.0003107	Lbp	2	158306493	158332852	+	4831	protein_coding	lipopolysaccharide binding protein [Source:MGI Symbol;Acc:MGI:1098776]	-
ENSMUSG0000002536	1702.571771	1821.098636	1940.504549	1244.623419	1097.277354	1177.360775	1821.391652	1173.087183	0.634406134	1.94E-05	0.0003133	Pa2g4	10	128557766	128565987	-	2804	protein_coding	proliferation-associated 2G4 [Source:MGI Symbol;Acc:MGI:894684]	-
ENSMUSG0000004616	0	0	0	18.0879528	11.8517806	21.40655954	0	17.11543098	-6.461106407	1.95E-05	0.0003143	Gldn	9	54286486	54341786	+	4658	protein_coding	gliomedin [Source:MGI Symbol;Acc:MGI:2388361]	-
ENSMUSG0000002235	1651.464555	1494.585867	1471.297923	983.6400997	1002.46311	1031.40696	1539.116115	1005.836721	0.613921574	1.95E-05	0.0003149	Ndufb9	15	58933756	58939488	+	1031	protein_coding	NADH:ubiquinone oxidoreductase subunit B9 [Source:MGI Symbol;Acc:MGI:1913468]	-
ENSMUSG0000000539	8956.790166	5614.966356	7520.711932	11179.21616	16185.58171	18611.05747	7364.156152	15325.28511	-1.057285758	1.98E-05	0.0003183	Nid1	13	13437551	13512269	+	6943	protein_coding	nidogen 1 [Source:MGI Symbol;Acc:MGI:97342]	-
ENSMUSG0000003065	28.0588638	28.43820889	14.52306226	224.8074133	74.07362878	71.03085667	23.67337832	123.3039663	-2.379894967	2.01E-05	0.000323	Tmc5	7	118597297	118675086	+	4470	protein_coding	transmembrane channel-like gene family 5 [Source:MGI Symbol;Acc:MGI:1921674]	-
ENSMUSG0000003700	5289.095827	5836.152425	6306.360496	3612.422573	4063.185451	3518.459969	5810.536249	3731.355997	0.638958312	2.01E-05	0.0003239	Tns2	15	102100413	102116401	+	5452	protein_coding	tensin 2 [Source:MGI Symbol;Acc:MGI:2387586]	-
ENSMUSG0000002012	91.19130735	173.7890544	55.85793176	17.22662171	21.72826444	31.13681388	106.9460978	23.36390001	2.197969189	2.02E-05	0.000324	Elane	10	79886247	79888215	+	984	protein_coding	elastase, neutrophil expressed [Source:MGI Symbol;Acc:MGI:2679229]	-
ENSMUSG0000003641	48.10090937	18.95880593	35.74907633	99.05307484	176.7890607	107.0327977	34.26959721	127.6249777	-1.894374537	2.03E-05	0.0003263	Arsi	18	60911780	60918561	+	2975	protein_coding	arylsulfatase i [Source:MGI Symbol;Acc:MGI:2670959]	-
ENSMUSG0000003381	478.0027869	391.8153225	337.3819078	714.04347	719.9956718	720.038821	402.4000058	718.0259876	-0.834548841	2.04E-05	0.0003278	Ppp1r16a	15	76671615	76694919	+	4119	protein_coding	protein phosphatase 1, regulatory subunit 16A [Source:MGI Symbol;Acc:MGI:1920312]	-
ENSMUSG0000003156	76.15977317	56.87641779	60.3265663	165.3755684	135.3078286	155.6840694	64.45425242	152.1224888	-1.238011302	2.04E-05	0.0003278	Tenm3	8	48227682	48843951	-	10237	protein_coding	teneurin transmembrane protein 3 [Source:MGI Symbol;Acc:MGI:1345183]	-
ENSMUSG0000002655	4511.464458	4495.343539	4583.70188	3165.39174	2991.586954	3227.525364	4530.169959	3128.168019	0.534211432	2.07E-05	0.0003318	Dcaf8	1	172148084	172197005	+	12996	protein_coding	DDB1 and CUL4 associated factor 8 [Source:MGI Symbol;Acc:MGI:91860]	-
ENSMUSG0000004270	334.7021611	369.6967156	313.9215765	560.7265367	595.5519754	552.6784464	339.4401511	569.6523195	-0.746578989	2.08E-05	0.000333	Commd10	18	46958862	47087992	+	1561	protein_coding	COMM domain containing 10 [Source:MGI Symbol;Acc:MGI:1916706]	-
ENSMUSG0000008757	0	0	3.351475906	19.81061497	40.49358373	31.13681388	1.117158635	30.48033753	-4.82579938	2.08E-05	0.000333	Hectd2os	19	36614052	36705641	-	3977	processed_transcrip	Hectd2, opposite strand [Source:MGI Symbol;Acc:MGI:1919243]	-
ENSMUSG0000005940	151.3174441	166.4161854	138.5276708	390.1829818	263.7021185	297.7457827	152.0871001	317.2102943	-1.061176614	2.08E-05	0.000333	Mamld1	X	71050256	71156056	+	4796	protein_coding	mastermind-like domain containing 1 [Source:MGI Symbol;Acc:MGI:3045303]	-
ENSMUSG0000001772	934.961426	786.7904461	912.718605	1536.614657	1386.658331	1327.206692	878.1568257	1416.82656	-0.690266825	2.10E-05	0.0003355	Pigt	2	164497520	164508301	+	3856	protein_coding	phosphatidylinositol glycan anchor biosynthesis, class T [Source:MGI Symbol;Acc:MGI:1926178]	-
ENSMUSG0000008339	80.16818229	77.94175771	107.247229	180.0181969	187.6531929	251.0405619	88.45238966	206.2373172	-1.222364785	2.14E-05	0.000341	Gm15542	7	140890887	140892080	+	1194	processed_pseudog	predicted gene 15542 [Source:MGI Symbol;Acc:MGI:3782990]	-
ENSMUSG0000000768	1.002102279	1.053266996	1.117158635	16.36529063	27.65415474	35.02891562	1.057509303	26.34945366	-4.639050776	2.14E-05	0.0003411	Dio2	12	90724552	90738438	-	5815	protein_coding	deiodinase, iodothyronine, type II [Source:MGI Symbol;Acc:MGI:1338833]	-
ENSMUSG0000003645	636.3349469	1011.136316	836.7518178	410.8549278	405.9234857	468.9982591	828.0743603	428.5922242	0.950068558	2.15E-05	0.0003431	Arhgap26	18	38601534	39376284	+	17331	protein_coding	Rho GTPase activating protein 26 [Source:MGI Symbol;Acc:MGI:1918552]	-
ENSMUSG0000002082	95.19971647	108.4865006	68.14667675	219.6394268	204.4432154	191.6860105	90.61096461	205.2562176	-1.178383581	2.17E-05	0.0003459	Slc46a1	11	78465697	78472059	+	3080	protein_coding	solute carrier family 46, member 1 [Source:MGI Symbol;Acc:MGI:1098733]	-
ENSMUSG0000002551	1309.747678	1815.832301	1968.433515	999.1440593	890.8588421	922.4281112	1698.004498	937.4770042	0.856631782	2.18E-05	0.0003465	Tspan4	7	141475240	141493427	+	2178	protein_coding	tetraspanin 4 [Source:MGI Symbol;Acc:MGI:1928097]	-
ENSMUSG0000002957	64.13454583	69.51562174	68.14667675	142.9809602	165.9249285	142.0617133	67.26561477	150.322534	-1.160060626	2.19E-05	0.0003473	Radil	5	142484839	142551098	-	4803	protein_coding	Ras association and DIL domains [Source:MGI Symbol;Acc:MGI:2443088]	-
ENSMUSG0000002672	13267.83417	9694.269432	18158.29646	24458.35751	28704.02498	30098.59574	13706.80002	27753.65941	-1.017799728	2.20E-05	0.0003484	Vim	2	13573927	13582826	+	4743	protein_coding	vimentin [Source:MGI Symbol;Acc:MGI:98932]	-
ENSMUSG0000004466	1.002102279	0	1.117158635	41.34389211	39.50593535	11.67630521	0.706420305	30.84204422	-5.454092876	2.21E-05	0.0003512	Plppr4	3	117319139	117360876	-	7144	protein_coding	phospholipid phosphatase related 4 [Source:MGI Symbol;Acc:MGI:106530]	-
ENSMUSG0000005597	37.07778431	73.72868973	48.03782131	13.78129737	5.925890302	8.757228904	52.94809845	9.488138859	2.469923125	2.22E-05	0.0003515	Fut2	7	45648591	45666394	-	5431	protein_coding	fucosyltransferase 2 [Source:MGI Symbol;Acc:MGI:109374]	-
ENSMUSG0000000228	13591.5132	12385.36661	21595.79358	4604.675984	8781.18178	7021.35153	15857.5578	6802.403098	1.221085776	2.22E-05	0.0003515	Angptl4	17	33773750	33781575	-	3286	protein_coding	angiopoietin-like 4 [Source:MGI Symbol;Acc:MGI:1888999]	-
ENSMUSG0000003159	43.09039798	353.8977107	97.19280126	15.50395954	31.60474828	21.40655954	164.72697	22.83842245	2.852987999	2.22E-05	0.0003515	Fgl1	8	41191434	41215156	-	4434	protein_coding	fibrinogen-like protein 1 [Source:MGI Symbol;Acc:MGI:102795]	-
ENSMUSG0000000641	572.2004011	410.7741285	575.3366971	306.6338665	243.9491508	234.4991295	519.4370756	261.6940489	0.988096193	2.23E-05	0.000352	Pfdn2	1	171345670	171359254	+	1937	protein_coding	prefoldin 2 [Source:MGI Symbol;Acc:MGI:1276111]	-
ENSMUSG0000007853	11.02312506	8.426135969	18.9916968	43.06655428	72.09833201	67.13875493	12.81365261	60.76788041	-2.249958467	2.24E-05	0.0003541	Nkain1	4	130531602	130574173	-	1426	protein_coding	Na+/K+ transporting ATPase interacting 1 [Source:MGI Symbol;Acc:MGI:1914399]	-
ENSMUSG0000000274	1251.625746	1369.247095	1109.338525	1963.834875	1868.630742	2149.413183	1243.403789	1993.9596	-0.681182973	2.25E-05	0.0003545	Ykt6	11	5955693	5967780	+	2984	protein_coding	YKT6 v-SNARE homolog (S. cerevisiae) [Source:MGI Symbol;Acc:MGI:1927550]	-
ENSMUSG0000001484	605.2697763	274.902686	588.7426008	1011.202694	1559.496798	1145.250936	489.6383543	1238.650143	-1.338858996	2.25E-05	0.0003546	Tppp3	8	105467493	105471526	-	1848	protein_coding	tubulin polymerization-promoting protein family member 3 [Source:MGI Symbol;Acc:MGI:1915221]	-
ENSMUSG0000002167	262.550797	263.316749	300.5156729	552.9745569	479.0094661	440.7805215	275.461073	490.9215149	-0.834665005	2.26E-05	0.0003561	Hmgcr	13	96648967	96670936	-	7275	protein_coding	3-hydroxy-3-methylglutaryl-Coenzyme A reductase [Source:MGI Symbol;Acc:MGI:96159]	-
ENSMUSG0000003223	4715.893323	4830.282244	2360.556196	1373.823082	1934.803184	1838.045044	3968.910655	1715.557103	1.210253431	2.27E-05	0.0003572	Cgnl1	9	71626509	71771602	-	7844	protein_coding	cingulin-like 1 [Source:MGI Symbol;Acc:MGI:1915428]	-
ENSMUSG0000002000	6621.891857	6904.165159	8962.96373	4516.820213	4298.245766	4725.011506	7496.340249	4513.359162	0.731925645	2.28E-05	0.000359	Ifngr1	10	19591949	19610229	+	2847	protein_coding	interferon gamma receptor 1 [Source:MGI Symbol;Acc:MGI:107655]	-
ENSMUSG0000002490	284.5970471	230.6654721	178.7453816	80.10379096	110.616619	105.0867468	231.1379967	98.6023856	1.232982999	2.30E-05	0.0003608	Peli3	19	4930651	4943127	-	3786	protein_coding	pellino 3 [Source:MGI Symbol;Acc:MGI:1924963]	-
ENSMUSG0000010352	92.19340963	94.79402965	55.85793176	5.167986514	23.70356121	17.51445781	80.94845701	15.46200184	2.399232649	2.31E-05	0.0003627	Gm37261	3	51672801	51673901	+	1101	TEC	predicted gene, 37261 [Source:MGI Symbol;Acc:MGI:5610489]	-
ENSMUSG0000000078	15.03153418	13.69247095	6.702951811	250.6473459	42.4688805	36.00194105	11.80898565	109.7060558	-3.213780725	2.39E-05	0.0003741	Tcf7	11	52252371	52283014	-	2625	protein_coding	transcription factor 7, T cell specific [Source:MGI Symbol;Acc:MGI:98507]	HMC
ENSMUSG0000002067	81.17028457	115.8593696	149.6992571	43.92788537	14.81472576	28.21773758	115.5763038	28.9867829	1.988145364	2.39E-05	0.000375	Sntg2	12	30174482	30373375	-	3410	protein_coding	syntrophin, gamma 2 [Source:MGI Symbol;Acc:MGI:1919541]	-
ENSMUSG0000002006	889.8668234	802.589451	1433.314529	508.1853405	519.5030499	462.187081	1041.923601	496.6251571	1.068661805	2.40E-05	0.0003753	Hnrnph3	10	63014664	63024217	-	3151	protein_coding	heterogeneous nuclear ribonucleoprotein H3 [Source:MGI Symbol;Acc:MGI:1926462]	-
ENSMUSG0000009780	39.08198887	45.29048083	84.90405628	7.75197977	3.950593535	14.59538151	56.42550866	8.765984937	2.685570981	2.41E-05	0.0003767	Gm16685	3	7612705	7690001	+	1592	antisense	predicted gene, 16685 [Source:MGI Symbol;Acc:MGI:4439609]	-
ENSMUSG0000009248	24.05045469	24.22514091	20.10885543	74.93580445	70.12303525	71.03085667	22.79481701	72.02989879	-1.658546813	2.41E-05	0.0003775	2610524H06Ri	5	114821935	114823468	-	947	protein_coding	RIKEN cDNA 2610524H06 gene [Source:MGI Symbol;Acc:MGI:2447819]	-
ENSMUSG0000005006	1323.77711	541.379236	2038.814509	294.5752313	475.0588726	432.0232926	1301.323618	400.5524655	1.700132789	2.43E-05	0.0003797	Grem2	1	174833785	174921819	-	3745	protein_coding	gremlin 2, DAN family BMP antagonist [Source:MGI Symbol;Acc:MGI:1344367]	-
ENSMUSG0000002980	1231.5837	2438.313096	1532.741648	944.8802009	646.9096914	664.5763713	1734.212815	752.1220878	1.204874147	2.44E-05	0.0003801	Abcg2	6	58584523	58695676	+	8153	protein_coding	ATP binding cassette subfamily G member 2 (Junior blood group) [Source:MGI Symbol;Acc:MGI:1347061]	-
ENSMUSG0000002858	1241.604723	1288.145536	1137.267491	1799.320638	1863.6925	2184.442099	1222.33925	1949.151746	-0.672982081	2.44E-05	0.0003803	Laptm5	4	130913125	130936141	+	3788	protein_coding	lysosomal-associated protein transmembrane 5 [Source:MGI Symbol;Acc:MGI:108046]	-
ENSMUSG0000002490	276.5802289	231.7187391	221.1974098	603.793091	461.2317952	398.9404278	243.1654593	487.988438	-1.004986625	2.44E-05	0.0003803	Efemp2	19	5473973	5481853	+	2648	protein_coding	epidermal growth factor-containing fibulin-like extracellular matrix protein 2 [Source:MGI Symbol;Acc:MGI:1891209]	-
ENSMUSG0000004672	1715.599101	1519.864275	1313.778555	952.6321807	771.3533877	933.131391	1516.413977	885.7056531	0.775735642	2.48E-05	0.000386	Rpl14-ps1	7	45324965	45325617	+	653	processed_pseudog	ribosomal protein L14, pseudogene 1 [Source:MGI Symbol;Acc:MGI:3710579]	-
ENSMUSG0000003461	335.7042633	872.1050728	316.1558938	210.1647849	114.5672125	105.0867468	507.98841	143.2729147	1.824728908	2.49E-05	0.0003875	Pik3ip1	11	3330401	3342971	+	4272	protein_coding	phosphoinositide-3-kinase interacting protein 1 [Source:MGI Symbol;Acc:MGI:1917016]	-
ENSMUSG0000004156	3344.015304	3560.042447	2522.544198	2051.690646	1438.016047	1523.757829	3142.20065	1671.154841	0.9107678	2.50E-05	0.0003885	Nop53	7	15936183	15946074	-	3847	protein_coding	NOP53 ribosome biogenesis factor [Source:MGI Symbol;Acc:MGI:2154441]	-
ENSMUSG0000003797	1846.874499	2527.840791	1489.172461	1036.181296	1040.981396	992.4859425	1954.62925	1023.216212	0.933854679	2.54E-05	0.0003949	Snn	16	11060945	11074985	+	2967	protein_coding	stannin [Source:MGI Symbol;Acc:MGI:1276549]	-
ENSMUSG0000002301	1.002102279	0	4.468634541	78.38112879	15.80237414	26.27168671	1.82357894	40.15172988	-4.491172823	2.55E-05	0.0003957	Aqp2	15	99579055	99584547	+	1419	protein_coding	aquaporin 2 [Source:MGI Symbol;Acc:MGI:1096865]	-
ENSMUSG0000004061	385.8093773	391.8153225	291.5784038	689.9261996	693.3291654	573.1119805	356.4010345	652.1224485	-0.871103945	2.56E-05	0.0003966	Ildr2	1	166254139	166316823	+	8251	protein_coding	immunoglobulin-like domain containing receptor 2 [Source:MGI Symbol;Acc:MGI:1196370]	-
ENSMUSG0000002776	4918.317983	4768.139691	3403.982362	6510.801676	8795.008857	9605.707082	4363.480012	8303.839205	-0.928193821	2.59E-05	0.0004016	Mbnl1	3	60472830	60629750	+	18717	protein_coding	muscleblind like splicing factor 1 [Source:MGI Symbol;Acc:MGI:1928482]	-
ENSMUSG0000002437	938.9698351	713.0617564	782.0110447	1450.481548	1264.189931	1365.154684	811.3475454	1359.942054	-0.745019466	2.61E-05	0.0004042	C2	17	34862604	34898265	-	3872	protein_coding	complement component 2 (within H-2S) [Source:MGI Symbol;Acc:MGI:88226]	-
ENSMUSG0000002506	1552.25643	1582.007028	1235.577451	762.2780107	800.9828392	964.2682049	1456.613636	842.5096849	0.790244348	2.63E-05	0.0004067	Gsto1	19	47854970	47864790	+	1968	protein_coding	glutathione S-transferase omega 1 [Source:MGI Symbol;Acc:MGI:1342273]	-
ENSMUSG0000006426	94.19761419	40.02414585	41.3348695	177.4342036	166.9125769	203.3623157	58.51887651	182.5696987	-1.637888074	2.64E-05	0.0004085	Hvcn1	5	122206804	122242297	+	3563	protein_coding	hydrogen voltage-gated channel 1 [Source:MGI Symbol;Acc:MGI:1921346]	-
ENSMUSG0000004951	8641.127949	7896.34267	8637.870568	5589.177414	3833.063377	5229.038681	8391.780395	4883.759824	0.780907553	2.69E-05	0.0004146	Rps23	13	90922958	90924950	+	1268	protein_coding	ribosomal protein S23 [Source:MGI Symbol;Acc:MGI:1913725]	-
ENSMUSG0000003976	445.935514	354.9509777	342.967701	100.775737	220.2455896	128.4393573	381.2847309	149.8202279	1.349732439	2.71E-05	0.0004179	Dnajc28	16	91614254	91619026	-	3565	protein_coding	DnaJ heat shock protein family (Hsp40) member C28 [Source:MGI Symbol;Acc:MGI:2181053]	-
ENSMUSG0000003277	69.14505722	83.20809269	147.4649398	28.42392582	31.60474828	25.29866128	99.93936326	28.44244513	1.811394946	2.71E-05	0.0004182	Mctp2	7	72077830	72306608	-	12351	protein_coding	multiple C2 domains, transmembrane 2 [Source:MGI Symbol;Acc:MGI:2685335]	-
ENSMUSG0000003237	816.7133571	1128.048953	941.7647295	583.1211449	558.0213368	517.6495308	962.1756798	552.9306708	0.798880776	2.72E-05	0.0004193	Usp3	9	66514637	66593142	-	6848	protein_coding	ubiquitin specific peptidase 3 [Source:MGI Symbol;Acc:MGI:2152450]	-
ENSMUSG0000006050	4.008409114	5.26633498	0	43.06655428	32.59239666	28.21773758	3.091581365	34.62556284	-3.468266325	2.74E-05	0.000422	Xcr1	9	123852315	123862029	-	4453	protein_coding	chemokine (C motif) receptor 1 [Source:MGI Symbol;Acc:MGI:1346338]	-
ENSMUSG0000007153	258.5423879	390.7620556	206.6743475	129.1996628	71.11068363	37.94799192	285.3262637	79.41944613	1.842178533	2.75E-05	0.000423	Gm10335	10	14523078	14523545	+	468	processed_pseudog	predicted gene 10335 [Source:MGI Symbol;Acc:MGI:3641693]	-
ENSMUSG0000007198	400.8409114	264.370016	184.3311748	560.7265367	945.1795032	661.657295	283.1807008	722.5211116	-1.350077124	2.77E-05	0.0004257	Fndc1	17	7738569	7827302	-	9903	protein_coding	fibronectin type III domain containing 1 [Source:MGI Symbol;Acc:MGI:1915905]	-
ENSMUSG0000002671	2548.346095	2050.710841	1857.83481	3095.623922	4535.281378	4459.375563	2152.297249	4030.093621	-0.904716921	2.77E-05	0.0004257	Mrc1	2	14229392	14332057	+	6371	protein_coding	mannose receptor, C type 1 [Source:MGI Symbol;Acc:MGI:97142]	-
ENSMUSG0000002148	1357.848588	1359.767692	1436.666005	2173.138329	2184.678225	1926.590359	1384.760761	2094.802304	-0.597313079	2.82E-05	0.0004325	Prelid1	13	55320500	55325272	+	2726	protein_coding	PRELI domain containing 1 [Source:MGI Symbol;Acc:MGI:1913744]	-
ENSMUSG0000002502	1389.91586	1400.845105	1079.175242	2081.837234	2077.024551	2140.655954	1289.978736	2099.839246	-0.702660727	2.82E-05	0.0004327	Add3	19	53140445	53247399	+	4938	protein_coding	adducin 3 (gamma) [Source:MGI Symbol;Acc:MGI:1351615]	-
ENSMUSG0000001407	15527.57481	12190.51221	13651.67852	5744.21701	8428.591307	8570.408021	13789.92185	7581.072112	0.863207595	2.84E-05	0.000435	Chp1	2	119547697	119587027	+	3499	protein_coding	calcineurin-like EF hand protein 1 [Source:MGI Symbol;Acc:MGI:1927185]	-
ENSMUSG0000002815	488.0238097	488.7158862	902.6641773	274.7646163	289.3809764	166.3873492	626.4679577	243.5109806	1.362554576	2.85E-05	0.0004361	Rorc	3	94372794	94398276	+	3559	protein_coding	RAR-related orphan receptor gamma [Source:MGI Symbol;Acc:MGI:104856]	THR-
ENSMUSG0000002741	816.7133571	894.2236797	990.9197094	593.457118	479.9971145	531.2718868	900.6189154	534.9087064	0.75083756	2.87E-05	0.0004388	Vps16	2	130424339	130444269	+	3727	protein_coding	VSP16 CORVET/HOPS core subunit [Source:MGI Symbol;Acc:MGI:2136772]	-
ENSMUSG0000002871	275.5781266	311.7670308	239.0719479	154.1782643	138.2707737	125.520281	275.4723685	139.3231063	0.982843109	2.88E-05	0.0004397	Tal1	4	115056426	115071755	+	4379	protein_coding	T cell acute lymphocytic leukemia 1 [Source:MGI Symbol;Acc:MGI:98480]	bHLI
ENSMUSG0000004014	1213.545859	1310.264143	739.5590165	2318.703282	1911.099623	2359.586677	1087.789673	2196.463194	-1.013551965	2.91E-05	0.0004433	Maob	X	16709282	16817366	-	2789	protein_coding	monoamine oxidase B [Source:MGI Symbol;Acc:MGI:96916]	-
ENSMUSG0000004832	5.010511393	6.319601977	8.937269082	70.62914902	20.74061606	56.43547516	6.75579415	49.26841341	-2.874403021	2.91E-05	0.0004433	Ckap2l	2	129268210	129297212	-	3137	protein_coding	cytoskeleton associated protein 2-like [Source:MGI Symbol;Acc:MGI:1917716]	-
ENSMUSG0000007375	492.0322188	382.3359196	334.0304319	740.7447336	676.5391429	863.0735588	402.7999254	760.1191454	-0.915388353	2.94E-05	0.0004479	5730409E04Ri	4	126609818	126614371	+	2891	protein_coding	RIKEN cDNA 5730409E04Rik gene [Source:MGI Symbol;Acc:MGI:3609248]	-
ENSMUSG0000003093	1796.769386	3340.962912	1154.02487	79.24245987	428.6393985	234.4991295	2097.252389	247.4603293	3.083666363	2.94E-05	0.0004479	Acsm3	7	119760923	119787513	+	4936	protein_coding	acyl-CoA synthetase medium-chain family member 3 [Source:MGI Symbol;Acc:MGI:99538]	-
ENSMUSG0000003891	17.03573874	25.27840791	101.6614358	3.445324342	4.938241919	3.892101735	47.99186082	4.091889332	3.553377444	2.94E-05	0.000448	3930402G23Ri	8	10924427	10928696	-	3323	lincRNA	RIKEN cDNA 3930402G23 gene [Source:MGI Symbol;Acc:MGI:1921277]	-
ENSMUSG0000003916	1328.787621	1279.7194	1231.108816	2236.876829	1771.8412	2098.815861	1279.871946	2035.84463	-0.669706456	2.97E-05	0.0004515	Dap	15	31224314	31274341	+	1925	protein_coding	death-associated protein [Source:MGI Symbol;Acc:MGI:1918190]	-
ENSMUSG0000002305	2421.079105	2143.398337	2112.546979	1438.422913	1393.571869	1540.299262	2225.674807	1457.431348	0.610941214	2.98E-05	0.0004524	Calcoco1	15	102706777	102722178	-	5731	protein_coding	calcium binding and coiled coil domain 1 [Source:MGI Symbol;Acc:MGI:1914738]	-
ENSMUSG0000003744	741.5556862	327.5660358	2538.184419	63.73850033	85.92540939	137.1965862	1202.43538	95.62016529	3.652751352	3.00E-05	0.0004555	Vnn1	10	23894688	23905343	+	2416	protein_coding	vanin 1 [Source:MGI Symbol;Acc:MGI:108395]	-
ENSMUSG0000003618	62.13034127	47.39701482	24.57748997	125.7543385	168.8878736	128.4393573	44.70161536	141.0271898	-1.652799067	3.01E-05	0.0004566	Fam69b	2	26628457	26636497	+	2476	protein_coding	family with sequence similarity 69, member B [Source:MGI Symbol;Acc:MGI:1927576]	-
ENSMUSG0000008491	14.0294319	32.65127688	42.45202814	0	2.962945151	1.946050868	29.71091231	1.636332006	4.218584605	3.02E-05	0.0004568	Gm12708	4	95193313	95209727	+	1293	lincRNA	predicted gene 12708 [Source:MGI Symbol;Acc:MGI:3652178]	-
ENSMUSG0000002648	1002.102279	1361.874226	1301.48981	681.3128887	731.8474524	737.5532788	1221.822105	716.90454	0.769123303	3.03E-05	0.0004585	Rnf2	1	151458004	151500955	-	4127	protein_coding	ring finger protein 2 [Source:MGI Symbol;Acc:MGI:1101759]	-
ENSMUSG0000004769	0	6.319601977	4.468634541	20.67194605	51.35771595	58.38152603	3.596078839	43.47039601	-3.607384379	3.03E-05	0.000459	Ccdc144b	3	36007244	36053547	-	6768	protein_coding	coiled-coil domain containing 144B [Source:MGI Symbol;Acc:MGI:2681852]	-
ENSMUSG0000009723	138.2901144	114.8061026	111.7158635	33.59191234	54.32066111	49.62429712	121.6040268	45.84562352	1.411871063	3.05E-05	0.0004608	4831440E17Ri	5	25499067	25501541	+	1028	lincRNA	RIKEN cDNA 4831440E17 gene [Source:MGI Symbol;Acc:MGI:2445144]	-
ENSMUSG0000007860	233.4898309	329.6725698	308.3357833	84.41044639	160.9866866	112.8709503	290.4993947	119.4226944	1.28427914	3.10E-05	0.0004675	Gm4070	7	105895139	105953967	-	9275	protein_coding	predicted gene 4070 [Source:MGI Symbol;Acc:MGI:3782245]	-
ENSMUSG0000003308	344.7231838	813.122121	512.7758136	173.1275482	253.8256346	216.9846717	556.8737061	214.6459515	1.375911956	3.10E-05	0.0004675	Clec1a	6	129424772	129452000	-	5180	protein_coding	C-type lectin domain family 1, member a [Source:MGI Symbol;Acc:MGI:2444151]	-
ENSMUSG0000002871	2.004204557	1.053266996	2.23431727	37.89856777	12.83942899	36.00194105	1.763929608	28.9133126	-4.039304341	3.13E-05	0.000472	Stil	4	115000159	115043196	+	5795	protein_coding	Scl/Tal1 interrupting locus [Source:MGI Symbol;Acc:MGI:107477]	-
ENSMUSG0000003869	351.7378998	362.3238467	520.595924	219.6394268	209.3814574	167.3603746	411.5525568	198.7937529	1.04859896	3.16E-05	0.0004754	Hoxb4	11	96318267	96321638	+	2566	protein_coding	homeobox B4 [Source:MGI Symbol;Acc:MGI:96185]	Hom
ENSMUSG0000002644	48.10090937	30.54474289	32.39760042	87.85577073	123.456048	110.9248995	37.01441756	107.4122394	-1.533030259	3.16E-05	0.0004757	Lrrn2	1	132880273	132940005	+	3595	protein_coding	leucine rich repeat protein 2, neuronal [Source:MGI Symbol;Acc:MGI:106037]	-
ENSMUSG0000008280	13.02732962	3.159800988	23.46033134	73.21314228	63.20949656	90.49136534	13.21582065	75.63800139	-2.522881933	3.19E-05	0.0004791	Gm14150	2	151458956	151460309	+	1354	processed_pseudog	predicted gene 14150 [Source:MGI Symbol;Acc:MGI:3651006]	-
ENSMUSG0000004099	1664.491885	2013.846497	1936.035915	1189.498229	1210.856918	1224.065996	1871.458099	1208.140381	0.631293867	3.21E-05	0.0004817	Abhd4	14	54254188	54270637	+	3279	protein_coding	abhydrolase domain containing 4 [Source:MGI Symbol;Acc:MGI:1915938]	-
ENSMUSG0000003923	93.19551191	44.23721384	34.63191769	352.284414	151.1102027	165.4143237	57.35488115	222.9363135	-1.956752226	3.22E-05	0.0004836	Tgfb2	1	186622792	186705989	-	5791	protein_coding	transforming growth factor, beta 2 [Source:MGI Symbol;Acc:MGI:98726]	-
ENSMUSG0000002550	8375.570845	7597.214843	9662.305036	4571.084071	4656.762129	5970.484062	8545.030241	5066.110087	0.754223582	3.24E-05	0.0004867	Rplp2	7	141447370	141451585	+	2072	protein_coding	ribosomal protein, large P2 [Source:MGI Symbol;Acc:MGI:1914436]	-
ENSMUSG0000010524	153.3216486	141.1377775	129.5904017	47.37320971	72.09833201	56.43547516	141.3499426	58.63567229	1.272790469	3.30E-05	0.0004947	Gm31305	3	97730339	97736166	+	1374	antisense	predicted gene, 31305 [Source:MGI Symbol;Acc:MGI:5590464]	-
ENSMUSG0000003594	2420.077003	2201.328022	2133.772993	1267.879358	1476.534334	1547.11044	2251.726006	1430.508044	0.654835485	3.30E-05	0.0004947	Fbxw2	2	34804514	34826311	-	9501	protein_coding	F-box and WD-40 domain protein 2 [Source:MGI Symbol;Acc:MGI:1353435]	-
ENSMUSG0000009026	53.11142077	41.07741285	187.6826507	20.67194605	6.913538686	11.67630521	93.95716144	13.08726332	2.835216726	3.31E-05	0.0004961	Eif4ebp3	18	36664060	36665917	+	744	protein_coding	eukaryotic translation initiation factor 4E binding protein 3 [Source:MGI Symbol;Acc:MGI:1270847]	-
ENSMUSG0000002639	708.486311	727.8074943	698.224147	987.9467552	1308.634108	1403.102676	711.5059841	1233.227846	-0.793119461	3.33E-05	0.0004975	Ptprc	1	138062861	138175708	-	18313	protein_coding	protein tyrosine phosphatase, receptor type, C [Source:MGI Symbol;Acc:MGI:97810]	-
ENSMUSG0000006847	567.1898897	607.7350567	630.0774703	374.6790222	382.2199245	363.9115122	601.6674722	373.6034863	0.687213587	3.33E-05	0.0004975	Mfap1a	2	121491911	121506665	-	4198	protein_coding	microfibrillar-associated protein 1A [Source:MGI Symbol;Acc:MGI:1914782]	-
ENSMUSG0000009434	14.0294319	2.106533992	3.351475906	14.64262846	151.1102027	82.70716187	6.495813933	82.81999768	-3.660708515	3.34E-05	0.0004988	Igkv14-126	6	67896172	67896642	+	358	IG_V_gene	immunoglobulin kappa variable 14-126 [Source:MGI Symbol;Acc:MGI:3643131]	-
ENSMUSG0000002416	2144.498876	2151.824473	3243.111518	1267.879358	1513.077324	1241.580454	2513.144956	1340.845712	0.906287417	3.34E-05	0.0004988	Mapk8ip3	17	24892153	24936977	-	14558	protein_coding	mitogen-activated protein kinase 8 interacting protein 3 [Source:MGI Symbol;Acc:MGI:1353598]	-
ENSMUSG0000003078	493.0343211	397.0816575	535.1189863	853.5791058	892.8341389	745.3374823	475.0783216	830.5835757	-0.806225989	3.35E-05	0.0004997	Tgfb1i1	7	128246812	128255699	+	5585	protein_coding	transforming growth factor beta 1 induced transcript 1 [Source:MGI Symbol;Acc:MGI:102784]	-
ENSMUSG0000004173	500.049037	308.6072299	1257.920623	3403.98045	4120.469057	5630.898185	688.8589634	4385.115898	-2.670482129	3.36E-05	0.0005008	Tmem45b	9	31426196	31464462	-	4724	protein_coding	transmembrane protein 45b [Source:MGI Symbol;Acc:MGI:2384574]	-
ENSMUSG0000003251	18.03784102	23.17187391	7.820110447	78.38112879	62.22184818	65.19270406	16.34327513	68.59856034	-2.064804928	3.40E-05	0.0005063	Scn5a	9	119483408	119579016	-	8764	protein_coding	sodium channel, voltage-gated, type V, alpha [Source:MGI Symbol;Acc:MGI:98251]	-
ENSMUSG0000003281	21.04414785	8.426135969	13.40590362	134.3676494	44.44417727	59.35455146	14.29206248	79.38879269	-2.472655273	3.42E-05	0.0005084	Igdcc4	9	65101486	65137940	+	9481	protein_coding	immunoglobulin superfamily, DCC subclass, member 4 [Source:MGI Symbol;Acc:MGI:1858497]	-
ENSMUSG0000000512	2970.231154	4877.679459	2874.449168	2087.00522	1422.213673	1602.572889	3574.119927	1703.930594	1.068570247	3.44E-05	0.0005112	Ndrg1	15	66929318	66969640	-	6596	protein_coding	N-myc downstream regulated gene 1 [Source:MGI Symbol;Acc:MGI:1341799]	-
ENSMUSG0000011635	2.004204557	1.053266996	1.117158635	24.1172704	21.72826444	26.27168671	1.391543396	24.03907385	-4.099305361	3.46E-05	0.0005138	Gm49450	15	98932137	98953551	-	899	protein_coding	predicted gene, 49450 [Source:MGI Symbol;Acc:MGI:6155101]	-
ENSMUSG0000006617	27.05676152	86.36789368	164.2223194	12.91996628	5.925890302	19.46050868	92.54899153	12.76878842	2.855257307	3.53E-05	0.0005241	E230001N04Ri	17	28522720	28525398	+	2679	lincRNA	RIKEN cDNA E230001N04 gene [Source:MGI Symbol;Acc:MGI:2443549]	-
ENSMUSG0000003030	287.603354	305.4474289	331.7961147	173.9888793	167.9002252	175.1445781	308.2822992	172.3445609	0.838371563	3.54E-05	0.0005245	Caprin2	6	148842492	148896237	-	14140	protein_coding	caprin family member 2 [Source:MGI Symbol;Acc:MGI:2448541]	-
ENSMUSG0000003245	65.13664811	18.95880593	50.27213858	111.11171	208.393809	192.6590359	44.78919754	170.7215183	-1.928602707	3.56E-05	0.0005276	Clstn2	9	97444395	98033181	-	5691	protein_coding	calsyntenin 2 [Source:MGI Symbol;Acc:MGI:1929897]	-
ENSMUSG0000005256	642.3475606	787.8437131	1193.125422	372.9563601	458.2688501	407.6976568	874.4388987	412.974289	1.082180658	3.59E-05	0.0005316	Cpne8	15	90487482	90679432	-	4164	protein_coding	copine VIII [Source:MGI Symbol;Acc:MGI:1914121]	-
ENSMUSG0000003923	660.3854016	608.7883237	547.4077313	1011.202694	894.8094357	1092.707562	605.5271522	999.5732308	-0.722803501	3.64E-05	0.0005383	Tbcd	11	121451949	121617164	+	8096	protein_coding	tubulin-specific chaperone d [Source:MGI Symbol;Acc:MGI:1919686]	-
ENSMUSG0000003244	5670.896795	9710.068437	6782.270074	3466.85762	4337.751701	3724.741361	7387.745102	3843.116894	0.942887918	3.66E-05	0.0005402	Tgfbr2	9	116084293	116175360	-	8187	protein_coding	transforming growth factor, beta receptor II [Source:MGI Symbol;Acc:MGI:98729]	-
ENSMUSG0000002973	934.961426	939.5141605	902.6641773	615.8517262	587.6507883	617.8711504	925.7132546	607.124255	0.608539468	3.66E-05	0.0005402	Mcm7	5	138164583	138172422	-	4426	protein_coding	minichromosome maintenance complex component 7 [Source:MGI Symbol;Acc:MGI:1298398]	-
ENSMUSG0000000094	35.07357975	52.6633498	46.92066268	111.11171	119.5054544	111.8979249	44.88586408	114.1716965	-1.348450939	3.68E-05	0.0005427	Hoxa4	6	52189671	52191753	-	1594	protein_coding	homeobox A4 [Source:MGI Symbol;Acc:MGI:96176]	Hom
ENSMUSG0000003818	328.6895474	319.1398998	292.6955624	525.4119622	492.8365435	531.2718868	313.5083265	516.5067975	-0.719945538	3.71E-05	0.0005476	Btbd10	7	113315626	113369392	-	5801	protein_coding	BTB (POZ) domain containing 10 [Source:MGI Symbol;Acc:MGI:1916065]	-
ENSMUSG0000004787	395.8304001	379.1761186	428.9889159	254.0926702	189.6284897	161.522222	401.3318115	201.747794	0.990415047	3.73E-05	0.0005493	A4galt	15	83226722	83251774	-	2278	protein_coding	alpha 1,4-galactosyltransferase [Source:MGI Symbol;Acc:MGI:3512453]	-
ENSMUSG0000003703	1534.218589	1172.286167	2223.145684	521.1053068	906.6612163	726.849999	1643.216813	718.2055074	1.194307314	3.79E-05	0.0005583	Inhbb	1	119415465	119422248	-	4255	protein_coding	inhibin beta-B [Source:MGI Symbol;Acc:MGI:96571]	-
ENSMUSG0000005805	718.5073338	419.2002644	509.4243377	974.1654578	1355.053582	1051.840494	549.0439786	1127.019845	-1.03696122	3.81E-05	0.0005595	Palld	8	61511433	61902690	-	10292	protein_coding	palladin, cytoskeletal associated protein [Source:MGI Symbol;Acc:MGI:1919583]	-
ENSMUSG0000003008	100.2102279	95.84729665	75.96678719	248.0633527	178.7643575	181.9557561	90.67477057	202.9278221	-1.162000463	3.81E-05	0.0005595	Slc41a3	6	90604725	90646412	+	2911	protein_coding	solute carrier family 41, member 3 [Source:MGI Symbol;Acc:MGI:1918949]	-
ENSMUSG0000005690	15.03153418	14.74573795	17.87453816	49.95720296	58.27125464	66.1657295	15.88393676	58.1313957	-1.872543384	3.84E-05	0.0005637	Usp13	3	32817546	32938071	+	7599	protein_coding	ubiquitin specific peptidase 13 (isopeptidase T-3) [Source:MGI Symbol;Acc:MGI:1919857]	-
ENSMUSG0000002837	31.06517064	27.3849419	29.04612452	99.05307484	68.14773848	90.49136534	29.16541235	85.89739289	-1.559082432	3.85E-05	0.0005652	Pappa	4	65124174	65357509	+	11027	protein_coding	pregnancy-associated plasma protein A [Source:MGI Symbol;Acc:MGI:97479]	-
ENSMUSG0000007861	8.016818229	16.85227194	26.81180725	158.4849197	44.44417727	86.59926361	17.2269658	96.50945354	-2.493739887	3.86E-05	0.0005654	Fyb2	4	104913456	105016863	+	3136	protein_coding	FYN binding protein 2 [Source:MGI Symbol;Acc:MGI:2685466]	-
ENSMUSG0000002851	5562.669749	4068.770406	4515.555204	8987.989878	7164.401376	7724.848919	4715.665119	7959.080058	-0.755130365	3.88E-05	0.0005681	Plpp3	4	105157347	105232764	+	3159	protein_coding	phospholipid phosphatase 3 [Source:MGI Symbol;Acc:MGI:1915166]	-
ENSMUSG0000005999	1.002102279	0	0	17.22662171	17.77767091	32.10983931	0.334034093	22.37137731	-5.884230138	3.89E-05	0.0005694	Nptx2	5	144545902	144557478	+	2536	protein_coding	neuronal pentraxin 2 [Source:MGI Symbol;Acc:MGI:1858209]	-
ENSMUSG0000002419	984.0644376	964.7925684	1028.903103	683.0355509	639.0085043	595.4915655	992.586703	639.1785402	0.634561019	3.89E-05	0.0005695	Phf1	17	26933127	26937890	+	2429	protein_coding	PHD finger protein 1 [Source:MGI Symbol;Acc:MGI:98647]	-
ENSMUSG0000003525	936.9656305	923.7151556	506.0728618	1385.020386	1768.878255	1954.808096	788.9178826	1702.902246	-1.109557852	3.90E-05	0.0005699	Abi3bp	16	56477878	56690135	+	7416	protein_coding	ABI gene family, member 3 (NESH) binding protein [Source:MGI Symbol;Acc:MGI:2444583]	-
ENSMUSG0000006727	245.5150583	135.8714425	98.3099599	907.8429642	274.5662507	528.3528105	159.8988202	570.2540085	-1.833814994	3.90E-05	0.0005699	Ppp1r3c	19	36731737	36736653	-	2719	protein_coding	protein phosphatase 1, regulatory subunit 3C [Source:MGI Symbol;Acc:MGI:1858229]	-
ENSMUSG0000005095	18.03784102	11.58593696	23.46033134	86.99443964	50.37006757	72.0038821	17.6947031	69.78946311	-1.983980625	3.90E-05	0.0005699	Insl6	19	29321344	29325356	-	708	protein_coding	insulin-like 6 [Source:MGI Symbol;Acc:MGI:1351595]	-
ENSMUSG0000004562	8.016818229	3.159800988	18.9916968	44.78921645	90.8636513	57.40850059	10.05610534	64.35378945	-2.685891694	3.93E-05	0.0005724	Odf3l1	9	56848659	56864679	-	1306	protein_coding	outer dense fiber of sperm tails 3-like 1 [Source:MGI Symbol;Acc:MGI:2681875]	-
ENSMUSG0000003651	235.4940355	213.8132002	227.9003616	391.0443129	369.3804955	392.1292498	225.7358658	384.1846861	-0.767166026	3.97E-05	0.0005783	Commd2	3	57644349	57657715	-	3312	protein_coding	COMM domain containing 2 [Source:MGI Symbol;Acc:MGI:1098806]	-
ENSMUSG0000004720	206.4330694	174.8423214	113.9501808	442.724178	314.072186	361.9654614	165.0751905	372.9206085	-1.174714644	3.98E-05	0.0005801	Dusp18	11	3895240	3901296	+	4632	protein_coding	dual specificity phosphatase 18 [Source:MGI Symbol;Acc:MGI:1922469]	-
ENSMUSG0000002478	3351.03002	3555.829379	3422.974058	4602.09199	5833.051354	6157.304945	3443.277819	5530.816097	-0.68363135	3.99E-05	0.0005807	Ak3	19	29020833	29047961	-	3120	protein_coding	adenylate kinase 3 [Source:MGI Symbol;Acc:MGI:1860835]	-
ENSMUSG0000006058	2320.868877	1083.811739	842.337611	4981.077668	2953.068667	3775.338683	1415.672742	3903.161673	-1.46298551	4.04E-05	0.0005867	H2-Eb1	17	34305877	34316199	+	1184	protein_coding	histocompatibility 2, class II antigen E beta [Source:MGI Symbol;Acc:MGI:95901]	-
ENSMUSG0000006721	2747.764448	3305.151834	2106.961186	1568.483907	1599.990382	1371.965862	2719.959156	1513.48005	0.845768136	4.05E-05	0.0005889	H2-T23	17	36029773	36032855	-	2889	protein_coding	histocompatibility 2, T region locus 23 [Source:MGI Symbol;Acc:MGI:95957]	-

ENSMUSG0000002882	2043.286546	1857.962981	2207.505463	1372.100419	1314.559999	1339.856022	2036.251663	1342.172147	0.601201979	4.07E-05	0.0005904	Maco1	4	134802759	134853345	-	6744	protein_coding	macoilin 1 [Source:MGI Symbol;Acc:MGI:1913396]	-
ENSMUSG0000003101	1026.152733	1063.799666	1072.47229	647.7209764	613.3296463	742.418406	1054.141563	667.8230095	0.658553366	4.11E-05	0.0005962	Swap70	7	110221711	110283506	+	8090	protein_coding	SWA-70 protein [Source:MGI Symbol;Acc:MGI:1298390]	-
ENSMUSG0000002301	4179.768604	6052.07216	5893.011801	7464.295188	12614.24516	14220.76671	5374.950855	11433.10235	-1.088891278	4.12E-05	0.0005967	Gpd1	15	99717515	99725005	+	2961	protein_coding	glycerol-3-phosphate dehydrogenase 1 (soluble) [Source:MGI Symbol;Acc:MGI:95679]	-
ENSMUSG0000001892	3432.200304	3938.165298	4902.092091	1521.972028	2461.219772	2310.935405	4090.819231	2098.042402	0.963474212	4.23E-05	0.0006117	Ccl6	11	83587882	83593087	-	1795	protein_coding	chemokine (C-C motif) ligand 6 [Source:MGI Symbol;Acc:MGI:98263]	-
ENSMUSG0000008134	138.2901144	117.9659036	161.9880021	316.1085084	250.8626895	267.5819943	139.4146734	278.1843974	-0.998187901	4.23E-05	0.0006117	Gm14303	2	172509521	172509691	-	171	processed_pseudog	predicted gene 14303 [Source:MGI Symbol;Acc:MGI:3651379]	-
ENSMUSG0000004751	77.16187545	26.3316749	89.37269082	12.0586352	9.876483837	13.62235607	64.28874706	11.8524917	2.438273957	4.24E-05	0.0006117	Olfr1396	11	49109982	49114874	-	4247	protein_coding	olfactory receptor 1396 [Source:MGI Symbol;Acc:MGI:3031230]	-
ENSMUSG0000001620	62.13034127	70.56888874	61.44372494	118.8636898	160.9866866	200.4432394	64.71431832	160.0978719	-1.305410057	4.24E-05	0.0006117	Syt14	1	192891233	193035775	-	11932	protein_coding	synaptotagmin XIV [Source:MGI Symbol;Acc:MGI:2444490]	-
ENSMUSG0000003925	3.006306836	0	3.351475906	34.45324342	29.62945151	21.40655954	2.119260914	28.49641816	-3.753939331	4.24E-05	0.0006117	Vstm2b	7	40898328	40930594	+	2747	protein_coding	V-set and transmembrane domain containing 2B [Source:MGI Symbol;Acc:MGI:1914525]	-
ENSMUSG0000006031	2.004204557	0	5.585793176	31.86925017	27.65415474	34.05589018	2.529999244	31.19309836	-3.645901329	4.26E-05	0.0006149	Mucl1	15	103751919	103755469	-	797	protein_coding	mucin-like 1 [Source:MGI Symbol;Acc:MGI:98393]	-
ENSMUSG0000002825	304.6390927	265.423283	389.8883637	530.5799487	616.2925915	635.3856083	319.9835798	594.0860495	-0.893070486	4.27E-05	0.0006161	Ccnc	4	21727701	21759922	+	5115	protein_coding	cyclin C [Source:MGI Symbol;Acc:MGI:1858199]	-
ENSMUSG0000002303	76.15977317	121.1257046	116.1844981	291.1299069	205.4308638	224.7688752	104.4899919	240.4432153	-1.204680311	4.28E-05	0.0006165	Cela1	15	100674421	100687921	-	2306	protein_coding	chymotrypsin-like elastase family, member 1 [Source:MGI Symbol;Acc:MGI:95314]	-
ENSMUSG0000005829	989.074949	1073.279069	956.2877917	608.9610775	625.1814269	692.7941089	1006.213937	642.3122044	0.647874102	4.30E-05	0.0006185	Zfp68	5	138603652	138619761	-	7962	protein_coding	zinc finger protein 68 [Source:MGI Symbol;Acc:MGI:1344427]	zf-C2
ENSMUSG0000002175	563.1814806	875.2648738	672.5294984	393.6283061	370.3681439	368.7766394	703.6586176	377.5910298	0.89772078	4.34E-05	0.0006246	Kctd6	14	8214140	8223563	+	3257	protein_coding	potassium channel tetramerisation domain containing 6 [Source:MGI Symbol;Acc:MGI:1918643]	-
ENSMUSG0000003161	518.086878	492.9289542	466.9723095	715.7661321	839.5011262	826.0985933	492.6627139	793.7886172	-0.687514701	4.35E-05	0.0006253	Tmem184c	8	77595982	77610698	-	2723	protein_coding	transmembrane protein 184C [Source:MGI Symbol;Acc:MGI:2384562]	-
ENSMUSG0000007935	44.09250026	18.95880593	39.10055223	260.9833189	79.99951908	110.9248995	34.05061947	150.6359125	-2.146443356	4.36E-05	0.0006259	Ackr4	9	104084157	104126933	-	5380	protein_coding	atypical chemokine receptor 4 [Source:MGI Symbol;Acc:MGI:2181676]	-
ENSMUSG0000003852	1568.290066	1513.544673	1613.177069	1030.151978	1056.783771	1093.680588	1565.003936	1060.205446	0.561883588	4.37E-05	0.0006271	Tbc1d17	7	44834623	44849079	-	9726	protein_coding	TBC1 domain family, member 17 [Source:MGI Symbol;Acc:MGI:2449973]	-
ENSMUSG0000005631	2341.913025	923.7151556	1994.128164	755.3873621	696.2921105	730.7421008	1753.252115	727.4738578	1.26905238	4.42E-05	0.000634	Tcim	8	24437180	24438984	-	1805	protein_coding	transcriptional and immune response regulator [Source:MGI Symbol;Acc:MGI:1916318]	-
ENSMUSG0000000963	958.0097784	531.899833	2315.869851	9480.671259	6064.161076	7649.92596	1268.593154	7731.586099	-2.607626061	4.44E-05	0.0006362	G0s2	1	193272161	193273217	-	871	protein_coding	G0/G1 switch gene 2 [Source:MGI Symbol;Acc:MGI:1316737]	-
ENSMUSG0000003245	248.5213651	243.3046761	292.6955624	403.1029481	533.3301272	487.4857423	261.5072012	474.6396059	-0.85993442	4.46E-05	0.000638	Mrps22	9	98588730	98601660	-	3991	protein_coding	mitochondrial ribosomal protein S22 [Source:MGI Symbol;Acc:MGI:1928137]	-
ENSMUSG0000005986	631.3244355	621.4275277	913.8357636	339.3644477	432.5899921	352.235207	722.1959089	374.7298823	0.946550741	4.53E-05	0.0006478	Tnip2	5	34496087	34513991	-	2685	protein_coding	TNFAIP3 interacting protein 2 [Source:MGI Symbol;Acc:MGI:2386643]	-
ENSMUSG0000003354	11.02312506	11.58593696	7.820110447	57.70918273	39.50593535	42.81311909	10.14305749	46.67607906	-2.200415165	4.55E-05	0.0006502	Angptl1	1	156838562	156861078	+	2841	protein_coding	angiopoietin-like 1 [Source:MGI Symbol;Acc:MGI:1919963]	-
ENSMUSG0000007901	5354.232475	5724.506124	7053.739623	4170.565116	2985.661064	3184.712245	6044.159407	3446.979475	0.810038066	4.56E-05	0.0006513	Ly6c1	15	75044018	75048830	-	1677	protein_coding	lymphocyte antigen 6 complex, locus C1 [Source:MGI Symbol;Acc:MGI:96882]	-
ENSMUSG0000002230	187.3931261	183.2684573	148.5820985	299.7432178	311.1092409	376.5608429	173.0812273	329.1377672	-0.925916684	4.57E-05	0.0006529	Zfpm2	15	40655035	41104592	+	9536	protein_coding	zinc finger protein, multitype 2 [Source:MGI Symbol;Acc:MGI:1334444]	zf-C2
ENSMUSG0000000262	1428.997849	1290.25207	1618.762862	926.7922481	930.3647775	753.1216857	1446.004261	870.0929038	0.732563762	4.58E-05	0.0006536	Akap8l	17	32321425	32350581	-	2075	protein_coding	A kinase (PRKA) anchor protein 8-like [Source:MGI Symbol;Acc:MGI:1860606]	-
ENSMUSG0000003723	155.3258532	172.7357874	157.5193676	85.27177747	70.12303525	74.9229584	161.860336	76.77259037	1.074438421	4.60E-05	0.000656	Spred3	7	29158829	29170411	-	6218	protein_coding	sprouty-related, EVH1 domain containing 3 [Source:MGI Symbol;Acc:MGI:2142186]	-
ENSMUSG0000003368	5373.272418	5018.817236	9832.113149	3490.97489	2890.846819	3098.112981	6741.400934	3159.97823	1.093040754	4.64E-05	0.0006606	Ucp2	7	100493337	100502020	+	6989	protein_coding	uncoupling protein 2 (mitochondrial, proton carrier) [Source:MGI Symbol;Acc:MGI:109354]	-
ENSMUSG0000005448	338.7105702	400.2414585	507.1900204	200.6901429	239.9985573	193.6320613	415.380683	211.4402538	0.973919763	4.64E-05	0.0006607	Tmem62	2	120977017	121007852	+	3348	protein_coding	transmembrane protein 62 [Source:MGI Symbol;Acc:MGI:2139461]	-
ENSMUSG0000003120	296.6222745	381.2826526	366.4280324	196.3834875	195.55438	180.0097052	348.1109865	190.6491909	0.867924866	4.65E-05	0.0006612	Mtcp1	X	75410444	75416588	-	4478	protein_coding	mature T cell proliferation 1 [Source:MGI Symbol;Acc:MGI:102699]	-
ENSMUSG0000006290	4594.638947	5438.017501	3668.748958	1755.392752	2775.291958	2557.11084	4567.135135	2362.598517	0.951110732	4.66E-05	0.0006622	Klhl24	16	20097542	20129221	+	10183	protein_coding	kelch-like 24 [Source:MGI Symbol;Acc:MGI:1923035]	-
ENSMUSG0000002298	11395.90711	8414.550032	9675.71094	5943.184491	6047.371054	6469.646109	9828.722695	6153.400551	0.675656459	4.66E-05	0.0006622	Sod1	16	90220754	90226329	+	3970	protein_coding	superoxide dismutase 1, soluble [Source:MGI Symbol;Acc:MGI:98351]	-
ENSMUSG0000000669	5442.417475	4797.631167	5723.203688	7385.052728	9180.191727	8437.103536	5321.08411	8334.115997	-0.647282142	4.73E-05	0.0006703	Cdc42	4	137319696	137357720	-	2966	protein_coding	cell division cycle 42 [Source:MGI Symbol;Acc:MGI:106211]	-
ENSMUSG0000002581	425.8934684	378.1228516	446.8634541	630.4943547	866.1676325	706.4164649	416.9599247	734.359484	-0.816286728	4.73E-05	0.0006703	Nudt5	2	5845019	5871895	+	6484	protein_coding	nudix (nucleoside diphosphate linked moiety X)-type motif 5 [Source:MGI Symbol;Acc:MGI:1858232]	-
ENSMUSG0000002895	1240.602621	1331.329483	1509.281316	863.0537478	818.7605101	912.6978569	1360.404473	864.8373716	0.653298296	4.77E-05	0.0006761	Nub1	5	24685532	24710378	+	4319	protein_coding	negative regulator of ubiquitin-like proteins 1 [Source:MGI Symbol;Acc:MGI:1889001]	-
ENSMUSG0000006980	298.626479	225.3991372	206.6743475	533.163942	399.9975954	490.4048186	243.5666546	474.5221187	-0.961566016	4.81E-05	0.0006812	Cacng7	7	3332955	3368221	+	2441	protein_coding	calcium channel, voltage-dependent, gamma subunit 7 [Source:MGI Symbol;Acc:MGI:1932374]	-
ENSMUSG0000002077	17543.80459	15536.74146	26983.84968	8210.207908	10947.09469	11069.13733	20021.46524	10075.47998	0.990706933	4.84E-05	0.0006843	Acox1	11	116171888	116199045	-	5082	protein_coding	acyl-Coenzyme A oxidase 1, palmitoyl [Source:MGI Symbol;Acc:MGI:1330812]	-
ENSMUSG0000001066	440.9250026	502.4083571	490.4326409	743.3287269	732.8351007	892.2643228	477.9220002	789.4760501	-0.724213444	4.86E-05	0.0006874	Plcd1	9	119071527	119093502	-	3093	protein_coding	phospholipase C, delta 1 [Source:MGI Symbol;Acc:MGI:97614]	-
ENSMUSG0000002177	1461.065122	1701.026199	681.4667675	621.8810438	232.0973702	409.6437076	1281.186029	421.2073739	1.604397711	4.88E-05	0.0006888	Nr1d2	14	18204054	18239127	-	5409	protein_coding	nuclear receptor subfamily 1, group D, member 2 [Source:MGI Symbol;Acc:MGI:2449205]	THR-
ENSMUSG0000002957	532.1163099	512.9410271	259.1808034	1138.679695	920.4882937	879.6149921	434.7460468	979.594327	-1.171491682	4.90E-05	0.0006908	Lfng	5	140607320	140615545	+	2505	protein_coding	LFNG O-fucosylpeptide 3-beta-N-acetylglucosaminyltransferase [Source:MGI Symbol;Acc:MGI:1095413]	-
ENSMUSG0000005728	456.958639	608.7883237	938.4132536	355.7297383	214.3196993	152.7649931	668.0534055	240.9381436	1.469832321	4.97E-05	0.0007009	St6galnac2	11	116675303	116694868	-	5790	protein_coding	ST6 (alpha-N-acetyl-neuraminyl-2,3-beta-galactosyl-1,3)-N-acetylgalactosaminide alpha-2,6-sialyltransferase 2 [Source:MGI Sym	-
ENSMUSG0000005670	1510.168134	1094.344409	1127.213063	794.1472609	668.6379558	657.7651932	1243.908535	706.8501366	0.815292253	4.98E-05	0.0007012	Ier5	1	155096361	155099636	-	3276	protein_coding	immediate early response 5 [Source:MGI Symbol;Acc:MGI:1337072]	-
ENSMUSG0000002226	941.9761419	717.2748243	665.8265466	1178.300925	1637.52102	1445.915795	775.0258376	1420.579247	-0.87357742	4.99E-05	0.0007024	Sdc2	15	32920723	33034935	+	3527	protein_coding	syndecan 2 [Source:MGI Symbol;Acc:MGI:1349165]	-
ENSMUSG0000002030	87.18289824	75.83522372	88.25553218	151.5942711	192.5914348	187.7939087	83.75788471	177.3265382	-1.08124374	5.02E-05	0.0007065	Chac2	11	30976707	30986350	-	1315	protein_coding	ChaC, cation transport regulator 2 [Source:MGI Symbol;Acc:MGI:1915294]	-
ENSMUSG0000000646	101.2123301	86.36789368	89.37269082	28.42392582	30.6170999	41.84009365	92.31763821	33.62703979	1.459700197	5.04E-05	0.0007079	A530013C23R	2	167691177	167729172	+	5964	lincRNA	RIKEN cDNA A530013C23 gene [Source:MGI Symbol;Acc:MGI:3041178]	-
ENSMUSG0000006003	14415.24128	15315.55539	10631.99873	8900.995438	6926.378115	7280.176296	13454.26513	7702.516616	0.804641563	5.05E-05	0.0007091	Rpl3	15	80077791	80091868	-	3175	protein_coding	ribosomal protein L3 [Source:MGI Symbol;Acc:MGI:1351605]	-
ENSMUSG0000006200	3769.908772	3550.563044	3937.984189	2517.670763	1684.928143	2335.261041	3752.818668	2179.286649	0.783935844	5.07E-05	0.0007115	Rpl34	3	130726831	130730398	-	2643	protein_coding	ribosomal protein L34 [Source:MGI Symbol;Acc:MGI:1915686]	-
ENSMUSG0000002070	575.2067079	708.8486884	1127.213063	348.8390897	397.0346503	376.5608429	803.7561531	374.1448609	1.102905622	5.07E-05	0.0007115	Adap2	11	80154105	80178958	+	1785	protein_coding	ArfGAP with dual PH domains 2 [Source:MGI Symbol;Acc:MGI:2663075]	-
ENSMUSG0000008994	156.3279555	72.67542273	176.5110644	19.81061497	50.37006757	36.00194105	135.1714809	35.39420786	1.937302251	5.10E-05	0.0007146	Gm16168	17	10496446	10569396	+	1691	antisense	predicted gene 16168 [Source:MGI Symbol;Acc:MGI:3802010]	-
ENSMUSG0000006158	802.6839252	752.0326352	851.27488	1181.746249	1646.409856	1276.609369	801.9971468	1368.255158	-0.770507893	5.11E-05	0.0007156	Dot1l	10	80755206	80795461	+	8705	protein_coding	DOT1-like, histone H3 methyltransferase (S. cerevisiae) [Source:MGI Symbol;Acc:MGI:2143886]	-
ENSMUSG0000007040	149.3132395	294.9147589	368.6623496	77.5197977	97.77718999	113.8439758	270.9634493	96.38032115	1.491525221	5.11E-05	0.0007156	Hs3st3b1	11	63885792	63922290	-	4911	protein_coding	heparan sulfate (glucosamine) 3-O-sulfotransferase 3B1 [Source:MGI Symbol;Acc:MGI:1333853]	-
ENSMUSG0000002141	1904.996432	1908.519797	2100.258234	1282.521986	1298.757625	1395.318472	1971.258154	1325.532694	0.572527474	5.13E-05	0.0007174	Eci2	13	34977748	35027096	-	5540	protein_coding	enoyl-Coenzyme A delta isomerase 2 [Source:MGI Symbol;Acc:MGI:1346064]	-
ENSMUSG0000011075	0	0	0	7.75197977	17.77767091	22.37958498	0	15.96974522	-6.358480202	5.15E-05	0.0007195	BC049987	9	44670453	44672238	-	1454	lincRNA	cDNA sequence BC049987 [Source:MGI Symbol;Acc:MGI:3039606]	-
ENSMUSG0000002222	2046.292853	2001.207293	2962.704701	1169.687614	1467.645498	1197.794309	2336.734949	1278.375807	0.870167396	5.20E-05	0.0007261	Zscan26	13	21442180	21453730	-	4366	protein_coding	zinc finger and SCAN domain containing 26 [Source:MGI Symbol;Acc:MGI:3531417]	zf-C2
ENSMUSG0000001009	3431.198202	4104.581484	5009.33932	2653.761075	2267.640689	2466.619475	4181.706335	2462.673746	0.763693469	5.22E-05	0.0007285	Slc3a2	19	8706882	8723369	-	3529	protein_coding	solute carrier family 3 (activators of dibasic and neutral amino acid transport), member 2 [Source:MGI Symbol;Acc:MGI:96955]	-
ENSMUSG0000007105	289.6075585	307.5539629	374.2481428	194.6608253	111.6042674	101.1946451	323.8032214	135.8199126	1.250527211	5.28E-05	0.0007365	Rpl7a-ps5	17	57838430	57839221	-	792	processed_pseudog	ribosomal protein L7A, pseudogene 5 [Source:MGI Symbol;Acc:MGI:3643553]	-
ENSMUSG0000005475	138.2901144	122.1789715	182.0968575	267.0126365	328.8869118	295.7997319	147.5219812	297.2330934	-1.011430891	5.33E-05	0.0007433	Fsd1l	4	53631471	53707009	+	9739	protein_coding	fibronectin type III and SPRY domain containing 1-like [Source:MGI Symbol;Acc:MGI:2442443]	-
ENSMUSG0000002863	10631.30307	9165.5294	11418.47841	7332.511532	6250.826621	6493.971745	10405.10363	6692.436632	0.636627749	5.35E-05	0.0007454	Ybx1	4	119277981	119294604	-	2531	protein_coding	Y box protein 1 [Source:MGI Symbol;Acc:MGI:99146]	CSD
ENSMUSG0000003154	697.4631859	315.9800988	401.05995	2292.86335	932.3400743	1000.270146	471.5010783	1408.49119	-1.578739116	5.37E-05	0.0007474	Sfrp1	8	23411502	23449632	+	4372	protein_coding	secreted frizzled-related protein 1 [Source:MGI Symbol;Acc:MGI:892014]	-
ENSMUSG0000002780	170.3573874	126.3920395	169.8081126	499.5720296	296.2945151	285.0964521	155.5191798	360.320999	-1.213204309	5.38E-05	0.0007481	Pfn2	3	57841895	57848079	-	3541	protein_coding	profilin 2 [Source:MGI Symbol;Acc:MGI:97550]	-
ENSMUSG0000009560	53.11142077	51.61008281	35.74907633	123.1703452	137.2831253	101.1946451	46.82352663	120.5493719	-1.361833076	5.39E-05	0.0007487	Gm21188	13	120034605	120052194	-	4572	protein_coding	predicted gene, 21188 [Source:MGI Symbol;Acc:MGI:5434543]	-
ENSMUSG0000005096	392.8240932	365.4836476	436.8090264	642.5529899	639.9961527	640.2507354	398.3722557	640.9332926	-0.686454139	5.39E-05	0.0007487	Prkca	11	107933387	108343928	-	8992	protein_coding	protein kinase C, alpha [Source:MGI Symbol;Acc:MGI:97595]	-
ENSMUSG0000002406	377.792559	445.5319394	250.2435343	862.1924167	730.859804	628.5744302	357.8560109	740.542217	-1.048857866	5.44E-05	0.0007539	Lbh	17	72918305	72941947	+	3212	protein_coding	limb-bud and heart [Source:MGI Symbol;Acc:MGI:1925139]	-
ENSMUSG0000003869	771.6187545	753.0859022	651.3034843	1103.365121	1193.079248	1101.464791	725.336047	1132.636386	-0.642528137	5.46E-05	0.0007562	Mapkap1	2	34406771	34624950	+	4059	protein_coding	mitogen-activated protein kinase associated protein 1 [Source:MGI Symbol;Acc:MGI:2444554]	-
ENSMUSG0000000152	376.7904568	367.5901816	401.05995	665.8089292	597.5272722	582.8422348	381.8135295	615.3928121	-0.689231715	5.55E-05	0.0007684	Tulp3	6	128321161	128355851	-	9342	protein_coding	tubby-like protein 3 [Source:MGI Symbol;Acc:MGI:1329045]	Tub
ENSMUSG0000007946	54.11352305	9.479402965	31.28044179	300.6045489	112.5919157	103.140696	31.62445593	172.1123869	-2.443192424	5.59E-05	0.0007735	Col4a3	1	82586921	82722059	+	9967	protein_coding	collagen, type IV, alpha 3 [Source:MGI Symbol;Acc:MGI:104688]	-
ENSMUSG0000005455	79.16608001	18.95880593	159.7536848	373.8176911	347.6522311	435.9153943	85.95952359	385.7951055	-2.167633605	5.64E-05	0.0007798	Gm4876	6	17065010	17171833	-	3058	antisense	predicted gene 4876 [Source:MGI Symbol;Acc:MGI:3647654]	-
ENSMUSG0000004151	246.5171605	311.7670308	242.4234238	506.4626783	428.6393985	488.4587678	266.9025384	474.5202815	-0.830355254	5.65E-05	0.0007802	Irf8	8	120736358	120756694	+	7055	protein_coding	interferon regulatory factor 8 [Source:MGI Symbol;Acc:MGI:96395]	IRF
ENSMUSG0000003831	28.0588638	66.35582075	5.585793176	217.0554336	182.714951	151.7919677	33.33349258	183.8541174	-2.461446792	5.67E-05	0.0007829	Kcnh2	5	24319589	24351604	-	4845	protein_coding	potassium voltage-gated channel, subfamily H (eag-related), member 2 [Source:MGI Symbol;Acc:MGI:1341722]	-
ENSMUSG0000003886	141.2964213	203.2805302	172.0424298	397.9349615	293.33157	337.6398255	172.2064605	342.9687857	-0.995162653	5.72E-05	0.0007895	Garnl3	2	32986224	33131654	-	12352	protein_coding	GTPase activating RANGAP domain-like 3 [Source:MGI Symbol;Acc:MGI:2139309]	-
ENSMUSG0000004179	433.9102866	662.5049405	401.05995	250.6473459	173.8261155	259.7977908	499.1583924	228.0904174	1.129462068	5.77E-05	0.0007951	Myrip	9	120301513	120474841	+	5537	protein_coding	myosin VIIA and Rab interacting protein [Source:MGI Symbol;Acc:MGI:2384407]	-
ENSMUSG0000004861	971.037108	1025.882054	783.1282033	1634.8064	1419.250727	1487.755888	926.6824552	1513.937672	-0.708017542	5.77E-05	0.0007955	Myof	19	37899036	38043577	-	12603	protein_coding	myoferlin [Source:MGI Symbol;Acc:MGI:1919192]	-
ENSMUSG0000003842	1634.428816	1632.563844	1405.385563	2180.890309	2582.700523	2483.160907	1557.459408	2415.583913	-0.632893707	5.78E-05	0.0007964	Usp5	6	124815019	124829484	-	4621	protein_coding	ubiquitin specific peptidase 5 (isopeptidase T) [Source:MGI Symbol;Acc:MGI:1347343]	-
ENSMUSG0000001734	382.8030704	329.6725698	354.1392874	549.5292326	625.1814269	572.1389551	355.5383092	582.2832049	-0.711193077	5.82E-05	0.0008014	Vtn	11	78499091	78502324	+	2030	protein_coding	vitronectin [Source:MGI Symbol;Acc:MGI:98940]	-
ENSMUSG0000002174	2782.838028	3198.771867	2134.890152	3783.827459	8100.692043	6007.459028	2705.500016	5963.992844	-1.140273788	5.83E-05	0.0008021	Pdhb	14	8165996	8173025	-	3861	protein_coding	pyruvate dehydrogenase (lipoamide) beta [Source:MGI Symbol;Acc:MGI:1915513]	-
ENSMUSG0000003953	377.792559	324.4062348	317.2730524	715.7661321	515.5524563	592.5724892	339.8239487	607.9636925	-0.839329211	5.89E-05	0.0008093	Tusc3	8	39005845	39165114	+	3432	protein_coding	tumor suppressor candidate 3 [Source:MGI Symbol;Acc:MGI:1933134]	-
ENSMUSG0000000397	9035.956246	9402.514474	7453.682414	6058.602856	4986.63669	5236.822885	8630.717712	5427.354143	0.669199555	5.92E-05	0.0008132	Rpl8	15	76904078	76906314	+	1359	protein_coding	ribosomal protein L8 [Source:MGI Symbol;Acc:MGI:1350927]	-
ENSMUSG0000002837	29.06096608	15.79900494	10.05442772	94.74641942	68.14773848	62.27362776	18.30479958	75.05592855	-2.029535524	6.02E-05	0.0008254	Astn2	4	65380803	66404611	-	4959	protein_coding	astrotactin 2 [Source:MGI Symbol;Acc:MGI:1889277]	-
ENSMUSG0000009709	185.3889215	164.3096514	244.6577411	83.5491153	103.7030803	81.73413644	198.1187713	89.66211068	1.14387598	6.06E-05	0.0008309	Gm9917	9	107558809	107568415	-	6021	antisense	predicted gene 9917 [Source:MGI Symbol;Acc:MGI:3704343]	-
ENSMUSG0000003737	124.2606825	20.01207293	51.38929722	203.2741362	388.1458148	275.3661978	65.22068423	288.9287163	-2.144918777	6.09E-05	0.0008337	Spon2	5	33198184	33218455	-	2947	protein_coding	spondin 2, extracellular matrix protein [Source:MGI Symbol;Acc:MGI:1923724]	-
ENSMUSG0000011590	53.11142077	125.3387725	102.7785944	27.56259474	28.64180313	11.67630521	93.74292925	22.62690102	2.047657109	6.12E-05	0.000838	AC113595.1	15	79889532	79893120	+	3589	antisense	novel transcript, antisense to Apobec3	-
ENSMUSG0000003974	585.2277307	524.5269641	298.2813556	928.5149103	1156.536257	913.6708823	469.3453501	999.5740167	-1.089890218	6.22E-05	0.0008504	Bahcc1	11	120232947	120292296	+	11118	protein_coding	BAH domain and coiled-coil containing 1 [Source:MGI Symbol;Acc:MGI:2679272]	-
ENSMUSG0000008685	432.9081844	322.2997008	574.2195385	244.6180283	192.5914348	172.2255018	443.1424746	203.1449883	1.123903674	6.23E-05	0.0008512	Snhg20	11	117076008	117078955	+	2795	lincRNA	small nucleolar RNA host gene 20 [Source:MGI Symbol;Acc:MGI:1924222]	-
ENSMUSG0000002237	730.5325611	236.9850741	204.4400302	1328.172534	1248.387557	1139.412783	390.6525552	1238.657625	-1.664110875	6.31E-05	0.0008614	Col14a1	15	55307750	55520803	+	7013	protein_coding	collagen, type XIV, alpha 1 [Source:MGI Symbol;Acc:MGI:1341272]	-
ENSMUSG0000002599	18.03784102	13.69247095	25.69464861	0	0	0.973025434	19.14165352	0.324341811	5.829329193	6.36E-05	0.0008684	Cps1	1	67123026	67231259	+	5555	protein_coding	carbamoyl-phosphate synthetase 1 [Source:MGI Symbol;Acc:MGI:891996]	-
ENSMUSG0000003148	1296.720349	1404.004906	1552.850503	957.8001672	924.4388872	890.3182719	1417.858586	924.1857754	0.617128117	6.37E-05	0.0008686	Plpbp	8	27042555	27056132	+	12648	protein_coding	pyridoxal phosphate binding protein [Source:MGI Symbol;Acc:MGI:1891207]	-
ENSMUSG0000003187	152.3195463	103.2201656	117.3016567	362.620387	218.2702928	264.662918	124.2804562	281.8511993	-1.181437342	6.39E-05	0.0008704	Ces2g	8	104961718	104969537	+	3574	protein_coding	carboxylesterase 2G [Source:MGI Symbol;Acc:MGI:1919611]	-
ENSMUSG0000007348	49.10301165	48.45028182	53.62361449	7.75197977	14.81472576	14.59538151	50.39230265	12.38736234	2.032300224	6.42E-05	0.0008748	H3f3aos	1	180813776	180816859	+	3044	antisense	H3 histone, family 3A, opposite strand [Source:MGI Symbol;Acc:MGI:3802006]	-
ENSMUSG0000001417	831.7448913	730.9672953	894.8440668	522.827969	528.3918853	455.375903	819.1854178	502.1985858	0.70563566	6.55E-05	0.0008907	Tvp23b	11	62879455	62916780	+	2235	protein_coding	trans-golgi network vesicle protein 23B [Source:MGI Symbol;Acc:MGI:1914760]	-
ENSMUSG0000003088	168.3531828	225.3991372	330.678956	86.99443964	114.5672125	95.35649251	241.477092	98.97271489	1.286703324	6.58E-05	0.0008952	Dnhd1	7	105650827	105721799	+	15081	protein_coding	dynein heavy chain domain 1 [Source:MGI Symbol;Acc:MGI:1924755]	-
ENSMUSG0000003014	44.09250026	76.88849072	119.535974	12.0586352	10.86413222	27.24471215	80.17232165	16.72249319	2.262772915	6.59E-05	0.0008955	Clec4e	6	123281789	123289870	-	4965	protein_coding	C-type lectin domain family 4, member e [Source:MGI Symbol;Acc:MGI:1861232]	-
ENSMUSG0000002667	5558.661339	5370.608413	3154.855986	9149.920122	8609.330961	8936.265584	4694.708579	8898.505556	-0.92245221	6.61E-05	0.0008969	Rgs5	1	169655501	169695813	+	4546	protein_coding	regulator of G-protein signaling 5 [Source:MGI Symbol;Acc:MGI:1098434]	-
ENSMUSG0000002826	1460.06302	939.5141605	1527.155854	2011.208085	3021.216406	2746.8508	1308.911012	2593.091763	-0.986229394	6.61E-05	0.0008974	Lmo4	3	144188530	144205220	-	3530	protein_coding	LIM domain only 4 [Source:MGI Symbol;Acc:MGI:109360]	-
ENSMUSG0000007869	311.6538087	420.2535314	368.6623496	599.4864356	642.9590978	625.6553539	366.8565632	622.7002958	-0.763572428	6.63E-05	0.0008987	Cisd3	11	97685826	97688621	+	833	protein_coding	CDGSH iron sulfur domain 3 [Source:MGI Symbol;Acc:MGI:101788]	-
ENSMUSG0000005250	7.01471595	14.74573795	7.820110447	84.41044639	40.49358373	36.97496648	9.860188114	53.95966554	-2.455115913	6.65E-05	0.000901	Epha3	16	63543534	63864175	-	8692	protein_coding	Eph receptor A3 [Source:MGI Symbol;Acc:MGI:99612]	-
ENSMUSG0000000030	115.241762	113.7528356	111.7158635	204.9967984	211.3567541	221.8497989	113.5701537	212.7344505	-0.90501757	6.65E-05	0.000901	Pemt	11	59970614	60046489	-	2104	protein_coding	phosphatidylethanolamine N-methyltransferase [Source:MGI Symbol;Acc:MGI:104535]	-
ENSMUSG0000007523	444.9334117	581.4033818	540.7047794	1447.036224	764.439849	1030.433934	522.347191	1080.636669	-1.049360752	6.66E-05	0.0009016	Amd1	10	40287458	40302186	-	3542	protein_coding	S-adenosylmethionine decarboxylase 1 [Source:MGI Symbol;Acc:MGI:88004]	-
ENSMUSG0000002424	3394.120418	3472.621286	2232.082953	1932.826956	1345.177099	1466.349329	3032.941552	1581.451128	0.9393549	6.68E-05	0.0009027	Cox7a2l	17	83501919	83514333	-	1147	protein_coding	cytochrome c oxidase subunit 7A2 like [Source:MGI Symbol;Acc:MGI:106015]	-
ENSMUSG0000003953	0	4.213067984	0	602.0704288	40.49358373	112.8709503	1.404355995	251.8116543	-7.485110677	6.70E-05	0.0009056	Mmd2	5	142562358	142608800	-	5193	protein_coding	monocyte to macrophage differentiation-associated 2 [Source:MGI Symbol;Acc:MGI:1922354]	-
ENSMUSG0000002977	227.4772172	188.5347923	271.4695484	394.4896372	467.1576855	410.6167331	229.1605193	424.0880186	-0.8883871	6.73E-05	0.000909	Irf5	6	29526625	29541871	+	2905	protein_coding	interferon regulatory factor 5 [Source:MGI Symbol;Acc:MGI:1350924]	IRF
ENSMUSG0000003121	1889.964897	1573.580892	2108.078345	1063.743891	1183.202764	1190.010106	1857.208045	1145.652253	0.697035441	6.74E-05	0.0009095	Ophn1	X	98554277	98891025	-	13100	protein_coding	oligophrenin 1 [Source:MGI Symbol;Acc:MGI:2151070]	-
ENSMUSG0000000899	5.010511393	8.426135969	20.10885543	201.551474	27.65415474	55.46244973	11.18183427	94.8893595	-3.094017739	6.79E-05	0.000915	Bmp7	2	172868012	172940321	-	4019	protein_coding	bone morphogenetic protein 7 [Source:MGI Symbol;Acc:MGI:103302]	-
ENSMUSG0000000122	12.02522734	33.70454388	23.46033134	152.4556021	52.34536434	103.140696	23.06336752	102.6472208	-2.157857506	6.82E-05	0.0009183	Uhrf1	17	56303321	56323486	+	4259	protein_coding	ubiquitin-like, containing PHD and RING finger domains, 1 [Source:MGI Symbol;Acc:MGI:1338889]	-
ENSMUSG0000003225	24.05045469	23.17187391	45.80350404	114.5570344	79.0118707	101.1946451	31.00861088	98.25451673	-1.669449148	6.86E-05	0.0009228	Kif23	9	61915905	61946774	-	7901	protein_coding	kinesin family member 23 [Source:MGI Symbol;Acc:MGI:1919069]	-
ENSMUSG0000001994	1580.315293	2123.386264	1743.88463	797.5925853	1062.709661	1170.549597	1815.862062	1010.283948	0.846213149	6.90E-05	0.0009285	Arid5b	10	68092520	68278740	-	10060	protein_coding	AT rich interactive domain 5B (MRF1-like) [Source:MGI Symbol;Acc:MGI:2175912]	ARID
ENSMUSG0000009169	25.05255697	14.74573795	34.63191769	0	0.987648384	2.919076301	24.81007087	1.302241562	4.287031581	6.93E-05	0.0009314	Apol11b	15	77633946	77643286	-	2463	protein_coding	apolipoprotein L 11b [Source:MGI Symbol;Acc:MGI:3036248]	-
ENSMUSG0000007289	258.5423879	325.4595018	422.2859641	192.0768321	126.4189931	108.9788486	335.4292846	142.4915579	1.232599225	6.97E-05	0.0009363	4933439C10Ri	11	59505662	59513647	+	5616	processed_transcrip	RIKEN cDNA 4933439C10 gene [Source:MGI Symbol;Acc:MGI:1921726]	-
ENSMUSG0000002460	8844.554711	9872.271554	8710.485879	6674.454582	5279.96826	4692.901667	9142.437381	5549.10817	0.720240134	6.99E-05	0.0009388	Rps14	18	60774510	60778546	+	1384	protein_coding	ribosomal protein S14 [Source:MGI Symbol;Acc:MGI:98107]	-
ENSMUSG0000002734	1751.674783	2959.680259	3622.945454	1052.546587	1286.905844	1524.730855	2778.100165	1288.061095	1.10891392	7.00E-05	0.0009388	Gpcpd1	2	132529082	132587729	-	7289	protein_coding	glycerophosphocholine phosphodiesterase 1 [Source:MGI Symbol;Acc:MGI:104898]	-
ENSMUSG0000002089	594.2466512	572.9772459	573.1023799	354.8684073	387.1581664	296.7727573	580.108759	346.2664437	0.744449908	7.03E-05	0.0009418	Pfas	11	68985697	69008460	-	7933	protein_coding	phosphoribosylformylglycinamidine synthase (FGAR amidotransferase) [Source:MGI Symbol;Acc:MGI:2684864]	-
ENSMUSG0000001547	40.08409114	38.97087886	68.14667675	238.5887107	109.6289706	124.5472555	49.06721558	157.5883123	-1.687309756	7.04E-05	0.0009428	Prrt1	17	34629533	34633126	+	2306	protein_coding	proline-rich transmembrane protein 1 [Source:MGI Symbol;Acc:MGI:1932118]	-
ENSMUSG0000000166	1236.594212	493.9822212	834.5175005	1804.488624	1847.890126	2341.099194	855.0313112	1997.825981	-1.224148394	7.09E-05	0.000949	Gstt1	10	75783813	75798584	-	1528	protein_coding	glutathione S-transferase, theta 1 [Source:MGI Symbol;Acc:MGI:107379]	-
ENSMUSG0000003568	2.004204557	0	3.351475906	35.31457451	20.74061606	22.37958498	1.785226821	26.14492518	-3.887679248	7.10E-05	0.0009499	Melk	4	44300876	44364675	+	2972	protein_coding	maternal embryonic leucine zipper kinase [Source:MGI Symbol;Acc:MGI:106924]	-
ENSMUSG0000002703	388.8156841	312.8202978	287.1097693	631.3556857	512.5895112	616.898125	329.5819171	586.947774	-0.832085465	7.13E-05	0.0009531	Stk39	2	68210445	68472268	-	4955	protein_coding	serine/threonine kinase 39 [Source:MGI Symbol;Acc:MGI:1858416]	-
ENSMUSG0000003203	2531.310356	2804.850011	3047.608757	1801.0433	1821.22362	1951.88902	2794.589708	1858.05198	0.588781026	7.20E-05	0.0009619	Ets1	9	32636221	32757820	+	8476	protein_coding	E26 avian leukemia oncogene 1, 5' domain [Source:MGI Symbol;Acc:MGI:95455]	ETS
ENSMUSG0000002196	8.016818229	7.372868973	69.26383538	0	0.987648384	0	28.21784086	0.329216128	6.387834086	7.25E-05	0.000968	Prss52	14	64104286	64113755	+	1064	protein_coding	protease, serine 52 [Source:MGI Symbol;Acc:MGI:1920632]	-
ENSMUSG0000007370	5675.907306	4855.560852	5013.807955	3782.104797	2826.649674	2970.646649	5181.758704	3193.133707	0.69834765	7.30E-05	0.0009723	Rpl31	1	39367842	39371911	+	1662	protein_coding	ribosomal protein L31 [Source:MGI Symbol;Acc:MGI:2149632]	-
ENSMUSG0000003624	91.19130735	116.9126366	71.49815265	306.6338665	172.8384672	212.1195446	93.20069886	230.5306261	-1.306820247	7.30E-05	0.0009723	Armh4	14	49675952	49783383	-	9271	protein_coding	armadillo-like helical domain containing 4 [Source:MGI Symbol;Acc:MGI:1914669]	-
ENSMUSG0000002667	107.2249438	88.47442767	31.28044179	242.0340351	227.1591283	229.6340024	75.65993776	232.9423886	-1.619481847	7.30E-05	0.0009723	Ccdc3	2	5137776	5230878	+	2670	protein_coding	coiled-coil domain containing 3 [Source:MGI Symbol;Acc:MGI:1921436]	-
ENSMUSG0000002762	1943.076318	1686.280461	985.3339163	2702.856947	3261.214963	3496.080384	1538.230232	3153.384098	-1.035347648	7.33E-05	0.0009752	Epb41l1	2	156420909	156543214	+	11530	protein_coding	erythrocyte membrane protein band 4.1 like 1 [Source:MGI Symbol;Acc:MGI:103010]	-
ENSMUSG0000003635	79.16608001	48.45028182	23.46033134	139.5356359	206.4185122	157.6301203	50.35889772	167.8614228	-1.732312124	7.33E-05	0.0009752	P2ry12	3	59216272	59262871	-	2546	protein_coding	purinergic receptor P2Y, G-protein coupled 12 [Source:MGI Symbol;Acc:MGI:1918089]	-
ENSMUSG0000004171	988.0728467	1205.990711	1034.488896	698.5395104	647.8973397	704.4704141	1076.184151	683.6357547	0.654471914	7.46E-05	0.000991	Ubr7	12	102757967	102777707	+	3261	protein_coding	ubiquitin protein ligase E3 component n-recognin 7 (putative) [Source:MGI Symbol;Acc:MGI:1913872]	-
ENSMUSG0000010960	1.002102279	8.426135969	0	33.59191234	43.45652888	38.92101735	3.142746082	38.65648619	-3.614776296	7.47E-05	0.0009914	Gm32389	8	27008467	27012903	+	1621	lincRNA	predicted gene, 32389 [Source:MGI Symbol;Acc:MGI:5591548]	-
ENSMUSG0000003008	602.2634694	676.1974115	602.1485044	397.9349615	411.849376	360.9924359	626.8697951	390.2589245	0.683672512	7.51E-05	0.0009967	Abtb1	6	88835914	88841984	-	4862	protein_coding	ankyrin repeat and BTB (POZ) domain containing 1 [Source:MGI Symbol;Acc:MGI:1933148]	-
ENSMUSG0000002962	220.4625013	185.3749913	211.1429821	107.6663857	110.616619	104.1137214	205.6601582	107.4655754	0.936532367	7.52E-05	0.0009967	Zkscan14	5	145194946	145201868	-	3621	protein_coding	zinc finger with KRAB and SCAN domains 14 [Source:MGI Symbol;Acc:MGI:1914485]	zf-C2
ENSMUSG0000004685	68.14295495	109.5397676	207.7915062	29.28525691	36.5429902	40.86706822	128.4914096	35.56510511	1.853182713	7.53E-05	0.0009972	Gpr1	1	63182691	63214543	-	2496	protein_coding	G protein-coupled receptor 1 [Source:MGI Symbol;Acc:MGI:2385324]	-
ENSMUSG0000004226	24.05045469	46.34374783	58.09224903	4.306655428	9.876483837	0.973025434	42.82881718	5.0520549	3.087798935	7.58E-05	0.0010028	Trem1	17	48232768	48246924	+	2996	protein_coding	triggering receptor expressed on myeloid cells 1 [Source:MGI Symbol;Acc:MGI:1930005]	-
ENSMUSG0000005620	234.4919332	266.47655	213.3772993	474.5934282	395.0593535	393.1022752	238.1152608	420.9183523	-0.82209467	7.58E-05	0.0010028	Pgpep1	8	70646435	70660388	-	5319	protein_coding	pyroglutamyl-peptidase I [Source:MGI Symbol;Acc:MGI:1913772]	-
ENSMUSG0000002913	1795.767283	1705.239267	2152.76469	1199.834202	1171.350983	1254.229784	1884.590413	1208.471656	0.640918575	7.60E-05	0.0010048	Dnajb6	5	29735688	29818134	+	6062	protein_coding	DnaJ heat shock protein family (Hsp40) member B6 [Source:MGI Symbol;Acc:MGI:1344381]	-
ENSMUSG0000008656	44.09250026	23.17187391	44.68634541	5.167986514	3.950593535	8.757228904	37.31690653	5.958602984	2.648782055	7.63E-05	0.0010083	Gm2830	13	54499705	54500089	-	385	processed_pseudog	predicted gene 2830 [Source:MGI Symbol;Acc:MGI:3781002]	-
ENSMUSG0000002244	12517.25956	13617.68899	8672.502485	6339.39679	6747.613758	6976.59236	11602.48368	6687.867636	0.794858545	7.72E-05	0.0010198	Myh9	15	77760587	77842175	-	12928	protein_coding	myosin, heavy polypeptide 9, non-muscle [Source:MGI Symbol;Acc:MGI:107717]	-
ENSMUSG0000002937	131.2753985	115.8593696	153.050733	247.2020216	238.0232605	278.2852741	133.395167	254.5035187	-0.932732004	7.75E-05	0.001023	Mthfd2l	5	90931117	91021368	+	6645	protein_coding	methylenetetrahydrofolate dehydrogenase (NADP+ dependent) 2-like [Source:MGI Symbol;Acc:MGI:1915871]	-
ENSMUSG0000003772	9.018920508	9.479402965	7.820110447	70.62914902	24.69120959	49.62429712	8.772811306	48.31488524	-2.462533922	7.79E-05	0.0010272	Ckap2	8	22168160	22185819	-	3215	protein_coding	cytoskeleton associated protein 2 [Source:MGI Symbol;Acc:MGI:1931797]	-
ENSMUSG0000002244	202.4246603	169.5759864	268.1180725	844.965795	314.072186	550.7323955	213.3729064	569.9234588	-1.418403329	7.80E-05	0.0010273	C1qtnf6	15	78523346	78531416	-	2702	protein_coding	C1q and tumor necrosis factor related protein 6 [Source:MGI Symbol;Acc:MGI:1919959]	-
ENSMUSG0000007104	481.0090937	489.7691532	422.2859641	301.46588	205.4308638	252.0135873	464.354737	252.9701104	0.875212445	7.82E-05	0.0010296	Impdh2-ps	8	100030558	100032102	+	1545	processed_pseudog	inosine monophosphate dehydrogenase 2, pseudogene [Source:MGI Symbol;Acc:MGI:3705743]	-
ENSMUSG0000004275	128.2690917	116.9126366	128.4732431	260.1219878	235.0603153	210.1734937	124.5516571	235.118599	-0.917352671	7.85E-05	0.0010326	Apobr	7	126584942	126589112	+	3779	protein_coding	apolipoprotein B receptor [Source:MGI Symbol;Acc:MGI:2176230]	-
ENSMUSG0000002934	709.4884133	717.2748243	576.4538558	1403.96967	992.5866257	1110.22202	667.7390311	1168.926105	-0.807887723	7.89E-05	0.0010377	Tpst2	5	112276691	112315361	+	2793	protein_coding	protein-tyrosine sulfotransferase 2 [Source:MGI Symbol;Acc:MGI:1309516]	-
ENSMUSG0000001865	709.4884133	762.5653052	531.7675104	351.4230829	419.7505631	314.2872151	667.9404096	361.820287	0.884972053	7.99E-05	0.0010496	Pnpo	11	96937825	96943986	-	2925	protein_coding	pyridoxine 5'-phosphate oxidase [Source:MGI Symbol;Acc:MGI:2144151]	-
ENSMUSG0000002443	159.3342623	159.0433164	178.7453816	331.612468	325.9239666	258.8247656	165.7076534	305.4537333	-0.883243456	7.99E-05	0.0010496	Mrps18b	17	35910379	35916389	-	2089	protein_coding	mitochondrial ribosomal protein S18B [Source:MGI Symbol;Acc:MGI:1914223]	-
ENSMUSG0000003442	980.0560285	754.1391692	754.0820788	1255.820723	1482.460224	1378.77704	829.4257588	1372.352662	-0.725957618	8.05E-05	0.0010562	Gcsh	8	116981810	116993537	-	1579	protein_coding	glycine cleavage system protein H (aminomethyl carrier) [Source:MGI Symbol;Acc:MGI:1915383]	-
ENSMUSG0000002289	338.7105702	657.2386056	484.8468477	230.8367309	249.8750411	213.09257	493.5986745	231.268114	1.093555083	8.10E-05	0.0010621	Slc15a2	16	36750177	36784962	-	7361	protein_coding	solute carrier family 15 (H+/peptide transporter), member 2 [Source:MGI Symbol;Acc:MGI:1890457]	-
ENSMUSG0000004027	494.0364234	490.8224202	412.2315364	299.7432178	265.6774152	251.0405619	465.6967933	272.1537316	0.774635275	8.11E-05	0.0010621	Accs	2	93833467	93849943	-	8364	protein_coding	1-aminocyclopropane-1-carboxylate synthase (non-functional) [Source:MGI Symbol;Acc:MGI:1919717]	-
ENSMUSG0000005685	3416.166668	4276.264004	3198.425173	2397.945742	2230.11005	2216.551938	3630.285282	2281.53591	0.670045682	8.11E-05	0.0010625	Pcbp2	15	102470539	102500061	+	5203	protein_coding	poly(rC) binding protein 2 [Source:MGI Symbol;Acc:MGI:108202]	-
ENSMUSG0000002179	1918.023761	1666.268388	1496.992571	2353.156526	3178.252499	2967.727573	1693.761573	2833.045533	-0.741827881	8.15E-05	0.0010662	Bmpr1a	14	34410734	34503341	-	6351	protein_coding	bone morphogenetic protein receptor, type 1A [Source:MGI Symbol;Acc:MGI:1338938]	-
ENSMUSG0000004020	13.02732962	15.79900494	13.40590362	64.59983142	34.56769343	78.81506014	14.07741273	59.32752833	-2.076212486	8.21E-05	0.0010731	Pclaf	9	65890237	65903266	+	4813	protein_coding	PCNA clamp associated factor [Source:MGI Symbol;Acc:MGI:1915276]	-
ENSMUSG0000003547	1836.853477	1068.012734	1066.886497	426.3588874	692.341517	683.0638545	1323.917569	600.5880863	1.141015805	8.23E-05	0.0010752	Tab3	X	85574022	85634469	+	6639	protein_coding	TGF-beta activated kinase 1/MAP3K7 binding protein 3 [Source:MGI Symbol;Acc:MGI:1913974]	-
ENSMUSG0000002645	8.016818229	10.53266996	14.52306226	94.74641942	39.50593535	37.94799192	11.02418348	57.40011556	-2.388155491	8.30E-05	0.0010841	Syt2	1	134646677	134762593	+	7638	protein_coding	synaptotagmin II [Source:MGI Symbol;Acc:MGI:99666]	-
ENSMUSG0000002662	670.4064244	851.0397328	414.4658537	228.2527377	318.0227796	316.233266	645.3040036	287.5029277	1.167338835	8.31E-05	0.0010842	Atf3	1	191170296	191218039	-	2154	protein_coding	activating transcription factor 3 [Source:MGI Symbol;Acc:MGI:109384]	TF_b
ENSMUSG0000001935	0	4.213067984	0	66.32249359	16.79002252	20.43353411	1.404355995	34.51535007	-4.619350474	8.33E-05	0.0010861	Gdpd2	X	100729873	100738895	+	4609	protein_coding	glycerophosphodiester phosphodiesterase domain containing 2 [Source:MGI Symbol;Acc:MGI:1918834]	-
ENSMUSG0000007296	298.626479	230.6654721	368.6623496	161.9302441	133.3325318	144.0077642	299.3181003	146.4235134	1.030182188	8.34E-05	0.0010861	Armcx5	X	135742733	135747322	+	4247	protein_coding	armadillo repeat containing, X-linked 5 [Source:MGI Symbol;Acc:MGI:2148026]	-
ENSMUSG0000003877	1618.39518	2126.546065	1979.605102	1124.037067	1294.807031	1128.709503	1908.182116	1182.517867	0.690346669	8.38E-05	0.0010909	Kdm3b	18	34777047	34838660	+	14095	protein_coding	KDM3B lysine (K)-specific demethylase 3B [Source:MGI Symbol;Acc:MGI:1923356]	-
ENSMUSG0000005221	64.13454583	48.45028182	449.0977714	3.445324342	14.81472576	2.919076301	187.227533	7.0597088	4.733237243	8.47E-05	0.0011027	Cd177	7	24743983	24760311	-	2840	protein_coding	CD177 antigen [Source:MGI Symbol;Acc:MGI:1916141]	-
ENSMUSG0000002897	6.012613672	2.106533992	0	33.59191234	42.4688805	21.40655954	2.706382555	32.48911746	-3.557077191	8.55E-05	0.0011108	Slc2a5	4	150119283	150144169	+	3402	protein_coding	solute carrier family 2 (facilitated glucose transporter), member 5 [Source:MGI Symbol;Acc:MGI:1928369]	-
ENSMUSG0000007300	342.7189793	357.0575117	294.9298797	206.7194605	176.7890607	170.2794509	331.5687902	184.5959907	0.844257842	8.55E-05	0.0011108	Vamp5	6	72368794	72380468	-	2707	protein_coding	vesicle-associated membrane protein 5 [Source:MGI Symbol;Acc:MGI:1858622]	-
ENSMUSG0000008504	27.05676152	31.59800988	33.51475906	5.167986514	3.950593535	0	30.72317682	3.039526683	3.318804719	8.59E-05	0.0011156	Parvaos	7	112526972	112535683	-	1285	antisense	parvin, alpha, opposite strand [Source:MGI Symbol;Acc:MGI:2445046]	-
ENSMUSG0000001585	264.5550016	153.7769814	348.5534942	633.0783479	588.6384367	515.7034799	255.6284924	579.1400882	-1.18060066	8.71E-05	0.0011295	Adamtsl4	3	95676201	95687917	-	4198	protein_coding	ADAMTS-like 4 [Source:MGI Symbol;Acc:MGI:2389008]	-
ENSMUSG0000007117	710.4905155	560.3380419	436.8090264	972.4427956	1045.919638	1076.16613	569.2125279	1031.509521	-0.856959489	8.72E-05	0.0011295	Arhgef10	8	14911663	15001085	+	9210	protein_coding	Rho guanine nucleotide exchange factor (GEF) 10 [Source:MGI Symbol;Acc:MGI:2444453]	-
ENSMUSG0000004909	1093.293586	1084.865006	851.27488	1484.934792	1655.298691	1842.910172	1009.811157	1661.047885	-0.717586389	8.72E-05	0.0011295	Zadh2	18	84088158	84097514	+	3321	protein_coding	zinc binding alcohol dehydrogenase, domain containing 2 [Source:MGI Symbol;Acc:MGI:2444835]	-
ENSMUSG0000007947	32.06727292	52.6633498	42.45202814	123.1703452	85.92540939	144.0077642	42.39421695	117.7011729	-1.474896339	8.77E-05	0.0011359	Utp14b	1	78658038	78671512	+	7679	protein_coding	UTP14B small subunit processome component [Source:MGI Symbol;Acc:MGI:2445092]	-
ENSMUSG0000002922	328.6895474	459.2244103	601.0313457	264.4286433	123.456048	156.6570948	462.9817678	181.5139287	1.348753071	8.86E-05	0.0011467	Lnx1	5	74592447	74702912	-	3829	protein_coding	ligand of numb-protein X 1 [Source:MGI Symbol;Acc:MGI:1278335]	-
ENSMUSG0000002749	297.6243767	264.370016	243.5405825	428.9428806	555.0583917	442.7265724	268.5116584	475.5759482	-0.823624635	8.90E-05	0.0011514	Pkia	3	7366669	7445366	+	3721	protein_coding	protein kinase inhibitor, alpha [Source:MGI Symbol;Acc:MGI:104747]	-
ENSMUSG0000008491	51.10721621	36.86434486	45.80350404	11.19730411	11.8517806	9.730254338	44.59168837	10.92644635	2.029017543	9.00E-05	0.0011633	C230037L18Ri	15	89476236	89484847	+	2017	antisense	RIKEN cDNA C230037L18 gene [Source:MGI Symbol;Acc:MGI:3697701]	-
ENSMUSG0000002066	626.3139241	741.4999653	463.6208336	972.4427956	1204.931028	1186.118004	610.478241	1121.163943	-0.876435121	9.02E-05	0.0011642	Dnmt3a	12	3806007	3914443	+	16642	protein_coding	DNA methyltransferase 3A [Source:MGI Symbol;Acc:MGI:1261827]	-
ENSMUSG0000003078	28.0588638	108.4865006	87.13837355	239.4500418	181.7273026	391.1562244	74.56124598	270.7778563	-1.862162394	9.02E-05	0.0011645	Itgax	7	128129547	128150657	+	4803	protein_coding	integrin alpha X [Source:MGI Symbol;Acc:MGI:96609]	-
ENSMUSG0000002077	1098.304097	884.7442767	1043.426165	646.8596453	664.6873623	591.5994637	1008.824846	634.3821571	0.669251748	9.04E-05	0.0011657	Mrpl38	11	116131817	116138868	-	2550	protein_coding	mitochondrial ribosomal protein L38 [Source:MGI Symbol;Acc:MGI:1926269]	-
ENSMUSG0000008994	16.03363646	23.17187391	31.28044179	62.87716925	79.99951908	83.6801873	23.49531739	75.51895855	-1.688510943	9.08E-05	0.0011695	Gm4117	13	89739460	89887166	+	52997	antisense	predicted gene 4117 [Source:MGI Symbol;Acc:MGI:3782293]	-
ENSMUSG0000002279	626.3139241	408.6675945	749.6134442	284.2392582	316.0474828	282.1773758	594.8649876	294.1547056	1.015913552	9.11E-05	0.0011728	B4galt4	16	38742264	38769049	+	3213	protein_coding	UDP-Gal:betaGlcNAc beta 1,4-galactosyltransferase, polypeptide 4 [Source:MGI Symbol;Acc:MGI:1928387]	-
ENSMUSG0000000336	1657.477169	1642.043247	1748.353264	1220.506148	1119.005619	1100.491766	1682.62456	1146.667844	0.552982006	9.21E-05	0.0011847	Ddx23	15	98645134	98662894	-	6229	protein_coding	DEAD (Asp-Glu-Ala-Asp) box polypeptide 23 [Source:MGI Symbol;Acc:MGI:1921601]	-
ENSMUSG0000001284	7790.343114	8526.196333	7116.300506	5727.851719	4252.81394	4038.05555	7810.946651	4672.90707	0.741084639	9.21E-05	0.0011847	Rps5	7	12922290	12926686	+	1734	protein_coding	ribosomal protein S5 [Source:MGI Symbol;Acc:MGI:1097682]	-
ENSMUSG0000003017	548.1499464	506.6214251	335.1475906	731.2700917	959.0065806	1062.543774	463.3063207	917.6068153	-0.985034044	9.24E-05	0.0011879	Erc1	6	119570796	119848167	-	16271	protein_coding	ELKS/RAB6-interacting/CAST family member 1 [Source:MGI Symbol;Acc:MGI:2151013]	-
ENSMUSG0000003284	502.0532416	1085.918273	683.7010848	343.6711032	353.5781214	341.5319273	757.2241998	346.2603839	1.128770613	9.29E-05	0.0011936	Abcc4	14	118482692	118706219	-	6389	protein_coding	ATP-binding cassette, sub-family C (CFTR/MRP), member 4 [Source:MGI Symbol;Acc:MGI:2443111]	-
ENSMUSG0000004821	752.5788112	584.5631828	529.5331931	358.3137316	371.3557923	333.7477238	622.2250624	354.4724159	0.812250496	9.30E-05	0.0011937	Amigo2	15	97244125	97247287	-	3163	protein_coding	adhesion molecule with Ig like domain 2 [Source:MGI Symbol;Acc:MGI:2145995]	-
ENSMUSG0000005233	7.01471595	31.59800988	8.937269082	177.4342036	66.17244171	54.48942429	15.84999831	99.36535654	-2.649843849	9.31E-05	0.0011944	Cx3cr1	9	119901616	120069879	-	4505	protein_coding	chemokine (C-X3-C motif) receptor 1 [Source:MGI Symbol;Acc:MGI:1333815]	-
ENSMUSG0000011375	40.08409114	20.01207293	42.45202814	6.890648685	4.938241919	2.919076301	34.18273074	4.915988968	2.787467392	9.32E-05	0.0011944	Gm5628	13	17949908	17950956	+	1049	processed_pseudog	predicted gene 5628 [Source:MGI Symbol;Acc:MGI:3779506]	-
ENSMUSG0000006219	239.5024446	293.8614919	256.9464861	424.6362252	432.5899921	478.7285134	263.4368075	445.3182436	-0.75738524	9.33E-05	0.0011946	Lancl2	6	57702540	57739439	+	3020	protein_coding	LanC (bacterial lantibiotic synthetase component C)-like 2 [Source:MGI Symbol;Acc:MGI:1919085]	-
ENSMUSG0000003250	16.03363646	7.372868973	7.820110447	167.0982306	29.62945151	38.92101735	10.40887196	78.54956649	-2.913595753	9.33E-05	0.0011946	Dclk3	9	111439081	111489118	+	3005	protein_coding	doublecortin-like kinase 3 [Source:MGI Symbol;Acc:MGI:3039580]	-
ENSMUSG0000004443	59.12403444	64.24928676	55.85793176	198.9674808	131.357235	114.8170012	59.74375099	148.3805723	-1.313815349	9.36E-05	0.0011967	Camsap3	8	3587293	3609075	+	6119	protein_coding	calmodulin regulated spectrin-associated protein family, member 3 [Source:MGI Symbol;Acc:MGI:1916947]	-
ENSMUSG0000007433	5.010511393	57.92968479	13.40590362	0.861331086	0	0	25.44869993	0.287110362	6.24107081	9.37E-05	0.0011972	Apoc4	7	19678083	19681479	-	2537	protein_coding	apolipoprotein C-IV [Source:MGI Symbol;Acc:MGI:87878]	-
ENSMUSG0000002824	1379.894838	1487.212998	1482.469509	927.6535792	965.9201193	1040.164189	1449.859115	977.9126291	0.568227996	9.41E-05	0.0012019	Nsmaf	4	6396207	6454271	-	8368	protein_coding	neutral sphingomyelinase (N-SMase) activation associated factor [Source:MGI Symbol;Acc:MGI:1341864]	-
ENSMUSG0000003633	191.4015352	146.4041125	88.25553218	322.9991571	348.6398795	304.5569608	142.0203933	325.3986658	-1.194035934	9.53E-05	0.0012165	Igsf10	3	59316735	59344394	-	10150	protein_coding	immunoglobulin superfamily, member 10 [Source:MGI Symbol;Acc:MGI:1923481]	-
ENSMUSG0000006051	891.871028	821.548257	894.8440668	530.5799487	604.4408109	563.3817262	869.4211172	566.1341619	0.619265664	9.56E-05	0.0012199	Tor3a	1	156653617	156674356	-	3892	protein_coding	torsin family 3, member A [Source:MGI Symbol;Acc:MGI:1353652]	-
ENSMUSG0000005855	10051.08585	11726.02147	9260.127927	7461.711194	5756.01478	5965.618934	10345.74508	6394.448303	0.694090005	9.58E-05	0.0012208	Rpl5	5	107900502	107909005	+	3920	protein_coding	ribosomal protein L5 [Source:MGI Symbol;Acc:MGI:102854]	-
ENSMUSG0000002806	541.1352305	490.8224202	401.05995	881.1417006	889.8711938	707.3894904	477.6725336	826.1341282	-0.789911854	9.63E-05	0.0012267	Sema4a	3	88435959	88461182	-	5406	protein_coding	sema domain, immunoglobulin domain (Ig), transmembrane domain (TM) and short cytoplasmic domain, (semaphorin) 4A [Source	-
ENSMUSG0000002768	1818.815636	1914.839399	1976.253626	1238.594101	1350.115341	1334.01787	1903.302887	1307.57577	0.541692029	9.64E-05	0.0012271	Fxr1	3	34019943	34070322	+	6553	protein_coding	fragile X mental retardation gene 1, autosomal homolog [Source:MGI Symbol;Acc:MGI:104860]	-
ENSMUSG0000002891	568.191992	350.7379097	631.1946289	875.973714	1051.845529	1179.306826	516.7081769	1035.708689	-1.003198599	9.65E-05	0.0012271	Mecr	4	131843470	131867786	+	3041	protein_coding	mitochondrial trans-2-enoyl-CoA reductase [Source:MGI Symbol;Acc:MGI:1349441]	-
ENSMUSG0000003806	1897.981716	2102.320924	1501.461206	1213.6155	826.6616972	1027.514858	1833.921282	1022.597352	0.842468776	9.72E-05	0.0012352	Rnf144b	13	47122656	47247991	+	4936	protein_coding	ring finger protein 144B [Source:MGI Symbol;Acc:MGI:2384986]	-
ENSMUSG0000006420	109.2291484	75.83522372	92.72416672	202.4128051	168.8878736	210.1734937	92.5961796	193.8247241	-1.065227421	9.74E-05	0.001237	4430402I18Rik	19	28893042	28967800	-	5420	protein_coding	RIKEN cDNA 4430402I18 gene [Source:MGI Symbol;Acc:MGI:1918036]	-
ENSMUSG0000000612	1392.922167	1469.30746	1394.213977	1018.954674	842.4640713	940.9155945	1418.814535	934.1114467	0.602679089	9.79E-05	0.0012433	Inpp5k	11	75630988	75648871	+	5728	protein_coding	inositol polyphosphate 5-phosphatase K [Source:MGI Symbol;Acc:MGI:1194899]	-
ENSMUSG0000002103	9594.127215	7324.418691	9809.769976	12578.87917	15469.53663	16301.09509	8909.438627	14783.1703	-0.730532554	9.81E-05	0.001244	Gstz1	12	87147165	87164723	+	5088	protein_coding	glutathione transferase zeta 1 (maleylacetoacetate isomerase) [Source:MGI Symbol;Acc:MGI:1341859]	-
ENSMUSG0000007103	10.02102279	26.3316749	111.7158635	5.167986514	3.950593535	2.919076301	49.35618707	4.012552117	3.612927144	9.81E-05	0.001244	Gm10309	17	86497732	86505232	-	2005	protein_coding	predicted gene 10309 [Source:MGI Symbol;Acc:MGI:3641941]	-
ENSMUSG0000003448	2241.702797	2544.693063	2089.086648	1561.593258	1464.682553	1543.218338	2291.827503	1523.164716	0.589419724	9.82E-05	0.0012441	Uaca	9	60794542	60880370	+	5807	protein_coding	uveal autoantigen with coiled-coil domains and ankyrin repeats [Source:MGI Symbol;Acc:MGI:1919815]	-
ENSMUSG0000006405	24.05045469	46.34374783	8.937269082	1.722662171	1.975296767	0	26.44382387	1.23265298	4.413073503	9.85E-05	0.0012475	Scgb3a1	11	49663610	49665118	+	598	protein_coding	secretoglobin, family 3A, member 1 [Source:MGI Symbol;Acc:MGI:1915912]	-
ENSMUSG0000002061	344.7231838	349.6846427	560.8136349	233.4207242	190.6161381	155.6840694	418.4071538	193.2403106	1.112925382	9.89E-05	0.0012511	Fam20a	11	109669749	109722279	-	3634	protein_coding	family with sequence similarity 20, member A [Source:MGI Symbol;Acc:MGI:2388266]	-
ENSMUSG0000002854	78.16397773	49.50354882	75.96678719	168.8208928	161.9743349	137.1965862	67.87810458	155.9972713	-1.200890868	9.90E-05	0.0012511	B4galt2	4	117869260	117883487	-	5031	protein_coding	UDP-Gal:betaGlcNAc beta 1,4- galactosyltransferase, polypeptide 2 [Source:MGI Symbol;Acc:MGI:1858493]	-
ENSMUSG0000005504	1039.180063	1217.576647	1538.327441	744.190058	782.2175199	596.4645909	1265.02805	707.6240563	0.837765171	9.92E-05	0.0012539	Pdlim1	19	40221173	40271842	-	5260	protein_coding	PDZ and LIM domain 1 (elfin) [Source:MGI Symbol;Acc:MGI:1860611]	-
ENSMUSG0000003033	1045.192677	1048.000661	873.6180527	1782.955347	1444.929585	1509.162448	988.9371302	1579.015793	-0.675068158	0.0001001	0.0012636	Ltbr	6	125306571	125313885	-	2354	protein_coding	lymphotoxin B receptor [Source:MGI Symbol;Acc:MGI:104875]	-
ENSMUSG0000003241	556.1667646	556.1249739	561.9307935	754.526031	1297.769976	1068.381926	558.0741774	1040.225978	-0.897963277	0.0001004	0.0012667	Rasa2	9	96539300	96631617	-	15856	protein_coding	RAS p21 protein activator 2 [Source:MGI Symbol;Acc:MGI:2149960]	-
ENSMUSG0000004539	468.9838664	471.8636143	1933.801598	204.9967984	339.751044	219.903748	958.2163594	254.8838635	1.910532734	0.0001007	0.0012693	Olfr1033	2	86020633	86045171	+	4261	protein_coding	olfactory receptor 1033 [Source:MGI Symbol;Acc:MGI:3030867]	-
ENSMUSG0000004654	2020.238194	2053.870642	1899.16968	1450.481548	1281.967602	1137.466732	1991.092839	1289.971961	0.625990503	0.0001009	0.001272	Fam43a	16	30599723	30602797	+	3075	protein_coding	family with sequence similarity 43, member A [Source:MGI Symbol;Acc:MGI:2676309]	-
ENSMUSG0000006045	50.10511393	54.7698838	75.96678719	3.445324342	20.74061606	6.811178036	60.28059497	10.33237281	2.555928943	0.0001013	0.001276	Kng2	16	22985854	23029482	-	2734	protein_coding	kininogen 2 [Source:MGI Symbol;Acc:MGI:3027157]	-
ENSMUSG0000003919	1204.526939	1278.666133	1221.054388	1712.326198	1830.112455	1803.016129	1234.749153	1781.818261	-0.529050736	0.0001015	0.0012771	Adk	14	21052574	21448569	+	8526	protein_coding	adenosine kinase [Source:MGI Symbol;Acc:MGI:87930]	-
ENSMUSG0000004121	61.128239	84.26135969	53.62361449	156.7622576	136.1295477	159.5761711	66.33773772	150.8779686	-1.184966911	0.0001017	0.0012793	Arhgap11a	2	113831492	113848661	-	5332	protein_coding	Rho GTPase activating protein 11A [Source:MGI Symbol;Acc:MGI:2444300]	-
ENSMUSG0000003537	160.3363646	101.1136316	175.3939057	301.46588	282.4674378	322.0714186	145.614634	302.0015788	-1.052890409	0.0001026	0.0012894	1810055G02Ri	19	3708333	3717881	+	1882	protein_coding	RIKEN cDNA 1810055G02 gene [Source:MGI Symbol;Acc:MGI:1919306]	-
ENSMUSG0000006225	24.05045469	13.69247095	8.937269082	49.09587188	67.16009009	72.97690753	15.56006491	63.07762317	-2.01040088	0.0001037	0.0013017	Opcml	9	27790775	28925410	+	14519	protein_coding	opioid binding protein/cell adhesion molecule-like [Source:MGI Symbol;Acc:MGI:97397]	-
ENSMUSG0000000342	4494.42872	4552.219957	4179.290454	3278.226112	2974.796932	2835.396114	4408.646377	3029.473053	0.541195873	0.0001055	0.0013238	Rps11	7	45122379	45124439	-	865	protein_coding	ribosomal protein S11 [Source:MGI Symbol;Acc:MGI:1351329]	-
ENSMUSG0000004848	5988.563217	4697.570803	5869.551469	3168.837064	3350.103318	3892.101735	5518.56183	3470.347372	0.669272537	0.000106	0.0013287	Fitm2	2	163466379	163472629	-	3967	protein_coding	fat storage-inducing transmembrane protein 2 [Source:MGI Symbol;Acc:MGI:2444508]	-
ENSMUSG0000003237	1.002102279	0	2.23431727	55.98652056	10.86413222	17.51445781	1.078806516	28.12170353	-4.728119109	0.0001061	0.0013296	Car12	9	66713686	66766845	+	7597	protein_coding	carbonic anhydrase 12 [Source:MGI Symbol;Acc:MGI:1923709]	-
ENSMUSG0000006983	14420.25179	11142.51155	16574.16551	19395.45339	28590.44541	30308.76924	14045.64295	26098.22268	-0.893823799	0.0001072	0.0013427	Ahnak	19	8989284	9076919	+	18543	protein_coding	AHNAK nucleoprotein (desmoyokin) [Source:MGI Symbol;Acc:MGI:1316648]	-
ENSMUSG0000004891	316.66432	313.8735648	137.4105121	436.6948604	786.1681135	793.0157285	255.982799	671.9595675	-1.391173174	0.0001073	0.0013435	Rnf24	2	131298064	131352892	-	6801	protein_coding	ring finger protein 24 [Source:MGI Symbol;Acc:MGI:1261771]	-
ENSMUSG0000004631	740.5535839	811.015587	945.1162054	521.1053068	512.5895112	529.325836	832.2284588	521.0068846	0.675285115	0.0001087	0.0013598	Zfp62	11	49203292	49218816	+	6060	protein_coding	zinc finger protein 62 [Source:MGI Symbol;Acc:MGI:99662]	zf-C2
ENSMUSG0000005055	645.3538674	411.8273955	270.3523897	736.4380782	1227.646941	1343.748124	442.5112175	1102.611048	-1.316310981	0.0001098	0.0013719	Kcnb1	2	167095969	167190155	-	11488	protein_coding	potassium voltage gated channel, Shab-related subfamily, member 1 [Source:MGI Symbol;Acc:MGI:96666]	-
ENSMUSG0000009393	2385.003423	2302.441653	1452.306226	3102.51457	4754.539319	4112.005483	2046.583767	3989.686458	-0.962841529	0.0001101	0.0013757	Hmgcs1	13	119690379	119708260	+	4594	protein_coding	3-hydroxy-3-methylglutaryl-Coenzyme A synthase 1 [Source:MGI Symbol;Acc:MGI:107592]	-
ENSMUSG0000006143	1377.890633	1089.078074	398.8256328	1611.550461	3110.10476	4394.182859	955.2647799	3038.612694	-1.669155124	0.0001113	0.0013899	Hipk2	6	38694390	38876165	-	8836	protein_coding	homeodomain interacting protein kinase 2 [Source:MGI Symbol;Acc:MGI:1314872]	-
ENSMUSG0000003461	510.0700598	555.0717069	825.5802314	310.9405219	337.7757472	346.3970544	630.2406661	331.7044412	0.925703375	0.0001126	0.0014045	Chst11	10	82985498	83195900	+	5527	protein_coding	carbohydrate sulfotransferase 11 [Source:MGI Symbol;Acc:MGI:1927166]	-
ENSMUSG0000002800	7.01471595	12.63920395	46.92066268	0.861331086	0	0	22.19152753	0.287110362	6.040806139	0.000113	0.0014082	Fga	3	83026076	83033627	+	3010	protein_coding	fibrinogen alpha chain [Source:MGI Symbol;Acc:MGI:1316726]	-
ENSMUSG0000002405	475.9985823	644.5994016	742.9104924	310.9405219	383.2075729	269.5280452	621.1694921	321.22538	0.951177296	0.0001142	0.0014225	Cyp4f13	17	32924688	32947402	-	3330	protein_coding	cytochrome P450, family 4, subfamily f, polypeptide 13 [Source:MGI Symbol;Acc:MGI:2158641]	-
ENSMUSG0000006428	760.5956295	784.6839121	1057.949228	1198.11154	1839.988939	2038.488284	867.742923	1692.196254	-0.963590179	0.0001145	0.0014257	Tank	2	61578585	61654171	+	6096	protein_coding	TRAF family member-associated Nf-kappa B activator [Source:MGI Symbol;Acc:MGI:107676]	-

ENSMUSG0000001890	1009.116995	1090.131341	1355.113425	683.896882	763.4522006	607.1678707	1151.45392	684.8389844	0.749393742	0.0001151	0.0014323	Slc22a5	11	53864542	53891660	-	3071	protein_coding	solute carrier family 22 (organic cation transporter), member 5 [Source:MGI Symbol;Acc:MGI:1329012]	-
ENSMUSG0000011065	55.11562532	73.72868973	48.03782131	18.0879528	19.75296767	12.64933064	58.96071212	16.8300837	1.808234866	0.0001155	0.0014359	Gm20735	8	122445268	122451322	-	1979	lincRNA	predicted gene, 20735 [Source:MGI Symbol;Acc:MGI:5434091]	-
ENSMUSG0000010425	88.18500052	50.55681581	94.95848399	24.97860148	27.65415474	18.48748324	77.90010011	23.70674649	1.715507705	0.0001156	0.0014362	Gm37174	1	121328092	121331149	+	3058	TEC	predicted gene, 37174 [Source:MGI Symbol;Acc:MGI:5610402]	-
ENSMUSG0000005037	40.08409114	60.03621878	54.74077313	12.91996628	15.80237414	4.865127169	51.62036102	11.19582253	2.202411023	0.0001159	0.0014394	Ch25h	19	34473786	34475135	-	1350	protein_coding	cholesterol 25-hydroxylase [Source:MGI Symbol;Acc:MGI:1333869]	-
ENSMUSG0000004074	1168.451257	1205.990711	1202.062691	855.301768	803.9457844	760.9058892	1192.16822	806.7178139	0.56314959	0.0001161	0.0014411	Rnf167	11	70647235	70651421	+	3539	protein_coding	ring finger protein 167 [Source:MGI Symbol;Acc:MGI:1917760]	-
ENSMUSG0000011279	42.0882957	28.43820889	44.68634541	150.73294	78.02422232	108.0058231	38.40428334	112.2543285	-1.549678732	0.0001164	0.0014423	Gm48878	10	122454132	122456809	+	2678	TEC	predicted gene, 48878 [Source:MGI Symbol;Acc:MGI:6098630]	-
ENSMUSG0000002608	1869.922852	1525.13061	1779.633706	1124.898398	1177.276873	1110.22202	1724.895723	1137.465764	0.600764407	0.0001164	0.0014423	Mrpl30	1	37890477	37898535	+	3226	protein_coding	mitochondrial ribosomal protein L30 [Source:MGI Symbol;Acc:MGI:1333820]	-
ENSMUSG0000002919	1911.009045	1605.178902	1443.368957	1026.706654	1081.47498	1040.164189	1653.185635	1049.448608	0.655905108	0.0001165	0.0014431	Grpel1	5	36464998	36474080	+	3528	protein_coding	GrpE-like 1, mitochondrial [Source:MGI Symbol;Acc:MGI:1334417]	-
ENSMUSG0000002199	563.1814806	266.47655	363.0765564	821.7098557	935.3030194	778.420347	397.5781957	845.1444074	-1.087353866	0.0001169	0.0014469	Wnt5a	14	28504750	28527448	+	7823	protein_coding	wingless-type MMTV integration site family, member 5A [Source:MGI Symbol;Acc:MGI:98958]	-
ENSMUSG0000003155	157.3300577	114.8061026	70.38099402	485.7907323	221.233238	269.5280452	114.1723848	325.5173385	-1.510723718	0.0001172	0.0014489	Slit2	5	47983138	48307733	+	25433	protein_coding	slit guidance ligand 2 [Source:MGI Symbol;Acc:MGI:1315205]	-
ENSMUSG0000003627	723.5178452	778.3643101	394.3569982	338.5031166	286.4180313	228.6609769	632.0797178	284.527375	1.151366644	0.0001181	0.0014593	Macrod1	19	7056768	7198061	+	1274	protein_coding	MACRO domain containing 1 [Source:MGI Symbol;Acc:MGI:2147583]	-
ENSMUSG0000000469	84.1765914	51.61008281	61.44372494	120.586352	189.6284897	176.1176035	65.74346638	162.1108151	-1.299303829	0.0001204	0.0014865	Hdac9	12	34047580	34917095	-	14665	protein_coding	histone deacetylase 9 [Source:MGI Symbol;Acc:MGI:1931221]	-
ENSMUSG0000005033	358.7526157	280.169021	213.3772993	427.2202185	812.8346198	660.6842695	284.0996453	633.5797026	-1.155899048	0.0001205	0.0014874	Amer1	X	95420318	95444872	-	8496	protein_coding	APC membrane recruitment 1 [Source:MGI Symbol;Acc:MGI:1919595]	-
ENSMUSG0000002647	537.1268213	569.8174449	334.0304319	874.2510519	901.7229744	924.3741621	480.3248994	900.1160628	-0.90542902	0.000122	0.0015048	Mr1	1	155127277	155146814	-	5981	protein_coding	major histocompatibility complex, class I-related [Source:MGI Symbol;Acc:MGI:1195463]	-
ENSMUSG0000009011	1432.004156	1681.014126	1085.878193	569.3398476	832.5875875	819.2874152	1399.632158	740.4049501	0.919223628	0.0001222	0.0015067	Shprh	10	11149427	11217595	+	14066	protein_coding	SNF2 histone linker PHD RING helicase [Source:MGI Symbol;Acc:MGI:1917581]	-
ENSMUSG0000002877	1088.283075	859.4658688	1060.183545	1586.57186	1462.707256	1694.03728	1002.644163	1581.105465	-0.657129295	0.0001226	0.0015096	Pef1	4	130102558	130128135	+	1915	protein_coding	penta-EF hand domain containing 1 [Source:MGI Symbol;Acc:MGI:1915148]	-
ENSMUSG0000007625	2794.863255	2846.98069	2665.540504	1927.65897	844.4393681	893.2373482	2769.12815	1221.778562	1.180057542	0.0001232	0.0015169	Gm23935	16	11144125	11144181	+	57	miRNA	predicted gene, 23935 [Source:MGI Symbol;Acc:MGI:5453712]	-
ENSMUSG0000002616	1.002102279	1.053266996	2.23431727	22.39460823	11.8517806	36.97496648	1.429895515	23.74045177	-4.068260739	0.0001234	0.0015182	Sphkap	1	83254139	83408200	-	9602	protein_coding	SPHK1 interactor, AKAP domain containing [Source:MGI Symbol;Acc:MGI:1924879]	-
ENSMUSG0000002401	499.0469348	674.0908775	965.2250608	279.0712717	411.849376	299.6918336	712.7876244	330.2041605	1.110184009	0.0001238	0.0015223	Pim1	17	29490812	29496111	+	2638	protein_coding	proviral integration site 1 [Source:MGI Symbol;Acc:MGI:97584]	-
ENSMUSG0000002046	3120.546496	4928.236275	3274.39196	1820.853915	1265.17758	2368.343906	3774.391577	1818.125133	1.053768734	0.0001243	0.0015271	Rps27a	11	29545846	29548109	-	1972	protein_coding	ribosomal protein S27A [Source:MGI Symbol;Acc:MGI:1925544]	-
ENSMUSG0000001952	2597.449106	4283.636873	1749.470423	1097.335803	1369.868308	1382.669141	2876.852134	1283.291084	1.164789211	0.0001251	0.0015358	Gyg	3	20122084	20155317	-	2344	protein_coding	glycogenin [Source:MGI Symbol;Acc:MGI:1351614]	-
ENSMUSG0000002383	977.0497217	965.8458354	1143.970442	1551.257285	2078.999848	1527.649931	1028.955333	1719.302355	-0.740663693	0.0001255	0.0015397	Acat2	17	12942890	12960747	-	2544	protein_coding	acetyl-Coenzyme A acetyltransferase 2 [Source:MGI Symbol;Acc:MGI:87871]	-
ENSMUSG0000008533	390.8198887	548.752105	720.5673197	308.3565286	264.6897668	234.4991295	553.3797711	269.1818083	1.038565364	0.0001264	0.0015496	Gm12940	4	127033731	127049398	+	2680	processed_transcrip	predicted gene 12940 [Source:MGI Symbol;Acc:MGI:3702626]	-
ENSMUSG0000000895	674.4148335	705.6888874	567.5165867	443.5855091	348.6398795	342.5049527	649.2067692	378.2434471	0.778752081	0.0001265	0.0015496	Vps72	3	95111022	95123051	+	3639	protein_coding	vacuolar protein sorting 72 [Source:MGI Symbol;Acc:MGI:1202305]	-
ENSMUSG0000002896	2162.536717	2327.720061	2196.333877	1173.132939	1579.249766	1478.998659	2228.863552	1410.460455	0.660461916	0.0001277	0.0015632	Ube4b	4	149328416	149426749	-	7078	protein_coding	ubiquitination factor E4B [Source:MGI Symbol;Acc:MGI:1927086]	-
ENSMUSG0000003280	387.8135818	483.4495512	301.6328315	758.8326864	685.4279783	677.2257019	390.9653215	707.1621222	-0.854692115	0.0001277	0.0015632	Srxn1	2	152105516	152111376	+	2820	protein_coding	sulfiredoxin 1 homolog (S. cerevisiae) [Source:MGI Symbol;Acc:MGI:104971]	-
ENSMUSG0000009913	48.10090937	35.81107787	58.09224903	97.33041267	125.4313447	132.331459	47.33474542	118.3644055	-1.3226631	0.0001288	0.0015757	Gm10603	7	100446737	100474846	-	6938	antisense	predicted gene 10603 [Source:MGI Symbol;Acc:MGI:3642592]	-
ENSMUSG0000003745	1005.108585	829.9743929	984.2167576	1378.129737	1438.016047	1464.403278	939.7665787	1426.849687	-0.602368092	0.0001301	0.0015902	Azin1	15	38487427	38519266	-	6256	protein_coding	antizyme inhibitor 1 [Source:MGI Symbol;Acc:MGI:1859169]	-
ENSMUSG0000001031	66.13875039	56.87641779	29.04612452	14.64262846	9.876483837	8.757228904	50.68709756	11.09211373	2.187973081	0.000131	0.0016001	Optc	1	133897199	133907999	-	2575	protein_coding	opticin [Source:MGI Symbol;Acc:MGI:2151113]	-
ENSMUSG0000008991	529.1100031	663.5582075	693.7555125	397.0736305	378.269331	358.0733596	628.8079077	377.8054404	0.734276329	0.0001314	0.0016041	Uckl1	2	181569149	181584892	-	4629	protein_coding	uridine-cytidine kinase 1-like 1 [Source:MGI Symbol;Acc:MGI:1915806]	-
ENSMUSG0000003212	1988.170921	2920.70938	2169.52207	1332.479189	1513.077324	1355.424429	2359.467457	1400.326981	0.752756641	0.0001315	0.0016045	Robo4	9	37401897	37415115	+	5302	protein_coding	roundabout guidance receptor 4 [Source:MGI Symbol;Acc:MGI:1921394]	-
ENSMUSG0000004351	226.475115	226.4524042	214.494458	124.0316763	131.357235	105.0867468	222.4739924	120.1585527	0.88865541	0.0001333	0.0016254	Hscb	5	110829070	110839777	-	905	protein_coding	HscB iron-sulfur cluster co-chaperone [Source:MGI Symbol;Acc:MGI:2141135]	-
ENSMUSG0000008728	17.03573874	46.34374783	37.9833936	4.306655428	5.925890302	4.865127169	33.78762672	5.032557633	2.749544882	0.0001334	0.0016254	Gm16015	5	43909215	43909885	-	390	antisense	predicted gene 16015 [Source:MGI Symbol;Acc:MGI:3801811]	-
ENSMUSG0000003054	22017.18916	10865.50233	6561.072665	25613.40249	40124.20324	41235.84486	13147.92139	35657.81686	-1.439356253	0.0001337	0.0016267	Plin1	7	79720218	79732903	-	4649	protein_coding	perilipin 1 [Source:MGI Symbol;Acc:MGI:1890505]	-
ENSMUSG0000003775	136.2859099	196.9609283	155.2850503	63.73850033	76.04892555	83.6801873	162.8439628	74.4892044	1.129739424	0.0001337	0.0016267	Ptger2	14	44988195	45003820	+	5193	protein_coding	prostaglandin E receptor 2 (subtype EP2) [Source:MGI Symbol;Acc:MGI:97794]	-
ENSMUSG0000005285	85.17869368	88.47442767	62.56088357	264.4286433	128.3942899	203.3623157	78.73800164	198.7284163	-1.335745331	0.0001341	0.0016306	Reep1	6	71707561	71810710	+	4112	protein_coding	receptor accessory protein 1 [Source:MGI Symbol;Acc:MGI:1098827]	-
ENSMUSG0000001889	1118.346143	1210.203779	1424.37726	721.7954497	874.0688196	715.1736938	1250.975727	770.3459877	0.699477437	0.0001353	0.0016438	Irf1	11	53770014	53778374	+	3976	protein_coding	interferon regulatory factor 1 [Source:MGI Symbol;Acc:MGI:96590]	IRF
ENSMUSG0000002233	3268.857633	3042.888352	3551.447301	2279.943384	2064.185122	2344.991295	3287.731095	2229.7066	0.560137273	0.0001359	0.0016507	Eif3e	15	43250058	43282719	-	6719	protein_coding	eukaryotic translation initiation factor 3, subunit E [Source:MGI Symbol;Acc:MGI:99257]	-
ENSMUSG0000002801	436.9165935	301.2343609	194.3856025	632.2170168	723.9462653	667.4954476	310.845519	674.5529099	-1.116539223	0.0001369	0.0016608	Pdgfc	3	81036416	81214040	+	5089	protein_coding	platelet-derived growth factor, C polypeptide [Source:MGI Symbol;Acc:MGI:1859631]	-
ENSMUSG0000007396	732.5367657	620.3742607	1052.363434	410.8549278	473.0835758	352.235207	801.7581536	412.0579036	0.960058998	0.000137	0.0016612	Trim68	7	102677582	102687327	-	3477	protein_coding	tripartite motif-containing 68 [Source:MGI Symbol;Acc:MGI:2142077]	-
ENSMUSG0000003594	1274.674098	1582.007028	2475.623536	851.8564436	961.9695258	889.3452465	1777.434887	901.057072	0.97995915	0.0001379	0.0016713	Ibtk	9	85687360	85749334	-	8628	protein_coding	inhibitor of Bruton agammaglobulinemia tyrosine kinase [Source:MGI Symbol;Acc:MGI:1918677]	-
ENSMUSG0000000961	602.2634694	407.6143275	299.3985142	831.1844976	1002.46311	847.5051528	436.4254371	893.7175866	-1.033174627	0.0001384	0.0016764	Sardh	2	27188393	27248337	-	5432	protein_coding	sarcosine dehydrogenase [Source:MGI Symbol;Acc:MGI:2183102]	-
ENSMUSG0000005554	43.09039798	63.19601977	25.69464861	97.33041267	150.1225543	143.0347388	43.99368879	130.1625686	-1.561822763	0.0001405	0.0017	Lair1	7	4003402	4063204	-	7475	protein_coding	leukocyte-associated Ig-like receptor 1 [Source:MGI Symbol;Acc:MGI:105492]	-
ENSMUSG0000003457	1.002102279	3.159800988	4.468634541	100.775737	19.75296767	9.730254338	2.876845936	43.41965301	-3.931009438	0.0001413	0.0017089	Inpp5j	11	3494375	3504821	-	5092	protein_coding	inositol polyphosphate 5-phosphatase J [Source:MGI Symbol;Acc:MGI:2158663]	-
ENSMUSG0000003016	14.0294319	20.01207293	14.52306226	74.07447336	56.29595787	43.78614452	16.18818903	58.05219192	-1.844281143	0.0001416	0.0017121	Klrd1	6	129591782	129598775	+	1281	protein_coding	killer cell lectin-like receptor, subfamily D, member 1 [Source:MGI Symbol;Acc:MGI:1196275]	-
ENSMUSG0000003065	3968.325023	3798.080788	4333.458346	2694.243636	2905.661545	2837.342165	4033.288052	2812.415782	0.52016256	0.0001421	0.001717	Arl6ip1	7	118118891	118129662	-	2482	protein_coding	ADP-ribosylation factor-like 6 interacting protein 1 [Source:MGI Symbol;Acc:MGI:1858943]	-
ENSMUSG0000000529	1082.270461	1242.855055	919.4215568	1622.747765	1760.977068	1747.553679	1081.515691	1710.426171	-0.661027144	0.0001431	0.0017281	Letm1	5	33739673	33782817	-	9650	protein_coding	leucine zipper-EF-hand containing transmembrane protein 1 [Source:MGI Symbol;Acc:MGI:1932557]	-
ENSMUSG0000002587	937.9677328	1128.048953	1234.460292	686.4808752	710.1191879	687.9289817	1100.158993	694.8430149	0.662695923	0.0001433	0.0017289	Uimc1	13	55027880	55100300	-	5085	protein_coding	ubiquitin interaction motif containing 1 [Source:MGI Symbol;Acc:MGI:103185]	-
ENSMUSG0000003192	644.3517652	649.8657366	661.3579121	450.4761578	421.7258599	408.6706822	651.8584713	426.9575666	0.610024208	0.0001435	0.0017303	Terf2	8	107069400	107096547	-	4597	protein_coding	telomeric repeat binding factor 2 [Source:MGI Symbol;Acc:MGI:1195972]	MYB
ENSMUSG0000003117	8.016818229	7.372868973	6.702951811	230.8367309	6.913538686	36.97496648	7.364213004	91.5750787	-3.637050041	0.000144	0.0017346	Slc38a5	X	8271133	8280179	+	2241	protein_coding	solute carrier family 38, member 5 [Source:MGI Symbol;Acc:MGI:2148066]	-
ENSMUSG0000003457	624.3097196	565.6043769	341.8505424	832.0458287	1330.362373	1041.137214	510.588213	1067.848472	-1.063710799	0.0001442	0.0017362	Daam1	12	71831078	71992333	+	8096	protein_coding	dishevelled associated activator of morphogenesis 1 [Source:MGI Symbol;Acc:MGI:1914596]	-
ENSMUSG0000002802	1683.531828	1872.708719	1274.678003	1920.768321	6500.701662	4250.175095	1610.306183	4223.881692	-1.391105462	0.0001452	0.0017473	Enpep	3	129269175	129332720	-	4706	protein_coding	glutamyl aminopeptidase [Source:MGI Symbol;Acc:MGI:106645]	-
ENSMUSG0000000233	436.9165935	452.9048083	437.926185	842.3818017	620.243185	722.9578973	442.5825289	728.527628	-0.71959419	0.000146	0.0017562	Dhrs1	14	55739020	55745690	-	2760	protein_coding	dehydrogenase/reductase (SDR family) member 1 [Source:MGI Symbol;Acc:MGI:1196314]	-
ENSMUSG0000002678	568.191992	564.5511099	624.4916771	409.9935967	321.9733731	296.7727573	585.7449263	342.9132424	0.77120796	0.0001463	0.0017581	Pkn3	2	30077684	30091022	+	4255	protein_coding	protein kinase N3 [Source:MGI Symbol;Acc:MGI:2388285]	-
ENSMUSG0000000657	248.5213651	180.1086563	198.8542371	482.3454079	351.6028246	359.0463851	209.1614195	397.6648725	-0.927013176	0.0001471	0.0017669	Slc4a3	1	75546266	75562172	+	8599	protein_coding	solute carrier family 4 (anion exchanger), member 3 [Source:MGI Symbol;Acc:MGI:109350]	-
ENSMUSG0000003179	453.9523322	325.4595018	331.7961147	629.6330236	680.4897364	610.086947	370.4026496	640.0699023	-0.788323289	0.0001472	0.0017672	Mmp15	8	95352268	95375080	+	5397	protein_coding	matrix metallopeptidase 15 [Source:MGI Symbol;Acc:MGI:109320]	-
ENSMUSG0000002681	8767.392836	5426.431564	5353.42418	3903.55248	3517.015895	3285.90689	6515.749527	3568.825088	0.868500386	0.000148	0.001775	Eng	2	32646595	32682669	+	4048	protein_coding	endoglin [Source:MGI Symbol;Acc:MGI:95392]	-
ENSMUSG0000002920	15.03153418	24.22514091	21.22601407	68.04515576	56.29595787	65.19270406	20.16089639	63.17793923	-1.651076293	0.0001481	0.0017752	Rhoh	5	65861213	65896700	+	10341	protein_coding	ras homolog family member H [Source:MGI Symbol;Acc:MGI:1921984]	-
ENSMUSG0000005824	53.11142077	73.72868973	21.22601407	10.33597303	0	2.919076301	49.35537485	4.418349776	3.456962644	0.0001482	0.0017752	Kcnh1	1	192190774	192510159	+	7331	protein_coding	potassium voltage-gated channel, subfamily H (eag-related), member 1 [Source:MGI Symbol;Acc:MGI:1341721]	-
ENSMUSG0000002275	36.07568203	44.23721384	11.17158635	95.6077505	152.0978511	96.32951794	30.49482741	114.6783732	-1.906388032	0.0001484	0.001777	P2rx6	16	17561885	17577800	+	3995	protein_coding	purinergic receptor P2X, ligand-gated ion channel, 6 [Source:MGI Symbol;Acc:MGI:1337113]	-
ENSMUSG0000002106	1653.46876	1822.151903	2063.391999	853.5791058	1156.536257	1243.526504	1846.337554	1084.547289	0.767829606	0.000149	0.0017823	Sav1	12	69965012	69987002	-	5265	protein_coding	salvador family WW domain containing 1 [Source:MGI Symbol;Acc:MGI:1927144]	-
ENSMUSG0000003639	4077.554172	894.2236797	2392.953797	5564.198813	8039.457844	8476.024554	2454.910549	7359.893737	-1.5839452	0.0001501	0.001794	Glb1l2	9	26763044	26806468	-	9043	protein_coding	galactosidase, beta 1-like 2 [Source:MGI Symbol;Acc:MGI:2388283]	-
ENSMUSG0000002086	137.2880122	155.8835154	82.66973901	259.2606568	276.5415474	266.6089689	125.2804222	267.470391	-1.092464743	0.0001504	0.0017967	Cacna1g	11	94408391	94474198	-	10137	protein_coding	calcium channel, voltage-dependent, T type, alpha 1G subunit [Source:MGI Symbol;Acc:MGI:1201678]	-
ENSMUSG0000002868	126.2648871	174.8423214	213.3772993	77.5197977	77.03657393	76.86900927	171.4948359	77.14179363	1.151241962	0.0001505	0.0017967	Ccdc163	4	116708509	116751206	+	2519	protein_coding	coiled-coil domain containing 163 [Source:MGI Symbol;Acc:MGI:1915644]	-
ENSMUSG0000004256	6.012613672	2.106533992	2.23431727	50.81853405	22.71591283	20.43353411	3.451154978	31.32266033	-3.172067627	0.0001515	0.0018073	Nek10	14	14803415	15012059	+	11849	protein_coding	NIMA (never in mitosis gene a)- related kinase 10 [Source:MGI Symbol;Acc:MGI:2685128]	-
ENSMUSG0000004709	15.03153418	42.13067984	10.05442772	0	0.987648384	0.973025434	22.40554725	0.653557939	5.146235601	0.0001526	0.0018184	Ofcc1	13	40001882	40361450	-	3575	protein_coding	orofacial cleft 1 candidate 1 [Source:MGI Symbol;Acc:MGI:2658851]	-
ENSMUSG0000002058	3787.946613	3201.931668	4301.060746	4981.077668	7585.139587	7378.451864	3763.646342	6648.22304	-0.820806667	0.0001526	0.0018184	Rock2	12	16894895	16987823	+	13897	protein_coding	Rho-associated coiled-coil containing protein kinase 2 [Source:MGI Symbol;Acc:MGI:107926]	-
ENSMUSG0000002336	1541.233305	2389.862814	2037.697351	1333.34052	862.217039	718.0927701	1989.597823	971.2167765	1.034154819	0.0001529	0.0018205	Tmem176a	6	48840919	48847071	+	3810	protein_coding	transmembrane protein 176A [Source:MGI Symbol;Acc:MGI:1913308]	-
ENSMUSG0000003596	1535.220691	1318.690279	1396.448294	896.6456601	1022.216077	827.0716187	1416.786421	915.3111187	0.630480985	0.0001531	0.0018223	Rusc2	4	43381979	43427088	+	7923	protein_coding	RUN and SH3 domain containing 2 [Source:MGI Symbol;Acc:MGI:2140371]	-
ENSMUSG0000001880	445.935514	510.8344931	541.8219381	309.2178597	301.232757	316.233266	499.5306484	308.8946276	0.692984912	0.0001535	0.0018255	Abca5	11	110269369	110337716	-	11437	protein_coding	ATP-binding cassette, sub-family A (ABC1), member 5 [Source:MGI Symbol;Acc:MGI:2386607]	-
ENSMUSG0000004043	922.9361986	901.5965487	1244.51472	602.0704288	618.2678882	392.1292498	1023.015822	537.489189	0.928083954	0.0001537	0.0018269	Ppp1r15a	7	45522916	45526268	-	2937	protein_coding	protein phosphatase 1, regulatory subunit 15A [Source:MGI Symbol;Acc:MGI:1927072]	-
ENSMUSG0000003031	782.6418796	861.5724028	777.5424101	359.1750627	541.2313143	493.3238949	807.2522308	464.5767573	0.798006731	0.0001539	0.0018278	Dennd5b	6	148988071	149101680	-	18081	protein_coding	DENN/MADD domain containing 5B [Source:MGI Symbol;Acc:MGI:2444273]	-
ENSMUSG0000001746	8423.671754	8257.613249	4623.919591	14752.87883	12313.0124	13392.72207	7101.734865	13486.20443	-0.925201074	0.000154	0.001828	Timp2	11	118301069	118355740	-	3896	protein_coding	tissue inhibitor of metalloproteinase 2 [Source:MGI Symbol;Acc:MGI:98753]	-
ENSMUSG0000002047	651.3664811	718.3280913	527.2988758	416.8842454	337.7757472	342.5049527	632.3311494	365.7216485	0.789538582	0.0001543	0.0018305	Zkscan17	11	59485520	59526751	-	4573	protein_coding	zinc finger with KRAB and SCAN domains 17 [Source:MGI Symbol;Acc:MGI:2679270]	zf-C2
ENSMUSG0000008691	198.4162512	157.9900494	187.6826507	68.04515576	107.6536738	73.94993297	181.3629838	83.21625419	1.126210291	0.0001545	0.0018321	Gm16124	9	53667246	53681265	+	516	antisense	predicted gene 16124 [Source:MGI Symbol;Acc:MGI:3802001]	-
ENSMUSG0000007424	872.8310847	829.9743929	856.8606732	1204.140858	1214.807512	1331.098793	853.2220503	1250.015721	-0.550825468	0.0001548	0.0018347	Dda1	8	71469199	71476098	+	2846	protein_coding	DET1 and DDB1 associated 1 [Source:MGI Symbol;Acc:MGI:1913748]	-
ENSMUSG0000002520	898.8857439	3020.769745	367.545191	11106.86435	13518.93108	12392.45192	1429.066893	12339.41578	-3.110019518	0.0001549	0.0018348	Scd2	19	44293676	44306864	+	5453	protein_coding	stearoyl-Coenzyme A desaturase 2 [Source:MGI Symbol;Acc:MGI:98240]	-
ENSMUSG0000003014	31.06517064	8.426135969	6.702951811	95.6077505	60.24655141	74.9229584	15.39808614	76.92575344	-2.312308152	0.0001555	0.0018401	Clec4b1	6	123049962	123071555	+	784	protein_coding	C-type lectin domain family 4, member b1 [Source:MGI Symbol;Acc:MGI:1917060]	-
ENSMUSG0000002466	423.8892639	385.4957206	248.009217	639.9689966	673.5761977	666.5224221	352.4647338	660.0225388	-0.90401899	0.0001556	0.0018401	Rab3il1	19	10001669	10038380	+	4543	protein_coding	RAB3A interacting protein (rabin3)-like 1 [Source:MGI Symbol;Acc:MGI:1922010]	-
ENSMUSG0000007457	32.06727292	25.27840791	23.46033134	65.4611625	84.937761	75.89598383	26.93533739	75.43163578	-1.481988849	0.0001561	0.0018447	Cass4	2	172393794	172433757	+	5340	protein_coding	Cas scaffolding protein family member 4 [Source:MGI Symbol;Acc:MGI:2444482]	-
ENSMUSG0000004239	1469.08194	1486.159731	1586.365262	1011.202694	1085.425574	1048.921418	1513.869978	1048.516562	0.529932069	0.0001568	0.0018516	Rbm7	9	48488701	48495299	-	4941	protein_coding	RNA binding motif protein 7 [Source:MGI Symbol;Acc:MGI:1914260]	-
ENSMUSG0000002839	1280.686712	977.4317724	1049.011958	1978.477504	1615.792756	1728.09317	1102.376814	1774.121143	-0.686443441	0.0001569	0.0018516	Alad	4	62509169	62519918	-	1871	protein_coding	aminolevulinate, delta-, dehydratase [Source:MGI Symbol;Acc:MGI:96853]	-
ENSMUSG0000000206	13.02732962	18.95880593	13.40590362	61.15450708	36.5429902	80.76111	15.13067972	59.48620276	-1.975504391	0.0001578	0.0018622	Ccne1	7	38097984	38107534	-	2662	protein_coding	cyclin E1 [Source:MGI Symbol;Acc:MGI:88316]	-
ENSMUSG0000003069	44.09250026	44.23721384	92.72416672	15.50395954	16.79002252	13.62235607	60.35129361	15.30544605	1.97714353	0.0001581	0.0018642	Tbx6	7	126781483	126785560	+	1804	protein_coding	T-box 6 [Source:MGI Symbol;Acc:MGI:102539]	T-bo
ENSMUSG0000002692	2744.758141	2933.348584	2535.950102	2028.434707	1621.718646	1472.187481	2738.018942	1707.446945	0.681067455	0.0001592	0.0018757	Egfl7	2	26580014	26593120	+	3581	protein_coding	EGF-like domain 7 [Source:MGI Symbol;Acc:MGI:2449923]	-
ENSMUSG0000001397	33.06937519	41.07741285	88.25553218	12.91996628	8.888835454	12.64933064	54.13410674	11.48604413	2.231665036	0.0001595	0.0018785	Mcemp1	8	3665754	3669259	+	1494	protein_coding	mast cell expressed membrane protein 1 [Source:MGI Symbol;Acc:MGI:1916439]	-
ENSMUSG0000002145	82.17238685	82.1548257	49.15497995	217.9167647	122.4683996	203.3623157	71.16073083	181.24916	-1.347732114	0.0001613	0.001898	Sema4d	13	51685529	51793747	-	7679	protein_coding	sema domain, immunoglobulin domain (Ig), transmembrane domain (TM) and short cytoplasmic domain, (semaphorin) 4D [Source	-
ENSMUSG0000003722	360.7568203	389.7087886	320.6245283	603.793091	540.2436659	572.1389551	357.0300457	572.0585707	-0.679984992	0.0001626	0.0019117	Mospd3	5	137596645	137601058	-	1839	protein_coding	motile sperm domain containing 3 [Source:MGI Symbol;Acc:MGI:1916179]	-
ENSMUSG0000002433	674.4148335	605.6285228	653.5378016	441.0015158	375.3063858	416.4548857	644.5270526	410.9209291	0.648921426	0.0001627	0.0019123	Psmb8	17	34197721	34201454	+	2736	protein_coding	proteasome (prosome, macropain) subunit, beta type 8 (large multifunctional peptidase 7) [Source:MGI Symbol;Acc:MGI:1346527	-
ENSMUSG0000002010	823.728073	498.1952892	387.6540464	900.0909844	1395.547166	1700.848458	569.8591362	1332.162203	-1.224439843	0.0001628	0.0019124	Slc16a7	10	125219270	125389465	-	11839	protein_coding	solute carrier family 16 (monocarboxylic acid transporters), member 7 [Source:MGI Symbol;Acc:MGI:1330284]	-
ENSMUSG0000003789	118.2480689	90.58096166	46.92066268	192.0768321	249.8750411	214.0655954	85.24989774	218.6724895	-1.355810615	0.0001632	0.0019156	Pcdh18	3	49743296	49757325	-	5366	protein_coding	protocadherin 18 [Source:MGI Symbol;Acc:MGI:1920423]	-
ENSMUSG0000003111	1.002102279	0	1.117158635	30.146588	7.90118707	32.10983931	0.706420305	23.38587146	-5.055002653	0.0001633	0.0019156	Igsf1	X	49782536	49797749	-	5768	protein_coding	immunoglobulin superfamily, member 1 [Source:MGI Symbol;Acc:MGI:2147913]	-
ENSMUSG0000003386	3.006306836	16.85227194	24.57748997	0	0	0	14.81202292	0	6.419891392	0.0001646	0.0019296	Fgg	3	83007724	83015049	+	2093	protein_coding	fibrinogen gamma chain [Source:MGI Symbol;Acc:MGI:95526]	-
ENSMUSG0000000889	1152.41762	1193.351507	1236.694609	1771.758043	1700.730517	1653.170212	1194.154579	1708.552924	-0.516992847	0.0001651	0.0019343	Vdac3	8	22577075	22593813	-	5420	protein_coding	voltage-dependent anion channel 3 [Source:MGI Symbol;Acc:MGI:106922]	-
ENSMUSG0000010336	60.12613672	65.30255376	178.7453816	3.445324342	19.75296767	27.24471215	101.3913574	16.81433472	2.598384621	0.0001654	0.0019369	Gm36927	9	85342511	85344351	-	1841	TEC	predicted gene, 36927 [Source:MGI Symbol;Acc:MGI:5610155]	-
ENSMUSG0000004111	58.12193216	38.97087886	53.62361449	147.2876156	116.5425093	102.1676705	50.2388085	121.9992652	-1.280813353	0.0001661	0.0019439	Ccdc8	7	16992708	16997516	+	4809	protein_coding	coiled-coil domain containing 8 [Source:MGI Symbol;Acc:MGI:3612184]	-
ENSMUSG0000010841	419.8808547	391.8153225	735.090382	280.7939339	229.134425	193.6320613	515.5955198	234.5201401	1.13530612	0.0001667	0.0019499	Snhg1	19	8723475	8726443	+	2969	processed_transcrip	small nucleolar RNA host gene 1 [Source:MGI Symbol;Acc:MGI:3763743]	-
ENSMUSG0000004010	647.358072	791.0035141	770.8394583	1031.874641	1210.856918	1270.771217	736.4003481	1171.167592	-0.669392912	0.0001673	0.001955	Plpp6	19	28963953	28966811	+	2859	protein_coding	phospholipid phosphatase 6 [Source:MGI Symbol;Acc:MGI:1921661]	-
ENSMUSG0000004184	4216.846388	3727.511899	3681.037703	2743.339508	2198.505302	2681.658095	3875.131997	2541.167635	0.608695924	0.0001686	0.0019691	Rpl37	15	5116645	5119140	+	1684	protein_coding	ribosomal protein L37 [Source:MGI Symbol;Acc:MGI:1914531]	-
ENSMUSG0000007186	7576.895329	5413.79236	9438.873309	14226.60554	12436.46845	13181.57555	7476.520333	13281.54985	-0.829023605	0.0001689	0.001971	Ppia	11	6413443	6419817	+	3508	protein_coding	peptidylprolyl isomerase A [Source:MGI Symbol;Acc:MGI:97749]	-
ENSMUSG0000003009	1257.63836	966.8991024	825.5802314	601.2090977	616.2925915	548.7863447	1016.705898	588.762678	0.788464441	0.0001692	0.0019743	Xpc	6	91489305	91515888	-	5494	protein_coding	xeroderma pigmentosum, complementation group C [Source:MGI Symbol;Acc:MGI:103557]	-
ENSMUSG0000003720	739.5514816	310.7137638	256.9464861	1150.73833	1052.833177	1130.655554	435.7372439	1111.40902	-1.350153387	0.0001695	0.001976	Islr	9	58156268	58204306	-	2686	protein_coding	immunoglobulin superfamily containing leucine-rich repeat [Source:MGI Symbol;Acc:MGI:1349645]	-
ENSMUSG0000003854	2171.555638	3385.200125	5070.783045	1591.739846	1532.830292	1884.750265	3542.512936	1669.773468	1.085032038	0.0001699	0.0019786	Ranbp9	13	43402675	43481282	-	5130	protein_coding	RAN binding protein 9 [Source:MGI Symbol;Acc:MGI:1928741]	-
ENSMUSG0000003838	371.7799454	351.7911767	351.9049701	622.7423749	505.6759725	599.3836672	358.4920307	575.9304049	-0.684185785	0.0001699	0.0019786	Pigu	2	155278243	155357430	-	9326	protein_coding	phosphatidylinositol glycan anchor biosynthesis, class U [Source:MGI Symbol;Acc:MGI:3039607]	-
ENSMUSG0000008253	534.1205145	497.1420222	464.7379923	306.6338665	253.8256346	331.8016729	498.666843	297.4203913	0.745535399	0.0001702	0.0019793	Gm13456	2	40555752	40557116	+	1365	processed_pseudog	predicted gene 13456 [Source:MGI Symbol;Acc:MGI:3651389]	-
ENSMUSG0000002659	87.18289824	122.1789715	82.66973901	390.1829818	172.8384672	205.3083665	97.3438696	256.1099385	-1.396638659	0.0001702	0.0019793	Ralgps2	1	156804166	156939626	-	12085	protein_coding	Ral GEF with PH domain and SH3 binding motif 2 [Source:MGI Symbol;Acc:MGI:1925505]	-
ENSMUSG0000004170	197.4141489	229.6122051	210.0258234	130.0609939	96.78954161	103.140696	212.3507258	109.9970772	0.946786385	0.0001708	0.0019856	Tmem273	14	32785963	32817984	+	1604	protein_coding	transmembrane protein 273 [Source:MGI Symbol;Acc:MGI:1916319]	-
ENSMUSG0000003065	370.7778431	298.0745599	211.1429821	478.0387525	606.4161076	668.468473	293.331795	584.3077777	-0.992843628	0.000171	0.0019863	Coq7	7	118509659	118533356	-	6434	protein_coding	demethyl-Q 7 [Source:MGI Symbol;Acc:MGI:107207]	-
ENSMUSG0000003790	1303.735064	1055.37353	1233.343133	1802.765962	1721.471133	1909.075901	1197.483909	1811.104332	-0.596808751	0.0001718	0.0019943	Sirpa	2	129592835	129632228	+	4911	protein_coding	signal-regulatory protein alpha [Source:MGI Symbol;Acc:MGI:108563]	-
ENSMUSG0000000884	2692.648823	3405.212198	2022.05713	1094.75181	1618.755701	1556.840694	2706.639384	1423.449402	0.927388631	0.0001734	0.0020113	Cd163	6	124304656	124330527	+	5461	protein_coding	CD163 antigen [Source:MGI Symbol;Acc:MGI:2135946]	-
ENSMUSG0000005243	18.03784102	12.63920395	8.937269082	198.9674808	51.35771595	24.32563584	13.20477135	91.55027752	-2.792191218	0.000174	0.0020173	Bmpr1b	3	141837136	142169425	-	5962	protein_coding	bone morphogenetic protein receptor, type 1B [Source:MGI Symbol;Acc:MGI:107191]	-
ENSMUSG0000002523	569.1940943	529.793299	576.4538558	777.7819703	1006.413703	875.7228904	558.4804164	886.6395212	-0.666498804	0.0001759	0.0020381	Sufu	19	46396896	46488804	+	6945	protein_coding	SUFU negative regulator of hedgehog signaling [Source:MGI Symbol;Acc:MGI:1345643]	-
ENSMUSG0000005829	1732.63484	1347.128488	2110.312662	1051.685256	1036.043155	955.510976	1730.02533	1014.413129	0.769960371	0.0001775	0.0020554	Spock2	10	60106219	60135198	+	5533	protein_coding	sparc/osteonectin, cwcv and kazal-like domains proteoglycan 2 [Source:MGI Symbol;Acc:MGI:1891351]	-
ENSMUSG0000002283	323.679036	411.8273955	349.6706528	220.5007579	216.294996	181.9557561	361.7256948	206.2505034	0.809887224	0.0001778	0.0020572	Iqcb1	16	36828385	36872721	+	5388	protein_coding	IQ calmodulin-binding motif containing 1 [Source:MGI Symbol;Acc:MGI:2443764]	-
ENSMUSG0000001244	12.02522734	22.11860692	29.04612452	96.46908159	48.3947708	87.57228904	21.06331959	77.47871381	-1.885577269	0.0001779	0.0020572	Kif11	19	37376403	37421859	+	4819	protein_coding	kinesin family member 11 [Source:MGI Symbol;Acc:MGI:1098231]	-
ENSMUSG0000004291	119.2501712	153.7769814	284.875452	85.27177747	57.28360626	47.67824626	185.9675349	63.41121	1.548722753	0.0001799	0.0020789	Mamstr	7	45639977	45646521	+	4877	protein_coding	MEF2 activating motif and SAP domain containing transcriptional regulator [Source:MGI Symbol;Acc:MGI:1921740]	-
ENSMUSG0000002080	983.0623353	899.4900147	897.0783841	601.2090977	599.5025689	657.7651932	926.543578	619.4922866	0.581054142	0.0001805	0.0020853	Ube2o	11	116537740	116581447	-	5288	protein_coding	ubiquitin-conjugating enzyme E2O [Source:MGI Symbol;Acc:MGI:2444266]	-
ENSMUSG0000011254	55.11562532	38.97087886	98.3099599	19.81061497	7.90118707	15.56840694	64.13215469	14.42673633	2.144662104	0.0001812	0.0020912	1300014J16Rik	12	80115863	80117199	+	1337	lincRNA	RIKEN cDNA 1300014J16 gene [Source:MGI Symbol;Acc:MGI:1921410]	-
ENSMUSG0000004567	371.7799454	312.8202978	350.7878115	478.0387525	797.0322457	655.8191424	345.1293516	643.6300469	-0.898406221	0.0001821	0.0021007	Pqlc3	12	16988648	17000408	-	5408	protein_coding	PQ loop repeat containing [Source:MGI Symbol;Acc:MGI:2444067]	-
ENSMUSG0000002465	3384.099395	2090.734987	4760.212945	5889.781963	8070.074944	7589.598383	3411.682442	7183.151763	-1.074170087	0.000183	0.0021095	Anxa1	19	20373428	20390944	-	2608	protein_coding	annexin A1 [Source:MGI Symbol;Acc:MGI:96819]	-
ENSMUSG0000004447	2847.974676	2671.085102	2554.941799	3587.443971	3927.877622	4156.764653	2691.333859	3890.695416	-0.53155528	0.0001836	0.0021153	Zfand3	17	30005051	30210828	+	3527	protein_coding	zinc finger, AN1-type domain 3 [Source:MGI Symbol;Acc:MGI:1096572]	-
ENSMUSG0000003939	2.004204557	3.159800988	5.585793176	37.89856777	19.75296767	26.27168671	3.583266241	27.97440738	-2.980454076	0.0001837	0.0021153	Neil3	8	53586867	53639065	-	5555	protein_coding	nei like 3 (E. coli) [Source:MGI Symbol;Acc:MGI:2384588]	-
ENSMUSG0000008988	115.241762	81.1015587	191.0341266	35.31457451	57.28360626	27.24471215	129.1258158	39.94763097	1.693133334	0.0001842	0.0021195	0610040B10Ri	5	143329247	143332704	+	2098	antisense	RIKEN cDNA 0610040B10 gene [Source:MGI Symbol;Acc:MGI:1914922]	-
ENSMUSG0000003294	667.4001176	606.6817898	792.0654724	1059.437235	1209.86927	1035.299622	688.7157932	1101.535189	-0.677752271	0.0001849	0.002127	Rasgrp2	19	6399340	6415216	+	12384	protein_coding	RAS, guanyl releasing protein 2 [Source:MGI Symbol;Acc:MGI:1333849]	-
ENSMUSG0000003306	834.7511981	619.3209937	707.1614161	1001.728053	1548.632666	1311.638285	720.4112026	1287.333001	-0.837032644	0.0001855	0.0021316	Gas7	11	67455437	67688990	+	11342	protein_coding	growth arrest specific 7 [Source:MGI Symbol;Acc:MGI:1202388]	-
ENSMUSG0000005863	1203.524837	1365.034027	1353.996266	863.0537478	907.6488647	901.0215517	1307.518376	890.5747214	0.554000567	0.0001856	0.0021316	Zfp110	7	12834761	12850577	+	4116	protein_coding	zinc finger protein 110 [Source:MGI Symbol;Acc:MGI:1890378]	zf-C2
ENSMUSG0000002220	3205.725189	980.5915734	4668.605937	11245.53865	15550.5238	21004.70004	2951.6409	15933.5875	-2.432497662	0.0001857	0.0021321	Npr3	15	11839896	11907287	-	9389	protein_coding	natriuretic peptide receptor 3 [Source:MGI Symbol;Acc:MGI:97373]	-
ENSMUSG0000000692	2334.898309	2129.705866	2190.748084	1320.420554	1657.273988	1438.131591	2218.450753	1471.942044	0.592113604	0.0001861	0.0021349	Ezh1	11	101191115	101226463	-	7440	protein_coding	enhancer of zeste 1 polycomb repressive complex 2 subunit [Source:MGI Symbol;Acc:MGI:1097695]	-
ENSMUSG0000005824	671.4085267	869.9985388	814.4086451	537.4705974	431.6023437	449.5377504	785.2719035	472.8702305	0.730923562	0.0001869	0.0021432	Cryzl1	16	91689322	91728975	-	4529	protein_coding	crystallin, zeta (quinone reductase)-like 1 [Source:MGI Symbol;Acc:MGI:1913859]	-
ENSMUSG0000003024	23.04835241	96.90056364	39.10055223	86.99443964	425.6764534	241.3103076	53.01648943	251.3270669	-2.244838965	0.0001892	0.0021681	Gys2	6	142422613	142473109	-	2657	protein_coding	glycogen synthase 2 [Source:MGI Symbol;Acc:MGI:2385254]	-
ENSMUSG0000004235	836.7554026	1048.000661	1204.297009	626.1876992	615.3049431	634.4125828	1029.684358	625.3017417	0.719206625	0.0001896	0.0021712	Gnl3	14	31012433	31019152	-	6101	protein_coding	guanine nucleotide binding protein-like 3 (nucleolar) [Source:MGI Symbol;Acc:MGI:1353651]	-
ENSMUSG0000001765	33.06937519	25.27840791	20.10885543	74.93580445	82.96246423	66.1657295	26.15221284	74.68799939	-1.509936885	0.0001898	0.0021723	Cd40	2	165055627	165072948	+	3406	protein_coding	CD40 antigen [Source:MGI Symbol;Acc:MGI:88336]	-
ENSMUSG0000004220	239.5024446	287.5418899	243.5405825	422.913563	404.9358373	435.9153943	256.861639	421.2549316	-0.713761032	0.0001904	0.0021773	Tbc1d22b	17	29549788	29606895	+	4139	protein_coding	TBC1 domain family, member 22B [Source:MGI Symbol;Acc:MGI:2681867]	-
ENSMUSG0000002294	167.3510805	133.7649085	218.9630925	89.5784329	64.19714494	76.86900927	173.3596938	76.88152904	1.170532119	0.0001908	0.0021806	Setd4	16	93583457	93604063	-	5379	protein_coding	SET domain containing 4 [Source:MGI Symbol;Acc:MGI:2136890]	-
ENSMUSG0000006328	375.7883545	460.2776773	359.7250805	543.499915	964.9324709	810.5301863	398.5970374	772.9875241	-0.954939137	0.0001913	0.0021858	Gm8995	7	106504239	106511671	-	7433	transcribed_unproc	predicted gene 8995 [Source:MGI Symbol;Acc:MGI:3644223]	-
ENSMUSG0000003841	16.03363646	25.27840791	20.10885543	0.861331086	0.987648384	2.919076301	20.47363327	1.589351924	3.701544752	0.0001923	0.0021948	Foxq1	13	31556134	31560976	+	4843	protein_coding	forkhead box Q1 [Source:MGI Symbol;Acc:MGI:1298228]	Fork
ENSMUSG0000006388	2830.938937	2696.36351	2801.833857	2075.807916	1932.827887	1922.698257	2776.378768	1977.111353	0.48970084	0.0001926	0.0021975	Uqcrh	4	116066965	116075071	-	1237	protein_coding	ubiquinol-cytochrome c reductase hinge protein [Source:MGI Symbol;Acc:MGI:1913826]	-
ENSMUSG0000002162	50.10511393	72.67542273	11.17158635	161.068913	149.1349059	223.7958498	44.65070767	177.9998896	-1.992314307	0.0001928	0.0021989	Cd180	13	102693558	102739629	+	4509	protein_coding	CD180 antigen [Source:MGI Symbol;Acc:MGI:1194924]	-
ENSMUSG0000005150	192.4036375	156.9367824	108.3643876	260.9833189	329.8745602	377.5338683	152.5682692	322.7972491	-1.079011959	0.0001935	0.0022047	Wdfy4	14	32959547	33185508	-	10552	protein_coding	WD repeat and FYVE domain containing 4 [Source:MGI Symbol;Acc:MGI:3584510]	-
ENSMUSG0000002903	7.01471595	10.53266996	10.05442772	223.9460823	32.59239666	13.62235607	9.200604543	90.05361166	-3.294358623	0.0001938	0.0022067	Arhgef16	4	154278486	154301676	-	5481	protein_coding	Rho guanine nucleotide exchange factor (GEF) 16 [Source:MGI Symbol;Acc:MGI:2446219]	-
ENSMUSG0000002122	152.3195463	178.0021223	119.535974	7.75197977	0	0	149.9525476	2.583993257	5.836232438	0.0001939	0.0022067	Acot3	12	84052144	84060402	+	3438	protein_coding	acyl-CoA thioesterase 3 [Source:MGI Symbol;Acc:MGI:2159619]	-
ENSMUSG0000002098	504.0574461	558.2315079	349.6706528	226.5300755	271.6033055	255.9056891	470.6532023	251.3463567	0.906016598	0.0001942	0.0022086	L2hgdh	12	69690433	69724873	-	3466	protein_coding	L-2-hydroxyglutarate dehydrogenase [Source:MGI Symbol;Acc:MGI:2384968]	-
ENSMUSG0000003958	634.3307424	785.7371791	613.3200907	937.1282211	1229.622238	1266.879115	677.7960041	1144.543191	-0.755478129	0.0001998	0.0022713	Myo9a	9	59750896	59928866	+	13034	protein_coding	myosin IXa [Source:MGI Symbol;Acc:MGI:107735]	-
ENSMUSG0000002204	6188.983673	6314.335642	5099.82917	3094.762591	4054.296615	3939.779981	5867.716161	3696.279729	0.666874139	0.000202	0.002295	Ephx2	14	66084374	66124500	-	2484	protein_coding	epoxide hydrolase 2, cytoplasmic [Source:MGI Symbol;Acc:MGI:99500]	-
ENSMUSG0000005333	1693.552851	1750.529748	2667.774821	1341.0925	959.0065806	914.6439078	2037.285806	1071.580996	0.926461014	0.0002024	0.0022975	Gas5	1	161034422	161038539	+	3760	processed_transcrip	growth arrest specific 5 [Source:MGI Symbol;Acc:MGI:95659]	-
ENSMUSG0000003834	1284.695121	2021.219366	2509.138295	925.930917	1106.16619	983.7287136	1938.350927	1005.275273	0.947151526	0.0002026	0.0022984	Mlxip	5	123394798	123457932	+	8326	protein_coding	MLX interacting protein [Source:MGI Symbol;Acc:MGI:2141183]	bHL
ENSMUSG0000002528	2337.904616	2870.152564	2893.440865	1882.008422	1750.112936	1418.671082	2700.499349	1683.59748	0.681446456	0.0002027	0.0022984	Sat1	X	155213132	155216449	-	2774	protein_coding	spermidine/spermine N1-acetyl transferase 1 [Source:MGI Symbol;Acc:MGI:98233]	-
ENSMUSG0000003193	762.599834	1172.286167	1338.356045	573.646503	609.3790528	595.4915655	1091.080682	592.8390404	0.879796812	0.000203	0.0023006	Taf1d	9	15306214	15316991	+	8949	protein_coding	TATA-box binding protein associated factor, RNA polymerase I, D [Source:MGI Symbol;Acc:MGI:1922566]	-
ENSMUSG0000004494	56.1177276	75.83522372	63.67804221	125.7543385	143.2090156	144.9807896	65.21033118	137.9813813	-1.081296687	0.000204	0.0023103	Ubtd2	11	32455370	32516687	+	1012	protein_coding	ubiquitin domain containing 2 [Source:MGI Symbol;Acc:MGI:2444752]	-
ENSMUSG0000003198	11965.10121	19235.81515	9305.931431	7919.078001	6028.605734	6191.360835	13502.2826	6713.014857	1.008150356	0.0002047	0.0023175	Agt	8	124556534	124569706	-	1891	protein_coding	angiotensinogen (serpin peptidase inhibitor, clade A, member 8) [Source:MGI Symbol;Acc:MGI:87963]	-
ENSMUSG0000000313	345.7252861	824.7080579	590.976918	310.0791908	234.0726669	210.1734937	587.136754	251.4417838	1.222589102	0.0002061	0.0023318	Tbc1d8	1	39371492	39478755	-	5239	protein_coding	TBC1 domain family, member 8 [Source:MGI Symbol;Acc:MGI:1927225]	-
ENSMUSG0000006832	1345.82336	1904.306729	1734.94736	1116.285087	993.574274	878.6419667	1661.692483	996.1671092	0.737837587	0.0002075	0.0023456	Aup1	6	83054521	83057808	+	3276	protein_coding	ancient ubiquitous protein 1 [Source:MGI Symbol;Acc:MGI:107789]	-
ENSMUSG0000001771	157.3300577	195.9076613	163.1051607	99.91440593	75.06127716	58.38152603	172.1142933	77.78573637	1.142931463	0.0002077	0.0023463	Tha1	11	117867952	117873481	-	2910	protein_coding	threonine aldolase 1 [Source:MGI Symbol;Acc:MGI:1919026]	-
ENSMUSG0000006846	370.7778431	409.7208615	354.1392874	250.6473459	204.4432154	218.9307226	378.212664	224.6737613	0.750487004	0.0002079	0.0023481	Gm5518	19	29160118	29160765	+	648	processed_pseudog	predicted gene 5518 [Source:MGI Symbol;Acc:MGI:3648673]	-
ENSMUSG0000003425	821.7238685	937.4076265	911.6014463	647.7209764	446.4170695	444.6726232	890.2443158	512.9368897	0.794450855	0.0002083	0.0023503	Arhgap27	11	103331497	103363692	-	6415	protein_coding	Rho GTPase activating protein 27 [Source:MGI Symbol;Acc:MGI:1916903]	-
ENSMUSG0000002095	731.5346634	675.1441445	713.8643679	996.560066	1050.85788	1046.975367	706.8477253	1031.464438	-0.545048802	0.0002089	0.0023558	Scfd1	12	51377510	51450101	+	13162	protein_coding	Sec1 family domain containing 1 [Source:MGI Symbol;Acc:MGI:1924233]	-
ENSMUSG0000011084	1471.086145	1252.334458	1228.874499	1795.875313	2119.493432	2127.033598	1317.431701	2014.134114	-0.612084615	0.0002093	0.0023591	Gpx4-ps2	10	45875291	45876086	+	796	transcribed_process	glutathione peroxidase 4, pseudogene 2 [Source:MGI Symbol;Acc:MGI:3779731]	-
ENSMUSG0000002243	2259.740638	2349.838668	1605.356959	1298.887277	1258.264041	1217.254818	2071.645422	1258.135379	0.719602075	0.0002113	0.0023798	Samm50	15	84192241	84217267	+	6556	protein_coding	SAMM50 sorting and assembly machinery component [Source:MGI Symbol;Acc:MGI:1915903]	-
ENSMUSG0000003619	48.10090937	46.34374783	65.91235948	18.0879528	16.79002252	11.67630521	53.45233889	15.51809351	1.780161132	0.0002143	0.0024131	Slc26a8	17	28637783	28690233	-	4756	protein_coding	solute carrier family 26, member 8 [Source:MGI Symbol;Acc:MGI:2385046]	-
ENSMUSG0000005472	1289.705633	1713.665403	1233.343133	844.1044639	916.5377001	844.5860765	1412.238056	868.4094135	0.701667562	0.0002152	0.0024215	Lrrc8c	5	105519388	105613018	+	7654	protein_coding	leucine rich repeat containing 8 family, member C [Source:MGI Symbol;Acc:MGI:2140839]	-
ENSMUSG0000002893	86.18079596	135.8714425	71.49815265	208.4421227	245.9244476	200.4432194	97.85013037	218.2699365	-1.15651425	0.0002164	0.0024325	Kcnab2	4	152390742	152477871	-	5578	protein_coding	potassium voltage-gated channel, shaker-related subfamily, beta member 2 [Source:MGI Symbol;Acc:MGI:109239]	-
ENSMUSG0000003201	126.2648871	103.2201656	122.8874499	266.1513054	236.0479637	185.8478579	117.4575009	229.3490423	-0.966119373	0.0002165	0.0024325	Nectin1	9	43743984	43807461	+	5653	protein_coding	nectin cell adhesion molecule 1 [Source:MGI Symbol;Acc:MGI:1926483]	-
ENSMUSG0000010021	45.09460254	64.24928676	94.95848399	18.0879528	17.77767091	24.32563584	68.1007911	20.06375318	1.762322429	0.000217	0.0024375	Gm28151	1	46849529	46850479	-	951	lincRNA	predicted gene 28151 [Source:MGI Symbol;Acc:MGI:5578857]	-
ENSMUSG0000005137	55.11562532	28.43820889	63.67804221	107.6663857	139.2584221	145.9538151	49.07729214	130.959541	-1.416297928	0.0002176	0.0024425	Flrt3	2	140650914	140671469	-	4865	protein_coding	fibronectin leucine rich transmembrane protein 3 [Source:MGI Symbol;Acc:MGI:1918686]	-
ENSMUSG0000002542	143.3006258	214.8664672	270.3523897	96.46908159	65.18479333	102.1676705	209.5064943	87.94051515	1.25045685	0.0002202	0.0024698	Katnal2	18	76977148	77047308	-	4061	protein_coding	katanin p60 subunit A-like 2 [Source:MGI Symbol;Acc:MGI:1924234]	-
ENSMUSG0000002022	741.5556862	620.3742607	859.0949905	477.1774214	360.4916601	448.564725	740.3416458	428.7446022	0.787361891	0.0002204	0.0024705	Llph	10	120227070	120232582	+	2181	protein_coding	LLP homolog, long-term synaptic facilitation (Aplysia) [Source:MGI Symbol;Acc:MGI:1913475]	-
ENSMUSG0000003129	1244.61103	1099.610744	920.5387154	1513.358717	1863.6925	2071.571149	1088.253496	1816.207455	-0.738476653	0.0002211	0.0024775	Phka2	X	160502166	160598878	+	6068	protein_coding	phosphorylase kinase alpha 2 [Source:MGI Symbol;Acc:MGI:97577]	-
ENSMUSG0000004085	7.01471595	4.213067984	12.28874499	51.67986514	32.59239666	35.02891562	7.838842974	39.76705914	-2.351994477	0.0002213	0.0024776	Dlk1	12	109452823	109463336	+	5657	protein_coding	delta like non-canonical Notch ligand 1 [Source:MGI Symbol;Acc:MGI:94900]	-
ENSMUSG0000000651	115.241762	117.9659036	129.5904017	258.3993257	197.5296767	218.9307226	120.9326891	224.9532417	-0.897019172	0.0002215	0.002479	Mvd	8	122433601	122443422	-	1859	protein_coding	mevalonate (diphospho) decarboxylase [Source:MGI Symbol;Acc:MGI:2179327]	-
ENSMUSG0000005285	0	1.053266996	0	32.73058125	11.8517806	10.70327977	0.351088999	18.42854721	-5.608982854	0.0002235	0.0024996	Nrk	X	138914430	139010532	+	9210	protein_coding	Nik related kinase [Source:MGI Symbol;Acc:MGI:1351326]	-
ENSMUSG0000002401	136.2859099	194.8543943	81.55258037	330.7511369	317.0351312	298.7188082	137.5642948	315.5016921	-1.196517131	0.0002238	0.002501	Fgd2	17	29360914	29379660	+	4134	protein_coding	FYVE, RhoGEF and PH domain containing 2 [Source:MGI Symbol;Acc:MGI:1347084]	-
ENSMUSG0000003785	24.05045469	16.85227194	14.52306226	62.01583816	50.37006757	66.1657295	18.47526296	59.51721174	-1.683719846	0.0002247	0.0025087	Zfp365	10	67886103	67912662	-	5205	protein_coding	zinc finger protein 365 [Source:MGI Symbol;Acc:MGI:2143676]	-
ENSMUSG0000002035	525.101594	349.6846427	340.7333837	559.0038745	956.0436355	1098.545715	405.1732068	871.1977416	-1.103641937	0.0002247	0.0025087	Sgcd	11	46896253	47988969	-	8257	protein_coding	sarcoglycan, delta (dystrophin-associated glycoprotein) [Source:MGI Symbol;Acc:MGI:1346525]	-
ENSMUSG0000008105	981.0581308	1508.278338	1136.150332	745.9127201	699.2550557	684.0368799	1208.4956	709.7348853	0.76765741	0.0002252	0.0025122	Gm15427	1	95498621	95499151	+	531	processed_pseudog	predicted pseudogene 15427 [Source:MGI Symbol;Acc:MGI:3642341]	-
ENSMUSG0000003630	4367.16173	4570.125496	4487.626238	3167.114402	3379.732769	2864.586877	4474.971155	3137.144683	0.512415909	0.0002267	0.0025281	Skp1a	11	52231995	52246858	+	2211	protein_coding	S-phase kinase-associated protein 1A [Source:MGI Symbol;Acc:MGI:103575]	-
ENSMUSG0000009202	36.07568203	130.6051075	81.55258037	173.9888793	223.2085347	505.0002001	82.74445664	300.732538	-1.862222972	0.0002314	0.0025783	Gbp11	5	105323042	105346472	-	2692	polymorphic_pseud	guanylate binding protein 11 [Source:MGI Symbol;Acc:MGI:3646307]	-
ENSMUSG0000002782	2571.394447	2794.317341	4354.68436	1565.899914	2046.407451	1634.682729	3240.132049	1748.996698	0.889502053	0.0002316	0.0025793	Ccnl1	3	65946151	65958249	-	5997	protein_coding	cyclin L1 [Source:MGI Symbol;Acc:MGI:1922664]	-
ENSMUSG0000004079	25.05255697	17.90553893	45.80350404	68.90648685	100.7401351	120.6551538	29.58719998	96.76725859	-1.712511326	0.0002328	0.0025918	Iqsec3	6	121372933	121473678	-	13577	protein_coding	IQ motif and Sec7 domain 3 [Source:MGI Symbol;Acc:MGI:2677208]	-
ENSMUSG0000002889	1400.938986	838.4005289	887.0239564	579.6758206	601.4778657	488.4587678	1042.121157	556.5374847	0.905192721	0.0002346	0.0026093	Sesn2	4	132492032	132510501	-	3475	protein_coding	sestrin 2 [Source:MGI Symbol;Acc:MGI:2651874]	-
ENSMUSG0000002682	300.6306836	274.902686	235.720472	417.7455765	589.6260851	444.6726232	270.4179472	484.0147616	-0.838713554	0.0002349	0.0026117	Dnm1	2	32308471	32353338	-	5249	protein_coding	dynamin 1 [Source:MGI Symbol;Acc:MGI:107384]	-
ENSMUSG0000005649	11018.11455	13373.33105	9729.334554	5879.44599	7648.349084	7466.997179	11373.59339	6998.264084	0.700681638	0.0002357	0.0026184	Adgrf5	17	43360451	43459557	+	9010	protein_coding	adhesion G protein-coupled receptor F5 [Source:MGI Symbol;Acc:MGI:2182928]	-
ENSMUSG0000003531	1077.25995	1162.806764	933.944619	1289.412635	2716.033055	2445.212915	1058.003777	2150.219535	-1.022875537	0.0002368	0.0026295	Gnptab	10	88379132	88447329	+	5883	protein_coding	N-acetylglucosamine-1-phosphate transferase, alpha and beta subunits [Source:MGI Symbol;Acc:MGI:3643902]	-
ENSMUSG0000001424	391.8219909	331.7791038	340.7333837	204.9967984	226.1714799	210.1734937	354.7781595	213.7805906	0.731589744	0.0002379	0.0026407	Pigl	11	62458460	62514417	+	2268	protein_coding	phosphatidylinositol glycan anchor biosynthesis, class L [Source:MGI Symbol;Acc:MGI:2681271]	-
ENSMUSG0000003725	5.010511393	7.372868973	15.64022089	112.8343722	29.62945151	35.02891562	9.34120042	59.16424645	-2.67372125	0.0002385	0.0026452	Itih2	2	10094593	10131396	-	3315	protein_coding	inter-alpha trypsin inhibitor, heavy chain 2 [Source:MGI Symbol;Acc:MGI:96619]	-
ENSMUSG0000002089	10.02102279	8.426135969	11.17158635	58.57051382	30.6170999	38.92101735	9.872915036	42.70287702	-2.116689502	0.0002401	0.0026618	Aurkb	11	69045647	69051664	+	2946	protein_coding	aurora kinase B [Source:MGI Symbol;Acc:MGI:107168]	-
ENSMUSG0000002175	1040.182165	555.0717069	1146.20476	508.1853405	395.0593535	412.5627839	913.819544	438.6024926	1.05856521	0.0002404	0.0026634	Plpp1	13	112800792	112867894	+	1750	protein_coding	phospholipid phosphatase 1 [Source:MGI Symbol;Acc:MGI:108412]	-
ENSMUSG0000002840	3635.627067	2401.448751	1558.436296	4271.340853	6124.407628	5807.988814	2531.837371	5401.245765	-1.09293333	0.0002408	0.0026643	Aco1	4	40143081	40198338	+	4496	protein_coding	aconitase 1 [Source:MGI Symbol;Acc:MGI:87879]	-
ENSMUSG0000005143	1006.110688	1209.150512	2137.124469	571.0625097	724.9339137	764.797991	1450.795223	686.9314715	1.078579612	0.0002408	0.0026643	Cd14	18	36725074	36726736	-	1572	protein_coding	CD14 antigen [Source:MGI Symbol;Acc:MGI:88318]	-
ENSMUSG0000002798	996.0896649	885.7975437	1402.034087	477.1774214	649.8726365	651.9270406	1094.640432	592.9923662	0.88458049	0.0002412	0.0026668	Rnpc3	3	113605067	113630149	-	6627	protein_coding	RNA-binding region (RNP1, RRM) containing 3 [Source:MGI Symbol;Acc:MGI:1914475]	-
ENSMUSG0000002605	16.03363646	11.58593696	14.52306226	161.068913	28.64180313	47.67824626	14.04754522	79.12965413	-2.495930652	0.0002423	0.002678	1500015O10Ri	1	43730602	43742578	+	902	protein_coding	RIKEN cDNA 1500015O10 gene [Source:MGI Symbol;Acc:MGI:1926146]	-
ENSMUSG0000003451	540.1331282	465.5440123	502.7213858	291.1299069	343.7016375	307.4760371	502.7995088	314.1025272	0.679481804	0.0002431	0.0026852	Hmgxb4	8	74993356	75031978	+	5227	protein_coding	HMG box domain containing 4 [Source:MGI Symbol;Acc:MGI:1918073]	HMG
ENSMUSG0000002648	1102.312506	1090.131341	874.7352114	1689.070259	1455.793718	1598.680788	1022.39302	1581.181588	-0.62889067	0.0002465	0.0027211	Fam129a	1	151571188	151721939	+	7380	protein_coding	family with sequence similarity 129, member A [Source:MGI Symbol;Acc:MGI:2137237]	-
ENSMUSG0000002572	52.10931849	68.46235475	63.67804221	167.9595617	99.75248676	172.2255018	61.41657181	146.6458501	-1.257382665	0.0002482	0.002738	Nmb	7	80902227	80905076	-	793	protein_coding	neuromedin B [Source:MGI Symbol;Acc:MGI:1915289]	-
ENSMUSG0000003553	7870.511296	7330.738293	10912.40555	5346.282048	5425.152572	5296.177436	8704.551713	5355.870685	0.700605634	0.0002483	0.002738	Eif1	11	100319885	100322096	+	2098	protein_coding	eukaryotic translation initiation factor 1 [Source:MGI Symbol;Acc:MGI:105125]	-
ENSMUSG0000003666	448.9418208	542.432503	461.3865163	298.0205556	308.1462957	215.0386209	484.2536134	273.7351574	0.822575072	0.0002492	0.0027445	Dennd3	15	73512560	73572242	+	5709	protein_coding	DENN/MADD domain containing 3 [Source:MGI Symbol;Acc:MGI:2146009]	-
ENSMUSG0000002564	166.3489783	176.9488553	125.1217671	301.46588	270.6156571	280.2313249	156.1398669	284.1042873	-0.862738417	0.0002492	0.0027445	Pfkfb4	9	108991778	109032228	+	4875	protein_coding	6-phosphofructo-2-kinase/fructose-2,6-biphosphatase 4 [Source:MGI Symbol;Acc:MGI:2687284]	-
ENSMUSG0000011687	361.7589226	418.1469975	488.1983236	260.1219878	226.1714799	254.9326637	422.7014145	247.0753771	0.773639048	0.0002497	0.0027483	AC115954.1	16	24529394	24530362	+	969	processed_pseudog	mortality factor 4 like 1 (Morf4l1) pseudogene	-
ENSMUSG0000010913	18.03784102	12.63920395	43.56918677	62.01583816	95.80189322	138.1696116	24.74874391	98.66244766	-1.999372561	0.0002518	0.0027701	Gm45187	7	98904268	98908055	+	3132	antisense	predicted gene 45187 [Source:MGI Symbol;Acc:MGI:5753763]	-
ENSMUSG0000009731	1.002102279	2.106533992	2.23431727	19.81061497	13.82707737	31.13681388	1.780984514	21.5150207	-3.610348925	0.0002533	0.0027831	Gm26569	16	29907519	29946754	-	4295	processed_transcrip	predicted gene, 26569 [Source:MGI Symbol;Acc:MGI:5477063]	-
ENSMUSG0000006293	417.8766502	432.8927354	458.0350404	620.1583816	676.5391429	690.848058	436.268142	662.5151942	-0.602747174	0.0002534	0.0027831	Mtap	4	89137122	89181081	+	3226	protein_coding	methylthioadenosine phosphorylase [Source:MGI Symbol;Acc:MGI:1914152]	-
ENSMUSG0000002224	579.215117	624.5873287	902.6641773	424.6362252	395.0593535	326.9365457	702.155541	382.2107081	0.876607569	0.0002535	0.0027831	Rai14	15	10568969	10714624	-	8462	protein_coding	retinoic acid induced 14 [Source:MGI Symbol;Acc:MGI:1922896]	-
ENSMUSG0000005549	101.1325	57.5687	83.1024	23.6792	39.2584	58.5831	80.6012	40.5069	1.029115909	0.0002537	0.0027838	Ptgs2	1	149975782	149983985	+	4460	protein_coding	Cox-2, COX2, cyclooxygenase 2, cyclooxygenase-2, Pghs2, PGHS-2, PHS-2, prostaglandin G/H synthase, Tis10 [Source:MGI Syn	-
ENSMUSG0000007094	116.2438643	81.1015587	90.48984945	171.404886	198.5173251	201.4162648	95.94509082	190.4461587	-0.987186788	0.0002541	0.0027852	Il1rl2	1	40324610	40367562	+	6812	protein_coding	interleukin 1 receptor-like 2 [Source:MGI Symbol;Acc:MGI:1913107]	-
ENSMUSG0000002291	2242.7049	2684.777573	2947.06448	1676.150293	1784.680629	1732.958298	2624.848984	1731.263073	0.600321592	0.0002541	0.0027852	Pros1	16	62854307	62929346	+	4088	protein_coding	protein S (alpha) [Source:MGI Symbol;Acc:MGI:1095733]	-
ENSMUSG0000003489	3767.904568	2391.969348	3121.341227	2106.815835	1262.214636	1548.083465	3093.738381	1639.037978	0.916309941	0.0002549	0.0027926	Rps29	12	69157722	69159186	-	693	protein_coding	ribosomal protein S29 [Source:MGI Symbol;Acc:MGI:107681]	-
ENSMUSG0000002890	224.4709104	285.4353559	513.8929722	164.5142373	121.4807512	143.0347388	341.2664129	143.0099091	1.253194354	0.0002552	0.0027942	Ptpru	4	131768457	131838288	-	7113	protein_coding	protein tyrosine phosphatase, receptor type, U [Source:MGI Symbol;Acc:MGI:1321151]	-
ENSMUSG0000003182	62.13034127	23.17187391	22.3431727	159.3462508	90.8636513	117.7360775	35.88179596	122.6486599	-1.76955419	0.0002564	0.0028053	Gins2	8	120578633	120589304	-	4097	protein_coding	GINS complex subunit 2 (Psf2 homolog) [Source:MGI Symbol;Acc:MGI:1921019]	-
ENSMUSG0000002218	745.5640953	2970.212929	2303.581106	718.3501254	639.9961527	835.8288476	2006.45271	731.3917086	1.455843363	0.0002584	0.002826	Slc7a8	14	54722209	54781946	-	5005	protein_coding	solute carrier family 7 (cationic amino acid transporter, y+ system), member 8 [Source:MGI Symbol;Acc:MGI:1355323]	-
ENSMUSG0000003109	1276.678303	1544.089416	1280.263796	714.04347	908.636513	931.1853401	1367.010505	851.288441	0.683736554	0.0002594	0.0028335	Cul4b	X	38533274	38576196	-	3873	protein_coding	cullin 4B [Source:MGI Symbol;Acc:MGI:1919834]	-
ENSMUSG0000002917	62.13034127	46.34374783	54.74077313	129.1996628	105.6783771	123.5742301	54.40495408	119.48409	-1.134871968	0.0002594	0.0028335	Cenpa	5	30666777	30674830	+	5860	protein_coding	centromere protein A [Source:MGI Symbol;Acc:MGI:88375]	-
ENSMUSG0000003166	697.4631859	511.8877601	693.7555125	1196.388878	960.9818774	1013.892502	634.3688195	1057.087752	-0.736975671	0.0002608	0.0028472	Aktip	8	91111784	91199976	-	7040	protein_coding	thymoma viral proto-oncogene 1 interacting protein [Source:MGI Symbol;Acc:MGI:3693832]	-
ENSMUSG0000003894	2453.146378	3664.315879	1233.343133	921.6242616	1072.586145	1144.27791	2450.268464	1046.162772	1.227985308	0.0002613	0.0028513	Cnst	1	179546370	179627478	+	8859	protein_coding	consortin, connexin sorting protein [Source:MGI Symbol;Acc:MGI:2445141]	-
ENSMUSG0000004471	18.03784102	20.01207293	34.63191769	152.4556021	53.33301272	72.0038821	24.22727721	92.59749899	-1.940171083	0.0002617	0.0028532	Dok7	5	35056766	35087839	+	4275	protein_coding	docking protein 7 [Source:MGI Symbol;Acc:MGI:3584043]	-
ENSMUSG0000004014	233.4898309	280.169021	198.8542371	128.3383318	132.3448834	121.6281792	237.504363	127.4371315	0.898601892	0.00026	0.0028556	Rgl3	9	21968711	21989446	-	3924	protein_coding	ral guanine nucleotide dissociation stimulator-like 3 [Source:MGI Symbol;Acc:MGI:1918996]	-
ENSMUSG0000003060	155.3258532	144.2975785	122.8874499	372.095029	232.0973702	249.094511	140.8369605	284.4289701	-1.014424393	0.0002639	0.0028742	Pak4	7	28558819	28598185	-	3535	protein_coding	p21 (RAC1) activated kinase 4 [Source:MGI Symbol;Acc:MGI:1917834]	-

ENSMUSG0000005473	494.0364234	507.6746921	461.3865163	820.8485246	676.5391429	758.9598383	487.6992106	752.1158353	-0.625137709	0.0002642	0.0028754	Msra	14	64122625	64455903	-		4295	protein_coding	methionine sulfoxide reductase A [Source:MGI Symbol;Acc:MGI:106916]	-
ENSMUSG0000005992	57.11982988	38.97087886	75.96678719	3.445324342	20.74061606	5.838152603	57.35249864	10.008031	2.529439879	0.0002645	0.0028767	Unc5c	3	141465216	141834924	+		19135	protein_coding	unc-5 netrin receptor C [Source:MGI Symbol;Acc:MGI:1095412]	-
ENSMUSG0000006012	217.4561945	220.1328022	225.6660443	141.258298	100.7401351	111.8979249	221.0850137	117.9654527	0.903966429	0.0002647	0.0028767	Gemin2	12	59013393	59028470	+		2415	protein_coding	gem nuclear organelle associated protein 2 [Source:MGI Symbol;Acc:MGI:1913853]	-
ENSMUSG0000002817	132.2775008	131.6583745	106.1300703	213.6101092	244.9367992	217.9576972	123.3553152	225.5015352	-0.868785952	0.0002647	0.0028767	Cth	3	157894248	157925077	-		2791	protein_coding	cystathionase (cystathionine gamma-lyase) [Source:MGI Symbol;Acc:MGI:1339968]	-
ENSMUSG0000002700	224.4709104	226.4524042	178.7453816	294.5752313	451.3553114	474.8364117	209.8895654	406.9223181	-0.953733102	0.0002654	0.0028822	Itga4	2	79255426	79333123	+		13978	protein_coding	integrin alpha 4 [Source:MGI Symbol;Acc:MGI:96603]	-
ENSMUSG0000000999	1052.207393	1138.581623	1214.351436	811.3738826	737.7733427	617.8711504	1135.046817	722.3394586	0.651463225	0.0002659	0.002886	Taz	X	74281912	74290151	+		5364	protein_coding	tafazzin [Source:MGI Symbol;Acc:MGI:109626]	-
ENSMUSG0000003611	2532.312458	2424.620625	2430.93719	1805.349955	1787.643575	1747.553679	2462.623424	1780.182403	0.468173897	0.0002668	0.0028943	Metap2	10	93858489	93897093	-		7654	protein_coding	methionine aminopeptidase 2 [Source:MGI Symbol;Acc:MGI:1929701]	-
ENSMUSG0000002257	533.1184122	537.166168	625.6088357	347.9777586	388.1458148	311.3681388	565.2978053	349.1639041	0.694889344	0.0002672	0.0028971	Gsdmd	15	75862327	75867408	+		2377	protein_coding	gasdermin D [Source:MGI Symbol;Acc:MGI:1916396]	-
ENSMUSG0000001890	203.4267626	224.3458702	264.7665965	391.9056439	413.8246728	361.9654614	230.8464098	389.231926	-0.754696933	0.0002693	0.0029175	P4ha2	11	54100095	54131665	+		6239	protein_coding	procollagen-proline, 2-oxoglutarate 4-dioxygenase (proline 4-hydroxylase), alpha II polypeptide [Source:MGI Symbol;Acc:MGI:89	-
ENSMUSG0000000138	1247.617337	1117.516283	1465.712129	886.3096871	701.2303525	797.8808557	1276.948583	795.1402984	0.682915454	0.0002714	0.0029384	Zmat2	18	36793923	36799660	+		2257	protein_coding	zinc finger, matrin type 2 [Source:MGI Symbol;Acc:MGI:1913742]	-
ENSMUSG0000003014	55.11562532	220.1328022	232.3689961	24.97860148	36.5429902	63.2466532	169.2058079	41.58941496	2.025873817	0.0002726	0.0029497	Clec4d	6	123262111	123275265	+		1599	protein_coding	C-type lectin domain family 4, member d [Source:MGI Symbol;Acc:MGI:1298389]	-
ENSMUSG0000002779	99.20812558	127.4453065	100.5442772	37.89856777	58.27125464	39.89404278	109.0659031	45.35462173	1.268482113	0.0002728	0.0029502	Smad9	3	54755582	54801257	+		5368	protein_coding	SMAD family member 9 [Source:MGI Symbol;Acc:MGI:1859993]	MH1
ENSMUSG0000008306	307.6453995	389.7087886	256.9464861	181.7408591	157.036093	175.1445781	318.1002247	171.3071767	0.89276422	0.0002745	0.0029665	Gm12191	11	49678710	49679057	-		348	processed_pseudog	predicted gene 12191 [Source:MGI Symbol;Acc:MGI:3651293]	-
ENSMUSG0000000128	117.2459666	63.19601977	90.48984945	221.362089	188.6408413	180.0097052	90.31061194	196.6708785	-1.122238829	0.0002746	0.0029665	Itgb7	15	102215995	102231944	-		2770	protein_coding	integrin beta 7 [Source:MGI Symbol;Acc:MGI:96616]	-
ENSMUSG0000003762	7.01471595	13.69247095	12.28874499	251.508677	15.80237414	33.08286475	10.99864396	100.1313053	-3.190112055	0.0002749	0.0029677	Cldn11	3	31149920	31164324	+		1870	protein_coding	claudin 11 [Source:MGI Symbol;Acc:MGI:106925]	-
ENSMUSG0000003336	529.1100031	487.6626192	552.9935244	726.1021052	838.5134778	828.0446441	523.2553822	797.553409	-0.607871924	0.0002767	0.0029858	Ipo13	4	117894486	117914999	-		4207	protein_coding	importin 13 [Source:MGI Symbol;Acc:MGI:2385205]	-
ENSMUSG0000000486	1120.350347	1290.25207	1313.778555	789.8406055	889.8711938	813.4492626	1241.460324	831.0536873	0.579045174	0.0002769	0.0029858	Srpk1	17	28587648	28622709	-		12376	protein_coding	serine/arginine-rich protein specific kinase 1 [Source:MGI Symbol;Acc:MGI:106908]	-
ENSMUSG0000002901	587.2319353	626.6938627	745.1448097	379.8470087	396.0470019	442.7265724	653.0235359	406.206861	0.684822856	0.0002788	0.0030044	Orc5	5	22486485	22550429	-		6166	protein_coding	origin recognition complex, subunit 5 [Source:MGI Symbol;Acc:MGI:1347044]	-
ENSMUSG0000004080	311.6538087	607.7350567	1359.582059	45.65054754	160.9866866	30.16378845	759.6569748	78.93367418	3.267186809	0.0002789	0.0030044	Chil3	3	106147554	106167564	-		1632	protein_coding	chitinase-like 3 [Source:MGI Symbol;Acc:MGI:1330860]	-
ENSMUSG0000003688	1786.748363	1303.944541	1395.331135	2415.172364	2189.616467	2460.781322	1495.341346	2355.190051	-0.655208468	0.0002797	0.0030103	Acaa2	18	74779197	74806207	+		1515	protein_coding	acetyl-Coenzyme A acyltransferase 2 (mitochondrial 3-oxoacyl-Coenzyme A thiolase) [Source:MGI Symbol;Acc:MGI:1098623]	-
ENSMUSG0000008328	1601.359441	1202.83091	1222.171547	906.120302	772.3410361	829.990695	1342.120633	836.1506777	0.682665149	0.0002799	0.0030103	Ctsf	19	4855129	4860912	+		1975	protein_coding	cathepsin F [Source:MGI Symbol;Acc:MGI:1861434]	-
ENSMUSG0000003279	259.5444902	361.2705797	107.247229	101.6370681	56.29595787	79.78808557	242.6874329	79.24037051	1.613860328	0.0002799	0.0030103	Lama1	17	67697259	67822647	+		9681	protein_coding	laminin, alpha 1 [Source:MGI Symbol;Acc:MGI:99892]	-
ENSMUSG0000002818	1949.088932	2066.509846	2220.911367	1377.268406	1478.50963	1497.486143	2078.836715	1451.08806	0.5186557	0.0002812	0.0030218	Zranb2	3	157534160	157548410	+		6759	protein_coding	zinc finger, RAN-binding domain containing 2 [Source:MGI Symbol;Acc:MGI:1858211]	-
ENSMUSG0000004053	305.641195	376.0163176	347.4363356	541.7772528	545.1819078	530.2988614	343.0312827	539.0860074	-0.652567799	0.000282	0.0030292	Abhd11	5	135009152	135012175	+		2172	protein_coding	abhydrolase domain containing 11 [Source:MGI Symbol;Acc:MGI:1916008]	-
ENSMUSG0000002783	19.03994329	9.479402965	42.45202814	103.3597303	146.1719608	57.40850059	23.6571248	102.3133972	-2.117233049	0.0002833	0.0030415	Ptx3	3	66219910	66225805	+		2682	protein_coding	pentraxin related gene [Source:MGI Symbol;Acc:MGI:104641]	-
ENSMUSG0000006393	697.4631859	794.1633151	389.8883637	1045.655938	1548.632666	1275.636344	627.1716216	1289.974982	-1.039874238	0.0002847	0.0030543	Pepd	7	34912379	35044708	+		3082	protein_coding	peptidase D [Source:MGI Symbol;Acc:MGI:97542]	-
ENSMUSG0000004615	32.06727292	21.06533992	23.46033134	76.65846662	61.23419979	75.89598383	25.53098139	71.26288341	-1.478990827	0.0002879	0.0030852	Chrm3	13	9875486	10360847	-		6253	protein_coding	cholinergic receptor, muscarinic 3, cardiac [Source:MGI Symbol;Acc:MGI:88398]	-
ENSMUSG0000006276	582.2214239	708.8486884	856.8606732	432.388205	449.3800146	376.5608429	715.9769285	419.4430208	0.770892002	0.0002879	0.0030852	Zfp512	5	31452431	31481754	+		6886	protein_coding	zinc finger protein 512 [Source:MGI Symbol;Acc:MGI:1917345]	zf-C2
ENSMUSG0000008553	0	0	1.117158635	9.474641942	17.77767091	22.37958498	0.372386212	16.54396594	-5.44769274	0.0002881	0.0030852	B430319H21R	11	63173914	63188454	+		734	lincRNA	RIKEN cDNA B430319H21 gene [Source:MGI Symbol;Acc:MGI:1925099]	-
ENSMUSG0000002114	1285.697223	1315.530478	2130.421517	776.9206392	865.1799842	944.8076962	1577.216406	862.3027732	0.871027593	0.0002908	0.0031123	Gtpbp4	13	8966331	8996083	-		9566	protein_coding	GTP binding protein 4 [Source:MGI Symbol;Acc:MGI:1916487]	-
ENSMUSG0000003245	386.8114795	456.0646093	518.3616067	169.6822239	300.2451087	224.7688752	453.7458985	231.5654026	0.971470983	0.0002913	0.0031168	Nmnat3	9	98287435	98420438	+		5004	protein_coding	nicotinamide nucleotide adenylyltransferase 3 [Source:MGI Symbol;Acc:MGI:1921330]	-
ENSMUSG0000003420	482.011196	222.2393362	602.1485044	897.5069912	1115.055025	971.0793829	435.4663455	994.5471331	-1.191665167	0.0002925	0.0031269	Loxl2	14	69609068	69695834	+		5405	protein_coding	lysyl oxidase-like 2 [Source:MGI Symbol;Acc:MGI:2137913]	-
ENSMUSG0000005902	36.07568203	13.69247095	11.17158635	105.9437235	70.12303525	63.2466532	20.31324644	79.77113732	-1.967721562	0.0002932	0.0031328	Kcp	6	29473162	29507952	-		7807	protein_coding	kielin/chordin-like protein [Source:MGI Symbol;Acc:MGI:2141640]	-
ENSMUSG0000002275	42.0882957	27.3849419	26.81180725	161.068913	79.99951908	69.0848058	32.09501495	103.3844126	-1.687356563	0.0002933	0.0031328	Tmem45a	16	56805161	56886166	-		3129	protein_coding	transmembrane protein 45a [Source:MGI Symbol;Acc:MGI:1913122]	-
ENSMUSG0000004955	78.16397773	84.26135969	77.08394583	38.75989885	15.80237414	24.32563584	79.83642775	26.29596961	1.595135832	0.0002949	0.003148	Tmie	9	110865711	110880113	-		3094	protein_coding	transmembrane inner ear [Source:MGI Symbol;Acc:MGI:2159400]	-
ENSMUSG0000005358	449.9439231	378.1228516	415.5830123	619.2970505	619.2555366	781.3394233	414.549929	673.2973368	-0.69930785	0.0002979	0.003178	Zfand2a	5	139471211	139484549	-		3407	protein_coding	zinc finger, AN1-type domain 2A [Source:MGI Symbol;Acc:MGI:2140729]	-
ENSMUSG0000007493	0	0	3.351475906	113.6957033	2.962945151	13.62235607	1.117158635	43.42700151	-5.321648962	0.0002989	0.0031871	Grem1	2	113746164	113758646	-		4148	protein_coding	gremlin 1, DAN family BMP antagonist [Source:MGI Symbol;Acc:MGI:1344337]	-
ENSMUSG0000005626	858.8016528	723.5944263	632.3117875	342.8097721	473.0835758	462.187081	738.2359555	426.0268096	0.794224263	0.0002992	0.0031886	Cep70	9	99243367	99300404	+		4806	protein_coding	centrosomal protein 70 [Source:MGI Symbol;Acc:MGI:1915371]	-
ENSMUSG0000006212	981.0581308	1554.622086	1074.706607	704.568828	704.1932976	680.1447782	1203.462275	696.3023013	0.789350758	0.0002999	0.0031939	Cttnbp2nl	3	105001915	105053146	-		8138	protein_coding	CTTNBP2 N-terminal like [Source:MGI Symbol;Acc:MGI:1933137]	-
ENSMUSG0000002252	3.006306836	1.053266996	1.117158635	161.068913	0.987648384	7.78420347	1.725577489	56.61358829	-5.027760566	0.0003004	0.0031971	Hrasls	16	29209695	29230531	+		3286	protein_coding	HRAS-like suppressor [Source:MGI Symbol;Acc:MGI:1351473]	-
ENSMUSG0000002117	2608.472231	2614.208684	2973.876287	2011.208085	1871.593687	1836.098994	2732.185734	1906.300255	0.519076834	0.0003013	0.0032055	Psmc1	12	100110154	100123405	+		1933	protein_coding	protease (prosome, macropain) 26S subunit, ATPase 1 [Source:MGI Symbol;Acc:MGI:106054]	-
ENSMUSG0000006689	332.6979565	361.2705797	302.7499901	219.6394268	187.6531929	175.1445781	332.2395088	194.1457326	0.774226994	0.0003024	0.0032152	Vsig10	5	117319083	117355005	+		5305	protein_coding	V-set and immunoglobulin domain containing 10 [Source:MGI Symbol;Acc:MGI:2448533]	-
ENSMUSG0000003503	1.002102279	0	0	60.29317599	5.925890302	5.838152603	0.334034093	24.01907297	-5.991324083	0.0003036	0.0032257	Nek11	9	105162156	105395524	-		4246	protein_coding	NIMA (never in mitosis gene a)-related expressed kinase 11 [Source:MGI Symbol;Acc:MGI:2442276]	-
ENSMUSG0000003243	2274.772172	2133.918934	1807.562672	2941.445657	3295.782657	3016.378845	2072.084593	3084.53572	-0.573750298	0.000304	0.003228	Cmtm6	9	114731116	114749344	+		3440	protein_coding	CKLF-like MARVEL transmembrane domain containing 6 [Source:MGI Symbol;Acc:MGI:2447165]	-
ENSMUSG0000006153	153.3216486	143.2443115	166.4566366	275.6259474	252.8379862	260.7708163	154.3408656	263.07825	-0.77019965	0.0003053	0.0032403	C1qtnf7	5	43515538	43618803	+		4162	protein_coding	C1q and tumor necrosis factor related protein 7 [Source:MGI Symbol;Acc:MGI:1925911]	-
ENSMUSG0000009720	71.14926178	66.35582075	88.25553218	23.25593931	35.55534181	16.54143237	75.25353824	25.11757117	1.584194014	0.0003072	0.0032586	Gm17690	3	96167464	96172416	-		3469	antisense	predicted gene, 17690 [Source:MGI Symbol;Acc:MGI:4937324]	-
ENSMUSG0000004077	1544.239611	2191.848619	1673.503636	1094.75181	1167.40039	1123.844376	1803.197289	1128.665525	0.675974432	0.0003094	0.0032804	Cept1	3	106502260	106547802	-		3116	protein_coding	choline/ethanolaminephosphotransferase 1 [Source:MGI Symbol;Acc:MGI:2139793]	-
ENSMUSG0000000366	4027.449058	3882.342148	3230.822773	2229.986181	2581.712875	2597.004883	3713.537993	2469.567979	0.588737293	0.00031	0.0032843	Snrnp200	2	127208386	127240451	+		7819	protein_coding	small nuclear ribonucleoprotein 200 (U5) [Source:MGI Symbol;Acc:MGI:2444401]	-
ENSMUSG0000007661	0	0	0	24.1172704	6.913538686	7.78420347	0	12.93833752	-6.060583715	0.000312	0.0033033	Ighg1	12	113325240	113330523	-		2592	IG_C_gene	immunoglobulin heavy constant gamma 1 (G1m marker) [Source:MGI Symbol;Acc:MGI:96446]	-
ENSMUSG0000003420	882.8521075	739.3934313	659.1235948	1129.205053	1176.289225	1265.906089	760.4563778	1190.466789	-0.646040365	0.0003145	0.003328	Gas2l1	11	5054132	5065327	-		6100	protein_coding	growth arrest-specific 2 like 1 [Source:MGI Symbol;Acc:MGI:1926176]	-
ENSMUSG0000001626	56.1177276	7.372868973	16.75737953	143.8422913	132.3448834	96.32951794	26.74932537	124.1722309	-2.210027413	0.0003185	0.0033692	Sertad4	1	192844488	192855756	-		3850	protein_coding	SERTA domain containing 4 [Source:MGI Symbol;Acc:MGI:2443496]	-
ENSMUSG0000001740	19.03994329	20.01207293	32.39760042	102.4983992	55.30830949	68.11178036	23.81653888	75.30616301	-1.66678196	0.0003189	0.0033713	Stac2	11	98036623	98053462	-		3139	protein_coding	SH3 and cysteine rich domain 2 [Source:MGI Symbol;Acc:MGI:2144518]	-
ENSMUSG0000005617	29.06096608	27.3849419	14.52306226	335.0577923	43.45652888	53.51639886	23.65632341	144.01024	-2.605491523	0.0003193	0.003374	Col8a2	4	126286793	126314330	+		4489	protein_coding	collagen, type VIII, alpha 2 [Source:MGI Symbol;Acc:MGI:88464]	-
ENSMUSG0000002407	181.3805124	137.9779765	91.60700809	488.3747255	244.9367992	277.3122486	136.988499	336.8745911	-1.297630304	0.0003198	0.0033772	Vit	17	78508064	78627408	+		3227	protein_coding	vitrin [Source:MGI Symbol;Acc:MGI:1921449]	-
ENSMUSG0000004333	2149.509388	1580.953761	1948.32466	1089.583823	1319.498241	1178.3338	1892.92927	1195.805288	0.662878066	0.000321	0.0033881	Filip1l	16	57353093	57573126	+		5335	protein_coding	filamin A interacting protein 1-like [Source:MGI Symbol;Acc:MGI:1925999]	-
ENSMUSG0000002848	224.4709104	261.210215	182.0968575	334.1964612	419.7505631	480.6745643	222.592661	411.5405295	-0.88546395	0.0003215	0.0033909	Bnc2	4	84275095	84675275	-		8174	protein_coding	basonuclin 2 [Source:MGI Symbol;Acc:MGI:2443805]	zf-C2
ENSMUSG0000003187	10997.07041	13060.51075	7993.270035	6453.953824	6382.183856	6443.374422	10683.61706	6426.504034	0.733318829	0.0003218	0.0033926	Cdh5	8	104101625	104144511	+		4203	protein_coding	cadherin 5 [Source:MGI Symbol;Acc:MGI:105057]	-
ENSMUSG0000002448	281.5907403	198.0141953	293.8127211	80.10379096	153.0854995	116.7630521	257.8058855	116.6507808	1.146162488	0.000322	0.0033927	Lvrn	18	46850039	46905446	+		3219	protein_coding	laeverin [Source:MGI Symbol;Acc:MGI:1921824]	-
ENSMUSG0000004160	10.02102279	13.69247095	8.937269082	0	0	0	10.88358761	0	5.977981926	0.0003227	0.0033982	Entpd3	9	120539818	120568327	+		3755	protein_coding	ectonucleoside triphosphate diphosphohydrolase 3 [Source:MGI Symbol;Acc:MGI:1321386]	-
ENSMUSG0000004290	1249.621541	1057.480064	880.3210046	647.7209764	650.8602849	687.9289817	1062.474203	662.170081	0.682565195	0.0003249	0.003417	Foxo4	X	101254528	101260873	+		3485	protein_coding	forkhead box O4 [Source:MGI Symbol;Acc:MGI:1891915]	Fork
ENSMUSG0000002327	350.7357975	335.9921718	348.5534942	661.5022737	486.9106532	545.8672683	345.0938212	564.7600651	-0.711295109	0.0003249	0.003417	Twf2	9	106203108	106215389	+		2319	protein_coding	twinfilin actin binding protein 2 [Source:MGI Symbol;Acc:MGI:1346078]	-
ENSMUSG0000003718	62.13034127	55.82315079	62.56088357	110.250379	133.3325318	131.3584336	60.17145855	124.9804481	-1.053686385	0.0003257	0.003424	Krt80	15	101347444	101370162	-		6393	protein_coding	keratin 80 [Source:MGI Symbol;Acc:MGI:1921377]	-
ENSMUSG0000004931	521.0931849	467.6505463	414.4658537	589.1504625	1876.531929	1006.108299	467.7365283	1157.263563	-1.30652852	0.0003271	0.0034347	Sorl1	9	41964720	42124297	-		11321	protein_coding	sortilin-related receptor, LDLR class A repeats-containing [Source:MGI Symbol;Acc:MGI:1202296]	-
ENSMUSG0000004072	1.002102279	3.159800988	0	99.91440593	5.925890302	8.757228904	1.387301089	38.19917505	-4.775856109	0.0003271	0.0034347	Esrp1	4	11331933	11386783	-		6305	protein_coding	epithelial splicing regulatory protein 1 [Source:MGI Symbol;Acc:MGI:1917326]	-
ENSMUSG0000001459	916.9235849	501.3550901	897.0783841	1329.033865	1881.470171	1355.424429	771.7856864	1521.976155	-0.97957755	0.0003273	0.0034349	Csf1	3	107741048	107760469	-		5281	protein_coding	colony stimulating factor 1 (macrophage) [Source:MGI Symbol;Acc:MGI:1339753]	-
ENSMUSG0000001699	30.06306836	15.79900494	37.9833936	73.21314228	90.8636513	82.70716187	27.94848897	82.26131848	-1.558948247	0.0003277	0.0034366	Matn4	2	164389393	164405160	-		5174	protein_coding	matrilin 4 [Source:MGI Symbol;Acc:MGI:1328314]	-
ENSMUSG0000002999	844.7722209	807.855786	775.3080928	1267.018027	1069.6232	1303.854081	809.3120332	1213.498436	-0.584393498	0.0003286	0.0034441	Anxa4	6	86736840	86793584	-		4645	protein_coding	annexin A4 [Source:MGI Symbol;Acc:MGI:88030]	-
ENSMUSG0000000602	166.3489783	150.6171804	184.3311748	306.6338665	251.8503379	327.9095712	167.0991112	295.4645918	-0.823061601	0.0003293	0.0034497	Kptn	7	16119895	16127516	+		3341	protein_coding	kaptin [Source:MGI Symbol;Acc:MGI:1890380]	-
ENSMUSG0000009660	182.3826147	96.90056364	205.5571889	61.15450708	74.07362878	61.30060233	161.6134557	65.5095794	1.30311508	0.0003298	0.0034527	Tpbgl	7	99624082	99627103	-		3022	protein_coding	trophoblast glycoprotein-like [Source:MGI Symbol;Acc:MGI:3646425]	-
ENSMUSG0000002962	1507.161827	1264.973662	1269.09221	991.3920795	798.0198941	791.0696777	1347.0759	860.1605504	0.646873092	0.0003306	0.0034593	Pdap1	5	145128769	145140238	-		5245	protein_coding	PDGFA associated protein 1 [Source:MGI Symbol;Acc:MGI:2448536]	-
ENSMUSG0000001937	606.2718786	649.8657366	607.7342976	836.3524841	959.0065806	1041.137214	621.2906376	945.4987596	-0.605470486	0.0003321	0.0034734	Cops3	11	59817795	59839838	-		3454	protein_coding	COP9 signalosome subunit 3 [Source:MGI Symbol;Acc:MGI:1349409]	-
ENSMUSG0000005990	52.10931849	77.94175771	81.55258037	161.9302441	163.9496317	130.3854081	70.53455219	152.088428	-1.110981728	0.0003339	0.0034875	Adamts14	10	61197112	61273438	-		5197	protein_coding	a disintegrin-like and metallopeptidase (reprolysin type) with thrombospondin type 1 motif, 14 [Source:MGI Symbol;Acc:MGI:217	-
ENSMUSG0000006631	196.4120466	87.42116068	194.3856025	71.4904801	56.29595787	52.54337342	159.4062699	60.10993713	1.405432809	0.000334	0.0034875	Rtp3	9	110984935	110990583	-		3057	protein_coding	receptor transporter protein 3 [Source:MGI Symbol;Acc:MGI:2446841]	-
ENSMUSG0000002007	45.09460254	49.50354882	70.38099402	22.39460823	7.90118707	8.757228904	54.99304846	13.01767473	2.065670566	0.000334	0.0034875	Pbld2	10	63024315	63058813	+		2480	protein_coding	phenazine biosynthesis-like protein domain containing 2 [Source:MGI Symbol;Acc:MGI:1914557]	-
ENSMUSG0000002091	42.0882957	86.36789368	86.02121491	241.172704	121.4807512	211.1465191	71.4924681	191.2666581	-1.422461637	0.0003377	0.0035223	Top2a	11	98992943	99024189	-		9013	protein_coding	topoisomerase (DNA) II alpha [Source:MGI Symbol;Acc:MGI:98790]	-
ENSMUSG0000006392	14.0294319	43.18394684	29.04612452	5.167986514	0.987648384	3.892101735	28.75316775	3.349245544	3.085198698	0.0003377	0.0035223	Cyp4a32	4	115600969	115621602	+		1536	protein_coding	cytochrome P450, family 4, subfamily a, polypeptide 32 [Source:MGI Symbol;Acc:MGI:3717148]	-
ENSMUSG0000004909	51.10721621	65.30255376	102.7785944	24.1172704	25.67885798	22.37958498	73.06278814	24.05857112	1.600600578	0.0003386	0.0035283	Gpr137c	14	45219717	45282725	+		8776	protein_coding	G protein-coupled receptor 137C [Source:MGI Symbol;Acc:MGI:1917963]	-
ENSMUSG0000004454	195.4099443	103.2201656	118.4188153	280.7939339	317.0351312	275.3661978	139.0163084	291.0650876	-1.064355028	0.0003387	0.0035283	Dact1	12	71309884	71320107	+		3761	protein_coding	dishevelled-binding antagonist of beta-catenin 1 [Source:MGI Symbol;Acc:MGI:1891740]	-
ENSMUSG0000008592	29.06096608	23.17187391	42.45202814	7.75197977	2.962945151	5.838152603	31.56162271	5.517692508	2.503820558	0.0003395	0.0035343	Gm12781	7	34653184	34660416	+		395	antisense	predicted gene 12781 [Source:MGI Symbol;Acc:MGI:3649569]	-
ENSMUSG0000007479	2837.953653	1564.101489	5713.149261	1089.583823	1443.941937	1384.615192	3371.734801	1306.046984	1.368282038	0.0003396	0.0035343	Arrdc3	13	80883384	80896042	+		6266	protein_coding	arrestin domain containing 3 [Source:MGI Symbol;Acc:MGI:2145242]	-
ENSMUSG0000003490	4014.421728	4738.648215	4687.597563	2764.011454	3384.671011	2846.099394	4480.222526	2998.26062	0.579487277	0.0003426	0.0035633	Sidt2	9	45937857	45955258	-		5146	protein_coding	SID1 transmembrane family, member 2 [Source:MGI Symbol;Acc:MGI:2446134]	-
ENSMUSG0000000621	1034.169552	778.3643101	798.7684242	617.5743884	350.6151762	376.5608429	870.4340953	448.2501358	0.956653301	0.0003436	0.0035718	Fblim1	4	141576062	141606096	-		4457	protein_coding	filamin binding LIM protein 1 [Source:MGI Symbol;Acc:MGI:1921452]	-
ENSMUSG0000003656	929.9509146	920.5553546	882.5553218	578.8144895	671.6009009	599.3836672	911.0205303	616.5996859	0.56359305	0.0003473	0.003608	Ppp6r2	15	89211553	89287010	+		9345	protein_coding	protein phosphatase 6, regulatory subunit 2 [Source:MGI Symbol;Acc:MGI:1918724]	-
ENSMUSG0000002670	4768.002642	5408.526025	5879.605897	7705.467892	7583.16429	7664.521342	5352.044855	7651.051175	-0.515642887	0.0003475	0.003608	Prdx6	1	161240112	161251219	-		4804	protein_coding	peroxiredoxin 6 [Source:MGI Symbol;Acc:MGI:894320]	-
ENSMUSG0000006195	690.44847	955.3131655	824.4630728	1278.215331	1312.584702	1264.933064	823.4082361	1285.244366	-0.642555566	0.0003501	0.0036334	Ppp4r1	17	65782573	65841926	+		5449	protein_coding	protein phosphatase 4, regulatory subunit 1 [Source:MGI Symbol;Acc:MGI:1917601]	-
ENSMUSG0000002795	817.7154594	897.3834807	745.1448097	1180.023587	1194.066896	1249.364657	820.0812499	1207.81838	-0.558323249	0.0003513	0.0036441	Slc35a3	3	116669470	116712831	-		10786	protein_coding	solute carrier family 35 (UDP-N-acetylglucosamine (UDP-GlcNAc) transporter), member 3 [Source:MGI Symbol;Acc:MGI:19176	-
ENSMUSG0000002813	2767.806494	2467.804572	3050.960233	1962.973544	1833.0754	1895.453545	2762.190433	1897.167496	0.541829671	0.0003523	0.0036517	Adh5	3	138443093	138455499	+		5408	protein_coding	alcohol dehydrogenase 5 (class III), chi polypeptide [Source:MGI Symbol;Acc:MGI:87929]	-
ENSMUSG0000002413	4500.441333	4312.075082	2523.661357	1727.830158	2178.752335	2226.282192	3778.725924	2044.288228	0.886486507	0.0003536	0.0036631	Eci1	17	24426683	24439316	+		1091	protein_coding	enoyl-Coenzyme A delta isomerase 1 [Source:MGI Symbol;Acc:MGI:94871]	-
ENSMUSG0000000536	957.0076761	1066.959467	968.5765367	528.8572866	707.1562428	663.6033458	997.51456	633.2056251	0.656283374	0.0003539	0.003665	Crbn	6	106780201	106800077	-		6982	protein_coding	cereblon [Source:MGI Symbol;Acc:MGI:1913277]	-
ENSMUSG0000009094	507.063753	540.325969	685.935402	1004.312046	821.7234553	1017.784604	577.7750413	947.9400349	-0.714958733	0.0003547	0.0036713	Ccdc71l	12	32378704	32382943	+		4240	protein_coding	coiled-coil domain containing 71 like [Source:MGI Symbol;Acc:MGI:1919373]	-
ENSMUSG0000005306	192.4036375	105.3266996	42.45202814	372.9563601	256.7885798	435.9153943	113.3941217	355.2201114	-1.645411039	0.0003592	0.0037154	Clec12a	6	129342691	129365303	+		5528	protein_coding	C-type lectin domain family 12, member a [Source:MGI Symbol;Acc:MGI:3040968]	-
ENSMUSG0000001045	2251.72382	2631.060956	2045.517461	1487.518785	1446.904882	1652.197187	2309.434079	1528.873618	0.595167776	0.0003598	0.0037193	Kansl3	1	36335730	36369181	-		6742	protein_coding	KAT8 regulatory NSL complex subunit 3 [Source:MGI Symbol;Acc:MGI:1918055]	-
ENSMUSG0000004348	919.9298918	1111.196681	1190.891105	745.9127201	631.1073172	683.0638545	1074.005893	686.6946306	0.644667136	0.0003608	0.0037276	Gm6863	12	96922423	96923058	-		636	processed_pseudog	predicted gene 6863 [Source:MGI Symbol;Acc:MGI:3647386]	-
ENSMUSG0000000137	430.9039798	430.7862014	427.8717573	620.1583816	619.2555366	709.3355412	429.8539795	649.5831532	-0.595478521	0.000361	0.0037276	Vps50	6	3498382	3603531	+		10856	protein_coding	VPS50 EARP/GARPII complex subunit [Source:MGI Symbol;Acc:MGI:1920538]	-
ENSMUSG0000000773	3270.861837	3309.364902	3530.221287	2161.079694	2601.465843	2245.742701	3370.149342	2336.096079	0.528801753	0.0003635	0.0037514	Cct4	11	22990519	23003780	+		4201	protein_coding	chaperonin containing Tcp1, subunit 4 (delta) [Source:MGI Symbol;Acc:MGI:104689]	-
ENSMUSG0000002609	181.3805124	167.4694524	211.1429821	87.85577073	113.5795641	89.51833991	186.6643156	96.98455826	0.945332233	0.0003638	0.0037529	Asnsd1	1	53344617	53352752	-		3528	protein_coding	asparagine synthetase domain containing 1 [Source:MGI Symbol;Acc:MGI:1917646]	-
ENSMUSG0000002579	350.7357975	326.5127688	224.5488857	772.6139838	472.0959274	533.2179377	300.5991507	592.6426163	-0.97916975	0.0003646	0.0037585	Slco3a1	7	74275419	74554780	-		8474	protein_coding	solute carrier organic anion transporter family, member 3a1 [Source:MGI Symbol;Acc:MGI:1351867]	-
ENSMUSG0000008140	399.8388092	567.7109109	531.7675104	300.6045489	309.1339441	268.5550197	499.7724101	292.7645042	0.77100901	0.0003648	0.0037585	Rps6-ps4	2	75191073	75191822	-		750	processed_pseudog	ribosomal protein S6, pseudogene 4 [Source:MGI Symbol;Acc:MGI:3650907]	-
ENSMUSG0000003995	702.4736973	979.5383064	1178.60236	596.0411112	516.5401047	491.3778441	953.5381213	534.65302	0.834017694	0.000365	0.0037585	Etfbkmt	6	149139849	149151171	+		2496	protein_coding	electron transfer flavoprotein beta subunit lysine methyltransferase [Source:MGI Symbol;Acc:MGI:2443575]	-
ENSMUSG0000002661	7.01471595	2.106533992	5.585793176	99.91440593	9.876483837	31.13681388	4.902347706	46.97590122	-3.261240639	0.0003708	0.0038143	Esrrg	1	187608791	188214885	+		6208	protein_coding	estrogen-related receptor gamma [Source:MGI Symbol;Acc:MGI:1347056]	ESR-
ENSMUSG0000005029	194.4078421	181.1619233	148.5820985	106.8050546	70.12303525	72.0038821	174.717288	82.97732399	1.071965426	0.0003708	0.0038143	Gm9843	16	76403251	76403652	-		402	processed_pseudog	predicted gene 9843 [Source:MGI Symbol;Acc:MGI:3708621]	-
ENSMUSG0000003814	191.4015352	249.6242781	252.4778516	336.7804545	430.6146953	483.5936406	231.1678883	416.9962635	-0.851181946	0.0003728	0.0038303	Cd84	1	171839697	171890718	+		6272	protein_coding	CD84 antigen [Source:MGI Symbol;Acc:MGI:1336885]	-
ENSMUSG0000004225	56.1177276	114.8061026	90.48984945	36.17590559	28.64180313	26.27168671	87.13789321	30.36313181	1.517513135	0.0003728	0.0038303	Isl1	13	116298281	116309689	-		3959	protein_coding	ISL1 transcription factor, LIM/homeodomain [Source:MGI Symbol;Acc:MGI:101791]	Hom
ENSMUSG0000002324	1182.480689	1055.37353	1572.959358	1924.213645	2144.184641	2128.979649	1270.271192	2065.792645	-0.70174194	0.000373	0.0038303	Parp3	9	106470322	106476949	-		3699	protein_coding	poly (ADP-ribose) polymerase family, member 3 [Source:MGI Symbol;Acc:MGI:1891258]	-
ENSMUSG0000002311	2340.910923	2367.744207	2067.860634	1542.643974	1677.026956	1487.755888	2258.838588	1569.142273	0.525759531	0.0003738	0.0038361	Sympk	7	19024377	19054618	+		10534	protein_coding	symplekin [Source:MGI Symbol;Acc:MGI:1915438]	-
ENSMUSG0000004860	28.0588638	31.59800988	67.02951811	9.474641942	10.86413222	5.838152603	42.22879727	8.725642255	2.27139722	0.000375	0.0038469	Gm5763	X	142631483	142634015	-		1530	processed_pseudog	predicted pseudogene 5763 [Source:MGI Symbol;Acc:MGI:3647841]	-
ENSMUSG0000005555	958.0097784	861.5724028	938.4132536	608.0997464	614.3172947	661.657295	919.3318116	628.0247787	0.549932384	0.0003767	0.0038598	Kxd1	8	70508272	70527956	-		3176	protein_coding	KxDL motif containing 1 [Source:MGI Symbol;Acc:MGI:1922870]	-
ENSMUSG0000002774	895.8794371	864.7322038	1082.526718	1485.796123	1369.868308	1410.886879	947.7127861	1422.18377	-0.585987324	0.0003768	0.0038598	Ufm1	3	53853376	53863830	-		5958	protein_coding	ubiquitin-fold modifier 1 [Source:MGI Symbol;Acc:MGI:1915140]	-
ENSMUSG0000005703	274.5760243	386.5489876	261.4151206	192.0768321	127.4066415	144.9807896	307.5133775	154.8214211	0.988550934	0.0003769	0.0038598	Gm7536	3	24333068	24333511	+		444	processed_pseudog	predicted gene 7536 [Source:MGI Symbol;Acc:MGI:3645137]	-
ENSMUSG0000002255	1079.264154	1419.803911	1064.652179	789.8406055	746.6621781	721.0118464	1187.906748	752.5048767	0.658534916	0.0003776	0.0038653	Mroh1	15	76380261	76453038	+		16468	protein_coding	maestro heat-like repeat family member 1 [Source:MGI Symbol;Acc:MGI:2442558]	-
ENSMUSG0000005481	430.9039798	450.7982743	456.9178818	614.129064	829.6246423	693.7671343	446.206712	712.5069469	-0.674852038	0.000378	0.0038671	Usp46	5	73998453	74068431	-		7841	protein_coding	ubiquitin specific peptidase 46 [Source:MGI Symbol;Acc:MGI:1916977]	-
ENSMUSG0000002036	2201.618706	2366.69094	2632.025745	3301.482051	3710.594978	3383.209433	2400.111797	3465.095487	-0.529868064	0.0003799	0.0038838	Hspa4	11	53259814	53300457	-		5528	protein_coding	heat shock protein 4 [Source:MGI Symbol;Acc:MGI:1342292]	-
ENSMUSG0000003915	4610.672584	3990.828648	3227.471297	2118.874471	2769.366068	2400.453745	3942.990843	2429.564761	0.698796785	0.0003807	0.0038893	Ube2h	6	30211289	30304539	-		4780	protein_coding	ubiquitin-conjugating enzyme E2H [Source:MGI Symbol;Acc:MGI:104632]	-
ENSMUSG0000003923	519.0889803	282.275555	746.2619683	200.6901429	201.4802703	260.7708163	515.8755012	220.9804098	1.223018432	0.0003808	0.0038893	Isg20	7	78913424	78920396	+		1860	protein_coding	interferon-stimulated protein [Source:MGI Symbol;Acc:MGI:1928895]	-
ENSMUSG0000002414	15414.33725	11956.68694	14404.64344	6728.718441	9779.694296	9113.356213	13925.22254	8540.58965	0.705347625	0.0003814	0.0038936	Epas1	17	86753907	86833410	+		5309	protein_coding	endothelial PAS domain protein 1 [Source:MGI Symbol;Acc:MGI:109169]	-
ENSMUSG0000004653	1575.304782	1157.540429	1012.145724	1808.79528	2223.196512	2466.619475	1248.330311	2166.203755	-0.794782355	0.0003821	0.0038978	Ar	X	98148769	98323215	+		10048	protein_coding	androgen receptor [Source:MGI Symbol;Acc:MGI:88064]	ESR-
ENSMUSG0000002575	472.9922755	423.4133324	521.7130826	626.1876992	904.6859195	880.5880176	472.7062302	803.8205454	-0.765643984	0.0003829	0.0039037	Mfsd8	3	40818103	40846886	-		9414	protein_coding	major facilitator superfamily domain containing 8 [Source:MGI Symbol;Acc:MGI:1919425]	-
ENSMUSG0000000334	84.1765914	84.26135969	45.80350404	132.6449872	213.3320509	167.3603746	71.41381838	171.1124709	-1.25746569	0.000383	0.0039037	Mob3a	10	80685253	80701977	-		3254	protein_coding	MOB kinase activator 3A [Source:MGI Symbol;Acc:MGI:3050117]	-
ENSMUSG0000004733	305.641195	377.0695846	1121.62727	283.3779272	127.4066415	131.3584336	601.4460164	180.7143341	1.733304317	0.0003834	0.0039051	Kcne4	1	78816758	78820028	+		2657	protein_coding	potassium voltage-gated channel, Isk-related subfamily, gene 4 [Source:MGI Symbol;Acc:MGI:1891125]	-
ENSMUSG0000003315	1239.600519	1371.353629	1393.096818	934.5442279	922.4635904	946.7537471	1334.683655	934.5871885	0.513935425	0.0003841	0.00391	Cnppd1	1	75134554	75142711	-		5635	protein_coding	cyclin Pas1/PHO80 domain containing 1 [Source:MGI Symbol;Acc:MGI:1916421]	-
ENSMUSG0000002902	506.0616507	534.006367	857.9778318	376.4016844	329.8745602	276.3392232	632.6819499	327.5384893	0.948854769	0.0003846	0.0039133	Miip	4	147860778	147868816	-		3354	protein_coding	migration and invasion inhibitory protein [Source:MGI Symbol;Acc:MGI:106506]	-
ENSMUSG0000003898	396.8325023	384.4424536	269.2352311	565.0331921	574.8113593	682.0908291	350.1700623	607.3117935	-0.793419098	0.000385	0.0039147	Bloc1s5	13	38592842	38635109	-		6439	protein_coding	biogenesis of lysosomal organelles complex-1, subunit 5, muted [Source:MGI Symbol;Acc:MGI:2178598]	-
ENSMUSG0000003979	441.9271049	444.4786724	550.7592072	315.2471773	282.4674378	288.9885538	479.0549948	295.567723	0.695735238	0.0003867	0.0039301	Zfand1	3	10339953	10351317	-		6267	protein_coding	zinc finger, AN1-type domain 1 [Source:MGI Symbol;Acc:MGI:1913611]	-
ENSMUSG0000003762	624.3097196	310.7137638	326.2103215	229.1140688	172.8384672	164.4412983	420.4112683	188.7979447	1.154728272	0.0003883	0.0039445	Atoh8	6	72206177	72235577	-		2507	protein_coding	atonal bHLH transcription factor 8 [Source:MGI Symbol;Acc:MGI:1918343]	bHLH
ENSMUSG0000000354	398.8367069	544.539037	420.0516468	252.3700081	287.4056797	171.2524763	454.4757969	237.009388	0.939062864	0.0003898	0.0039575	Ier3	17	35821684	35822923	-		1131	protein_coding	immediate early response 3 [Source:MGI Symbol;Acc:MGI:104814]	-
ENSMUSG0000003007	126.2648871	92.68749566	100.5442772	368.6497046	198.5173251	180.9827307	106.4988866	249.3832535	-1.228171181	0.0003908	0.0039658	Gxylt2	6	100704734	100810913	+		8510	protein_coding	glucoside xylosyltransferase 2 [Source:MGI Symbol;Acc:MGI:2682940]	-
ENSMUSG0000002811	244.512956	347.5781087	167.5737953	722.6567808	365.429902	677.2257019	253.22162	588.4374616	-1.216356056	0.0003912	0.0039676	Ctsk	3	95499256	95509362	+		1512	protein_coding	cathepsin K [Source:MGI Symbol;Acc:MGI:107823]	-
ENSMUSG0000004284	4.008409114	1.053266996	0	41.34389211	5.925890302	40.86706822	1.68722537	29.37895021	-4.093738919	0.000392	0.0039737	Lrrtm3	10	63928472	64090277	-		4251	protein_coding	leucine rich repeat transmembrane neuronal 3 [Source:MGI Symbol;Acc:MGI:2389177]	-
ENSMUSG0000002664	977.0497217	1228.109317	1110.455683	646.8596453	736.7856943	769.6631181	1105.204908	717.7694859	0.622940186	0.0003927	0.0039779	Nmt2	2	3284212	3328877	+		6556	protein_coding	N-myristoyltransferase 2 [Source:MGI Symbol;Acc:MGI:1202298]	-
ENSMUSG0000003883	142.2985236	189.5880593	87.13837355	271.319292	342.7139892	293.853681	139.6749855	302.6289874	-1.114001109	0.0003945	0.0039928	Ralgps1	2	33133417	33371486	-		7657	protein_coding	Ral GEF with PH domain and SH3 binding motif 1 [Source:MGI Symbol;Acc:MGI:1922008]	-
ENSMUSG0000004061	893.8752325	1062.746399	843.4547696	679.5902265	498.7624338	504.0271747	933.3588004	560.7932783	0.734333883	0.0003947	0.0039928	Pck2	14	55540266	55551242	+		7806	protein_coding	phosphoenolpyruvate carboxykinase 2 (mitochondrial) [Source:MGI Symbol;Acc:MGI:1860456]	-
ENSMUSG0000002800	331.6958542	460.2776773	281.5239761	588.2891315	638.0208559	681.1178036	357.8325025	635.8092637	-0.828816787	0.0003948	0.0039928	Snx7	3	117781647	117868936	-		6147	protein_coding	sorting nexin 7 [Source:MGI Symbol;Acc:MGI:1923811]	-
ENSMUSG0000002824	4549.544345	5508.58639	4988.113306	3547.822742	3583.188336	3474.673824	5015.41468	3535.228301	0.504534689	0.000401	0.0040514	Sdcbp	4	6365650	6408423	+		6904	protein_coding	syndecan binding protein [Source:MGI Symbol;Acc:MGI:1337026]	-
ENSMUSG0000001844	274.5760243	372.8565166	418.9344882	212.7487781	196.5420284	175.1445781	355.4556764	194.8117949	0.866278124	0.000401	0.0040514	Rpain	11	70970213	70977833	+		2842	protein_coding	RPA interacting protein [Source:MGI Symbol;Acc:MGI:1916973]	-
ENSMUSG0000002054	704.4779019	824.7080579	1023.31731	573.646503	448.3923662	487.4857423	850.8344232	503.1748705	0.756946529	0.0004031	0.0040698	Bzw2	12	36091835	36158080	-		5413	protein_coding	basic leucine zipper and W2 domains 2 [Source:MGI Symbol;Acc:MGI:1914162]	-
ENSMUSG0000004631	197.4141489	210.6533992	127.3560844	285.1005893	403.948189	366.8305885	178.4745442	351.9597889	-0.977970056	0.0004033	0.0040698	Stxbp6	12	44852484	45074709	-		5297	protein_coding	syntaxin binding protein 6 (amisyn) [Source:MGI Symbol;Acc:MGI:2384963]	-
ENSMUSG0000004875	1106.320916	1615.711572	1705.901236	876.8350451	932.3400743	834.8558222	1475.977908	881.3436472	0.743684149	0.000404	0.0040747	Foxo3	10	42181841	42276755	-		7571	protein_coding	forkhead box O3 [Source:MGI Symbol;Acc:MGI:1890081]	Fork
ENSMUSG0000002701	767.6103454	764.6718392	790.9483137	532.3026109	435.5529372	546.8402938	774.4101661	504.898614	0.616704957	0.0004056	0.0040895	Cwc22	2	77881159	77946375	-		6822	protein_coding	CWC22 spliceosome-associated protein [Source:MGI Symbol;Acc:MGI:2136773]	-
ENSMUSG0000003049	209.4393762	187.4815253	203.3228716	111.11171	122.4683996	101.1946451	200.0812577	111.5915849	0.842633021	0.0004095	0.0041237	Gas2	7	51862015	51994975	+		4993	protein_coding	growth arrest specific 2 [Source:MGI Symbol;Acc:MGI:95657]	-
ENSMUSG0000003467	283.5949448	283.328822	302.7499901	450.4761578	441.4788275	440.7805215	289.8912523	444.2451689	-0.616217266	0.0004095	0.0041237	Tdg	10	82629828	82650799	+		5374	protein_coding	thymine DNA glycosylase [Source:MGI Symbol;Acc:MGI:108247]	-
ENSMUSG0000002804	249.5234674	131.6583745	268.1180725	508.1853405	512.5895112	362.9384868	216.4333048	461.2377795	-1.091971965	0.0004097	0.0041238	Thbs3	3	89215180	89226837	+		4823	protein_coding	thrombospondin 3 [Source:MGI Symbol;Acc:MGI:98739]	-
ENSMUSG0000005892	265.5571038	278.062487	223.431727	152.4556021	142.2213673	149.8459168	255.6837726	148.1742954	0.787269893	0.0004111	0.0041338	Gm10052	9	123689233	123690192	-		960	processed_pseudog	predicted pseudogene 10052 [Source:MGI Symbol;Acc:MGI:3704348]	-
ENSMUSG0000003219	1086.27887	1269.18673	520.595924	1763.144732	2123.444025	2171.792768	958.6871748	2019.460508	-1.07451525	0.0004111	0.0041338	Kank2	9	21766784	21798744	-		5235	protein_coding	KN motif and ankyrin repeat domains 2 [Source:MGI Symbol;Acc:MGI:2384568]	-
ENSMUSG0000007474	684.4358563	610.8948577	1000.974137	456.5054754	405.9234857	429.1042163	765.4349504	430.5110591	0.829618388	0.0004142	0.004162	Kiz	2	146855864	146970097	+		2278	protein_coding	kizuna centrosomal protein [Source:MGI Symbol;Acc:MGI:2684960]	-
ENSMUSG0000009453	26.05465924	17.90553893	17.87453816	54.26385839	63.20949656	62.27362776	20.61157878	59.9156609	-1.535371903	0.0004159	0.0041768	Gm21399	8	129252998	129253594	+		597	processed_pseudog	predicted gene, 21399 [Source:MGI Symbol;Acc:MGI:5434754]	-
ENSMUSG0000004410	33.06937519	25.27840791	26.81180725	1.722662171	7.90118707	0.973025434	28.38653012	3.532291558	3.023310174	0.0004162	0.0041782	Il1f9	2	24186476	24193568	+		1648	protein_coding	interleukin 1 family, member 9 [Source:MGI Symbol;Acc:MGI:2449929]	-
ENSMUSG0000005935	338.7105702	396.0283905	504.9557031	664.086267	693.3291654	701.5513378	413.2315546	686.3222567	-0.732709322	0.0004177	0.0041911	Wdr83os	8	85080740	85082339	+		1031	protein_coding	WD repeat domain 83 opposite strand [Source:MGI Symbol;Acc:MGI:3041257]	-
ENSMUSG0000003461	545.1436396	685.6768145	554.1106831	406.5482724	358.5163633	344.4510036	594.9770457	369.8385464	0.685394901	0.0004198	0.0042091	Ssh3	19	4261668	4269172	-		2740	protein_coding	slingshot protein phosphatase 3 [Source:MGI Symbol;Acc:MGI:2683546]	-
ENSMUSG0000003917	1493.132395	1492.479333	1316.012872	956.077505	1048.882584	975.9445101	1433.874867	993.6348662	0.529433342	0.0004228	0.0042348	Polg	7	79446231	79466362	-		10742	protein_coding	polymerase (DNA directed), gamma [Source:MGI Symbol;Acc:MGI:1196389]	-
ENSMUSG0000002518	678.4232426	716.2215573	1051.246276	467.7027795	456.2935533	491.3778441	815.2970252	471.7913923	0.788743151	0.0004229	0.0042348	R3hcc1l	19	42518759	42592343	+		7397	protein_coding	R3H domain and coiled-coil containing 1 like [Source:MGI Symbol;Acc:MGI:1196316]	-
ENSMUSG0000003253	579.215117	539.272702	688.1697193	395.3509683	389.1334632	304.5569608	602.2191794	363.0137974	0.729595494	0.000423	0.0042348	Cep63	9	102584588	102626534	-		9598	protein_coding	centrosomal protein 63 [Source:MGI Symbol;Acc:MGI:2158560]	-
ENSMUSG0000002246	238.5003423	105.3266996	232.3689961	90.43976399	61.23419979	74.9229584	192.065346	75.53230739	1.344946253	0.0004244	0.0042463	Endou	15	97711015	97731339	-		2745	protein_coding	endonuclease, polyU-specific [Source:MGI Symbol;Acc:MGI:97746]	-
ENSMUSG0000006979	32.06727292	55.82315079	32.39760042	109.3890479	81.97481585	119.6821284	40.09600804	103.6819974	-1.370882436	0.0004248	0.0042481	Slfn9	11	82978390	82991830	-		5965	protein_coding	schlafen 9 [Source:MGI Symbol;Acc:MGI:2445121]	-
ENSMUSG0000002442	135.2838076	163.2563844	145.2306226	250.6473459	270.6156571	240.3372821	147.9236049	253.8667617	-0.779372349	0.0004251	0.004249	Atat1	17	35897595	35910075	-		4586	protein_coding	alpha tubulin acetyltransferase 1 [Source:MGI Symbol;Acc:MGI:1913869]	-
ENSMUSG0000001583	8372.564538	10200.89086	6605.75901	5139.562588	5007.377306	5525.811438	8393.071468	5224.250444	0.684007076	0.0004257	0.004253	Sqstm1	11	50199366	50210827	-		3356	protein_coding	sequestosome 1 [Source:MGI Symbol;Acc:MGI:107931]	-
ENSMUSG0000002053	7778.317887	11138.29848	5326.612373	8560.76966	34333.62076	24541.64749	8081.076248	22478.6793	-1.475916463	0.0004266	0.0042598	Acaca	11	84129672	84401651	+		17074	protein_coding	acetyl-Coenzyme A carboxylase alpha [Source:MGI Symbol;Acc:MGI:108451]	-
ENSMUSG0000000164	1249.621541	978.4850394	1155.142029	1515.942711	1862.704852	1878.912113	1127.749537	1752.519892	-0.635723583	0.0004282	0.0042729	Akr1b3	6	34302434	34317478	-		8384	protein_coding	aldo-keto reductase family 1, member B3 (aldose reductase) [Source:MGI Symbol;Acc:MGI:1353494]	-
ENSMUSG0000007949	168.3531828	152.7237144	149.6992571	223.0847512	359.5040117	306.5030116	156.9253848	296.3639248	-0.915822488	0.0004322	0.0043067	6530402F18Ri	2	29245107	29253006	-		5511	processed_transcrip	RIKEN cDNA 6530402F18 gene [Source:MGI Symbol;Acc:MGI:1923470]	-
ENSMUSG0000002646	5731.022931	3547.403243	4026.239721	6206.751803	8302.172314	9393.587538	4434.888632	7967.503885	-0.845139128	0.0004322	0.0043067	Atp2b4	1	133699457	133801041	-		12248	protein_coding	ATPase, Ca++ transporting, plasma membrane 4 [Source:MGI Symbol;Acc:MGI:88111]	-
ENSMUSG0000004567	76.15977317	47.39701482	40.21771087	203.2741362	110.616619	120.6551538	54.59149962	144.8486363	-1.406601126	0.0004323	0.0043067	Col27a1	4	63214004	63334991	+		13022	protein_coding	collagen, type XXVII, alpha 1 [Source:MGI Symbol;Acc:MGI:2672118]	-
ENSMUSG0000002502	734.5409702	852.0929998	709.3957334	555.5585502	425.6764534	450.5107758	765.3432345	477.2485931	0.680689423	0.0004331	0.0043132	Mxi1	19	53310506	53375810	+		5619	protein_coding	MAX interactor 1, dimerization protein [Source:MGI Symbol;Acc:MGI:97245]	bHLH
ENSMUSG0000002127	418.8787525	409.7208615	377.5996187	242.8953661	275.5538991	244.2293839	402.0664109	254.2262164	0.662061172	0.0004339	0.0043182	Zfp839	12	110850253	110869996	+		3749	protein_coding	zinc finger protein 839 [Source:MGI Symbol;Acc:MGI:1920055]	-
ENSMUSG0000003479	29.06096608	23.17187391	30.16328315	55.12518948	91.85129969	79.78808557	27.46537438	75.58819158	-1.459025225	0.0004345	0.0043221	Gna15	10	81502306	81524225	-		2135	protein_coding	guanine nucleotide binding protein, alpha 15 [Source:MGI Symbol;Acc:MGI:95770]	-
ENSMUSG0000002961	8041.870786	8966.461938	7526.297725	6310.972864	4931.32838	4698.73982	8178.21015	5313.680355	0.621996222	0.0004366	0.004341	Rpl6	5	121204481	121209241	+		3140	protein_coding	ribosomal protein L6 [Source:MGI Symbol;Acc:MGI:108057]	-
ENSMUSG0000002013	27.05676152	46.34374783	11.17158635	86.99443964	122.4683996	84.65321274	28.19069857	98.03868399	-1.79462757	0.0004374	0.0043468	Apc2	10	80295977	80318263	+		11281	protein_coding	adenomatosis polyposis coli 2 [Source:MGI Symbol;Acc:MGI:1346052]	-
ENSMUSG0000005977	69.14505722	321.2464338	265.8837552	38.75989885	87.90070615	51.57034799	218.7584154	59.41031767	1.881775208	0.0004381	0.0043506	Rps26-ps1	8	107439131	107439579	-		449	transcribed_process	ribosomal protein S26, pseudogene 1 [Source:MGI Symbol;Acc:MGI:3704322]	-
ENSMUSG0000002515	624.3097196	800.482917	409.9972191	1141.263688	1302.708218	1026.541833	611.5966186	1156.837913	-0.919157005	0.0004389	0.0043568	Cbr2	11	120729489	120732114	-		1327	protein_coding	carbonyl reductase 2 [Source:MGI Symbol;Acc:MGI:107200]	-
ENSMUSG0000003148	162.3405691	214.8664672	120.6531326	485.7907323	303.2080538	288.0155284	165.9533896	359.0047715	-1.113385109	0.0004404	0.0043679	Slc25a15	8	22375551	22398597	-		5629	protein_coding	solute carrier family 25 (mitochondrial carrier ornithine transporter), member 15 [Source:MGI Symbol;Acc:MGI:1342274]	-
ENSMUSG0000002376	446.9376163	614.0546587	578.688173	318.6925017	316.0474828	357.1003342	546.5601493	330.6134396	0.72501221	0.0004406	0.0043679	Sfi1	11	3131850	3193463	-		7559	protein_coding	Sfi1 homolog, spindle assembly associated (yeast) [Source:MGI Symbol;Acc:MGI:1926137]	-
ENSMUSG0000008562	38.07988659	32.65127688	41.3348695	6.890648685	9.876483837	11.67630521	37.35534432	9.481145909	1.983893014	0.0004407	0.0043679	Appbp2os	11	85234775	85238304	+		1031	antisense	amyloid beta precursor protein (cytoplasmic tail) binding protein 2, opposite strand [Source:MGI Symbol;Acc:MGI:3603817]	-
ENSMUSG0000003188	41.08619342	68.46235475	44.68634541	192.9381632	103.7030803	108.9788486	51.41163119	135.2066973	-1.396861146	0.0004416	0.0043739	Rrad	8	104628070	104631327	-		1577	protein_coding	Ras-related associated with diabetes [Source:MGI Symbol;Acc:MGI:1930943]	-
ENSMUSG0000002927	288.6054562	343.3650407	179.8625403	530.5799487	515.5524563	500.135073	270.6110124	515.4224927	-0.928742484	0.0004437	0.0043924	Fam69a	5	107908053	107987085	-		2892	protein_coding	family with sequence similarity 69, member A [Source:MGI Symbol;Acc:MGI:1914516]	-
ENSMUSG0000004430	0	0	0	12.0586352	11.8517806	8.757228904	0	10.8892149	-5.808725585	0.000444	0.0043951	Apol7c	15	77524852	77533316	-		2060	protein_coding	apolipoprotein L 7c [Source:MGI Symbol;Acc:MGI:1920912]	-
ENSMUSG0000003170	1024.148529	979.5383064	1108.221366	1347.983149	1656.28634	1663.873492	1037.302734	1556.04766	-0.584915907	0.0004448	0.0043951	Itfg1	8	85717578	85840921	-		11045	protein_coding	integrin alpha FG-GAP repeat containing 1 [Source:MGI Symbol;Acc:MGI:106419]	-
ENSMUSG0000001436	1361.856997	2236.085833	1419.908625	876.8350451	850.3652584	1071.301003	1672.617152	932.8337687	0.842483005	0.0004451	0.0043951	Mertk	2	128698956	128802894	+		4336	protein_coding	c-mer proto-oncogene tyrosine kinase [Source:MGI Symbol;Acc:MGI:96965]	-
ENSMUSG0000002202	5.010511393	4.213067984	84.90405628	1.722662171	0	0	31.37587855	0.574220724	5.69857085	0.0004451	0.0043951	Olfm4	14	79984081	80023139	+		2962	protein_coding	olfactomedin 4 [Source:MGI Symbol;Acc:MGI:2685142]	-
ENSMUSG0000003027	1145.402904	858.4126018	816.6429623	1821.715246	1297.769976	1653.170212	940.1528229	1590.885145	-0.75873882	0.0004451	0.0043951	Camk1	6	113334124	113343984	-		5975	protein_coding	calcium/calmodulin-dependent protein kinase I [Source:MGI Symbol;Acc:MGI:1098535]	-
ENSMUSG0000005161	557.1688669	655.1320716	497.1355927	864.7764099	845.4270165	952.5918997	569.8121771	887.598442	-0.639127945	0.0004465	0.0044062	Rap2a	14	120478444	120507194	+		4191	protein_coding	RAS related protein 2a [Source:MGI Symbol;Acc:MGI:97855]	-
ENSMUSG0000011690	192.4036375	254.8906131	319.5073697	31.00791908	106.6660254	116.7630521	255.6005401	84.81233219	1.593970179	0.0004492	0.0044311	AC154232.2	16	36875140	36880234	+		5095	TEC	TEC	-
ENSMUSG0000000258	15.03153418	5.26633498	13.40590362	30.146588	111.6042674	38.92101735	11.23459093	60.22395757	-2.419990937	0.0004505	0.004441	Pon1	6	5168090	5193946	-		2898	protein_coding	paraoxonase 1 [Source:MGI Symbol;Acc:MGI:103295]	-
ENSMUSG0000002123	18566.95102	20428.11339	13297.53924	7274.802349	11419.19061	10664.35875	17430.86788	9786.117239	0.832885644	0.0004523	0.0044563	Aldh6a1	12	84430717	84451004	-		5784	protein_coding	aldehyde dehydrogenase family 6, subfamily A1 [Source:MGI Symbol;Acc:MGI:1915077]	-
ENSMUSG0000005874	5.010511393	0	4.468634541	18.94928388	42.4688805	25.29866128	3.159715311	28.90560855	-3.190792595	0.0004552	0.0044814	Kcnj14	7	45816460	45824782	-		2717	protein_coding	potassium inwardly-rectifying channel, subfamily J, member 14 [Source:MGI Symbol;Acc:MGI:2384820]	-
ENSMUSG0000006188	228.4793195	261.210215	294.9298797	160.2075819	152.0978511	118.7091029	261.5398048	143.671512	0.862720379	0.0004554	0.0044814	Ccdc62	5	123930679	123969895	+		8505	protein_coding	coiled-coil domain containing 62 [Source:MGI Symbol;Acc:MGI:2684996]	-
ENSMUSG0000008668	133.2796031	132.7116415	154.1678917	68.90648685	72.09833201	75.89598383	140.0530454	72.30026757	0.953921871	0.0004555	0.0044814	Gm11560	11	99932162	99933109	+		948	processed_pseudog	predicted gene 11560 [Source:MGI Symbol;Acc:MGI:3652066]	-
ENSMUSG0000008344	238.5003423	102.1668986	111.7158635	256.6766635	441.4788275	438.8344706	150.7943682	378.9966539	-1.327724596	0.0004602	0.0045233	Gm15519	6	112563642	112565207	+		1566	processed_pseudog	predicted gene 15519 [Source:MGI Symbol;Acc:MGI:3782965]	-
ENSMUSG0000006365	666.3980153	727.8074943	577.5710144	1080.109181	972.833658	948.6997979	657.2588413	1000.547546	-0.606185356	0.0004603	0.0045233	Zbtb18	1	177442351	177450764	+		6138	protein_coding	zinc finger and BTB domain containing 18 [Source:MGI Symbol;Acc:MGI:1353609]	ZBTB
ENSMUSG0000004004	2639.537402	2230.819498	2194.09956	1602.93715	1676.039307	1620.087347	2354.81882	1633.021268	0.528247904	0.000463	0.0045479	Ndufb10	17	24722060	24724478	-		1217	protein_coding	NADH:ubiquinone oxidoreductase subunit B10 [Source:MGI Symbol;Acc:MGI:1915592]	-
ENSMUSG0000002906	1555.262736	1493.5326	1987.425212	1173.99427	1065.672606	969.133332	1678.740183	1069.600069	0.649930193	0.0004655	0.0045677	Cdk11b	4	155624854	155649938	+		3752	protein_coding	cyclin-dependent kinase 11B [Source:MGI Symbol;Acc:MGI:88353]	-
ENSMUSG0000002334	602.2634694	430.7862014	469.2066268	819.1258624	793.0816521	791.0696777	500.7520992	801.0923974	-0.67742849	0.0004655	0.0045677	Trip6	5	137309654	137314404	-		2429	protein_coding	thyroid hormone receptor interactor 6 [Source:MGI Symbol;Acc:MGI:1343458]	-
ENSMUSG0000010670	37.07778431	22.11860692	65.91235948	79.24245987	178.7643575	145.9538151	41.7029169	134.6535441	-1.69265828	0.0004659	0.0045693	Gm30270	6	21852418	21853483	+		524	antisense	predicted gene, 30270 [Source:MGI Symbol;Acc:MGI:5589429]	-
ENSMUSG0000001847	815.7125468	763.6185722	608.8514562	1097.335803	1260.239338	1079.085206	729.3937611	1145.553449	-0.650736398	0.0004665	0.0045728	Kdm6b	11	69398508	69413675	-		6954	protein_coding	KDM1 lysine (K)-specific demethylase 6B [Source:MGI Symbol;Acc:MGI:2448492]	-
ENSMUSG0000002884	2253.728025	1751.583015	1813.148465	3010.352144	2880.970335	2774.095512	1939.486501	2888.472664	-0.574515177	0.000468	0.0045844	Map7d1	4	126232167	126256343	-		3652	protein_coding	MAP7 domain containing 1 [Source:MGI Symbol;Acc:MGI:2384297]	-
ENSMUSG0000005140	2024.246603	1894.827326	2289.058044	1406.553663	1469.620795	1444.942769	2069.377324	1440.372409	0.522700734	0.0004722	0.0046234	Ppp1r37	7	19530800	19563076	-		4110	protein_coding	protein phosphatase 1, regulatory subunit 37 [Source:MGI Symbol;Acc:MGI:2687042]	-
ENSMUSG0000003683	8.016818229	4.213067984	2.23431727	46.51187862	23.70356121	23.35261041	4.821401161	31.18935008	-2.683293333	0.0004733	0.0046316	Lpar3	3	146220963	146286186	+		2464	protein_coding	lysophosphatidic acid receptor 3 [Source:MGI Symbol;Acc:MGI:1929469]	-
ENSMUSG0000002064	8.016818229	30.54474289	12.28874499	94.74641942	49.38241919	65.19270406	16.95010203	69.77384756	-2.043809979	0.0004757	0.0046528	Rrm2	12	24708241	24714146	+		2771	protein_coding	ribonucleotide reductase M2 [Source:MGI Symbol;Acc:MGI:98181]	-
ENSMUSG0000005311	1.002102279	0	0	79.24245987	3.950593535	1.946050868	0.334034093	28.37970143	-6.23116774	0.0004759	0.0046528	Fank1	7	133776861	133881532	+		2299	protein_coding	fibronectin type 3 and ankyrin repeat domains 1 [Source:MGI Symbol;Acc:MGI:1914180]	-
ENSMUSG0000002533	2243.707002	1975.928885	3005.156729	1509.052062	1499.250247	1471.214456	2408.264205	1493.172255	0.689461963	0.0004767	0.004658	Sbds	5	130245731	130255530	-		2033	protein_coding	SBDS ribosome maturation factor [Source:MGI Symbol;Acc:MGI:1913961]	-
ENSMUSG0000002741	184.3868193	945.8337625	593.2112353	197.2448186	200.4926219	169.3064255	574.4772724	189.014622	1.603479279	0.0004771	0.0046596	Lpin3	2	160880670	160906002	+		3694	protein_coding	lipin 3 [Source:MGI Symbol;Acc:MGI:1891342]	-
ENSMUSG0000003774	5.010511393	1.053266996	7.820110447	62.87716925	14.81472576	33.08286475	4.627962945	36.92491992	-3.005183162	0.0004776	0.0046616	Phyhipl	10	70557682	70655965	-		9732	protein_coding	phytanoyl-CoA hydroxylase interacting protein-like [Source:MGI Symbol;Acc:MGI:1918161]	-
ENSMUSG0000000671	576.2088102	550.858639	617.7887253	420.3295698	310.1215925	352.235207	581.6187248	360.8954564	0.687394807	0.0004797	0.0046783	Acot13	13	24817948	24831540	-		744	protein_coding	acyl-CoA thioesterase 13 [Source:MGI Symbol;Acc:MGI:1914084]	-
ENSMUSG0000002307	1019.138017	964.7925684	1512.632792	558.1425435	648.8849881	759.9328638	1165.521126	655.6534651	0.830005094	0.0004798	0.0046783	Mospd1	X	53344598	53370502	-		2085	protein_coding	motile sperm domain containing 1 [Source:MGI Symbol;Acc:MGI:1917630]	-
ENSMUSG0000001998	2.004204557	2.106533992	5.585793176	200.6901429	0.987648384	11.67630521	3.232177242	71.11803218	-4.468418994	0.0004823	0.0047004	Myb	10	21124935	21160984	-		8933	protein_coding	myeloblastosis oncogene [Source:MGI Symbol;Acc:MGI:97249]	MYB
ENSMUSG0000005313	221.4646036	219.0795352	281.5239761	131.7836561	145.1843124	129.4123827	240.6893716	135.4601171	0.828894548	0.0004826	0.0047009	Mapk11	15	89142486	89149628	-		2887	protein_coding	mitogen-activated protein kinase 11 [Source:MGI Symbol;Acc:MGI:1338024]	-
ENSMUSG0000004828	1010.119097	1031.148389	624.4916771	1282.521986	1824.186565	1791.339824	888.5863877	1632.682792	-0.877187318	0.0004855	0.004726	Frmd6	12	70825514	70902234	+		6392	protein_coding	FERM domain containing 6 [Source:MGI Symbol;Acc:MGI:2442579]	-
ENSMUSG0000002860	1193.503814	1491.426066	1425.494419	1017.232012	664.6873623	757.0137875	1370.141433	812.9777206	0.752401333	0.0004894	0.004762	Echdc2	4	108165437	108179308	+		2287	protein_coding	enoyl Coenzyme A hydratase domain containing 2 [Source:MGI Symbol;Acc:MGI:1289238]	-
ENSMUSG0000004557	1406.951599	659.3451396	1022.200151	2879.429819	1630.607482	1989.837012	1029.498963	2166.624771	-1.073508162	0.0004915	0.0047795	Penk	4	4133531	4138819	-		1634	protein_coding	preproenkephalin [Source:MGI Symbol;Acc:MGI:104629]	-
ENSMUSG0000003987	1285.697223	1402.951639	1213.234278	729.5474295	904.6859195	927.2932384	1300.627713	853.8421958	0.607667823	0.0004925	0.004787	Heca	10	17868612	17948067	-		6247	protein_coding	hdc homolog, cell cycle regulator [Source:MGI Symbol;Acc:MGI:2685715]	-
ENSMUSG0000002940	234.4919332	246.4644771	191.0341266	323.8604882	429.6270469	407.6976568	223.9968456	387.0617306	-0.787728056	0.000493	0.004787	Abcb9	5	124061530	124095798	-		5601	protein_coding	ATP-binding cassette, sub-family B (MDR/TAP), member 9 [Source:MGI Symbol;Acc:MGI:1861729]	-
ENSMUSG0000002788	3046.390927	3071.326561	4733.401137	1962.112213	2344.677263	2087.139555	3617.039542	2131.309677	0.763029012	0.000493	0.004787	Prpf38b	3	108902805	108911727	-		6949	protein_coding	PRP38 pre-mRNA processing factor 38 (yeast) domain containing B [Source:MGI Symbol;Acc:MGI:1914171]	-
ENSMUSG0000002540	230.4835241	220.1328022	241.3062652	392.766975	336.7880989	374.614792	230.6408638	368.056622	-0.674939792	0.0004944	0.0047979	Ddit3	10	127290774	127296288	+		1713	protein_coding	DNA-damage inducible transcript 3 [Source:MGI Symbol;Acc:MGI:109247]	TF_b
ENSMUSG0000010769	16.03363646	13.69247095	29.04612452	2.583939257	1.975296767	0.973025434	19.59074397	1.844105153	3.394292656	0.0004963	0.0048137	Gm44430	6	88519013	88520007	+		995	TEC	predicted gene, 44430 [Source:MGI Symbol;Acc:MGI:5690822]	-
ENSMUSG0000003962	255.536081	195.9076613	282.6411347	131.7836561	118.517806	145.9538151	244.694959	132.0850924	0.889097607	0.0005001	0.004848	Trmt9b	8	36457548	36516570	+		6387	protein_coding	tRNA methyltransferase 9B [Source:MGI Symbol;Acc:MGI:2442328]	-
ENSMUSG0000003230	1610.378362	1602.019101	2111.429821	1130.927715	1211.844567	1125.790427	1774.609094	1156.18757	0.617980973	0.0005012	0.0048564	Ube2q2	9	55148854	55207529	+		4305	protein_coding	ubiquitin-conjugating enzyme E2Q family member 2 [Source:MGI Symbol;Acc:MGI:2388672]	-
ENSMUSG0000006042	2368.969787	1550.409018	1722.658616	2496.137486	3899.235819	3755.878174	1880.67914	3383.750493	-0.847153173	0.0005025	0.0048671	Sntb1	15	55636388	55906949	-		5324	protein_coding	syntrophin, basic 1 [Source:MGI Symbol;Acc:MGI:101781]	-
ENSMUSG0000004492	74.15556862	36.86434486	92.72416672	206.7194605	142.2213673	164.4412983	67.9146934	171.1273754	-1.335165538	0.0005033	0.0048716	H1fx	6	87980421	87981637	-		1217	protein_coding	H1 histone family, member X [Source:MGI Symbol;Acc:MGI:2685307]	-

ENSMUSG0000002738	32.06727292	12.63920395	4.468634541	121.4476831	45.43182565	80.761111	16.3917038	82.54687324	-2.325444936	0.0005049	0.004885	Fbln7	2	128862981	128897034	+	3228	protein_coding	fibulin 7 [Source:MGI Symbol;Acc:MGI:1917620]	-
ENSMUSG0000003721	1609.376259	2204.487823	1359.582059	851.8564436	1190.116302	815.3953135	1724.482047	952.4560199	0.85668063	0.000506	0.0048932	Spry1	3	37639947	37644598	+	2906	protein_coding	sprouty RTK signaling antagonist 1 [Source:MGI Symbol;Acc:MGI:1345139]	-
ENSMUSG0000004072	46.09670482	63.19601977	46.92066268	243.7566972	107.6536738	96.32951794	52.07112909	149.2466297	-1.520962176	0.0005067	0.0048957	Cep126	9	8076461	8134294	-	4616	protein_coding	centrosomal protein 126 [Source:MGI Symbol;Acc:MGI:2680221]	-
ENSMUSG0000000676	0	0	2.23431727	13.78129737	18.76531929	22.37958498	0.744772423	18.30873388	-4.678286301	0.0005068	0.0048957	Tph2	10	115078641	115185022	-	2879	protein_coding	tryptophan hydroxylase 2 [Source:MGI Symbol;Acc:MGI:2651811]	-
ENSMUSG0000006135	12033.24416	4650.173788	7802.235908	4175.733103	3863.680477	3823.989955	8161.884619	3954.467845	1.045430275	0.0005141	0.0049636	Cxcl12	6	117168535	117181367	+	8176	protein_coding	chemokine (C-X-C motif) ligand 12 [Source:MGI Symbol;Acc:MGI:103556]	-
ENSMUSG0000011544	2.004204557	2.106533992	0	9.474641942	22.71591283	30.16378845	1.370246183	20.78478107	-3.897002902	0.0005157	0.0049767	Gm34759	15	12068248	12085197	-	2787	lincRNA	predicted gene, 34759 [Source:MGI Symbol;Acc:MGI:5593918]	-
ENSMUSG0000002786	429.9018775	487.6626192	434.5747091	610.6837397	718.020375	803.7190083	450.7130686	710.8077077	-0.656827817	0.0005163	0.00498	Gdap2	3	100162381	100206981	+	4794	protein_coding	ganglioside-induced differentiation-associated-protein 2 [Source:MGI Symbol;Acc:MGI:1338001]	-
ENSMUSG0000002529	7782.326296	7857.371791	11007.36403	6136.983985	4981.698448	5520.946311	8882.35404	5546.542915	0.679268515	0.0005182	0.0049956	Rps24	14	24487125	24496959	+	6256	protein_coding	ribosomal protein S24 [Source:MGI Symbol;Acc:MGI:98147]	-
ENSMUSG0000004622	526.1036963	633.0134647	647.9520084	1131.789046	959.0065806	822.2064915	602.3563898	971.0007062	-0.689498987	0.0005185	0.0049957	Scand1	2	156311847	156312704	-	759	protein_coding	SCAN domain-containing 1 [Source:MGI Symbol;Acc:MGI:1343132]	-
ENSMUSG0000003731	460.9670482	184.3217243	337.3819078	138.6743048	151.1102027	143.0347388	327.5568934	144.2730821	1.183478135	0.0005203	0.0050101	Jade3	X	20425688	20519939	+	5209	protein_coding	jade family PHD finger 3 [Source:MGI Symbol;Acc:MGI:2148019]	-
ENSMUSG0000010743	9.018920508	13.69247095	93.84132536	1.722662171	5.925890302	0.973025434	38.85090561	2.873859302	3.764815316	0.0005251	0.0050543	Gm45060	6	58964874	58972234	+	593	antisense	predicted gene 45060 [Source:MGI Symbol;Acc:MGI:5753636]	-
ENSMUSG0000003348	2086.376944	2439.366363	2013.119861	1299.748608	1610.854514	1452.726973	2179.621056	1454.443365	0.583856231	0.0005254	0.0050543	Fndc3a	14	72537946	72710003	-	9944	protein_coding	fibronectin type III domain containing 3A [Source:MGI Symbol;Acc:MGI:1196463]	-
ENSMUSG0000003085	1356.846485	1358.714425	1400.916929	873.3897208	941.2289097	1043.083265	1372.15928	952.5672985	0.526790023	0.0005259	0.0050565	Ate1	7	130391493	130520369	-	5579	protein_coding	arginyltransferase 1 [Source:MGI Symbol;Acc:MGI:1333870]	-
ENSMUSG0000003610	449.9439231	368.6434486	355.256446	701.1235037	650.8602849	549.7593701	391.2812726	633.9143862	-0.695826497	0.0005281	0.005075	Prr5	15	84669620	84703673	+	1917	protein_coding	proline rich 5 (renal) [Source:MGI Symbol;Acc:MGI:1924714]	-
ENSMUSG0000005384	113.2375575	124.2855055	194.3856025	76.65846662	19.75296767	27.24471215	143.9695552	41.21871548	1.797806139	0.0005285	0.0050768	Lipg	18	74939322	74961263	-	3787	protein_coding	lipase, endothelial [Source:MGI Symbol;Acc:MGI:1341803]	-
ENSMUSG0000002899	1770.714726	243.3046761	4426.182513	197.2448186	210.3691057	187.7939087	2146.733972	198.4692777	3.435128011	0.0005324	0.0051111	Rbp7	4	149449687	149454978	-	718	protein_coding	retinol binding protein 7, cellular [Source:MGI Symbol;Acc:MGI:1890409]	-
ENSMUSG0000009704	357.7505135	354.9509777	369.7795083	198.9674808	252.8379862	160.5491966	360.8269998	204.1182212	0.822192145	0.0005336	0.0051196	1600020E01Ri	6	86526247	86582466	+	4328	antisense	RIKEN cDNA 1600020E01 gene [Source:MGI Symbol;Acc:MGI:1919262]	-
ENSMUSG0000008502	62.13034127	29.49147589	124.0046085	19.81061497	12.83942899	18.48748324	71.87547522	17.0458424	2.072375938	0.0005382	0.0051615	C230035I16Ri	13	23427975	23431022	-	917	lincRNA	RIKEN cDNA C230035I16 gene [Source:MGI Symbol;Acc:MGI:2444850]	-
ENSMUSG0000001430	283.5949448	175.8955883	242.4234238	522.827969	440.4911792	357.1003342	233.971319	440.1398274	-0.911755067	0.0005405	0.0051791	Glis2	16	4594713	4624924	+	4373	protein_coding	GLIS family zinc finger 2 [Source:MGI Symbol;Acc:MGI:1932535]	zf-C2
ENSMUSG0000003149	5902.382421	3982.402512	6228.159391	2385.887107	3170.351312	3596.302003	5370.981442	3050.846807	0.816062004	0.0005406	0.0051791	Eif4ebp1	8	27260329	27276674	+	2196	protein_coding	eukaryotic translation initiation factor 4E binding protein 1 [Source:MGI Symbol;Acc:MGI:103267]	-
ENSMUSG0000007857	94.19761419	105.3266996	41.3348695	223.0847512	158.0237414	219.903748	80.28639443	200.3374135	-1.317400711	0.0005444	0.005213	1110065P20Ri	4	124849080	124851196	-	2069	protein_coding	RIKEN cDNA 1110065P20 gene [Source:MGI Symbol;Acc:MGI:1916170]	-
ENSMUSG0000003780	6527.694243	6472.325691	6618.047755	5174.015831	4150.098508	4419.48152	6539.355896	4581.19862	0.513314774	0.0005447	0.005213	Rpl10a	17	28328471	28331033	+	2431	protein_coding	ribosomal protein L10A [Source:MGI Symbol;Acc:MGI:1343877]	-
ENSMUSG0000004787	279.5865357	258.050414	272.586707	158.4849197	175.8014123	158.6031457	270.0745523	164.2964926	0.717585652	0.0005475	0.0052378	Gpr157	4	150087365	150105927	+	4900	protein_coding	G protein-coupled receptor 157 [Source:MGI Symbol;Acc:MGI:2442046]	-
ENSMUSG0000002109	12.02522734	12.63920395	1.117158635	45.65054754	52.34536434	39.89404278	8.593863311	45.96331822	-2.408585378	0.0005486	0.0052441	Lrrc9	12	72441866	72530750	+	7666	protein_coding	leucine rich repeat containing 9 [Source:MGI Symbol;Acc:MGI:1925507]	-
ENSMUSG0000002125	176.370001	191.6945933	195.5027612	417.7455765	280.492141	307.4760371	187.8557852	335.2379182	-0.836946742	0.0005488	0.0052441	Erg28	12	85815448	85824550	-	4235	protein_coding	ergosterol biosynthesis 28 [Source:MGI Symbol;Acc:MGI:1915571]	-
ENSMUSG0000004896	2325.879389	3735.938035	2180.693656	987.9467552	1745.174694	1470.24143	2747.503693	1401.12096	0.971775133	0.0005521	0.0052735	Prex2	1	10993465	11303681	+	16609	protein_coding	phosphatidylinositol-3,4,5-trisphosphate-dependent Rac exchange factor 2 [Source:MGI Symbol;Acc:MGI:1923385]	-
ENSMUSG0000002630	1022.144324	1179.659036	1702.54976	744.190058	789.1310586	740.4723551	1301.45104	757.9311572	0.779716956	0.000555	0.0052981	Ilkap	1	91373861	91398815	-	5556	protein_coding	integrin-linked kinase-associated serine/threonine phosphatase 2C [Source:MGI Symbol;Acc:MGI:1914694]	-
ENSMUSG0000007905	76.15977317	70.56888874	71.49815265	438.4175226	116.5425093	142.0617133	72.74227152	232.3405817	-1.67657932	0.0005554	0.0052995	Kcnip3	2	127456498	127522094	-	3546	protein_coding	Kv channel interacting protein 3, calsenilin [Source:MGI Symbol;Acc:MGI:1929258]	-
ENSMUSG0000004015	393.8261955	390.7620556	244.6577411	615.8517262	653.82323	557.5435736	343.0819974	609.0728433	-0.827205749	0.0005561	0.005303	Tax1bp3	11	73177083	73183162	+	2989	protein_coding	Tax1 (human T cell leukemia virus type I) binding protein 3 [Source:MGI Symbol;Acc:MGI:1923531]	-
ENSMUSG0000002747	545.1436396	594.0425858	451.3320886	850.1337815	775.3039812	829.0176696	530.1727713	818.1518108	-0.625625443	0.0005565	0.0053045	Kif3b	2	153291413	153333390	+	5635	protein_coding	kinesin family member 3B [Source:MGI Symbol;Acc:MGI:107688]	-
ENSMUSG0000000642	1982.158307	2805.903278	3199.542331	1269.60202	1735.29821	1617.168271	2662.534639	1540.6895	0.789276742	0.0005569	0.0053054	C330007P06Ri	X	36823737	36874111	-	7424	protein_coding	RIKEN cDNA C330007P06 gene [Source:MGI Symbol;Acc:MGI:1924894]	-
ENSMUSG0000003030	861.8079596	1167.019832	786.4796792	569.3398476	609.3790528	520.5686071	938.4358235	566.4291691	0.728474931	0.0005585	0.0053179	Tmtc1	6	148232430	148444389	-	8578	protein_coding	transmembrane and tetratricopeptide repeat containing 1 [Source:MGI Symbol;Acc:MGI:3039590]	-
ENSMUSG0000005816	30.06306836	38.97087886	22.3431727	61.15450708	104.6907287	85.62623817	30.45903997	83.82382464	-1.457006334	0.0005597	0.0053267	Gm5431	11	48887422	48902214	-	3862	protein_coding	predicted gene 5431 [Source:MGI Symbol;Acc:MGI:3645205]	-
ENSMUSG0000003029	1084.274665	1022.722253	999.8569785	1412.58298	1436.04075	1491.64799	1035.617966	1446.75724	-0.482120401	0.0005642	0.0053671	Sec13	6	113728062	113740743	-	3184	protein_coding	SEC13 homolog, nuclear pore and COPII coat complex component [Source:MGI Symbol;Acc:MGI:99832]	-
ENSMUSG0000010821	154.3237509	119.0191706	243.5405825	61.15450708	91.85129969	60.32757689	172.2945013	71.11112789	1.27711954	0.0005662	0.0053828	Olfr1372-ps1	11	52154722	52168159	-	9812	transcribed_unproc	olfactory receptor 1372, pseudogene 1 [Source:MGI Symbol;Acc:MGI:3031206]	-
ENSMUSG0000001514	1887.960693	1737.890544	1665.683525	1342.815162	1158.511554	1160.819342	1763.844921	1220.715353	0.530837742	0.0005672	0.0053899	Actn1	12	80167547	80260371	-	5533	protein_coding	actinin, alpha 1 [Source:MGI Symbol;Acc:MGI:2137706]	-
ENSMUSG0000002947	407.8556274	595.0958528	417.8173296	752.8033688	841.476423	767.7170673	473.5896033	787.3322863	-0.733211673	0.0005694	0.0054085	Camkk2	5	122731170	122779409	-	10921	protein_coding	calcium/calmodulin-dependent protein kinase kinase 2, beta [Source:MGI Symbol;Acc:MGI:2444812]	-
ENSMUSG0000005039	248.5213651	260.156948	174.2767471	446.1695023	378.269331	376.5608429	227.6516867	400.3332254	-0.813791585	0.0005705	0.0054163	C77080	4	129219578	129261404	-	5770	protein_coding	expressed sequence C77080 [Source:MGI Symbol;Acc:MGI:2140651]	-
ENSMUSG0000007881	36.07568203	18.95880593	8.937269082	0	2.962945151	0	21.32391901	0.987648384	4.47766612	0.0005717	0.0054244	Nlrp12	7	3218784	3249740	-	7545	protein_coding	NLR family, pyrin domain containing 12 [Source:MGI Symbol;Acc:MGI:2676630]	-
ENSMUSG0000002195	167.3510805	121.1257046	144.1134639	250.6473459	252.8379862	252.0135873	144.1967497	251.8329732	-0.803721938	0.0005723	0.0054278	Eef1akmt1	14	57549597	57571612	-	947	protein_coding	EEF1A alpha lysine methyltransferase 1 [Source:MGI Symbol;Acc:MGI:1915293]	-
ENSMUSG0000010275	38.07988659	34.75781087	20.10885543	69.76781793	82.96246423	94.38346708	30.9821843	82.37124975	-1.405774653	0.0005751	0.0054513	Naaladl2	3	23798102	25144261	-	10411	protein_coding	N-acetylated alpha-linked acidic dipeptidase-like 2 [Source:MGI Symbol;Acc:MGI:2685867]	-
ENSMUSG0000007867	1045.192677	1096.450943	1135.033173	791.5632677	774.3163329	754.0947112	1092.225598	773.3247706	0.497878776	0.0005827	0.0055205	Casc3	11	98804905	98833814	+	4303	protein_coding	cancer susceptibility candidate 3 [Source:MGI Symbol;Acc:MGI:2179723]	-
ENSMUSG0000002418	1625.409896	1746.31668	2039.931668	1180.023587	1320.485889	1029.460909	1803.886081	1176.656795	0.616284854	0.0005838	0.0055282	Luc7l	17	26252910	26285504	+	7509	protein_coding	Luc7-like [Source:MGI Symbol;Acc:MGI:1914228]	-
ENSMUSG0000003803	400.8409114	450.7982743	406.6457432	729.5474295	605.4284592	604.2487944	419.4283097	646.4082277	-0.6245282	0.0005851	0.005538	Igsf8	1	172261641	172319841	+	5217	protein_coding	immunoglobulin superfamily, member 8 [Source:MGI Symbol;Acc:MGI:2154090]	-
ENSMUSG0000008511	5.010511393	2.106533992	0	18.94928388	37.53063858	18.48748324	2.372348462	24.98913524	-3.36888386	0.0005868	0.0055506	B130011K05R	11	63413169	63417888	+	3270	lincRNA	RIKEN cDNA B130011K05 gene [Source:MGI Symbol;Acc:MGI:2443995]	-
ENSMUSG0000004988	333.7000588	477.1299492	440.1605023	284.2392582	193.5790832	198.4971885	416.9968368	225.43851	0.885593447	0.0005884	0.0055636	2810025M15R	1	157412352	157420236	+	1410	transcribed_process	RIKEN cDNA 2810025M15 gene [Source:MGI Symbol;Acc:MGI:1917203]	-
ENSMUSG0000003202	52.10931849	74.78195672	68.14667675	113.6957033	161.9743349	138.1696116	65.01265065	137.9465499	-1.08544831	0.0005909	0.0055836	Ubash3b	9	41011098	41161697	-	7326	protein_coding	ubiquitin associated and SH3 domain containing, B [Source:MGI Symbol;Acc:MGI:1920078]	-
ENSMUSG0000003873	23.04835241	28.43820889	25.69464861	155.0395954	57.28360626	55.46244973	25.72706997	89.2618838	-1.797813329	0.0005919	0.0055909	Mboat1	13	30136489	30246717	+	3659	protein_coding	membrane bound O-acyltransferase domain containing 1 [Source:MGI Symbol;Acc:MGI:2387184]	-
ENSMUSG0000002325	79.16608001	135.8714425	71.49815265	227.3914066	201.4802703	186.8208833	95.51189172	205.2308534	-1.103441309	0.0005931	0.0055994	Slc26a6	9	108853283	108913049	+	5411	protein_coding	solute carrier family 26, member 6 [Source:MGI Symbol;Acc:MGI:2159728]	-
ENSMUSG0000002236	1154.421825	1358.714425	1138.384649	1551.257285	2043.444506	2288.55582	1217.173633	1961.08587	-0.687893193	0.0005975	0.0056366	Zhx1	15	58047003	58076541	-	6031	protein_coding	zinc fingers and homeoboxes 1 [Source:MGI Symbol;Acc:MGI:109271]	Hom
ENSMUSG0000002869	6433.496629	6427.03521	7484.962856	9169.730737	9289.820698	10136.00594	6781.831565	9531.852459	-0.491112893	0.0005977	0.0056366	Prdx1	4	116685544	116700822	+	3043	protein_coding	peroxiredoxin 1 [Source:MGI Symbol;Acc:MGI:99523]	-
ENSMUSG0000009095	182.3826147	201.1739963	328.4446388	125.7543385	116.5425093	94.38346708	237.3337499	112.2267716	1.078675279	0.0005989	0.0056452	Gm17251	17	33759992	33761870	+	925	processed_transcrip	predicted gene, 17251 [Source:MGI Symbol;Acc:MGI:4936885]	-
ENSMUSG0000001126	633.3286401	313.8735648	184.3311748	185.1861834	115.5548609	89.51833991	377.1777932	130.0864614	1.535140887	0.0005998	0.0056514	Exoc3l2	7	19463331	19496762	+	4016	protein_coding	exocyst complex component 3-like 2 [Source:MGI Symbol;Acc:MGI:1921713]	-
ENSMUSG0000002062	40.08409114	43.18394684	11.17158635	124.8930074	91.85129969	92.43741621	31.47987478	103.0605744	-1.707316237	0.0006006	0.0056561	Map2k6	11	110399122	110525522	+	3444	protein_coding	mitogen-activated protein kinase kinase 6 [Source:MGI Symbol;Acc:MGI:1346870]	-
ENSMUSG0000003132	7570.882715	6541.841313	7049.270988	9863.102261	11634.49796	9269.040282	7053.998339	10255.54683	-0.539860565	0.0006062	0.0057055	Flna	X	74223461	74249820	-	9400	protein_coding	filamin, alpha [Source:MGI Symbol;Acc:MGI:95556]	-
ENSMUSG0000003424	4332.08815	4703.890405	7191.150135	2850.144562	3327.387405	3321.908831	5409.042897	3166.480266	0.772466234	0.0006065	0.0057055	Golgb1	16	36875140	36933085	+	11862	protein_coding	golgi autoantigen, golgin subfamily b, macrogolgin 1 [Source:MGI Symbol;Acc:MGI:1099447]	-
ENSMUSG0000006022	151.3174441	141.1377775	99.42711853	406.5482724	222.2208863	228.6609769	130.6274467	285.8100452	-1.129654599	0.0006082	0.0057183	Casc4	2	121866970	121936220	+	5858	protein_coding	cancer susceptibility candidate 4 [Source:MGI Symbol;Acc:MGI:2443129]	-
ENSMUSG0000005148	1294.716144	1048.000661	988.6853922	789.8406055	693.3291654	653.8730915	1110.467399	712.3476208	0.640447451	0.0006094	0.0057271	Cbr1	16	93605853	93610505	+	2554	protein_coding	carbonyl reductase 1 [Source:MGI Symbol;Acc:MGI:88284]	-
ENSMUSG0000004986	180.3784102	136.9247095	167.5737953	1193.804885	212.3444025	296.7727573	161.6256383	567.6406815	-1.812832783	0.0006097	0.0057274	Arl4c	1	88673125	88702221	-	5492	protein_coding	ADP-ribosylation factor-like 4C [Source:MGI Symbol;Acc:MGI:2445172]	-
ENSMUSG0000001818	418.8787525	600.3621878	513.8929722	298.0205556	313.0845376	320.1253677	511.0446375	310.4101537	0.719205219	0.0006114	0.0057401	Uchl5	1	143777272	143807466	+	2921	protein_coding	ubiquitin carboxyl-terminal esterase L5 [Source:MGI Symbol;Acc:MGI:1914848]	-
ENSMUSG0000002977	962.0181875	1394.525503	1433.314529	738.1607404	549.1325014	845.559102	1263.286073	710.9507812	0.829049453	0.0006117	0.0057401	Adcyap1r1	6	55451978	55501451	+	6601	protein_coding	adenylate cyclase activating polypeptide 1 receptor 1 [Source:MGI Symbol;Acc:MGI:108449]	-
ENSMUSG0000004280	294.6180699	487.6626192	388.7712051	190.3541699	228.1467766	221.8497989	390.3506314	213.4502485	0.871162621	0.000612	0.005742	Hecw2	1	53806876	54195168	-	12947	protein_coding	HECT, C2 and WW domain containing E3 ubiquitin protein ligase 2 [Source:MGI Symbol;Acc:MGI:2685817]	-
ENSMUSG0000004660	65.13664811	67.40908775	68.14667675	22.39460823	32.59239666	22.37958498	66.89747087	25.78886329	1.377781676	0.0006138	0.0057517	Hrk	5	118164648	118189478	+	5677	protein_coding	harakiri, BCL2 interacting protein (contains only BH3 domain) [Source:MGI Symbol;Acc:MGI:1201608]	-
ENSMUSG0000002307	969.0329034	1204.937444	1093.698304	1641.697049	1519.990863	1597.707762	1089.222884	1586.465225	-0.5427362	0.0006139	0.0057517	Akirin1	4	123734559	123750345	-	3450	protein_coding	akirin 1 [Source:MGI Symbol;Acc:MGI:1915300]	-
ENSMUSG0000005492	1680.525521	1286.039002	1323.832983	1874.256442	2650.848262	2433.53661	1430.132502	2319.547105	-0.697376917	0.0006167	0.0057749	Klhl5	5	65107539	65168188	+	7057	protein_coding	kelch-like 5 [Source:MGI Symbol;Acc:MGI:1919028]	-
ENSMUSG0000002078	230.4835241	170.6292534	111.7158635	75.79713553	85.92540939	55.46244973	170.9428803	72.39499821	1.24060595	0.0006223	0.0058244	Camkk1	11	73019008	73042073	+	3728	protein_coding	calcium/calmodulin-dependent protein kinase kinase 1, alpha [Source:MGI Symbol;Acc:MGI:1891766]	-
ENSMUSG0000007457	0	2.106533992	0	42.20522319	4.938241919	20.43353411	0.702177997	22.52566641	-5.003853432	0.0006243	0.005841	Kcng1	2	168260117	168281736	-	4508	protein_coding	potassium voltage-gated channel, subfamily G, member 1 [Source:MGI Symbol;Acc:MGI:3616086]	-
ENSMUSG0000010922	372.7820476	385.4957206	290.4612452	241.172704	151.1102027	160.5491966	349.5796711	184.2773678	0.922035432	0.0006253	0.0058471	Gm10297	7	60753363	60754013	-	651	processed_pseudog	predicted pseudogene 10297 [Source:MGI Symbol;Acc:MGI:3642512]	-
ENSMUSG0000010986	460.9670482	410.7741285	599.9141871	763.1393418	836.538181	782.3124488	490.5517879	793.9966572	-0.695181513	0.0006294	0.0058824	Hspa14	2	3488850	3512814	-	4057	protein_coding	heat shock protein 14 [Source:MGI Symbol;Acc:MGI:1354164]	-
ENSMUSG0000008701	0	0	0	9.474641942	6.913538686	15.56840694	0	10.65219586	-5.776208669	0.0006318	0.0059022	1700041M05R	4	125228597	125242052	+	306	lincRNA	RIKEN cDNA 1700041M05 gene [Source:MGI Symbol;Acc:MGI:1920510]	-
ENSMUSG0000002875	13.02732962	21.06533992	2.23431727	71.4904801	56.29595787	46.70522082	12.1089956	58.16388627	-2.258584235	0.0006364	0.0059417	Pla2g2e	4	138877942	138882817	+	917	protein_coding	phospholipase A2, group IIE [Source:MGI Symbol;Acc:MGI:1349660]	-
ENSMUSG0000003221	10.02102279	5.26633498	12.28874499	49.09587188	19.75296767	68.11178036	9.192034251	45.65353997	-2.31517401	0.0006371	0.0059456	Ccnb2	9	70407692	70421547	-	1531	protein_coding	cyclin B2 [Source:MGI Symbol;Acc:MGI:88311]	-
ENSMUSG0000004419	2838.955755	2265.577309	1941.621708	1120.591742	1654.311043	1399.210574	2348.718257	1391.37112	0.755732068	0.0006376	0.0059473	Gpr146	5	139377697	139396415	+	6786	protein_coding	G protein-coupled receptor 146 [Source:MGI Symbol;Acc:MGI:1933113]	-
ENSMUSG0000011604	0	1.053266996	1.117158635	16.36529063	13.82707737	18.48748324	0.72347521	16.22661708	-4.517482229	0.0006415	0.0059783	Gm20369	15	84182709	84183015	-	307	processed_pseudog	predicted gene, 20369 [Source:MGI Symbol;Acc:MGI:5012554]	-
ENSMUSG0000010050	11.02312506	11.58593696	31.28044179	0.861331086	1.975296767	0	17.96316794	0.945542618	4.253152512	0.0006416	0.0059783	Gm28286	1	154027363	154034301	-	586	lincRNA	predicted gene 28286 [Source:MGI Symbol;Acc:MGI:5578992]	-
ENSMUSG0000005860	242.5087514	269.636351	202.205713	153.3169332	71.11068363	101.1946451	238.1169385	108.540754	1.130918371	0.0006422	0.0059812	Rpl28-ps1	1	128038569	128038982	+	414	processed_pseudog	ribosomal protein L28, pseudogene 1 [Source:MGI Symbol;Acc:MGI:3705349]	-
ENSMUSG0000002718	22872.98451	31703.33658	16261.36109	9913.920795	13385.59854	14608.03084	23612.56073	12635.85006	0.902057455	0.0006425	0.0059813	Cat	2	103453849	103485160	-	2613	protein_coding	catalase [Source:MGI Symbol;Acc:MGI:88271]	-
ENSMUSG0000003137	202.4246603	205.3870642	214.494458	358.3137316	300.2451087	340.5589018	207.4353942	333.0392474	-0.683809033	0.0006434	0.0059866	Haus7	X	73437315	73459044	-	1438	protein_coding	HAUS augmin-like complex, subunit 7 [Source:MGI Symbol;Acc:MGI:1920988]	-
ENSMUSG0000006012	23879.0952	29005.91981	27334.63749	20551.3597	16811.75079	17850.15158	26739.88416	18404.42069	0.538913061	0.0006525	0.0060681	Tpt1	14	75845093	75848525	+	2380	protein_coding	tumor protein, translationally-controlled 1 [Source:MGI Symbol;Acc:MGI:104890]	-
ENSMUSG0000000245	388.8156841	343.3650407	377.5996187	556.4198813	544.1942594	552.6784464	369.9267812	551.097529	-0.575042232	0.0006536	0.0060753	Rgs19	2	181688419	181693977	-	2230	protein_coding	regulator of G-protein signaling 19 [Source:MGI Symbol;Acc:MGI:1915153]	-
ENSMUSG0000003524	14.0294319	4.213067984	5.585793176	33.59191234	40.49358373	37.94799192	7.942764354	37.344496	-2.222790204	0.0006544	0.0060787	Pcyt1b	X	93654863	93749951	+	5357	protein_coding	phosphate cytidylyltransferase 1, choline, beta isoform [Source:MGI Symbol;Acc:MGI:2147987]	-
ENSMUSG0000003041	64.13454583	58.98295178	279.2896588	44.78921645	20.74061606	24.32563584	134.1357188	29.95182278	2.158540577	0.0006546	0.0060787	Pglyrp1	7	18871331	18890459	+	1837	protein_coding	peptidoglycan recognition protein 1 [Source:MGI Symbol;Acc:MGI:1345092]	-
ENSMUSG0000001700	1153.419723	1367.140561	2282.355092	888.0323492	917.5253485	695.7131852	1600.971792	833.756961	0.940960255	0.0006581	0.0061078	Sdc4	2	164424247	164443887	-	2547	protein_coding	syndecan 4 [Source:MGI Symbol;Acc:MGI:1349164]	-
ENSMUSG0000002944	75.1576709	47.39701482	77.08394583	131.7836561	145.1843124	141.0886879	66.54621052	139.3522188	-1.066275167	0.0006593	0.0061165	Rhof	5	123103044	123132692	-	4602	protein_coding	ras homolog family member F (in filopodia) [Source:MGI Symbol;Acc:MGI:1345629]	-
ENSMUSG0000000781	1720.609612	1734.730743	2368.376307	906.9816331	1178.264522	1355.424429	1941.238887	1146.890195	0.75938959	0.0006601	0.0061203	Zfp655	5	145231715	145247302	+	5598	protein_coding	zinc finger protein 655 [Source:MGI Symbol;Acc:MGI:1919861]	zf-C2
ENSMUSG0000003432	536.1247191	367.5901816	282.6411347	629.6330236	776.2916296	837.7748985	395.4520118	747.8998506	-0.918282609	0.0006615	0.0061308	Slc26a2	18	61192919	61211612	-	12370	protein_coding	solute carrier family 26 (sulfate transporter), member 2 [Source:MGI Symbol;Acc:MGI:892977]	-
ENSMUSG0000002279	775.6271636	423.4133324	515.0101308	1030.151978	960.9818774	1094.653613	571.350209	1028.595823	-0.847782358	0.0006622	0.0061335	Tfrc	16	32608920	32632794	+	6185	protein_coding	transferrin receptor [Source:MGI Symbol;Acc:MGI:98822]	-
ENSMUSG0000001535	168.3531928	126.3920395	145.2306226	219.6394268	271.6033055	315.2602405	146.658615	268.8343243	-0.872893885	0.0006629	0.0061371	Cd48	1	171682009	171705258	+	1189	protein_coding	CD48 antigen [Source:MGI Symbol;Acc:MGI:88339]	-
ENSMUSG0000002865	348.731593	268.583084	462.503675	222.2234201	177.7767091	157.6301203	359.9394506	185.8767498	0.951914205	0.0006642	0.0061465	Ppie	4	123127115	123139951	-	1719	protein_coding	peptidylprolyl isomerase E (cyclophilin E) [Source:MGI Symbol;Acc:MGI:1917118]	-
ENSMUSG0000004155	1216.552166	882.6377427	776.4252515	1857.029821	1508.139082	1480.94471	958.5383868	1615.371204	-0.752742458	0.0006676	0.0061744	Fmod	1	134037254	134048277	+	3306	protein_coding	fibromodulin [Source:MGI Symbol;Acc:MGI:1328364]	-
ENSMUSG0000010901	129.2711939	73.72868973	99.42711853	50.81853405	35.55534181	35.02891562	100.8090007	40.46759716	1.314328136	0.000669	0.0061847	Gm31024	7	28167275	28169627	-	2353	TEC	predicted gene, 31024 [Source:MGI Symbol;Acc:MGI:5590183]	-
ENSMUSG0000002630	436.9165935	566.6576439	441.2776609	298.0205556	315.0598344	284.1234267	481.6172994	299.0679389	0.687495862	0.0006716	0.0062057	Scly	1	91298338	91320175	+	4549	protein_coding	selenocysteine lyase [Source:MGI Symbol;Acc:MGI:1355310]	-
ENSMUSG0000002397	2132.473649	2606.835815	2502.435343	1521.110697	1759.98942	1690.145178	2413.914936	1657.081765	0.542798222	0.0006724	0.0062103	Ubr2	17	46928295	47010556	-	9814	protein_coding	ubiquitin protein ligase E3 component n-recognin 2 [Source:MGI Symbol;Acc:MGI:1861099]	-
ENSMUSG0000003263	2518.283026	2879.631967	2819.708395	1900.096375	2088.876332	1768.960239	2739.207796	1919.310982	0.513142293	0.0006764	0.0062438	Atxn2l	7	126491708	126503437	-	4782	protein_coding	ataxin 2-like [Source:MGI Symbol;Acc:MGI:2446242]	-
ENSMUSG0000006314	473.9943778	377.0695846	407.7629019	659.7796116	652.8355817	610.086947	419.6089547	640.9007134	-0.61074814	0.0006789	0.0062637	Clip2	5	134489383	134552434	-	5187	protein_coding	CAP-GLY domain containing linker protein 2 [Source:MGI Symbol;Acc:MGI:1313136]	-
ENSMUSG0000001837	408.8577297	368.6434486	750.7306029	225.6687444	283.4550861	231.5800532	509.4105937	246.9012946	1.044660479	0.0006801	0.0062715	Cep95	11	106789252	106819930	+	4199	protein_coding	centrosomal protein 95 [Source:MGI Symbol;Acc:MGI:2443502]	-
ENSMUSG0000003025	358.7526157	161.1498504	202.205713	123.1703452	103.7030803	78.81506014	240.7027264	101.8961619	1.23979626	0.0006805	0.0062721	Srgap3	6	112717971	112947266	-	9852	protein_coding	SLIT-ROBO Rho GTPase activating protein 3 [Source:MGI Symbol;Acc:MGI:2152938]	-
ENSMUSG0000000038	17.03573874	37.91761186	16.75737953	66.32249359	81.97481585	69.0848058	23.90357671	72.46070508	-1.598994731	0.0006809	0.0062733	Mx1	16	97447035	97462907	-	3785	protein_coding	MX dynamin-like GTPase 1 [Source:MGI Symbol;Acc:MGI:97243]	-
ENSMUSG0000009533	17.03573874	11.58593696	13.40590362	44.78921645	42.4688805	47.67824626	14.00919311	44.97878107	-1.680543335	0.0006818	0.0062777	Gm9821	2	91945703	91947655	+	1953	protein_coding	predicted gene 9821 [Source:MGI Symbol;Acc:MGI:3704208]	-
ENSMUSG0000004631	322.6769337	393.9218565	270.3523897	531.4412798	505.6759725	605.2218198	328.9837267	547.4463574	-0.734204865	0.0006834	0.0062898	Ccbe1	18	66045302	66302739	-	9511	protein_coding	collagen and calcium binding EGF domains 1 [Source:MGI Symbol;Acc:MGI:2445053]	-
ENSMUSG0000000685	340.7147747	367.5901816	571.9852212	225.6687444	249.8750411	214.0655954	426.7633925	229.8697936	0.892018047	0.0006843	0.006295	Tmco6	18	36735068	36742391	+	1817	protein_coding	transmembrane and coiled-coil domains 6 [Source:MGI Symbol;Acc:MGI:1919233]	-
ENSMUSG0000003148	1157.428132	966.8991024	809.9400105	1497.854758	1534.805588	1492.621015	978.0890816	1508.427121	-0.624587545	0.0006898	0.0063423	Erlin2	8	27023261	27040328	+	5829	protein_coding	ER lipid raft associated 2 [Source:MGI Symbol;Acc:MGI:2387215]	-
ENSMUSG0000006648	116.2438643	69.51562174	89.37269082	45.65054754	15.80237414	28.21773758	91.71072563	29.89021975	1.611809339	0.0006924	0.0063629	Gm5786	12	59081019	59081900	+	882	processed_pseudog	predicted pseudogene 5786 [Source:MGI Symbol;Acc:MGI:3645003]	-
ENSMUSG0000004072	44.09250026	61.08948577	8.937269082	161.068913	95.80189322	179.0366798	38.0397517	145.3024953	-1.930802076	0.0006928	0.006364	Kcna2	3	107101146	107115005	+	11906	protein_coding	potassium voltage-gated channel, shaker-related subfamily, member 2 [Source:MGI Symbol;Acc:MGI:96659]	-
ENSMUSG0000004168	236.4961378	165.3629184	217.8459339	675.2835711	246.9120959	519.5955816	206.56833	480.5970829	-1.218684636	0.0006947	0.0063781	Amot	X	145446425	145505181	-	8402	protein_coding	angiomotin [Source:MGI Symbol;Acc:MGI:108440]	-
ENSMUSG0000000761	117.2459666	200.1207293	146.3477812	248.9246837	386.170518	294.8267064	154.5714924	309.9739694	-1.003656094	0.0006951	0.0063787	Homer1	13	93299635	93405129	+	11976	protein_coding	homer scaffolding protein 1 [Source:MGI Symbol;Acc:MGI:1347345]	-
ENSMUSG0000003799	10.02102279	13.69247095	24.57748997	69.76781793	41.48123212	56.43547516	16.09699457	55.89484174	-1.804382643	0.0006959	0.0063828	Igsf9	1	172481788	172498878	+	6612	protein_coding	immunoglobulin superfamily, member 9 [Source:MGI Symbol;Acc:MGI:2135283]	-
ENSMUSG0000007164	452.9502299	397.0816575	603.265663	314.3858462	259.7515249	261.7438417	484.4325168	278.627071	0.79682282	0.0006965	0.0063849	Tut1	19	8953850	8966207	+	2747	protein_coding	terminal uridylyl transferase 1, U6 snRNA-specific [Source:MGI Symbol;Acc:MGI:1917294]	-
ENSMUSG0000006338	47.0988071	34.75781087	35.74907633	72.35181119	100.7401351	107.0327977	39.2018981	93.37491468	-1.24874076	0.0006985	0.0064002	Zfp947	17	22144359	22166190	-	2872	protein_coding	zinc finger protein 947 [Source:MGI Symbol;Acc:MGI:3646759]	zf-C2
ENSMUSG0000000338	3872.123205	4252.038863	3387.224982	2782.960738	2520.478675	2701.118604	3837.129017	2668.186006	0.524167214	0.0007023	0.0064319	Rabac1	7	24969752	24972754	-	1523	protein_coding	Rab acceptor 1 (prenylated) [Source:MGI Symbol;Acc:MGI:1201692]	-
ENSMUSG0000004185	1246.615235	1198.617842	1059.066386	1460.817521	2010.852109	2083.247454	1168.099821	1851.639028	-0.664266061	0.0007034	0.0064359	Tcf20	15	82808436	82987872	-	8245	protein_coding	transcription factor 20 [Source:MGI Symbol;Acc:MGI:108399]	-
ENSMUSG0000003220	162.3405691	411.8273955	174.2767471	118.8636898	75.06127716	91.46439078	249.4815706	95.12978592	1.38972154	0.0007034	0.0064359	Rab27a	9	73044854	73097629	+	3879	protein_coding	RAB27A, member RAS oncogene family [Source:MGI Symbol;Acc:MGI:1861441]	-
ENSMUSG0000002093	413.8682411	511.8877601	304.9843074	269.5966298	180.7396542	148.8728914	410.2467695	199.7363918	1.037268788	0.0007045	0.0064397	Plcd3	11	103070304	103101658	-	3393	protein_coding	phospholipase C, delta 3 [Source:MGI Symbol;Acc:MGI:107451]	-
ENSMUSG0000003109	25.05255697	34.75781087	16.75737953	215.3327714	42.4688805	71.03085667	25.52258246	109.6108362	-2.103169421	0.0007045	0.0064397	Smarca1	X	47809368	47892974	-	6118	protein_coding	SWI/SNF related, matrix associated, actin dependent regulator of chromatin, subfamily a, member 1 [Source:MGI Symbol;Acc:MG	-
ENSMUSG0000003853	967.0286989	1124.889152	1015.497199	1538.337319	1742.211749	1361.262582	1035.805017	1547.27055	-0.578983237	0.0007069	0.0064586	Atf5	7	44812256	44816658	-	1993	protein_coding	activating transcription factor 5 [Source:MGI Symbol;Acc:MGI:2141857]	TF_b
ENSMUSG0000004462	2687.638311	2473.070907	2739.272974	2032.741362	1702.705814	1830.260841	2633.327397	1855.236006	0.505074961	0.0007074	0.0064601	Swi5	2	32278816	32288075	-	5551	protein_coding	SWI5 recombination repair homolog (yeast) [Source:MGI Symbol;Acc:MGI:1920181]	-
ENSMUSG0000006345	52.10931849	51.61008281	24.57748997	236.8660485	92.83894807	87.57228904	42.76563042	139.0924285	-1.700938903	0.0007089	0.0064699	D630045J12Ri	6	38123174	38254009	-	13450	protein_coding	RIKEN cDNA D630045J12 gene [Source:MGI Symbol;Acc:MGI:2669829]	-
ENSMUSG0000002806	7.01471595	11.58593696	1.117158635	59.43184491	18.76531929	50.59732256	6.572603848	42.93149558	-2.701312384	0.0007112	0.0064856	Iqgap3	3	88081971	88121048	+	11384	protein_coding	IQ motif containing GTPase activating protein 3 [Source:MGI Symbol;Acc:MGI:3028642]	-
ENSMUSG0000003942	2178.570354	2198.168221	1729.361567	2582.270595	3803.433926	3505.810638	2035.366714	3297.171719	-0.695711085	0.0007113	0.0064856	Tmem135	7	89139714	89404222	-	7572	protein_coding	transmembrane protein 135 [Source:MGI Symbol;Acc:MGI:1920009]	-
ENSMUSG0000007069	108.2270461	102.1668986	118.4188153	205.8581295	188.6408413	181.9557561	109.6042533	192.1515756	-0.811047262	0.000712	0.0064892	Sars2	7	28741992	28753871	+	3313	protein_coding	seryl-aminoacyl-tRNA synthetase 2 [Source:MGI Symbol;Acc:MGI:1919234]	-
ENSMUSG0000005983	288.6054562	323.3529678	319.5073697	210.1647849	180.7396542	139.142637	310.4885979	176.6823587	0.811829231	0.0007126	0.0064914	Sclt1	3	41626720	41742514	-	7569	protein_coding	sodium channel and clathrin linker 1 [Source:MGI Symbol;Acc:MGI:1914411]	-
ENSMUSG0000007876	89.1871028	88.47442767	101.6614358	31.00791908	52.34536434	35.02891562	93.10765542	39.46073301	1.241446997	0.0007141	0.0065017	Slfn1	11	83116849	83122670	+	1864	protein_coding	schlafen 1 [Source:MGI Symbol;Acc:MGI:1313259]	-
ENSMUSG0000001741	62.13034127	74.78195672	25.69464861	235.1433864	114.5672125	130.3854081	54.20231554	160.020023	-1.560833168	0.0007154	0.0065094	Plxdc1	11	97923238	97986444	-	4836	protein_coding	plexin domain containing 1 [Source:MGI Symbol;Acc:MGI:1919574]	-
ENSMUSG0000003177	5.010511393	5.26633498	25.69464861	103.3597303	47.40712242	42.81311909	11.99049833	64.52665726	-2.439645866	0.0007156	0.0065094	Ccl22	8	94745590	94751699	+	2325	protein_coding	chemokine (C-C motif) ligand 22 [Source:MGI Symbol;Acc:MGI:1306779]	-
ENSMUSG0000002922	1034.169552	1149.114293	1316.012872	794.1472609	741.7239362	817.3413644	1166.432239	784.4041872	0.572086728	0.0007165	0.0065138	Fip1l1	5	74535449	74598800	+	13122	protein_coding	FIP1 like 1 (S. cerevisiae) [Source:MGI Symbol;Acc:MGI:1914149]	-
ENSMUSG0000003700	246.5171605	312.8202978	292.6955624	151.5942711	164.9372801	184.8748324	284.0110069	167.1354612	0.765298733	0.0007168	0.0065138	Zfp113	5	138139702	138155744	-	6754	protein_coding	zinc finger protein 113 [Source:MGI Symbol;Acc:MGI:1929116]	zf-C2
ENSMUSG0000000940	345.7252861	272.796152	244.6577411	415.1615833	526.4165885	526.4067597	287.7263931	489.3283105	-0.764730397	0.0007175	0.006517	Elk1	X	20933395	20950608	-	5381	protein_coding	ELK1, member of ETS oncogene family [Source:MGI Symbol;Acc:MGI:101833]	ETS
ENSMUSG0000003288	177.3721033	271.742885	211.1429821	481.4840768	348.6398795	369.7496648	220.0859901	399.9578737	-0.862766544	0.0007208	0.0065437	Acsl3	1	78657825	78707743	+	4357	protein_coding	acyl-CoA synthetase long-chain family member 3 [Source:MGI Symbol;Acc:MGI:1921455]	-
ENSMUSG0000006237	288.6054562	258.050414	265.8837552	371.2336979	473.0835758	459.2680047	270.8465418	434.5284261	-0.681019526	0.0007228	0.0065585	Borcs7	19	46689906	46703382	+	2176	protein_coding	BLOC-1 related complex subunit 7 [Source:MGI Symbol;Acc:MGI:1913689]	-
ENSMUSG0000002601	317.6664223	366.5369146	384.3025705	165.3755684	238.0232605	216.9846717	356.1686358	206.7945002	0.785435685	0.0007235	0.0065623	Carf	1	60098247	60153953	+	14557	protein_coding	calcium response factor [Source:MGI Symbol;Acc:MGI:2182269]	-
ENSMUSG0000002667	2312.852059	1625.190975	1701.432601	2453.070932	3786.643903	3412.400196	1879.825212	3217.371677	-0.775048974	0.000726	0.0065814	Ddr2	1	169972307	170110762	-	9264	protein_coding	discoidin domain receptor family, member 2 [Source:MGI Symbol;Acc:MGI:1345277]	-
ENSMUSG0000002846	2215.648138	1972.769084	1873.475031	1417.750967	1484.435521	1388.507294	2020.630751	1430.231261	0.498735773	0.0007269	0.0065857	Npr2	4	43631935	43651244	+	4667	protein_coding	natriuretic peptide receptor 2 [Source:MGI Symbol;Acc:MGI:97372]	-
ENSMUSG0000006087	35.07357975	36.86434486	56.9750904	11.19730411	7.90118707	15.56840694	42.971005	11.55563271	1.892896506	0.0007272	0.0065857	Olfr1420	19	11886565	11898079	+	3066	protein_coding	olfactory receptor 1420 [Source:MGI Symbol;Acc:MGI:3031254]	-
ENSMUSG0000003845	320.6727292	290.7016909	296.0470383	439.2788537	515.5524563	446.6186741	302.4738195	467.1499947	-0.626447398	0.0007288	0.0065948	Abhd17c	7	84109356	84151893	-	4606	protein_coding	abhydrolase domain containing 17C [Source:MGI Symbol;Acc:MGI:1917428]	-
ENSMUSG0000008105	12.02522734	8.426135969	22.3431727	51.67986514	56.29595787	42.81311909	14.26484534	50.2629807	-1.823351771	0.0007291	0.0065948	Hist2h3c2	3	96238108	96239127	-	1020	protein_coding	histone cluster 2, H3c2 [Source:MGI Symbol;Acc:MGI:2448357]	-
ENSMUSG0000003961	420.882957	508.7279591	359.7250805	237.7273796	255.8009314	279.2582995	429.7786656	257.5955368	0.739248527	0.0007292	0.0065948	Mocos	18	24653691	24701556	+	2884	protein_coding	molybdenum cofactor sulfurase [Source:MGI Symbol;Acc:MGI:1915841]	-
ENSMUSG0000002793	1085.276768	1159.646963	1164.079298	811.3738826	793.0816521	831.9367459	1136.334343	812.1307602	0.484482847	0.00073	0.0065987	Crtc2	3	90254163	90264125	+	4094	protein_coding	CREB regulated transcription coactivator 2 [Source:MGI Symbol;Acc:MGI:1921593]	-
ENSMUSG0000007551	105.2207393	101.1136316	87.13837355	187.7701767	158.0237414	188.7669342	97.82424814	178.1869507	-0.864531188	0.0007308	0.0066025	Gm13375	2	20969054	20969997	+	854	antisense	predicted gene 13375 [Source:MGI Symbol;Acc:MGI:3649913]	-
ENSMUSG0000006544	0	0	0	9.474641942	6.913538686	14.59538151	0	10.32785404	-5.731711846	0.000733	0.0066195	Mir143	18	61649196	61649258	-	63	miRNA	microRNA 143 [Source:MGI Symbol;Acc:MGI:2676828]	-
ENSMUSG0000000205	9.018920508	2.106533992	5.585793176	37.03723668	20.74061606	36.97496648	5.570415892	31.58427307	-2.498699628	0.0007378	0.0066596	Spag5	11	78301529	78322457	+	5624	protein_coding	sperm associated antigen 5 [Source:MGI Symbol;Acc:MGI:1927470]	-
ENSMUSG0000002188	49.10301165	31.59800988	23.46033134	110.250379	93.82659646	72.97690753	34.72045096	92.35129431	-1.408053784	0.0007442	0.0067139	Rnase6	14	51123908	51132187	+	2667	protein_coding	ribonuclease, RNase A family, 6 [Source:MGI Symbol;Acc:MGI:1925666]	-
ENSMUSG0000002906	369.7757408	178.0021223	261.4151206	749.3580445	468.1453339	483.5936406	269.7309946	567.0323396	-1.071859274	0.0007446	0.0067139	Mmp23	4	155650655	155653384	-	2340	protein_coding	matrix metallopeptidase 23 [Source:MGI Symbol;Acc:MGI:1347361]	-
ENSMUSG0000002833	110.2312506	80.0482917	91.60700809	273.0419541	148.1472576	181.9557561	93.96218348	201.0483226	-1.098150291	0.0007481	0.0067429	Coro2a	4	46536937	46602202	-	6459	protein_coding	coronin, actin binding protein 2A [Source:MGI Symbol;Acc:MGI:1345966]	-
ENSMUSG0000002720	77.16187545	57.92968479	41.3348695	169.6822239	124.4436964	113.8439758	58.80880991	135.9899653	-1.20766947	0.000751	0.00677	Fgf7	2	126034658	126091185	+	3248	protein_coding	fibroblast growth factor 7 [Source:MGI Symbol;Acc:MGI:95521]	-
ENSMUSG0000002248	531.1142077	701.4758194	411.1143778	262.7059811	320.9857247	327.9095712	547.9014683	303.8670923	0.8512968	0.0007521	0.0067717	Pde1b	15	103503034	103530052	+	5396	protein_coding	phosphodiesterase 1B, Ca2+-calmodulin dependent [Source:MGI Symbol;Acc:MGI:97523]	-
ENSMUSG0000006428	299.6285813	360.2173127	377.5996187	225.6687444	207.4061606	205.3083665	345.8151709	212.7944238	0.699447776	0.0007528	0.0067717	Cdpf1	15	85806972	85811697	-	1215	protein_coding	cysteine rich, DPF motif domain containing 1 [Source:MGI Symbol;Acc:MGI:1919605]	-
ENSMUSG0000004467	148.3111372	161.1498504	280.4068174	109.3890479	58.27125464	77.8420347	196.6226017	81.8341124	1.26129411	0.0007528	0.0067717	Zfp612	8	110079746	110092741	+	6083	protein_coding	zinc finger protein 612 [Source:MGI Symbol;Acc:MGI:2443465]	zf-C2
ENSMUSG0000002880	1274.674098	1071.172535	1228.874499	1844.971185	1557.521501	1839.01807	1191.573711	1747.170252	-0.552231972	0.0007533	0.0067728	Hdac1	4	129516104	129542713	-	3445	protein_coding	histone deacetylase 1 [Source:MGI Symbol;Acc:MGI:108086]	-
ENSMUSG0000008664	2.004204557	1.053266996	10.05442772	28.42392582	28.64180313	36.00194105	4.37063309	31.02255667	-2.846892613	0.0007538	0.0067746	Gm13470	2	46419535	46442710	-	421	lincRNA	predicted gene 13470 [Source:MGI Symbol;Acc:MGI:3649387]	-
ENSMUSG0000008409	88.18500052	50.55681581	53.62361449	23.25593931	15.80237414	25.29866128	64.12181027	21.45232491	1.579490697	0.0007542	0.0067746	Gm16418	1	166431705	166431875	-	171	processed_pseudog	predicted pseudogene 16418 [Source:MGI Symbol;Acc:MGI:3644548]	-
ENSMUSG0000002898	1203.524837	1128.048953	1746.118947	728.6860984	846.4146649	865.0196106	1359.230912	813.373458	0.740761675	0.0007554	0.0067821	Klhl7	5	24100605	24160792	+	3959	protein_coding	kelch-like 7 [Source:MGI Symbol;Acc:MGI:1196453]	-
ENSMUSG0000004857	3583.517748	2767.985666	2943.713004	4274.786178	4619.231491	4672.468133	3098.405473	4522.191934	-0.545358859	0.0007583	0.0068048	Mlec	5	115142981	115158179	-	6007	protein_coding	malectin [Source:MGI Symbol;Acc:MGI:1924015]	-
ENSMUSG0000010554	35.07357975	33.70454388	73.73246992	9.474641942	9.876483837	16.54143237	47.50353118	11.96418605	1.990142166	0.0007604	0.0068207	Gm43136	5	110153487	110154122	-	636	antisense	predicted gene 43136 [Source:MGI Symbol;Acc:MGI:5663273]	-
ENSMUSG0000003867	596.2508558	555.0717069	660.2407534	697.6781793	1424.188969	1370.019811	603.8544387	1163.96232	-0.946512175	0.0007642	0.0068511	Trps1	15	50654752	50890463	-	13380	protein_coding	transcriptional repressor GATA binding 1 [Source:MGI Symbol;Acc:MGI:1927616]	zf-G/
ENSMUSG0000004518	133.2796031	310.7137638	285.9926106	129.1996628	86.91305777	89.51833991	243.3286592	101.8770202	1.253898604	0.0007645	0.0068511	Shroom2	X	152609509	152769465	-	8375	protein_coding	shroom family member 2 [Source:MGI Symbol;Acc:MGI:107194]	-
ENSMUSG0000001833	435.9144912	392.8685895	368.6623496	554.6972191	655.7985268	613.9790487	399.1484768	608.1582649	-0.606659762	0.0007671	0.0068666	Ksr1	11	79013440	79146407	-	7103	protein_coding	kinase suppressor of ras 1 [Source:MGI Symbol;Acc:MGI:105051]	-
ENSMUSG0000010471	68.14295495	109.5397676	53.62361449	136.0903115	207.4061606	188.7669342	77.10211234	177.4211354	-1.200586539	0.0007672	0.0068666	Gbp6	5	105270702	105293698	-	5954	protein_coding	guanylate binding protein 6 [Source:MGI Symbol;Acc:MGI:2140937]	-
ENSMUSG0000007099	91.19130735	48.45028182	82.66973901	27.56259474	26.66650636	28.21773758	74.10377606	27.48227956	1.431194826	0.0007674	0.0068666	1700055D18Ri	4	45011687	45015406	+	3720	antisense	RIKEN cDNA 1700055D18 gene [Source:MGI Symbol;Acc:MGI:1920655]	-
ENSMUSG0000011189	43.09039798	45.29048083	43.56918677	11.19730411	7.90118707	18.48748324	43.9833552	12.52865814	1.812810832	0.0007697	0.0068842	Gm19810	10	79888712	79888962	+	251	processed_pseudog	predicted gene, 19810 [Source:MGI Symbol;Acc:MGI:5011995]	-
ENSMUSG0000000989	412.8661388	380.2293856	367.545191	708.0141524	556.04604	556.5705481	386.8802385	606.8769135	-0.649815849	0.0007703	0.0068863	Snap47	11	59407134	59451186	-	2651	protein_coding	synaptosomal-associated protein, 47 [Source:MGI Symbol;Acc:MGI:1915076]	-
ENSMUSG0000002760	1606.369953	1078.545404	1136.150332	2120.597133	1956.531448	2022.919877	1273.688563	2033.349486	-0.674627533	0.0007717	0.006896	Map1lc3a	2	155276297	155278073	+	1181	protein_coding	microtubule-associated protein 1 light chain 3 alpha [Source:MGI Symbol;Acc:MGI:1915661]	-
ENSMUSG0000003204	381.8009682	322.2997008	386.5368878	230.8367309	211.3567541	240.3372821	363.5458522	227.5102557	0.675949252	0.0007736	0.006909	Rpusd4	9	35267865	35277731	+	2647	protein_coding	RNA pseudouridylate synthase domain containing 4 [Source:MGI Symbol;Acc:MGI:1919239]	-
ENSMUSG0000003294	769.61455	772.0447081	508.307179	214.4714403	458.2688501	324.9904949	683.3221457	332.5769284	1.040051279	0.0007812	0.0069736	Ucp3	7	100472990	100486432	+	3135	protein_coding	uncoupling protein 3 (mitochondrial, proton carrier) [Source:MGI Symbol;Acc:MGI:1099787]	-
ENSMUSG0000004064	377.792559	439.2123374	361.9593978	566.7558543	631.1073172	591.5994637	392.9880981	596.4875451	-0.601620902	0.000788	0.007031	Ppip5k2	1	97706048	97770411	-	14381	protein_coding	diphosphoinositol pentakisphosphate kinase 2 [Source:MGI Symbol;Acc:MGI:2142810]	-
ENSMUSG0000004680	8.016818229	8.426135969	6.702951811	202.4128051	5.925890302	24.32563584	7.715302003	77.55477709	-3.33043679	0.0007891	0.0070375	Lrrc75b	10	75550123	75560330	-	4603	protein_coding	leucine rich repeat containing 75B [Source:MGI Symbol;Acc:MGI:2143657]	-
ENSMUSG0000007054	7.01471595	5.26633498	14.52306226	46.51187862	31.60474828	36.00194105	8.934704396	38.03952265	-2.099796724	0.0007899	0.0070415	Mrgprb1	7	48444113	48456342	-	4185	protein_coding	MAS-related GPR, member B1 [Source:MGI Symbol;Acc:MGI:3033115]	-
ENSMUSG0000004676	1511.170236	1723.144806	2319.221327	1109.394438	1243.449315	1082.004282	1851.178789	1144.949345	0.693007696	0.0007908	0.0070457	Rhoj	12	75308322	75401456	+	6484	protein_coding	ras homolog family member J [Source:MGI Symbol;Acc:MGI:1931551]	-
ENSMUSG0000002464	1131.373473	1020.615719	1129.44738	2118.874471	1391.596573	1718.362916	1093.812191	1742.944653	-0.672426524	0.0007912	0.0070457	Cndp2	18	84667470	84685633	-	2119	protein_coding	CNDP dipeptidase 2 (metallopeptidase M20 family) [Source:MGI Symbol;Acc:MGI:1913304]	-
ENSMUSG0000005121	38.07988659	15.79900494	41.3348695	92.16242616	82.96246423	88.54531447	31.73792034	87.89006829	-1.470647853	0.0007917	0.0070457	Gpr183	14	121952551	121965195	-	2887	protein_coding	G protein-coupled receptor 183 [Source:MGI Symbol;Acc:MGI:2442034]	-
ENSMUSG0000003159	42.0882957	18.95880593	15.64022089	228.2527377	48.3947708	68.11178036	25.56244084	114.9197629	-2.166850067	0.0007919	0.0070457	Pdgfrl	8	40926212	40990785	+	3126	protein_coding	platelet-derived growth factor receptor-like [Source:MGI Symbol;Acc:MGI:1916047]	-
ENSMUSG0000003966	129.2711939	104.2734326	138.5276708	57.70918273	64.19714494	64.21967863	124.0240991	62.0420021	0.999934894	0.0007923	0.0070464	Dusp26	8	31089471	31097047	+	2305	protein_coding	dual specificity phosphatase 26 (putative) [Source:MGI Symbol;Acc:MGI:1914209]	-
ENSMUSG0000006607	1.002102279	31.59800988	8.937269082	0	0	0	13.84579375	0	6.32441489	0.0007941	0.007059	Cyp4a10	4	115518264	115533649	+	2109	protein_coding	cytochrome P450, family 4, subfamily a, polypeptide 10 [Source:MGI Symbol;Acc:MGI:88611]	-
ENSMUSG0000007942	983.0623353	948.9935635	911.6014463	1237.73277	1376.781847	1394.345447	947.8857817	1336.286688	-0.495104404	0.0008016	0.0071223	Arpc4	6	113378115	113390448	+	2321	protein_coding	actin related protein 2/3 complex, subunit 4 [Source:MGI Symbol;Acc:MGI:1915339]	-
ENSMUSG0000002493	1556.264839	1336.595818	1629.934449	1965.557537	2594.552304	2282.717668	1507.598369	2280.942503	-0.5972649	0.0008022	0.0071238	Ehbp1l1	19	5707376	5726317	-	6440	protein_coding	EH domain binding protein 1-like 1 [Source:MGI Symbol;Acc:MGI:3612340]	-
ENSMUSG0000002897	684.4358563	887.9040777	658.0064361	464.2574551	511.6018628	410.6167331	743.4487901	462.1586837	0.685950942	0.0008047	0.0071425	Pex14	4	148960535	149009876	-	6272	protein_coding	peroxisomal biogenesis factor 14 [Source:MGI Symbol;Acc:MGI:1927868]	-
ENSMUSG0000008189	3863.104284	2982.852133	3429.67701	2554.708	992.5866257	1371.965862	3425.211142	1639.753496	1.062450655	0.0008052	0.0071437	Rpl17-ps10	7	72648757	72649311	-	555	processed_pseudog	ribosomal protein L17, pseudogene 10 [Source:MGI Symbol;Acc:MGI:3642871]	-
ENSMUSG0000002942	0	0	0	12.0586352	5.925890302	12.64933064	0	10.21128538	-5.716605962	0.0008064	0.0071507	Rimbp2	5	128757791	128953486	-	7935	protein_coding	RIMS binding protein 2 [Source:MGI Symbol;Acc:MGI:2443235]	-
ENSMUSG0000007086	16.03363646	7.372868973	6.702951811	40.48256102	40.49358373	37.94799192	10.03648575	39.64137889	-1.973371874	0.0008082	0.007164	Skint3	4	112232245	112300468	+	3843	protein_coding	selection and upkeep of intraepithelial T cells 3 [Source:MGI Symbol;Acc:MGI:3045331]	-
ENSMUSG0000003167	402.845116	393.9218565	184.3311748	569.3398476	679.502088	765.7710164	327.0327158	671.5236507	-1.037021025	0.0008164	0.0072324	Cdh11	8	102632095	102785642	-	8904	protein_coding	cadherin 11 [Source:MGI Symbol;Acc:MGI:99217]	-
ENSMUSG0000002480	500.049037	613.0013917	603.265663	283.3779272	421.7258599	306.5030116	572.1053639	337.2022662	0.763250378	0.0008201	0.0072579	Pcgf5	19	36348309	36460970	+	12629	protein_coding	polycomb group ring finger 5 [Source:MGI Symbol;Acc:MGI:1923505]	-
ENSMUSG0000002284	136.2859099	219.0795352	258.0636447	118.8636898	58.27125464	36.00194105	204.4763633	71.0456285	1.521122285	0.0008202	0.0072579	Thpo	16	20724454	20734511	-	2628	protein_coding	thrombopoietin [Source:MGI Symbol;Acc:MGI:101875]	-
ENSMUSG0000006019	46.09670482	60.03621878	67.02951811	19.81061497	23.70356121	19.46050868	57.7208139	20.99156162	1.459146721	0.0008204	0.0072579	Gm11353	3	26492554	26493180	+	627	processed_pseudog	predicted gene 11353 [Source:MGI Symbol;Acc:MGI:3649794]	-
ENSMUSG0000008349	50.10511393	35.81107787	59.20940767	11.19730411	7.90118707	20.43353411	48.37519982	13.17734176	1.877239062	0.0008231	0.0072786	Gm11263	4	79327708	79328456	+	749	processed_pseudog	predicted gene 11263 [Source:MGI Symbol;Acc:MGI:3651899]	-
ENSMUSG0000008499	11.02312506	6.319601977	11.17158635	0	0	0	9.504771131	0	5.782117992	0.0008239	0.0072818	Gm11419	11	81985406	81988769	+	683	lincRNA	predicted gene 11419 [Source:MGI Symbol;Acc:MGI:3651781]	-
ENSMUSG0000005434	45.09460254	62.14275277	40.21771087	197.2448186	103.7030803	88.54531447	49.15168873	129.8310711	-1.40267946	0.0008306	0.0073381	Kcnn4	7	24370263	24386690	+	5115	protein_coding	potassium intermediate/small conductance calcium-activated channel, subfamily N, member 4 [Source:MGI Symbol;Acc:MGI:127	-
ENSMUSG0000003851	152.3195463	127.4453065	124.0046085	315.2471773	224.1961831	211.1465191	134.5898205	250.1966265	-0.894894792	0.0008473	0.0074816	Grtp1	8	13172022	13200620	-	4093	protein_coding	GH regulated TBC protein 1 [Source:MGI Symbol;Acc:MGI:1914040]	-
ENSMUSG0000004577	57.11982988	43.18394684	27.92896588	14.64262846	8.888835454	10.70327977	42.74424753	11.41158123	1.901757561	0.0008482	0.0074863	Ifitm10	7	142325837	142373753	-	4789	protein_coding	interferon induced transmembrane protein 10 [Source:MGI Symbol;Acc:MGI:2444776]	-
ENSMUSG0000010192	89.1871028	57.92968479	40.21771087	163.6529063	149.1349059	124.5472555	62.44483282	145.7783559	-1.220235143	0.0008491	0.007491	Gm28156	11	101339660	101347939	-	1136	antisense	predicted gene 28156 [Source:MGI Symbol;Acc:MGI:5578862]	-
ENSMUSG0000003174	1380.89694	1379.779765	752.9649201	2543.510696	1748.137639	2268.122286	1171.213875	2186.590207	-0.90051893	0.0008498	0.007493	Mmp2	8	92827291	92853420	+	3371	protein_coding	matrix metallopeptidase 2 [Source:MGI Symbol;Acc:MGI:97009]	-
ENSMUSG0000003976	584.2256284	503.4616241	433.4575505	741.6060647	785.1804651	810.5301863	507.0482677	779.105572	-0.618916369	0.0008517	0.0075044	Cc2d2a	5	43662346	43740972	+	6809	protein_coding	coiled-coil and C2 domain containing 2A [Source:MGI Symbol;Acc:MGI:1924487]	-
ENSMUSG0000003792	2670.602573	4064.557338	3792.753567	1287.689973	2246.900073	2196.118404	3509.304492	1910.23615	0.877582226	0.0008519	0.0075044	Ssh2	11	77216287	77460220	+	18519	protein_coding	slingshot protein phosphatase 2 [Source:MGI Symbol;Acc:MGI:2679255]	-
ENSMUSG0000009021	595.2487535	650.9190036	729.5045888	931.9602346	936.2906678	1053.786545	658.5574486	974.0124824	-0.564937856	0.000853	0.0075109	Tmem189	2	167642608	167689822	-	3265	protein_coding	transmembrane protein 189 [Source:MGI Symbol;Acc:MGI:2142624]	-
ENSMUSG0000004189	1154.421825	1240.748521	1043.426165	722.6567808	736.7856943	865.0196106	1146.198837	774.8206952	0.565250131	0.0008542	0.007518	Wipi1	11	109573331	109611967	-	4410	protein_coding	WD repeat domain, phosphoinositide interacting 1 [Source:MGI Symbol;Acc:MGI:1261864]	-
ENSMUSG0000006778	62.13034127	75.83522372	254.7121688	639.1076655	241.973854	421.3200128	130.8925779	434.1338441	-1.73172672	0.0008551	0.0075219	Pi15	1	17601901	17630939	+	6923	protein_coding	peptidase inhibitor 15 [Source:MGI Symbol;Acc:MGI:1934659]	-
ENSMUSG0000001859	31889.90081	19883.57435	37604.67682	61133.83513	42291.10379	59850.79443	29792.71733	54425.24445	-0.869324117	0.0008557	0.0075221	Sparc	11	55394500	55423183	-	3788	protein_coding	secreted acidic cysteine rich glycoprotein [Source:MGI Symbol;Acc:MGI:98373]	-
ENSMUSG0000011679	0	0	0	6.029317599	7.90118707	17.51445781	0	10.48165416	-5.751520602	0.0008559	0.0075221	AC161376.2	16	31590196	31593220	-	1615	lincRNA	novel transcript	-
ENSMUSG0000001800	655.3748902	526.633498	451.3320886	897.5069912	812.8346198	877.6689413	544.4468256	862.6701841	-0.663474173	0.0008602	0.0075562	Cyth4	15	78597047	78622019	+	7552	protein_coding	cytohesin 4 [Source:MGI Symbol;Acc:MGI:2441702]	-
ENSMUSG0000004069	280.588638	147.4573795	180.9796989	688.2035374	351.6028246	346.3970544	203.0085721	462.0678055	-1.186563046	0.0008657	0.0076008	Col16a1	4	130047840	130099283	+	6750	protein_coding	collagen, type XVI, alpha 1 [Source:MGI Symbol;Acc:MGI:1095396]	-
ENSMUSG0000000191	2416.068594	2004.367094	1313.778555	3176.589044	3301.708547	3347.207492	1911.404747	3275.168361	-0.776708762	0.0008662	0.0076022	Nfix	8	84699876	84800344	-	7351	protein_coding	nuclear factor I/X [Source:MGI Symbol;Acc:MGI:97311]	CTF/
ENSMUSG0000001502	167.3510805	171.6825204	210.0258234	478.0387525	249.8750411	346.3970544	183.0198081	358.103616	-0.969899132	0.0008683	0.007617	Galns	8	122578242	122611463	-	2650	protein_coding	galactosamine (N-acetyl)-6-sulfate sulfatase [Source:MGI Symbol;Acc:MGI:1355303]	-
ENSMUSG0000002301	66.13875039	32.65127688	42.45202814	143.8422913	109.6289706	94.38346708	47.08068514	115.9515763	-1.299070066	0.0008722	0.0076457	Racgap1	15	99620496	99651656	-	7079	protein_coding	Rac GTPase-activating protein 1 [Source:MGI Symbol;Acc:MGI:1349423]	-
ENSMUSG0000002390	377.792559	309.6604969	226.7832029	441.8628469	597.5272722	627.6014048	304.7454196	555.6638413	-0.865263816	0.0008724	0.0076457	Paqr4	17	23736186	23740867	-	2644	protein_coding	progestin and adipoQ receptor family member IV [Source:MGI Symbol;Acc:MGI:1923748]	-
ENSMUSG0000006139	25.05255697	27.3849419	21.22601407	74.93580445	47.40712242	77.8420347	24.55450431	66.72832052	-1.442132898	0.0008742	0.0076575	Krt79	15	101929332	101940324	-	4753	protein_coding	keratin 79 [Source:MGI Symbol;Acc:MGI:2385030]	-
ENSMUSG0000000873	4372.172242	7251.743268	4980.293196	3367.804545	3345.165076	3408.508095	5534.736235	3373.825905	0.714117639	0.0008798	0.0077032	Hipk1	3	103739815	103791563	-	10914	protein_coding	homeodomain interacting protein kinase 1 [Source:MGI Symbol;Acc:MGI:1314873]	-
ENSMUSG0000009208	550.154151	716.2215573	576.4538558	453.060151	287.4056797	276.3392232	614.2765214	338.935018	0.85668521	0.0008821	0.0077196	Gm6793	8	112013433	112015036	-	1604	protein_coding	predicted gene 6793 [Source:MGI Symbol;Acc:MGI:3643578]	-
ENSMUSG0000003744	255.536081	381.2826526	887.0239564	196.3834875	170.8631704	222.8228243	507.9475633	196.6898274	1.368147909	0.0008832	0.0077261	Arid5a	1	36307733	36324029	+	12540	protein_coding	AT rich interactive domain 5A (MRF1-like) [Source:MGI Symbol;Acc:MGI:2443039]	ARID
ENSMUSG0000002296	455.9565368	431.8394684	502.7213858	627.0490303	727.8968588	723.9309227	463.505797	692.9589373	-0.580102382	0.0008871	0.0077565	Ifngr2	16	91547072	91565623	+	3767	protein_coding	interferon gamma receptor 2 [Source:MGI Symbol;Acc:MGI:107654]	-
ENSMUSG0000004909	1437.014668	1478.786863	1118.275794	978.4721132	807.8963779	826.0985933	1344.692441	870.8223615	0.626706838	0.0008886	0.007766	Sephs2	7	127271879	127274055	-	2177	protein_coding	selenophosphate synthetase 2 [Source:MGI Symbol;Acc:MGI:108388]	-
ENSMUSG0000000191	13527.37866	8781.086946	21764.48453	26710.7383	28858.09812	31179.627	14690.98338	28916.15447	-0.976961271	0.0008898	0.007773	Slc1a5	7	16781340	16798274	+	4173	protein_coding	solute carrier family 1 (neutral amino acid transporter), member 5 [Source:MGI Symbol;Acc:MGI:105305]	-
ENSMUSG0000003096	1220.560575	1150.16756	1193.125422	847.5497882	889.8711938	829.0176696	1187.951186	855.4795505	0.4737635	0.0008922	0.0077894	Zranb1	7	132931142	132986391	+	5472	protein_coding	zinc finger, RAN-binding domain containing 1 [Source:MGI Symbol;Acc:MGI:106441]	-
ENSMUSG0000004566	638.3391515	639.3330666	493.7841168	981.9174376	813.8222682	955.510976	590.485445	917.0835606	-0.634900184	0.0008925	0.0077894	Mfsd5	15	102279454	102281754	+	1754	protein_coding	major facilitator superfamily domain containing 5 [Source:MGI Symbol;Acc:MGI:2145901]	-
ENSMUSG0000004053	81.17028457	87.42116068	46.92066268	140.396967	148.1472576	173.1985272	71.83736931	153.9142506	-1.096618086	0.0008953	0.0078096	Adam22	5	8072352	8368160	-	14348	protein_coding	a disintegrin and metallopeptidase domain 22 [Source:MGI Symbol;Acc:MGI:1340046]	-
ENSMUSG0000000885	802.6839252	783.6306451	698.224147	1060.298566	1063.697309	1125.790427	761.5129058	1083.262101	-0.508088495	0.0008993	0.0078383	Rala	13	17880571	17944239	-	2899	protein_coding	v-ral simian leukemia viral oncogene A (ras related) [Source:MGI Symbol;Acc:MGI:1927243]	-
ENSMUSG0000000323	1301.73086	1313.423944	1281.380955	920.7629305	973.8213064	941.8886199	1298.845253	945.4909523	0.458244195	0.0008994	0.0078383	Eif2b5	16	20498817	20509323	+	2595	protein_coding	eukaryotic translation initiation factor 2B, subunit 5 epsilon [Source:MGI Symbol;Acc:MGI:2446176]	-
ENSMUSG0000004248	496.0406279	619.3209937	458.0350404	369.5110357	286.4180313	291.9076301	524.465554	315.9455657	0.730436937	0.0009031	0.0078639	Mustn1	14	30879200	30881610	-	1236	protein_coding	musculoskeletal, embryonic nuclear protein 1 [Source:MGI Symbol;Acc:MGI:1913425]	-

ENSMUSG0000005183	25.05255697	7.372868973	34.63191769	0.861331086	2.962945151	2.919076301	22.35244788	2.247784179	3.330579432	0.0009032	0.0078639	Gypa		8	80493781	80510542	+	1848	protein_coding	glycophorin A [Source:MGI Symbol;Acc:MGI:95880]	-
ENSMUSG0000003896	1233.587905	1230.215851	1372.987963	1737.3048	1786.655926	1770.906289	1278.930573	1764.955672	-0.464827689	0.0009049	0.0078751	Ube2l3		16	17152013	17202649	-	5841	protein_coding	ubiquitin-conjugating enzyme E2L 3 [Source:MGI Symbol;Acc:MGI:109240]	-
ENSMUSG0000002886	91.19130735	48.45028182	103.8957531	122.3090142	253.8256346	236.4451804	81.17911408	204.1932764	-1.330296753	0.0009068	0.0078882	Hgf		5	16553495	16620152	+	7186	protein_coding	hepatocyte growth factor [Source:MGI Symbol;Acc:MGI:96079]	-
ENSMUSG0000002758	1690.546544	1789.500626	2082.383696	894.0616668	1397.522463	1126.763452	1854.143622	1139.449194	0.70267267	0.0009088	0.0079006	Helz2		2	181227615	181242027	-	11529	protein_coding	helicase with zinc finger 2, transcriptional coactivator [Source:MGI Symbol;Acc:MGI:2385169]	-
ENSMUSG0000010574	13.02732962	99.00709763	58.09224903	12.0586352	10.86413222	1.94605868	56.7088921	8.289606096	2.768506631	0.0009091	0.0079006	Gm43595		3	90468898	90470959	-	1071	unprocessed_pseud	predicted gene 43595 [Source:MGI Symbol;Acc:MGI:5663732]	-
ENSMUSG0000000162	3508.360077	3750.683773	4912.146519	2204.146248	2804.92141	2738.093571	4057.063457	2582.387076	0.651761123	0.0009119	0.0079215	Ifrd1		12	40201567	40248504	-	5858	protein_coding	interferon-related developmental regulator 1 [Source:MGI Symbol;Acc:MGI:1316717]	-
ENSMUSG0000002560	2017.231887	2152.87774	2352.736086	1381.575061	1621.718646	1569.490025	2174.281904	1524.261244	0.51252167	0.0009138	0.0079341	Mkln1		6	31398735	31516811	+	28943	protein_coding	muskelin 1, intracellular mediator containing kelch motifs [Source:MGI Symbol;Acc:MGI:1351638]	-
ENSMUSG0000002040	989.074949	1003.763447	986.4510749	753.6646999	590.6137335	679.1717528	993.0964904	674.4833954	0.557637136	0.0009145	0.0079362	Tnip1		11	54910785	54962917	-	3539	protein_coding	TNFAIP3 interacting protein 1 [Source:MGI Symbol;Acc:MGI:1926194]	-
ENSMUSG0000005198	284.5970471	450.7982743	281.5239761	158.4849197	216.294996	146.9268405	338.9730992	173.9022521	0.963556311	0.0009151	0.0079383	Sec31b		19	44516957	44545864	-	3950	protein_coding	Sec31 homolog B (S. cerevisiae) [Source:MGI Symbol;Acc:MGI:2685187]	-
ENSMUSG0000004302	9.018920508	11.58593696	4.468634541	24.97860148	40.49358373	43.78614452	8.357830669	36.41944325	-2.115963526	0.0009167	0.0079457	Trpv3		11	73267388	73300363	+	5835	protein_coding	transient receptor potential cation channel, subfamily V, member 3 [Source:MGI Symbol;Acc:MGI:2181407]	-
ENSMUSG0000004565	293.6159676	359.1640457	383.1854119	236.0047175	179.7520058	132.331459	345.3218084	182.6960608	0.916573868	0.0009168	0.0079457	Plekha7		7	116123485	116308376	-	12933	protein_coding	pleckstrin homology domain containing, family A member 7 [Source:MGI Symbol;Acc:MGI:2445094]	-
ENSMUSG0000010709	120.2522734	180.1086563	140.761988	63.73850033	87.90070615	58.38152603	147.0409726	70.00691084	1.071553516	0.000921	0.0079784	Gm43597		5	150599932	150607003	-	4620	lincRNA	predicted gene 43597 [Source:MGI Symbol;Acc:MGI:5663734]	-
ENSMUSG0000001986	109.2291484	123.2322385	90.48984945	211.026116	172.8384672	202.3892902	107.6504121	195.4179578	-0.859830188	0.0009245	0.0080046	Qrsl1		10	43874188	43901745	-	3876	protein_coding	glutaminyl-tRNA synthase (glutamine-hydrolyzing)-like 1 [Source:MGI Symbol;Acc:MGI:1923813]	-
ENSMUSG0000002393	2.004204557	2.106533992	2.23431727	102.4983992	5.925890302	3.892101735	2.115018607	37.43879707	-4.149787552	0.0009274	0.0080264	Crisp2		17	40764020	40807003	-	2818	protein_coding	cysteine-rich secretory protein 2 [Source:MGI Symbol;Acc:MGI:98815]	-
ENSMUSG0000002606	0	5.26633498	1.117158635	17.22662171	31.60474828	20.43353411	2.127831205	23.08830137	-3.443612076	0.000928	0.008028	Slc9a2		1	40680574	40769273	+	6984	protein_coding	solute carrier family 9 (sodium/hydrogen exchanger), member 2 [Source:MGI Symbol;Acc:MGI:105075]	-
ENSMUSG0000008144	25.05255697	38.97087886	71.49815265	8.613310856	10.86413222	13.62235607	45.17386283	11.03326638	2.034526454	0.0009288	0.0080306	Gm6161		1	9574548	9575649	+	1102	processed_pseudog	predicted gene 6161 [Source:MGI Symbol;Acc:MGI:3644104]	-
ENSMUSG0000002349	383.8051727	369.6967156	309.452942	671.8382468	507.6512692	534.1909631	354.3182768	571.2268264	-0.689081299	0.00093	0.0080373	Pcbp4		9	106453291	106464012	+	4268	protein_coding	poly(rC) binding protein 4 [Source:MGI Symbol;Acc:MGI:1890471]	-
ENSMUSG0000003158	1496.138702	1537.769814	1877.943666	1218.783486	1055.796122	989.5668662	1637.284061	1088.048825	0.58911544	0.0009327	0.0080573	Gsr		8	33652523	33698163	+	2754	protein_coding	glutathione reductase [Source:MGI Symbol;Acc:MGI:95804]	-
ENSMUSG0000006433	2421.079105	1689.440262	5601.433397	6027.594937	9007.35326	8059.569668	3237.317588	7698.172622	-1.249787516	0.0009337	0.0080592	mt-Rnr2	MT		1094	2675	+	1582	Mt_rRNA	mitochondrially encoded 16S rRNA [Source:MGI Symbol;Acc:MGI:102492]	-
ENSMUSG0000005784	6083.762933	5752.944333	4772.50169	4226.551637	2904.673897	3445.483061	5536.402985	3525.569532	0.651014538	0.000934	0.0080592	Rpl32		6	115805505	115808747	-	963	protein_coding	ribosomal protein L32 [Source:MGI Symbol;Acc:MGI:98038]	-
ENSMUSG0000007388	972.0392103	960.5795004	1034.488896	1597.769164	1396.534815	1291.204751	989.035869	1428.50291	-0.53075764	0.0009342	0.0080592	Il11ra1		4	41699989	41769474	+	4471	protein_coding	interleukin 11 receptor, alpha chain 1 [Source:MGI Symbol;Acc:MGI:107426]	-
ENSMUSG0000003719	1210.539553	1318.690279	1562.904931	992.2534106	813.8222682	901.9945771	1364.044921	902.6900853	0.595074082	0.0009361	0.0080718	Rbm17		2	11585437	11604153	-	2405	protein_coding	RNA binding motif protein 17 [Source:MGI Symbol;Acc:MGI:1924188]	-
ENSMUSG0000003815	20.04204557	17.90553893	21.22601407	48.23454079	65.18479333	50.59732256	19.72453286	54.67221889	-1.47027964	0.0009426	0.0081237	5033430I15Rik		13	45965253	45966508	+	952	lincRNA	RIKEN cDNA 5033430I15 gene [Source:MGI Symbol;Acc:MGI:1923250]	-
ENSMUSG0000005362	242.5087514	552.965173	237.9547893	168.8208928	153.0854995	121.6281792	344.4762379	147.8448572	1.21988412	0.0009439	0.0081316	Tll1		8	64014931	64206271	-	7758	protein_coding	tolloid-like [Source:MGI Symbol;Acc:MGI:106923]	-
ENSMUSG0000002814	981.0581308	856.3060678	1093.698304	683.896882	669.6256042	616.898125	977.0208342	656.8068704	0.572604699	0.0009517	0.0081944	Mrpl9		3	94443318	94451130	+	2703	protein_coding	mitochondrial ribosomal protein L9 [Source:MGI Symbol;Acc:MGI:2137211]	-
ENSMUSG0000003043	62.13034127	11.58593696	50.27213858	3.445324342	2.962945151	11.67630521	41.32947227	6.028191566	2.783419302	0.0009541	0.0082117	Sbk2		7	4940512	4964406	-	3147	protein_coding	SH3-binding domain kinase family, member 2 [Source:MGI Symbol;Acc:MGI:2685925]	-
ENSMUSG0000001625	621.3034127	686.7300815	733.9732233	913.8722818	1027.154319	1026.541833	680.6689058	989.1894778	-0.539403002	0.0009547	0.0082128	Prelid3b		2	174465067	174473081	-	1860	protein_coding	PRELI domain containing 3B [Source:MGI Symbol;Acc:MGI:1913640]	-
ENSMUSG0000002843	1739.649556	1515.651207	1371.870804	2163.663687	2132.332861	2534.731255	1542.390522	2276.909268	-0.561659316	0.0009558	0.0082185	Nol6		4	41114427	41124455	-	5242	protein_coding	nucleolar protein family 6 (RNA-associated) [Source:MGI Symbol;Acc:MGI:2140151]	-
ENSMUSG0000008529	10.02102279	7.372868973	21.22601407	0	0	0.973025434	12.87330194	0.324341811	5.255831126	0.0009575	0.0082271	F730035M05R		12	70227841	70234165	+	1296	lincRNA	RIKEN cDNA F730035M05 gene [Source:MGI Symbol;Acc:MGI:3041236]	-
ENSMUSG0000003201	16.03363646	32.65127688	49.15497995	143.8422913	65.18479333	99.24859425	32.61329776	102.7585596	-1.661553207	0.0009579	0.0082271	Trim29		9	43310848	43336115	+	3473	protein_coding	tripartite motif-containing 29 [Source:MGI Symbol;Acc:MGI:1919419]	-
ENSMUSG0000002645	3734.835192	3865.489876	3314.609671	2423.785675	2630.107646	2754.635003	3638.31158	2602.842775	0.48333489	0.0009581	0.0082271	Adipor1		1	134415378	134433351	+	5025	protein_coding	adiponectin receptor 1 [Source:MGI Symbol;Acc:MGI:1919924]	-
ENSMUSG0000002954	1713.594896	1214.416847	1041.191848	1910.432348	2464.182717	2324.557761	1323.067864	2233.057609	-0.754756386	0.0009601	0.0082407	Acads		5	115110299	115119346	-	2269	protein_coding	acyl-Coenzyme A dehydrogenase, short chain [Source:MGI Symbol;Acc:MGI:87868]	-
ENSMUSG0000002667	2658.577345	2024.379167	5322.143738	1598.630495	1705.668759	1485.809837	3335.033417	1596.70303	1.0625142	0.0009612	0.0082466	Uap1		1	170141938	170174957	-	4011	protein_coding	UDP-N-acetylglucosamine pyrophosphorylase 1 [Source:MGI Symbol;Acc:MGI:1334459]	-
ENSMUSG0000002579	5864.302534	6676.659488	5288.628979	4527.156186	3546.645346	3963.132592	5943.197001	4012.311375	0.566739305	0.000962	0.0082491	Rpl14		9	120571444	120574654	+	1651	protein_coding	ribosomal protein L14 [Source:MGI Symbol;Acc:MGI:1914365]	-
ENSMUSG0000007958	1459.060918	1035.361457	1236.694609	1250.438751	2787.143739	2479.268805	1243.705661	2255.617098	-0.85862544	0.0009718	0.0083268	Tmem182		1	40805601	40856887	+	2716	protein_coding	transmembrane protein 182 [Source:MGI Symbol;Acc:MGI:1923725]	-
ENSMUSG0000001792	198.4162512	294.9147589	250.2435343	473.7320971	420.7382115	372.6687411	247.8581815	422.3796832	-0.769956803	0.0009719	0.0083268	B4galt5		2	167298444	167349183	-	4308	protein_coding	UDP-Gal:betaGlcNAc beta 1,4-galactosyltransferase, polypeptide 5 [Source:MGI Symbol;Acc:MGI:1927169]	-
ENSMUSG0000002746	17.03573874	27.3849419	1.117158635	728.6860984	68.14773848	150.8189422	15.17927976	315.8842597	-4.377826225	0.0009752	0.008351	Angpt4		2	151911210	151945337	+	2335	protein_coding	angiopoietin 4 [Source:MGI Symbol;Acc:MGI:1336887]	-
ENSMUSG0000002513	654.3727879	778.3643101	691.5211952	944.0188698	1111.104432	1061.570748	708.0860978	1038.898017	-0.552876675	0.000982	0.0084056	Pcyt2		11	120610087	120617936	-	4346	protein_coding	phosphate cytidylyltransferase 2, ethanolamine [Source:MGI Symbol;Acc:MGI:1915921]	-
ENSMUSG0000007907	16.03363646	46.34374783	70.38099402	6.890648685	9.876483837	10.70327977	44.25279277	9.156804098	2.274340671	0.0009903	0.0084729	Gm3086		12	69963409	69969736	+	620	transcribed_process	predicted gene 3086 [Source:MGI Symbol;Acc:MGI:3781262]	-
ENSMUSG0000002793	2584.421777	4264.678067	1976.253626	1669.259644	1641.471614	1355.424429	2941.78449	1555.385229	0.919421598	0.0010087	0.0086259	Npr1		3	90450591	90465866	-	4783	protein_coding	natriuretic peptide receptor 1 [Source:MGI Symbol;Acc:MGI:97371]	-
ENSMUSG0000004440	952.999267	912.1292186	799.8855828	1422.918953	1197.029841	1266.879115	888.3380228	1295.609303	-0.544407405	0.0010103	0.0086321	Sptssa		12	54645392	54656573	-	1518	protein_coding	serine palmitoyltransferase, small subunit A [Source:MGI Symbol;Acc:MGI:1913399]	-
ENSMUSG0000005973	849.7827323	785.7371791	635.6632634	1134.37304	1142.70918	1113.141096	757.0610583	1130.074439	-0.577476227	0.0010103	0.0086321	Ndufs8		19	3908870	3912717	-	963	protein_coding	NADH:ubiquinone oxidoreductase core subunit S8 [Source:MGI Symbol;Acc:MGI:2385079]	-
ENSMUSG0000002121	209.4393762	150.6171804	186.5654921	314.3858462	280.492141	325.9635203	182.2073496	306.9471692	-0.752162417	0.0010112	0.0086354	Akr1c13		13	4191150	4205596	+	1434	protein_coding	aldo-keto reductase family 1, member C13 [Source:MGI Symbol;Acc:MGI:1351662]	-
ENSMUSG0000000377	1163.440745	1279.7194	1685.792381	888.0323492	894.8094357	876.6959158	1376.317509	886.5125669	0.634307574	0.0010161	0.0086738	Brd8		18	34598615	34624601	-	10633	protein_coding	bromodomain containing 8 [Source:MGI Symbol;Acc:MGI:1925906]	-
ENSMUSG0000008496	98.2060233	38.97087886	35.74907633	99.91440593	204.4432154	209.2004683	57.64199283	171.1860299	-1.566541374	0.0010169	0.0086762	B430010I23Rik		8	41017468	41024216	+	3270	antisense	RIKEN cDNA B430010I23 gene [Source:MGI Symbol;Acc:MGI:1926099]	-
ENSMUSG0000004436	27.05676152	53.7166168	21.22601407	9.474641942	6.913538686	3.892101735	33.99979746	6.760094121	2.323306711	0.0010173	0.0086762	BC024139		15	76119517	76126596	-	4567	protein_coding	cDNA sequence BC024139 [Source:MGI Symbol;Acc:MGI:2442591]	-
ENSMUSG0000003053	77.16187545	61.08948577	45.80350404	17.22662171	18.76531929	28.21773758	61.35162176	21.40322619	1.523612019	0.0010204	0.0086979	Hddc3		7	80343104	80350547	+	3775	protein_coding	HD domain containing 3 [Source:MGI Symbol;Acc:MGI:1915945]	-
ENSMUSG0000002270	2174.561945	2957.573725	1760.642009	2901.824427	5077.500341	4823.287075	2297.59256	4267.537281	-0.893151711	0.0010212	0.0087009	Zbtb20		16	42875881	43642602	+	30730	protein_coding	zinc finger and BTB domain containing 20 [Source:MGI Symbol;Acc:MGI:1929213]	ZBTI
ENSMUSG0000006771	369.7757408	411.8273955	442.3948195	291.1299069	201.4802703	241.3103076	407.9993186	244.6401616	0.736291501	0.0010223	0.0087061	Prkag1		15	98812797	98831521	-	2175	protein_coding	protein kinase, AMP-activated, gamma 1 non-catalytic subunit [Source:MGI Symbol;Acc:MGI:108411]	-
ENSMUSG0000000165	1778.731545	975.3252384	1060.183545	1717.494185	2790.106684	2895.723691	1271.413443	2467.774853	-0.95647177	0.0010231	0.0087092	Hoxc8		15	102990607	102994111	+	2832	protein_coding	homeobox C8 [Source:MGI Symbol;Acc:MGI:96198]	Hom
ENSMUSG0000001584	205.4309671	151.6704474	71.49815265	283.3779272	348.6398795	350.2891562	142.8665224	327.4356543	-1.194423338	0.0010259	0.0087295	Rxrg		1	167598384	167639623	+	2297	protein_coding	retinoid X receptor gamma [Source:MGI Symbol;Acc:MGI:98216]	RXR
ENSMUSG0000004334	171.3594896	224.3458702	506.0728618	160.2075819	78.02422232	85.62623817	300.5927405	107.9526808	1.474924746	0.0010275	0.0087392	Hoxd9		2	74697727	74700208	+	2136	protein_coding	homeobox D9 [Source:MGI Symbol;Acc:MGI:96210]	Hom
ENSMUSG0000002824	3659.677522	3710.659627	5637.182473	2533.174723	2971.833987	2406.291898	4335.839874	2637.100202	0.717304246	0.0010319	0.0087724	Pnisr		4	21847583	21876475	+	10187	protein_coding	PNN interacting serine/arginine-rich [Source:MGI Symbol;Acc:MGI:1913875]	-
ENSMUSG0000007028	117.2459666	114.8061026	156.4022089	258.3993257	203.4555671	232.5530787	129.4847594	231.4693238	-0.840080664	0.0010343	0.0087887	Gmppb		9	108049242	108052801	+	2785	protein_coding	GDP-mannose pyrophosphorylase B [Source:MGI Symbol;Acc:MGI:2660880]	-
ENSMUSG0000002823	5920.420262	5915.14745	5236.122523	4569.361409	3138.746564	3250.877974	5690.563412	3652.995316	0.639369029	0.0010373	0.0088078	Rps20		4	3831334	3835665	-	4080	protein_coding	ribosomal protein S20 [Source:MGI Symbol;Acc:MGI:1914677]	-
ENSMUSG0000004033	1160.434439	1164.913298	1385.276708	926.7922481	673.5761977	800.799932	1236.874815	800.3894593	0.627311987	0.0010375	0.0088078	Nsmce4a		7	130532526	130573118	-	5044	protein_coding	NSE4 homolog A, SMC5-SMC6 complex component [Source:MGI Symbol;Acc:MGI:1915122]	-
ENSMUSG0000002815	878.8436983	808.909053	1085.878193	1324.72721	1384.683034	1433.266464	924.5436483	1380.892236	-0.579028637	0.0010411	0.0088343	Eif4e		3	138526179	138559696	+	6008	protein_coding	eukaryotic translation initiation factor 4E [Source:MGI Symbol;Acc:MGI:95305]	-
ENSMUSG0000002276	3.006306836	6.319601977	5.585793176	12.0586352	31.60474828	50.59732256	4.97056733	31.42023534	-2.662313616	0.0010416	0.0088351	Ncam2		16	81200697	81626828	+	11330	protein_coding	neural cell adhesion molecule 2 [Source:MGI Symbol;Acc:MGI:97282]	-
ENSMUSG0000003121	2.004204557	3.159800988	0	12.91996628	33.58004505	14.59538151	1.721335182	20.36513095	-3.545092742	0.0010444	0.0088521	Gpr165	X		96713441	96719388	+	4545	protein_coding	G protein-coupled receptor 165 [Source:MGI Symbol;Acc:MGI:1923456]	-
ENSMUSG0000000048	2143.496774	1477.733596	1407.61988	1081.831844	1027.154319	1021.676705	1676.283417	1043.554289	0.683921859	0.0010446	0.0088521	Pdgfb		15	79995874	80014977	-	4161	protein_coding	platelet derived growth factor, B polypeptide [Source:MGI Symbol;Acc:MGI:97528]	-
ENSMUSG0000003893	1123.356654	1465.094392	1063.535021	691.6488617	885.9206002	715.1736938	1217.328689	764.2477186	0.67192744	0.0010511	0.008903	Sccpdh		1	179668210	179687189	+	7562	protein_coding	saccharopine dehydrogenase (putative) [Source:MGI Symbol;Acc:MGI:1924486]	-
ENSMUSG0000002808	507.063753	510.8344931	515.0101308	666.6702602	766.4151458	841.6670002	510.969459	758.2508021	-0.569069296	0.0010551	0.0089332	Gatb		3	85574119	85655622	+	4299	protein_coding	glutamyl-tRNA(Gln) amidotransferase, subunit B [Source:MGI Symbol;Acc:MGI:2442496]	-
ENSMUSG0000004408	2666.594163	2762.719331	2452.163204	3681.32906	3356.029208	3866.803074	2627.158899	3634.720447	-0.468302086	0.0010626	0.0089927	S100a1		3	90511034	90514392	-	694	protein_coding	S100 calcium binding protein A1 [Source:MGI Symbol;Acc:MGI:1338917]	-
ENSMUSG0000005393	5.010511393	4.213067984	2.23431727	11.19730411	28.64180313	39.89404278	3.819298883	26.57771668	-2.787655609	0.001066	0.009017	Shisa6		11	66211725	66525964	-	7449	protein_coding	shisa family member 6 [Source:MGI Symbol;Acc:MGI:2685725]	-
ENSMUSG0000011237	46.09670482	32.65127688	46.92066268	13.78129737	11.8517806	13.62235607	41.88954812	13.08514468	1.677217484	0.0010681	0.0090309	Gm38560		10	82845733	82859008	-	646	bidirectional_prom	predicted gene, 38560 [Source:MGI Symbol;Acc:MGI:5621445]	-
ENSMUSG0000002658	574.2046056	449.7450073	570.8680626	774.3366459	790.118707	833.8827967	531.6058919	799.4460499	-0.588577928	0.0010708	0.0090492	Prrx1		1	163245119	163313710	-	11066	protein_coding	paired related homeobox 1 [Source:MGI Symbol;Acc:MGI:97712]	Hom
ENSMUSG0000003446	52.10931849	20.01207293	62.56088357	7.75197977	11.8517806	12.64933064	44.89409166	10.75103034	2.065993486	0.0010712	0.0090492	Six4		12	73099609	73113456	-	10855	protein_coding	sine oculis-related homeobox 4 [Source:MGI Symbol;Acc:MGI:106034]	Hom
ENSMUSG0000001552	2150.51149	2077.042516	2865.511899	1343.676494	1595.05214	1638.57483	2364.355302	1525.767821	0.631937031	0.0010756	0.0090815	Arnt		3	95434388	95497240	+	8007	protein_coding	aryl hydrocarbon receptor nuclear translocator [Source:MGI Symbol;Acc:MGI:88071]	bHLI
ENSMUSG0000003102	388.8156841	456.0646093	394.3569982	268.7352987	196.5420284	279.2582995	413.0790972	248.1785422	0.734390522	0.0010806	0.0091201	Akip1		7	109703690	109712189	+	1305	protein_coding	A kinase (PRKA) interacting protein 1 [Source:MGI Symbol;Acc:MGI:3041226]	-
ENSMUSG0000003978	165.346876	67.40908775	71.49815265	230.8367309	231.1097218	252.0135873	101.4180388	237.98668	-1.228201166	0.0010831	0.0091368	Cercam		2	29869164	29882840	+	3383	protein_coding	cerebral endothelial cell adhesion molecule [Source:MGI Symbol;Acc:MGI:2139134]	-
ENSMUSG0000003046	5.010511393	3.159800988	16.75737953	42.20522319	38.51828697	38.92101735	8.309230637	39.88150917	-2.275549727	0.0010852	0.009151	Siglecg		7	43408204	43418358	+	4806	protein_coding	sialic acid binding Ig-like lectin G [Source:MGI Symbol;Acc:MGI:2443630]	-
ENSMUSG0000002907	1305.739269	1609.39197	567.5165867	2782.099406	2150.110531	2452.024093	1160.882609	2461.411344	-1.084101671	0.0010878	0.0091681	Mxra8		4	155839680	155844088	+	2918	protein_coding	matrix-remodelling associated 8 [Source:MGI Symbol;Acc:MGI:1922011]	-
ENSMUSG0000007259	122.256478	76.88849072	246.8920584	285.1005893	464.1947404	368.7766394	148.679009	372.6906564	-1.326815764	0.0010885	0.0091701	Ear2		14	44102654	44103534	+	719	protein_coding	eosinophil-associated, ribonuclease A family, member 2 [Source:MGI Symbol;Acc:MGI:108020]	-
ENSMUSG0000004808	157.3300577	403.4012595	186.5654921	106.8050546	93.82659646	111.8979249	249.0989364	104.1765253	1.257453923	0.0010898	0.0091769	Gm4737		16	46152985	46155077	-	2093	protein_coding	predicted gene 4737 [Source:MGI Symbol;Acc:MGI:3643647]	-
ENSMUSG0000006293	3176.664223	2811.169613	3345.890112	860.4697545	2082.950441	1802.043103	3111.241316	1581.8211	0.976156168	0.0010941	0.0092092	Gm10123		9	96895658	96896147	+	490	processed_pseudog	predicted pseudogene 10123 [Source:MGI Symbol;Acc:MGI:3704342]	-
ENSMUSG0000004294	48.10090937	46.34374783	67.02951811	142.9809602	95.80189322	111.8979249	53.82472511	116.8935928	-1.122354532	0.001095	0.0092125	Greb1l		18	10325177	10562934	+	11016	protein_coding	growth regulation by estrogen in breast cancer-like [Source:MGI Symbol;Acc:MGI:3576497]	-
ENSMUSG0000006078	3.006306836	1.053266996	3.351475906	16.36529063	17.77767091	24.32563584	2.470349913	19.48953246	-2.981544371	0.001096	0.0092138	Lrrtm1		6	77242689	77257791	+	3033	protein_coding	leucine rich repeat transmembrane neuronal 1 [Source:MGI Symbol;Acc:MGI:2389173]	-
ENSMUSG0000003231	715.5010269	684.6235475	668.0608639	1096.474472	948.1424484	929.2392893	689.3951461	991.2854032	-0.524156704	0.0010962	0.0092138	Csk		9	57626646	57653631	-	3737	protein_coding	c-src tyrosine kinase [Source:MGI Symbol;Acc:MGI:88537]	-
ENSMUSG0000000523	13.02732962	8.426135969	3.351475906	161.068913	25.67885798	12.64933064	8.268313832	66.46570054	-3.003293528	0.0010966	0.0092138	Dnah2		11	69420809	69549110	-	14968	protein_coding	dynein, axonemal, heavy chain 2 [Source:MGI Symbol;Acc:MGI:107731]	-
ENSMUSG0000003266	1389.91586	1230.215851	1174.133726	1666.675651	1825.174213	1893.507494	1264.755146	1795.119119	-0.5048816	0.0010993	0.0092318	Pon2		6	5264147	5298455	-	7339	protein_coding	paraoxonase 2 [Source:MGI Symbol;Acc:MGI:106687]	-
ENSMUSG0000007570	2921.128142	2801.69021	2950.415956	3545.238748	4402.936495	4319.259901	2891.078102	4089.145048	-0.500087854	0.0011023	0.0092531	Selenot		3	58576636	58593133	+	3436	protein_coding	selenoprotein T [Source:MGI Symbol;Acc:MGI:1916477]	-
ENSMUSG0000000928	1575.304782	953.2066315	1628.81729	2416.895026	2384.183198	2194.172353	1385.776235	2331.750193	-0.750777446	0.0011028	0.0092534	Rarres2		6	48569696	48572789	-	2656	protein_coding	retinoic acid receptor responder (tazarotene induced) 2 [Source:MGI Symbol;Acc:MGI:1918910]	-
ENSMUSG0000002680	8.016818229	4.213067984	7.820110447	77.5197977	23.70356121	17.51445781	6.68333222	39.57927224	-2.569755314	0.0011052	0.0092695	Ak8		2	28700164	28813165	+	1873	protein_coding	adenylate kinase 8 [Source:MGI Symbol;Acc:MGI:1916120]	-
ENSMUSG0000002562	285.5991494	295.9680259	249.1263757	575.3691652	404.9358373	411.5897585	276.8978503	463.9649203	-0.745112622	0.0011069	0.0092793	Phf6	X		52912266	52956943	+	6946	protein_coding	PHD finger protein 6 [Source:MGI Symbol;Acc:MGI:1918248]	-
ENSMUSG0000003888	314.6601155	346.5248417	361.9593978	242.8953661	199.5049735	187.7939087	341.0481183	210.0647495	0.697553516	0.0011082	0.0092858	A230050P20Ri		9	20868642	20874307	+	3417	protein_coding	RIKEN cDNA A230050P20 gene [Source:MGI Symbol;Acc:MGI:2441788]	-
ENSMUSG0000009036	0	0	4.468634541	82.6877842	8.888835454	7.78420347	1.489544847	33.12027438	-4.511582044	0.0011098	0.0092949	4933411K16Ri		19	42052248	42053627	+	1380	protein_coding	RIKEN cDNA 4933411K16 gene [Source:MGI Symbol;Acc:MGI:1914015]	-
ENSMUSG0000010788	841.765914	821.548257	1188.656788	1189.498229	2015.790351	1898.372621	950.6569863	1701.220401	-0.839587344	0.0011107	0.0092987	Gm44250		6	5216258	5220486	-	4229	TEC	predicted gene, 44250 [Source:MGI Symbol;Acc:MGI:5690642]	-
ENSMUSG0000000547	7.01471595	17.90553893	11.17158635	62.01583816	27.65415474	51.57034799	12.03061375	47.08011363	-1.97256583	0.0011124	0.009309	Asf1b		8	83955507	83970197	+	2338	protein_coding	anti-silencing function 1B histone chaperone [Source:MGI Symbol;Acc:MGI:1914179]	-
ENSMUSG0000002045	884.856312	922.6618886	1117.158635	724.379443	571.8484142	606.1948452	974.8922786	634.1409008	0.619669279	0.0011133	0.0093122	Limk2		11	3344256	3409189	-	6099	protein_coding	LIM motif-containing protein kinase 2 [Source:MGI Symbol;Acc:MGI:1197517]	-
ENSMUSG0000002259	181.3805124	151.6704474	159.7536848	391.9056439	308.1462957	221.8497989	164.2682149	307.3005795	-0.90412906	0.0011143	0.009316	Lynx1		15	74747852	74753046	-	4662	protein_coding	Ly6/neurotoxin 1 [Source:MGI Symbol;Acc:MGI:1345180]	-
ENSMUSG0000003735	871.8289824	1001.656913	1161.844981	551.2518948	678.5144396	712.2546175	1011.776959	647.3403173	0.644467197	0.0011157	0.0093237	Uvssa		5	33378549	33419754	+	8666	protein_coding	UV stimulated scaffold protein A [Source:MGI Symbol;Acc:MGI:1918351]	-
ENSMUSG0000004175	17.03573874	10.53266996	24.57748997	0.861331086	2.962945151	0	17.38196622	1.274758746	3.784339686	0.0011187	0.0093449	Trem3		17	48247777	48258841	+	994	protein_coding	triggering receptor expressed on myeloid cells 3 [Source:MGI Symbol;Acc:MGI:1930003]	-
ENSMUSG0000003792	54.11352305	15.79900494	14.52306226	73.21314228	124.4436964	100.2216197	28.14519675	99.29281944	-1.812420347	0.0011197	0.0093479	Bank1		3	136053363	136326066	-	7486	protein_coding	B cell scaffold protein with ankyrin repeats 1 [Source:MGI Symbol;Acc:MGI:2442120]	-
ENSMUSG0000003553	1.002102279	2.106533992	3.351475906	130.922325	6.913538686	0.973025434	2.153370725	46.26962971	-4.43643741	0.0011204	0.0093479	Ccdc180		4	45890303	45950774	+	5292	protein_coding	coiled-coil domain containing 180 [Source:MGI Symbol;Acc:MGI:2685871]	-
ENSMUSG0000002820	5305.129463	6216.381811	8350.760798	3562.46537	3859.729884	4731.822684	6624.090691	4051.339313	0.709310464	0.0011206	0.0093479	Asph		4	9448069	9669344	-	16102	protein_coding	aspartate-beta-hydroxylase [Source:MGI Symbol;Acc:MGI:1914186]	-
ENSMUSG0000002855	8.016818229	7.372868973	0	150.73294	10.86413222	19.46050868	5.129895734	60.35252696	-3.549262301	0.0011215	0.0093511	Ttc39a		4	109406623	109444745	+	4904	protein_coding	tetratricopeptide repeat domain 39A [Source:MGI Symbol;Acc:MGI:2444350]	-
ENSMUSG0000003554	3.006306836	40.02415485	44.68634541	4.306655428	0.987648384	0	29.2389327	1.764767937	4.015287385	0.0011233	0.0093618	Gc		5	89417522	89457898	-	1845	protein_coding	vitamin D binding protein [Source:MGI Symbol;Acc:MGI:95669]	-
ENSMUSG0000001850	6.012613672	4.213067984	17.87453816	38.75989885	42.4688885	41.84009365	9.36673994	41.02295767	-2.141533338	0.0011238	0.0093624	Adora2b		11	62248984	62266453	+	1845	protein_coding	adenosine A2b receptor [Source:MGI Symbol;Acc:MGI:99403]	-
ENSMUSG0000002889	646.3559697	555.0717069	759.6678719	434.9721982	415.7999696	419.373962	653.6985162	423.3820432	0.626220397	0.0011282	0.0093949	Trnau1ap		4	132311763	132329538	-	2710	protein_coding	tRNA selenocysteine 1 associated protein 1 [Source:MGI Symbol;Acc:MGI:1919037]	-
ENSMUSG0000002635	52.10931849	55.82315079	60.3265663	94.74641942	115.5548609	133.3044844	56.08634519	114.5352549	-1.029776181	0.0011287	0.009395	Rgs18		1	144752683	144775435	-	2031	protein_coding	regulator of G-protein signaling 18 [Source:MGI Symbol;Acc:MGI:1927498]	-
ENSMUSG0000008510	30.06306836	33.70454388	51.38929722	10.33597303	12.83942899	8.757228904	38.38563648	10.64421031	1.849537431	0.0011305	0.0094056	Gm12758		4	34234377	34240207	+	5232	antisense	predicted gene 12758 [Source:MGI Symbol;Acc:MGI:3702543]	-
ENSMUSG0000002676	1021.142222	785.7371791	868.0322596	1230.842121	1377.769495	1337.909971	891.6372202	1315.507196	-0.560726074	0.0011322	0.0094153	Mmadhc		2	50279881	50296801	-	2012	protein_coding	methylmalonic aciduria (cobalamin deficiency) cblD type, with homocystinuria [Source:MGI Symbol;Acc:MGI:1923786]	-
ENSMUSG0000009559	1164.442848	1451.401921	1110.455588	937.9895522	694.3168138	708.3625158	1242.100151	780.2229606	0.670383017	0.0011391	0.0094668	Gm6472	X		152909505	152910155	-	651	transcribed_process	predicted pseudogene 6472 [Source:MGI Symbol;Acc:MGI:3648396]	-
ENSMUSG0000005023	12.02522734	3.159800988	14.52306226	50.81853405	38.51828697	36.00194105	9.902696863	41.77958736	-2.080676681	0.0011533	0.0095825	Cxcr3	X		101731535	101734269	-	1731	protein_coding	chemokine (C-X-C motif) receptor 3 [Source:MGI Symbol;Acc:MGI:1277207]	-
ENSMUSG0000003586	4.008409114	2.106533992	0	261.84465	6.913538686	27.24471215	2.038314369	98.66763362	-5.586545429	0.0011558	0.0095989	Cdhr3		12	33033796	33092875	-	4569	protein_coding	cadherin-related family member 3 [Source:MGI Symbol;Acc:MGI:1916014]	-
ENSMUSG0000005672	789.6565956	891.0638787	932.8274604	571.9238408	646.9096914	575.0580314	871.1826449	597.9638545	0.542899607	0.0011656	0.0096755	Nbeal2		9	110624789	110654161	-	9554	protein_coding	neurobeachin-like 2 [Source:MGI Symbol;Acc:MGI:2448554]	-
ENSMUSG0000004316	309.6496041	293.8614919	239.0719479	408.2709346	541.2313143	432.996318	280.8610146	460.8328556	-0.713208712	0.0011673	0.0096858	Pyurf		6	57686576	57692078	-	3573	protein_coding	Pigy upstream reading frame [Source:MGI Symbol;Acc:MGI:1913709]	-
ENSMUSG0000003359	1.002102279	4.213067984	1.117158635	86.99443964	8.888835454	4.865127169	2.110776299	33.58280076	-3.994671936	0.0011694	0.0096992	Myo5c		9	75232020	75305451	+	9697	protein_coding	myosin VC [Source:MGI Symbol;Acc:MGI:2442485]	-
ENSMUSG0000000187	1077.25995	881.5844757	796.5341069	1372.100419	1445.917234	1302.881056	918.4595107	1373.632903	-0.580305206	0.0011726	0.0097211	Ltbp1		17	75005568	75392512	+	10480	protein_coding	latent transforming growth factor beta binding protein 1 [Source:MGI Symbol;Acc:MGI:109151]	-
ENSMUSG0000003990	164.3447737	135.8714425	176.5110644	100.775737	45.43182565	33.08286475	158.9090935	59.7634758	1.406131537	0.0011781	0.0097623	Eva1c		16	90826719	90905109	+	3806	protein_coding	eva-1 homolog C (C. elegans) [Source:MGI Symbol;Acc:MGI:1918217]	-
ENSMUSG0000006387	1256.636257	681.4637465	706.0442575	1315.252568	1711.594649	1932.428511	881.3814204	1653.091909	-0.906875276	0.0011793	0.0097679	Slc24a3		2	145167754	145642166	+	4564	protein_coding	solute carrier family 24 (sodium/potassium/calcium exchanger), member 3 [Source:MGI Symbol;Acc:MGI:2137513]	-
ENSMUSG0000006807	445.935514	254.8906131	317.2730524	512.4919959	627.1567237	685.0099054	339.3663931	608.2195417	-0.84086568	0.0011806	0.0097748	Tcf15		2	152143561	152149097	+	980	protein_coding	transcription factor 15 [Source:MGI Symbol;Acc:MGI:104664]	bHLI
ENSMUSG0000002743	477.0006846	337.0454388	459.1521991	285.9619204	200.4926219	248.1214856	424.3994408	244.858676	0.79254209	0.0011935	0.009877	Dtd1		2	144599897	144768758	+	2353	protein_coding	D-tyrosyl-tRNA deacylase 1 [Source:MGI Symbol;Acc:MGI:1913294]	-
ENSMUSG0000006414	1307.743474	1376.619964	1333.88741	906.9816331	1049.870232	895.1833991	1339.416949	950.6784214	0.494754825	0.0011944	0.0098799	Arih2		9	108602942	108649386	-	7672	protein_coding	ariadne RBR E3 ubiquitin protein ligase 2 [Source:MGI Symbol;Acc:MGI:1344361]	-
ENSMUSG0000003330	1844.870295	6561.853386	1261.272099	737.2994093	591.6013819	617.8711504	3222.66526	648.9239805	2.312102465	0.0011953	0.009883	Dpyd		3	118562129	119432924	+	8884	protein_coding	dihydropyrimidine dehydrogenase [Source:MGI Symbol;Acc:MGI:2139667]	-
ENSMUSG0000004553	369.7757408	973.2187044	403.2942673	217.9167647	302.2204054	233.5261041	582.0962375	251.2210914	1.212641155	0.0011975	0.0098971	Kcna5		6	126532551	126535412	-	2862	protein_coding	potassium voltage-gated channel, shaker-related subfamily, member 5 [Source:MGI Symbol;Acc:MGI:96662]	-
ENSMUSG0000005854	3285.893372	2869.099297	2516.958405	2192.087613	1170.363335	822.2064915	2890.650358	1394.885813	1.050979733	0.0011994	0.0099085	Rpl23a		11	78180932	78183584	-	847	protein_coding	ribosomal protein L23A [Source:MGI Symbol;Acc:MGI:3040672]	-
ENSMUSG0000003061	131.2753985	139.0312435	78.20110447	209.3034538	266.6650636	210.1734937	116.1692488	228.7140037	-0.975246421	0.0012014	0.0099205	Sytl2		7	90302252	90410719	+	13941	protein_coding	synaptotagmin-like 2 [Source:MGI Symbol;Acc:MGI:1933366]	-
ENSMUSG0000008168	372.7820476	696.2094844	392.122681	250.6473459	256.7885798	264.662918	487.038071	257.3662812	0.92030954	0.0012066	0.0099586	Rps2-ps13	X		52898588	52899469	+	882	processed_pseudog	ribosomal protein S2, pseudogene 13 [Source:MGI Symbol;Acc:MGI:3705640]	-
ENSMUSG0000002966	2396.026548	1546.19595	1467.946447	2626.19848	3316.523273	3149.683329	1803.389648	3030.801694	-0.748723589	0.0012103	0.0099853	Tspan12		6	21771395	21852515	-	3311	protein_coding	tetraspanin 12 [Source:MGI Symbol;Acc:MGI:1889818]	-
ENSMUSG0000003467	287.603354	234.8785401	443.5119782	562.4491989	581.724898	598.4106418	321.9979574	580.8615796	-0.851949482	0.0012316	0.010156	Dbn1		13	55473429	55488111	-	4514	protein_coding	drebrin 1 [Source:MGI Symbol;Acc:MGI:1931838]	-
ENSMUSG0000006879	176.370001	215.9197342	253.5950102	400.5189548	364.4422536	320.1253677	215.2949151	361.6955254	-0.750034307	0.0012339	0.0101709	Col28a1		6	7997808	8192617	-	4904	protein_coding	collagen, type XXVIII, alpha 1 [Source:MGI Symbol;Acc:MGI:2685312]	-
ENSMUSG0000003135	3263.847121	1894.827326	2522.544198	3484.084241	4817.748816	4935.185	2560.406215	4412.339352	-0.785041462	0.0012357	0.010181	Syap1	X		162857057	162888447	-	2351	protein_coding	synapse associated protein 1 [Source:MGI Symbol;Acc:MGI:1914293]	-
ENSMUSG0000003686	284.5970471	236.9850741	270.3523897	386.7376574	416.7876179	408.6706822	263.9781703	404.0653192	-0.613751422	0.001237	0.0101869	Mrpl55		11	59202486	59206134	+	1027	protein_coding	mitochondrial ribosomal protein L55 [Source:MGI Symbol;Acc:MGI:1914462]	-
ENSMUSG0000003936	7847.462944	7286.501079	10327.01442	4627.070592	5728.360626	5999.674825	8486.992816	5451.702014	0.638566014	0.0012432	0.0102335	Picalm		7	90130213	90213465	+	15732	protein_coding	phosphatidylinositol binding clathrin assembly protein [Source:MGI Symbol;Acc:MGI:2385902]	-
ENSMUSG0000007270	2010.217171	1959.076613	2437.640142	2666.681041	3439.979321	3823.989955	2135.644642	3310.216772	-0.632234294	0.0012446	0.0102389	Smim10l1		6	133104909	133110899	+	3068	protein_coding	small integral membrane protein 10 like 1 [Source:MGI Symbol;Acc:MGI:1914379]	-
ENSMUSG0000006041	651.3664811	858.4126018	706.0442575	540.9159218	431.6023437	348.3431053	738.6077801	440.2871236	0.745579715	0.0012449	0.0102389	Rps16-ps2		5	129128077	129128517	-	441	processed_pseudog	ribosomal protein S16, pseudogene 2 [Source:MGI Symbol;Acc:MGI:3704259]	-
ENSMUSG0000003008	3381.093088	5890.922309	7420.167655	1968.141531	3325.412108	3129.249795	5564.061017	2807.601145	0.986838107	0.0012483	0.010262	Aldh1l1		6	90486427	90600203	+	4516	protein_coding	aldehyde dehydrogenase 1 family, member L1 [Source:MGI Symbol;Acc:MGI:1340024]	-
ENSMUSG0000002248	431.9060821	522.4204301	428.9889159	618.4357195	718.020375	820.2604407	461.1051427	718.9055117	-0.640288839	0.0012496	0.0102645	Nckap1l		15	103453794	103498810	+	5935	protein_coding	NCK associated protein 1 like [Source:MGI Symbol;Acc:MGI:1926063]	-
ENSMUSG0000004447	622.305515	725.7009603	703.8099402	519.3826446	267.652712	333.7477238	683.9388052	373.5943601	0.871146578	0.0012497	0.0102645	Ascc1		10	60002805	60099988	+	2193	protein_coding	activating signal cointegrator 1 complex subunit 1 [Source:MGI Symbol;Acc:MGI:1916340]	-
ENSMUSG0000000621	0	0	0	54.26385839	0.987648384	0.973025434	0	18.74151074	-6.594413563	0.0012625	0.0103617	Fam131c		4	141368220	141384175	+	1433	protein_coding	family with sequence similarity 131, member C [Source:MGI Symbol;Acc:MGI:2685539]	-
ENSMUSG0000002014	3035.367802	2942.827987	2971.64197	3837.229986	3897.260522	4352.342765	2983.279253	4028.944425	-0.433444293	0.0012626	0.0103617	Rab1a		11	20201432	20226856	+	3799	protein_coding	RAB1A, member RAS oncogene family [Source:MGI Symbol;Acc:MGI:97842]	-
ENSMUSG0000002958	19541.99654	15332.40766	18777.20234	24006.15869	30301.05241	24864.69193	17883.86885	26390.63434	-0.561355232	0.0012742	0.0104526	Actb		5	142903115	142906754	-	2679	protein_coding	actin, beta [Source:MGI Symbol;Acc:MGI:87904]	-
ENSMUSG0000000609	530.1121054	548.752105	594.3283939	928.5149103	748.6374749	793.0157285	557.7308681	823.3893712	-0.562686279	0.0012822	0.0105133	Tbcb		7	30224131	30232272	-	1442	protein_coding	tubulin folding cofactor B [Source:MGI Symbol;Acc:MGI:1913661]	-
ENSMUSG0000004678	1572.298475	1620.977907	1798.625403	956.077505	1255.301096	1173.468673	1663.967262	1128.282425	0.560755823	0.0012896	0.010569	Epm2aip1		9	111271929	111279093	+	7165	protein_coding	EPM2A (laforin) interacting protein 1 [Source:MGI Symbol;Acc:MGI:1925031]	-
ENSMUSG0000004389	176.370001	140.0845105	94.95848399	274.7646163	240.9862056	261.7438417	137.1376652	259.1648879	-0.916520653	0.0012983	0.0106362	S1pr2		9	20962361	20976781	-	7971	protein_coding	sphingosine-1-phosphate receptor 2 [Source:MGI Symbol;Acc:MGI:99569]	-
ENSMUSG0000004219	3.006306836	0	0	65.4611625	5.925890302	7.78420347	1.002102279	26.39041876	-4.683870649	0.0012998	0.0106437	Slc35f2		9	53771538	53818154	+	2716	protein_coding	solute carrier family 35, member F2 [Source:MGI Symbol;Acc:MGI:1919272]	-
ENSMUSG0000007959	1.002102279	0	0	55.98652056	1.975296767	3.892101735	0.334034093	20.61797302	-5.771007206	0.0013029	0.0106643	Clec2l		6	38663069	38680864	+	1428	protein_coding	C-type lectin domain family 2, member L [Source:MGI Symbol;Acc:MGI:2141402]	-
ENSMUSG0000008639	11.02312506	16.85227194	14.52306226	35.31457451	53.33301272	44.75916995	14.13281975	44.46891906	-1.654097658	0.0013047	0.0106746	Mccc1os		3	35999833	36025106	+	7442	antisense	methylcrotonoyl-Coenzyme A carboxylase 1 (alpha), opposite strand [Source:MGI Symbol;Acc:MGI:3590669]	-
ENSMUSG0000004802	0	0	0	47.37320971	1.975296767	0.973025434	0	16.77384397	-6.434795251	0.0013054	0.0106758	Tmem229a		6	24951141	24956297	-	5157	protein_coding	transmembrane protein 229A [Source:MGI Symbol;Acc:MGI:2442812]	-
ENSMUSG0000002817	31.06517064	24.22514091	29.04612452	2.583993257	9.876483837	3.892101735	28.11214535	5.45085961	2.380582078	0.001306	0.0106758	1810013D15Ri		3	157925220	157938355	+	2121	antisense	RIKEN cDNA 1810013D15 gene [Source:MGI Symbol;Acc:MGI:1916292]	-
ENSMUSG0000007272	0	0	1.117158635	9.474641942	10.86413222	16.54143237	0.372386212	12.29340218	-5.02026846	0.0013082	0.0106891	Klra14-ps		6	130148799	130162569	-	1113	transcribed_unproc	killer cell lectin-like receptor subfamily A, member 14, pseudogene [Source:MGI Symbol;Acc:MGI:1321089]	-
ENSMUSG0000003657	35.07357975	24.22514091	90.48984945	14.64262846	10.86413222	8.757228904	49.92952337	11.42132986	2.121919183	0.0013116	0.010712	Fxyd7		7	31042513	31051467	-	693	protein_coding	FXYD domain-containing ion transport regulator 7 [Source:MGI Symbol;Acc:MGI:1889006]	-
ENSMUSG0000003198	945.984551	729.9140283	909.3671291	456.5054754	629.1320204	547.8133192	861.7552361	544.483605	0.66299751	0.0013225	0.0107962	2810004N23Ri		8	124836945	124863120	-	6324	protein_coding	RIKEN cDNA 2810004N23 gene [Source:MGI Symbol;Acc:MGI:1913773]	-
ENSMUSG0000001449	628.3181287	681.4637465	631.1946289	801.0379096	1082.462629	1087.842435	646.992168	990.4476577	-0.613905595	0.0013237	0.0108017	Ankrd52		10	128377115	128408704	+	8401	protein_coding	ankyrin repeat domain 52 [Source:MGI Symbol;Acc:MGI:2444029]	-
ENSMUSG0000004032	469.9859687	439.2123374	565.2822694	696.8168482	730.859804	787.1775759	491.4935252	738.2847427	-0.587306214	0.0013282	0.0108333	Slc25a24		3	109123149	109168457	+	3431	protein_coding	solute carrier family 25 (mitochondrial carrier, phosphate carrier), member 24 [Source:MGI Symbol;Acc:MGI:1917160]	-
ENSMUSG0000000345	199.4183534	265.423283	380.9510946	109.3890479	172.8384672	131.3584336	281.9309104	137.8619829	1.032474527	0.0013303	0.0108459	Bicd1		6	149408886	149563329	+	27373	protein_coding	BICD cargo adaptor 1 [Source:MGI Symbol;Acc:MGI:1101760]	-
ENSMUSG0000002296	213.4477853	258.050414	290.4612452	135.2289804	170.8631704	128.4393573	253.9864815	144.843836	0.810229045	0.0013362	0.0108889	Donson		16	91676808	91688770	-	3130	protein_coding	downstream neighbor of SON [Source:MGI Symbol;Acc:MGI:1890621]	-
ENSMUSG0000005189	289.6075585	219.0795352	205.5571889	132.6449872	137.2831253	142.0617133	238.0814275	137.329942	0.795032616	0.0013371	0.0108916	Klhdc1		12	69241176	69284632	+	3137	protein_coding	kelch domain containing 1 [Source:MGI Symbol;Acc:MGI:2672853]	-
ENSMUSG0000003752	391.8219909	461.3309443	349.6706528	277.3486096	226.1714799	243.2563584	400.941196	248.9254826	0.68712163	0.0013379	0.0108939	Atg14		14	47540893	47570649	-	4322	protein_coding	autophagy related 14 [Source:MGI Symbol;Acc:MGI:1261775]	-
ENSMUSG0000002771	606.2718786	564.5511099	329.5617974	780.3659635	971.8460096	920.4820604	500.128262	890.8980112	-0.832153063	0.0013484	0.0109744	Acad9		3	36065979	36092853	+	8192	protein_coding	acyl-Coenzyme A dehydrogenase family, member 9 [Source:MGI Symbol;Acc:MGI:1914272]	-
ENSMUSG0000003915	261.5486947	416.0404635	216.7287752	798.4539163	577.7743045	436.8884198	298.1059778	604.3722135	-1.019774125	0.0013495	0.0109787	Fam102a		2	32535332	32569756	+	6066	protein_coding	family with sequence similarity 102, member A [Source:MGI Symbol;Acc:MGI:2138935]	-
ENSMUSG0000007863	4.008409114	1.053266996	2.23431727	21.53327714	17.77767091	17.51445781	2.431997794	18.94180195	-2.952774557	0.0013501	0.0109787	Tomt		7	101898370	101906359	-	2857	protein_coding	transmembrane O-methyltransferase [Source:MGI Symbol;Acc:MGI:3769724]	-
ENSMUSG0000002838	1083.272563	1938.011273	1471.297923	900.9523155	908.636513	867.9386869	1497.527253	892.5091718	0.746524639	0.0013618	0.0110686	Ugcg		4	59189257	59222833	+	5212	protein_coding	UDP-glucose ceramide glucosyltransferase [Source:MGI Symbol;Acc:MGI:1332243]	-
ENSMUSG0000003591	45.09460254	55.82315079	32.39760042	132.6449872	76.04892555	108.0058231	44.43845125	105.5665786	-1.248083194	0.0013662	0.0111	Cd276		9	58524298	58555437	-	3658	protein_coding	CD276 antigen [Source:MGI Symbol;Acc:MGI:2183926]	-
ENSMUSG0000002184	1520.189157	1746.31668	1348.410473	905.258971	1087.400871	1102.437816	1538.305436	1031.699219	0.576704066	0.0013815	0.0112194	Peli2		14	48120823	48281575	+	9089	protein_coding	pellino 2 [Source:MGI Symbol;Acc:MGI:1891445]	-
ENSMUSG0000007867	3.006306836	5.26633498	23.46033134	0	0	0	10.57765772	0	5.933124871	0.0013822	0.0112204	Mup20		4	62050234	62054158	-	928	protein_coding	major urinary protein 20 [Source:MGI Symbol;Acc:MGI:3651981]	-
ENSMUSG0000003264	82.17238685	100.0603646	78.20110447	221.362089	173.8261155	127.4663318	86.81128531	174.2181788	-1.005912257	0.0013876	0.0112589	Pygm		19	6384399	6398459	+	3218	protein_coding	muscle glycogen phosphorylase [Source:MGI Symbol;Acc:MGI:97830]	-
ENSMUSG0000003827	6560.763618	6574.49259	5522.115134	4925.952478	3731.335594	4023.460169	6219.123781	4226.91608	0.557036056	0.0013882	0.0112592	Fau		19	6057888	6059524	+	815	protein_coding	Finkel-Biskis-Reilly murine sarcoma virus (FBR-MuSV) ubiquitously expressed (fox derived) [Source:MGI Symbol;Acc:MGI:102	-
ENSMUSG0000010929	20.04204557	10.53266996	26.81180725	1.722662171	3.950593535	0.973025434	19.12884093	2.215427047	3.118080681	0.0013984	0.0113298	Gm2814		8	10898169	10900558	-	1277	lincRNA	predicted gene 2814 [Source:MGI Symbol;Acc:MGI:3780984]	-
ENSMUSG0000004746	12.02522734	1.053266996	2.23431727	36.17590559	23.70356121	43.78614452	5.104270537	34.55520377	-2.743601507	0.0013987	0.0113298	Gpr141b		13	19727426	19732951	-	2764	lincRNA	G protein-coupled receptor 141B [Source:MGI Symbol;Acc:MGI:2441809]	-
ENSMUSG0000011317	22.04625013	26.3316749	39.10055223	7.75197977	5.925890302	6.811178036	29.15949242	6.829682703	2.088502939	0.0013987	0.0113298	Gm36839		13	34872512	34875432	-	2093	lincRNA	predicted gene, 36839 [Source:MGI Symbol;Acc:MGI:5595998]	-
ENSMUSG0000002084	724.5199474	669.8778095	854.6263559	1039.62662	1196.042193	1076.16613	749.6747043	1103.944981	-0.558482802	0.0014007	0.0113409	Blmh		11	76924809	76987379	+	5891	protein_coding	bleomycin hydrolase [Source:MGI Symbol;Acc:MGI:1345186]	-
ENSMUSG0000002640	234.4919332	249.6242781	233.4861548	153.3169332	147.1596092	148.8728914	239.2007887	149.7831446	0.675096734	0.0014065	0.011377	Ddx59		1	136415271	136440158	+	2953	protein_coding	DEAD (Asp-Glu-Ala-Asp) box polypeptide 59 [Source:MGI Symbol;Acc:MGI:1915247]	-
ENSMUSG0000010118	886.8605166	885.7975437	977.5138058	1268.740689	1304.683515	1222.119945	916.7239554	1265.181383	-0.464981223	0.001407	0.011377	Eif4a-ps4		1	60703932	60705149	+	1218	processed_pseudog	eukaryotic translation initiation factor 4A, pseudogene 4 [Source:MGI Symbol;Acc:MGI:1195958]	-
ENSMUSG0000005943	25.05255697	17.90553893	29.04612452	252.3700081	62.22184818	28.21773758	24.0014068	114.2698646	-2.253590843	0.001407	0.011377	Actg2		6	83512905	83536265	-	1557	protein_coding	actin, gamma 2, smooth muscle, enteric [Source:MGI Symbol;Acc:MGI:104589]	-
ENSMUSG0000004012	39.08198887	44.23721384	67.02951811	19.81061497	14.81472576	15.56840694	50.11624027	16.73124922	1.577412554	0.0014081	0.0113806	Rep15		6	147032584	147034245	+	1662	protein_coding	RAB15 effector protein [Source:MGI Symbol;Acc:MGI:1913782]	-
ENSMUSG0000001539	141.2964213	42.13067984	118.4188153	232.5593931	205.4308638	341.5319273	100.6153055	259.8407281	-1.368318621	0.0014096	0.0113881	Cd83		13	43784775	43803132	+	2572	protein_coding	CD83 antigen [Source:MGI Symbol;Acc:MGI:1328316]	-
ENSMUSG0000000756	3133.573825	3186.132663	2862.160423	4074.957366	4101.703738	4069.192364	3060.622304	4081.951156	-0.415362785	0.001413	0.0114108	Ppp2r1a		17	20945311	20965916	+	3411	protein_coding	protein phosphatase 2, regulatory subunit A, alpha [Source:MGI Symbol;Acc:MGI:1926334]	-
ENSMUSG0000003213	21.04414785	8.426135969	7.820110447	40.48256102	60.24655141	38.92101735	12.43013142	46.55004326	-1.896113099	0.0014166	0.0114346	Abcg4		9	44273188	44288615	-	5211	protein_coding	ATP binding cassette subfamily G member 4 [Source:MGI Symbol;Acc:MGI:1890594]	-
ENSMUSG0000003526	735.5430725	835.2407279	1207.648485	664.086267	387.1581664	439.8074961	926.144095	497.0173098	0.896976744	0.00142	0.0114574	Pkig		2	163658386	163726158	+	3990	protein_coding	protein kinase inhibitor, gamma [Source:MGI Symbol;Acc:MGI:1343086]	-
ENSMUSG0000003058	107.2249438	72.67542273	153.050733	52.54119622	41.48123212	46.70522082	110.9836999	46.90921639	1.239960178	0.0014253	0.0114954	2200002D01Ri		7	29246561	29248466	-	1564	protein_coding	RIKEN cDNA 2200002D01 gene [Source:MGI Symbol;Acc:MGI:1919525]	-
ENSMUSG0000002043	1083.272563	1015.349384	1154.02487	840.6591395	707.1562428	705.4434395	1084.215606	751.0862739	0.529052081	0.0014262	0.0114975	Pes1		11	3963975	3980004	+	3024	protein_coding	pescadillo ribosomal biogenesis factor 1 [Source:MGI Symbol;Acc:MGI:1890613]	-
ENSMUSG0000002842	1629.418305	2018.059565	1963.964881	1349.705811	1349.127692	1234.769275	1870.480917	1311.200926	0.512328155	0.0014289	0.0115141	Nfx1		4	40970906	41025993	+	8564	protein_coding	nuclear transcription factor, X-box binding 1 [Source:MGI Symbol;Acc:MGI:1921414]	zf-NI
ENSMUSG0000003685	10.02102279	6.319601977	16.75737953	68.04515576	25.67885798	41.84009365	11.0326681	45.1880358	-2.041671614	0.0014313	0.0115286	Wnt4		4	137277489	137299726	+	4194	protein_coding	wingless-type MMTV integration site family, member 4 [Source:MGI Symbol;Acc:MGI:98957]	-
ENSMUSG0000002408	2267.757457	2461.48497	7080.55143	1864.7818	1565.422688	1636.62878	3936.597952	1688.944423	1.220708151	0.0014352	0.0115553	Cyp1b1		17	79706953	79715041	-	5128	protein_coding	cytochrome P450, family 1, subfamily b, polypeptide 1 [Source:MGI Symbol;Acc:MGI:88590]	-
ENSMUSG0000002104	621.3034127	580.3501149	608.8514562	871.6670586	798.0198941	873.7768395	603.5016613	847.8212641	-0.490494378	0.0014418	0.0116033	Mthfd1		12	76255298	76319803	+	4442	protein_coding	methylenetetrahydrofolate dehydrogenase (NADP+ dependent), methenyltetrahydrofolate cyclohydrolase, formyltetrahydrofolate s	-
ENSMUSG0000002098	914.9193804	722.5411593	880.3210046	1115.423756	1273.078767	1339.856022	839.2605148	1242.786182	-0.566146821	0.0014453	0.0116262	Sec23a		12	58958383	59012017	-	4840	protein_coding	SEC23 homolog A, COPII coat complex component [Source:MGI Symbol;Acc:MGI:1349635]	-
ENSMUSG0000003768	24.05045469	196.9609283	119.535974	25.83993257	27.65415474	30.16378845	113.5157856	27.88595859	2.02507666	0.0014488	0.0116481	Aspg		12	112106679	112127559	+	3521	protein_coding	asparaginase [Source:MGI Symbol;Acc:MGI:2144822]	-
ENSMUSG0000006688	493.0343211	638.2797996	499.3699099	296.2978934	374.3187374	356.1273088	543.5613436	342.2479799	0.668130788	0.0014498	0.0116481	Zfp617		8	71908635	71934629	+	3024	protein_coding	zinc finger protein 617 [Source:MGI Symbol;Acc:MGI:2684459]	zf-C2
ENSMUSG0000001991	702.4736973	673.0376105	789.8311551	1031.874641	1062.709661	973.9984592	721.780821	1022.86092	-0.503257462	0.0014499	0.0116481	P4ha1		10	59323296	59373304	+	4114	protein_coding	procollagen-proline, 2-oxoglutarate 4-dioxygenase (proline 4-hydroxylase), alpha 1 polypeptide [Source:MGI Symbol;Acc:MGI:97	-
ENSMUSG0000005511	398.8367069	544.539037	577.5710144	692.5102928	928.3894807	860.1544835	506.9822528	827.0180523	-0.706174908	0.0014551	0.0116847	Arntl		7	113207465	113314126	+	7523	protein_coding	aryl hydrocarbon receptor nuclear translocator-like [Source:MGI Symbol;Acc:MGI:1096381]	bHLI
ENSMUSG0000003212	511.0721621	718.3280913	422.2859641	946.6028631	886.9082486	913.6708823	550.5620725	915.7273313	-0.733820091	0.0014578	0.0117021	Hmbs		9	44336339	44344228	-	3909	protein_coding	hydroxymethylbilane synthase [Source:MGI Symbol;Acc:MGI:96112]	-
ENSMUSG0000002717	1724.618022	1751.583015	1894.701045	1385.881717	1204.931028	1296.069878	1790.300694	1295.627541	0.466242503	0.0014619	0.0117298	Eif3m		2	104999656	105017080	-	2590	protein_coding	eukaryotic translation initiation factor 3, subunit M [Source:MGI Symbol;Acc:MGI:1351744]	-
ENSMUSG0000004672	736.5451748	999.5503793	692.6383538	1291.996628	1309.621757	1165.68447	809.5779693	1255.767618	-0.633268642	0.0014665	0.0117614	Cdc42se1		3	95228732	95236409	+	3258	protein_coding	CDC42 small effector 1 [Source:MGI Symbol;Acc:MGI:1889510]	-
ENSMUSG0000002786	300.6306836	232.7720061	224.5488857	460.8121308	367.4051988	406.7246313	252.6505251	411.6473203	-0.703890419	0.0014731	0.0118091	Vangl1		3	102153583	102204693	-	7156	protein_coding	VANGL planar cell polarity 1 [Source:MGI Symbol;Acc:MGI:2159344]	-
ENSMUSG0000007652	0	5.26633498	0	8.613310856	57.28360626	19.46050868	1.755444993	28.45247526	-4.014896608	0.0014777	0.0118412	Igkv15-103		6	68437440	68437925	+	375	IG_V_gene	immunoglobulin kappa chain variable 15-103 [Source:MGI Symbol;Acc:MGI:96513]	-
ENSMUSG0000003717	379.7967636	459.2244103	310.5701006	222.2234201	249.8750411	222.8228243	383.1970915	231.6404285	0.726901513	0.0014783	0.0118412	E330009J07Rik		6	40401375	40436135	-	7811	protein_coding	RIKEN cDNA E330009J07 gene [Source:MGI Symbol;Acc:MGI:2444256]	-
ENSMUSG0000003386	2982.256381	4676.505463	3134.74713	1595.185171	2443.442101	2244.769676	3597.836325	2094.465649	0.780698547	0.0014814	0.011861	Klf9		19	23141226	23166911	+	3263	protein_coding	Kruppel-like factor 9 [Source:MGI Symbol;Acc:MGI:1333856]	zf-C2
ENSMUSG0000002895	1657.477169	1498.798935	1482.469509	1952.637571	2231.097699	2288.55582	1546.248538	2157.430363	-0.480271926	0.00149	0.0119244	Vamp3		4	151047300	151057963	-	2889	protein_coding	vesicle-associated membrane protein 3 [Source:MGI Symbol;Acc:MGI:1321389]	-
ENSMUSG0000003926	115.241762	75.83522372	142.9963053	233.4207242	191.6037864	223.7958498	111.3577637	216.2734535	-0.959052841	0.0014927	0.0119409	Gimap3		6	48764464	48770851	-	2577	protein_coding	GTPase, IMAP family member 3 [Source:MGI Symbol;Acc:MGI:1932723]	-
ENSMUSG0000003091	1231.5837	861.5724028	960.7564263	696.8168482	562.9595787	678.1987273	1017.970843	645.9917181	0.656073453	0.0014951	0.0119551	Tmem159		7	120102353	120120992	+	1962	protein_coding	transmembrane protein 159 [Source:MGI Symbol;Acc:MGI:1925752]	-
ENSMUSG0000000590	548.1499464	630.9069307	502.7213858	320.4151638	395.0593535	377.5338683	560.5927543	364.3361285	0.622547767	0.0014963	0.0119597	Pex1		5	3596066	3637232	+	10356	protein_coding	peroxisomal biogenesis factor 1 [Source:MGI Symbol;Acc:MGI:1918632]	-
ENSMUSG0000001986	1465.073531	1339.755619	1605.356959	985.3627619	1038.018451	1103.410842	1470.062036	1042.264018	0.496201143	0.0014976	0.0119627	Gopc		10	52335850	52382124	-	10958	protein_coding	golgi associated PDZ and coiled-coil motif containing [Source:MGI Symbol;Acc:MGI:2149946]	-
ENSMUSG0000005606	136.2859099	77.94175771	105.0129117	235.1433864	169.875522	218.9307226	106.4135264	207.9832103	-0.96641468	0.001498	0.0119627	Otulinl		15	27655069	27681579	-	3241	protein_coding	OTU deubiquitinase with linear linkage specificity like [Source:MGI Symbol;Acc:MGI:2687281]	-
ENSMUSG0000009381	16.03363646	15.79900494	21.22601407	28.42392582	89.87600292	64.21967863	17.68621849	60.83986912	-1.781721909	0.0014988	0.0119643	Gm5627		9	102739648	102756685	+	931	lincRNA	predicted gene 5627 [Source:MGI Symbol;Acc:MGI:3645308]	-
ENSMUSG0000002118	77.16187545	106.3799666	92.72416672	54.26385839	20.74061606	22.37958498	92.08866959	32.46135314	1.496958302	0.0015016	0.011981	Tc2n		12	101645443	101718523	-	6096	protein_coding	tandem C2 domains, nuclear [Source:MGI Symbol;Acc:MGI:1921663]	-
ENSMUSG0000001012	3.006306836	0	0	44.78921645	4.938241919	16.54143237	1.002102279	22.08963025	-4.424544418	0.0015025	0.0119832	Slc47a1		11	61343401	61378345	-	3383	protein_coding	solute carrier family 47, member 1 [Source:MGI Symbol;Acc:MGI:1914723]	-
ENSMUSG0000002245	2193.601888	2239.245634	2352.736086	1676.150293	1488.386114	1757.283933	2261.861202	1640.60678	0.463151748	0.0015036	0.0119868	Ndufa6		15	82350140	82354322	-	1801	protein_coding	NADH:ubiquinone oxidoreductase subunit A6 [Source:MGI Symbol;Acc:MGI:1914380]	-
ENSMUSG0000003927	0	0	0	33.59191234	5.925890302	0.973025434	0	13.49694269	-6.12159661	0.001507	0.0120092	Pcsk1n	X		7919822	7924410	+	2193	protein_coding	proprotein convertase subtilisin/kexin type 1 inhibitor [Source:MGI Symbol;Acc:MGI:1353431]	-
ENSMUSG0000001892	29.06096608	5.26633498	14.52306226	59.43184491	90.8636513	44.75916995	16.28345444	65.01822205	-1.992488768	0.0015108	0.0120341	Alox15		11	70344152	70352031	-	2454	protein_coding	arachidonate 15-lipoxygenase [Source:MGI Symbol;Acc:MGI:87997]	-
ENSMUSG0000004713	227.4772172	154.8302484	272.586707	1272.186013	370.3681439	285.0964521	218.2980576	642.5502031	-1.558185083	0.0015128	0.0120455	Cd24a		10	43578284	43584265	+	2580	protein_coding	CD24a antigen [Source:MGI Symbol;Acc:MGI:88323]	-
ENSMUSG0000005054	33.06937519	22.11860692	8.937269082	90.43976399	60.24655141	58.38152603	21.37508373	69.68928047	-1.699826029	0.001514	0.0120497	Adra1b		11	43774606	43901210	-	3382	protein_coding	adrenergic receptor, alpha 1b [Source:MGI Symbol;Acc:MGI:104774]	-
ENSMUSG0000008749	8.016818229	14.74573795	35.74907633	0.861331086	1.975296767	2.919076301	19.5038775	1.918568051	3.35786599	0.0015151	0.0120533	Gm13523		2	32873755	32876001	-	488	antisense	predicted gene 13523 [Source:MGI Symbol;Acc:MGI:3651426]	-
ENSMUSG0000002595	353.7421044	307.5539629	370.8966669	243.7566972	206.4185122	197.5241631	344.0642447	215.8997908	0.671137295	0.0015175	0.0120672	Mettl21a		1	64606473	64617242	-	3005	protein_coding	methyltransferase like 21A [Source:MGI Symbol;Acc:MGI:1914349]	-
ENSMUSG0000002745	53.11142077	30.54474289	45.80350404	126.6156696	78.02422232	98.27556881	43.15322257	100.9718202	-1.227141965	0.0015191	0.0120745	Fam110a		2	151969398	151980219	-	2207	protein_coding	family with sequence similarity 110, member A [Source:MGI Symbol;Acc:MGI:1921097]	-
ENSMUSG0000002755	236268.6605	56962.78568	166853.228	269001.45	403790.1652	451573.3196	153361.5581	374788.3116	-1.289139336	0.0015241	0.0121078	Car3		3	14863512	14872523	+	2865	protein_coding	carbonic anhydrase 3 [Source:MGI Symbol;Acc:MGI:88270]	-
ENSMUSG0000011352	31.06517064	16.85227194	53.62361449	3.445324342	6.913538686	9.730254338	33.84701902	6.696372455	2.344798491	0.0015246	0.0121078	Gm32699		13	17944291	17946278	+	1988	TEC	predicted gene, 32699 [Source:MGI Symbol;Acc:MGI:5591858]	-
ENSMUSG0000008556	82.17238685	78.99502471	96.07564263	122.3090142	191.6037864	187.7939087	85.74768473	167.2355698	-0.963027579	0.0015283	0.0121282	A730017L22Ri		2	130873059	130906396	-	6012	processed_transcrip	RIKEN cDNA A730017L22 gene [Source:MGI Symbol;Acc:MGI:3584452]	-
ENSMUSG0000006727	16506.62873	18416.37343	16419.99762	13930.30765	10819.68804	11093.46297	17114.33326	11947.81955	0.51841711	0.0015284	0.0121282	Rplp0		5	115559467	115563727	+	1659	protein_coding	ribosomal protein, large, P0 [Source:MGI Symbol;Acc:MGI:1927636]	-
ENSMUSG0000002000	1.002102279	3.159800988	1.117158635	60.29317599	7.90118707	6.811178036	1.759687301	25.00184703	-3.832762155	0.0015307	0.0121413	Moxd1		10	24223517	24302790	+	3198	protein_coding	monooxygenase, DBH-like 1 [Source:MGI Symbol;Acc:MGI:1921582]	-
ENSMUSG0000007541	2809.894789	2094.948055	2653.251759	1757.976746	1735.29821	1717.389891	2519.364868	1736.888282	0.536559912	0.0015441	0.012242	Fnbp1		2	31026206	31142008	-	10030	protein_coding	formin binding protein 1 [Source:MGI Symbol;Acc:MGI:109606]	-
ENSMUSG0000003005	2254.730127	2139.185269	2101.375393	2744.200839	2926.402161	3084.490625	2165.09693	2918.364542	-0.430579066	0.0015584	0.0123506	Copg1		6	87887814	87913595	+	11011	protein_coding	coatomer protein complex, subunit gamma 1 [Source:MGI Symbol;Acc:MGI:1858696]	-
ENSMUSG0000002574	20.04204557	61.08948577	87.13837355	2.583993257	18.76531929	0.973025434	56.0899683	7.440779327	2.922478623	0.0015605	0.0123615	Il6		5	30013114	30019981	+	2428	protein_coding	interleukin 6 [Source:MGI Symbol;Acc:MGI:96559]	-
ENSMUSG0000009029	9.018920508	13.69247095	3.351475906	173.9888793	11.8517806	22.37958498	8.687622454	69.40674829	-2.996931209	0.0015644	0.0123876	Lrrc10b		19	10455371	10457447	-	2077	protein_coding	leucine rich repeat containing 10B [Source:MGI Symbol;Acc:MGI:2685551]	-

ENSMUSG0000003490	1359.852792	1315.530478	1505.92984	1717.494185	2132.332861	2333.31499	1393.771037	2061.047345	-0.564283616	0.001569	0.0124189	Cobll1	2	65088339	65239403	-	8342	protein_coding	Cobl-like 1 [Source:MGI Symbol;Acc:MGI:2442894]	-
ENSMUSG0000005085	1025.150631	424.4665994	617.7887253	449.6148267	235.0603153	187.7939087	689.1353186	290.8230169	1.243905362	0.0015701	0.0124224	Atp5k	5	108433244	108434448	-	616	protein_coding	ATP synthase, H+ transporting, mitochondrial F1F0 complex, subunit E [Source:MGI Symbol;Acc:MGI:106636]	-
ENSMUSG0000000074	10143.27926	9202.393745	9551.706331	7790.739669	5718.484142	6488.133592	9632.45978	6665.785801	0.531062891	0.0015712	0.0124253	Rpl13	8	123102350	123105244	+	2595	protein_coding	ribosomal protein L13 [Source:MGI Symbol;Acc:MGI:105922]	-
ENSMUSG0000003465	4212.837979	3869.702944	2990.633666	2387.609769	2577.762282	2526.947052	3691.058196	2497.439701	0.563739786	0.0015729	0.0124339	Tmem109	19	10870660	10882001	-	3798	protein_coding	transmembrane protein 109 [Source:MGI Symbol;Acc:MGI:1915789]	-
ENSMUSG0000007879	79.16608001	45.29048083	42.45202814	261.84465	95.80189322	111.8979249	55.63619633	156.5148227	-1.491914192	0.0015807	0.0124901	Dact3	7	16875317	16887462	+	2989	protein_coding	dishevelled-binding antagonist of beta-catenin 3 [Source:MGI Symbol;Acc:MGI:3654828]	-
ENSMUSG0000010376	51.10721621	33.70454388	43.56918677	15.50395954	8.888835454	15.56840694	42.79364895	13.32040064	1.680609742	0.0015856	0.0125221	Gm37858	1	177635707	177636937	-	1231	TEC	predicted gene, 37858 [Source:MGI Symbol;Acc:MGI:5611086]	-
ENSMUSG0000000956	1097.301995	3270.394023	1685.792381	1006.896039	923.4512388	927.2932384	2017.829466	952.5468387	1.082864775	0.0015866	0.0125221	Mkl2	16	13256481	13417529	+	8990	protein_coding	MKL/myocardin-like 2 [Source:MGI Symbol;Acc:MGI:3050795]	-
ENSMUSG0000006684	93.19551191	85.31462668	25.69464861	141.258298	201.4802703	208.2274428	68.0682624	183.655337	-1.428743296	0.0015868	0.0125221	Hmcn1	1	150562524	150993435	-	21191	protein_coding	hemicentin 1 [Source:MGI Symbol;Acc:MGI:2685047]	-
ENSMUSG0000008670	7.01471595	2.106533992	13.40590362	29.28525691	32.59239666	45.73219539	7.509051188	35.86994965	-2.263204215	0.0015902	0.0125397	Gm16263	13	118695292	118715396	-	502	antisense	predicted gene 16263 [Source:MGI Symbol;Acc:MGI:3826536]	-
ENSMUSG0000002182	852.7890391	808.909053	949.5848399	1065.466553	1538.756182	1432.293439	870.427644	1345.505391	-0.628162397	0.0015903	0.0125397	Ap3m1	14	21031442	21052508	-	7015	protein_coding	adaptor-related protein complex 3, mu 1 subunit [Source:MGI Symbol;Acc:MGI:1929212]	-
ENSMUSG0000004919	93.19551191	73.72868973	44.68634541	197.2448186	150.1225543	124.5472555	70.53684902	157.3048762	-1.15540191	0.0015943	0.0125656	Rtl5	X	102066544	102071304	-	4761	protein_coding	retrotransposon Gag like 5 [Source:MGI Symbol;Acc:MGI:3045324]	-
ENSMUSG0000005947	66.13875039	72.67542273	68.14667675	218.7780957	101.7277835	137.1965862	68.98694996	152.5674885	-1.147005062	0.0015963	0.0125703	B3gnt8	7	25626654	25635100	+	6474	protein_coding	UDP-GlcNAc:betaGal beta-1,3-N-acetylglucosaminyltransferase 8 [Source:MGI Symbol;Acc:MGI:2385269]	-
ENSMUSG0000002258	8.016818229	10.53266996	6.702951811	0	0	0	8.41748	0	5.607425159	0.0015965	0.0125703	Ly6g	15	75155240	75159126	+	942	protein_coding	lymphocyte antigen 6 complex, locus G [Source:MGI Symbol;Acc:MGI:109440]	-
ENSMUSG0000004582	27.05676152	13.69247095	24.57748997	58.57051382	56.29595787	61.30060233	21.77557415	58.72235801	-1.430611072	0.0015969	0.0125703	Ptprcap	19	4154606	4156751	+	927	protein_coding	protein tyrosine phosphatase, receptor type, C polypeptide-associated protein [Source:MGI Symbol;Acc:MGI:97811]	-
ENSMUSG0000003499	49298.4216	55584.05919	32330.5709	27499.71757	28636.86489	29176.16763	45737.6839	28437.58336	0.68559295	0.0015987	0.0125789	Eef2	10	81176631	81182498	+	3324	protein_coding	eukaryotic translation elongation factor 2 [Source:MGI Symbol;Acc:MGI:95288]	-
ENSMUSG0000002663	42.0882957	17.90553893	36.86623496	93.88508833	82.96246423	72.0038821	32.28668987	82.95047822	-1.361308726	0.0016118	0.0126773	Batf3	1	191097847	191108945	+	1597	protein_coding	basic leucine zipper transcription factor, ATF-like 3 [Source:MGI Symbol;Acc:MGI:1925491]	TF_b
ENSMUSG0000001806	290.6096608	279.115754	342.967701	407.4096035	549.1325014	494.2969204	304.2310386	483.6130084	-0.668564247	0.0016133	0.0126837	Ints2	11	86210681	86257575	-	10609	protein_coding	integrator complex subunit 2 [Source:MGI Symbol;Acc:MGI:1917672]	-
ENSMUSG0000001838	104.218637	58.98295178	77.08394583	31.00791908	39.50593535	28.21773758	80.0951782	32.91053067	1.285203145	0.0016158	0.0126933	Abi3	11	95830074	95842476	-	4153	protein_coding	ABI gene family, member 3 [Source:MGI Symbol;Acc:MGI:1913860]	-
ENSMUSG0000002249	11.02312506	9.479402965	15.64022089	59.43184491	23.70356121	49.62429712	12.04758297	44.25323441	-1.882430521	0.0016162	0.0126933	Shisa9	16	11984113	12270902	+	5242	protein_coding	shisa family member 9 [Source:MGI Symbol;Acc:MGI:1919805]	-
ENSMUSG0000001834	227.4772172	243.3046761	294.9298797	171.404886	127.4066415	147.8998659	255.2372577	148.9037978	0.775393799	0.0016166	0.0126933	Zkscan6	11	65807175	65829239	+	2371	protein_coding	zinc finger with KRAB and SCAN domains 6 [Source:MGI Symbol;Acc:MGI:1289293]	zf-C2
ENSMUSG0000011378	1.002102279	2.106533992	3.351475906	18.94928388	12.83942899	21.40655954	2.153370725	17.73175747	-3.058180263	0.0016207	0.0127191	Gm33195	13	43864669	43868589	+	2337	lincRNA	predicted gene, 33195 [Source:MGI Symbol;Acc:MGI:5592354]	-
ENSMUSG0000004240	729.5304588	656.1853386	1302.606969	367.7883735	548.144853	481.6475897	896.1075887	465.8602721	0.943867228	0.0016225	0.0127191	Zmym6	4	127077383	127124372	+	7692	protein_coding	zinc finger, MYM-type 6 [Source:MGI Symbol;Acc:MGI:106505]	-
ENSMUSG0000002900	167.3510805	270.689618	188.7998094	118.0023587	83.95011262	119.6821284	208.946836	107.2115332	0.961591401	0.0016225	0.0127191	Fbxo44	4	148152800	148160482	-	3024	protein_coding	F-box protein 44 [Source:MGI Symbol;Acc:MGI:1354744]	-
ENSMUSG0000002686	672.410629	718.3280913	577.5710144	925.930917	927.4018323	978.8635864	656.1032449	944.0654452	-0.524593632	0.0016226	0.0127191	Psmd5	2	34849734	34874968	-	7230	protein_coding	proteasome (prosome, macropain) 26S subunit, non-ATPase, 5 [Source:MGI Symbol;Acc:MGI:1914248]	-
ENSMUSG0000005867	562.1793783	426.5731334	752.9649201	875.112383	1042.956693	1026.541833	580.5724773	981.5369696	-0.757815933	0.0016363	0.012821	Tubb2a	13	34074274	34078007	-	1610	protein_coding	tubulin, beta 2A class IIA [Source:MGI Symbol;Acc:MGI:107861]	-
ENSMUSG0000002064	304.6390927	219.0795352	349.6706528	1051.685256	431.6023437	456.3489284	291.1297602	646.5455092	-1.151850839	0.0016393	0.0128293	Id2	12	25093799	25097140	-	2438	protein_coding	inhibitor of DNA binding 2 [Source:MGI Symbol;Acc:MGI:96397]	bHLH
ENSMUSG0000003228	797.6734138	817.335189	834.5175005	638.2463344	495.7994886	416.4548857	816.5087011	516.8335696	0.658933418	0.0016393	0.0128293	Imp3	9	56937475	56938398	+	924	protein_coding	IMP3, U3 small nucleolar ribonucleoprotein [Source:MGI Symbol;Acc:MGI:1916119]	-
ENSMUSG0000004004	1178.47228	1452.455188	1345.058997	888.0323492	956.0436355	957.4570268	1325.328821	933.8443372	0.505126976	0.0016394	0.0128293	Orc3	4	34570796	34614944	-	6088	protein_coding	origin recognition complex, subunit 3 [Source:MGI Symbol;Acc:MGI:1354944]	-
ENSMUSG0000000315	569.1940943	802.589451	634.5461048	402.241617	408.8864309	468.0252336	668.77655	426.3844272	0.649474934	0.001641	0.0128363	Slc2a3	6	122727809	122801640	-	7245	protein_coding	solute carrier family 2 (facilitated glucose transporter), member 3 [Source:MGI Symbol;Acc:MGI:95757]	-
ENSMUSG0000003139	1569.292168	2044.391239	1578.545152	1080.970512	1208.881622	1226.985072	1730.742853	1172.279069	0.562248193	0.0016448	0.0128611	Mecp2	X	74026592	74135363	-	15067	protein_coding	methyl CpG binding protein 2 [Source:MGI Symbol;Acc:MGI:99918]	MBD
ENSMUSG0000002649	687.4421631	764.6718392	897.0783841	446.1695023	574.8113593	505.9732256	783.0641288	508.9846957	0.621688887	0.0016463	0.0128672	Tfb2m	1	179528055	179546267	-	2804	protein_coding	transcription factor B2, mitochondrial [Source:MGI Symbol;Acc:MGI:107937]	-
ENSMUSG0000007888	3.006306836	0	3.351475906	14.64262846	31.60474828	14.59538151	2.119260914	20.28091941	-3.260002665	0.0016476	0.0128718	Gm14434	2	176473278	176486783	-	2774	protein_coding	predicted gene 14434 [Source:MGI Symbol;Acc:MGI:3702417]	zf-C2
ENSMUSG0000005924	1156.42603	1253.387725	988.6853922	1527.140015	1672.088714	1653.170212	1132.833049	1617.466313	-0.513494075	0.001653	0.012908	Sept9	11	117199661	117362325	+	7565	protein_coding	septin 9 [Source:MGI Symbol;Acc:MGI:1858222]	-
ENSMUSG0000004952	1860.903931	1021.668986	1689.143856	2508.196121	2761.464881	2440.347788	1523.905591	2570.00293	-0.753946151	0.0016536	0.012908	Cdc42ep1	15	78842624	78850897	+	2560	protein_coding	CDC42 effector protein (Rho GTPase binding) 1 [Source:MGI Symbol;Acc:MGI:1929763]	-
ENSMUSG0000002697	81.17028457	45.29048083	102.7785944	167.0982306	161.9743349	159.5761711	76.41311995	162.8829122	-1.093340051	0.0016557	0.0129118	Psd4	2	24367580	24414954	+	11786	protein_coding	pleckstrin and Sec7 domain containing 4 [Source:MGI Symbol;Acc:MGI:2674093]	-
ENSMUSG0000002086	2501.247287	2277.163246	1787.453816	2941.445657	3471.584069	3399.750866	2188.62145	3270.926864	-0.579433666	0.0016559	0.0129118	Ankrd40	11	94328001	94341841	+	4255	protein_coding	ankyrin repeat domain 40 [Source:MGI Symbol;Acc:MGI:1918702]	-
ENSMUSG0000006166	135.2838076	163.2563844	136.2933535	93.88508833	40.49358373	58.38152603	144.9445152	64.25339936	1.169539013	0.0016562	0.0129118	Gdpd1	11	87033867	87074062	-	2336	protein_coding	glycerophosphodiester phosphodiesterase domain containing 1 [Source:MGI Symbol;Acc:MGI:1913819]	-
ENSMUSG0000000809	3194.702064	1488.266265	2779.490684	3789.856777	5070.586802	4911.83239	2487.486338	4590.758656	-0.883981692	0.0016585	0.012925	Fgfrl1	5	108692382	108706924	+	2782	protein_coding	fibroblast growth factor receptor-like 1 [Source:MGI Symbol;Acc:MGI:2150920]	-
ENSMUSG0000004637	31.06517064	55.82315079	34.63191769	15.50395954	10.86413222	6.811178036	40.50674637	11.0597566	1.865703759	0.001661	0.0129386	Asphd1	7	126945567	126949582	-	1672	protein_coding	aspartate beta-hydroxylase domain containing 1 [Source:MGI Symbol;Acc:MGI:2685014]	-
ENSMUSG0000009716	203.4267626	125.3387725	246.8920584	93.02375724	95.80189322	97.30254338	191.8858645	95.37606462	1.008208136	0.0016644	0.0129599	2310010J17Rik	7	90124822	90129984	-	603	bidirectional_prom	RIKEN cDNA 2310010J17 gene [Source:MGI Symbol;Acc:MGI:1925579]	-
ENSMUSG0000004164	96.20181875	122.1789715	119.535974	204.1354673	201.4802703	176.1176035	112.6389214	193.9111137	-0.785035706	0.0016663	0.0129695	Kif21b	1	136131389	136177998	+	12184	protein_coding	kinesin family member 21B [Source:MGI Symbol;Acc:MGI:109234]	-
ENSMUSG0000011187	85.17869368	70.56888874	61.44372494	32.73058125	19.75296767	35.02891562	72.39710245	29.17082151	1.310210429	0.0016703	0.0129952	Gm6477	10	39198538	39199301	+	764	processed_pseudog	predicted gene 6477 [Source:MGI Symbol;Acc:MGI:3647945]	-
ENSMUSG0000005260	283.5949448	173.7890544	300.5156729	135.2289804	106.6660254	150.8189422	252.633224	130.9046494	0.948006136	0.0016754	0.0130291	Isoc2b	7	4844959	4870258	-	1373	protein_coding	isochorismatase domain containing 2b [Source:MGI Symbol;Acc:MGI:1914691]	-
ENSMUSG0000002159	1425.991542	1539.876348	1403.151246	1087.861161	1081.47498	1051.840494	1456.339712	1073.725545	0.439707895	0.0016872	0.0131157	Nsun2	13	69533746	69635780	+	5737	protein_coding	NOL1/NOP2/Sun domain family member 2 [Source:MGI Symbol;Acc:MGI:107252]	-
ENSMUSG0000005091	335.7042633	428.6796674	351.9049701	249.7860148	228.1467766	235.472155	372.0963003	237.8016488	0.645441453	0.0016948	0.0131692	Cdr2l	11	115381916	115396132	+	3700	protein_coding	cerebellar degeneration-related protein 2-like [Source:MGI Symbol;Acc:MGI:2684867]	-
ENSMUSG0000005172	199.4183534	169.5759864	255.8293275	114.5570344	119.5054544	117.7360775	208.2745558	117.2661888	0.828135537	0.0016988	0.013195	Wdcp	12	4843303	4860043	+	5891	protein_coding	WD repeat and coiled coil containing [Source:MGI Symbol;Acc:MGI:3040699]	-
ENSMUSG0000006301	7929.635331	10317.80349	7474.908428	4823.454079	6199.468905	5914.048587	8574.115751	5645.65719	0.602911916	0.0017002	0.0131998	Ccni	5	93181933	93206495	-	3303	protein_coding	cyclin I [Source:MGI Symbol;Acc:MGI:1341077]	-
ENSMUSG0000003725	207.4351717	155.8835154	167.5737953	261.84465	303.2080538	309.4220879	176.9641608	291.4915973	-0.718605389	0.0017065	0.0132433	Pomk	8	25980604	25994133	-	3613	protein_coding	protein-O-mannose kinase [Source:MGI Symbol;Acc:MGI:1921903]	-
ENSMUSG0000002194	576.2088102	800.482917	603.265663	1053.407918	994.5619224	962.322154	659.9857968	1003.430665	-0.604565535	0.0017107	0.0132706	Prkcd	14	30595354	30626210	-	5544	protein_coding	protein kinase C, delta [Source:MGI Symbol;Acc:MGI:97598]	-
ENSMUSG0000003656	65.13664811	47.39701482	78.20110447	130.922325	129.3819383	118.7091029	63.5782558	126.3377887	-0.992199237	0.0017148	0.013297	Ndrg4	8	95676980	95715119	+	4893	protein_coding	N-myc downstream regulated gene 4 [Source:MGI Symbol;Acc:MGI:2384590]	-
ENSMUSG0000002067	2279.782684	2434.100028	2490.146598	2912.1604	3591.089523	3721.822284	2401.343103	3408.357403	-0.505158859	0.001725	0.0133707	Rab10	12	3247428	3309969	-	3513	protein_coding	RAB10, member RAS oncogene family [Source:MGI Symbol;Acc:MGI:105066]	-
ENSMUSG0000003661	2608.472231	694.1029504	2153.881849	3600.363938	4493.800146	4455.483461	1818.81901	4183.215848	-1.201566954	0.0017298	0.0133988	Eepd1	9	25481547	25604110	+	2935	protein_coding	endonuclease/exonuclease/phosphatase family domain containing 1 [Source:MGI Symbol;Acc:MGI:1914734]	-
ENSMUSG0000002685	10593.22319	10557.94837	5605.902032	4204.157029	5501.201497	5410.021412	8919.024529	5038.459979	0.823976629	0.0017305	0.0133988	Crat	2	30400471	30415813	-	5388	protein_coding	carnitine acetyltransferase [Source:MGI Symbol;Acc:MGI:109501]	-
ENSMUSG0000002976	830.742789	591.9360518	445.7462955	335.9191234	401.9728922	298.7188082	622.8083787	345.5369412	0.85060759	0.0017308	0.0133988	Plxna4	6	32144268	32588192	-	14103	protein_coding	plexin A4 [Source:MGI Symbol;Acc:MGI:2179061]	-
ENSMUSG0000007901	48.10090937	86.36789368	50.27213858	24.97860148	20.74061606	22.37958498	61.58031388	22.69960084	1.438199267	0.0017334	0.0134136	Insl3	8	71689214	71690575	+	623	protein_coding	insulin-like 3 [Source:MGI Symbol;Acc:MGI:108427]	-
ENSMUSG0000000268	746.5661976	838.4005289	628.9603116	928.5149103	1306.658812	1275.636344	737.9756794	1170.270022	-0.664680209	0.001736	0.0134276	Prkd1	12	50341231	50649098	-	3653	protein_coding	protein kinase D1 [Source:MGI Symbol;Acc:MGI:99879]	-
ENSMUSG0000005755	1285.697223	1147.007759	1400.916929	894.0616668	912.5871066	922.4281112	1277.87397	909.6922949	0.490228721	0.0017383	0.0134398	Lgals8	13	12439415	12464944	-	3688	protein_coding	lectin, galactose binding, soluble 8 [Source:MGI Symbol;Acc:MGI:1928481]	-
ENSMUSG0000004314	599.2571626	426.5731334	701.5756229	319.5538328	384.1952213	318.1793168	575.801973	340.6427903	0.757367094	0.0017425	0.0134571	Tmem186	16	8633229	8637712	-	3391	protein_coding	transmembrane protein 186 [Source:MGI Symbol;Acc:MGI:1913940]	-
ENSMUSG0000007478	116.2438643	69.51562174	119.535974	177.4342036	188.6408413	222.8228243	101.7651533	196.2992898	-0.947671853	0.0017428	0.0134571	Plxnc1	10	94790866	94944835	-	8349	protein_coding	plexin C1 [Source:MGI Symbol;Acc:MGI:1890127]	-
ENSMUSG0000003830	100.2102279	88.47442767	89.37269082	166.2368995	144.196664	179.0366798	92.68578212	163.1567478	-0.81543409	0.0017429	0.0134571	Spats2l	1	57774162	57948394	+	8041	protein_coding	spermatogenesis associated, serine-rich 2-like [Source:MGI Symbol;Acc:MGI:1914448]	-
ENSMUSG0000003965	455.9565368	424.4665994	281.5239761	544.3612461	664.6873623	877.6689413	387.3157041	695.5725165	-0.843754353	0.0017434	0.0134571	Cpeb3	19	37021291	37208601	-	7414	protein_coding	cytoplasmic polyadenylation element binding protein 3 [Source:MGI Symbol;Acc:MGI:2443075]	-
ENSMUSG0000004244	852.7890391	1139.63489	857.9778318	1285.10598	1607.891569	1455.646049	950.1339202	1449.547866	-0.609208186	0.0017465	0.0134758	Mindy2	9	70599014	70657174	-	8600	protein_coding	MINDY lysine 48 deubiquitinase 2 [Source:MGI Symbol;Acc:MGI:2443086]	-
ENSMUSG0000001087	60.12613672	58.98295178	61.44372494	21.53327714	28.64180313	25.29866128	60.18427115	25.15791385	1.261035101	0.0017493	0.0134918	2810454H06Ri	6	134897961	134901108	+	3148	antisense	RIKEN cDNA 2810454H06 gene [Source:MGI Symbol;Acc:MGI:1920063]	-
ENSMUSG0000010674	40.08409114	41.07741285	29.04612452	4.306655428	15.80237414	3.892101735	36.73587617	8.000377101	2.209748409	0.001752	0.0135069	Gm42847	5	33990510	33993062	-	2553	TEC	predicted gene 42847 [Source:MGI Symbol;Acc:MGI:5662984]	-
ENSMUSG0000002905	553.1604578	701.4758194	569.750904	375.5403533	430.6146953	402.8325296	608.1290604	402.9958594	0.593985473	0.0017585	0.0135516	Pank4	4	154964123	154980938	+	3487	protein_coding	pantothenate kinase 4 [Source:MGI Symbol;Acc:MGI:2387466]	-
ENSMUSG0000010581	32.06727292	12.63920395	31.28044179	3.445324342	4.938241919	5.838152603	25.32897289	4.740572955	2.423915522	0.0017599	0.0135571	Mir703	5	98475556	98475664	-	109	miRNA	microRNA 703 [Source:MGI Symbol;Acc:MGI:3629657]	-
ENSMUSG0000008538	337.7084679	290.7016909	496.018434	223.0847512	217.2826444	194.6050868	374.809531	211.6574941	0.823501749	0.0017678	0.0136121	Snhg17	2	158353700	158361580	-	7052	processed_transcrip	small nucleolar RNA host gene 17 [Source:MGI Symbol;Acc:MGI:1915358]	-
ENSMUSG0000001773	945.984551	845.7733979	592.0940767	349.7004207	589.6260851	339.5858764	794.6173418	426.3041274	0.899125449	0.0017742	0.0136559	Mmp9	2	164940780	164955850	+	3539	protein_coding	matrix metallopeptidase 9 [Source:MGI Symbol;Acc:MGI:97011]	-
ENSMUSG0000001842	1987.168818	1694.706597	1493.641095	1131.789046	1287.893492	1102.437816	1725.17217	1174.040118	0.555556716	0.001778	0.0136794	Akap1	11	88830792	88864586	-	4390	protein_coding	A kinase (PRKA) anchor protein 1 [Source:MGI Symbol;Acc:MGI:104729]	-
ENSMUSG0000003391	2474.190526	5110.451465	3903.352271	2104.231842	2398.997924	2030.70408	3829.331421	2177.977949	0.814076705	0.0017795	0.013685	Gde1	7	118688545	118705778	-	1692	protein_coding	glycerophosphodiester phosphodiesterase 1 [Source:MGI Symbol;Acc:MGI:1891827]	-
ENSMUSG0000000939	233.4898309	448.6917403	450.21493	243.7566972	159.9990382	143.0347388	377.4655004	182.2634914	1.04854505	0.001784	0.0137146	Syn2	6	115134902	115282006	+	7031	protein_coding	synapsin II [Source:MGI Symbol;Acc:MGI:103020]	-
ENSMUSG0000003836	242.5087514	205.3870642	194.3856025	332.473799	314.072186	364.8845377	214.0938061	337.1435076	-0.654223389	0.0017915	0.0137666	Fbxo25	8	13907803	13940522	+	2977	protein_coding	F-box protein 25 [Source:MGI Symbol;Acc:MGI:1914072]	-
ENSMUSG0000005955	228.4793195	165.3629184	424.5202814	129.1996628	137.2831253	95.35649251	272.7875064	120.6130936	1.176391747	0.0017926	0.0137693	Ccdc28a	10	18213676	18234998	-	2964	protein_coding	coiled-coil domain containing 28A [Source:MGI Symbol;Acc:MGI:2443508]	-
ENSMUSG0000002981	2886.054562	4027.692993	4632.85686	2833.779272	2137.271102	2063.786945	3848.868139	2344.945773	0.714660581	0.0018007	0.0138224	Tmem176b	6	48833818	48841496	-	2371	protein_coding	transmembrane protein 176B [Source:MGI Symbol;Acc:MGI:1916348]	-
ENSMUSG0000004108	384.807275	360.2173127	583.1568076	888.8936803	700.2427041	681.1178036	442.7271317	756.751396	-0.774352372	0.001801	0.0138224	Ostc	3	130695912	130709444	-	2751	protein_coding	oligosaccharyltransferase complex subunit (non-catalytic) [Source:MGI Symbol;Acc:MGI:1913607]	-
ENSMUSG0000003119	1391.920065	1223.896249	1263.506416	944.0188698	957.0312838	926.320213	1293.107577	942.4567889	0.456475615	0.0018026	0.0138291	Fundc2	X	75382399	75396459	+	2785	protein_coding	FUN14 domain containing 2 [Source:MGI Symbol;Acc:MGI:1914641]	-
ENSMUSG0000008173	111.2333529	115.8593696	82.66973901	62.87716925	36.5429902	33.08286475	103.2541538	44.16767473	1.221179206	0.0018092	0.0138736	Hmgb1-ps2	X	146175942	146176577	+	636	processed_pseudog	high mobility group box 1, pseudogene 2 [Source:MGI Symbol;Acc:MGI:3768537]	-
ENSMUSG0000003585	1643.447737	1798.980029	2152.76469	1254.959392	1378.757144	1234.769275	1865.064152	1289.49527	0.532292979	0.0018121	0.0138901	Ythdc1	5	86804221	86836659	+	13651	protein_coding	YTH domain containing 1 [Source:MGI Symbol;Acc:MGI:2443713]	-
ENSMUSG0000007861	1817.813533	1665.215121	1758.407692	1363.487108	1164.437444	1276.609369	1747.145449	1268.177974	0.462042294	0.0018209	0.0139521	Smarcd2	11	106263179	106272972	-	3589	protein_coding	SWI/SNF related, matrix associated, actin dependent regulator of chromatin, subfamily d, member 2 [Source:MGI Symbol;Acc:MG	-
ENSMUSG0000009688	0	2.106533992	1.117158635	18.0879528	14.81472576	11.67630521	1.074564209	14.85966125	-3.807253182	0.0018254	0.0139811	Shisa8	15	82206168	82212815	-	2776	protein_coding	shisa family member 8 [Source:MGI Symbol;Acc:MGI:2146080]	-
ENSMUSG0000009383	4.008409114	6.319601977	16.75737953	0	0	0	9.02846354	0	5.70505626	0.0018296	0.0140071	Gm23144	15	88835300	88835406	+	107	snRNA	predicted gene, 23144 [Source:MGI Symbol;Acc:MGI:5452921]	-
ENSMUSG0000010927	44.09250026	63.19601977	74.84962856	18.0879528	25.67885798	25.29866128	60.71271619	23.02182402	1.400732929	0.0018373	0.0140607	Gm45220	7	90201278	90203786	+	2509	TEC	predicted gene 45220 [Source:MGI Symbol;Acc:MGI:5753796]	-
ENSMUSG0000002209	299.6285813	335.9921718	289.3440865	398.7962926	515.5524563	541.9751666	308.3216132	485.4413052	-0.654075407	0.0018403	0.0140775	Dmtn	14	70601263	70636048	-	9804	protein_coding	dematin actin binding protein [Source:MGI Symbol;Acc:MGI:99670]	-
ENSMUSG0000002959	33.06937519	36.86434486	27.92896588	72.35181119	63.20949656	88.54531447	32.62089531	74.70220741	-1.194024879	0.0018424	0.014088	Sdsl	5	120458186	120472810	-	1497	protein_coding	serine dehydratase-like [Source:MGI Symbol;Acc:MGI:2182607]	-
ENSMUSG0000010675	47.0988071	24.22514091	20.10885543	77.5197977	72.09833201	90.49136534	30.47760115	80.03649835	-1.387762916	0.0018471	0.0141181	Tpi-rs11	6	127183409	127183718	-	310	processed_pseudog	triosephosphate isomerase related sequence 11 [Source:MGI Symbol;Acc:MGI:98799]	-
ENSMUSG0000005977	312.6559109	330.7258368	235.720472	198.1061497	152.0978511	165.4143237	293.0340732	171.8727748	0.769161613	0.0018537	0.014163	Slx1b	7	126689468	126695784	-	3521	protein_coding	SLX1 structure-specific endonuclease subunit homolog B (S. cerevisiae) [Source:MGI Symbol;Acc:MGI:1915220]	-
ENSMUSG0000001986	786.6502887	489.7691532	573.1023799	354.0070762	393.0840567	377.5338683	616.5072739	374.8750004	0.71831985	0.0018646	0.0142346	Crybg1	10	43950636	44148853	-	7525	protein_coding	crystallin beta-gamma domain containing 1 [Source:MGI Symbol;Acc:MGI:109544]	-
ENSMUSG0000004714	13.02732962	21.06533992	6.702951811	66.32249359	30.6170999	52.54337342	13.59854045	49.82765564	-1.87165959	0.0018646	0.0142346	Tet1	10	62804570	62908996	-	15457	protein_coding	tet methylcytosine dioxygenase 1 [Source:MGI Symbol;Acc:MGI:1098693]	-
ENSMUSG0000003035	221.4646036	302.2876279	317.2730524	174.8502104	166.9125769	158.6031457	280.3417613	166.7886443	0.747990227	0.0018663	0.0142413	Tead4	6	128224288	128300823	-	3598	protein_coding	TEA domain family member 4 [Source:MGI Symbol;Acc:MGI:106907]	TEA
ENSMUSG0000008440	62.13034127	64.24928676	59.20940767	17.22662171	25.67885798	31.13681388	61.8630119	24.68076452	1.330652434	0.0018753	0.0143048	Gm14018	2	121485432	121491512	+	455	unprocessed_pseud	predicted gene 14018 [Source:MGI Symbol;Acc:MGI:3649456]	-
ENSMUSG0000003176	589.2361398	760.4587712	704.9270988	882.0030316	1072.586145	1217.254818	684.8740033	1057.281331	-0.626368402	0.0018784	0.0143225	Nudt7	8	114133557	114154739	+	5618	protein_coding	nudix (nucleoside diphosphate linked moiety X)-type motif 7 [Source:MGI Symbol;Acc:MGI:1914778]	-
ENSMUSG0000001598	66.13875039	57.92968479	130.7075603	167.9595617	205.4308638	182.9287816	84.92533183	185.4397357	-1.128961214	0.0018839	0.0143585	Lrrc27	7	139212988	139242979	+	3637	protein_coding	leucine rich repeat containing 27 [Source:MGI Symbol;Acc:MGI:1923862]	-
ENSMUSG0000004024	19559.03227	38071.38884	21758.89874	12888.09703	15923.85489	16137.62682	26463.10662	14983.19291	0.820646609	0.0018872	0.0143779	Lrp1	10	127538161	127621148	-	17717	protein_coding	low density lipoprotein receptor-related protein 1 [Source:MGI Symbol;Acc:MGI:96828]	-
ENSMUSG0000002845	12.02522734	7.372868973	7.820110447	56.84785165	22.71591283	31.13681388	9.072735588	36.90019279	-2.023470414	0.0018884	0.0143809	Atp8b5	4	43267159	43373833	+	6473	protein_coding	ATPase, class I, type 8B, member 5 [Source:MGI Symbol;Acc:MGI:2444287]	-
ENSMUSG0000003022	155.3258532	78.99502471	87.13837355	185.1861834	237.0356121	233.5261041	107.1530838	218.5826332	-1.026046332	0.0018928	0.0144088	Ptpro	6	137252319	137463233	+	6723	protein_coding	protein tyrosine phosphatase, receptor type, O [Source:MGI Symbol;Acc:MGI:1097152]	-
ENSMUSG0000003746	633.3286401	591.9360518	640.131898	838.0751463	875.056468	865.0196106	621.7988633	859.3837416	-0.466790824	0.0018936	0.0144088	Ints7	1	191575636	191623688	+	7319	protein_coding	integrator complex subunit 7 [Source:MGI Symbol;Acc:MGI:1924315]	-
ENSMUSG0000003341	1354.842281	2836.44802	1360.699218	965.5521469	1059.746716	898.1024754	1850.663173	974.4671127	0.925387065	0.0019018	0.0144651	Gucd1	10	75351111	75517972	-	8206	protein_coding	guanylyl cyclase domain containing 1 [Source:MGI Symbol;Acc:MGI:1916028]	-
ENSMUSG0000004203	92.19340963	104.2734326	71.49815265	189.4928388	137.2831253	177.0906289	89.32166497	167.955531	-0.910645301	0.001904	0.014476	Igsf3	3	101377083	101463059	+	8479	protein_coding	immunoglobulin superfamily, member 3 [Source:MGI Symbol;Acc:MGI:1926158]	-
ENSMUSG0000003919	268.5634107	412.8806625	360.8422392	529.7186176	572.8360626	567.2738279	347.4287708	556.6095027	-0.680354334	0.0019057	0.0144829	1110008P14Ri	2	32377097	32381938	-	2694	protein_coding	RIKEN cDNA 1110008P14 gene [Source:MGI Symbol;Acc:MGI:1920987]	-
ENSMUSG0000002923	1797.771488	1141.741424	1034.488896	2189.50362	2523.44162	1992.756088	1324.667269	2235.233776	-0.7545009	0.0019084	0.014498	Pdgfra	5	75152292	75198215	+	10024	protein_coding	platelet derived growth factor receptor, alpha polypeptide [Source:MGI Symbol;Acc:MGI:97530]	-
ENSMUSG0000005276	43.09039798	75.83522372	43.56918677	12.0586352	20.74061606	21.40655954	54.16493616	18.0686036	1.589151171	0.0019095	0.0145001	Gm9889	3	115715150	115716728	+	1579	lincRNA	predicted gene 9889 [Source:MGI Symbol;Acc:MGI:3642568]	-
ENSMUSG0000006234	0	0	0	5.167986514	23.70356121	2.919076301	0	10.59687467	-5.766754765	0.0019139	0.0145276	Serpinb2	1	107511423	107535478	+	2349	protein_coding	serine (or cysteine) peptidase inhibitor, clade B, member 2 [Source:MGI Symbol;Acc:MGI:97609]	-
ENSMUSG0000002946	894.8773348	680.4104795	866.9151009	1143.847682	1199.992786	1221.146919	814.0676384	1188.329129	-0.545609279	0.0019159	0.014532	Vps29	5	122354369	122364984	+	6148	protein_coding	VPS29 retromer complex component [Source:MGI Symbol;Acc:MGI:1928344]	-
ENSMUSG0000005858	335.7042633	351.7911767	242.4234238	179.1568658	203.4555671	169.3064255	309.9729546	183.9729528	0.753592951	0.001916	0.014532	Serhl	15	83089506	83116674	+	7393	protein_coding	serine hydrolase-like [Source:MGI Symbol;Acc:MGI:1890404]	-
ENSMUSG0000004533	22.04625013	16.85227194	32.39760042	70.62914902	51.35771595	65.19270406	23.76537416	62.39318968	-1.396909168	0.0019168	0.014532	Cenpe	3	135212537	135273611	+	8620	protein_coding	centromere protein E [Source:MGI Symbol;Acc:MGI:1098230]	-
ENSMUSG0000010193	340.7147747	251.7308121	292.6955624	403.1029481	576.7866561	475.8094371	295.0470497	485.2330118	-0.716810228	0.0019191	0.0145433	Gm28438	1	24612775	24613119	-	345	unprocessed_pseud	predicted gene 28438 [Source:MGI Symbol;Acc:MGI:5579144]	-
ENSMUSG0000004932	1128.367166	1669.428189	597.6798698	1808.79528	2586.651117	2575.598323	1131.825075	2323.681573	-1.037536471	0.0019267	0.014595	Kmt5a	5	124439930	124462308	+	5307	protein_coding	lysine methyltransferase 5A [Source:MGI Symbol;Acc:MGI:1915206]	-
ENSMUSG0000003422	280.588638	149.5639134	210.0258234	434.1108671	319.9980763	430.0772417	213.3927916	394.7287284	-0.887001841	0.0019288	0.0146057	Gpc1	1	92831645	92860779	+	5159	protein_coding	glypican 1 [Source:MGI Symbol;Acc:MGI:1194891]	-
ENSMUSG0000008669	12.02522734	13.69247095	23.46033134	1.722662171	2.962945151	0.973025434	16.3926765	1.886210919	3.121969124	0.0019323	0.0146261	A730081D07R	13	40956416	41001151	-	1804	antisense	RIKEN cDNA A730081D07 gene [Source:MGI Symbol;Acc:MGI:2441825]	-
ENSMUSG0000003008	1046.194779	1110.143414	489.3154822	1327.311203	1857.76661	1990.810038	881.8845583	1725.29595	-0.967782387	0.0019442	0.0147099	Plxna1	6	89316314	89362620	-	9054	protein_coding	plexin A1 [Source:MGI Symbol;Acc:MGI:107685]	-
ENSMUSG0000005865	251.5276719	293.8614919	185.4483334	127.4770007	154.0731479	128.4393573	243.6124991	136.6631686	0.835231127	0.001945	0.0147099	Samd12	15	53453810	53902537	-	9283	protein_coding	sterile alpha motif domain containing 12 [Source:MGI Symbol;Acc:MGI:2444518]	-
ENSMUSG0000005128	3974.337637	4243.612727	3295.617974	2024.989382	2765.415474	2778.960639	3837.856113	2523.121832	0.605302262	0.0019496	0.0147387	Pcmtd1	1	7088920	7173628	+	6654	protein_coding	protein-L-isoaspartate (D-aspartate) O-methyltransferase domain containing 1 [Source:MGI Symbol;Acc:MGI:2441773]	-
ENSMUSG0000002466	218.4582967	216.9730012	153.050733	747.6353823	319.0104279	278.2852741	196.160677	448.3103614	-1.192816942	0.0019715	0.0148988	Fads2	19	10064164	10101503	-	1508	protein_coding	fatty acid desaturase 2 [Source:MGI Symbol;Acc:MGI:1930079]	-
ENSMUSG0000002314	314.6601155	238.0383411	223.431727	394.4896372	428.6393985	413.5358094	258.7100612	412.221615	-0.670837814	0.0019878	0.0150153	Wrb	16	96145407	96157852	+	4368	protein_coding	tryptophan rich basic protein [Source:MGI Symbol;Acc:MGI:2136882]	-
ENSMUSG0000004861	145.3048304	147.4573795	337.3819078	60.29317599	102.7154319	97.30254338	210.0480392	86.77038176	1.276032541	0.0019895	0.0150221	Nog	11	89300638	89302332	-	1695	protein_coding	noggin [Source:MGI Symbol;Acc:MGI:104327]	-
ENSMUSG0000002952	116.2438643	114.8061026	63.67804221	193.7994943	211.3567541	172.2255018	98.2426697	192.4605834	-0.968009738	0.0019913	0.0150298	Chek2	5	110839979	110874145	+	3001	protein_coding	checkpoint kinase 2 [Source:MGI Symbol;Acc:MGI:1355321]	-
ENSMUSG0000002430	150.3153418	87.42116068	71.49815265	178.2955347	290.3686248	201.4612648	103.0782184	223.3601414	-1.112911324	0.0019923	0.0150316	Dtna	18	23415135	23659715	+	16930	protein_coding	dystrobrevin alpha [Source:MGI Symbol;Acc:MGI:106039]	-
ENSMUSG0000003481	4496.432924	2852.247025	4546.835645	4874.272613	8041.43314	7940.860565	3965.171865	6952.188773	-0.810040477	0.0020063	0.0151273	Scn7a	2	66673425	66784914	-	7338	protein_coding	sodium channel, voltage-gated, type VII, alpha [Source:MGI Symbol;Acc:MGI:102965]	-
ENSMUSG0000005139	16.03363646	11.58593696	12.28874499	1.722662171	0	0	13.3027728	0.574220724	4.450402814	0.0020066	0.0151273	Tacstd2	6	67534062	67535796	-	1735	protein_coding	tumor-associated calcium signal transducer 2 [Source:MGI Symbol;Acc:MGI:1861606]	-
ENSMUSG0000005157	562.1793783	477.1299492	700.4584643	1105.949114	772.3410361	948.6997979	579.9225973	942.3299826	-0.701006142	0.0020087	0.0151372	Tceal8	X	136168984	136172342	-	2563	protein_coding	transcription elongation factor A (SII)-like 8 [Source:MGI Symbol;Acc:MGI:1913934]	-
ENSMUSG0000002906	759.5935272	532.9531	770.8394583	989.6694173	1018.265484	1298.988954	687.7953618	1102.307952	-0.680392674	0.0020135	0.0151621	Mrpl20	4	155802878	155809975	+	1664	protein_coding	mitochondrial ribosomal protein L20 [Source:MGI Symbol;Acc:MGI:2137221]	-
ENSMUSG0000002527	2871.023028	2947.041055	2508.021136	1801.0433	2218.25827	1833.179917	2775.36174	1950.827162	0.50879304	0.002014	0.0151621	Flnb	14	7817957	7951588	+	9198	protein_coding	filamin, beta [Source:MGI Symbol;Acc:MGI:2446089]	-
ENSMUSG0000005983	212.4456831	244.3579431	269.2352311	94.74641942	152.0978511	151.7919677	242.0129524	132.8787461	0.866634619	0.0020145	0.0151621	Zfp874b	13	67471513	67484258	-	3789	protein_coding	zinc finger protein 874b [Source:MGI Symbol;Acc:MGI:3040702]	zf-C2
ENSMUSG0000010487	6.012613672	5.26633498	4.468634541	31.86925017	40.49358373	11.67630521	5.249194398	28.01304637	-2.413414882	0.0020161	0.0151683	Trdc	14	54142851	54148868	+	3865	TR_C_gene	T cell receptor delta, constant region [Source:MGI Symbol;Acc:MGI:98612]	-
ENSMUSG0000003497	1150.413416	1532.503479	1603.122642	670.9769157	995.5495708	967.1872812	1428.679846	877.9045892	0.702799066	0.0020217	0.0152043	Dopey1	9	86467154	86555923	+	20476	protein_coding	dopey family member 1 [Source:MGI Symbol;Acc:MGI:1289294]	-
ENSMUSG0000006882	10504.03608	9302.45411	10540.39172	5962.133774	7530.818926	7778.365318	10115.62731	7090.439339	0.512700616	0.0020299	0.0152596	Csde1	3	103020426	103058186	+	10902	protein_coding	cold shock domain containing E1, RNA binding [Source:MGI Symbol;Acc:MGI:92356]	CSD
ENSMUSG0000001691	1595.346828	1316.583745	1550.616186	805.344565	1157.523906	967.1872812	1487.515586	976.6852507	0.607342599	0.002036	0.0152995	Sulf1	1	12692277	12861192	+	11294	protein_coding	sulfatase 1 [Source:MGI Symbol;Acc:MGI:2138563]	-
ENSMUSG0000004873	204.4288648	238.0383411	283.7582933	134.3676494	163.9496317	113.8439758	242.0751664	137.3870856	0.816800522	0.0020407	0.0153235	Ggnbp1	17	26973217	27036378	+	6943	protein_coding	gametogenetin binding protein 1 [Source:MGI Symbol;Acc:MGI:3055306]	-
ENSMUSG0000004358	38.07988659	30.54474289	21.22601407	112.8343722	74.07362878	53.51639886	29.95021451	80.14146662	-1.418813783	0.0020408	0.0153235	Pxylp1	9	96823336	96892669	-	6148	protein_coding	2-phosphoxylose phosphatase 1 [Source:MGI Symbol;Acc:MGI:2442444]	-
ENSMUSG0000003908	1159.432336	983.7513744	712.7472093	1683.902272	1341.226505	1573.382126	951.9769733	1532.836968	-0.686937931	0.0020442	0.0153332	Zfp503	14	21983959	21989601	-	4216	protein_coding	zinc finger protein 503 [Source:MGI Symbol;Acc:MGI:1353644]	-
ENSMUSG0000008128	102.2144324	46.34374783	87.13837355	29.28525691	24.69120959	36.00194105	78.56551793	29.99280252	1.389672208	0.0020443	0.0153332	Gm6274	X	36668217	36669013	+	797	processed_pseudog	predicted gene 6274 [Source:MGI Symbol;Acc:MGI:3645367]	-
ENSMUSG0000005477	424.8913661	463.4374783	548.5248899	299.7432178	319.0104279	328.8825966	478.9512448	315.8787475	0.600269775	0.0020446	0.0153332	Kctd18	1	57955101	58018956	-	12973	protein_coding	potassium channel tetramerisation domain containing 18 [Source:MGI Symbol;Acc:MGI:3603813]	-
ENSMUSG0000001188	1286.699326	926.8749566	1111.572842	1550.395954	1562.459743	1934.374562	1108.382375	1682.410087	-0.601866458	0.0020518	0.0153813	Gltp	5	114669398	114690984	-	1762	protein_coding	glycolipid transfer protein [Source:MGI Symbol;Acc:MGI:1929253]	-
ENSMUSG0000002767	1.002102279	1.053266996	0	18.0879528	13.82707737	8.757228904	0.685123092	13.55741969	-4.280736794	0.002054	0.0153911	Pex5l	3	32949408	33143247	-	8846	protein_coding	peroxisomal biogenesis factor 5-like [Source:MGI Symbol;Acc:MGI:1916672]	-
ENSMUSG0000003198	3750.868829	3248.275416	2607.448255	4236.026279	4900.71128	5388.614852	3202.1975	4841.784137	-0.596309905	0.0020583	0.0154171	Gnpat	8	124863033	124890057	+	3587	protein_coding	glyceronephosphate O-acyltransferase [Source:MGI Symbol;Acc:MGI:1343460]	-
ENSMUSG0000003110	129.2711939	103.2201656	79.3182631	199.8288119	198.5173251	172.2255018	103.9365409	190.1905463	-0.869853967	0.0020592	0.015418	Sash3	X	48146436	48161565	+	2677	protein_coding	SAM and SH3 domain containing 3 [Source:MGI Symbol;Acc:MGI:1921381]	-
ENSMUSG0000003373	32.06727292	33.70454388	7.820110447	43.92788537	130.3695867	95.35649251	24.53064241	89.88465484	-1.868020308	0.0020678	0.0154759	Egr3	14	70077203	70082614	+	4406	protein_coding	early growth response 3 [Source:MGI Symbol;Acc:MGI:1306780]	zf-C2
ENSMUSG0000002801	122.256478	108.4865006	112.8330222	56.84785165	49.38241919	71.03085667	114.5253336	59.0870425	0.955249052	0.0020697	0.0154785	Egf	3	129677565	129755316	-	11275	protein_coding	epidermal growth factor [Source:MGI Symbol;Acc:MGI:95290]	-
ENSMUSG0000002913	1632.424612	2528.894058	5332.198166	1003.450715	1676.039307	1522.784804	3164.505612	1400.758275	1.175784575	0.0020698	0.0154785	Fosl2	5	32135801	32157842	+	9680	protein_coding	fos-like antigen 2 [Source:MGI Symbol;Acc:MGI:102858]	TF_b
ENSMUSG0000002585	253.5318765	279.115754	223.431727	416.8842454	366.4175504	375.5878174	252.0264525	386.2965377	-0.616080791	0.0020819	0.0155627	Dnaaf5	5	139150223	139186510	+	3514	protein_coding	dynein, axonemal assembly factor 5 [Source:MGI Symbol;Acc:MGI:3616079]	-
ENSMUSG0000003785	1070.245234	1262.867128	801.0027415	2906.131083	1363.942418	1694.03728	1044.705034	1988.036927	-0.928344444	0.0020921	0.0156334	Cpe	8	64592542	64693054	-	2609	protein_coding	carboxypeptidase E [Source:MGI Symbol;Acc:MGI:101932]	-
ENSMUSG0000002759	685.4379586	644.5994016	660.2407534	871.6670586	904.6859195	991.512917	663.4260379	922.621965	-0.475538985	0.002093	0.0156338	Armc1	3	19131402	19163065	-	3673	protein_coding	armadillo repeat containing 1 [Source:MGI Symbol;Acc:MGI:1921502]	-
ENSMUSG0000005021	279.5865357	271.742885	186.5654921	585.7051382	313.0845376	486.5127169	245.9649709	461.7674642	-0.908615629	0.0020981	0.0156651	Eva1b	4	126147744	126149875	+	1942	protein_coding	eva-1 homolog B (C. elegans) [Source:MGI Symbol;Acc:MGI:1922063]	-
ENSMUSG0000003210	345.7252861	295.9680259	216.7287752	467.7027795	452.3429598	482.6206152	286.1406958	467.5554515	-0.70730068	0.0020989	0.0156651	Nlrx1	9	44252717	44268599	-	5750	protein_coding	NLR family member X1 [Source:MGI Symbol;Acc:MGI:2429611]	-
ENSMUSG0000003012	10.02102279	9.479402965	3.351475906	118.8636898	19.75296767	14.59538151	7.617300552	51.07067966	-2.742893943	0.0021	0.0156674	Lrrc23	6	124769863	124779727	-	1734	protein_coding	leucine rich repeat containing 23 [Source:MGI Symbol;Acc:MGI:1315192]	-
ENSMUSG0000002396	1219.558473	1415.590843	1386.393866	914.7336129	992.5866257	995.4050188	1340.514394	967.5750858	0.470385751	0.0021041	0.0156917	Enpp4	17	44096308	44105809	-	4560	protein_coding	ectonucleotide pyrophosphatase/phosphodiesterase 4 [Source:MGI Symbol;Acc:MGI:2682634]	-
ENSMUSG0000004264	18.03784102	9.479402965	32.39760042	50.81853405	57.28360626	81.73413644	19.9716148	63.27875891	-1.667590942	0.0021071	0.0157073	Rgsl1	1	153779381	153844142	-	5219	protein_coding	regulator of G-protein signaling like 1 [Source:MGI Symbol;Acc:MGI:2685048]	-
ENSMUSG0000002438	526.1036963	609.8415907	421.1688055	903.5363088	739.7486394	779.3933725	519.0380308	807.5594402	-0.63763698	0.0021117	0.0157353	Bin1	18	32377177	32435736	+	2451	protein_coding	bridging integrator 1 [Source:MGI Symbol;Acc:MGI:108092]	-
ENSMUSG0000002097	1249.621541	1231.269118	1324.950141	968.9974713	931.3524259	914.6439078	1268.6136	938.3312683	0.434855068	0.0021139	0.0157456	Klhdc2	12	69296681	69310687	+	5438	protein_coding	kelch domain containing 2 [Source:MGI Symbol;Acc:MGI:1916804]	-
ENSMUSG0000002137	1831.842965	2316.134124	1527.155854	2501.305473	3147.635399	3228.498389	1891.710981	2959.14642	-0.645310938	0.0021176	0.0157667	Mcur1	13	43538393	43560191	-	7076	protein_coding	mitochondrial calcium uniporter regulator 1 [Source:MGI Symbol;Acc:MGI:1923387]	-
ENSMUSG0000007346	14.0294319	14.74573795	24.57748997	59.43184491	45.43182565	45.73219539	17.78421994	50.19862198	-1.503366062	0.0021218	0.0157858	Pnldc1	17	12888729	12910230	-	2660	protein_coding	poly(A)-specific ribonuclease (PARN)-like domain containing 1 [Source:MGI Symbol;Acc:MGI:2685159]	-
ENSMUSG0000010594	19.03994329	12.63920395	35.74907633	2.583993257	1.975296767	5.838152603	22.47607452	3.465814209	2.699706357	0.0021218	0.0157858	Gm42809	3	89912916	89914053	+	1138	antisense	predicted gene 42809 [Source:MGI Symbol;Acc:MGI:5662946]	-
ENSMUSG0000003270	2908.100813	2476.230708	2257.777602	1801.904631	1899.247842	1685.280051	2547.369707	1795.477508	0.504787838	0.0021254	0.0158062	Kank1	19	25236975	25434496	+	6166	protein_coding	KN motif and ankyrin repeat domains 1 [Source:MGI Symbol;Acc:MGI:2147707]	-
ENSMUSG0000005842	154.3237509	137.9779765	176.5110644	67.18382468	103.7030803	77.8420347	156.2709306	82.90964656	0.916181349	0.0021334	0.0158595	Cxcl2	5	90903871	90905938	+	2068	protein_coding	chemokine (C-X-C motif) ligand 2 [Source:MGI Symbol;Acc:MGI:1340094]	-
ENSMUSG0000002060	3.006306836	17.90553893	25.69464861	0.861331086	0.987648384	0.973025434	15.53549813	0.940668301	4.04554613	0.0021405	0.0159055	Apob	12	7977648	8016835	+	13934	protein_coding	apolipoprotein B [Source:MGI Symbol;Acc:MGI:88052]	-
ENSMUSG0000009389	0	0	0	11.19730411	2.962945151	13.62235607	0	9.260868446	-5.575783189	0.0021437	0.0159235	Ighv1-53	12	115158403	115158835	-	351	IG_V_gene	immunoglobulin heavy variable 1-53 [Source:MGI Symbol;Acc:MGI:3576502]	-
ENSMUSG0000011125	8.016818229	28.43820889	8.937269082	1.722662171	0	0.973025434	15.1307654	0.898562535	4.038066621	0.0021446	0.0159237	Gm47818	9	77836030	77848111	+	2740	lincRNA	predicted gene, 47818 [Source:MGI Symbol;Acc:MGI:6097006]	-
ENSMUSG0000002906	901.8920508	752.0326352	908.2499704	579.6758206	629.1320204	435.9153943	854.0582188	548.2410785	0.639383526	0.0021469	0.0159341	Mib2	4	155654677	155669198	-	4724	protein_coding	mindbomb E3 ubiquitin protein ligase 2 [Source:MGI Symbol;Acc:MGI:2679684]	-
ENSMUSG0000007502	60.12613672	86.36789368	39.10055223	16.36529063	22.71591283	26.27168671	61.86486088	21.78429672	1.510274252	0.002153	0.0159685	Accsl	2	93855361	93869157	-	2801	protein_coding	1-aminocyclopropane-1-carboxylate synthase (non-functional)-like [Source:MGI Symbol;Acc:MGI:3584519]	-
ENSMUSG0000002855	3382.09519	4076.143275	3352.593064	2459.100249	2730.847781	2596.031857	3603.61051	2595.326629	0.473608348	0.0021532	0.0159685	Osbpl9	4	109061145	109202272	-	7006	protein_coding	oxysterol binding protein-like 9 [Source:MGI Symbol;Acc:MGI:1923784]	-
ENSMUSG0000003114	77.16187545	37.91761186	73.73246992	116.2796966	139.2584221	151.7919677	62.93731908	135.7766954	-1.108530844	0.0021544	0.0159714	Syp	X	7638471	7653256	+	5211	protein_coding	synaptophysin [Source:MGI Symbol;Acc:MGI:98467]	-
ENSMUSG0000001554	487.0217074	515.0475611	720.5673197	405.6869413	313.0845376	324.0174694	574.2121961	347.5963161	0.722974407	0.0021704	0.0160831	Nat9	11	115182832	115187859	-	2066	protein_coding	N-acetyltransferase 9 (GCN5-related, putative) [Source:MGI Symbol;Acc:MGI:1913426]	-
ENSMUSG0000002505	163.3426714	189.5880593	160.8708435	967.2748091	239.0109089	242.283333	171.2671914	482.8563503	-1.496023068	0.0021732	0.016098	Gk	X	85701937	85776819	-	7743	protein_coding	glycerol kinase [Source:MGI Symbol;Acc:MGI:106594]	-
ENSMUSG0000010095	47.0988071	49.50354882	37.9833936	21.53327714	8.888835454	5.838152603	44.8619165	12.08675507	1.880446775	0.0021746	0.0161014	Gm10138	1	151339604	151345231	-	2843	antisense	predicted gene 10138 [Source:MGI Symbol;Acc:MGI:3708522]	-
ENSMUSG0000005796	487.0217074	621.4275277	504.9557031	775.197977	759.5016071	827.0716187	537.8016461	787.2570676	-0.549765302	0.0021814	0.0161458	Itpk1	12	102568582	102704930	-	3320	protein_coding	inositol 1,3,4-triphosphate 5/6 kinase [Source:MGI Symbol;Acc:MGI:2446159]	-
ENSMUSG0000004647	2997.287915	2090.734987	1683.558063	1520.249366	1347.152395	1189.03708	2257.193655	1352.146281	0.739328717	0.002187	0.0161757	Sox18	2	181669836	181671640	-	1620	protein_coding	SRY (sex determining region Y)-box 18 [Source:MGI Symbol;Acc:MGI:103559]	HMG
ENSMUSG0000004971	133.2796031	129.5518405	126.2389258	201.551474	220.2455896	204.3353411	129.6901231	208.7108016	-0.68581963	0.0021872	0.0161757	Lig4	8	9969049	9977686	-	5202	protein_coding	ligase IV, DNA, ATP-dependent [Source:MGI Symbol;Acc:MGI:1335098]	-
ENSMUSG0000004274	3061.422461	992.1775103	1341.707521	899.2296534	845.4270165	748.2565586	1798.435831	830.9710761	1.113964345	0.0021916	0.0162019	Id1	2	152736251	152737410	+	1160	protein_coding	inhibitor of DNA binding 1 [Source:MGI Symbol;Acc:MGI:96396]	bHLH
ENSMUSG0000000234	114.2396598	101.1136316	146.3477812	203.2741362	217.2826444	200.4432394	120.5670242	207.0000067	-0.780977961	0.0021946	0.0162175	Armc6	8	70220172	70234466	-	3262	protein_coding	armadillo repeat containing 6 [Source:MGI Symbol;Acc:MGI:1924063]	-
ENSMUSG0000004077	615.2907991	470.8103473	631.1946289	434.1108671	296.2945151	255.9056891	572.4319251	328.7703571	0.798876235	0.002198	0.0162367	Oard1	17	48410014	48417266	+	1558	protein_coding	O-acyl-ADP-ribose deacylase 1 [Source:MGI Symbol;Acc:MGI:2146818]	-
ENSMUSG0000000803	785.6481864	847.8799319	1014.380041	1367.793764	1174.313928	1300.935005	882.636053	1281.014232	-0.537913323	0.0022011	0.0162529	Ap2s1	7	16738410	16749294	+	1472	protein_coding	adaptor-related protein complex 2, sigma 1 subunit [Source:MGI Symbol;Acc:MGI:2141861]	-
ENSMUSG0000002788	780.637675	766.7783732	888.141115	578.8144895	596.5396238	550.7323955	811.8523877	575.3621696	0.496489993	0.0022047	0.0162731	Clcc1	3	108653913	108678840	+	4559	protein_coding	chloride channel CLIC-like 1 [Source:MGI Symbol;Acc:MGI:2385186]	-
ENSMUSG0000007004	1372.880122	863.6789368	937.2960949	1646.865036	1936.778481	1540.299262	1057.951718	1707.980926	-0.690695	0.0022065	0.0162798	Fat1	8	44935447	45052257	+	15655	protein_coding	FAT atypical cadherin 1 [Source:MGI Symbol;Acc:MGI:109168]	-
ENSMUSG0000005137	1498.142907	1577.79396	1310.427079	1122.314405	1028.141967	945.7807216	1462.121315	1032.079031	0.502408518	0.0022116	0.0163109	Pcdh1	18	38185914	38212053	-	8797	protein_coding	protocadherin 1 [Source:MGI Symbol;Acc:MGI:104692]	-
ENSMUSG0000003867	706.4821064	618.2677267	740.6761752	496.9880364	479.0094661	466.0791828	688.4753361	480.6922284	0.517987503	0.0022124	0.0163109	Arfrp1	2	181357690	181365404	-	4320	protein_coding	ADP-ribosylation factor related protein 1 [Source:MGI Symbol;Acc:MGI:1923938]	-
ENSMUSG0000003035	10.02102279	8.426135969	25.69464861	0.861331086	1.975296767	0	14.71393579	0.945542618	3.964855406	0.0022148	0.0163171	Pzp	6	128483567	128526720	-	6414	protein_coding	PZP, alpha-2-macroglobulin like [Source:MGI Symbol;Acc:MGI:87854]	-
ENSMUSG0000003596	1143.3987	950.0468305	1331.653093	720.0727876	824.6864004	744.3644568	1141.699541	763.0412149	0.581358559	0.002215	0.0163171	Ints6l	X	56454857	56507843	+	7173	protein_coding	integrator complex subunit 6 like [Source:MGI Symbol;Acc:MGI:2442593]	-
ENSMUSG0000004472	49.10301165	26.3316749	36.86623496	11.19730411	7.90118707	13.62235607	37.43364051	10.90694909	1.778982638	0.0022175	0.016329	Gpr152	19	4139799	4145738	+	3922	protein_coding	G protein-coupled receptor 152 [Source:MGI Symbol;Acc:MGI:2685519]	-
ENSMUSG0000009024	40.08409114	57.92968479	93.84132536	11.19730411	25.67885798	24.32563584	63.95170043	20.40059931	1.652235296	0.0022293	0.0164095	Gm14027	2	129016952	129021756	+	622	processed_transcrip	predicted gene 14027 [Source:MGI Symbol;Acc:MGI:3757578]	-
ENSMUSG0000004736	432.9081844	568.7641779	416.7001709	689.9261996	738.760991	715.1736938	472.7908444	714.6202948	-0.595748429	0.0022331	0.0164224	Abhd17b	19	21653309	21685637	+	2475	protein_coding	abhydrolase domain containing 17B [Source:MGI Symbol;Acc:MGI:1917816]	-
ENSMUSG0000008370	1.002102279	0	3.351475906	18.94928388	9.876483837	21.40655954	1.451192728	16.74410909	-3.554474199	0.0022335	0.0164224	Gm4912	X	92093699	92094742	-	1044	unprocessed_pseud	predicted pseudogene 4912 [Source:MGI Symbol;Acc:MGI:3643140]	-
ENSMUSG0000008987	104.218637	122.1789715	235.720472	53.40252731	83.95011262	58.38152603	154.0393602	65.24472198	1.23947397	0.0022337	0.0164224	Etohd2	13	59769497	59775407	+	5225	antisense	ethanol decreased 2 [Source:MGI Symbol;Acc:MGI:1261417]	-
ENSMUSG0000002201	4573.5948	2276.109979	5919.823608	7420.367302	8443.406033	8454.617994	4256.509462	8106.130443	-0.929369893	0.0022374	0.0164434	Rgcc	14	79288756	79301645	-	918	protein_coding	regulator of cell cycle [Source:MGI Symbol;Acc:MGI:1913464]	-
ENSMUSG0000011320	519.0889803	478.1832162	764.1365065	309.2178597	394.0717051	343.4779781	587.1362343	348.9225143	0.750768404	0.0022429	0.0164772	Gm46430	13	74579358	74617945	-	5899	lincRNA	predicted gene, 46430 [Source:MGI Symbol;Acc:MGI:5826067]	-
ENSMUSG0000002485	3461.26127	3297.778965	3350.358747	2767.456778	2278.504821	2297.313049	3369.799661	2447.758216	0.461030437	0.002483	0.01651	Sf3b2	19	5273923	5295455	-	3231	protein_coding	splicing factor 3b, subunit 2 [Source:MGI Symbol;Acc:MGI:2441856]	-
ENSMUSG0000003760	2489.22206	2953.360657	3134.74713	2088.727883	2019.740945	2074.490225	2859.109949	2060.986351	0.472081472	0.002252	0.0165307	Bclaf1	10	20312469	20344613	+	15301	protein_coding	BCL2-associated transcription factor 1 [Source:MGI Symbol;Acc:MGI:1917580]	-
ENSMUSG0000000447	76.15977317	36.86434486	64.79520084	233.4207242	103.7030803	113.8439758	59.27310629	150.3225934	-1.34371011	0.0022531	0.0165322	Clec11a	7	44302687	44306902	-	2978	protein_coding	C-type lectin domain family 11, member a [Source:MGI Symbol;Acc:MGI:1298219]	-
ENSMUSG0000008308	53.11142077	63.19601977	44.68634541	18.0879528	26.66650636	14.59538151	53.66459531	19.78328022	1.442349517	0.0022554	0.0165431	Gm11249	4	67930881	67931739	+	859	processed_pseudog	predicted gene 11249 [Source:MGI Symbol;Acc:MGI:3650834]	-
ENSMUSG0000002575	112.2354552	76.88849072	58.09224903	145.5649535	165.9249285	184.8748324	82.40539832	165.4549048	-1.002417703	0.0022573	0.0165503	Plk4	3	40800019	40816883	+	6855	protein_coding	polo like kinase 4 [Source:MGI Symbol;Acc:MGI:101783]	-
ENSMUSG0000003949	5.010511393	2.106533992	0	43.92788537	13.82707737	12.64933064	2.372348462	23.46809779	-3.286536539	0.0022606	0.0165677	Ccdc27	4	154026369	154042677	-	2089	protein_coding	coiled-coil domain containing 27 [Source:MGI Symbol;Acc:MGI:2685881]	-
ENSMUSG0000005112	156.3279555	193.8011273	242.4234238	119.7250209	94.81424484	111.8979249	197.5175022	108.8123969	0.858036148	0.002264	0.0165865	Gimap9	6	48676129	48679114	+	1642	protein_coding	GTPase, IMAP family member 9 [Source:MGI Symbol;Acc:MGI:3511744]	-
ENSMUSG0000002642	1.002102279	1.053266996	0	18.0879528	9.876483837	11.67630521	0.685123092	13.21358061	-4.244161911	0.0022654	0.01659	Gpr37l1	1	135160234	135167681	-	2269	protein_coding	G protein-coupled receptor 37-like 1 [Source:MGI Symbol;Acc:MGI:1928503]	-
ENSMUSG0000001763	27.05676152	14.74573795	11.17158635	259.2606568	23.70356121	29.19076301	17.65802861	104.0516603	-2.55801869	0.0022667	0.016593	Rab11fip4	11	79591212	79698023	+	7451	protein_coding	RAB11 family interacting protein 4 (class II) [Source:MGI Symbol;Acc:MGI:2442920]	-
ENSMUSG0000005452	121.2543757	151.6704474	126.2389258	250.6473459	197.5296767	220.8767735	133.054583	223.017932	-0.746063764	0.0022697	0.0166089	Sh3bp2	5	34525838	34563641	+	4296	protein_coding	SH3-domain binding protein 2 [Source:MGI Symbol;Acc:MGI:1346349]	-
ENSMUSG0000004840	123.2585803	80.0482917	67.02951811	164.5142373	195.55438	168.3334	90.11213003	176.1340058	-0.964026619	0.0022742	0.0166346	Gli2	1	118834132	119053619	-	6869	protein_coding	GLI-Kruppel family member GLI2 [Source:MGI Symbol;Acc:MGI:95728]	zf-C2
ENSMUSG0000008539	2164.540922	1967.502749	2819.708395	1628.777083	1586.163304	1443.969744	2317.250689	1552.970044	0.577183262	0.002275	0.0166346	Firre	X	50555744	50635321	-	8247	processed_transcrip	functional intergenic repeating RNA element [Source:MGI Symbol;Acc:MGI:2147989]	-
ENSMUSG0000002083	541.1352305	442.3721384	601.0313457	292.8525691	385.1828697	324.0174694	528.1795715	334.0176361	0.661573529	0.002278	0.016646	Coro6	11	77462411	77470484	+	3011	protein_coding	coronin 6 [Source:MGI Symbol;Acc:MGI:2183448]	-
ENSMUSG0000000973	715.5010269	747.8195672	719.4501611	524.5506311	538.2683691	518.6225562	727.5902517	527.1471855	0.464960925	0.0022784	0.016646	Pou6f1	15	100575318	100599984	-	6949	protein_coding	POU domain, class 6, transcription factor 1 [Source:MGI Symbol;Acc:MGI:102935]	Pou
ENSMUSG0000004837	11.02312506	9.479402965	50.27213858	2.583993257	3.950593535	1.946050868	23.59155554	2.82687922	3.060511452	0.0022898	0.0167234	Olfr1394	11	49154377	49162854	+	3024	protein_coding	olfactory receptor 1394 [Source:MGI Symbol;Acc:MGI:3031228]	-
ENSMUSG0000003051	291.6117631	391.8153225	494.9012754	259.2606568	207.4061606	209.2004683	392.7761203	225.2890952	0.800418763	0.0022933	0.0167424	Snrpa1	7	66059003	66074587	+	3172	protein_coding	small nuclear ribonucleoprotein polypeptide A' [Source:MGI Symbol;Acc:MGI:1916231]	-
ENSMUSG0000001550	764.6040386	754.1391692	868.0322596	966.413478	1664.187527	1262.013988	795.5918225	1297.538331	-0.705482063	0.0022951	0.0167484	Hivep2	10	13966075	14151374	+	13720	protein_coding	human immunodeficiency virus type I enhancer binding protein 2 [Source:MGI Symbol;Acc:MGI:1338076]	zf-C2
ENSMUSG0000010714	32.06727292	52.6633498	45.80350404	12.91996628	13.82707737	17.51445781	43.51137559	14.75383382	1.561262236	0.0022965	0.0167524	Gm6598	5	130220451	130223065	-	2615	antisense	predicted gene 6598 [Source:MGI Symbol;Acc:MGI:3646314]	-
ENSMUSG0000006093	6823.314415	7040.036602	7533.000677	5826.904794	5000.463767	4928.373822	7132.117231	5251.914128	0.441382677	0.0022978	0.0167554	Rpl26	11	68901583	68906989	+	1444	protein_coding	ribosomal protein L26 [Source:MGI Symbol;Acc:MGI:106022]	-
ENSMUSG0000002229	754.5830158	1421.910445	755.1992374	531.4412798	499.7500822	591.5994637	977.2308993	540.9302752	0.853294548	0.0022989	0.0167564	Slc25a32	15	39091231	39112716	-	6081	protein_coding	solute carrier family 25, member 32 [Source:MGI Symbol;Acc:MGI:1917156]	-
ENSMUSG0000002638	438.920798	346.5248417	894.8440668	306.6338665	244.9367992	251.0405619	560.0965689	267.5370759	1.065095006	0.0022997	0.0167564	Sctr	1	120006894	120063536	+	2012	protein_coding	secretin receptor [Source:MGI Symbol;Acc:MGI:2441720]	-
ENSMUSG0000003132	11643.42638	11836.6145	11259.84188	9596.089625	7939.705357	8017.729574	11579.96092	8517.841519	0.443019924	0.002301	0.0167585	Rps4x	X	102184941	102189394	-	1631	protein_coding	ribosomal protein S4, X-linked [Source:MGI Symbol;Acc:MGI:98158]	-
ENSMUSG0000009695	118.2480689	225.3991372	253.5950102	52.54119622	111.6042674	92.43741621	199.0807387	85.5276266	1.220340425	0.0023018	0.0167585	Gdap10	12	32821872	32826904	+	5003	lincRNA	ganglioside-induced differentiation-associated-protein 10 [Source:MGI Symbol;Acc:MGI:1338008]	-
ENSMUSG0000003287	701.471595	1168.073099	620.0230425	444.4468402	483.947708	490.4048186	829.8559121	472.9331223	0.811458039	0.0023029	0.0167601	Ccdc85a	11	28385685	28584324	-	6971	protein_coding	coiled-coil domain containing 85A [Source:MGI Symbol;Acc:MGI:2445069]	-
ENSMUSG0000000102	677.4211403	674.0908775	494.9012754	442.724178	374.3187374	360.9924359	615.4710978	392.6784505	0.648213523	0.002308	0.0167901	Icam2	11	106377656	106388075	-	1904	protein_coding	intercellular adhesion molecule 2 [Source:MGI Symbol;Acc:MGI:96394]	-
ENSMUSG0000002551	18.03784102	31.59800988	112.8330222	11.19730411	13.82707737	8.757228904	54.15629102	11.2605368	2.2637272	0.0023152	0.0168361	Muc2	7	141690340	141754693	+	7127	protein_coding	mucin 2 [Source:MGI Symbol;Acc:MGI:1339364]	-
ENSMUSG0000002937	726.524152	383.3891866	515.0101308	940.5735455	913.574755	904.9136534	541.6411565	919.6873179	-0.763436551	0.0023186	0.0168547	Pf4	5	90772435	90773383	+	721	protein_coding	platelet factor 4 [Source:MGI Symbol;Acc:MGI:1888711]	-
ENSMUSG0000004681	24.05045469	35.81107787	11.17158635	271.319292	30.6170999	51.57034799	23.6777063	117.8355799	-2.315171741	0.0023214	0.0168681	Ddit4l	3	137621612	137628333	+	2916	protein_coding	DNA-damage-inducible transcript 4-like [Source:MGI Symbol;Acc:MGI:1920534]	-
ENSMUSG0000004370	255.536081	260.156948	270.3523587	366.9270425	385.1828697	403.805555	262.0151396	385.3051557	-0.556307404	0.0023243	0.016883	Pde12	14	26659958	26669883	-	7725	protein_coding	phosphodiesterase 12 [Source:MGI Symbol;Acc:MGI:2443226]	-
ENSMUSG0000007489	86.18079596	106.3799666	107.247229	40.48256102	47.40712242	59.35455146	99.93599718	49.08141163	1.027294867	0.0023275	0.0168998	Sptbn5	2	120041493	120085693	-	11662	processed_transcrip	spectrin beta, non-erythrocytic 5 [Source:MGI Symbol;Acc:MGI:2685200]	-
ENSMUSG0000007137	920.931994	1114.356482	1139.501808	808.7898894	672.5885493	694.7401597	1058.263428	725.3728661	0.544276006	0.0023299	0.0169104	Hpcal1	12	17690856	17791933	+	1695	protein_coding	hippocalcin-like 1 [Source:MGI Symbol;Acc:MGI:1855689]	-

ENSMUSG0000005062	580.2172193	562.4445759	732.8560647	455.6441443	372.3434407	398.9404278	625.17262	408.9760043	0.611281763	0.0023346	0.0169326	Ubald2	11	116434094	116439077	+	1532	protein_coding	UBA-like domain containing 2 [Source:MGI Symbol;Acc:MGI:1914635]	-
ENSMUSG0000003736	113.2375575	75.83522372	82.66973901	209.3034538	192.5914348	130.3854081	90.58084007	177.4267656	-0.969396284	0.0023348	0.0169326	Nov	15	54745702	54754039	+	2869	protein_coding	nephroblastoma overexpressed gene [Source:MGI Symbol;Acc:MGI:109185]	-
ENSMUSG0000003676	13.02732962	15.79900494	8.937269082	55.12518948	28.64180313	40.86706822	12.58786788	41.54468694	-1.722088162	0.0023368	0.0169404	Kif15	9	122951046	123018733	+	11020	protein_coding	kinesin family member 15 [Source:MGI Symbol;Acc:MGI:1098258]	-
ENSMUSG0000002485	963.0202898	605.6285228	1466.829288	536.6092663	540.2436659	530.2988614	1011.826034	535.7172645	0.91718851	0.0023395	0.0169533	Pold4	19	4231899	4233631	+	910	protein_coding	polymerase (DNA-directed), delta 4 [Source:MGI Symbol;Acc:MGI:1916995]	-
ENSMUSG0000004026	3.006306836	3.159800988	0	9.474641942	20.74061606	28.21773758	2.055369275	19.47766519	-3.222713039	0.0023417	0.0169633	Gbp2b	3	142594847	142619179	+	2823	protein_coding	guanylate binding protein 2b [Source:MGI Symbol;Acc:MGI:95666]	-
ENSMUSG0000005821	11.02312506	54.7698838	25.69464861	7.75197977	1.975296767	4.865127169	30.49588582	4.864134569	2.635628659	0.0023461	0.0169789	Gstp3	19	4057477	4059446	-	709	protein_coding	glutathione S-transferase pi 3 [Source:MGI Symbol;Acc:MGI:2385078]	-
ENSMUSG0000001820	675.4169358	616.1611927	487.081165	844.1044639	889.8711938	914.6439078	592.8864312	882.8731885	-0.573764839	0.0023468	0.0169789	Stk4	2	164070322	164155524	+	5860	protein_coding	serine/threonine kinase 4 [Source:MGI Symbol;Acc:MGI:1929004]	-
ENSMUSG0000002550	5801.170091	5244.216374	4704.355013	6522.860311	8017.729579	7660.62924	5249.913826	7400.406377	-0.495204052	0.0023468	0.0169789	Taldo1	7	141392199	141402968	+	2171	protein_coding	transaldolase 1 [Source:MGI Symbol;Acc:MGI:1274789]	-
ENSMUSG0000003306	690.44847	448.6917403	442.3948195	722.6567808	1034.067858	924.3741621	527.1783433	893.6996002	-0.760689655	0.0023475	0.0169789	Entpd6	2	150749042	150771675	+	4775	protein_coding	ectonucleoside triphosphate diphosphohydrolase 6 [Source:MGI Symbol;Acc:MGI:1202295]	-
ENSMUSG0000004541	839.7617095	607.7350567	617.7887253	935.405559	1207.893973	1062.543774	688.4284972	1068.614435	-0.633738963	0.0023526	0.0170075	1190002N15Ri	9	94517864	94538081	-	3950	protein_coding	RIKEN cDNA 1190002N15 gene [Source:MGI Symbol;Acc:MGI:1916111]	-
ENSMUSG0000008752	43.09039798	62.14275277	53.62361449	18.94928388	24.69120959	17.51445781	52.95225508	20.38498376	1.378095807	0.0023533	0.0170075	Gm12319	11	70654741	70656493	-	357	lincRNA	predicted gene 12319 [Source:MGI Symbol;Acc:MGI:3649749]	-
ENSMUSG0000004477	14.0294319	9.479402965	15.64022089	131.7836561	29.62945151	23.35261041	13.04968525	61.58857267	-2.242331245	0.0023584	0.0170378	Scml4	10	42860370	42960780	+	7725	protein_coding	Scm polycomb group protein like 4 [Source:MGI Symbol;Acc:MGI:2446140]	-
ENSMUSG0000007125	113.2375575	122.1789715	67.02951811	206.7194605	177.7767091	190.712985	100.815349	191.7363849	-0.92590616	0.0023632	0.0170657	2210408I21Rik	13	77135540	77613784	+	5948	protein_coding	RIKEN cDNA 2210408I21 gene [Source:MGI Symbol;Acc:MGI:1919621]	-
ENSMUSG0000010396	17.03573874	40.02414585	44.68634541	80.10379096	71.11068363	121.6281792	33.91541	90.94755127	-1.426586209	0.002368	0.0170942	Gm30173	3	52516964	52550219	+	1128	lincRNA	predicted gene, 30173 [Source:MGI Symbol;Acc:MGI:5589332]	-
ENSMUSG0000002089	22.04625013	28.43820889	51.38929722	7.75197977	11.8517806	3.892101735	33.95791875	7.831954037	2.115263503	0.0023713	0.017111	Aloxe3	11	69125896	69149115	+	6397	protein_coding	arachidonate lipoxygenase 3 [Source:MGI Symbol;Acc:MGI:1345140]	-
ENSMUSG0000003235	3.006306836	3.159800988	0	84.41044639	7.90118707	3.892101735	2.055369275	32.06791173	-3.952886624	0.0023793	0.0171624	Tinag	9	76951693	77045794	-	1735	protein_coding	tubulointerstitial nephritis antigen [Source:MGI Symbol;Acc:MGI:1349477]	-
ENSMUSG0000004798	569.1940943	572.9772459	539.5876208	743.3287269	797.0322457	783.2854742	560.5863203	774.5488156	-0.46607806	0.0023829	0.0171814	Ino80c	18	24104757	24121953	-	2828	protein_coding	INO80 complex subunit C [Source:MGI Symbol;Acc:MGI:2443014]	-
ENSMUSG0000010205	24.05045469	17.90553893	68.14667675	1.722662171	8.888835454	8.757228904	36.70089012	6.456242176	2.516902055	0.0023845	0.0171863	I830127L07Rik	15	75131377	75135128	-	798	transcribed_unproc	RIKEN cDNA I830127L07 gene [Source:MGI Symbol;Acc:MGI:3612406]	-
ENSMUSG0000009045	0	0	0	38.75989885	1.975296767	0.973025434	0	13.90274035	-6.164372121	0.0023899	0.0172191	4930571K23Ri	7	125368861	125371013	+	2153	lincRNA	RIKEN cDNA 4930571K23 gene [Source:MGI Symbol;Acc:MGI:1923111]	-
ENSMUSG0000005881	435.9144912	804.695985	754.0820788	315.2471773	371.3557923	430.0772417	664.8975183	372.2267371	0.837000883	0.0023966	0.0172592	Pirb	7	3711409	3720391	-	4471	protein_coding	paired Ig-like receptor B [Source:MGI Symbol;Acc:MGI:894311]	-
ENSMUSG0000002518	39.08198887	50.55681581	77.08394583	109.3890479	130.3695867	113.8439758	55.57425017	117.8675368	-1.087754591	0.0023973	0.0172592	Loxl4	19	42593982	42612813	-	4213	protein_coding	lysyl oxidase-like 4 [Source:MGI Symbol;Acc:MGI:1914823]	-
ENSMUSG0000008383	314.6601155	273.849419	274.8210243	173.9888793	145.1843124	207.2544174	287.7768529	175.4758697	0.713902021	0.0023994	0.017267	Gm13841	5	115801677	115802159	-	483	processed_pseudog	predicted gene 13841 [Source:MGI Symbol;Acc:MGI:3650890]	-
ENSMUSG0000008732	9.018920508	5.26633498	30.16328315	0.861331086	0.987648384	0.973025434	14.81617955	0.940668301	3.976840415	0.0024018	0.0172743	Gm12503	4	44092632	44109311	+	3127	processed_transcrip	predicted gene 12503 [Source:MGI Symbol;Acc:MGI:3651570]	-
ENSMUSG0000000298	129305.2654	171264.3734	64708.06247	69884.95896	54491.52428	65947.7718	121759.2338	63441.41835	0.940533774	0.0024031	0.0172743	Apoe	7	19696109	19699188	-	2138	protein_coding	apolipoprotein E [Source:MGI Symbol;Acc:MGI:88057]	-
ENSMUSG0000008591	43.09039798	24.22514091	65.91235948	14.64262846	12.83942899	11.67630521	44.40929946	13.05278755	1.76297453	0.0024031	0.0172743	Gm14320	2	167662109	167667697	+	1023	antisense	predicted gene 14320 [Source:MGI Symbol;Acc:MGI:3649455]	-
ENSMUSG0000007823	2774.821209	1367.140561	3333.601367	3895.8005	5480.460881	4852.477838	2491.854379	4742.913073	-0.928565666	0.0024069	0.0172949	Klhdc7a	4	139960220	139968026	-	7807	protein_coding	kelch domain containing 7A [Source:MGI Symbol;Acc:MGI:2444612]	-
ENSMUSG0000002203	0	4.213067984	0	35.31457451	7.90118707	19.46050868	1.404355995	20.89209008	-3.895198272	0.002416	0.0173531	Pbk	14	65805837	65817822	+	1791	protein_coding	PDZ binding kinase [Source:MGI Symbol;Acc:MGI:1289156]	-
ENSMUSG0000006414	70.1471595	81.1015587	206.6743475	40.48256102	51.35771595	37.94799192	119.3076886	43.2627563	1.462423781	0.0024179	0.0173607	Zfp69	4	120930137	120951699	-	2782	protein_coding	zinc finger protein 69 [Source:MGI Symbol;Acc:MGI:107794]	zf-C2
ENSMUSG0000003846	2812.901096	3016.556677	3037.554329	2219.650208	2205.418841	2314.827507	2955.670701	2246.632185	0.395683811	0.0024241	0.0173983	Chmp4b	2	154651705	154694785	+	2027	protein_coding	charged multivesicular body protein 4B [Source:MGI Symbol;Acc:MGI:1922858]	-
ENSMUSG0000003121	2435.108537	1314.477211	2187.396608	1112.839763	1250.362854	1186.118004	1978.994119	1183.106873	0.742285312	0.0024259	0.0174046	Efnb1	X	99136130	99148991	+	3452	protein_coding	ephrin B1 [Source:MGI Symbol;Acc:MGI:102708]	-
ENSMUSG0000004102	6.012613672	20.01207293	4.468634541	61.15450708	27.65415474	44.75916995	10.16444038	44.52261059	-2.130954684	0.0024283	0.017415	Map7d2	X	159414572	159498757	-	4121	protein_coding	MAP7 domain containing 2 [Source:MGI Symbol;Acc:MGI:1917474]	-
ENSMUSG0000008651	34.07147747	77.94175771	41.3348695	12.0586352	22.71591283	10.70327977	51.1160349	15.15927593	1.757026995	0.0024356	0.0174607	9130208D14Ri	7	105824223	105831515	-	7293	unprocessed_pseud	RIKEN cDNA 9130208D14 gene [Source:MGI Symbol;Acc:MGI:1924950]	-
ENSMUSG0000003857	15.03153418	5.26633498	12.28874499	472.0094349	19.75296767	36.97496648	10.86220472	176.2457897	-4.020260679	0.0024374	0.0174652	Susd4	1	182763860	182896591	+	3858	protein_coding	sushi domain containing 4 [Source:MGI Symbol;Acc:MGI:2138351]	-
ENSMUSG0000004913	737.5472771	566.6576439	687.0525607	324.7218193	472.0959274	448.564725	663.7524939	415.1274906	0.678001661	0.0024381	0.0174652	C5ar1	7	16246743	16259540	-	2570	protein_coding	complement component 5a receptor 1 [Source:MGI Symbol;Acc:MGI:88232]	-
ENSMUSG0000002573	346.7273884	225.3991372	233.4861548	388.4603196	479.0094661	491.3778441	268.5375601	452.9492099	-0.752899303	0.0024391	0.0174656	Jmjd8	17	25828867	25831842	+	2758	protein_coding	jumonji domain containing 8 [Source:MGI Symbol;Acc:MGI:1919356]	-
ENSMUSG0000003122	1163.440745	1300.78474	1129.44738	925.0695859	780.2422232	853.3433054	1197.890955	852.8850382	0.489800598	0.0024416	0.0174767	Igbp1	X	100494291	100516125	+	5717	protein_coding	immunoglobulin (CD79A) binding protein 1 [Source:MGI Symbol;Acc:MGI:1346500]	-
ENSMUSG0000002286	471.9901732	402.3479925	552.9935244	266.1513054	349.6275278	275.3661978	475.7772301	297.0483437	0.679912349	0.0024448	0.0174931	D16Ertd472e	16	78540338	78576708	-	6548	protein_coding	DNA segment, Chr 16, ERATO Doi 472, expressed [Source:MGI Symbol;Acc:MGI:1196400]	-
ENSMUSG0000001890	3309.943826	3747.523972	2419.765604	1951.77624	2229.122402	2025.838953	3159.077801	2068.912532	0.610780177	0.0024487	0.0175145	Arrb1	7	99535466	99606771	+	11175	protein_coding	arrestin, beta 1 [Source:MGI Symbol;Acc:MGI:99473]	-
ENSMUSG0000002608	7.01471595	2.106533992	5.585793176	24.97860148	25.67885798	21.40655954	4.902347706	24.02133967	-2.290271758	0.0024501	0.017518	Chst10	1	38863867	38898161	-	11964	protein_coding	carbohydrate sulfotransferase 10 [Source:MGI Symbol;Acc:MGI:2138283]	-
ENSMUSG0000004144	590.2382421	763.6185722	506.0728618	946.6028631	966.9077677	935.0774419	619.9765587	949.5293575	-0.6147501	0.0024587	0.0175672	Arhgap32	9	32116136	32268446	+	17179	protein_coding	Rho GTPase activating protein 32 [Source:MGI Symbol;Acc:MGI:2450166]	-
ENSMUSG0000003602	9.018920508	23.17187391	17.87453816	3.445324342	0.987648384	0.973025434	16.6884442	1.801999387	3.181605899	0.0024589	0.0175672	1810046K07Ri	9	51289686	51328917	-	1437	protein_coding	RIKEN cDNA 1810046K07 gene [Source:MGI Symbol;Acc:MGI:1917059]	-
ENSMUSG0000002638	13568.46485	6747.228377	16465.80112	19050.05962	23530.72274	26963.5078	12260.49812	23181.43005	-0.918955943	0.002461	0.0175757	Dbi	1	120113280	120121078	-	1422	protein_coding	diazepam binding inhibitor [Source:MGI Symbol;Acc:MGI:94865]	-
ENSMUSG0000006434	3.006306836	2.106533992	1.117158635	31.86925017	39.50593535	1.946050868	2.076666488	24.44041213	-3.547770929	0.0024654	0.0175999	mt-Tl1	MT	2676	2750	+	75	Mt_tRNA	mitochondrially encoded tRNA leucine 1 [Source:MGI Symbol;Acc:MGI:102482]	-
ENSMUSG0000011429	0	0	0	9.474641942	4.938241919	10.70327977	0	8.372054544	-5.429874143	0.0024679	0.0176114	Gm48371	14	9443375	9469555	-	457	lincRNA	predicted gene, 48371 [Source:MGI Symbol;Acc:MGI:6097844]	-
ENSMUSG0000002299	5.010511393	0	6.702951811	48.23454079	13.82707737	25.29866128	3.904487735	29.12009315	-2.906025477	0.0024761	0.0176629	Wnt10b	15	98770712	98778150	-	3401	protein_coding	wingless-type MMTV integration site family, member 10B [Source:MGI Symbol;Acc:MGI:108061]	-
ENSMUSG0000005283	4217.848491	4273.104203	3526.869811	2826.888623	2835.53851	1668.738619	4005.940835	2443.721917	0.71301717	0.002478	0.0176699	Junb	8	84974484	84978718	-	2301	protein_coding	jun B proto-oncogene [Source:MGI Symbol;Acc:MGI:96647]	TF_b
ENSMUSG0000003166	1519.187054	1744.210146	1038.957531	832.9071598	941.2289097	914.6439078	1434.118244	896.2599924	0.678493378	0.0024844	0.017709	Rbl2	8	91070057	91123844	+	7558	protein_coding	RB transcriptional corepressor like 2 [Source:MGI Symbol;Acc:MGI:105085]	-
ENSMUSG0000008558	88.18500052	72.67542273	63.67804221	43.06655428	24.69120959	17.51445781	74.84615515	28.42407389	1.391420843	0.0024884	0.0177309	Gm11613	11	97644184	97660367	-	1971	processed_transcrip	predicted gene 11613 [Source:MGI Symbol;Acc:MGI:3651819]	-
ENSMUSG0000002223	199.4183534	117.9659036	131.824709	96.46908159	45.43182565	52.54337342	149.7363253	64.81476022	1.205038494	0.0024894	0.0177311	Ankrd33b	15	31291478	31367726	-	8761	protein_coding	ankyrin repeat domain 33B [Source:MGI Symbol;Acc:MGI:1917904]	-
ENSMUSG0000002275	989.074949	864.7322038	733.9732233	586.5664693	612.3419979	562.4087007	862.5934587	587.1057226	0.555477687	0.0024981	0.0177862	Klhl22	16	17759618	17793382	+	5234	protein_coding	kelch-like 22 [Source:MGI Symbol;Acc:MGI:1337995]	-
ENSMUSG0000003900	1152.41762	579.2968479	833.4003419	1366.932433	1516.040269	1503.324295	855.03827	1462.098999	-0.773695439	0.0024991	0.0177867	Cpq	15	33083129	33594552	+	5026	protein_coding	carboxypeptidase Q [Source:MGI Symbol;Acc:MGI:1889205]	-
ENSMUSG0000003264	250.5255697	291.7549579	338.4990665	459.9507997	407.8987825	502.0811238	293.593198	456.6435687	-0.63818945	0.0025134	0.0178817	Chsy1	7	66109515	66173798	+	4176	protein_coding	chondroitin sulfate synthase 1 [Source:MGI Symbol;Acc:MGI:2681120]	-
ENSMUSG0000007281	339.7126725	338.0987057	526.1817172	267.8739676	223.2085347	201.4162648	401.3310318	230.8329224	0.796513555	0.002516	0.0178934	Ahnak2	12	112772194	112802657	-	14163	protein_coding	AHNAK nucleoprotein 2 [Source:MGI Symbol;Acc:MGI:2144831]	-
ENSMUSG0000008480	46.09670482	33.70454388	41.3348695	13.78129737	16.79002252	9.730254338	40.37870606	13.43385808	1.588209972	0.0025248	0.0179487	5830444B04Ri	4	155398763	155421704	-	5410	processed_transcrip	RIKEN cDNA 5830444B04 gene [Source:MGI Symbol;Acc:MGI:3603170]	-
ENSMUSG0000003128	10002.98495	3587.427389	11103.43968	3601.225269	4309.109898	3994.269406	8231.284003	3968.201524	1.052639782	0.0025267	0.0179555	Chrdl1	X	143285674	143394262	-	6758	protein_coding	chordin-like 1 [Source:MGI Symbol;Acc:MGI:1933172]	-
ENSMUSG0000004479	2717.70138	2970.212929	2959.353225	1987.090814	2279.49247	2165.954616	2882.422511	2144.1793	0.42694394	0.002534	0.0180003	Isca1	13	59755409	59769810	-	10680	protein_coding	iron-sulfur cluster assembly 1 [Source:MGI Symbol;Acc:MGI:1916296]	-
ENSMUSG0000002225	669.4043221	698.3160184	537.3533035	441.8628469	424.688805	425.2121146	635.024548	430.5879222	0.560751703	0.0025384	0.0180249	Nadk2	15	9071260	9110891	+	5118	protein_coding	NAD kinase 2, mitochondrial [Source:MGI Symbol;Acc:MGI:1915896]	-
ENSMUSG0000007492	241.5066491	235.9318071	250.2435343	353.1457451	343.7016375	378.5068937	242.5606635	358.4514255	-0.563543264	0.0025517	0.0181129	Fam122a	19	24476502	24477356	-	855	protein_coding	family with sequence similarity 122, member A [Source:MGI Symbol;Acc:MGI:1915284]	-
ENSMUSG0000000854	21232.54308	14837.37217	19792.69954	11488.43402	12403.87605	13876.31571	18620.8716	12589.54193	0.564712209	0.0025658	0.0182055	Mgst1	6	138140316	138156755	+	2013	protein_coding	microsomal glutathione S-transferase 1 [Source:MGI Symbol;Acc:MGI:1913850]	-
ENSMUSG0000004010	348.731593	315.9800988	339.6162251	493.542712	527.4042369	445.6456487	334.7759723	488.8641992	-0.546162063	0.002584	0.0183279	Klhl42	6	147091379	147112778	+	6449	protein_coding	kelch-like 42 [Source:MGI Symbol;Acc:MGI:2444786]	-
ENSMUSG0000002986	144.3027281	126.3920395	97.19280126	227.3914066	172.8384672	288.0155284	122.6291896	229.4151341	-0.902442398	0.0025903	0.0183659	Ephb6	6	41605482	41620509	+	3773	protein_coding	Eph receptor B6 [Source:MGI Symbol;Acc:MGI:1096338]	-
ENSMUSG0000007692	15.03153418	7.372868973	13.40590362	39.62122994	32.59239666	42.81311909	11.93676892	38.34224856	-1.682998515	0.0025916	0.0183677	Trac	14	54220521	54224206	+	935	TR_C_gene	T cell receptor alpha constant [Source:MGI Symbol;Acc:MGI:4439838]	-
ENSMUSG0000002048	3977.343944	3197.7186	2446.577411	4453.081713	5128.858057	5407.102336	3207.213318	4996.347368	-0.63939005	0.002593	0.0183707	Dynll2	11	87979525	87987533	-	2736	protein_coding	dynein light chain LC8-type 2 [Source:MGI Symbol;Acc:MGI:1915347]	-
ENSMUSG0000009785	12.02522734	10.53266996	16.75737953	0.861331086	0	1.946050868	13.10509228	0.935793984	3.805123452	0.0025983	0.018398	4933405D12Ri	3	122864737	122924116	-	1479	antisense	RIKEN cDNA 4933405D12 gene [Source:MGI Symbol;Acc:MGI:1921307]	-
ENSMUSG0000009671	6.012613672	4.213067984	14.52306226	0	0	0	8.249581305	0	5.57553424	0.002599	0.018398	Mrgpra2b	7	47463806	47528862	-	1334	protein_coding	MAS-related GPR, member A2B [Source:MGI Symbol;Acc:MGI:3033098]	-
ENSMUSG0000005641	122.256478	235.9318071	120.6531326	237.7273796	361.4793085	376.5608429	159.6138059	325.2558436	-1.026292804	0.0026007	0.018398	Adap1	5	139271876	139325622	-	3292	protein_coding	ArfGAP with dual PH domains 1 [Source:MGI Symbol;Acc:MGI:2442201]	-
ENSMUSG0000002008	742.5577885	676.1974115	618.9058839	961.2454915	1076.536738	886.4261702	679.2203613	974.7361333	-0.52072252	0.0026008	0.018398	Tysnd1	10	61695514	61702773	+	2286	protein_coding	trypsin domain containing 1 [Source:MGI Symbol;Acc:MGI:1919017]	-
ENSMUSG0000004499	141.2964213	148.5106465	191.0341266	78.38112879	104.6907287	79.78808557	160.2803981	87.61998101	0.871616406	0.0026032	0.0184082	Shld1	2	132686931	132751055	+	2117	protein_coding	shieldin complex subunit 1 [Source:MGI Symbol;Acc:MGI:1920997]	-
ENSMUSG0000000210	225.4730127	250.6775451	174.2767471	130.922325	136.295477	90.49136534	216.8091016	119.2363891	0.862564544	0.0026056	0.0184185	Ddb2	2	91211572	91236982	-	2736	protein_coding	damage specific DNA binding protein 2 [Source:MGI Symbol;Acc:MGI:1355314]	-
ENSMUSG0000003322	293.6159676	331.7791038	328.4446388	453.060151	435.5529372	530.2988614	317.9465701	472.9706499	-0.573164658	0.0026185	0.0185027	Rac2	15	78559167	78572783	-	3103	protein_coding	Rac family small GTPase 2 [Source:MGI Symbol;Acc:MGI:97846]	-
ENSMUSG0000005489	230.4835241	187.4815253	246.8920584	142.9809602	138.2707737	112.8709503	221.6190359	131.3742281	0.753418763	0.0026202	0.0185061	Atp5s	12	69724950	69744660	+	2039	protein_coding	ATP synthase, H+ transporting, mitochondrial F0 complex, subunit S [Source:MGI Symbol;Acc:MGI:1915305]	-
ENSMUSG0000000568	781.6397773	853.1462668	1289.201065	618.4357195	618.2678882	559.4896244	974.6623697	598.7310774	0.702540314	0.002621	0.0185061	Pan2	10	128303335	128321358	+	8472	protein_coding	PAN2 poly(A) specific ribonuclease subunit [Source:MGI Symbol;Acc:MGI:1918984]	-
ENSMUSG0000002492	881.8500052	1133.315288	515.0101308	1381.575061	1579.249766	1551.975567	843.3918079	1504.266798	-0.834451098	0.0026343	0.0185933	Vldlr	19	27216484	27254231	+	10068	protein_coding	very low density lipoprotein receptor [Source:MGI Symbol;Acc:MGI:98935]	-
ENSMUSG0000004153	23.04835241	6.319601977	13.40590362	62.01583816	35.55534181	50.59732256	14.25795267	49.38950084	-1.789586066	0.0026394	0.0186222	H2-Ob	17	34238903	34245907	+	3153	protein_coding	histocompatibility 2, O region beta locus [Source:MGI Symbol;Acc:MGI:95925]	-
ENSMUSG0000004708	655.3748902	578.2435809	730.6217474	483.206739	401.9728922	442.7265724	654.7467395	442.6354012	0.564111344	0.0026411	0.0186271	Ngrn	7	80261215	80265374	+	1714	protein_coding	neugrin, neurite outgrowth associated [Source:MGI Symbol;Acc:MGI:1933212]	-
ENSMUSG0000003032	276.5802289	192.7478603	240.1891066	437.5561915	331.8498569	388.2371481	236.5057319	385.8810655	-0.706443491	0.0026432	0.0186352	Ift122	6	115853470	115926699	+	10319	protein_coding	intraflagellar transport 122 [Source:MGI Symbol;Acc:MGI:1932386]	-
ENSMUSG0000010929	208.437274	311.7670308	328.4446388	154.1782643	170.8631704	169.3064255	282.8829812	164.7826201	0.779247213	0.0026481	0.0186622	Dcst2	3	89361975	89377549	+	2285	protein_coding	DC-STAMP domain containing 2 [Source:MGI Symbol;Acc:MGI:2685606]	-
ENSMUSG0000002357	1404.947395	1329.222949	1827.671527	1032.735972	1019.253132	1066.435875	1520.613957	1039.474993	0.548585174	0.0026534	0.0186928	C1qtnf12	4	155962318	155966629	+	1366	protein_coding	C1q and tumor necrosis factor related 12 [Source:MGI Symbol;Acc:MGI:1914639]	-
ENSMUSG0000004025	100.2102279	65.30255376	42.45202814	207.5807916	155.0607962	115.7900266	69.32160325	159.4772048	-1.200157013	0.0026562	0.0186967	Sema3d	5	12383385	12588948	+	8595	protein_coding	sema domain, immunoglobulin domain (Ig), short basic domain, secreted, (semaphorin) 3D [Source:MGI Symbol;Acc:MGI:18601	-
ENSMUSG0000003637	4982.452529	4570.125496	4990.347624	3631.371857	3731.335594	3774.365658	4847.641883	3712.357703	0.384970961	0.0026567	0.0186967	Serbp1	6	67238176	67297736	+	20117	protein_coding	serpine1 mRNA binding protein 1 [Source:MGI Symbol;Acc:MGI:1914120]	-
ENSMUSG0000003704	1446.033588	1152.274094	948.4676813	1762.283401	1780.730036	1793.285874	1182.258454	1778.766437	-0.588970589	0.002657	0.0186967	Smpd1	7	105554360	105558389	+	3337	protein_coding	sphingomyelin phosphodiesterase 1, acid lysosomal [Source:MGI Symbol;Acc:MGI:98325]	-
ENSMUSG0000004622	276.5802289	285.4353559	506.0728618	142.1196291	220.2455896	189.7399596	356.0294822	184.0350594	0.952300252	0.0026588	0.0187025	Plaur	7	24462484	24475968	+	2617	protein_coding	plasminogen activator, urokinase receptor [Source:MGI Symbol;Acc:MGI:97612]	-
ENSMUSG0000003659	97.20392103	67.40908775	113.9501808	315.2471773	115.5548609	215.0386209	92.85439652	215.2802197	-1.21483359	0.0026635	0.0187286	Cpz	5	35502218	35525698	-	2245	protein_coding	carboxypeptidase Z [Source:MGI Symbol;Acc:MGI:88487]	-
ENSMUSG0000003026	148.3111372	107.4332336	98.3099599	200.6901429	187.6531929	243.2563584	118.0181102	210.5332314	-0.833204938	0.0026859	0.0188791	Lrmp	6	145115653	145174934	+	4070	protein_coding	lymphoid-restricted membrane protein [Source:MGI Symbol;Acc:MGI:108424]	-
ENSMUSG0000003344	2251.72382	2140.238536	1913.692742	1410.860318	1600.97803	1582.139355	2101.885033	1531.325901	0.457207768	0.0026976	0.0189539	Specc1l	10	75212073	75312743	+	13265	protein_coding	sperm antigen with calponin homology and coiled-coil domains 1-like [Source:MGI Symbol;Acc:MGI:1921642]	-
ENSMUSG0000003075	1816.811431	1613.605038	1217.702912	2119.735802	2356.529044	2556.137815	1549.373127	2344.13422	-0.597049559	0.0027038	0.0189905	Thap12	7	98703103	98718062	+	4125	protein_coding	THAP domain containing 12 [Source:MGI Symbol;Acc:MGI:1920231]	THA
ENSMUSG0000007034	347.7294907	77.94175771	106.1300703	801.0379096	342.7139892	412.5627839	177.2671062	518.7715609	-1.548451427	0.0027199	0.0190899	Ccnd1	7	144929931	144939925	-	4709	protein_coding	cyclin D1 [Source:MGI Symbol;Acc:MGI:88313]	-
ENSMUSG0000002008	6167.939525	5313.731995	7149.815265	3408.287106	4191.579741	4793.123287	6210.495595	4130.996711	0.588273074	0.0027205	0.0190899	Aifm2	10	61715263	61739260	+	4121	protein_coding	apoptosis-inducing factor, mitochondrion-associated 2 [Source:MGI Symbol;Acc:MGI:1918611]	-
ENSMUSG0000005532	2508.262003	2687.937374	6301.891861	1488.380116	2176.777038	1980.106758	3832.69708	1881.754637	1.026270611	0.002721	0.0190899	Tead1	7	112679318	112906807	+	13570	protein_coding	TEA domain family member 1 [Source:MGI Symbol;Acc:MGI:101876]	TEA
ENSMUSG0000000170	205.4309671	232.7720061	246.8920584	309.2178597	362.4669568	392.1292498	228.3650105	354.6046888	-0.634887532	0.002725	0.0191068	Gramd3	18	56432132	56503792	+	2605	protein_coding	GRAM domain containing 3 [Source:MGI Symbol;Acc:MGI:1914815]	-
ENSMUSG0000007411	0	101.1136116	16.75737953	0.861331086	1.975296767	3.892101735	39.29033705	2.242909863	4.139930501	0.0027255	0.0191068	Saa1	7	46740497	46742980	-	2478	protein_coding	serum amyloid A 1 [Source:MGI Symbol;Acc:MGI:98221]	-
ENSMUSG0000002166	3265.851326	3555.829379	4752.392834	2813.968657	2386.158495	2539.596382	3858.024513	2579.907845	0.580360515	0.0027301	0.0191249	Btf3	13	98309896	98317006	-	1739	protein_coding	basic transcription factor 3 [Source:MGI Symbol;Acc:MGI:1202875]	-
ENSMUSG0000001867	613.2865945	461.3309443	312.8044179	686.4808752	851.3529068	896.1564245	462.4739856	811.3300688	-0.809983654	0.0027304	0.0191249	Sp2	11	96953341	96982959	-	3414	protein_coding	Sp2 transcription factor [Source:MGI Symbol;Acc:MGI:1926162]	zf-C2
ENSMUSG0000003504	29.06096608	60.03621878	48.03782131	167.9595617	67.16009009	117.7360775	45.71166872	117.6185764	-1.366509439	0.0027311	0.0191249	Ccl5	11	83525778	83530518	-	960	protein_coding	chemokine (C-C motif) ligand 5 [Source:MGI Symbol;Acc:MGI:98262]	-
ENSMUSG0000000845	316.66432	327.5660358	365.3108737	454.7828132	514.5648079	500.135073	336.5137432	489.8275647	-0.541734889	0.0027339	0.0191371	Nutf2	8	105860580	105879330	+	2034	protein_coding	nuclear transport factor 2 [Source:MGI Symbol;Acc:MGI:1915301]	-
ENSMUSG0000003754	20.04204557	9.479402965	33.51475906	87.85577073	53.33301272	54.48942429	21.0120692	65.22606925	-1.639703144	0.0027375	0.0191551	H2-DMb2	17	34143307	34151553	+	1710	protein_coding	histocompatibility 2, class II, locus Mb2 [Source:MGI Symbol;Acc:MGI:95923]	-
ENSMUSG0000004679	85.17869368	108.4865006	158.6365262	141.258298	420.7382115	274.3931723	117.4339068	278.7965606	-1.247648366	0.0027448	0.0191992	Ppp1r3b	8	35375739	35388139	+	6216	protein_coding	protein phosphatase 1, regulatory subunit 3B [Source:MGI Symbol;Acc:MGI:2177268]	-
ENSMUSG0000003124	5485.507873	4309.968548	5543.341148	6187.802519	8129.333847	8765.013107	5112.93919	7694.049824	-0.589545189	0.0027475	0.0192027	Sh3bgrl	X	109095365	109197873	+	3298	protein_coding	SH3-binding domain glutamic acid-rich protein like [Source:MGI Symbol;Acc:MGI:1930849]	-
ENSMUSG0000002799	277.5823312	251.7308121	125.1217671	347.9777586	504.6883241	429.1042163	218.1449701	427.2567663	-0.968278879	0.0027479	0.0192027	Sfrp2	3	83766321	83774316	+	2153	protein_coding	secreted frizzled-related protein 2 [Source:MGI Symbol;Acc:MGI:108078]	-
ENSMUSG0000004045	1471.086145	1717.878471	2206.388305	1120.591742	1294.807031	1142.331859	1798.450973	1185.910211	0.600644021	0.0027494	0.0192027	Sgms1	19	32122727	32389714	-	10299	protein_coding	sphingomyelin synthase 1 [Source:MGI Symbol;Acc:MGI:2444110]	-
ENSMUSG0000000634	1006.110688	887.9040777	1118.275794	737.2994093	674.5638461	710.3085667	1004.096853	707.3906073	0.504972936	0.0027494	0.0192027	Ggt5	10	75589340	75617200	+	5922	protein_coding	gamma-glutamyltransferase 5 [Source:MGI Symbol;Acc:MGI:1346063]	-
ENSMUSG0000001432	897.8836416	561.3913089	631.1946289	1540.059981	952.0930419	1105.356893	696.8231932	1199.169972	-0.783167999	0.0027531	0.019221	Bicc1	10	70922832	71159700	-	7441	protein_coding	BicC family RNA binding protein 1 [Source:MGI Symbol;Acc:MGI:1933388]	-
ENSMUSG0000002587	266.5592061	204.3337972	194.3856025	276.4872785	544.1942594	413.5358094	221.7595353	411.4057824	-0.890183089	0.0027602	0.01926	Unc5a	13	54949411	55006018	+	4168	protein_coding	unc-5 netrin receptor A [Source:MGI Symbol;Acc:MGI:894682]	-
ENSMUSG0000006794	400.8409114	430.7862014	478.1438959	627.9103614	617.2802398	614.9520741	436.5903362	620.0475585	-0.506691701	0.0027607	0.01926	Zfp160	17	21008903	21043070	+	7194	protein_coding	zinc finger protein 160 [Source:MGI Symbol;Acc:MGI:108187]	zf-C2
ENSMUSG0000002604	616.2929013	674.0908775	1054.597752	462.534793	497.7747854	445.6456487	781.6605102	468.6517423	0.737611491	0.0027642	0.0192775	Ercc5	1	44147744	44181260	+	5864	protein_coding	excision repair cross-complementing rodent repair deficiency, complementation group 5 [Source:MGI Symbol;Acc:MGI:103582]	-
ENSMUSG0000006369	1414.968417	1286.039002	1206.531326	1694.238245	1934.803184	1745.607628	1302.512915	1791.549686	-0.45959862	0.0027662	0.0192843	Cycs	6	50562563	50566538	-	3404	protein_coding	cytochrome c, somatic [Source:MGI Symbol;Acc:MGI:88578]	-
ENSMUSG0000005920	1848.878704	168.5227194	3934.632713	17152.54724	14133.24837	21324.82541	1984.011379	17536.87367	-3.143922073	0.0027694	0.0192994	Lep	6	29060220	29073877	+	3354	protein_coding	leptin [Source:MGI Symbol;Acc:MGI:104663]	-
ENSMUSG0000001804	3614.582919	3220.890474	2751.561719	4815.7021	4182.690905	4521.649191	3195.678371	4506.680732	-0.495872194	0.0027709	0.0193026	Cyb5r3	15	83153494	83172592	-	4298	protein_coding	cytochrome b5 reductase 3 [Source:MGI Symbol;Acc:MGI:94893]	-
ENSMUSG0000000639	353.7421044	253.8373461	426.7545987	223.0847512	173.8261155	199.4702139	344.7780164	198.7936935	0.793279776	0.0027735	0.0193131	Hyi	4	118359990	118362744	+	2209	polymorphic_pseud	hydroxypyruvate isomerase (putative) [Source:MGI Symbol;Acc:MGI:1915430]	-
ENSMUSG0000000115	475.9985823	640.3863336	574.2195385	384.1536642	356.5410665	387.2641226	563.5348182	375.9862844	0.583388074	0.0027756	0.0193208	Mxd1	6	86647042	86669161	-	7072	protein_coding	MAX dimerization protein 1 [Source:MGI Symbol;Acc:MGI:96908]	bHLI
ENSMUSG0000002732	5.010511393	11.58593696	10.05442772	30.146588	25.67885798	43.78614452	8.883625356	33.2038635	-1.906703107	0.0027831	0.0193661	Knl1	2	119047119	119105501	+	8542	protein_coding	kinetochore scaffold 1 [Source:MGI Symbol;Acc:MGI:1923714]	-
ENSMUSG0000004071	461.9691504	616.1611927	568.6337453	692.5101928	976.7842515	901.0215517	548.9213628	856.7719987	-0.64218144	0.0027863	0.0193808	Sh3pxd2b	11	32347820	32428173	+	9709	protein_coding	SH3 and PX domains 2B [Source:MGI Symbol;Acc:MGI:2442062]	-
ENSMUSG0000002065	793.6650047	778.3643101	592.0940767	286.8232515	524.4412918	438.8344706	721.3744638	416.6996713	0.792833486	0.0027965	0.0194448	Klf11	12	24651274	24662789	+	4340	protein_coding	Kruppel-like factor 11 [Source:MGI Symbol;Acc:MGI:2653368]	zf-C2
ENSMUSG0000002765	44.09250026	8.426135969	78.20110447	348.8390897	115.5548609	84.65321274	43.5732469	183.0157211	-2.07283576	0.0027977	0.0194456	Wisp2	2	163820861	163833146	+	1924	protein_coding	WNT1 inducible signaling pathway protein 2 [Source:MGI Symbol;Acc:MGI:1328326]	-
ENSMUSG0000003910	2811.898994	2479.390509	3069.95193	3253.24751	5165.401047	4537.217598	2787.080477	4318.622052	-0.631743329	0.0028018	0.0194671	F13a1	13	36867178	37050244	-	6739	protein_coding	coagulation factor XIII, A1 subunit [Source:MGI Symbol;Acc:MGI:1921395]	-
ENSMUSG0000002396	126.2648871	151.6704474	119.535974	58.57051382	75.06127716	80.761111	132.4904362	71.46430066	0.892937906	0.002808	0.0195028	Cyp39a1	17	43667425	43751431	+	3053	protein_coding	cytochrome P450, family 39, subfamily a, polypeptide 1 [Source:MGI Symbol;Acc:MGI:1927096]	-
ENSMUSG0000006343	61.128239	10.53266996	30.16328315	58.57051382	188.6408413	121.6281792	33.94139737	122.9465114	-1.853573578	0.0028099	0.0195067	Wscd2	5	113490333	113589725	+	15332	protein_coding	WSC domain containing 2 [Source:MGI Symbol;Acc:MGI:2445030]	-
ENSMUSG0000002499	6122.844922	8033.267379	6099.686148	4525.433524	4567.873775	5073.354612	6751.932817	4722.220637	0.51585933	0.0028124	0.0195067	Eif3a	19	60761116	60790693	-	5176	protein_coding	eukaryotic translation initiation factor 3, subunit A [Source:MGI Symbol;Acc:MGI:95301]	-
ENSMUSG0000009008	10.02102279	12.63920395	25.69464861	1.722662171	2.962945151	0.973025434	16.11829178	1.886210919	3.096689571	0.0028124	0.0195067	Gm16587	1	164143432	164146366	-	863	antisense	predicted gene 16587 [Source:MGI Symbol;Acc:MGI:4415007]	-
ENSMUSG0000004221	180.3784102	169.5759864	145.2306226	294.5752313	237.0356121	262.7168671	165.061673	264.7759035	-0.681551504	0.0028127	0.0195067	Abhd14a	9	106440051	106447678	-	2111	protein_coding	abhydrolase domain containing 14A [Source:MGI Symbol;Acc:MGI:1915894]	-
ENSMUSG0000003975	378.7946613	324.4062348	355.256446	244.6180283	238.0232605	227.6879515	352.819114	236.7764134	0.575304973	0.0028162	0.0195236	Thap3	4	151982639	151988997	-	1334	protein_coding	THAP domain containing, apoptosis associated protein 3 [Source:MGI Symbol;Acc:MGI:1917126]	THA
ENSMUSG0000003085	1179.474382	1379.779765	1558.436296	1042.210614	925.4265356	848.4781783	1372.563481	938.7051091	0.547634667	0.0028253	0.0195795	Tacc2	7	130577438	130764785	+	15408	protein_coding	transforming, acidic coiled-coil containing protein 2 [Source:MGI Symbol;Acc:MGI:1928899]	-
ENSMUSG0000000868	10135.26245	10945.55062	10939.21736	8921.667385	7091.315395	6765.445841	10673.34348	7592.80954	0.491230087	0.0028366	0.0196448	Rpl10	X	74270812	74273135	+	2053	protein_coding	ribosomal protein L10 [Source:MGI Symbol;Acc:MGI:105943]	-
ENSMUSG0000003162	244.512956	317.0333658	119.535974	402.241617	461.2317952	509.8653273	227.0274319	457.7795798	-1.01074995	0.0028368	0.0196448	Casp3	8	46617291	46639689	+	3357	protein_coding	caspase 3 [Source:MGI Symbol;Acc:MGI:107739]	-
ENSMUSG0000009956	2.004204557	0	0	41.34389211	10.86413222	2.919076301	0.668068186	18.37570021	-4.740179739	0.0028398	0.0196509	Gm28729	9	96486001	96525264	-	1964	protein_coding	predicted gene 28729 [Source:MGI Symbol;Acc:MGI:5579435]	-
ENSMUSG0000008199	34.07147747	36.86434486	52.50645586	16.36529063	13.82707737	11.67630521	41.14742606	13.9562244	1.554998643	0.0028398	0.0196509	Gm13408	2	34564999	34565796	+	798	processed_pseudog	predicted gene 13408 [Source:MGI Symbol;Acc:MGI:3649613]	-
ENSMUSG0000000522	6.012613672	6.319601977	5.585793176	145.5649535	4.938241919	11.67630521	5.972669608	54.05983353	-3.180141151	0.0028492	0.0197084	Corin	5	72300025	72504473	-	5400	protein_coding	corin [Source:MGI Symbol;Acc:MGI:1349451]	-
ENSMUSG0000002255	489.025912	530.846566	517.2444481	701.1235037	749.6251233	684.0368799	512.3723087	711.595169	-0.473921472	0.0028546	0.0197383	Bop1	15	76452989	76477277	-	2957	protein_coding	block of proliferation 1 [Source:MGI Symbol;Acc:MGI:1334460]	-
ENSMUSG0000004177	1915.017454	1129.10222	2726.984229	1077.525188	1127.894454	910.751806	1923.701301	1038.723816	0.888945739	0.0028598	0.0197621	Enc1	13	97241105	97253034	+	4737	protein_coding	ectodermal-neural cortex 1 [Source:MGI Symbol;Acc:MGI:109610]	-
ENSMUSG0000003011	257.5402856	150.6171804	194.3856025	1037.903958	233.0850186	352.235207	200.8476895	541.0747279	-1.43009143	0.0028603	0.0197621	Ret	6	118151745	118197718	-	6763	protein_coding	ret proto-oncogene [Source:MGI Symbol;Acc:MGI:97902]	-
ENSMUSG0000002722	4.008409114	0	3.351475906	14.64262846	18.76531929	24.32563584	2.453295007	19.24452786	-2.968119085	0.0028612	0.0197621	Duoxa1	2	122302191	122313730	-	3977	protein_coding	dual oxidase maturation factor 1 [Source:MGI Symbol;Acc:MGI:2384861]	-
ENSMUSG0000003017	3.006306836	9.479402965	7.820110447	30.146588	25.67885798	28.21773758	6.768606749	28.01439452	-2.056692005	0.0028665	0.0197917	Klra13-ps	6	130290878	130306428	-	1041	transcribed_unproc	killer cell lectin-like receptor subfamily A, member 13, pseudogene [Source:MGI Symbol;Acc:MGI:1321090]	-
ENSMUSG0000004353	20.04204557	22.11860692	12.28874499	36.17590559	65.18479333	54.48942429	18.14979916	51.95004107	-1.511739367	0.0028698	0.0198073	Sorcs1	19	50143299	50678646	-	8441	protein_coding	sortilin-related VPS10 domain containing receptor 1 [Source:MGI Symbol;Acc:MGI:1929666]	-
ENSMUSG0000001556	126298.9586	133144.5342	83703.11074	134195.3831	211867.3683	256324.09	114382.2012	200795.6138	-0.811862281	0.0028747	0.0198339	Lpl	8	68880491	68907448	+	10478	protein_coding	lipoprotein lipase [Source:MGI Symbol;Acc:MGI:96820]	-
ENSMUSG0000003978	530.1121054	497.1420222	682.5839261	647.7209764	1293.819383	1116.060173	569.9460179	1019.200177	-0.838405312	0.0028775	0.0198458	Cpeb2	5	43233170	43289724	+	7816	protein_coding	cytoplasmic polyadenylation element binding protein 2 [Source:MGI Symbol;Acc:MGI:2442640]	-
ENSMUSG0000002409	2016.229785	1995.940958	1560.670613	2473.742878	2755.538991	2661.224561	1857.613785	2630.16881	-0.501454851	0.0028793	0.0198507	Twsg1	17	65921972	65951226	-	5153	protein_coding	twisted gastrulation BMP signaling modulator 1 [Source:MGI Symbol;Acc:MGI:2137520]	-
ENSMUSG0000007948	87.18289824	153.7769814	182.0968575	69.76781793	73.0859804	36.00194105	141.0189124	59.61857979	1.239982967	0.0028829	0.0198842	Phyhd1	2	30266203	30282149	+	2657	protein_coding	phytanoyl-CoA dioxygenase domain containing 1 [Source:MGI Symbol;Acc:MGI:3612860]	-
ENSMUSG0000000143	2491.226265	1130.155487	1733.830202	3072.367982	2846.402642	3571.003342	1785.070651	3163.257989	-0.825318297	0.0028885	0.0198993	Col18a1	10	77052178	77166548	-	7021	protein_coding	collagen, type XVIII, alpha 1 [Source:MGI Symbol;Acc:MGI:88451]	-
ENSMUSG0000002897	821.7238685	855.2528008	714.9815265	1048.239931	1116.042674	1171.522622	797.3193986	1111.935076	-0.4794318	0.0029061	0.0200135	Nos3	5	24364810	24384474	+	4132	protein_coding	nitric oxide synthase 3, endothelial cell [Source:MGI Symbol;Acc:MGI:97362]	-
ENSMUSG0000008501	30.06306836	12.63920395	10.05442772	3.445324342	0	0	17.58556668	1.148441447	3.878407194	0.0029113	0.0200389	Gm11335	13	23527011	23530943	-	1080	lincRNA	predicted gene 11335 [Source:MGI Symbol;Acc:MGI:3705194]	-
ENSMUSG0000002925	12.02522734	33.70454388	18.9916968	0	2.962945151	4.865127169	21.57382267	2.60935744	3.070591189	0.0029124	0.0200389	Gnrhr	5	86180754	86197901	-	3197	protein_coding	gonadotropin releasing hormone receptor [Source:MGI Symbol;Acc:MGI:95790]	-
ENSMUSG0000003424	731.5346634	580.3501149	777.5424101	338.5031166	520.4906982	435.9153943	696.4757295	431.6364031	0.690942892	0.002913	0.0200389	Slc25a37	14	69241848	69285112	-	6021	protein_coding	solute carrier family 25, member 37 [Source:MGI Symbol;Acc:MGI:1914962]	-
ENSMUSG0000003258	464.9754573	256.997147	506.0728618	593.457118	1026.166671	712.2546175	409.3484887	777.2928021	-0.924984997	0.0029237	0.0201048	Mst1r	9	107906873	107920383	+	5007	protein_coding	macrophage stimulating 1 receptor (c-met-related tyrosine kinase) [Source:MGI Symbol;Acc:MGI:99614]	-
ENSMUSG0000005335	62.13034127	62.14275277	51.38929722	30.146588	23.70356121	15.56840694	58.55413042	23.13951872	1.335595876	0.0029265	0.0201165	Gm9905	7	121033252	121035096	-	1845	TEC	predicted gene 9905 [Source:MGI Symbol;Acc:MGI:3642311]	-
ENSMUSG0000004118	758.5914249	736.2336303	744.0276511	534.8866042	563.9472271	539.0560903	746.2842354	545.9633072	0.451107059	0.0029304	0.0201297	Prkd2	7	16842902	16870464	+	5051	protein_coding	protein kinase D2 [Source:MGI Symbol;Acc:MGI:2141917]	-
ENSMUSG0000007661	2149.509388	776.2577761	115.0673394	7007.789712	8916.489608	8124.762372	1013.611501	8016.347231	-2.983366364	0.0029305	0.0201297	Ighm	12	113418558	113422730	-	1886	IG_C_gene	immunoglobulin heavy constant mu [Source:MGI Symbol;Acc:MGI:96448]	-
ENSMUSG0000003714	1.002102279	1.053266996	0	75.79713553	0	5.838152603	0.685123092	27.21176271	-5.294496113	0.0029339	0.0201445	Cfap61	2	145934784	146215039	+	10606	protein_coding	cilia and flagella associated protein 61 [Source:MGI Symbol;Acc:MGI:1926024]	-
ENSMUSG0000004169	355.7463089	411.8273955	621.1402012	801.8992407	925.4265356	655.8191424	462.9046352	794.3816395	-0.779799607	0.0029352	0.0201445	Rasl12	9	65398506	65414853	+	6568	protein_coding	RAS-like, family 12 [Source:MGI Symbol;Acc:MGI:1918034]	-
ENSMUSG0000002228	897.8836416	840.5070629	786.4796792	1095.613141	1148.63507	1213.362716	841.6234612	1152.536976	-0.453171478	0.0029359	0.0201445	Rnf19a	15	36239933	36283147	-	4434	protein_coding	ring finger protein 19A [Source:MGI Symbol;Acc:MGI:1353623]	-
ENSMUSG0000003064	150.3153418	85.31462668	126.2389258	183.4635212	270.6156571	226.7149261	120.6229648	226.9313681	-0.910408201	0.0029436	0.0201895	Rab30	7	92741603	92844535	+	9642	protein_coding	RAB30, member RAS oncogene family [Source:MGI Symbol;Acc:MGI:1923235]	-
ENSMUSG0000007160	712.4947201	565.6043769	784.2453619	540.0545907	311.1092409	327.9095712	687.448153	393.0244676	0.805506798	0.0029447	0.0201899	Fam189a2	19	23972571	24031019	-	2547	protein_coding	family with sequence similarity 189, member A2 [Source:MGI Symbol;Acc:MGI:2685813]	-
ENSMUSG0000003845	216.4540922	179.0553893	153.050733	105.9437235	115.5548609	93.41044164	182.8534048	104.9696754	0.801773412	0.0029481	0.0201999	Tmem255b	8	13435189	13468473	+	1581	protein_coding	transmembrane protein 255B [Source:MGI Symbol;Acc:MGI:2685533]	-
ENSMUSG0000002067	754.5830158	763.6185722	889.2582736	596.9024423	561.9719304	550.7323955	802.4866205	569.8689227	0.493311097	0.0029483	0.0201999	Ddx52	11	83942062	83963088	+	3332	protein_coding	DEAD (Asp-Glu-Ala-Asp) box polypeptide 52 [Source:MGI Symbol;Acc:MGI:1925644]	-
ENSMUSG0000002232	1.002102279	0	3.351475906	22.39460823	8.888835454	17.51445781	1.451192728	16.26596716	-3.513311033	0.0029505	0.0202075	Shcbp1	8	4735976	4779567	-	6295	protein_coding	Shc SH2-domain binding protein 1 [Source:MGI Symbol;Acc:MGI:1338802]	-
ENSMUSG0000000495	2026.250807	3852.850672	4202.750786	2166.24768	1909.124326	1611.330118	3360.617422	1895.567375	0.825930308	0.0029533	0.0202194	Hspb1	5	135887919	135889563	+	1031	protein_coding	heat shock protein 1 [Source:MGI Symbol;Acc:MGI:96240]	-
ENSMUSG0000004498	397.8346046	269.636351	375.3653014	224.8074133	211.3567541	208.2274428	347.6120857	214.7972034	0.69428082	0.0029586	0.020248	Sft2d3	18	31909094	31911903	-	2810	protein_coding	SFT2 domain containing 3 [Source:MGI Symbol;Acc:MGI:1914408]	-
ENSMUSG0000003817	47.0988071	30.54474289	45.80350404	108.5277168	64.19714494	107.0327977	41.14901801	93.25255315	-1.18136498	0.0029608	0.0202559	Slamf7	1	171632403	171653035	-	4577	protein_coding	SLAM family member 7 [Source:MGI Symbol;Acc:MGI:1922595]	-
ENSMUSG0000005997	6.012613672	6.319601977	0	29.28525691	13.82707737	41.84009365	4.110738549	28.31747598	-2.771006916	0.002963	0.0202636	Ntm	9	28994750	29963141	-	8079	protein_coding	neurotrimin [Source:MGI Symbol;Acc:MGI:2446259]	-
ENSMUSG0000005968	568.191992	557.1782409	680.3496088	403.1029481	419.7505631	427.1581654	601.9066139	416.6705589	0.530426331	0.0029655	0.0202734	Zfp637	6	117841180	117845956	+	2565	protein_coding	zinc finger protein 637 [Source:MGI Symbol;Acc:MGI:2448537]	zf-C2
ENSMUSG0000010765	271.5697175	324.4062348	237.9547893	186.0475145	175.8014123	130.3854081	277.9769139	164.0781116	0.760028068	0.0029687	0.0202874	Gm44220	6	93241720	93244206	+	2487	TEC	predicted gene, 44220 [Source:MGI Symbol;Acc:MGI:5690612]	-
ENSMUSG0000002813	73.15346634	42.13067984	44.68634541	252.3700081	58.27125464	153.7380185	53.3234972	154.7930938	-1.537499044	0.0029735	0.020313	Tmem56	3	121201761	121283098	-	7874	protein_coding	transmembrane protein 56 [Source:MGI Symbol;Acc:MGI:1923195]	-
ENSMUSG0000003236	279.5865357	255.9438801	264.7665965	202.4128051	84.937761	97.30254338	266.7656708	128.2177032	1.054213814	0.0029759	0.0203219	Plscr1	9	92249750	92272278	+	2462	protein_coding	phospholipid scramblase 1 [Source:MGI Symbol;Acc:MGI:893575]	-
ENSMUSG0000003689	196.4120466	155.8835154	187.6826507	322.9991571	239.9985573	322.0714186	179.9927376	295.0230443	-0.71330524	0.0029826	0.0203472	Rap2b	3	61361638	61368430	+	6793	protein_coding	RAP2B, member of RAS oncogene family [Source:MGI Symbol;Acc:MGI:1921262]	-
ENSMUSG0000002261	478.0027869	397.0816575	526.1817172	255.8153324	348.6398795	282.1773758	467.0887205	295.5441959	0.660918712	0.0029828	0.0203472	Mapk12	15	89130584	89140703	-	3805	protein_coding	mitogen-activated protein kinase 12 [Source:MGI Symbol;Acc:MGI:1353438]	-
ENSMUSG0000002697	2039.278137	2146.558138	2483.443646	1358.319122	1692.82933	1638.57483	2223.093307	1563.241094	0.50813836	0.0029829	0.0203472	7-Mar	2	60209887	60250676	+	10391	protein_coding	membrane-associated ring finger (C3HC4) 7 [Source:MGI Symbol;Acc:MGI:1931053]	-
ENSMUSG0000005291	4306.033491	3767.536045	3250.931628	6475.487101	4718.983978	5553.056151	3774.833722	5582.509077	-0.56448911	0.0029908	0.0203912	Lamb2	9	108479736	108490530	+	6082	protein_coding	laminin, beta 2 [Source:MGI Symbol;Acc:MGI:99916]	-
ENSMUSG0000002806	210.4414785	183.2684573	248.009217	124.8930074	136.295477	127.4663318	213.9063843	129.5516054	0.723265908	0.0029915	0.0203912	Gpatch4	3	88043108	88055993	+	2447	protein_coding	G patch domain containing 4 [Source:MGI Symbol;Acc:MGI:1913864]	-
ENSMUSG0000000914	42.0882957	40.02414585	140.761988	20.67194605	27.65415474	15.56840694	74.29147653	21.29816925	1.801026875	0.0029974	0.0204242	Dqx1	6	83057844	83067318	+	3419	protein_coding	DEAQ RNA-dependent ATPase [Source:MGI Symbol;Acc:MGI:2136388]	-
ENSMUSG0000003487	40.08409114	20.01207293	15.64022089	62.01583816	60.24655141	85.62623817	25.24546165	69.29620925	-1.450780183	0.0029997	0.0204319	Gipc3	10	81335265	81343266	-	3521	protein_coding	GIPC PDZ domain containing family, member 3 [Source:MGI Symbol;Acc:MGI:2387006]	-
ENSMUSG0000003603	100.2102279	114.8061026	161.9880021	55.12518948	77.03657393	52.54337342	125.6681108	61.56837894	1.029368794	0.0030041	0.0204547	Zfp57	17	37001163	37010635	+	3786	protein_coding	zinc finger protein 57 [Source:MGI Symbol;Acc:MGI:99204]	zf-C2
ENSMUSG0000003219	10.02102279	8.426135969	32.39760042	82.68778422	40.49358373	59.35455146	16.94825306	60.84530647	-1.852782943	0.0030075	0.0204702	Bcl2l10	9	75347758	75351632	+	1220	protein_coding	Bcl2-like 10 [Source:MGI Symbol;Acc:MGI:1330841]	-
ENSMUSG0000006611	326.6853428	262.263482	306.1014661	224.8074133	150.1225543	134.2775099	298.350097	169.7358258	0.811853125	0.0030105	0.0204832	Gm10154	4	83505185	83505538	-	354	processed_pseudog	predicted gene 10154 [Source:MGI Symbol;Acc:MGI:3642271]	-
ENSMUSG0000003769	452.9502299	434.9992694	354.1392874	724.379443	652.8355817	533.2179377	414.0295956	636.8109874	-0.620989704	0.0030128	0.0204913	Ahdc1	4	133011260	133078110	+	8444	protein_coding	AT hook, DNA binding motif, containing 1 [Source:MGI Symbol;Acc:MGI:2444218]	-
ENSMUSG0000003202	1120.350347	1130.155487	1314.895714	1469.430832	1746.162342	1862.37068	1188.467183	1692.654618	-0.510164463	0.0030163	0.0205021	Rexo2	9	48468512	48480623	-	3385	protein_coding	RNA exonuclease 2 [Source:MGI Symbol;Acc:MGI:1888981]	-
ENSMUSG0000005208	59.12403444	36.86434486	42.45202814	94.74641942	106.6660254	85.62623817	46.14680248	95.67956101	-1.049705689	0.0030165	0.0205021	Rgs14	13	55369732	55384687	+	4239	protein_coding	regulator of G-protein signaling 14 [Source:MGI Symbol;Acc:MGI:1859709]	-
ENSMUSG0000008635	28.0588638	14.74573795	24.57748997	6.029317599	4.938241919	1.946050868	22.46069724	4.304536795	2.375049159	0.0030224	0.0205276	Gm13270	2	13271601	13325827	+	4357	processed_transcrip	predicted gene 13270 [Source:MGI Symbol;Acc:MGI:3651704]	-
ENSMUSG0000003837	61.128239	77.94175771	20.10885543	448.7534956	96.78954161	78.81506014	53.05961738	208.1193658	-1.971429272	0.0030225	0.0205276	Pcp4l1	1	171173262	171196268	-	1577	protein_coding	Purkinje cell protein 4-like 1 [Source:MGI Symbol;Acc:MGI:1913675]	-
ENSMUSG0000009772	308.6475018	203.2805302	389.8883637	156.7622576	163.9496317	177.0906289	300.6054653	165.9341727	0.857070203	0.0030294	0.020561	2310015A10Ri	12	80120546	80132844	-	2330	processed_transcrip	RIKEN cDNA 2310015A10 gene [Source:MGI Symbol;Acc:MGI:1916798]	-
ENSMUSG0000004207	2476.19473	2462.538237	2560.527592	2054.274639	1725.421726	1761.176035	2499.75352	1846.957467	0.436385218	0.0030303	0.020561	Hnrnpf	6	117900324	117925622	+	3047	protein_coding	heterogeneous nuclear ribonucleoprotein F [Source:MGI Symbol;Acc:MGI:2138741]	-
ENSMUSG0000006229	7.01471595	1.053266996	0	310.9405219	7.90118707	35.02891562	2.689327649	117.9568749	-5.445336986	0.0030307	0.020561	Trank1	9	111311739	111395775	+	13123	protein_coding	tetratricopeptide repeat and ankyrin repeat containing 1 [Source:MGI Symbol;Acc:MGI:1341834]	-
ENSMUSG0000003292	244.512956	220.1328022	208.9086648	762.2780107	305.1833506	336.6668001	224.518141	468.0427205	-1.06045398	0.0030341	0.0205768	Itgbl1	14	123659971	123975618	+	4229	protein_coding	integrin, beta-like 1 [Source:MGI Symbol;Acc:MGI:2443439]	-
ENSMUSG0000002955	1.002102279	0	0	67.18382468	0	2.919076301	0.334034093	23.36763366	-5.95045713	0.0030367	0.0205779	Hnf1a	5	114948980	114971094	-	5032	protein_coding	HNF1 homeobox A [Source:MGI Symbol;Acc:MGI:98504]	Hom
ENSMUSG0000009802	28.0588638	53.7166168	99.42711853	16.36529063	16.79002252	22.37958498	60.40086638	18.51163271	1.704863093	0.003037	0.0205779	Gm27003	2	126675916	126683805	+	497	antisense	predicted gene, 27003 [Source:MGI Symbol;Acc:MGI:5504118]	-
ENSMUSG0000010786	77.16187545	106.3799666	55.85793176	28.42392582	30.6170999	41.84009365	79.79992461	33.62703979	1.249341696	0.0030385	0.0205779	Gm5112	6	122811670	122813537	+	2368	TEC	predicted gene 5112 [Source:MGI Symbol;Acc:MGI:3779460]	-
ENSMUSG0000002610	3523.391612	2352.998469	3577.14195	4260.143549	4680.465691	6548.461169	3151.177344	5163.02347	-0.712294235	0.0030396	0.0205779	Nabp1	1	51465862	51478425	-	8498	protein_coding	nucleic acid binding protein 1 [Source:MGI Symbol;Acc:MGI:1923258]	-
ENSMUSG0000003707	4484.407697	3781.228516	5106.532122	5826.043463	6445.393352	6685.657755	4457.389445	6319.031524	-0.503518098	0.0030398	0.0205779	Selenof	3	144570304	144597680	+	5819	protein_coding	selenoprotein F [Source:MGI Symbol;Acc:MGI:1927947]	-
ENSMUSG0000002790	2222.662854	1088.024807	3550.330143	4210.186346	4572.812017	5294.231385	2287.005935	4692.409916	-1.036943969	0.0030415	0.0205824	S100a11	3	93520488	93526287	+	511	protein_coding	S100 calcium binding protein A11 [Source:MGI Symbol;Acc:MGI:1338798]	-
ENSMUSG0000008657	13.02732962	11.58593696	31.28044179	0	1.975296767	3.892101735	18.63123612	1.955799501	3.275929895	0.0030461	0.0206055	Gm14104	2	132845999	132847256	-	356	antisense	predicted gene 14104 [Source:MGI Symbol;Acc:MGI:3651500]	-
ENSMUSG0000002088	3733.83309	3433.650407	697.1069884	818.2645313	463.207092	551.705421	2621.530162	611.0590147	2.100966605	0.0030511	0.0206255	Nr1d1	11	98767932	98775333	-	3077	protein_coding	nuclear receptor subfamily 1, group D, member 1 [Source:MGI Symbol;Acc:MGI:2444210]	THR
ENSMUSG0000007501	1.002102279	13.69247095	13.40590362	0	0	0	9.366825617	0	5.759024062	0.0030512	0.0206255	Gm10800	2	98666547	98667301	-	660	protein_coding	predicted gene 10800 [Source:MGI Symbol;Acc:MGI:3641657]	-
ENSMUSG0000003340	25.05255697	33.70454388	21.22601407	70.62914902	57.28360626	60.32757689	26.6610383	62.74677739	-1.234582645	0.0030533	0.0206322	Nudt15	14	73518877	73548242	-	3579	protein_coding	nudix (nucleoside diphosphate linked moiety X)-type motif 15 [Source:MGI Symbol;Acc:MGI:2443366]	-
ENSMUSG0000009735	122.256478	121.1257046	192.1512853	83.5491153	67.16009046	73.94993297	145.1778226	74.88637945	0.952515394	0.0030557	0.0206409	2310001H17Ri	6	129208412	129238725	-	7241	lincRNA	RIKEN cDNA 2310001H17 gene [Source:MGI Symbol;Acc:MGI:1923682]	-
ENSMUSG0000009014	83.17448913	42.13067984	52.50645586	95.6077505	128.3942899	179.0366798	59.27054161	134.3462401	-1.17762071	0.0030586	0.0206533	Ugt1a6b	1	88103252	88219003	+	2382	protein_coding	UDP glucuronosyltransferase 1 family, polypeptide A6B [Source:MGI Symbol;Acc:MGI:3580629]	-
ENSMUSG0000002737	2.004204557	4.213067984	4.468634541	25.83993257	13.82707737	20.43353411	3.561969028	20.03351468	-2.502208538	0.00306	0.0206552	Bub1	2	127801122	127831865	-	4354	protein_coding	BUB1, mitotic checkpoint serine/threonine kinase [Source:MGI Symbol;Acc:MGI:1100510]	-
ENSMUSG0000003860	500.049037	439.2123374	452.4492473	549.5292326	843.4517197	806.6380846	463.9035406	733.2063456	-0.659672796	0.0030621	0.0206615	Dock10	1	80501073	80758527	-	18267	protein_coding	dedicator of cytokinesis 10 [Source:MGI Symbol;Acc:MGI:2146320]	-
ENSMUSG0000002209	405.8514228	1558.835154	2592.925192	374.6790222	389.1334632	311.3681388	1519.203923	358.3935414	2.083607527	0.0030725	0.0207241	Slc39a14	14	70303469	70351425	-	10550	protein_coding	solute carrier family 39 (zinc transporter), member 14 [Source:MGI Symbol;Acc:MGI:2384851]	-
ENSMUSG0000002677	12.02522734	10.53266996	16.75737953	50.81853405	35.55534181	33.08286475	13.10509228	39.81891354	-1.608779127	0.0030793	0.020763	Prkcq	2	11172108	11301222	+	7081	protein_coding	protein kinase C, theta [Source:MGI Symbol;Acc:MGI:97601]	-
ENSMUSG0000004394	407.8556274	325.4595018	242.4234238	479.7614147	617.2802398	527.3797851	325.2461843	541.4738132	-0.734099769	0.0030806	0.0207638	Naalad2	9	18321951	18402995	-	4749	protein_coding	N-acetylated alpha-linked acidic dipeptidase 2 [Source:MGI Symbol;Acc:MGI:1919810]	-
ENSMUSG0000009108	1069.243131	1196.511308	1387.511025	866.4990721	680.4897364	900.0485262	1217.755155	815.6791116	0.577731032	0.0030868	0.0207983	Rpl6l	10	111125851	111126721	+	871	processed_pseudog	ribosomal protein L6-like [Source:MGI Symbol;Acc:MGI:3647789]	-
ENSMUSG0000002639	91.19130735	280.169021	109.4815463	74.07447336	53.33301272	58.38152603	160.2806249	61.9296707	1.370686512	0.0030926	0.0208282	Nr5a2	1	136842571	136960448	-	5899	protein_coding	nuclear receptor subfamily 5, group A, member 2 [Source:MGI Symbol;Acc:MGI:1346834]	SF-lil
ENSMUSG0000001989	1.002102279	1.053266996	0	71.4904801	0.987648384	1.946050868	0.685123092	24.8080597	-5.161015855	0.0030934	0.0208282	Lrriq1	10	103046031	103236322	-	6449	protein_coding	leucine-rich repeats and IQ motif containing 1 [Source:MGI Symbol;Acc:MGI:1922228]	-
ENSMUSG0000011428	20.04204557	5.26633498	23.46033134	0.861331086	0	2.919076301	16.2562373	1.260135796	3.694547884	0.0030976	0.0208485	9630015K15Ri	14	64116314	64127605	+	572	antisense	RIKEN cDNA 9630015K15 gene [Source:MGI Symbol;Acc:MGI:1925819]	-
ENSMUSG0000010727	31.0657064	28.43820889	22.3431727	5.167986514	8.888835454	7.78420347	27.28218408	7.280341812	1.91407547	0.0031093	0.0209196	Gm10461	5	32607020	32611805	-	4786	TEC	predicted gene 10461 [Source:MGI Symbol;Acc:MGI:3642164]	-
ENSMUSG0000002296	842.7680163	915.2890196	920.5387154	1124.037067	1309.621757	1238.661377	892.8652504	1224.106734	-0.455129519	0.0031107	0.0209215	Tmem50b	16	91574503	91597800	-	2446	protein_coding	transmembrane protein 50B [Source:MGI Symbol;Acc:MGI:1925225]	-
ENSMUSG0000000350	1000.098074	1647.309582	1274.678003	776.0593081	843.4517197	879.6149921	1307.361886	833.0420067	0.650215519	0.0031286	0.0210299	Impdh1	6	29200434	29216364	-	3294	protein_coding	inosine monophosphate dehydrogenase 1 [Source:MGI Symbol;Acc:MGI:96567]	-
ENSMUSG0000003963	442.9292071	511.8877601	470.3237854	746.7740512	657.7738236	632.466532	475.0469176	679.0048022	-0.51586304	0.0031291	0.0210299	Coro7	16	4626133	4679777	-	7423	protein_coding	coronin 7 [Source:MGI Symbol;Acc:MGI:1926135]	-
ENSMUSG0000002669	602.2634694	555.0717069	530.6503517	374.6790222	392.0964083	420.3469874	562.6618427	395.7074727	0.508455399	0.0031309	0.0210299	Vamp4	1	162570515	162599084	+	4565	protein_coding	vesicle-associated membrane protein 4 [Source:MGI Symbol;Acc:MGI:1858730]	-
ENSMUSG0000002499	608.2760831	381.2826526	470.3237854	727.8247673	731.8474554	853.3433054	486.627507	771.005175	-0.663425158	0.0031319	0.0210299	Plce1	19	38481109	38785030	+	15998	protein_coding	phospholipase C, epsilon 1 [Source:MGI Symbol;Acc:MGI:1921305]	-
ENSMUSG0000002669	42.0882957	90.58096166	79.3182631	28.42392582	25.67885798	30.16378845	70.66250682	28.08885742	1.329235142	0.0031324	0.0210299	Fmo4	1	162793188	162813972	-	3707	protein_coding	flavin containing monooxygenase 4 [Source:MGI Symbol;Acc:MGI:2429497]	-
ENSMUSG0000003435	65.13664811	49.50354882	50.27213858	192.0768321	94.81424484	91.46439078	54.9707785	126.1184892	-1.199091787	0.0031357	0.0210445	Ramp1	1	91179822	91225196	+	3022	protein_coding	receptor (calcitonin) activity modifying protein 1 [Source:MGI Symbol;Acc:MGI:1858418]	-
ENSMUSG0000001919	1048.198983	2866.992763	650.1863257	656.3342872	624.1937785	604.2487944	1521.792691	628.2589534	1.276359169	0.0031447	0.0210974	Scn1b	7	31116524	31127003	-	1859	protein_coding	sodium channel, voltage-gated, type I, beta [Source:MGI Symbol;Acc:MGI:98247]	-
ENSMUSG0000000032	206.4330694	150.6171804	170.9252712	358.3137316	295.3068667	241.3103076	159.9918403	298.310302	-0.761497274	0.0031467	0.0211033	Arvcf	16	18348182	18407076	+	6715	protein_coding	armadillo repeat gene deleted in velocardiofacial syndrome [Source:MGI Symbol;Acc:MGI:109620]	-
ENSMUSG0000004351	217.4561945	167.4694524	254.7121688	330.7511369	328.8869118	377.5338683	213.2126052	345.7239723	-0.697798741	0.0031542	0.0211393	Rai2	X	161717069	161779496	+	2403	protein_coding	retinoic acid induced 2 [Source:MGI Symbol;Acc:MGI:1344378]	-
ENSMUSG0000002370	567.1898897	422.3600654	483.7296891	354.8684073	278.5168442	320.1253677	491.0932147	317.8368731	0.627299256	0.0031543	0.0211393	Ogfod2	5	124112297	124115483	+	2260	protein_coding	2-oxoglutarate and iron-dependent oxygenase domain containing 2 [Source:MGI Symbol;Acc:MGI:1913877]	-
ENSMUSG0000005908	51.10721621	144.2975785	79.3182631	36.17590559	12.83942899	38.92101735	91.57435259	29.31211731	1.64067305	0.0031556	0.0211398	Fcgr4	1	171018920	171029761	+	1256	protein_coding	Fc receptor, IgG, low affinity IV [Source:MGI Symbol;Acc:MGI:2179523]	-

ENSMUSG0000007307	101.2123301	77.94175771	97.19280126	24.1172704	45.43182565	51.57034799	92.11562971	40.37314801	1.194678911	0.0031568	0.0211403	Srp54a	12	55080262	55115367	+	5325	protein_coding	signal recognition particle 54A [Source:MGI Symbol;Acc:MGI:1346087]	-
ENSMUSG0000003416	11.02312506	12.63920395	7.820110447	74.07447336	23.70356121	27.24471215	10.49414649	41.67424891	-1.990964426	0.003158	0.021141	Emid1	11	5106265	5152257	-	2890	protein_coding	EMI domain containing 1 [Source:MGI Symbol;Acc:MGI:2155091]	-
ENSMUSG0000005442	1.002102279	6.319601977	22.3431727	0	0	0	9.88829232	0	5.836007841	0.0031595	0.0211437	Fabp1	6	71199827	71205023	+	553	protein_coding	fatty acid binding protein 1, liver [Source:MGI Symbol;Acc:MGI:95479]	-
ENSMUSG0000002080	4.008409114	4.213067984	4.468634541	32.73058125	8.888835454	30.16378845	4.230037213	23.92773505	-2.503529653	0.0031634	0.0211619	Pimreg	11	72042032	72047370	+	2529	protein_coding	PICALM interacting mitotic regulator [Source:MGI Symbol;Acc:MGI:1924434]	-
ENSMUSG0000003465	252.5297742	195.9076613	241.3062652	130.0609939	133.3325318	157.6301203	229.9145669	140.3412153	0.712855175	0.0031672	0.0211735	Cd300a	11	114890041	114904654	+	4738	protein_coding	CD300A molecule [Source:MGI Symbol;Acc:MGI:2443411]	-
ENSMUSG0000003579	331.6958542	241.1981421	256.9464861	409.1322657	423.7011566	444.6726232	276.6134941	425.8353485	-0.621484671	0.0031674	0.0211735	Twist1	12	33957671	33959829	+	1628	protein_coding	twist basic helix-loop-helix transcription factor 1 [Source:MGI Symbol;Acc:MGI:98872]	bHLH
ENSMUSG0000002136	146.3069327	123.2322385	136.2933535	204.9967984	206.4185122	245.2024093	135.2775082	218.8725733	-0.693485826	0.0031708	0.021189	Tbc1d7	13	43151740	43171501	-	4042	protein_coding	TBC1 domain family, member 7 [Source:MGI Symbol;Acc:MGI:1914296]	-
ENSMUSG0000004157	4083.566785	2842.767622	5185.850385	5821.736808	6360.455591	7654.791088	4037.394931	6612.327829	-0.711761477	0.0031767	0.0212209	Selenow	7	15917208	15922402	-	736	protein_coding	selenoprotein W [Source:MGI Symbol;Acc:MGI:1100878]	-
ENSMUSG0000002855	2014.22558	1928.53187	1931.56728	2419.479019	2625.169404	2694.307426	1958.108243	2579.65195	-0.397569309	0.003186	0.0212754	Eps15	4	109280268	109387817	+	6931	protein_coding	epidermal growth factor receptor pathway substrate 15 [Source:MGI Symbol;Acc:MGI:104583]	-
ENSMUSG0000003527	93.19551191	81.1015587	39.10055223	134.3676494	151.1102027	172.2255018	71.13254095	152.5677846	-1.09754917	0.0031884	0.0212838	Hpse	5	100679484	100719716	-	3516	protein_coding	heparanase [Source:MGI Symbol;Acc:MGI:1343124]	-
ENSMUSG0000002840	1454.050406	1310.264143	1444.486115	1588.294522	2510.602191	2417.968203	1402.933555	2172.288305	-0.630551237	0.0031991	0.0213473	Mpdz	4	81278500	81442815	-	12437	protein_coding	multiple PDZ domain protein [Source:MGI Symbol;Acc:MGI:1343489]	-
ENSMUSG0000002499	488.0238097	394.9751235	602.1485044	684.758213	803.9457844	822.2064915	495.0491459	770.3034963	-0.638022395	0.0032034	0.0213684	Fam45a	19	60811585	60836227	+	6242	protein_coding	family with sequence similarity 45, member A [Source:MGI Symbol;Acc:MGI:1915144]	-
ENSMUSG0000008621	20.04204557	16.85227194	17.87453816	46.51187862	34.56769343	74.9229584	18.25628522	52.00084348	-1.508952544	0.0032064	0.0213809	A330040F15Ri	19	12517915	12596566	-	2323	processed_transcrip	RIKEN cDNA A330040F15 gene [Source:MGI Symbol;Acc:MGI:3698434]	-
ENSMUSG0000002429	798.6755161	707.7954214	763.0193479	1193.804885	1362.95477	897.1294499	756.4967618	1151.296368	-0.605825911	0.0032131	0.0214182	Adamts10	17	33524204	33553782	+	5453	protein_coding	a disintegrin-like and metallopeptidase (reprolysin type) with thrombospondin type 1 motif, 10 [Source:MGI Symbol;Acc:MGI:244	-
ENSMUSG0000003164	61.128239	43.18394684	45.80350404	236.8660485	79.99951908	86.59926361	50.03856329	134.4882771	-1.427459308	0.0032169	0.0214359	Mfap3l	8	60632827	60676729	+	6837	protein_coding	microfibrillar-associated protein 3-like [Source:MGI Symbol;Acc:MGI:1918556]	-
ENSMUSG0000006384	610.2802877	518.2073621	585.3911249	447.8921645	320.9857247	350.2891562	571.2929249	373.0556818	0.613893985	0.0032254	0.0214848	Ppcdc	9	57385095	57440124	-	5803	protein_coding	phosphopantothenoylcysteine decarboxylase [Source:MGI Symbol;Acc:MGI:1914062]	-
ENSMUSG0000005470	234.4919332	186.4282583	222.3145684	131.7836561	129.3819383	138.1696116	214.4115866	133.1117353	0.687955127	0.0032269	0.0214871	Ankrd24	10	81628540	81647610	+	8142	protein_coding	ankyrin repeat domain 24 [Source:MGI Symbol;Acc:MGI:1890394]	-
ENSMUSG0000003220	19.03994329	9.479402965	33.51475906	210.1647849	40.49358373	34.05589018	20.67803511	94.90475293	-2.202678103	0.0032372	0.0215481	Aqp9	9	71110659	71168682	-	4143	protein_coding	aquaporin 9 [Source:MGI Symbol;Acc:MGI:1891066]	-
ENSMUSG0000003291	366.769434	448.6917403	365.3108737	455.6441443	772.3410361	770.6361436	393.5906827	666.207108	-0.758671888	0.0032519	0.0216379	Cspg4	9	56865033	56899870	+	11931	protein_coding	chondroitin sulfate proteoglycan 4 [Source:MGI Symbol;Acc:MGI:2153093]	-
ENSMUSG0000006227	2417.070696	2630.007689	2656.603235	2128.349112	1675.051659	1518.892702	2567.893873	1774.097824	0.533194059	0.0032539	0.0216437	Morf4l1	9	90091665	90114774	-	9754	protein_coding	mortality factor 4 like 1 [Source:MGI Symbol;Acc:MGI:1096551]	-
ENSMUSG0000005044	5.010511393	7.372868973	2.23431727	30.146588	23.70356121	17.51445781	4.872565879	23.78820234	-2.282615336	0.0032573	0.0216587	Lypd6	2	50066429	50193569	+	3729	protein_coding	LY6/PLAUR domain containing 6 [Source:MGI Symbol;Acc:MGI:2443848]	-
ENSMUSG0000005176	544.1415373	524.5269641	508.307179	419.4682387	288.3933281	307.4760371	525.6585601	338.4458679	0.634093465	0.0032613	0.0216776	Xrcc1	7	24546289	24573440	+	5831	protein_coding	X-ray repair complementing defective repair in Chinese hamster cells 1 [Source:MGI Symbol;Acc:MGI:99137]	-
ENSMUSG0000001834	6590.826686	4522.728481	6721.943508	8878.60083	8060.19846	10085.40862	5945.166225	9008.069304	-0.599508413	0.0032723	0.0217432	Anxa6	11	54979108	55033445	-	2930	protein_coding	annexin A6 [Source:MGI Symbol;Acc:MGI:88255]	-
ENSMUSG0000004651	171.3594896	152.7237144	252.4778516	92.16242616	90.8636513	123.5742301	192.1870185	102.2001025	0.910789478	0.003275	0.0217474	Cox17	16	38346991	38352763	+	5773	protein_coding	cytochrome c oxidase assembly protein 17, copper chaperone [Source:MGI Symbol;Acc:MGI:1333806]	-
ENSMUSG0000003730	988.0728467	803.642718	799.8855828	1172.271607	1160.486851	1509.162448	863.8670492	1280.640302	-0.567621573	0.0032753	0.0217474	Ttc13	8	124671332	124721983	-	10286	protein_coding	tetratricopeptide repeat domain 13 [Source:MGI Symbol;Acc:MGI:2384573]	-
ENSMUSG0000003938	510.0700598	522.4204301	609.9686148	396.2122994	367.4051988	377.5338683	547.4863682	380.3837888	0.5246686	0.0032832	0.021792	Wdr45	X	7714333	7728206	+	3310	protein_coding	WD repeat domain 45 [Source:MGI Symbol;Acc:MGI:1859606]	-
ENSMUSG0000006791	69.14505722	82.1548257	108.3643876	168.8208928	140.2460705	162.4952474	86.55475685	157.1874036	-0.863556034	0.0032939	0.0218501	Zfp991	4	147132038	147180579	+	2917	protein_coding	zinc finger protein 991 [Source:MGI Symbol;Acc:MGI:3701604]	zf-C2
ENSMUSG0000005238	34.07147747	35.81107787	39.10055223	91.30109507	60.24655141	82.70716187	36.32770252	78.08493612	-1.106403141	0.0032943	0.0218501	Trpm3	19	22139119	22989884	+	8061	protein_coding	transient receptor potential cation channel, subfamily M, member 3 [Source:MGI Symbol;Acc:MGI:2443101]	-
ENSMUSG0000003081	1271.667792	1432.443115	1158.493505	987.9467552	913.574755	841.6670002	1287.534804	914.3961701	0.493594234	0.0032963	0.021856	Rnf40	7	127588767	127604799	+	5621	protein_coding	ring finger protein 40 [Source:MGI Symbol;Acc:MGI:2142048]	-
ENSMUSG0000000193	5073.643837	6130.013917	2910.198245	3532.318782	1887.396061	1535.434135	4704.618666	2318.382993	1.020845593	0.003299	0.021866	Vwf	6	125546774	125686679	+	16064	protein_coding	Von Willebrand factor [Source:MGI Symbol;Acc:MGI:98941]	-
ENSMUSG0000002052	1071.247336	1106.983613	1196.476898	810.5125515	845.4270165	846.5321274	1124.902616	834.1572318	0.431353186	0.0033025	0.0218812	Rnft1	11	86484657	86499025	+	4719	protein_coding	ring finger protein, transmembrane 1 [Source:MGI Symbol;Acc:MGI:1924142]	-
ENSMUSG0000000820	951.9971647	995.3373113	1060.183545	1472.876156	1238.511073	1429.374362	1002.506007	1380.253864	-0.461667702	0.0033093	0.0219113	Cers4	8	4493026	4531680	+	12264	protein_coding	ceramide synthase 4 [Source:MGI Symbol;Acc:MGI:1914510]	Hom
ENSMUSG0000002784	256.5381833	399.1881915	253.5950102	149.8716089	174.8137639	195.5781122	303.1071283	173.4211617	0.806393146	0.0033093	0.0219113	Dclre1b	3	103800605	103809444	-	8405	protein_coding	DNA cross-link repair 1B [Source:MGI Symbol;Acc:MGI:2156057]	-
ENSMUSG0000003336	1160.434439	1043.787593	1832.140162	2491.830831	1833.0754	2439.374762	1345.454065	2254.760331	-0.745179374	0.0033139	0.0219333	Prrg3	X	71962624	71972722	+	6087	protein_coding	proline rich Gla (G-carboxyglutamic acid) 3 (transmembrane) [Source:MGI Symbol;Acc:MGI:2685214]	-
ENSMUSG0000011282	137.2880122	140.0845105	207.7915062	74.93580445	82.96246423	101.1946451	161.7213429	86.3643046	0.904970796	0.0033241	0.0219936	Gm9118	10	56497341	56498094	+	754	processed_pseudog	predicted gene 9118 [Source:MGI Symbol;Acc:MGI:3648185]	-
ENSMUSG0000003306	1023.146426	1218.629914	836.7518178	708.0141524	703.2056492	657.7651932	1026.176053	689.6616649	0.573448634	0.0033306	0.0220272	Lmo7	14	101729957	101934710	+	11951	protein_coding	LIM domain only 7 [Source:MGI Symbol;Acc:MGI:1353586]	-
ENSMUSG0000002033	38.07988659	22.11860692	36.86623496	67.18382468	70.12303525	82.70716187	32.35490949	73.33800726	-1.179988349	0.0033316	0.0220272	Hcn2	10	79716634	79736108	+	3195	protein_coding	hyperpolarization-activated, cyclic nucleotide-gated K+ 2 [Source:MGI Symbol;Acc:MGI:1298210]	-
ENSMUSG0000000597	817.7154594	438.1590704	746.2619683	1366.071102	936.2906678	1176.387749	667.3788327	1159.583173	-0.797153372	0.0033328	0.0220277	Rcn1	2	105386291	105399319	-	3196	protein_coding	reticulocalbin 1 [Source:MGI Symbol;Acc:MGI:104559]	-
ENSMUSG0000007559	2421.079105	2815.382681	2676.71209	2136.962423	1837.025994	1641.493907	2637.724625	1871.827441	0.494598642	0.0033359	0.0220402	Nrbp2	15	76085518	76091627	-	3894	protein_coding	nuclear receptor binding protein 2 [Source:MGI Symbol;Acc:MGI:2385017]	-
ENSMUSG0000005469	1220.560575	2147.611405	1206.531326	956.9388361	894.8094357	871.8307887	1524.901102	907.8596868	0.748124582	0.0033412	0.0220677	Emcn	3	137341067	137432185	+	5409	protein_coding	endomucin [Source:MGI Symbol;Acc:MGI:1891716]	-
ENSMUSG0000002815	162.3405691	136.9247095	272.586707	117.1410276	85.92540939	64.21967863	190.6173285	89.09537189	1.094474254	0.0033537	0.0221422	Mttp	3	138089854	138144968	-	6206	protein_coding	microsomal triglyceride transfer protein [Source:MGI Symbol;Acc:MGI:106926]	-
ENSMUSG0000000124	1401.941088	2297.175318	1515.984268	1161.935634	1133.820345	972.0524083	1738.366891	1089.269462	0.6742794	0.0033557	0.0221479	Gramd1a	7	31130127	31155896	-	5239	protein_coding	GRAM domain containing 1A [Source:MGI Symbol;Acc:MGI:105490]	-
ENSMUSG0000004026	197.4141489	488.7158862	261.4151206	148.1489467	158.0237414	159.5761711	315.8483852	155.2496198	1.024683786	0.0033624	0.022184	Dnm3	1	161982453	162478034	-	24452	protein_coding	dynamin 3 [Source:MGI Symbol;Acc:MGI:1341299]	-
ENSMUSG0000001652	602.2634694	793.1100481	725.0359543	998.2827282	1010.364297	1009.027375	706.8031573	1005.891467	-0.509359407	0.0033733	0.0222473	Lnx2	5	147016655	147076586	-	4557	protein_coding	ligand of numb-protein X 2 [Source:MGI Symbol;Acc:MGI:2155959]	-
ENSMUSG0000002214	1744.660067	2785.891205	2899.026658	916.4562751	1875.544281	1271.744242	2476.525977	1354.581599	0.870623023	0.0033744	0.0222473	Osmr	15	6813577	6874969	-	12113	protein_coding	oncostatin M receptor [Source:MGI Symbol;Acc:MGI:1330819]	-
ENSMUSG0000002701	64.13454583	86.36789368	127.3560844	45.65054754	31.60474828	43.78614452	92.61950798	40.34714678	1.195344526	0.0033919	0.0223548	Cybrd1	2	71117923	71142926	+	5339	protein_coding	cytochrome b reductase 1 [Source:MGI Symbol;Acc:MGI:2654575]	-
ENSMUSG0000002984	2560.371322	2409.874887	2385.133686	2969.008252	3426.152243	3428.941629	2451.793298	3274.700708	-0.417360417	0.0034101	0.0224667	Mtpn	6	35508906	35539888	-	3960	protein_coding	myotrophin [Source:MGI Symbol;Acc:MGI:99445]	-
ENSMUSG0000004270	794.6671069	710.9552224	557.462159	907.8429642	1163.449796	1060.597723	687.6948294	1043.963494	-0.60152698	0.0034276	0.0225747	Atpaf2	11	60400626	60418457	-	2137	protein_coding	ATP synthase mitochondrial F1 complex assembly factor 2 [Source:MGI Symbol;Acc:MGI:2180561]	-
ENSMUSG0000000140	14.0294319	16.85227194	29.04612452	74.93580445	33.58004505	69.0848058	19.97594278	59.20021843	-1.573579283	0.0034314	0.0225917	Ube2c	2	164769898	164778822	+	2600	protein_coding	ubiquitin-conjugating enzyme E2C [Source:MGI Symbol;Acc:MGI:1915862]	-
ENSMUSG0000001947	1063.230518	881.5844757	1073.589448	771.7526527	674.5638461	683.0638545	1006.134814	709.7934511	0.502989923	0.0034433	0.0226621	Xab2	8	3608421	3621316	-	4685	protein_coding	XPA binding protein 2 [Source:MGI Symbol;Acc:MGI:1914689]	-
ENSMUSG0000002922	789.6565956	708.8486884	989.8025508	527.9959555	531.3548305	606.1948452	829.4359449	555.1818771	0.579012105	0.0034509	0.0227036	Chic2	5	74997988	75044774	-	9750	protein_coding	cysteine-rich hydrophobic domain 2 [Source:MGI Symbol;Acc:MGI:1921527]	-
ENSMUSG0000007050	1496.138702	1613.605038	1450.071909	898.3683223	1157.523906	1153.035139	1519.93855	1069.642456	0.507310012	0.0034578	0.0227412	Rgma	7	73375509	73419899	+	5544	protein_coding	repulsive guidance molecule family member A [Source:MGI Symbol;Acc:MGI:2679262]	-
ENSMUSG0000003281	637.3370492	715.1682904	563.0479522	1163.658297	825.6740488	916.5899586	638.5177639	968.640768	-0.601459509	0.0034689	0.0228064	Arap1	7	101348067	101412586	+	8433	protein_coding	ArfGAP with RhoGAP domain, ankyrin repeat and PH domain 1 [Source:MGI Symbol;Acc:MGI:1916960]	-
ENSMUSG0000008475	4.008409114	5.26633498	24.57748997	0	0.987648384	0	11.28407802	0.329216128	5.064191483	0.0034728	0.0228238	Gm12798	4	101403081	101407856	+	666	lincRNA	predicted gene 12798 [Source:MGI Symbol;Acc:MGI:3651483]	-
ENSMUSG0000003022	226.475115	261.210215	249.1263757	405.6869413	356.5410665	339.5858764	245.6039019	367.2712947	-0.581415949	0.003484	0.0228891	Dera	6	137754546	137857340	+	8127	protein_coding	deoxyribose-phosphate aldolase (putative) [Source:MGI Symbol;Acc:MGI:1913762]	-
ENSMUSG0000003305	5334.190429	2311.921056	1825.43721	5125.78129	6969.834644	7499.107018	3157.182898	6531.574318	-1.048671735	0.0034863	0.0228964	Pygb	2	150786735	150831758	+	4809	protein_coding	brain glycogen phosphorylase [Source:MGI Symbol;Acc:MGI:97828]	-
ENSMUSG0000011675	191.4015352	134.8181755	272.586707	109.3890479	70.12303525	112.8709503	199.6021392	97.46101114	1.032582032	0.0034987	0.0229701	AC121151.1	17	16557246	16558043	+	798	processed_pseudog	ribosomal protein L7A (Rpl7a) pseudogene	-
ENSMUSG0000004421	13.02732962	3.159800988	27.92896588	78.38112879	49.38241919	46.70522082	14.7053655	58.15625627	-1.990381191	0.0035195	0.023098	Aqp5	15	99590782	99594829	+	2599	protein_coding	aquaporin 5 [Source:MGI Symbol;Acc:MGI:106215]	-
ENSMUSG0000008582	168.3531828	148.5106465	154.1678917	79.24245987	81.97481585	108.9788486	157.0105736	90.06537477	0.803258788	0.0035319	0.0231716	Gm4285	14	75843379	75844964	-	902	antisense	predicted gene 4285 [Source:MGI Symbol;Acc:MGI:3782463]	-
ENSMUSG0000002228	13247.79212	15645.22796	12719.96822	8855.344891	10444.38166	10704.2528	13870.7996	10001.32645	0.471919631	0.0035357	0.0231884	Pabpc1	15	36595661	36609668	-	6540	protein_coding	poly(A) binding protein, cytoplasmic 1 [Source:MGI Symbol;Acc:MGI:1349722]	-
ENSMUSG0000004184	99.20812558	58.98295178	80.43542174	175.7115415	139.2584221	136.2235607	79.54216637	150.3978414	-0.918958801	0.0035389	0.0232013	Rhod	19	4425459	4439432	-	1714	protein_coding	ras homolog family member D [Source:MGI Symbol;Acc:MGI:108446]	-
ENSMUSG0000003077	69.14505722	81.1015587	35.74907633	291.1299069	94.81424484	119.6821284	61.99856408	168.5420934	-1.442870461	0.0035517	0.0232773	Pak1	7	97788541	97912381	+	3943	protein_coding	p21 (RAC1) activated kinase 1 [Source:MGI Symbol;Acc:MGI:1339975]	-
ENSMUSG0000010796	169.3552851	225.3991372	384.3025705	97.33041267	156.0484446	122.6012047	259.6856643	125.3266873	1.051247847	0.0035624	0.0233393	Gm43980	6	49254851	49256462	+	1612	TEC	predicted gene, 43980 [Source:MGI Symbol;Acc:MGI:5690372]	-
ENSMUSG0000009408	0	0	0	3.445324342	19.75296767	3.892101735	0	9.030131251	-5.535372432	0.0035638	0.0233402	Ighv1-64	12	115507545	115508031	-	405	IG_V_gene	immunoglobulin heavy variable 1-64 [Source:MGI Symbol;Acc:MGI:4439789]	-
ENSMUSG0000006210	104.218637	111.6463016	138.5276708	69.76781793	59.25890302	63.2466532	118.1308698	64.09112472	0.879875357	0.0035767	0.0234165	Zfp119b	17	55938381	55949500	-	2166	protein_coding	zinc finger protein 119b [Source:MGI Symbol;Acc:MGI:2385323]	zf-C2
ENSMUSG0000001979	102.2144324	56.87641779	69.26383538	124.8930074	150.1225543	174.1715526	76.11822853	149.7290381	-0.97350834	0.0035836	0.023453	Lrp11	10	7589800	7625483	+	3418	protein_coding	low density lipoprotein receptor-related protein 11 [Source:MGI Symbol;Acc:MGI:2442989]	-
ENSMUSG0000001376	1.002102279	0	0	11.19730411	7.90118707	10.70327977	0.334034093	9.933923651	-4.714892903	0.0035854	0.0234547	Ly6g6e	17	35076902	35078804	+	1273	protein_coding	lymphocyte antigen 6 complex, locus G6E [Source:MGI Symbol;Acc:MGI:1917524]	-
ENSMUSG0000000128	2365.96348	2024.379167	2390.719479	1819.131253	1115.055025	1452.726973	2260.354042	1462.304417	0.627995688	0.0035863	0.0234547	Pfdn5	15	102326116	102331873	+	1394	protein_coding	prefoldin 5 [Source:MGI Symbol;Acc:MGI:1928753]	-
ENSMUSG0000006436	61766.57815	44100.28913	44485.25685	52813.37684	115089.6785	100943.6046	50117.37471	89615.5533	-0.838431317	0.0035876	0.023455	mt-Nd5	MT	11742	13565	+	1824	protein_coding	mitochondrially encoded NADH dehydrogenase 5 [Source:MGI Symbol;Acc:MGI:102496]	-
ENSMUSG0000001524	388.8156841	327.5660358	382.0682532	500.4333607	508.6389176	612.0329978	366.149991	540.3684254	-0.561235959	0.0035893	0.0234574	Nipsnap3b	4	53011880	53022060	+	1687	protein_coding	nipsnap homolog 3B [Source:MGI Symbol;Acc:MGI:1913786]	-
ENSMUSG0000002649	5.010511393	6.319601977	0	77.5197977	10.86413222	13.62235607	3.776704457	34.00209533	-3.163079822	0.0035957	0.023482	Kif26b	1	178529125	178939200	+	14696	protein_coding	kinesin family member 26B [Source:MGI Symbol;Acc:MGI:2447076]	-
ENSMUSG0000002280	964.022392	969.0056364	804.3542174	1211.892837	1261.226986	1305.800132	912.4607486	1259.639985	-0.464791255	0.0035976	0.023482	Snx4	16	33251442	33300269	+	3988	protein_coding	sorting nexin 4 [Source:MGI Symbol;Acc:MGI:1916400]	-
ENSMUSG0000003752	152.3195463	166.4161854	229.0175202	124.0316763	86.91305777	79.78808557	182.5844173	96.91093989	0.910629988	0.003598	0.023482	Bcdin3d	15	99470079	99474730	-	4652	protein_coding	BCDIN3 domain containing [Source:MGI Symbol;Acc:MGI:1922534]	-
ENSMUSG0000003774	102473.9769	125259.7775	77974.32126	72768.69544	66489.47684	64076.64389	101902.6919	67778.27206	0.588297439	0.003598	0.023482	Eef1a1	9	78478449	78489151	-	2493	protein_coding	eukaryotic translation elongation factor 1 alpha 1 [Source:MGI Symbol;Acc:MGI:1096881]	-
ENSMUSG0000002976	13.02732962	8.426135969	7.820110447	102.4983992	16.79002252	24.32563584	9.757858679	47.87135252	-2.29494412	0.0036045	0.0235163	Ccdc136	6	29396309	29426994	+	4334	protein_coding	coiled-coil domain containing 136 [Source:MGI Symbol;Acc:MGI:1918128]	-
ENSMUSG0000006876	49.10301165	14.74573795	44.68634541	347.1164275	48.3947708	65.19270406	36.178365	153.5679675	-2.086874911	0.0036106	0.0235408	Gstm6	3	107938847	107943749	-	1989	protein_coding	glutathione S-transferase, mu 6 [Source:MGI Symbol;Acc:MGI:1309467]	-
ENSMUSG0000002790	2464.169503	3590.58719	2807.41965	1496.993427	1958.506745	2217.524964	2954.058781	1891.008378	0.643689291	0.0036116	0.0235408	Dram2	3	106547798	106575890	+	3758	protein_coding	DNA-damage regulated autophagy modulator 2 [Source:MGI Symbol;Acc:MGI:1914421]	-
ENSMUSG0000001881	715.5010269	346.5248417	741.7933338	1039.62662	1195.054544	1003.189222	601.2730675	1079.290129	-0.84401656	0.0036121	0.0235408	Lsp1	7	142460809	142494867	+	5174	protein_coding	lymphocyte specific 1 [Source:MGI Symbol;Acc:MGI:96832]	-
ENSMUSG0000002595	5.010511393	4.213067984	1.117158635	47.37320971	10.86413222	14.59538151	3.446912671	24.27757448	-2.809125707	0.0036187	0.0235756	Akr1cl	1	65012689	65032759	-	4209	protein_coding	aldo-keto reductase family 1, member C-like [Source:MGI Symbol;Acc:MGI:1918111]	-
ENSMUSG0000002846	590.2382421	698.3160184	689.2868779	418.6069076	441.4788275	507.9192764	659.2803795	456.0016705	0.531978749	0.0036207	0.0235809	Gba2	4	43566928	43578873	-	4481	protein_coding	glucosidase beta 2 [Source:MGI Symbol;Acc:MGI:2654325]	-
ENSMUSG0000003825	408.8577297	418.1469975	357.4907633	556.4198813	541.2313143	582.8422348	394.8318301	560.1644768	-0.504171116	0.003624	0.0235941	Bcl9	3	97203662	97297917	-	8446	protein_coding	B cell CLL/lymphoma 9 [Source:MGI Symbol;Acc:MGI:1924828]	-
ENSMUSG0000006512	40.08409114	45.29048083	55.85793176	24.1172704	13.82707737	10.70327977	47.07750125	16.21587585	1.528757755	0.0036276	0.0236092	Snord104	11	106500993	106501062	+	70	snoRNA	small nucleolar RNA, C/D box 104 [Source:MGI Symbol;Acc:MGI:3819515]	-
ENSMUSG0000004525	14.0294319	4.213067984	6.702951811	43.92788537	23.70356121	32.10983931	8.315150565	33.2470953	-1.994318368	0.0036341	0.0236411	Klhdc9	1	171358446	171360798	-	1558	protein_coding	kelch domain containing 9 [Source:MGI Symbol;Acc:MGI:1916124]	-
ENSMUSG0000003051	360.7568203	463.4374783	130.7075603	136.9516426	154.0731479	122.6012047	318.3006196	137.8753317	1.207640427	0.0036351	0.0236411	Tarsl2	7	65644898	65692091	+	6741	protein_coding	threonyl-tRNA synthetase-like 2 [Source:MGI Symbol;Acc:MGI:2444486]	-
ENSMUSG0000002739	181.3805124	157.9900494	65.91235948	15.50395954	79.0118707	22.37958498	135.0943071	38.96513841	1.797977907	0.0036388	0.023657	Il1b	2	129364570	129371139	-	1907	protein_coding	interleukin 1 beta [Source:MGI Symbol;Acc:MGI:96543]	-
ENSMUSG0000009716	287.603354	290.7016909	185.4483334	149.0102778	141.2337189	156.6570948	254.5844594	148.9670305	0.774006286	0.0036422	0.0236709	Cep83os	10	94671025	94688576	-	6566	bidirectional_prom	centrosomal protein 83, opposite strand [Source:MGI Symbol;Acc:MGI:1914973]	-
ENSMUSG0000003185	1563.279555	1715.771937	1457.892019	1115.423756	1167.40039	1217.254818	1578.98117	1166.692988	0.436775625	0.003644	0.0236743	Mau2	8	70016123	70042734	-	9131	protein_coding	MAU2 sister chromatid cohesion factor [Source:MGI Symbol;Acc:MGI:1921799]	-
ENSMUSG0000002747	1384.905349	969.0056364	1552.850503	854.4404369	897.7723808	759.9328638	1302.253829	837.3818938	0.636935663	0.003646	0.0236797	Pdrg1	2	153008890	153015427	-	2443	protein_coding	p53 and DNA damage regulated 1 [Source:MGI Symbol;Acc:MGI:1915809]	-
ENSMUSG0000003310	256.5381833	358.1107787	280.4068174	212.7487781	171.8508188	152.7649931	298.3519265	179.12153	0.734716466	0.0036523	0.0237121	Cdc14b	13	64189268	64275290	-	9179	protein_coding	CDC14 cell division cycle 14B [Source:MGI Symbol;Acc:MGI:2441808]	-
ENSMUSG0000002204	3.006306836	1.053266996	2.23431727	14.64262846	22.71591283	10.70327977	2.097963701	16.02060702	-2.92744342	0.0036539	0.023714	Stmn4	14	66344296	66361680	+	1916	protein_coding	stathmin-like 4 [Source:MGI Symbol;Acc:MGI:1931224]	-
ENSMUSG0000004263	541.1352305	470.8103473	590.976918	673.5609089	1047.894935	798.8538811	534.3074986	840.1032417	-0.652634774	0.0036578	0.0237311	Pla2g6	15	79286228	79328390	-	5434	protein_coding	phospholipase A2, group VI [Source:MGI Symbol;Acc:MGI:1859152]	-
ENSMUSG0000002160	322.6769337	440.2656044	249.1263757	613.2677329	491.8488951	585.7613111	337.3563046	563.6259797	-0.740249827	0.0036677	0.0237871	Lpcat1	13	73467197	73516422	+	4632	protein_coding	lysophosphatidylcholine acyltransferase 1 [Source:MGI Symbol;Acc:MGI:2384812]	-
ENSMUSG0000001983	1079.264154	557.1782409	542.9390967	1155.044986	1321.473537	1400.183599	726.4604972	1292.234041	-0.830375888	0.0036692	0.0237887	Slc16a10	10	40033532	40142258	-	7356	protein_coding	solute carrier family 16 (monocarboxylic acid transporters), member 10 [Source:MGI Symbol;Acc:MGI:1919722]	-
ENSMUSG0000011317	3.006306836	5.26633498	11.17158635	22.39460823	23.70356121	39.89404278	6.8198419	28.66407074	-2.155655328	0.0036723	0.0238006	Mylf-ps	12	101221997	101222417	-	421	processed_pseudog	myosin light chain, alkali, fast skeletal muscle, pseudogene [Source:MGI Symbol;Acc:MGI:97270]	-
ENSMUSG0000003237	387.8135818	329.6725698	194.3856025	512.4919959	555.0583917	516.6765053	303.9572514	528.075631	-0.795739185	0.0036755	0.0238134	Aph1b	9	66775202	66795490	-	5163	protein_coding	aph1 homolog B, gamma secretase subunit [Source:MGI Symbol;Acc:MGI:3522097]	-
ENSMUSG0000004073	5226.965485	4608.043108	5257.348537	4012.941528	3669.113746	3722.79531	5030.78571	3801.616861	0.404103567	0.0036787	0.0238161	Eif4h	5	134619721	134639490	-	5551	protein_coding	eukaryotic translation initiation factor 4H [Source:MGI Symbol;Acc:MGI:1341822]	-
ENSMUSG0000002524	2538.325072	2654.23283	1847.780383	2807.078008	4307.134602	4063.354211	2346.779428	3725.855607	-0.666692534	0.003679	0.0238161	Tbl1x	X	77511013	77662983	+	7052	protein_coding	transducin (beta)-like 1 X-linked [Source:MGI Symbol;Acc:MGI:1336172]	-
ENSMUSG0000003870	481.0090937	476.0766822	508.307179	291.1299069	365.429902	348.3431053	488.4643183	334.9676381	0.54499442	0.0036798	0.0238161	Gmeb2	2	181251449	181288035	-	4988	protein_coding	glucocorticoid modulatory element binding protein 2 [Source:MGI Symbol;Acc:MGI:2652836]	SAN
ENSMUSG0000006387	4299.018775	4583.817967	3584.96206	5610.710692	5792.557771	5596.842295	4155.932934	5666.703586	-0.44725926	0.0036812	0.0238168	Chd4	6	125095981	125130591	+	8019	protein_coding	chromodomain helicase DNA binding protein 4 [Source:MGI Symbol;Acc:MGI:1344380]	-
ENSMUSG0000009774	122.256478	191.6945933	196.6199198	86.99443964	102.7154319	88.54531447	170.1903304	92.75172868	0.875274206	0.0036834	0.0238228	Gm16973	14	56696280	56701268	-	2421	lincRNA	predicted gene, 16973 [Source:MGI Symbol;Acc:MGI:4439897]	-
ENSMUSG0000002557	3125.557007	3186.132663	2722.515594	2155.911707	2356.529044	2236.985472	3011.401755	2249.808741	0.420793501	0.0036883	0.0238463	Gaa	11	119267887	119285454	+	4783	protein_coding	glucosidase, alpha, acid [Source:MGI Symbol;Acc:MGI:95609]	-
ENSMUSG0000003088	617.2950036	358.1107787	941.7647295	437.5561915	193.5790832	229.6340024	639.0568373	286.9230924	1.154140407	0.0037017	0.0239251	Vwa3a	7	120739318	120805742	+	7205	protein_coding	von Willebrand factor A domain containing 3A [Source:MGI Symbol;Acc:MGI:3041229]	-
ENSMUSG0000002750	2407.049673	2099.161123	2589.573716	1750.224766	1534.805588	1823.449663	2365.261504	1702.826672	0.473946657	0.0037088	0.0239623	Rtf2	2	172440556	172469908	+	2644	protein_coding	replication termination factor 2 [Source:MGI Symbol;Acc:MGI:1913654]	-
ENSMUSG0000007142	88.18500052	58.98295178	49.15497995	23.25593931	32.59239666	25.29866128	65.44097742	27.04899908	1.278750965	0.0037172	0.0240082	Grid2	6	63255876	64704323	+	19172	protein_coding	glutamate receptor, ionotropic, delta 2 [Source:MGI Symbol;Acc:MGI:95813]	-
ENSMUSG0000003592	1626.411998	2170.783279	2311.401216	1528.001346	1340.238857	1264.933064	2036.198831	1377.724422	0.563264507	0.0037223	0.024033	H2-Q4	17	35379617	35385290	+	2596	protein_coding	histocompatibility 2, Q region locus 4 [Source:MGI Symbol;Acc:MGI:95933]	-
ENSMUSG0000002735	194.4078421	108.4865006	178.7453816	91.30109507	82.96246423	77.8420347	160.5465748	84.035198	0.933339616	0.003724	0.0240355	Mcm8	2	132816141	132844197	+	3718	protein_coding	minichromosome maintenance 8 homologous recombination repair factor [Source:MGI Symbol;Acc:MGI:1913884]	-
ENSMUSG0000001530	2136.482058	2839.607821	2496.84955	2926.803029	4952.068996	3967.997719	2490.97981	3948.956581	-0.664691021	0.0037285	0.0240568	Sash1	10	8722219	8886070	-	8568	protein_coding	SAM and SH3 domain containing 1 [Source:MGI Symbol;Acc:MGI:1917347]	-
ENSMUSG0000002679	148.3111372	178.0021223	153.050733	260.1219878	233.0850186	252.0135873	159.7879975	248.4068646	-0.636992053	0.0037378	0.0241084	Med27	2	29346819	29524793	+	3310	protein_coding	mediator complex subunit 27 [Source:MGI Symbol;Acc:MGI:1916225]	-
ENSMUSG0000002938	822.7259707	854.1995338	1198.711216	648.5823075	639.0085043	614.9520741	958.5455734	634.180962	0.595506054	0.003747	0.0241577	Eif2b1	5	124570213	124579131	-	2581	protein_coding	eukaryotic translation initiation factor 2B, subunit 1 (alpha) [Source:MGI Symbol;Acc:MGI:2384802]	-
ENSMUSG0000011067	46.09670482	63.19601977	61.44372494	27.56259474	22.71591283	22.37958498	56.91214984	24.21936418	1.229181449	0.0037481	0.0241577	Rpl10-ps5	8	94222993	94223634	+	642	processed_pseudog	ribosomal protein L10, pseudogene 5 [Source:MGI Symbol;Acc:MGI:3781583]	-
ENSMUSG0000006968	848.78063	1019.562452	1014.380041	787.2566122	518.5154015	470.9443099	960.9077077	592.2387745	0.697355949	0.0037495	0.0241589	Gm10275	10	29698746	29699380	-	635	transcribed_process	predicted pseudogene 10275 [Source:MGI Symbol;Acc:MGI:3704354]	-
ENSMUSG0000007881	51.10721621	70.56888874	89.37269082	128.3383318	143.2090156	128.4393573	70.34959859	133.3289016	-0.924868054	0.0037628	0.0242359	Prkcg	7	3289179	3331099	+	3612	protein_coding	protein kinase C, gamma [Source:MGI Symbol;Acc:MGI:97597]	-
ENSMUSG0000006937	1.002102279	0	0	62.01583816	2.962945151	0	0.334034093	21.65959444	-5.841066127	0.0037727	0.024282	Ctxn3	18	57468516	57478133	+	1605	protein_coding	cortexin 3 [Source:MGI Symbol;Acc:MGI:3642816]	-
ENSMUSG0000001447	925.9425054	961.6327674	674.7638157	625.3263681	503.7006757	551.705421	854.1130295	560.2441549	0.608298538	0.0037727	0.024282	Rnf166	8	122466147	122476064	-	1985	protein_coding	ring finger protein 166 [Source:MGI Symbol;Acc:MGI:1915968]	-
ENSMUSG0000005254	187.3931261	318.0866328	164.2223194	116.2796966	123.456048	113.8439758	223.2340261	117.8599068	0.92186242	0.0037741	0.024282	St6galnac3	3	153198266	153725174	-	8994	protein_coding	ST6 (alpha-N-acetyl-neuraminyl-2,3-beta-galactosyl-1,3)-N-acetylgalactosaminide alpha-2,6-sialyltransferase 3 [Source:MGI Sym	-
ENSMUSG0000001251	240.5045469	266.47655	249.1263757	336.7804545	368.3928471	548.7863447	252.0358242	417.9865488	-0.729378709	0.0037751	0.024282	Mlkl	8	111311797	111338177	-	2303	protein_coding	mixed lineage kinase domain-like [Source:MGI Symbol;Acc:MGI:1921818]	-
ENSMUSG0000005932	182.3826147	139.0312435	129.5904017	246.3406905	221.233238	281.2043504	150.3347533	249.5927596	-0.730077111	0.0037928	0.0243871	Eda	X	99975606	100400762	+	6825	protein_coding	ectodysplasin-A [Source:MGI Symbol;Acc:MGI:1195272]	-
ENSMUSG0000003274	602.2634694	549.805372	569.750904	460.8121308	341.7263408	343.4779781	573.9399151	382.0054832	0.586354118	0.0037997	0.0244233	D430042O09R	7	125707888	125874793	+	17086	protein_coding	RIKEN cDNA D430042O09 gene [Source:MGI Symbol;Acc:MGI:2442760]	-
ENSMUSG0000003839	79.16608001	84.26135969	58.09224903	40.48256102	25.67885798	33.08286475	73.83989624	33.08142792	1.156035919	0.0038031	0.024437	Gpr162	6	124858444	124863983	-	3838	protein_coding	G protein-coupled receptor 162 [Source:MGI Symbol;Acc:MGI:1315214]	-
ENSMUSG0000002246	697.4631859	662.5049405	678.1152916	520.2439757	498.7624338	439.8074961	679.3611393	486.2713019	0.482104724	0.0038159	0.0245104	Rpap3	15	97675097	97705825	-	3224	protein_coding	RNA polymerase II associated protein 3 [Source:MGI Symbol;Acc:MGI:1277218]	-
ENSMUSG0000002125	580.2172193	545.592304	656.8892775	432.388205	429.6270469	381.42597	594.2329336	414.4804073	0.519231852	0.0038186	0.0245195	Ccnk	12	108179579	108203359	+	3042	protein_coding	cyclin K [Source:MGI Symbol;Acc:MGI:1276106]	-
ENSMUSG0000002222	141.2964213	159.0433164	186.5654921	105.9437235	93.82659646	68.11178036	162.3017433	89.29403345	0.859607759	0.003835	0.0246159	Ripk3	14	55784995	55788865	-	2292	protein_coding	receptor-interacting serine-threonine kinase 3 [Source:MGI Symbol;Acc:MGI:2154952]	-
ENSMUSG0000004749	1276.678303	1134.368555	1146.20476	1372.96175	1886.408413	2056.975767	1185.750539	1772.11531	-0.579341165	0.0038362	0.0246159	Adamts12	15	11064790	11349231	+	10921	protein_coding	a disintegrin-like and metallopeptidase (reprolysin type) with thrombospondin type 1 motif, 12 [Source:MGI Symbol;Acc:MGI:214	-
ENSMUSG0000005653	1559.271146	1732.624209	2156.116166	1209.308844	1294.807031	1298.015929	1816.00384	1267.377268	0.518789882	0.0038406	0.0246356	Rlim	X	103957163	103981284	-	8917	protein_coding	ring finger protein, LIM domain interacting [Source:MGI Symbol;Acc:MGI:1342291]	-
ENSMUSG0000003380	137.2880122	142.1910445	174.2767471	222.2234201	242.9615024	254.9326637	151.2519346	240.0391954	-0.66700254	0.0038449	0.0246547	Alg3	16	20605374	20611735	-	1690	protein_coding	asparagine-linked glycosylation 3 (alpha-1,3-mannosyltransferase) [Source:MGI Symbol;Acc:MGI:1098592]	-
ENSMUSG0000000354	504.0574461	328.6193028	861.3293078	306.6338665	289.3809764	271.474096	564.6686856	289.1629796	0.964965401	0.0038582	0.0247311	Ercc1	7	19344778	19356524	+	3111	protein_coding	excision repair cross-complementing rodent repair deficiency, complementation group 1 [Source:MGI Symbol;Acc:MGI:95412]	-
ENSMUSG0000003102	4267.953605	4655.440123	3497.823687	3354.023247	2529.367511	2508.459568	4140.405805	2797.283442	0.565646932	0.0038595	0.0247311	Eif3f	7	108934417	108942951	+	2891	protein_coding	eukaryotic translation initiation factor 3, subunit F [Source:MGI Symbol;Acc:MGI:1913335]	-
ENSMUSG0000010921	8.016818229	13.69247095	34.63191769	1.722662171	3.950593535	0	18.78040229	1.891085235	3.314576173	0.0038618	0.0247311	Gm44907	7	90457252	90458884	+	1633	TEC	predicted gene 44907 [Source:MGI Symbol;Acc:MGI:5753483]	-
ENSMUSG0000001014	47.0988071	37.91761186	42.45202814	121.4476831	78.02422232	72.97690753	42.48948236	90.81627097	-1.097448597	0.0038623	0.024732	Tnfrsf13b	11	61126755	61149372	+	2836	protein_coding	tumor necrosis factor receptor superfamily, member 13b [Source:MGI Symbol;Acc:MGI:1889411]	-
ENSMUSG0000003002	1242.606825	1202.83091	873.6180527	1492.686771	1837.025994	1620.087347	1106.351929	1649.933371	-0.576174793	0.003879	0.0248305	Lrig1	6	94604529	94700158	-	5585	protein_coding	leucine-rich repeats and immunoglobulin-like domains 1 [Source:MGI Symbol;Acc:MGI:107935]	-
ENSMUSG0000005377	1093.293586	1482.999931	1514.867109	949.1868563	927.4018323	941.8886199	1363.720209	939.4924362	0.53732195	0.0038835	0.0248507	Ubxn7	16	32332257	32393747	+	12655	protein_coding	UBX domain protein 7 [Source:MGI Symbol;Acc:MGI:2146388]	-
ENSMUSG0000003582	909.908869	813.122121	782.0110447	666.6702602	499.7500822	550.7323955	835.0140115	572.384246	0.544352435	0.0039227	0.0250921	Tk2	8	104226685	104248558	-	3571	protein_coding	thymidine kinase 2, mitochondrial [Source:MGI Symbol;Acc:MGI:1913266]	-
ENSMUSG0000003084	223.4688081	150.6171804	121.7702912	297.1592245	259.7515249	322.0714186	165.2854266	292.994056	-0.824331069	0.0039239	0.0250921	Rgs10	7	128373621	128418758	-	1711	protein_coding	regulator of G-protein signalling 10 [Source:MGI Symbol;Acc:MGI:1915115]	-
ENSMUSG0000002251	56.1177276	52.6633498	21.22601407	98.19174376	110.616619	95.35649251	43.33569716	101.3882851	-1.222238466	0.0039267	0.0251016	Srl	16	4480216	4541816	-	5325	protein_coding	sarcalumenin [Source:MGI Symbol;Acc:MGI:2146620]	-
ENSMUSG0000003189	2.004204557	0	0	56.84785165	2.962945151	2.919076301	0.668068186	20.9099577	-4.930984599	0.0039345	0.0251378	Tsnaxip1	8	105827731	105844680	+	2227	protein_coding	translin-associated factor X (Tsnax) interacting protein 1 [Source:MGI Symbol;Acc:MGI:1919486]	-
ENSMUSG0000003446	433.9102866	202.2272633	199.9713957	490.9587188	568.885469	535.1639886	278.7029819	531.6693921	-0.930629286	0.0039362	0.0251378	Scara3	14	65919394	65953935	-	3597	protein_coding	scavenger receptor class A, member 3 [Source:MGI Symbol;Acc:MGI:2444418]	-
ENSMUSG0000001520	489.025912	562.4445759	487.081165	335.9191234	357.5287149	380.4529446	512.8505509	357.9669276	0.519136912	0.0039364	0.0251378	Cnksr3	10	7119063	7212237	-	3837	protein_coding	Cnksr family member 3 [Source:MGI Symbol;Acc:MGI:2674130]	-
ENSMUSG0000007343	1.002102279	0	0	65.4611625	0	1.946050868	0.334034093	22.46907112	-5.893890942	0.0039498	0.0252147	Arhgdig	17	26196226	26207786	-	3252	protein_coding	Rho GDP dissociation inhibitor (GDI) gamma [Source:MGI Symbol;Acc:MGI:108430]	-
ENSMUSG0000006960	45.09460254	38.97087886	14.52306226	381.5696709	39.50593535	47.67824626	32.86284788	156.2512842	-2.248814966	0.0039585	0.0252621	Ank3	10	69398773	70027438	+	34838	protein_coding	ankyrin 3, epithelial [Source:MGI Symbol;Acc:MGI:88026]	-
ENSMUSG0000003517	243.5108537	321.2464338	139.6448294	503.8786851	457.2812017	366.8305885	234.8007056	442.6634918	-0.914232953	0.0039606	0.0252663	Plekhh3	11	101162679	101171351	-	3582	protein_coding	pleckstrin homology domain containing, family H (with MyTH4 domain) member 3 [Source:MGI Symbol;Acc:MGI:2384950]	-
ENSMUSG0000000290	141.2964213	179.0553893	100.5442772	66.32249359	81.97481585	70.05783123	140.2986959	72.78504689	0.948588342	0.0039643	0.0252748	Kcnn1	8	70842049	70863258	-	4587	protein_coding	potassium intermediate/small conductance calcium-activated channel, subfamily N, member 1 [Source:MGI Symbol;Acc:MGI:193	-
ENSMUSG0000005094	386.8114795	495.0354882	353.0221287	218.7780957	308.1462957	231.5800532	411.6230321	252.8348149	0.704090449	0.0039646	0.0252748	Amigo1	3	108186335	108192286	+	5540	protein_coding	adhesion molecule with Ig like domain 1 [Source:MGI Symbol;Acc:MGI:2653612]	-
ENSMUSG0000010434	63.13244355	92.68749566	184.3311748	36.17590559	54.32066111	43.78614452	113.3837074	44.76090374	1.340768975	0.0039696	0.0252948	Gm37691	1	193281271	193283843	+	2573	TEC	predicted gene, 37691 [Source:MGI Symbol;Acc:MGI:5610919]	-
ENSMUSG0000003000	2678.619391	2672.138369	2913.549721	2217.927545	2066.160419	1989.837012	2754.76916	2091.308325	0.397344311	0.0039712	0.0252948	Cct7	6	85451514	85468475	+	6778	protein_coding	chaperonin containing Tcp1, subunit 7 (eta) [Source:MGI Symbol;Acc:MGI:107184]	-
ENSMUSG0000001886	1.002102279	1.053266996	0	6.890648685	12.83942899	16.54143237	0.685123092	12.09050335	-4.111855756	0.0039718	0.0252948	Pnpla5	15	84112356	84123175	-	2172	protein_coding	patatin-like phospholipase domain containing 5 [Source:MGI Symbol;Acc:MGI:1923022]	-
ENSMUSG0000002467	166.3489783	350.7379097	353.0221287	134.3676494	151.1102027	164.4412983	290.0363389	149.9730501	0.95123593	0.0039792	0.0253337	Ms4a6d	19	11586604	11604849	-	1735	protein_coding	membrane-spanning 4-domains, subfamily A, member 6D [Source:MGI Symbol;Acc:MGI:1916024]	-
ENSMUSG0000002204	1811.80092	1632.563844	1283.615272	2116.290477	2489.861575	2251.580854	1575.993345	2285.910969	-0.536189854	0.0039834	0.0253518	Trim35	14	66297031	66311424	+	4618	protein_coding	tripartite motif-containing 35 [Source:MGI Symbol;Acc:MGI:1914104]	-
ENSMUSG0000002033	5.010511393	7.372868973	7.820110447	32.73058125	20.74061606	24.32563584	6.734496937	25.93227772	-1.951617278	0.0039907	0.0253891	Hmmr	11	40701395	40733422	-	5431	protein_coding	hyaluronan mediated motility receptor (RHAMM) [Source:MGI Symbol;Acc:MGI:104667]	-
ENSMUSG0000002430	81.17028457	71.62215573	27.92896588	129.1996628	136.295477	145.9538151	60.24046873	137.1496516	-1.183503666	0.0039939	0.0254008	Cdh2	18	16588877	16809246	-	8066	protein_coding	cadherin 2 [Source:MGI Symbol;Acc:MGI:88355]	-
ENSMUSG0000006224	18.03784102	18.95880593	21.22601407	68.90648685	35.55534181	49.62429712	19.40755367	51.36204193	-1.407917996	0.0040154	0.025529	Cks2	13	51645232	51650660	+	692	protein_coding	CDC28 protein kinase regulatory subunit 2 [Source:MGI Symbol;Acc:MGI:1913447]	-
ENSMUSG0000005364	1102.312506	1030.095122	889.2582736	1292.857959	1422.213673	1488.728914	1007.221967	1401.266849	-0.475899233	0.0040171	0.0255311	Aldh7a1	18	56509687	56572951	-	5181	protein_coding	aldehyde dehydrogenase family 7, member A1 [Source:MGI Symbol;Acc:MGI:108186]	-
ENSMUSG0000003070	627.3160264	525.5802311	603.265663	961.2454915	808.8840263	760.9058892	585.3873068	843.678469	-0.52763017	0.0040216	0.0255513	Coro1a	7	126699773	126707787	-	2783	protein_coding	coronin, actin binding protein 1A [Source:MGI Symbol;Acc:MGI:1345961]	-
ENSMUSG0000003713	138.2901144	102.1668986	64.79520084	256.6766635	189.6284897	166.3873492	101.750738	204.2308341	-1.003826171	0.004024	0.0255579	Aff3	1	38177326	38664955	-	6654	protein_coding	AF4/FMR2 family, member 3 [Source:MGI Symbol;Acc:MGI:106927]	AF-4
ENSMUSG0000001052	1.002102279	1.053266996	1.117158635	20.67194605	8.888835454	8.757228904	1.057509303	12.77267014	-3.600259169	0.0040288	0.0255751	Gm266	12	111484609	111485823	-	1215	protein_coding	predicted gene 266 [Source:MGI Symbol;Acc:MGI:2685112]	-
ENSMUSG0000002176	841.765914	634.0667317	1050.129117	1291.996628	1225.671644	1394.345447	841.9872543	1304.004573	-0.631334746	0.0040295	0.0255751	Ndufs4	13	114287795	114388258	-	2708	protein_coding	NADH:ubiquinone oxidoreductase core subunit S4 [Source:MGI Symbol;Acc:MGI:1343135]	-
ENSMUSG0000002173	948.9908579	1014.296117	1065.769338	1378.129737	1305.671163	1350.559302	1009.685438	1344.786734	-0.413758214	0.0040334	0.0255914	Psmd6	14	14112174	14120984	-	2800	protein_coding	proteasome (prosome, macropain) 26S subunit, non-ATPase, 6 [Source:MGI Symbol;Acc:MGI:1913663]	-
ENSMUSG0000002411	239.5024446	132.7116415	186.5654921	93.02375724	90.8636513	115.7900266	186.2598594	99.89247839	0.899842587	0.0040477	0.0256731	Tedc2	17	24215054	24220851	-	2551	protein_coding	tubulin epsilon and delta complex 2 [Source:MGI Symbol;Acc:MGI:1919266]	-
ENSMUSG0000003036	2.004204557	2.106533992	1.117158635	11.19730411	4.938241919	37.94799192	1.742632395	18.02784598	-3.363118508	0.0040499	0.0256785	Klrb1a	6	128609227	128623533	-	1595	protein_coding	killer cell lectin-like receptor subfamily B member 1A [Source:MGI Symbol;Acc:MGI:107540]	-
ENSMUSG0000003235	325.6832406	300.1810939	233.4861548	459.9507997	501.7253789	384.3450463	286.4501631	448.6737417	-0.646569947	0.0040587	0.0257255	Lrrc1	9	77430823	77544870	-	8195	protein_coding	leucine rich repeat containing 1 [Source:MGI Symbol;Acc:MGI:2442313]	-
ENSMUSG0000003200	740.5535839	558.2315079	406.6457432	833.7684909	908.636513	1081.031257	568.476945	941.1454203	-0.726566954	0.0040643	0.0257524	Sesn3	9	14275067	14333101	+	15269	protein_coding	sestrin 3 [Source:MGI Symbol;Acc:MGI:1922997]	-
ENSMUSG0000010660	13.02732962	6.319601977	3.351475906	0	0	0	7.566135835	0	5.455896627	0.004069	0.0257736	Gm43181	5	43784046	43786117	-	2072	TEC	predicted gene 43181 [Source:MGI Symbol;Acc:MGI:5663318]	-
ENSMUSG0000001983	93.19551191	70.56888874	37.9833936	102.4983992	206.4185122	157.6301203	67.24926475	155.5156772	-1.205814291	0.0040856	0.025868	Wasf1	10	40883475	40938570	+	2854	protein_coding	WAS protein family, member 1 [Source:MGI Symbol;Acc:MGI:1890563]	-
ENSMUSG0000007831	118.2480689	120.0724376	88.25553218	211.026116	190.6161381	159.5761711	108.8586795	187.0728084	-0.780550467	0.0040867	0.025868	F8a	X	73228291	73230795	+	2505	protein_coding	factor 8-associated gene A [Source:MGI Symbol;Acc:MGI:95474]	-
ENSMUSG0000002438	1.002102279	1.053266996	0	64.59983142	0.987648384	1.946050868	0.685123092	22.51117689	-5.021164044	0.0040922	0.025894	Proc	18	32123129	32139570	-	1566	protein_coding	protein C [Source:MGI Symbol;Acc:MGI:97771]	-
ENSMUSG0000006600	328.6895474	615.1079257	642.3662153	279.9326028	313.0845376	309.4220879	528.7212295	300.8130761	0.813342859	0.0041025	0.0259505	Zfp979	4	147611937	147642513	-	4111	protein_coding	zinc finger protein 979 [Source:MGI Symbol;Acc:MGI:2148252]	zf-C2
ENSMUSG0000002737	610.2802877	491.8756872	420.0516468	301.46588	373.3310891	295.7997319	507.4025406	323.5322336	0.650167398	0.0041075	0.0259732	Mall	2	127704386	127729932	-	2006	protein_coding	mal, T cell differentiation protein-like [Source:MGI Symbol;Acc:MGI:2385152]	-
ENSMUSG0000000053	1625.409896	1524.077343	1861.186286	1122.314405	1283.942899	1233.79625	1670.224509	1213.351184	0.461106276	0.0041131	0.026	Tom1l2	11	60226714	60352905	-	8764	protein_coding	target of myb1-like 2 (chicken) [Source:MGI Symbol;Acc:MGI:2443306]	-
ENSMUSG0000002195	9684.316421	10595.86598	8785.335507	9182.650703	26296.13822	19754.36236	9688.50597	18411.05043	-0.926200841	0.0041161	0.0260102	Tkt	14	30548359	30574720	+	7355	protein_coding	transketolase [Source:MGI Symbol;Acc:MGI:105992]	-
ENSMUSG0000003143	890.8689257	729.9140283	738.4418579	772.6139838	2061.222177	1658.035339	786.4082706	1497.2905	-0.92866331	0.0041219	0.0260381	Morc4	X	139821632	139871677	-	6051	protein_coding	microrchidia 4 [Source:MGI Symbol;Acc:MGI:1922996]	-
ENSMUSG0000001912	1619.397282	2565.758402	2939.244369	1101.642458	1736.285859	1351.532328	2374.800018	1396.486882	0.766060811	0.0041379	0.0261286	Ccl9	11	83572919	83578636	-	3045	protein_coding	chemokine (C-C motif) ligand 9 [Source:MGI Symbol;Acc:MGI:104533]	-
ENSMUSG0000003447	2271.765866	1801.086563	1520.452903	1387.604379	1239.498722	1048.921418	1864.43511	1225.341506	0.605561102	0.004139	0.0261286	Caskin2	11	115799183	115813639	-	5177	protein_coding	CASK-interacting protein 2 [Source:MGI Symbol;Acc:MGI:2157062]	-
ENSMUSG0000003075	864.8142664	745.7130332	1063.535021	1121.453073	1450.855476	1483.863787	891.3541068	1352.057445	-0.601092149	0.0041411	0.026129	Far1	7	113513834	113571511	+	8055	protein_coding	fatty acyl CoA reductase 1 [Source:MGI Symbol;Acc:MGI:1914670]	-
ENSMUSG0000003557	823.728073	768.8849072	777.5424101	1075.802526	1006.413703	1091.734537	790.0517968	1057.983589	-0.421251803	0.0041419	0.026129	Utp6	11	79932321	79962390	-	4517	protein_coding	UTP6 small subunit processome component [Source:MGI Symbol;Acc:MGI:2445193]	-
ENSMUSG0000011524	4.008409114	5.26633498	4.468634541	18.0879528	15.80237414	31.13681388	4.581126212	21.67571361	-2.242352003	0.004148	0.026159	Gm5144	15	11906519	11907464	+	946	bidirectional_prom	predicted gene 5144 [Source:MGI Symbol;Acc:MGI:3779468]	-
ENSMUSG0000002747	689.4463677	658.2918726	728.3874302	536.6092663	482.9600597	379.4799192	692.0418901	466.3497484	0.568770129	0.0041553	0.0261961	Ccm2l	2	153065955	153081735	+	2407	protein_coding	cerebral cavernous malformation 2-like [Source:MGI Symbol;Acc:MGI:2385159]	-
ENSMUSG0000005949	1039.180063	1060.639865	1103.752732	706.2914902	792.0940038	845.559102	1067.857553	781.3148653	0.45102963	0.0041589	0.0262052	Fcgr3	1	171051174	171064935	-	1736	protein_coding	Fc receptor, IgG, low affinity III [Source:MGI Symbol;Acc:MGI:95500]	-
ENSMUSG0000001897	717.5052315	704.6356204	670.2951811	537.4705974	486.9106532	502.0811238	697.4786777	508.8207915	0.454784729	0.0041596	0.0262052	Sart3	5	113742446	113772510	-	5657	protein_coding	squamous cell carcinoma antigen recognized by T cells 3 [Source:MGI Symbol;Acc:MGI:1858230]	-
ENSMUSG0000002018	119.2501712	310.7137638	100.5442772	85.27177747	61.23419979	67.13875493	176.8360707	71.21491073	1.311304578	0.0041662	0.0262384	Cpm	10	117629500	117687352	+	5964	protein_coding	carboxypeptidase M [Source:MGI Symbol;Acc:MGI:1917824]	-
ENSMUSG0000000277	2356.944559	2158.144075	1930.450122	1572.790562	1548.632666	1653.170212	2148.512919	1591.531147	0.433095704	0.0041861	0.0263546	Kdelr1	7	45872772	45883732	+	1874	protein_coding	KDEL (Lys-Asp-Glu-Leu) endoplasmic reticulum protein retention receptor 1 [Source:MGI Symbol;Acc:MGI:1915387]	-
ENSMUSG0000005554	51.10721621	49.50354882	25.69464861	63.73850033	159.9990382	97.30254338	42.10180455	107.0133606	-1.341816042	0.0041916	0.0263805	Timd4	11	46810800	46844332	+	2173	protein_coding	T cell immunoglobulin and mucin domain containing 4 [Source:MGI Symbol;Acc:MGI:2445125]	-
ENSMUSG0000003319	3152.613769	4950.354882	2820.825554	2542.649365	2134.308157	2107.57309	3641.264735	2261.510204	0.687101455	0.0041952	0.0263944	Tie1	4	118471191	118490061	-	4917	protein_coding	tyrosine kinase with immunoglobulin-like and EGF-like domains 1 [Source:MGI Symbol;Acc:MGI:99906]	-
ENSMUSG0000010230	11.02312506	15.79900494	12.28874499	1.722662171	1.975296767	0.973025434	13.03695833	1.556994791	3.063082992	0.0042135	0.0265004	Gm38192	9	73024293	73026139	+	1847	TEC	predicted gene, 38192 [Source:MGI Symbol;Acc:MGI:5611420]	-
ENSMUSG0000001972	970.0350057	1006.923248	697.1069884	1022.399999	1626.656888	1887.669342	891.3550808	1512.242076	-0.76219069	0.0042248	0.0265624	Lyst	13	13590397	13778803	+	17459	protein_coding	lysosomal trafficking regulator [Source:MGI Symbol;Acc:MGI:107448]	-
ENSMUSG0000009870	23.04835241	45.29048083	50.27213858	12.0586352	12.83942899	13.62235607	39.53699061	12.84014009	1.621288742	0.0042269	0.0265669	Gm27252	7	63896533	63899698	+	717	processed_transcrip	predicted gene 27252 [Source:MGI Symbol;Acc:MGI:5521095]	-
ENSMUSG0000002843	19.03994329	17.90553893	21.22601407	177.4342036	32.59239666	29.19076301	19.39049877	79.7391211	-2.042911994	0.0042283	0.0265669	Epb41l4b	4	56991972	57143437	-	8858	protein_coding	erythrocyte membrane protein band 4.1 like 4b [Source:MGI Symbol;Acc:MGI:1859149]	-
ENSMUSG0000002898	10939.95058	9554.184922	14410.22914	6031.040261	8242.913411	8378.72201	11634.78824	7550.891894	0.623747812	0.0042357	0.0266045	H6pd	4	149979475	150009023	-	5236	protein_coding	hexose-6-phosphate dehydrogenase (glucose 1-dehydrogenase) [Source:MGI Symbol;Acc:MGI:2140356]	-
ENSMUSG0000008941	13.02732962	13.63920395	10.05442772	0.861331086	0	1.946050868	11.9069871	0.935793984	3.668809477	0.0042479	0.0266718	Gm22009	3	69085105	69085447	-	343	scaRNA	predicted gene, 22009 [Source:MGI Symbol;Acc:MGI:5451786]	-
ENSMUSG0000002018	127.2669894	125.3387725	86.02121491	56.84785165	56.29595787	65.19270406	112.8756589	59.44550453	0.926792932	0.004252	0.0266803	E2f7	10	110745439	110787384	+	9009	protein_coding	E2F transcription factor 7 [Source:MGI Symbol;Acc:MGI:1289147]	E2F
ENSMUSG0000003194	118.2480689	132.7116415	182.0968575	80.96512205	75.06127716	82.70716187	144.3521893	79.57785369	0.857522007	0.0042521	0.0266803	Adat1	8	111966908	111992302	-	4399	protein_coding	adenosine deaminase, tRNA-specific 1 [Source:MGI Symbol;Acc:MGI:1353631]	-
ENSMUSG0000004089	54.11352305	51.61008281	79.3182631	244.6180283	89.87600292	111.8979249	61.68062298	148.7973187	-1.273571907	0.0042535	0.0266804	Kcnd3	3	105452330	105674002	+	9587	protein_coding	potassium voltage-gated channel, Shal-related family, member 3 [Source:MGI Symbol;Acc:MGI:1928743]	-
ENSMUSG0000004607	1096.299893	1076.43887	910.4842877	1240.316763	1546.657369	1693.064255	1027.741017	1493.346129	-0.53863153	0.0042807	0.0268364	Lrrc8d	5	105699969	105832436	+	12573	protein_coding	leucine rich repeat containing 8D [Source:MGI Symbol;Acc:MGI:1922368]	-
ENSMUSG0000002883	86.18079596	85.31462668	78.20110447	370.3723668	94.81424484	161.522222	83.2321757	208.9029446	-1.328844789	0.0042813	0.0268364	Stmn1	4	134468320	134473843	+	1922	protein_coding	stathmin 1 [Source:MGI Symbol;Acc:MGI:96739]	-
ENSMUSG0000002622	1.002102279	1.053266996	2.23431727	29.28525691	10.86413222	5.838152603	1.429895515	15.32918058	-3.439469188	0.0042837	0.0268428	Spata3	1	86016816	86029958	+	2666	protein_coding	spermatogenesis associated 3 [Source:MGI Symbol;Acc:MGI:1917310]	-
ENSMUSG0000011543	4.008409114	2.106533992	18.9916968	22.39460823	56.29595787	47.67824626	8.368879968	42.12293745	-2.341581722	0.0042904	0.0268757	Gm36107	14	106265292	106271248	+	426	lincRNA	predicted gene, 36107 [Source:MGI Symbol;Acc:MGI:5595266]	-
ENSMUSG0000006822	9566.068352	2994.43807	9513.722938	16367.87462	11875.48417	17990.26725	7358.076453	15411.20868	-1.066585566	0.004308	0.0269771	Lgals1	15	78926725	78930465	+	903	protein_coding	lectin, galactose binding, soluble 1 [Source:MGI Symbol;Acc:MGI:96777]	-
ENSMUSG0000002242	1364.863303	1313.423944	1007.677089	670.9769157	903.6982711	870.8577632	1228.654779	815.17765	0.592514488	0.0043129	0.0269988	Josd1	15	79674251	79687919	-	3440	protein_coding	Josephin domain containing 1 [Source:MGI Symbol;Acc:MGI:1921408]	-
ENSMUSG0000003916	529.1100031	451.8515413	673.646657	351.4230829	382.2199245	344.4510036	551.5360672	359.3646703	0.617717242	0.0043186	0.0270252	Cmc1	9	118064522	118150177	-	1169	protein_coding	COX assembly mitochondrial protein 1 [Source:MGI Symbol;Acc:MGI:1915149]	-
ENSMUSG0000002270	274.5760243	218.0262682	363.0765564	195.5221564	135.3078286	155.6840694	285.226283	162.1713515	0.81275237	0.0043235	0.0270357	Ccdc191	16	43889800	43964314	+	4230	protein_coding	coiled-coil domain containing 191 [Source:MGI Symbol;Acc:MGI:1922661]	-
ENSMUSG0000002684	378.7946613	379.1761186	289.3440865	762.2780107	425.6764534	579.9231585	349.1049555	589.2925409	-0.75551713	0.0043246	0.0270357	Lamc3	2	31887291	31946539	+	6091	protein_coding	laminin gamma 3 [Source:MGI Symbol;Acc:MGI:1344394]	-
ENSMUSG0000003433	167.3510805	164.3096514	121.7702912	224.8074133	268.6403604	242.283333	151.1436748	245.2437022	-0.69651258	0.0043255	0.0270357	Plcg2	8	117498291	117635142	+	4298	protein_coding	phospholipase C, gamma 2 [Source:MGI Symbol;Acc:MGI:97616]	-
ENSMUSG0000004362	20.04204557	14.74573795	25.69464861	55.12518948	51.35771595	44.75916995	20.16081071	50.41402513	-1.325411227	0.0043261	0.0270357	1700019D03Ri	1	52922324	53020179	-	4330	protein_coding	RIKEN cDNA 1700019D03 gene [Source:MGI Symbol;Acc:MGI:1914330]	-
ENSMUSG0000002682	14.0294319	9.479402965	7.820110447	42.20522319	40.49358373	22.37958498	10.44298177	35.02613063	-1.742082583	0.0043311	0.0270579	Efcc1	6	87730869	87755908	+	4053	protein_coding	EF hand and coiled-coil domain containing 1 [Source:MGI Symbol;Acc:MGI:3611451]	-
ENSMUSG0000004485	997.0917672	1132.262021	863.563625	745.051389	714.0697814	600.3566926	997.6391377	686.492621	0.539192054	0.0043484	0.0271572	Lemd2	17	27189601	27204438	-	2595	protein_coding	LEM domain containing 2 [Source:MGI Symbol;Acc:MGI:2385045]	-
ENSMUSG0000002143	702.4736973	727.8074943	1194.242581	523.6893	581.724898	492.3508695	874.8412576	532.5883559	0.715656629	0.004354	0.0271833	Snrnp48	13	38204938	38227665	+	10129	protein_coding	small nuclear ribonucleoprotein 48 (U11/U12) [Source:MGI Symbol;Acc:MGI:1915047]	-
ENSMUSG0000002068	562.1793783	530.846566	310.5701006	590.8731247	999.5001644	926.320213	467.8653483	838.897834	-0.841556563	0.0043592	0.0272065	Mmp28	11	83440768	83463071	-	3874	protein_coding	matrix metallopeptidase 28 (epilysin) [Source:MGI Symbol;Acc:MGI:2153062]	-
ENSMUSG0000004132	1733.636942	1505.118537	1715.955664	1078.386519	1310.609405	1213.362716	1651.570381	1200.786213	0.460130675	0.0043678	0.0272509	Pcf11	7	92643543	92669934	-	7827	protein_coding	PCF11 cleavage and polyadenylation factor subunit [Source:MGI Symbol;Acc:MGI:1919579]	-
ENSMUSG0000005292	1262.648871	675.1441445	1042.309007	502.1560229	602.4655141	672.3605747	993.3673407	592.3273706	0.746320468	0.0043695	0.0272525	Ctif	18	75431224	75697696	-	6173	protein_coding	CBP80/20-dependent translation initiation factor [Source:MGI Symbol;Acc:MGI:2685518]	-
ENSMUSG0000002189	501.0511393	601.4154548	673.646657	429.8042117	399.009947	377.5338683	592.0377504	402.116009	0.557243978	0.004373	0.0272651	Mettl6	14	31473578	31495040	-	7752	protein_coding	methyltransferase like 6 [Source:MGI Symbol;Acc:MGI:1914261]	-
ENSMUSG0000003066	2623.503765	2836.44802	2467.803425	1872.53378	2101.715761	1992.756088	2642.58507	1989.001876	0.410071436	0.0043785	0.0272903	Ipo5	14	120911224	120947999	+	6001	protein_coding	importin 5 [Source:MGI Symbol;Acc:MGI:1917822]	-
ENSMUSG0000001984	4948.381052	2960.733526	3777.113346	4600.369328	7420.202307	7512.729374	3895.409308	6511.100336	-0.741034127	0.0043942	0.027379	Lama4	10	38965515	39110188	+	12068	protein_coding	laminin, alpha 4 [Source:MGI Symbol;Acc:MGI:109321]	-
ENSMUSG0000003760	1.002102279	0	0	62.87716925	1.975296767	0	0.334034093	21.61748867	-5.838205147	0.0043995	0.027403	Kdf1	4	133518963	133530790	+	2379	protein_coding	keratinocyte differentiation factor 1 [Source:MGI Symbol;Acc:MGI:1916323]	-
ENSMUSG0000009046	0	0	0	4.306655428	0.987648384	25.29866128	0	10.19765503	-5.712337267	0.0044158	0.0274956	Gm17043	12	103786113	103804362	-	1283	unprocessed_pseud	predicted gene 17043 [Source:MGI Symbol;Acc:MGI:4937870]	-
ENSMUSG0000002218	2456.152685	2517.308121	2894.558024	2029.296038	2001.963274	1821.503612	2622.672943	1950.920975	0.426707966	0.0044178	0.027499	Acin1	14	54642161	54686931	-	10527	protein_coding	apoptotic chromatin condensation inducer 1 [Source:MGI Symbol;Acc:MGI:1891824]	-
ENSMUSG0000003510	430.9039798	291.7549579	481.4953718	535.7479352	800.9828392	672.3605747	401.3847698	669.6971164	-0.738362746	0.0044226	0.0275113	Dcbld2	16	58408443	58486927	+	8401	protein_coding	discoidin, CUB and LCCL domain containing 2 [Source:MGI Symbol;Acc:MGI:1920629]	-
ENSMUSG0000002210	25269.01106	22795.8576	18110.25864	33275.80383	28536.12475	31949.29012	22058.37576	31253.73957	-0.50268998	0.0044227	0.0275113	Itm2b	14	73362226	73385289	-	2732	protein_coding	integral membrane protein 2B [Source:MGI Symbol;Acc:MGI:1309517]	-

ENSMUSG0000003796	2247.715411	2327.720061	3548.095825	1561.593258	1986.1609	1639.547856	2707.843766	1729.100671	0.64708295	0.0044333	0.0275676	Zc3h7a	16	11136592	11176393	-	9160	protein_coding	zinc finger CCCH type containing 7 A [Source:MGI Symbol;Acc:MGI:2445044]	-
ENSMUSG0000008977	832.7469935	540.325969	1112.690001	929.3762414	2203.443544	1787.447722	828.5876544	1640.089169	-0.985011098	0.0044352	0.0275703	Slc5a3	16	92058322	92087473	+	10913	protein_coding	solute carrier family 5 (inositol transporters), member 3 [Source:MGI Symbol;Acc:MGI:1858226]	-
ENSMUSG0000010300	28.0588638	12.63920395	32.39760042	57.70918273	67.16009009	61.30060233	24.36522273	62.05662505	-1.349736553	0.0044475	0.0276296	Gm4430	2	129197252	129199194	-	1943	TEC	predicted gene 4430 [Source:MGI Symbol;Acc:MGI:3782614]	-
ENSMUSG0000007249	310.6517064	295.9680259	186.5654921	380.7083398	463.207092	473.8633863	264.3950748	439.259606	-0.730971848	0.0044477	0.0276296	Ppp1r3e	14	54872669	54877550	-	2508	protein_coding	protein phosphatase 1, regulatory subunit 3E [Source:MGI Symbol;Acc:MGI:2145790]	-
ENSMUSG0000002804	469.9859687	506.6214251	539.5876208	685.6195441	701.2303525	693.7671343	505.3983382	693.5390103	-0.456898169	0.0044494	0.027631	Scamp3	3	89177473	89182765	+	2270	protein_coding	secretory carrier membrane protein 3 [Source:MGI Symbol;Acc:MGI:1346346]	-
ENSMUSG0000003094	1120.350347	957.4196995	1345.058997	750.2193755	723.9462653	860.1544835	1140.943015	778.1067081	0.552062049	0.0044625	0.0277034	Thumpd1	7	119715093	119720798	-	3004	protein_coding	THUMP domain containing 1 [Source:MGI Symbol;Acc:MGI:2444479]	-
ENSMUSG0000007666	0	0	0	0	26.66650636	11.67630521	0	12.78093719	-6.037231093	0.0044661	0.0277167	Ighv7-1	12	113896408	113896946	-	363	IG_V_gene	immunoglobulin heavy variable 7-1 [Source:MGI Symbol;Acc:MGI:4439622]	-
ENSMUSG0000000678	11.02312506	9.479402965	18.9916968	108.5277168	32.59239666	21.40655954	13.16474161	54.17555766	-2.047310155	0.0044691	0.0277257	Ttc25	11	100545607	100572568	+	6147	protein_coding	tetratricopeptide repeat domain 25 [Source:MGI Symbol;Acc:MGI:1921657]	-
ENSMUSG0000004928	1813.805124	1555.675353	1348.410473	1130.066384	1139.746235	1091.734537	1572.630317	1120.515719	0.489246206	0.0044713	0.0277304	Lix1l	3	96601149	96626171	+	3620	protein_coding	Lix1-like [Source:MGI Symbol;Acc:MGI:3036267]	-
ENSMUSG0000002779	3215.746212	2880.685234	1661.214891	2856.17388	5841.94019	6053.191224	2585.882112	4917.101764	-0.92700334	0.004477	0.0277564	Bche	3	73635808	73708415	-	6741	protein_coding	butyrylcholinesterase [Source:MGI Symbol;Acc:MGI:894278]	-
ENSMUSG0000003053	321.6748314	312.8202978	342.967701	794.1472609	421.7258599	468.9982591	325.8209434	561.6237933	-0.7864158	0.0044924	0.0278425	Cib1	7	80227147	80232813	-	2303	protein_coding	calcium and integrin binding 1 (calmyrin) [Source:MGI Symbol;Acc:MGI:1344418]	-
ENSMUSG0000008002	1.002102279	0	4.468634541	14.64262846	12.83942899	22.37958498	1.82357894	16.62054747	-3.213642286	0.0044953	0.0278512	Gm5915	8	105660334	105660787	-	454	processed_pseudog	predicted pseudogene 5915 [Source:MGI Symbol;Acc:MGI:3642938]	-
ENSMUSG0000008716	18.03784102	12.63920395	35.74907633	4.306655428	4.938241919	0	22.14204043	3.081632449	2.836515226	0.0044988	0.0278642	Gm16230	10	127671604	127676724	-	521	antisense	predicted gene 16230 [Source:MGI Symbol;Acc:MGI:3802022]	-
ENSMUSG0000002804	602.2634694	631.9601977	544.0562554	814.819207	788.1434102	828.0446441	592.7599742	810.3357538	-0.450840071	0.0045006	0.0278645	Gba	3	89202928	89208966	+	2559	protein_coding	glucosidase, beta, acid [Source:MGI Symbol;Acc:MGI:95665]	-
ENSMUSG0000005669	639.3412538	633.0134647	771.9566169	499.5720296	456.2935533	486.5127169	681.4371118	480.7927666	0.502572956	0.0045028	0.0278645	Elmod3	6	72565922	72598413	-	3560	protein_coding	ELMO/CED-12 domain containing 3 [Source:MGI Symbol;Acc:MGI:2445168]	-
ENSMUSG0000006415	22.04625013	23.17187391	16.75737953	5.167986514	1.975296767	5.838152603	20.65850119	4.327145295	2.250060393	0.0045033	0.0278645	Izumo1	7	45621811	45628823	+	2992	protein_coding	izumo sperm-egg fusion 1 [Source:MGI Symbol;Acc:MGI:1920706]	-
ENSMUSG0000003703	182.3826147	167.4694524	131.824719	355.7297383	237.0356121	237.4182058	160.5589287	276.7278521	-0.785501262	0.0045052	0.027867	Apbb1	7	105558483	105581653	-	4408	protein_coding	amyloid beta (A4) precursor protein-binding, family B, member 1 [Source:MGI Symbol;Acc:MGI:107765]	-
ENSMUSG0000001925	501.0511393	666.7180085	490.4326409	361.7590559	401.9728922	324.9904949	552.7339296	362.907481	0.607123043	0.004514	0.0279075	Ahr	12	35497974	35535038	-	6163	protein_coding	aryl-hydrocarbon receptor [Source:MGI Symbol;Acc:MGI:105043]	bHLI
ENSMUSG0000005336	0	0	0	31.00791908	1.975296767	0.973025434	0	11.31874709	-5.868186633	0.0045158	0.0279075	Rxfp2	5	150018675	150082184	+	5690	protein_coding	relaxin/insulin-like family peptide receptor 2 [Source:MGI Symbol;Acc:MGI:2153463]	-
ENSMUSG0000002810	2320.868877	1623.084441	2411.945493	3445.324342	2870.106203	3129.249795	2118.632937	3148.22678	-0.571498762	0.0045171	0.0279075	Ecm1	3	95734147	95739569	-	2521	protein_coding	extracellular matrix protein 1 [Source:MGI Symbol;Acc:MGI:103060]	-
ENSMUSG0000003126	3.006306836	6.319601977	3.351475906	23.25593931	18.76531929	18.48748324	4.225794906	20.16958061	-2.25656904	0.0045178	0.0279075	Cenpi	X	134308084	134362639	+	5681	protein_coding	centromere protein I [Source:MGI Symbol;Acc:MGI:2147897]	-
ENSMUSG0000003231	1031.163245	1174.392701	1098.166938	830.3231665	832.5875875	801.7729574	1101.240961	821.5612371	0.422568571	0.004523	0.0279309	Clk3	9	57750624	57765860	-	8450	protein_coding	CDC-like kinase 3 [Source:MGI Symbol;Acc:MGI:1098670]	-
ENSMUSG0000002561	86.18079596	55.82315079	113.9501808	191.215501	175.8014123	135.2505353	85.31804252	167.4224829	-0.974433346	0.0045256	0.0279377	Map3k7cl	16	87553330	87595336	+	1652	protein_coding	Map3k7 C-terminal like [Source:MGI Symbol;Acc:MGI:2446584]	-
ENSMUSG0000003123	0	1.053266996	0	47.37320971	6.913538686	0	0.351088999	18.0955828	-5.581971213	0.00453	0.0279554	Pgk2	17	40207015	40208640	-	1626	protein_coding	phosphoglycerate kinase 2 [Source:MGI Symbol;Acc:MGI:97563]	-
ENSMUSG0000003354	925.9425054	895.2769467	1031.13742	677.8675644	750.6127716	616.898125	950.7856241	681.7928203	0.4796921	0.004532	0.0279586	Znrf1	8	111536097	111626030	+	7018	protein_coding	zinc and ring finger 1 [Source:MGI Symbol;Acc:MGI:2177308]	-
ENSMUSG0000008728	10.02102279	10.53266996	18.9916968	1.722662171	1.975296767	0	13.18179652	1.23265298	3.40616323	0.0045402	0.0279921	4933424M12Ri	4	107783720	107789921	+	2141	lincRNA	RIKEN cDNA 4933424M12 gene [Source:MGI Symbol;Acc:MGI:1914798]	-
ENSMUSG0000003314	206.4330694	256.997147	258.0636447	170.5435549	145.1843124	106.0597723	240.4979537	140.5958799	0.772544304	0.0045407	0.0279921	Slc22a15	3	101855776	101924453	-	14239	protein_coding	solute carrier family 22 (organic anion/cation transporter), member 15 [Source:MGI Symbol;Acc:MGI:3607704]	-
ENSMUSG0000007370	4.008409114	2.106533992	1.117158635	21.53327714	13.82707737	13.62235607	2.410700581	16.3275702	-2.74656017	0.0045419	0.0279921	Cenps	4	149127121	149137629	-	1909	protein_coding	centromere protein S [Source:MGI Symbol;Acc:MGI:1917178]	-
ENSMUSG0000000101	927.94671	858.4126018	1257.920623	745.051389	600.4902173	658.7382187	1014.759978	668.093275	0.602411829	0.0045524	0.0280477	Ilf2	3	90476126	90488379	+	8580	protein_coding	interleukin enhancer binding factor 2 [Source:MGI Symbol;Acc:MGI:1915031]	-
ENSMUSG0000003293	620.3013105	490.8224202	617.7887253	802.7605718	890.8588421	778.420347	576.304152	824.0132536	-0.515758807	0.0045551	0.0280547	Hspa13	16	75745431	75767104	-	6834	protein_coding	heat shock protein 70 family, member 13 [Source:MGI Symbol;Acc:MGI:1309463]	-
ENSMUSG0000002795	951.9971647	923.7151556	955.1706331	763.1393418	667.6503074	624.6823285	943.6276511	685.1573259	0.461293383	0.0045572	0.0280586	Slc50a1	3	89268246	89270570	-	1466	protein_coding	solute carrier family 50 (sugar transporter), member 1 [Source:MGI Symbol;Acc:MGI:107417]	-
ENSMUSG0000002063	128.2690917	250.6775451	107.247229	304.0498732	330.8622086	308.4490625	162.0646219	314.4537148	-0.955810116	0.0045603	0.0280681	Cmpk2	12	26469204	26479837	+	4163	protein_coding	cytidine monophosphate (UMP-CMP) kinase 2, mitochondrial [Source:MGI Symbol;Acc:MGI:99830]	-
ENSMUSG0000002205	1079.264154	1147.007759	1140.618967	1434.116258	1435.053102	1623.979449	1122.29696	1497.716269	-0.416305616	0.0045682	0.0281078	Ppp2r2a	14	67014056	67072444	-	5381	protein_coding	protein phosphatase 2, regulatory subunit B, alpha [Source:MGI Symbol;Acc:MGI:1919228]	-
ENSMUSG0000004030	208.437274	216.9730012	355.256446	164.5142373	133.3325318	140.1156625	260.2222404	145.9874772	0.832075131	0.0045746	0.0281377	Rbm48	5	3583978	3596585	-	3071	protein_coding	RNA binding motif protein 48 [Source:MGI Symbol;Acc:MGI:2442653]	-
ENSMUSG0000006282	7074.842087	6504.976968	6277.314371	8659.822735	11871.53357	8236.660297	6619.044475	9589.338868	-0.534761268	0.0045771	0.0281441	Actg1	11	120345690	120348542	-	2658	protein_coding	actin, gamma, cytoplasmic 1 [Source:MGI Symbol;Acc:MGI:87906]	-
ENSMUSG0000003795	378.7946613	428.6796674	412.2315364	318.6925017	239.0109089	247.1484602	406.5686217	268.2839569	0.598326415	0.004585	0.0281832	Nsrp1	11	77044292	77078435	-	3628	protein_coding	nuclear speckle regulatory protein 1 [Source:MGI Symbol;Acc:MGI:2144305]	-
ENSMUSG0000003573	174.3657965	149.5639134	78.20110447	208.4421227	296.2945151	278.2852741	134.0436048	261.007304	-0.959050981	0.0045937	0.0282276	Dagla	19	10245265	10304877	-	5747	protein_coding	diacylglycerol lipase, alpha [Source:MGI Symbol;Acc:MGI:2677061]	-
ENSMUSG0000008383	24.05045469	32.65127688	23.46033134	42.20522319	85.92540939	70.05783123	26.72068764	66.06282127	-1.30326537	0.0046128	0.0283358	Ldha-ps2	4	119334389	119335445	-	1057	processed_pseudog	lactate dehydrogenase A, pseudogene 2 [Source:MGI Symbol;Acc:MGI:96761]	-
ENSMUSG0000002635	88.18500052	104.2734326	201.0885543	270.4579609	209.3814574	313.3141897	131.1823292	264.384536	-1.013149594	0.0046368	0.0284738	Rgs1	1	144242296	144249242	-	6947	protein_coding	regulator of G-protein signaling 1 [Source:MGI Symbol;Acc:MGI:1354694]	-
ENSMUSG0000003842	270.5676152	233.8252731	347.4363356	166.2368995	176.7890607	185.8478579	283.9430746	176.2912727	0.687370335	0.0046392	0.0284793	Poli	18	70508680	70530620	-	3863	protein_coding	polymerase (DNA directed), iota [Source:MGI Symbol;Acc:MGI:1347081]	-
ENSMUSG0000002034	1404.947395	1279.7194	1301.48981	1751.947428	1687.891088	1789.393773	1328.718868	1743.07743	-0.391511148	0.0046454	0.0285079	Mgat1	11	49244191	49263030	+	4733	protein_coding	mannoside acetylglucosaminyltransferase 1 [Source:MGI Symbol;Acc:MGI:96973]	-
ENSMUSG0000003556	322.6769337	650.9190036	415.5830123	261.84465	253.8256346	276.3392232	463.0596499	264.0031693	0.810493149	0.004651	0.0285325	Pcdh17	14	84443563	84539002	+	12991	protein_coding	protocadherin 17 [Source:MGI Symbol;Acc:MGI:2684924]	-
ENSMUSG0000004784	17.03573874	12.63920395	6.702951811	36.17590559	42.4688805	34.05589018	12.12596483	37.56689209	-1.623768901	0.0046617	0.0285817	Diras2	13	52504380	52531279	-	4786	protein_coding	DIRAS family, GTP-binding RAS-like 2 [Source:MGI Symbol;Acc:MGI:1915453]	-
ENSMUSG0000008517	185.3889215	169.5759864	137.4105121	94.74641942	105.6783771	91.46439078	164.12514	97.29639575	0.75572834	0.0046621	0.0285817	D830026I12Ril	6	17197751	17210152	+	2112	lincRNA	RIKEN cDNA D830026I12 gene [Source:MGI Symbol;Acc:MGI:2442531]	-
ENSMUSG0000003303	1006.110688	1534.610013	727.2702715	573.646503	741.7239362	539.0560903	1089.330324	618.1421765	0.817699014	0.0046636	0.0285817	Micall1	15	79108898	79136900	+	8341	protein_coding	microtubule associated monooxygenase, calponin and LIM domain containing -like 1 [Source:MGI Symbol;Acc:MGI:105870]	-
ENSMUSG0000002290	114.2396598	54.7698838	138.5276708	216.1941025	175.8014123	237.4182058	102.5124048	209.8045735	-1.034167038	0.0046746	0.0286402	Cd86	16	36603869	36666081	-	3661	protein_coding	CD86 antigen [Source:MGI Symbol;Acc:MGI:101773]	-
ENSMUSG0000003547	715.5010269	417.0937305	401.05995	745.051389	854.3158519	1011.946451	511.2182358	870.4378974	-0.767034975	0.0047001	0.0287867	Galm	17	80127471	80185101	+	2476	protein_coding	galactose mutarotase [Source:MGI Symbol;Acc:MGI:2442420]	-
ENSMUSG0000005857	115.241762	78.99502471	41.3348695	155.9009265	194.5667316	155.6840694	78.52388542	168.7172425	-1.100191032	0.00471	0.0288326	Gpc6	14	116925315	117976544	+	6425	protein_coding	glypican 6 [Source:MGI Symbol;Acc:MGI:1346322]	-
ENSMUSG0000002163	5.010511393	4.213067984	2.23431727	100.775737	4.938241919	5.838152603	3.819299883	37.18404385	-3.281570916	0.0047116	0.0288326	Ocln	13	100496507	100552718	-	4222	protein_coding	occludin [Source:MGI Symbol;Acc:MGI:106183]	-
ENSMUSG0000003132	11.02312506	3.159800988	2.23431727	55.12518948	8.888835454	35.02891562	5.472414441	33.01431352	-2.583809461	0.0047122	0.0288326	Dmrtc1a	X	102903216	102908687	-	1388	protein_coding	DMRT-like family C1a [Source:MGI Symbol;Acc:MGI:1918137]	-
ENSMUSG0000007849	16.03363646	3.159800988	10.05442772	0.861331086	0	0	9.749288388	0.287110362	4.858387473	0.0047183	0.0288602	Zfp978	4	147361303	147391004	+	1287	protein_coding	zinc finger protein 978 [Source:MGI Symbol;Acc:MGI:3701123]	zf-C2
ENSMUSG0000002110	932.9572214	1746.31668	893.7269082	751.0807066	711.1068363	574.0850059	1191.00027	678.7575163	0.811104851	0.0047201	0.028862	Prkch	12	73584796	73778185	+	7530	protein_coding	protein kinase C, eta [Source:MGI Symbol;Acc:MGI:97600]	-
ENSMUSG0000000147	5727.014522	5712.920187	5947.752574	4341.970002	4772.31699	4435.049927	5795.895761	4516.44564	0.359874282	0.0047354	0.0289463	Tcf25	8	123373753	123403835	+	5390	protein_coding	transcription factor 25 (basic helix-loop-helix) [Source:MGI Symbol;Acc:MGI:1914105]	-
ENSMUSG0000009498	258.5423879	371.8032496	415.5830123	224.8074133	227.1591283	167.3603746	348.6428833	206.4423054	0.754937647	0.0047407	0.028969	Rpl9-ps4	13	72454762	72455340	-	579	processed_pseudog	ribosomal protein L9, pseudogene 4 [Source:MGI Symbol;Acc:MGI:3704410]	-
ENSMUSG0000003404	43.09039798	15.79900494	37.9833936	80.96512205	101.7277835	62.27362776	32.29093217	81.65551111	-1.337714602	0.0047425	0.0289708	Galnt17	5	130872082	131308497	-	9408	protein_coding	polypeptide N-acetylgalactosaminyltransferase 17 [Source:MGI Symbol;Acc:MGI:2137594]	-
ENSMUSG0000004137	708.486311	786.7904461	771.9566169	620.1583816	465.1823887	414.5088348	755.744458	499.9498684	0.595190606	0.0047458	0.028981	Ccdc9	7	16274042	16286795	-	5507	protein_coding	coiled-coil domain containing 9 [Source:MGI Symbol;Acc:MGI:1921443]	-
ENSMUSG0000002148	3.006306836	5.26633498	3.351475906	23.25593931	14.81472576	19.46050868	3.874705907	19.17705791	-2.309759311	0.0047478	0.0289842	Mxd3	13	55325168	55329823	-	2369	protein_coding	Max dimerization protein 3 [Source:MGI Symbol;Acc:MGI:104987]	bHLI
ENSMUSG0000002847	1121.35245	873.1583398	961.8735849	675.2835711	730.859804	704.4704141	985.4614582	703.5379297	0.486529056	0.004759	0.029043	Hint2	4	43654227	43656466	-	1540	protein_coding	histidine triad nucleotide binding protein 2 [Source:MGI Symbol;Acc:MGI:1916167]	-
ENSMUSG0000004987	21.04414785	22.11860692	32.39760042	64.59983142	58.27125464	51.57034799	25.18678506	58.14714468	-1.211394477	0.0047614	0.0290483	Nlrc3	16	3945007	3976632	-	7069	protein_coding	NLR family, CARD domain containing 3 [Source:MGI Symbol;Acc:MGI:2444070]	-
ENSMUSG0000003186	510.0700598	541.379236	450.21493	614.129064	821.7234553	768.6900927	500.5547419	734.8475373	-0.553182513	0.0047759	0.029127	Ints10	8	68793929	68831667	+	6885	protein_coding	integrator complex subunit 10 [Source:MGI Symbol;Acc:MGI:1918135]	-
ENSMUSG0000007093	863.8121642	733.0738293	925.00735	1250.652736	1153.573312	1106.329918	840.6311145	1170.185322	-0.477487832	0.0047868	0.0291841	Rraga	4	86575668	86577285	+	1618	protein_coding	Ras-related GTP binding A [Source:MGI Symbol;Acc:MGI:1915691]	-
ENSMUSG0000003218	33.06937519	4.213067984	29.04612452	27.56259474	187.6531929	83.6801873	22.10952256	99.63199165	-2.170556985	0.0047902	0.0291954	Scg3	9	75643189	75684056	-	2738	protein_coding	secretogranin III [Source:MGI Symbol;Acc:MGI:103032]	-
ENSMUSG0000007653	11.02312506	0	0	16.36529063	45.43182565	45.73219539	3.674375022	35.84310389	-3.265538054	0.0047918	0.0291954	Igkv4-91	6	68768555	68769103	-	358	IG_V_gene	immunoglobulin kappa chain variable 4-91 [Source:MGI Symbol;Acc:MGI:3642277]	-
ENSMUSG0000003256	25.05255697	40.02414585	36.86623496	329.0284747	51.35771595	36.97496648	33.98097926	139.1203857	-2.035762877	0.0047953	0.0292025	Acpp	9	104288251	104337748	-	7330	protein_coding	acid phosphatase, prostate [Source:MGI Symbol;Acc:MGI:1928480]	-
ENSMUSG0000009706	179.3763079	174.8423214	223.431727	132.6449872	110.616619	105.0867468	192.5501188	116.1161177	0.727527868	0.0047961	0.0292025	9330151L19Ri	12	69197211	69199867	+	2545	bidirectional_prom	RIKEN cDNA 9330151L19 gene [Source:MGI Symbol;Acc:MGI:3041168]	-
ENSMUSG0000000882	162.3405691	115.8593696	173.1595885	99.91440593	58.27125464	76.86900927	150.4531757	78.35155661	0.938473417	0.0048052	0.0292484	Acyp1	12	85272398	85288438	-	1380	protein_coding	acylphosphatase 1, erythrocyte (common) type [Source:MGI Symbol;Acc:MGI:1913454]	-
ENSMUSG0000001976	43.09039798	23.17187391	15.64022089	124.0316763	62.22184818	51.57034799	27.30083093	79.27462416	-1.53552685	0.0048219	0.0293405	Ccdc170	10	4482502	4562231	+	3792	protein_coding	coiled-coil domain containing 170 [Source:MGI Symbol;Acc:MGI:2685067]	-
ENSMUSG0000003394	4.008409114	1.053266996	0	51.67986514	5.925890302	7.78420347	1.68722537	21.79665297	-3.670465216	0.0048323	0.0293933	Zswim5	4	116877376	116989264	+	5582	protein_coding	zinc finger SWIM-type containing 5 [Source:MGI Symbol;Acc:MGI:1921714]	-
ENSMUSG0000002514	921.9340963	800.482917	793.182631	650.3049696	594.564327	571.1659296	838.5332148	605.3450754	0.470018708	0.0048349	0.0293933	Mob2	7	142008535	142061065	-	6248	protein_coding	MOB kinase activator 2 [Source:MGI Symbol;Acc:MGI:1919891]	-
ENSMUSG0000007118	129.2711939	95.84729665	110.5987049	48.23454079	75.06127716	48.65127169	111.9057318	57.31569655	0.96786133	0.0048353	0.0293933	3830408C21Ri	13	107022553	107055497	+	4846	processed_transcrip	RIKEN cDNA 3830408C21 gene [Source:MGI Symbol;Acc:MGI:1917940]	-
ENSMUSG0000003258	1097.301995	1226.002783	1467.946447	888.8936803	939.2536129	855.2893563	1263.750408	894.4788832	0.498336595	0.004839	0.0294062	Rbm6	9	107773559	107873237	-	13944	protein_coding	RNA binding motif protein 6 [Source:MGI Symbol;Acc:MGI:1338037]	-
ENSMUSG0000002665	103.2165347	117.9659036	110.5987049	68.90648685	62.22184818	52.54337342	110.5937144	61.22390282	0.851273545	0.0048522	0.0294768	Dusp12	1	170873498	170885540	-	2743	protein_coding	dual specificity phosphatase 12 [Source:MGI Symbol;Acc:MGI:1890614]	-
ENSMUSG0000002907	145.3048304	229.6122051	154.1678917	337.6417856	255.8009314	301.6378845	176.3616424	298.3602005	-0.759105071	0.0048577	0.0295007	Cptp	4	155864723	155869440	-	2709	protein_coding	ceramide-1-phosphate transfer protein [Source:MGI Symbol;Acc:MGI:1933107]	-
ENSMUSG0000003015	28.0588638	32.65127688	44.68634541	6.890648685	14.81472576	11.67630521	35.13216203	11.12722655	1.66447638	0.0048769	0.0296081	Clec1b	6	129397297	129409335	+	5103	protein_coding	C-type lectin domain family 1, member b [Source:MGI Symbol;Acc:MGI:1913287]	-
ENSMUSG0000004399	728.5283566	769.9381741	675.8809743	1006.896039	932.3400743	994.4319933	724.7825017	977.8893689	-0.432065329	0.0048831	0.0296359	Mgat2	12	69184157	69186770	+	2614	protein_coding	mannoside acetylglucosaminyltransferase 2 [Source:MGI Symbol;Acc:MGI:2384966]	-
ENSMUSG0000002622	1701.569669	2165.516944	1495.875413	2223.956863	2901.710951	2901.561844	1787.654009	2675.743219	-0.581685295	0.0048858	0.029643	Psmd1	1	86064387	86139151	+	7265	protein_coding	proteasome (prosome, macropain) 26S subunit, non-ATPase, 1 [Source:MGI Symbol;Acc:MGI:1917497]	-
ENSMUSG0000010990	948.9908579	717.2748243	923.8901913	1216.199493	1162.462148	1296.069878	863.3852912	1224.910506	-0.504584349	0.0048911	0.029665	Chmp1b	18	67203531	67207880	+	4350	protein_coding	charged multivesicular body protein 1B [Source:MGI Symbol;Acc:MGI:1914314]	-
ENSMUSG0000008747	9.018920508	10.53266996	20.10885543	39.62122994	55.30830949	29.19076301	13.22014863	41.37343415	-1.651971214	0.0048946	0.0296771	4930506C21Ri	17	8293366	8311080	-	1984	processed_transcrip	RIKEN cDNA 4930506C21 gene [Source:MGI Symbol;Acc:MGI:1922310]	-
ENSMUSG0000007421	968.0308011	965.8458354	766.3708238	1034.458634	1578.262117	1582.139355	900.0824868	1398.286702	-0.635046318	0.0049017	0.02971	Dnajb14	3	137867675	137916557	+	15741	protein_coding	DnaJ heat shock protein family (Hsp40) member B14 [Source:MGI Symbol;Acc:MGI:1917854]	-
ENSMUSG0000002217	144.3027281	154.8302484	157.5193676	96.46908159	98.76483837	76.86900927	152.217448	90.70097641	0.746031642	0.0049039	0.0297138	4931414P19Ri	14	54583663	54605993	-	3078	protein_coding	RIKEN cDNA 4931414P19 gene [Source:MGI Symbol;Acc:MGI:1921609]	-
ENSMUSG0000002475	437.9186958	476.0766822	347.4363356	278.2099406	289.3809764	274.3931723	420.4772378	280.6613631	0.583767168	0.0049066	0.0297206	Tmem2	19	21778342	21858327	+	6667	protein_coding	transmembrane protein 2 [Source:MGI Symbol;Acc:MGI:1890373]	-
ENSMUSG0000004827	1406.951599	1887.454457	1818.734258	1062.021229	1339.251208	1115.087147	1704.380105	1172.119861	0.540179584	0.0049197	0.0297902	Rbm33	5	28317121	28419239	+	12502	protein_coding	RNA binding motif protein 33 [Source:MGI Symbol;Acc:MGI:1919670]	-
ENSMUSG0000002536	6077.75032	6687.192158	6173.418618	5334.223413	4370.344098	4147.034399	6312.787032	4617.200637	0.451154723	0.004922	0.029795	Rps26	10	128624534	128626747	-	1544	protein_coding	ribosomal protein S26 [Source:MGI Symbol;Acc:MGI:1351628]	-
ENSMUSG0000002771	20.04204557	24.22514091	39.10055223	78.38112879	46.41947404	81.73413644	27.78924624	68.84491309	-1.313911538	0.0049266	0.0298128	Ccna2	3	36564865	36572150	-	4083	protein_coding	cyclin A2 [Source:MGI Symbol;Acc:MGI:108069]	-
ENSMUSG0000000196	761.5977317	645.6526686	777.5424101	585.7051382	449.3800146	443.6995978	728.2642702	492.9282502	0.562288039	0.0049295	0.029821	Fam50a	X	74313033	74320149	+	2288	protein_coding	family with sequence similarity 50, member A [Source:MGI Symbol;Acc:MGI:1351626]	-
ENSMUSG0000002647	116.2438643	120.0724376	144.1134639	62.87716925	85.92540939	42.81311909	126.8099219	63.87189924	0.989391247	0.0049553	0.029962	Rgs16	1	153740349	153745468	+	2339	protein_coding	regulator of G-protein signaling 16 [Source:MGI Symbol;Acc:MGI:108407]	-
ENSMUSG0000005910	181.3805124	128.4985735	574.2195385	80.10379096	149.1349059	93.41044164	294.6995415	107.5497128	1.454334089	0.004956	0.029962	Ifitm6	7	141015699	141017924	-	905	protein_coding	interferon induced transmembrane protein 6 [Source:MGI Symbol;Acc:MGI:2686976]	-
ENSMUSG0000004702	138.2901144	171.6825204	229.0175202	88.71710182	115.5548609	95.35649251	179.663385	99.87615174	0.846914356	0.0049579	0.0299636	Mipol1	12	57230427	57497199	+	9342	protein_coding	mirror-image polydactyly 1 [Source:MGI Symbol;Acc:MGI:1920740]	-
ENSMUSG0000005603	3.006306836	3.159800988	17.87453816	0	0	0	8.013548663	0	5.532452249	0.0049702	0.0300282	Cyp3a11	5	145854426	145879964	-	5093	protein_coding	cytochrome P450, family 3, subfamily a, polypeptide 11 [Source:MGI Symbol;Acc:MGI:88609]	-
ENSMUSG0000006045	1550.252225	1620.977907	1355.113425	1829.467226	2279.49247	2166.927641	1508.781186	2091.962446	-0.471175355	0.0049944	0.0301647	Rnf14	18	38296635	38317847	+	3515	protein_coding	ring finger protein 14 [Source:MGI Symbol;Acc:MGI:1929668]	-
ENSMUSG0000002910	2708.682459	2065.456579	2625.322793	3299.759389	3367.880989	3549.596782	2466.487277	3405.74572	-0.465480279	0.0049975	0.0301736	Lrpap1	5	35091501	35105766	-	4116	protein_coding	low density lipoprotein receptor-related protein associated protein 1 [Source:MGI Symbol;Acc:MGI:96829]	-
ENSMUSG0000007950	83.17448913	52.6633498	102.7785944	31.86925017	40.49358373	35.02891562	79.53881112	35.79724984	1.152456415	0.0050187	0.0302917	Gm11131	17	35377021	35380292	-	3122	antisense	predicted gene 11131 [Source:MGI Symbol;Acc:MGI:3779386]	-
ENSMUSG0000007531	29.06096608	11.58593696	15.64022089	44.78921645	62.22184818	48.65127169	18.76237464	51.88744544	-1.46195439	0.0050248	0.0303172	Scn2a	2	65620771	65767447	+	12732	protein_coding	sodium channel, voltage-gated, type II, alpha [Source:MGI Symbol;Acc:MGI:98248]	-
ENSMUSG0000008714	56.1177276	38.97087886	31.28044179	86.99443964	84.937761	88.54531447	42.12301608	86.82583837	-1.039961826	0.0050261	0.0303172	A830082K12R	13	78198017	78279391	+	8255	antisense	RIKEN cDNA A830082K12 gene [Source:MGI Symbol;Acc:MGI:2443527]	-
ENSMUSG0000005109	161.3384669	190.6413263	145.2306226	226.5300755	271.6033055	294.8267064	165.7368052	264.3200292	-0.672204347	0.0050291	0.0303255	Mblac2	13	81711341	81753275	+	3957	protein_coding	metallo-beta-lactamase domain containing 2 [Source:MGI Symbol;Acc:MGI:1920102]	-
ENSMUSG0000003224	3240.798769	2492.029713	5219.365144	2445.318952	2067.148067	1932.428511	3650.731209	2148.29851	0.764842009	0.005031	0.0303269	Anp32a	9	62341293	62378812	+	3166	protein_coding	acidic (leucine-rich) nuclear phosphoprotein 32 family, member A [Source:MGI Symbol;Acc:MGI:108447]	-
ENSMUSG0000008503	252.5297742	228.5589382	268.1180725	184.3248523	139.2584221	151.7919677	249.7355949	158.458414	0.654571227	0.0050345	0.0303383	4933421O10Ri	4	33027142	33031323	-	3847	processed_transcrip	RIKEN cDNA 4933421O10 gene [Source:MGI Symbol;Acc:MGI:1918323]	-
ENSMUSG0000004447	64.13454583	45.29048083	173.1595885	27.56259474	30.6170999	38.92101735	94.19487171	32.366904	1.540461343	0.0050422	0.0303681	Lncpint	6	31082565	31220488	-	7378	lincRNA	long non-protein coding RNA, Trp53 induced transcript [Source:MGI Symbol;Acc:MGI:2673128]	-
ENSMUSG0000003348	129.2711939	186.4282583	212.2601407	91.30109507	106.6660254	100.2216197	175.986531	99.39624673	0.823764757	0.0050427	0.0303681	Catsper2	2	121392631	121413792	-	3695	protein_coding	cation channel, sperm associated 2 [Source:MGI Symbol;Acc:MGI:2387404]	-
ENSMUSG0000003583	76.15977317	87.42116068	77.08394583	127.4770007	138.2707737	140.1156625	80.22162656	135.2878123	-0.753490988	0.0050511	0.0304089	Polr3g	13	81673843	81710113	-	3936	protein_coding	polymerase (RNA) III (DNA directed) polypeptide G [Source:MGI Symbol;Acc:MGI:1914736]	-
ENSMUSG0000002010	1226.573189	1098.557477	2061.157682	966.413478	799.0075425	871.8307887	1462.096116	879.0839364	0.733597358	0.0050558	0.0304276	Anapc16	10	59987409	60003135	-	4890	protein_coding	anaphase promoting complex subunit 16 [Source:MGI Symbol;Acc:MGI:1289325]	-
ENSMUSG0000006154	3.006306836	2.106533992	1.117158635	10.33597303	16.79002252	17.51445781	2.076666488	14.88015112	-2.826218368	0.0050575	0.0304279	Orm2	4	63362449	63365878	+	775	protein_coding	orosomucoid 2 [Source:MGI Symbol;Acc:MGI:97444]	-
ENSMUSG0000002413	2137.48416	3042.888352	1951.676136	1457.372197	1660.236933	1622.033398	2377.349549	1579.880843	0.589667482	0.0050603	0.0304338	Abca3	17	24351950	24410201	+	8797	protein_coding	ATP-binding cassette, sub-family A (ABC1), member 3 [Source:MGI Symbol;Acc:MGI:1351617]	-
ENSMUSG0000001737	474.9964801	460.2776773	421.1688055	560.7265367	700.2427041	674.3066256	452.1476543	645.0919555	-0.511906367	0.0050617	0.0304338	Nlk	11	78567168	78697373	-	5447	protein_coding	nemo like kinase [Source:MGI Symbol;Acc:MGI:1201387]	-
ENSMUSG0000005734	412.8661388	443.4254054	358.6079219	580.5371517	546.1695562	594.51854	404.9664887	573.7417493	-0.502258296	0.0050657	0.0304477	Sphk2	7	45709467	45718002	-	4159	protein_coding	sphingosine kinase 2 [Source:MGI Symbol;Acc:MGI:1861380]	-
ENSMUSG0000003687	53.11142077	69.51562174	54.74077313	113.6957033	107.6536738	101.1946451	59.12260521	107.5146741	-0.863270049	0.0050894	0.0305807	Dna2	10	62947026	62974185	+	5878	protein_coding	DNA replication helicase/nuclease 2 [Source:MGI Symbol;Acc:MGI:2443732]	-
ENSMUSG0000002905	2796.86746	2190.795352	1759.52485	2977.621563	3991.087119	3364.72195	2249.062554	3444.476877	-0.614728996	0.0050979	0.0306215	Ski	4	155154075	155222592	-	5562	protein_coding	ski sarcoma viral oncogene homolog (avian) [Source:MGI Symbol;Acc:MGI:98310]	-
ENSMUSG0000003180	1893.973307	2386.703013	1421.025784	1264.434034	1270.115821	1208.497589	1900.567368	1247.682481	0.607284271	0.005101	0.0306306	Pgls	8	71592176	71601092	+	3770	protein_coding	6-phosphogluconolactonase [Source:MGI Symbol;Acc:MGI:1913421]	-
ENSMUSG0000002009	363.7631271	223.2926032	186.5654921	609.8224086	414.8123212	393.1022752	257.8737408	472.5790017	-0.873382745	0.0051048	0.0306433	Unc5b	10	60762593	60831581	-	6258	protein_coding	unc-5 netrin receptor B [Source:MGI Symbol;Acc:MGI:894703]	-
ENSMUSG0000004603	13.02732962	7.372868973	3.351475906	23.25593931	32.59239666	36.00194105	7.917224833	30.61675901	-1.939182984	0.005108	0.0306507	Calhm6	10	34126067	34127984	-	1094	protein_coding	calcium homeostasis modulator family member 6 [Source:MGI Symbol;Acc:MGI:2443082]	-
ENSMUSG0000003126	91.19130735	65.30255376	69.26383538	118.0023587	148.1472576	137.1965862	75.2525655	134.4487342	-0.834834112	0.0051093	0.0306507	Btk	X	134542336	134583570	-	3149	protein_coding	Bruton agammaglobulinemia tyrosine kinase [Source:MGI Symbol;Acc:MGI:88216]	-
ENSMUSG0000011042	74.15556862	68.46235475	146.3477812	35.31457451	49.38241919	40.86706822	96.32190153	41.85468731	1.202384995	0.0051206	0.0307088	1700012D14Ri	7	111083064	111122684	-	4153	processed_transcrip	RIKEN cDNA 1700012D14 gene [Source:MGI Symbol;Acc:MGI:1922729]	-
ENSMUSG0000003836	3716.797151	2650.019762	3008.508205	4034.474805	4776.267584	4547.920877	3125.108439	4452.887755	-0.510705788	0.0051334	0.0307695	Lasp1	11	97799000	97838764	+	6495	protein_coding	LIM and SH3 protein 1 [Source:MGI Symbol;Acc:MGI:109656]	-
ENSMUSG0000002803	307.6453995	345.4715747	326.2103215	380.7083398	572.8360626	588.6803874	326.4424319	514.0749299	-0.654516266	0.005134	0.0307695	Tbck	3	132684144	132841688	+	12127	protein_coding	TBC1 domain containing kinase [Source:MGI Symbol;Acc:MGI:2445052]	-
ENSMUSG0000006655	1603.363646	1886.40119	1870.123555	1402.247007	1280.979954	941.8886199	1786.629464	1208.37186	0.563868632	0.0051555	0.0308709	Hmgb1	5	149046702	149184489	-	5023	protein_coding	high mobility group box 1 [Source:MGI Symbol;Acc:MGI:96113]	HMC
ENSMUSG0000003856	1164.442848	922.6618886	1337.238886	883.7256938	670.6132526	713.227643	1141.447874	755.8555298	0.594152816	0.0051555	0.0308709	Ift172	5	31253277	31291116	-	6617	protein_coding	intraflagellar transport 172 [Source:MGI Symbol;Acc:MGI:2682064]	-
ENSMUSG0000006322	12382.97786	9219.246017	14165.57149	14072.42728	22304.06345	20129.95017	11922.59846	18835.4803	-0.659744852	0.0051559	0.0308709	Ldha	7	46841475	46855627	+	3310	protein_coding	lactate dehydrogenase A [Source:MGI Symbol;Acc:MGI:96759]	-
ENSMUSG0000001948	1271.667792	1224.949516	1114.924318	960.3841604	876.0441164	825.1255678	1203.847209	887.1846149	0.440232107	0.0051578	0.0308728	Trip10	17	57249455	57263665	+	3180	protein_coding	thyroid hormone receptor interactor 10 [Source:MGI Symbol;Acc:MGI:2146901]	-
ENSMUSG0000006141	1892.971204	2325.613527	1806.445513	1355.735129	1576.28682	1372.938887	2008.343415	1434.986945	0.485123884	0.0051758	0.030965	Zcchc14	8	121598703	121652901	-	7780	protein_coding	zinc finger, CCHC domain containing 14 [Source:MGI Symbol;Acc:MGI:2159407]	-
ENSMUSG0000008567	1.002102279	0	0	4.306655428	16.79002252	8.757228904	0.334034093	9.951302285	-4.713265164	0.0051782	0.030965	Gm12022	11	18467479	18493255	-	1340	lincRNA	predicted gene 12022 [Source:MGI Symbol;Acc:MGI:3650976]	-
ENSMUSG0000003119	70.1471595	103.2201656	81.55258037	154.1782643	132.3448834	159.5761711	84.97330183	148.699773	-0.808225007	0.0051782	0.030965	F8	X	75172715	75382615	-	9905	protein_coding	coagulation factor VIII [Source:MGI Symbol;Acc:MGI:88383]	-
ENSMUSG0000003734	1.002102279	3.159800988	0	11.19730411	15.80237414	14.59538151	1.387301089	13.86501992	-3.307877369	0.0051866	0.0310051	Slc9a7	X	20105754	20291807	-	3820	protein_coding	solute carrier family 9 (sodium/hydrogen exchanger), member 7 [Source:MGI Symbol;Acc:MGI:2444530]	-
ENSMUSG0000002437	43.09039798	13.69247095	13.40590362	58.57051382	73.0859804	72.97690753	23.39625752	68.21113392	-1.536972206	0.0051896	0.0310135	Tslp	18	32815383	32819799	+	1133	protein_coding	thymic stromal lymphopoietin [Source:MGI Symbol;Acc:MGI:1855696]	-
ENSMUSG0000002737	380.7988659	339.1519727	326.2103215	732.1314228	426.6641018	532.2449123	348.7203867	563.6801456	-0.693257923	0.005193	0.0310236	Nphp1	2	127740732	127788897	-	2708	protein_coding	nephronophthisis 1 (juvenile) homolog (human) [Source:MGI Symbol;Acc:MGI:1858233]	-
ENSMUSG0000003271	275.5781266	291.7549579	208.9086648	164.5142373	135.3078286	177.0906289	258.7472498	158.9708983	0.70303378	0.0051968	0.0310365	Trib3	2	152337422	152344032	-	2125	protein_coding	tribbles pseudokinase 3 [Source:MGI Symbol;Acc:MGI:1345675]	-
ENSMUSG0000002633	1956.103648	1690.493529	1695.846808	2495.276155	2421.713837	2219.471014	1780.814662	2378.820335	-0.417640077	0.0052079	0.0310928	Pam	1	97795114	98095646	-	11892	protein_coding	peptidylglycine alpha-amidating monooxygenase [Source:MGI Symbol;Acc:MGI:97475]	-
ENSMUSG0000004046	2051.303364	1908.519797	2199.685353	1501.300082	1626.656888	1539.326236	2053.169505	1555.761069	0.400254527	0.0052324	0.0312293	Mybbp1a	11	72441355	72457168	+	5803	protein_coding	MYB binding protein (P160) 1a [Source:MGI Symbol;Acc:MGI:106181]	-
ENSMUSG0000008624	61.128239	97.95383064	108.3643876	31.00791908	51.35771595	39.89404278	89.14881908	40.75322594	1.130801885	0.0052381	0.0312534	Gm15787	5	110154400	110176504	-	2992	processed_transcrip	predicted gene 15787 [Source:MGI Symbol;Acc:MGI:3783229]	-
ENSMUSG0000003235	1080.266256	871.0518058	580.9224903	1771.758043	1168.388038	1327.206692	844.0801841	1422.450924	-0.752752267	0.0052476	0.0313	Ctsh	9	90054152	90076089	+	4499	protein_coding	cathepsin H [Source:MGI Symbol;Acc:MGI:107285]	-
ENSMUSG0000001392	27.05676152	35.81107787	15.64022089	7.75197977	6.913538686	5.838152603	26.16935343	6.83455702	1.935954917	0.0052501	0.0313047	Thap8	7	30280094	30290237	+	2432	protein_coding	THAP domain containing 8 [Source:MGI Symbol;Acc:MGI:1922879]	-
ENSMUSG0000003395	9.018920508	5.26633498	22.3431727	67.18382468	22.71591283	50.59732256	12.20947606	46.83235335	-1.948116887	0.0052555	0.0313268	Aspm	1	139454772	139494091	+	11590	protein_coding	abnormal spindle microtubule assembly [Source:MGI Symbol;Acc:MGI:1334448]	-
ENSMUSG0000002892	30.06306836	29.49147589	35.74907633	163.6529063	52.34536434	53.51639886	31.76787353	89.83822315	-1.50314284	0.0052811	0.0314696	Padi2	4	140906344	140952586	+	5013	protein_coding	peptidyl arginine deiminase, type II [Source:MGI Symbol;Acc:MGI:1338892]	-
ENSMUSG0000009823	320.6727292	403.4012595	414.4658537	240.3113729	264.6897668	258.8247654	379.5132808	254.608635	0.575706562	0.0052848	0.0314819	Snhg6	1	9941959	9944118	-	1146	lincRNA	small nucleolar RNA host gene 6 [Source:MGI Symbol;Acc:MGI:1921074]	-
ENSMUSG0000005274	54.11352305	67.40908775	54.74077313	10.33597303	29.62945151	26.27168671	58.75446131	22.07903708	1.419189786	0.0053037	0.0315844	Trim30b	7	104355382	104369884	-	7527	protein_coding	tripartite motif-containing 30B [Source:MGI Symbol;Acc:MGI:4821256]	-
ENSMUSG0000002017	30.06306836	27.3849419	23.46033134	166.2368995	38.51828697	51.57034799	26.9694472	85.44184483	-1.665315979	0.0053069	0.0315932	Rab36	10	75037058	75054748	+	6175	protein_coding	RAB36, member RAS oncogene family [Source:MGI Symbol;Acc:MGI:1924127]	-
ENSMUSG0000001879	33819.9498	25463.7829	17242.22638	8782.99308	16020.64443	17450.23813	25508.65302	14084.62521	0.856897185	0.0053247	0.0316891	Acsl1	8	46471037	46536051	+	10483	protein_coding	acyl-CoA synthetase long-chain family member 1 [Source:MGI Symbol;Acc:MGI:102797]	-
ENSMUSG0000003663	535.1226168	555.0717069	468.0894682	708.8754834	700.2427041	736.5802534	519.4279306	715.2328136	-0.46110486	0.0053467	0.0318096	Alg5	3	54735539	54751318	+	3728	protein_coding	asparagine-linked glycosylation 5 (dolichyl-phosphate beta-glucosyltransferase) [Source:MGI Symbol;Acc:MGI:1913498]	-
ENSMUSG0000003243	1203.524837	934.2478255	970.810854	1659.785002	1333.325318	1441.050667	1036.194505	1478.053662	-0.512375781	0.0053511	0.0318194	Crtap	9	114375134	114390675	-	1772	protein_coding	cartilage associated protein [Source:MGI Symbol;Acc:MGI:1891221]	-
ENSMUSG0000002590	933.9593237	1005.869981	1015.497199	1180.884918	1546.657369	1384.615192	985.1088348	1370.71916	-0.476392656	0.005353	0.0318194	Lypla1	1	4807788	4848410	+	7145	protein_coding	lysophospholipase 1 [Source:MGI Symbol;Acc:MGI:1344588]	-
ENSMUSG0000003911	338.7105702	310.7137638	239.0719479	518.5213135	507.6512692	377.5338683	296.1654273	467.9021504	-0.659311775	0.0053546	0.0318194	Itga9	9	118606690	118901003	+	7691	protein_coding	integrin alpha 9 [Source:MGI Symbol;Acc:MGI:104756]	-
ENSMUSG0000002018	976.0476194	1280.772667	913.8357636	1310.945912	1692.82933	1744.634603	1056.88535	1582.803282	-0.582399498	0.0053551	0.0318194	Osbpl8	10	111164752	111297249	+	9401	protein_coding	oxysterol binding protein-like 8 [Source:MGI Symbol;Acc:MGI:2443807]	-
ENSMUSG0000001369	2369.971889	1591.486431	2057.806206	2784.6834	2873.069148	3015.405819	2006.421509	2891.052789	-0.526873664	0.005368	0.0318857	Pea15a	1	172196728	172206804	-	3861	protein_coding	phosphoprotein enriched in astrocytes 15A [Source:MGI Symbol;Acc:MGI:104799]	-
ENSMUSG0000001950	18989.83818	14714.13994	16702.63876	13809.7213	10275.49378	11386.34363	16802.20562	11823.8529	0.506923408	0.0053702	0.0318892	Ubb	11	62551171	62553213	+	1675	protein_coding	ubiquitin B [Source:MGI Symbol;Acc:MGI:98888]	-
ENSMUSG0000003178	229.4814218	171.6825204	166.4566366	374.6790222	284.4427345	269.5280452	189.2068596	309.549934	-0.710070102	0.0054026	0.0320713	Katnb1	8	95081186	95103149	+	6085	protein_coding	katanin p80 (WD40-containing) subunit B 1 [Source:MGI Symbol;Acc:MGI:1921437]	-
ENSMUSG0000005650	1282.690917	1307.104342	2018.705654	881.1417006	1155.548609	859.181458	1536.166971	965.2905892	0.670235267	0.0054078	0.0320884	Cebpb	2	167688915	167690418	+	1504	protein_coding	CCAAT/enhancer binding protein (C/EBP), beta [Source:MGI Symbol;Acc:MGI:88373]	C/EB
ENSMUSG0000002525	72.15136406	80.0482917	73.73246992	45.65054754	17.77767091	7.78420347	75.31070856	23.73747397	1.657626248	0.00541	0.0320884	Ribc1	X	152004582	152016295	-	1547	protein_coding	RIB43A domain with coiled-coils 1 [Source:MGI Symbol;Acc:MGI:1913861]	-
ENSMUSG0000002171	936.9656305	860.5191358	958.522109	596.9024423	730.859804	669.4414984	918.6689584	665.7345816	0.465016068	0.0054107	0.0320884	Trim23	13	104178797	104203372	+	5747	protein_coding	tripartite motif-containing 23 [Source:MGI Symbol;Acc:MGI:1933161]	-
ENSMUSG0000002972	1050.203188	878.4246747	1101.518414	785.5339501	590.6137335	713.227643	1010.048759	696.4584422	0.535824501	0.0054159	0.0321092	Mepce	5	137781906	137787655	-	3553	protein_coding	methylphosphate capping enzyme [Source:MGI Symbol;Acc:MGI:106477]	-
ENSMUSG0000002549	3646.650192	3907.620556	3993.842121	2940.584326	3147.635399	2594.085806	3849.370956	2894.101844	0.411461043	0.0054185	0.0321147	Ifitm3	7	141009586	141010770	-	678	protein_coding	interferon induced transmembrane protein 3 [Source:MGI Symbol;Acc:MGI:1913391]	-
ENSMUSG0000000345	1643.447737	1766.328752	1564.022089	2287.695363	2059.24688	2197.091429	1657.93286	2181.344558	-0.395865132	0.0054236	0.0321311	Ncstn	1	172066013	172082795	-	3975	protein_coding	nicastrin [Source:MGI Symbol;Acc:MGI:1891700]	-
ENSMUSG0000003021	17.03573874	14.74573795	2.23431727	135.2289804	23.70356121	23.35261041	11.33859798	60.76171735	-2.418649031	0.0054247	0.0321311	Art4	6	136848451	136857733	-	2517	protein_coding	ADP-ribosyltransferase 4 [Source:MGI Symbol;Acc:MGI:1202710]	-
ENSMUSG0000006615	10.02102279	11.58593696	23.46033134	1.722662171	1.975296767	2.919076301	15.02243036	2.205678413	2.77044466	0.0054372	0.0321946	Mup3	4	62083476	62087342	-	1068	protein_coding	major urinary protein 3 [Source:MGI Symbol;Acc:MGI:97235]	-
ENSMUSG0000003476	1266.65728	1543.036149	1198.711216	898.3683223	990.6113289	988.5938407	1336.134882	959.191164	0.478418265	0.0054433	0.0322205	Map4k5	12	69803750	69893200	-	8614	protein_coding	mitogen-activated protein kinase kinase kinase kinase 5 [Source:MGI Symbol;Acc:MGI:1925503]	-
ENSMUSG0000001994	1623.405691	1706.292534	1245.631878	1970.725524	2135.295806	2587.274628	1525.110034	2231.098653	-0.548577769	0.0054452	0.0322218	Atp2b1	10	98914406	99026143	+	15218	protein_coding	ATPase, Ca++ transporting, plasma membrane 1 [Source:MGI Symbol;Acc:MGI:104653]	-
ENSMUSG0000003686	861.8079596	1004.816714	786.4796792	648.5823075	553.0830949	660.6842695	884.3681177	620.783224	0.510477592	0.0054475	0.0322251	Syde2	3	145987870	146021720	+	5751	protein_coding	synapse defective 1, Rho GTPase, homolog 2 (C. elegans) [Source:MGI Symbol;Acc:MGI:3036264]	-
ENSMUSG0000005614	19.03994329	5.26633498	21.22601407	41.34389211	73.0859804	36.97496648	15.17743078	50.46827966	-1.733688404	0.0054532	0.0322489	AI504432	3	107039504	107054347	+	5569	lincRNA	expressed sequence AI504432 [Source:MGI Symbol;Acc:MGI:2139742]	-
ENSMUSG0000010731	10.02102279	9.479402965	2.23431727	0	0	0	7.244914341	0	5.392912573	0.0054594	0.0322754	Gm43332	5	149183183	149184226	+	1044	lincRNA	predicted gene 43332 [Source:MGI Symbol;Acc:MGI:5663469]	-
ENSMUSG0000004006	33.06937519	22.11860692	30.16328315	72.35181119	57.28360626	58.38152603	28.45042175	62.67231449	-1.140078861	0.0054618	0.032279	Plcb2	2	118707517	118728438	-	6597	protein_coding	phospholipase C, beta 2 [Source:MGI Symbol;Acc:MGI:107465]	-
ENSMUSG0000003587	347.7294907	444.4786724	427.8717573	324.7218193	164.9372801	202.3892902	406.6933068	230.6827965	0.816175677	0.0054639	0.0322815	Pawr	10	108332121	108414240	+	1745	protein_coding	PRKC, apoptosis, WT1, regulator [Source:MGI Symbol;Acc:MGI:2149961]	-
ENSMUSG0000011466	33.06937519	43.18394684	23.46033134	12.0586352	11.8517806	7.78420347	33.23788446	10.56487309	1.652434285	0.005466	0.0322838	Gm30918	13	37541357	37549725	+	2372	lincRNA	predicted gene, 30918 [Source:MGI Symbol;Acc:MGI:5590077]	-
ENSMUSG0000010950	63.13244355	7.372868973	108.3643876	9.474641942	18.76531929	7.78420347	59.62323338	12.0080549	2.313437388	0.0054678	0.0322841	Gm44956	8	10962957	10970814	-	2887	lincRNA	predicted gene 44956 [Source:MGI Symbol;Acc:MGI:5753532]	-
ENSMUSG0000005010	1373.882224	1224.949516	1404.268404	622.7423749	944.1918549	1029.460909	1334.366715	865.4650462	0.625085408	0.0054755	0.0323195	Agmo	12	37241641	37582202	+	6397	protein_coding	alkylglycerol monooxygenase [Source:MGI Symbol;Acc:MGI:2442495]	-
ENSMUSG0000002607	33.06937519	9.479402965	33.51475906	93.88508833	54.32066111	67.13875493	25.35451241	71.78150146	-1.503052709	0.0054911	0.0324013	Il18r1	1	40465552	40500854	+	4817	protein_coding	interleukin 18 receptor 1 [Source:MGI Symbol;Acc:MGI:105383]	-
ENSMUSG0000000958	529.1100031	1181.76557	663.5922293	509.9080027	410.8617276	389.2101735	791.4892674	436.6599679	0.857687435	0.0054988	0.032431	Apobec3	15	79891659	79915906	+	9454	protein_coding	apolipoprotein B mRNA editing enzyme, catalytic polypeptide 3 [Source:MGI Symbol;Acc:MGI:1933111]	-
ENSMUSG0000002860	21274.63138	17302.01694	20288.71797	13069.83789	15281.88344	15214.22568	19621.78876	14521.98234	0.434243364	0.0054996	0.032431	Scp2	4	108043839	108144998	-	3478	protein_coding	sterol carrier protein 2, liver [Source:MGI Symbol;Acc:MGI:98254]	-
ENSMUSG0000007994	232.4877286	157.9900494	204.4400302	131.7836561	79.0118707	118.7091029	198.3059361	109.8348766	0.850972391	0.0055067	0.0324625	Gm11273	13	21501029	21501418	-	390	protein_coding	predicted gene 11273 [Source:MGI Symbol;Acc:MGI:3649411]	-
ENSMUSG0000002275	1065.234722	1122.782618	1026.668786	1302.332601	1453.818421	1705.713585	1071.562042	1487.288203	-0.472706685	0.0055135	0.032492	Tfg	16	56690332	56717450	-	2292	protein_coding	Trk-fused gene [Source:MGI Symbol;Acc:MGI:1338041]	-
ENSMUSG0000002673	594.2466512	542.432503	462.503675	732.9927538	720.9833201	808.5841355	533.0609431	754.1867365	-0.500008646	0.0055164	0.0324991	Pip4k2a	2	18842255	18998126	-	3477	protein_coding	phosphatidylinositol-5-phosphate 4-kinase, type II, alpha [Source:MGI Symbol;Acc:MGI:1298206]	-
ENSMUSG0000003207	0	16.85227194	15.64022089	0	0	0	10.83083094	0	5.969162523	0.0055188	0.0325032	Apoa5	9	46268633	46271919	+	2522	protein_coding	apolipoprotein A-V [Source:MGI Symbol;Acc:MGI:1913363]	-
ENSMUSG0000007467	70.1471595	53.7166168	72.61531129	149.8716089	98.76483837	118.7091029	65.4930292	122.4485167	-0.904567627	0.0055243	0.0325252	Foxs1	2	152931898	152933208	-	1311	protein_coding	forkhead box S1 [Source:MGI Symbol;Acc:MGI:95546]	Fork
ENSMUSG0000003726	317.6664223	309.6604969	502.7213858	248.0633527	212.3444025	220.8767735	376.6827683	227.0948429	0.728816103	0.0055356	0.0325815	Kin	2	10080593	10092806	+	1705	protein_coding	Kin17 DNA and RNA binding protein [Source:MGI Symbol;Acc:MGI:96676]	-
ENSMUSG0000007649	15.03153418	8.426135969	16.75737953	58.57051382	36.5429902	28.21773758	13.40501656	41.11041387	-1.620602832	0.0055536	0.0326772	Trbc2	6	41546730	41548352	+	692	TR_C_gene	T cell receptor beta, constant 2 [Source:MGI Symbol;Acc:MGI:4835227]	-
ENSMUSG0000003207	112.2354552	132.7116415	87.13837355	568.4785165	123.456048	184.8748324	110.6951568	292.269799	-1.401414375	0.0055626	0.03272	Cadm1	9	47530173	47857637	+	10440	protein_coding	cell adhesion molecule 1 [Source:MGI Symbol;Acc:MGI:1889272]	-
ENSMUSG0000002048	666.3980153	651.9722706	852.3920387	568.4785165	449.3800146	426.18514	723.5874415	481.3478904	0.587117	0.0055723	0.0327665	Supt4a	11	87737552	87743623	+	942	protein_coding	suppressor of Ty 4A [Source:MGI Symbol;Acc:MGI:107416]	-
ENSMUSG0000011338	17.03573874	11.58593696	15.64022089	2.583993257	0	2.919076301	14.75396553	1.834356519	2.993149796	0.0055832	0.0328123	Gm9512	13	15118632	15119128	+	497	processed_pseudog	predicted gene 9512 [Source:MGI Symbol;Acc:MGI:3779922]	-
ENSMUSG0000004519	1718.605408	1766.328752	1221.054388	1273.047345	804.9334328	851.3972546	1568.66285	976.4593439	0.683627928	0.0055844	0.0328123	Cirbp	10	80165985	80172786	+	2604	protein_coding	cold inducible RNA binding protein [Source:MGI Symbol;Acc:MGI:893588]	-
ENSMUSG0000003259	872.8310847	831.0276599	885.9067977	601.2090977	703.2056492	520.5686071	863.2551808	608.3277847	0.505029616	0.0055853	0.0328123	Uba7	9	107975505	107984060	+	6692	protein_coding	ubiquitin-like modifier activating enzyme 7 [Source:MGI Symbol;Acc:MGI:1349462]	-
ENSMUSG0000009061	23.04835241	24.22514091	13.40590362	49.09587188	50.37006757	49.62429712	20.22646565	49.69674552	-1.292915201	0.0055882	0.0328188	Gm3571	10	93778660	93779719	+	1060	processed_pseudog	predicted gene 3571 [Source:MGI Symbol;Acc:MGI:3781748]	-
ENSMUSG0000002262	102.2144324	96.90056364	103.8957531	277.3486096	144.196664	153.7380185	101.003583	191.7610974	-0.926696797	0.0055932	0.0328379	Rabl2	15	89582533	89591923	-	2960	protein_coding	RAB, member RAS oncogene family-like 2 [Source:MGI Symbol;Acc:MGI:1915958]	-
ENSMUSG0000002014	74.15556862	28.43820889	116.1844981	236.8660485	125.4313447	186.8208833	72.92609186	183.0394255	-1.329684089	0.0055979	0.0328554	Slc1a4	11	20302180	20332713	-	3926	protein_coding	solute carrier family 1 (glutamate/neutral amino acid transporter), member 4 [Source:MGI Symbol;Acc:MGI:2135601]	-
ENSMUSG0000000637	4639.73355	4595.403904	4021.771087	2906.131083	3627.632513	3245.039822	4418.969514	3259.601139	0.439176191	0.0056196	0.0329719	Pgrmc1	X	36598206	36606079	+	1857	protein_coding	progesterone receptor membrane component 1 [Source:MGI Symbol;Acc:MGI:1858305]	-
ENSMUSG0000004220	519.0889803	588.7762508	532.884669	659.7796116	830.6122907	855.2893563	546.9166334	781.8937529	-0.515225546	0.0056237	0.0329862	0610010F05Ril	11	23564961	23633639	-	5226	protein_coding	RIKEN cDNA 0610010F05 gene [Source:MGI Symbol;Acc:MGI:1918925]	-
ENSMUSG0000003398	75.1576709	93.74076265	80.43542174	204.1354673	115.5548609	144.0077642	83.1112851	154.5660308	-0.897022019	0.0056261	0.0329899	Tesk2	4	116720948	116805956	+	4272	protein_coding	testis-specific kinase 2 [Source:MGI Symbol;Acc:MGI:2385204]	-
ENSMUSG0000005862	3869.116898	7165.375374	5475.194471	2198.11693	3803.433926	3611.87041	5503.228914	3204.473755	0.780266676	0.0056316	0.0330113	Gda	19	21391307	21473445	-	6135	protein_coding	guanine deaminase [Source:MGI Symbol;Acc:MGI:95678]	-
ENSMUSG0000002252	646.3559697	575.0837799	661.3579121	471.1481038	450.367663	446.6186741	627.5992205	456.0448136	0.460390439	0.0056379	0.0330358	Zfp263	16	3744093	3750790	+	4521	protein_coding	zinc finger protein 263 [Source:MGI Symbol;Acc:MGI:1921370]	zf-C2
ENSMUSG0000002267	429.9018775	420.2535314	423.4031227	296.2978934	286.4180313	323.044444	424.5195106	301.9201229	0.491798547	0.0056393	0.0330358	Mcm4	16	15623897	15637400	-	3589	protein_coding	minichromosome maintenance complex component 4 [Source:MGI Symbol;Acc:MGI:103199]	-
ENSMUSG0000008738	142.2985236	176.9488553	237.9547893	110.250379	113.5795641	90.49136534	185.7340561	104.7737695	0.82438989	0.0056477	0.0330751	Frg2f1	4	119530308	119539529	-	2760	protein_coding	FSHD region gene 2 family member 1 [Source:MGI Symbol;Acc:MGI:3035485]	-
ENSMUSG0000002737	862.8100619	706.7421544	655.7721189	515.9373203	551.1077981	488.4587678	741.7747784	518.5012954	0.517095878	0.005668	0.0331836	Mrps5	2	127587222	127606829	+	6578	protein_coding	mitochondrial ribosomal protein S5 [Source:MGI Symbol;Acc:MGI:1924971]	-
ENSMUSG0000003559	1268.661485	1646.256315	1766.227802	989.6694173	1170.363335	1096.599664	1560.381867	1085.544139	0.523453339	0.0056857	0.0332766	Prpf39	12	65036333	65063386	+	11967	protein_coding	pre-mRNA processing factor 39 [Source:MGI Symbol;Acc:MGI:104602]	-
ENSMUSG0000003957	78.16397773	104.2734326	82.66973901	56.84785165	30.6170999	37.94799192	88.36904978	41.80431449	1.075339541	0.005703	0.0333672	Ccser1	6	61180324	62382865	+	9635	protein_coding	coiled-coil serine rich 1 [Source:MGI Symbol;Acc:MGI:3045354]	-
ENSMUSG0000003041	1005.108585	932.1412915	940.6475709	704.568828	764.439849	678.1987273	959.2991493	715.7358015	0.422772302	0.0057142	0.0334222	Dmwd	7	19076227	19082776	+	2556	protein_coding	dystrophia myotonica-containing WD repeat motif [Source:MGI Symbol;Acc:MGI:94907]	-
ENSMUSG0000003004	21.04414785	25.27840791	1.117158635	62.01583816	62.22184818	65.19270406	15.81323813	63.14346347	-1.991729151	0.0057323	0.0335179	Gkn3	6	87383256	87388935	-	856	protein_coding	gastrokine 3 [Source:MGI Symbol;Acc:MGI:1916138]	-
ENSMUSG0000001980	1642.445635	1435.602916	1365.167852	846.6884571	1108.141487	1143.304885	1481.072134	1032.711609	0.520723334	0.0057408	0.033557	Pex3	10	13523842	13553142	-	4740	protein_coding	peroxisomal biogenesis factor 3 [Source:MGI Symbol;Acc:MGI:1929646]	-
ENSMUSG0000004195	691.4505722	513.9942941	628.9603116	869.0830654	870.1182261	853.3433054	611.4683927	864.1815323	-0.498907813	0.0057433	0.0335614	Pigs	11	78328415	78342782	+	2501	protein_coding	phosphatidylinositol glycan anchor biosynthesis, class S [Source:MGI Symbol;Acc:MGI:2687325]	-
ENSMUSG0000004877	241.5066491	208.5468652	166.4566366	351.4230829	273.5786023	361.9654614	205.5033837	328.9890489	-0.678120928	0.0057546	0.0336165	P2ry6	7	100937630	100974649	-	2887	protein_coding	pyrimidinergic receptor P2Y, G-protein coupled, 6 [Source:MGI Symbol;Acc:MGI:2673874]	-
ENSMUSG0000007929	306.6432973	134.8181755	130.7075603	282.5165961	392.0964083	515.7034799	190.723011	396.7721614	-1.055249884	0.0057569	0.0336195	Clec7a	6	129461591	129472779	-	2638	polymorphic_pseud	C-type lectin domain family 7, member a [Source:MGI Symbol;Acc:MGI:1861431]	-
ENSMUSG0000009854	7.01471595	20.01207293	14.52306226	0	2.962945151	0.973025434	13.84995038	1.311990195	3.433565952	0.005768	0.0336739	Mir7211	10	94586137	94586199	-	63	miRNA	microRNA 7211 [Source:MGI Symbol;Acc:MGI:5530918]	-
ENSMUSG0000000316	1296.720349	1121.729351	1410.971356	1642.55838	1754.06353	1811.773358	1276.473685	1736.131756	-0.443738646	0.0057736	0.0336959	Sri	5	8046078	8069379	+	4852	protein_coding	sorcin [Source:MGI Symbol;Acc:MGI:98419]	-
ENSMUSG0000004754	1568.290066	1367.140561	1702.54976	1168.826283	1117.030322	1145.250936	1545.993462	1143.702514	0.434656817	0.0057902	0.0337748	Cltb	13	54592401	54611344	-	4868	protein_coding	clathrin, light polypeptide (Lcb) [Source:MGI Symbol;Acc:MGI:1921575]	-
ENSMUSG0000001635	60.12613672	65.30255376	160.8708435	49.95720296	29.62945151	28.21773758	95.43317798	35.93479735	1.404152846	0.0057907	0.0337748	Col20a1	2	180986535	181018363	+	5142	protein_coding	collagen, type XX, alpha 1 [Source:MGI Symbol;Acc:MGI:1920618]	-
ENSMUSG0000003725	681.4295495	620.3742607	601.0313457	900.0909844	827.6493456	842.6400257	634.2783853	856.7934519	-0.433728761	0.0057954	0.0337916	Mex3c	18	73573044	73592575	+	3738	protein_coding	mex3 RNA binding family member C [Source:MGI Symbol;Acc:MGI:2652843]	-
ENSMUSG0000003333	2953.195415	2785.891205	2959.353225	2078.39191	2230.11005	2376.128109	2899.479948	2228.210023	0.380022912	0.0058241	0.0339483	Dnm2	9	21424908	21507759	+	5330	protein_coding	dynamin 2 [Source:MGI Symbol;Acc:MGI:109547]	-
ENSMUSG0000008509	94.19761419	108.4865006	113.9501808	24.1172704	60.24655141	58.38152603	105.5447652	47.58178261	1.153572594	0.0058311	0.0339785	Gm15635	7	99660130	99667500	+	2870	antisense	predicted gene 15635 [Source:MGI Symbol;Acc:MGI:3783079]	-
ENSMUSG0000002119	1164.442848	1179.659036	1577.427993	1587.433191	2131.345212	2118.276369	1307.176625	1945.684924	-0.573869565	0.0058329	0.0339787	Pfkp	13	6579768	6648777	-	13401	protein_coding	phosphofructokinase, platelet [Source:MGI Symbol;Acc:MGI:1891833]	-
ENSMUSG0000004126	138.2901144	123.2322385	168.6909539	99.05307484	65.18479335	72.97690753	143.4044356	79.0715919	0.855585998	0.0058547	0.0340953	Rusc1	3	89083981	89093311	-	5598	protein_coding	RUN and SH3 domain containing 1 [Source:MGI Symbol;Acc:MGI:1919546]	-
ENSMUSG0000002388	2078.360126	1153.327361	1110.455683	2866.509853	2153.073477	2278.825566	1447.381057	2432.802965	-0.749024252	0.0058609	0.0341204	Smoc2	17	14279506	14404790	+	6951	protein_coding	SPARC related modular calcium binding 2 [Source:MGI Symbol;Acc:MGI:1929881]	-
ENSMUSG0000003173	185.3889215	106.3799666	262.5322793	90.43976399	97.77718999	87.57228904	184.7670558	91.92974767	1.006510442	0.005867	0.0341452	Irx3	8	91798525	91802067	-	2762	protein_coding	Iroquois related homeobox 3 [Source:MGI Symbol;Acc:MGI:1197522]	Hom
ENSMUSG0000004420	0	0	0	10.33597303	10.86413222	1.946050868	0	7.715385372	-5.312311055	0.0058794	0.0342067	Cdc25c	18	34732997	34751533	-	1941	protein_coding	cell division cycle 25C [Source:MGI Symbol;Acc:MGI:88350]	-
ENSMUSG0000002803	131.2753985	37.91761186	96.07564263	10.33597303	43.45652888	28.21773758	88.42288433	27.3367465	1.698831777	0.0058878	0.0342451	Ptgfr	3	151796502	151837630	-	6778	protein_coding	prostaglandin F receptor [Source:MGI Symbol;Acc:MGI:97796]	-
ENSMUSG0000006404	238.5003423	155.8835154	203.3228716	315.2471773	319.0104279	304.5569608	199.2355764	312.9381887	-0.650908274	0.0058905	0.0342483	Trerf1	17	47140875	47361958	+	9070	processed_transcrip	transcriptional regulating factor 1 [Source:MGI Symbol;Acc:MGI:2442086]	zf-C2
ENSMUSG0000002489	153.3216486	116.9126366	103.8957531	171.404886	236.0479637	232.5530787	124.7100128	213.3353095	-0.77210064	0.005892	0.0342483	Apba1	19	23758876	23949597	+	6632	protein_coding	amyloid beta (A4) precursor protein binding, family A, member 1 [Source:MGI Symbol;Acc:MGI:1860297]	-
ENSMUSG0000002602	1400.938986	1322.903347	1896.935363	885.448356	1113.079728	1107.302944	1540.259232	1035.277009	0.573217534	0.0058992	0.0342792	Casp8	1	58795374	58847503	+	2893	protein_coding	caspase 8 [Source:MGI Symbol;Acc:MGI:1261423]	-
ENSMUSG0000003562	515.0805712	594.0425858	426.7545987	708.8754834	815.797565	712.2546175	511.9592519	745.6425553	-0.541932362	0.0059029	0.0342905	Ric8b	10	84917616	85018337	+	5272	protein_coding	RIC8 guanine nucleotide exchange factor B [Source:MGI Symbol;Acc:MGI:2682307]	-
ENSMUSG0000002977	1440.020974	1583.060295	1715.955664	2050.829315	2062.209825	2149.413183	1579.678978	2087.484108	-0.40230407	0.0059069	0.0343029	Gars	6	55038007	55079500	+	3119	protein_coding	glycyl-tRNA synthetase [Source:MGI Symbol;Acc:MGI:2449057]	-
ENSMUSG0000004300	211.4435808	222.2393362	163.1051607	354.0070762	241.973854	423.2660637	198.9293592	339.748998	-0.771828105	0.0059166	0.0343423	Gng2	14	19872559	19977627	-	4390	protein_coding	guanine nucleotide binding protein (G protein), gamma 2 [Source:MGI Symbol;Acc:MGI:102705]	-

ENSMUSG0000000639	587.2319353	536.112901	732.8560647	1008.618701	866.1676325	828.0446441	618.7336337	900.9436593	-0.542780833	0.0059174	0.0343423	Elovl1	4	118428093	118432953	+	2726	protein_coding	elongation of very long chain fatty acids (FEN1/Elo2, SUR4/Elo3, yeast)-like 1 [Source:MGI Symbol;Acc:MGI:1858959]	-
ENSMUSG0000002141	2782.838028	3317.791038	3715.669621	1962.973544	2607.391733	2282.717668	3272.099562	2284.360982	0.518478075	0.0059239	0.0343681	Prpf4b	13	34875302	34906064	+	8430	protein_coding	pre-mRNA processing factor 4B [Source:MGI Symbol;Acc:MGI:109584]	-
ENSMUSG0000003260	1440.020974	1738.943811	1233.343133	958.6614983	1039.993748	1090.761511	1470.769306	1029.805586	0.514464448	0.0059255	0.0343681	Slc25a20	9	108662088	108684641	+	4844	protein_coding	solute carrier family 25 (mitochondrial carnitine/acylcarnitine translocase), member 20 [Source:MGI Symbol;Acc:MGI:1928738]	-
ENSMUSG0000002742	567.1898897	667.7712755	701.5756229	913.0109507	843.4517197	914.6439078	645.5122627	890.3688594	-0.464475765	0.005947	0.0344825	Sec23b	2	144556229	144590749	+	6302	protein_coding	SEC23 homolog B, COPII coat complex component [Source:MGI Symbol;Acc:MGI:1350925]	-
ENSMUSG0000009710	22.04625013	14.74573795	17.87453816	47.37320971	41.48123212	45.73219539	18.22217541	44.8622124	-1.298578679	0.005951	0.0344926	1810034E14Ri	13	64248700	64271852	+	2245	antisense	RIKEN cDNA 1810034E14 gene [Source:MGI Symbol;Acc:MGI:1913753]	-
ENSMUSG0000006421	3415.164566	2684.777573	3873.188988	4282.538158	5306.634766	4771.716727	3324.377042	4786.963217	-0.526025914	0.0059525	0.0344926	Ifi27	12	103434211	103440239	+	1467	protein_coding	interferon, alpha-inducible protein 27 [Source:MGI Symbol;Acc:MGI:1277180]	-
ENSMUSG0000008678	2297.820525	2133.918934	1944.973184	1392.772365	1594.064491	1702.794509	2125.570881	1563.210455	0.443658206	0.0059602	0.0345265	Isoc2a	7	4877153	4896973	+	2244	protein_coding	isochorismatase domain containing 2a [Source:MGI Symbol;Acc:MGI:3609243]	-
ENSMUSG0000004044	1581.317396	1296.571672	949.5848399	993.9760728	676.5391429	449.5377504	1275.824636	706.684322	0.851996649	0.0059657	0.0345479	Spns2	11	72451638	72489904	-	4083	protein_coding	spinster homolog 2 [Source:MGI Symbol;Acc:MGI:2384936]	-
ENSMUSG0000009939	30.06306836	65.30255376	42.45202814	109.3890479	83.95011262	99.24859425	45.93921675	97.52925158	-1.087980812	0.0059878	0.0346653	Ms4a14	19	11301249	11314454	-	4065	protein_coding	membrane-spanning 4-domains, subfamily A, member 14 [Source:MGI Symbol;Acc:MGI:2686122]	-
ENSMUSG0000002699	175.3678988	160.0965834	227.9003616	284.2392582	301.232757	288.0155284	187.7882813	291.1625146	-0.633676477	0.0059991	0.0347112	Nup35	2	80617236	80658906	+	3366	protein_coding	nucleoporin 35 [Source:MGI Symbol;Acc:MGI:1916732]	-
ENSMUSG0000004527	19.03994329	26.3316749	11.17158635	180.879528	34.56769343	24.32563584	18.84773485	79.92428575	-2.08499164	0.0059995	0.0347112	Lca5l	16	96158407	96192271	-	6583	protein_coding	Leber congenital amaurosis 5-like [Source:MGI Symbol;Acc:MGI:3041157]	-
ENSMUSG0000002236	18.03784102	0	16.75737953	50.81853405	60.24655141	52.54337342	11.59840685	54.53615296	-2.233059162	0.0060382	0.0349244	Has2	15	56665627	56694539	-	4237	protein_coding	hyaluronan synthase 2 [Source:MGI Symbol;Acc:MGI:107821]	-
ENSMUSG0000002896	888.8647211	828.9211259	707.1614161	1031.874641	1171.350983	1151.089088	808.3157544	1118.104904	-0.467502458	0.006047	0.0349648	Slc4a2	5	24423837	24440950	+	8322	protein_coding	solute carrier family 4 (anion exchanger), member 2 [Source:MGI Symbol;Acc:MGI:109351]	-
ENSMUSG0000002244	3.006306836	1.053266996	0	29.28525691	8.888835454	7.78420347	1.353191277	15.31943194	-3.476215962	0.006054	0.0349943	Adamts20	15	94270163	94465418	-	8997	protein_coding	a disintegrin-like and metallopeptidase (reprolysin type) with thrombospondin type 1 motif, 20 [Source:MGI Symbol;Acc:MGI:266	-
ENSMUSG0000007049	283.5949448	151.6704474	515.0101308	112.8343722	166.9125769	151.7919677	316.7585077	143.8463056	1.138997225	0.0060733	0.0350948	Ctcfl	2	173093609	173119525	-	3217	protein_coding	CCCTC-binding factor (zinc finger protein)-like [Source:MGI Symbol;Acc:MGI:3652571]	zf-C2
ENSMUSG0000003028	1322.775008	1659.948786	1346.176155	845.8271261	1175.301577	947.7267725	1442.96665	989.6184917	0.544417573	0.0060784	0.0351137	Creld1	6	113483297	113493343	+	4251	protein_coding	cysteine-rich with EGF-like domains 1 [Source:MGI Symbol;Acc:MGI:2152539]	-
ENSMUSG0000002281	1467.077736	1624.137708	1084.761035	1743.334117	2441.466805	2145.521081	1391.99216	2110.107334	-0.599853227	0.0060969	0.0352093	Gsk3b	16	38089001	38246084	+	9831	protein_coding	glycogen synthase kinase 3 beta [Source:MGI Symbol;Acc:MGI:1861437]	-
ENSMUSG0000005172	9.018920508	16.85227194	1.117158635	151.5942711	18.76531929	13.62235607	8.996117027	61.32731548	-2.767380855	0.0061075	0.0352559	Kctd14	7	97451323	97459557	+	2568	protein_coding	potassium channel tetramerisation domain containing 14 [Source:MGI Symbol;Acc:MGI:1289222]	-
ENSMUSG0000007886	530.1121054	520.3138961	783.1282033	810.5125515	964.9324709	1058.651672	611.1847349	944.6988981	-0.628535619	0.0061088	0.0352559	Zfp970	2	177464733	177478129	+	2172	protein_coding	zinc finger protein 970 [Source:MGI Symbol;Acc:MGI:3652255]	zf-C2
ENSMUSG0000004509	916.9235849	1211.257046	706.0442575	645.1369831	621.2308334	544.8942429	944.7416293	603.7540198	0.646002001	0.0061106	0.0352559	Magi1	6	93675455	94283917	-	10579	protein_coding	membrane associated guanylate kinase, WW and PDZ domain containing 1 [Source:MGI Symbol;Acc:MGI:1203522]	-
ENSMUSG0000002897	336.7063656	269.636351	421.1688055	192.0768321	234.0726669	216.9846717	342.5038407	214.3780569	0.676115207	0.0061156	0.0352681	Casz1	4	148804429	148954889	+	12224	protein_coding	castor zinc finger 1 [Source:MGI Symbol;Acc:MGI:1196251]	-
ENSMUSG0000004967	79.16608001	70.56888874	250.2435343	44.78921645	58.27125464	46.70522082	133.3261677	49.9218973	1.416411659	0.0061174	0.0352681	Catsperg1	7	29181321	29214035	-	7044	protein_coding	cation channel sperm associated auxiliary subunit gamma 1 [Source:MGI Symbol;Acc:MGI:2443617]	-
ENSMUSG0000002096	3257.834508	2320.347192	1855.600493	2947.474975	4626.145029	4791.177236	2477.927398	4121.59908	-0.733873956	0.0061183	0.0352681	Tshr	12	91384563	91549808	+	6012	protein_coding	thyroid stimulating hormone receptor [Source:MGI Symbol;Acc:MGI:98849]	-
ENSMUSG0000002175	653.3706857	602.4687218	730.6217474	883.7256938	959.0065806	874.749865	662.1537183	905.8273798	-0.452236571	0.0061431	0.0353976	Map3k1	13	111746428	111808993	-	7447	protein_coding	mitogen-activated protein kinase kinase kinase 1 [Source:MGI Symbol;Acc:MGI:1346872]	-
ENSMUSG0000006801	28.0588638	21.06533992	54.74077313	9.474641942	3.950593535	13.62235607	34.62165895	9.01586385	1.937277104	0.0061446	0.0353976	Gm14648	X	47900345	47900827	+	483	processed_pseudog	predicted gene 14648 [Source:MGI Symbol;Acc:MGI:3705508]	-
ENSMUSG0000005467	2778.829619	1664.161854	3664.280324	1521.110697	1589.126249	1717.389891	2702.423932	1609.208946	0.747864187	0.0061568	0.0354568	1600014C10Ri	7	38183217	38197568	+	3949	protein_coding	RIKEN cDNA 1600014C10 gene [Source:MGI Symbol;Acc:MGI:1919494]	-
ENSMUSG0000007661	1.002102279	0	0	8.613310856	18.76531929	2.919076301	0.334034093	10.09923548	-4.736661366	0.0061612	0.0354686	Ighg3	12	113356224	113361232	-	2512	IG_C_gene	Immunoglobulin heavy constant gamma 3 [Source:MGI Symbol;Acc:MGI:2144790]	-
ENSMUSG0000011694	147.309035	456.0646093	151.9335744	108.5277168	96.78954161	118.7091029	251.7690729	108.0087871	1.220943264	0.0061626	0.0354686	AC130815.2	16	31233590	31248313	+	4634	lincRNA	novel transcript	-
ENSMUSG0000003028	2.004204557	3.159800988	1.117158635	14.64262846	7.90118707	23.35261041	2.093721394	15.29880865	-2.863338027	0.0061699	0.0354997	St8sia1	6	142821545	142964452	-	9341	protein_coding	ST8 alpha-N-acetyl-neuraminide alpha-2,8-sialyltransferase 1 [Source:MGI Symbol;Acc:MGI:106011]	-
ENSMUSG0000005152	7.01471595	5.26633498	26.81180725	1.722662171	0	0.973025434	13.03095273	0.898562535	3.821683433	0.0061776	0.0355225	Usp29	7	6730578	6967219	+	18078	protein_coding	ubiquitin specific peptidase 29 [Source:MGI Symbol;Acc:MGI:1888998]	-
ENSMUSG0000002005	11.02312506	18.95880593	10.05442772	24.97860148	45.43182565	51.57034799	13.3454529	40.66025837	-1.603991825	0.0061777	0.0355225	Ascl1	10	87490819	87493660	-	2481	protein_coding	achaete-scute family bHLH transcription factor 1 [Source:MGI Symbol;Acc:MGI:96919]	bHLH
ENSMUSG0000003450	330.6937519	286.4886229	303.8671488	429.8042117	468.1453339	410.6167331	307.0165079	436.1887596	-0.506225574	0.0061828	0.0355411	Pcnx4	12	72536383	72580119	+	4300	protein_coding	pecanex homolog 4 [Source:MGI Symbol;Acc:MGI:1914958]	-
ENSMUSG0000002452	98.2060233	169.5759864	165.339478	81.82645313	63.20949656	83.6801873	144.3738292	76.23871233	0.919320137	0.0061924	0.0355852	Gnal	18	67088336	67226792	+	5761	protein_coding	guanine nucleotide binding protein, alpha stimulating, olfactory type [Source:MGI Symbol;Acc:MGI:95774]	-
ENSMUSG0000003843	1701.569669	1970.66255	1726.010091	1361.764446	1431.102508	1188.064055	1799.414103	1326.977003	0.439346242	0.0062099	0.035675	Mllt6	11	97663414	97685463	+	7451	protein_coding	myeloid/lymphoid or mixed-lineage leukemia; translocated to, 6 [Source:MGI Symbol;Acc:MGI:1935145]	-
ENSMUSG0000004127	12.02522734	7.372868973	1.117158635	62.87716925	13.82707737	31.13681388	6.838418317	35.94702017	-2.386222564	0.0062175	0.0357077	Tox	4	6686353	6991557	-	7609	protein_coding	thymocyte selection-associated high mobility group box [Source:MGI Symbol;Acc:MGI:2181659]	HMG
ENSMUSG0000003426	503.0553439	473.9701482	428.9889159	654.611625	603.4531625	692.7941089	468.6714693	650.2862988	-0.472131663	0.0062204	0.0357131	Ints14	9	64960905	64986978	+	3277	protein_coding	integrator complex subunit 14 [Source:MGI Symbol;Acc:MGI:1917132]	-
ENSMUSG0000005660	1005.108585	1213.36358	837.8689764	1339.369838	1632.582778	1471.214456	1018.78038	1481.055691	-0.539462905	0.0062281	0.0357465	Chd9	8	90828352	91054516	+	23148	protein_coding	chromodomain helicase DNA binding protein 9 [Source:MGI Symbol;Acc:MGI:1924001]	-
ENSMUSG0000008703	7.01471595	20.01207293	13.40590362	0.861331086	0.987648384	2.919076301	13.47756417	1.589351924	3.094954213	0.0062472	0.0358453	Gm16143	10	63055603	63057467	-	538	antisense	predicted gene 16143 [Source:MGI Symbol;Acc:MGI:3801780]	-
ENSMUSG0000002357	1.002102279	0	0	48.23454079	3.950593535	0	0.334034093	17.39504478	-5.525163807	0.0062522	0.0358628	Iqcf3	9	106503225	106561631	-	2677	protein_coding	IQ motif containing F3 [Source:MGI Symbol;Acc:MGI:1915515]	-
ENSMUSG0000003906	316.66432	319.1398998	404.4114259	239.4500418	245.9244476	182.9287816	346.7385486	222.767757	0.637392093	0.0062598	0.0358953	Mtg2	2	180070588	180085902	+	5949	protein_coding	mitochondrial ribosome associated GTPase 2 [Source:MGI Symbol;Acc:MGI:106565]	-
ENSMUSG0000005718	8.016818229	14.74573795	8.937269082	58.57051382	22.71591283	28.21773758	10.56660842	36.50138808	-1.792342576	0.0062638	0.0359003	Scn3a	2	65457118	65567627	-	12459	protein_coding	sodium channel, voltage-gated, type III, alpha [Source:MGI Symbol;Acc:MGI:98249]	-
ENSMUSG0000002793	1036.173756	1485.106465	1121.62727	821.7098557	885.9206002	808.5841355	1214.302497	838.7381971	0.533861025	0.0062645	0.0359003	Ints3	3	90391388	90433622	-	5677	protein_coding	integrator complex subunit 3 [Source:MGI Symbol;Acc:MGI:2140050]	-
ENSMUSG0000003223	715.5010269	787.8437131	733.9732233	591.7344558	478.0218177	542.948192	745.7726545	537.5681552	0.471721943	0.0062724	0.035935	Rp9	9	22411047	22469743	-	3570	protein_coding	retinitis pigmentosa 9 (human) [Source:MGI Symbol;Acc:MGI:2157166]	-
ENSMUSG0000001738	207.4351717	320.1931668	175.3939057	439.2788537	428.6393985	332.7746984	234.3407481	400.2309835	-0.7720819	0.0062791	0.0359623	Traf4	11	78158499	78165589	-	3292	protein_coding	TNF receptor associated factor 4 [Source:MGI Symbol;Acc:MGI:1202880]	-
ENSMUSG0000003727	533.1184122	529.793299	483.7296891	351.4230829	368.3928471	395.0483261	515.5471334	371.6214187	0.472873377	0.0062817	0.035966	Gemin5	11	58120002	58168539	-	6618	protein_coding	gem nuclear organelle associated protein 5 [Source:MGI Symbol;Acc:MGI:2449311]	-
ENSMUSG0000003975	1051.20529	966.8991024	1160.727822	808.7898894	729.8721556	801.7729574	1059.610738	780.1450008	0.441397968	0.0062883	0.0359821	Dnttip2	3	122274388	122285271	+	3309	protein_coding	deoxynucleotidyltransferase, terminal, interacting protein 2 [Source:MGI Symbol;Acc:MGI:1923173]	-
ENSMUSG0000002551	5024.540825	3490.526825	5127.758136	6247.234364	6355.517349	7128.384328	4547.608595	6577.045347	-0.532328781	0.0062884	0.0359821	Cd151	7	141467392	141471473	+	2741	protein_coding	CD151 antigen [Source:MGI Symbol;Acc:MGI:1096360]	-
ENSMUSG0000003910	106.2228415	124.2855055	154.1678917	212.7487781	237.0356121	183.901807	128.2254129	211.2287324	-0.721638799	0.0062991	0.0360326	Nexn	3	152236982	152266350	-	3826	protein_coding	nexilin [Source:MGI Symbol;Acc:MGI:1916060]	-
ENSMUSG0000004569	591.2403444	490.8224202	482.6125304	382.431002	349.6275278	369.7496648	521.5584317	367.2693982	0.506148453	0.0063034	0.036046	Wdr89	12	75630596	75669537	-	3192	protein_coding	WD repeat domain 89 [Source:MGI Symbol;Acc:MGI:1919588]	-
ENSMUSG0000000103	285.5991494	238.0383411	313.9215765	435.8335293	408.8864309	385.3180718	279.1863557	410.0126773	-0.555106188	0.0063118	0.0360834	Mapk7	11	61488812	61494406	-	4394	protein_coding	mitogen-activated protein kinase 7 [Source:MGI Symbol;Acc:MGI:1346347]	-
ENSMUSG0000000239	1573.300577	1381.886299	1160.727822	1103.365121	771.3533877	892.2643228	1371.971566	922.3276104	0.572692888	0.0063152	0.0360918	Use1	8	71366848	71369732	+	2203	protein_coding	unconventional SNARE in the ER 1 homolog (S. cerevisiae) [Source:MGI Symbol;Acc:MGI:1914273]	-
ENSMUSG0000000299	8655.15738	5266.33498	7617.904734	9258.447839	10655.73841	14108.86879	7179.799031	11341.01835	-0.659504812	0.0063248	0.0361266	Prkar2b	12	31958476	32061296	-	3652	protein_coding	protein kinase, cAMP dependent regulatory, type II beta [Source:MGI Symbol;Acc:MGI:97760]	-
ENSMUSG0000011132	43.09039798	43.18394684	35.74907633	13.78129737	12.83942899	20.43353411	40.67447372	15.68475349	1.377234766	0.0063253	0.0361266	A830035O19R	9	45954101	45955998	+	1898	antisense	RIKEN cDNA A830035O19 gene [Source:MGI Symbol;Acc:MGI:3704334]	-
ENSMUSG0000010673	377.792559	479.2364832	446.8634541	576.2304963	616.2925915	655.8191424	434.6308321	616.1140767	-0.503549198	0.0063271	0.0361266	Gm20559	6	3333194	3346128	-	4690	processed_transcrip	predicted gene, 20559 [Source:MGI Symbol;Acc:MGI:5295666]	-
ENSMUSG0000006720	178.3742056	249.6242781	215.6116166	157.6235887	112.5919157	108.0058231	214.5367001	126.0737759	0.764637287	0.0063405	0.0361919	H2-K2	17	33974831	33978790	-	1049	unprocessed_pseud	histocompatibility 2, K region locus 2 [Source:MGI Symbol;Acc:MGI:95906]	-
ENSMUSG0000007855	2.004204557	2.106533992	1.117158635	41.34389211	4.938241919	6.811178036	1.742632395	17.69777069	-3.343477728	0.0063452	0.0362075	Fam229a	4	129491190	129491956	+	637	protein_coding	family with sequence similarity 229, member A [Source:MGI Symbol;Acc:MGI:1915483]	-
ENSMUSG0000002801	262.550797	208.5468652	317.2730524	187.7701767	128.3942899	152.7649931	262.7902382	156.3098199	0.747641558	0.0063537	0.036245	Gar1	3	129824912	129831396	-	1714	protein_coding	GAR1 ribonucleoprotein [Source:MGI Symbol;Acc:MGI:1930948]	-
ENSMUSG0000002864	575.2067079	468.7038133	529.5331931	855.301768	703.2056492	686.9559562	524.4812381	748.4877912	-0.51336416	0.0063762	0.0363565	P3h1	4	119232915	119248975	+	3846	protein_coding	prolyl 3-hydroxylase 1 [Source:MGI Symbol;Acc:MGI:1888921]	-
ENSMUSG0000002860	1912.011148	1955.916812	1327.184459	2110.26116	2927.389809	2881.128309	1731.704139	2639.593093	-0.607844161	0.0063771	0.0363565	Cpt2	4	107903981	107923610	-	2391	protein_coding	carnitine palmitoyltransferase 2 [Source:MGI Symbol;Acc:MGI:109176]	-
ENSMUSG0000002072	11.02312506	14.74573795	2.23431727	0	0.987648384	0	9.334393427	0.329216128	4.796655617	0.006389	0.0364135	Cacng1	11	107703218	107716522	-	1249	protein_coding	calcium channel, voltage-dependent, gamma subunit 1 [Source:MGI Symbol;Acc:MGI:1206582]	-
ENSMUSG0000009934	10.02102279	0	39.10055223	228.2527377	200.4926219	269.5280452	16.37385834	232.7578016	-3.831554292	0.0064	0.0364649	1700047G03Ri	15	11964276	11970086	+	1493	lincRNA	RIKEN cDNA 1700047G03 gene [Source:MGI Symbol;Acc:MGI:1920589]	-
ENSMUSG0000004641	16.03363646	36.86434486	12.28874499	54.26385839	51.35771595	73.94993297	21.72890877	59.8571691	-1.460179911	0.0064024	0.0364676	B430212C06Ri	18	67321209	67344103	-	3720	processed_transcrip	RIKEN cDNA B430212C06 gene [Source:MGI Symbol;Acc:MGI:2442134]	-
ENSMUSG0000003818	572.2004011	661.4516736	518.3616067	421.1909009	399.9975954	419.373962	584.0045605	413.5208194	0.498108406	0.0064085	0.0364802	Scarf1	11	75513540	75526582	+	2887	protein_coding	scavenger receptor class F, member 1 [Source:MGI Symbol;Acc:MGI:2449455]	-
ENSMUSG0000002898	2.004204557	4.213067984	3.351475906	79.24245987	7.90118707	2.919076301	3.189582816	30.02090775	-3.241005423	0.0064136	0.0365087	Angptl7	4	148495183	148500460	-	2157	protein_coding	angiopoietin-like 7 [Source:MGI Symbol;Acc:MGI:3605801]	-
ENSMUSG0000002240	916.9235849	851.0397328	681.4667675	1141.263688	1151.598015	1127.736478	816.4766951	1140.199394	-0.481365174	0.006417	0.0365174	Slc25a17	15	81318911	81360812	-	2847	protein_coding	solute carrier family 25 (mitochondrial carrier, peroxisomal membrane protein), member 17 [Source:MGI Symbol;Acc:MGI:13422	-
ENSMUSG0000003602	490.0280142	441.3188714	540.7047794	371.2336979	337.7757472	323.044444	490.6838884	344.017963	0.511556232	0.0064199	0.0365224	Parp2	14	50807841	50821301	+	3628	protein_coding	poly (ADP-ribose) polymerase family, member 2 [Source:MGI Symbol;Acc:MGI:1341112]	-
ENSMUSG0000003138	1820.81984	1688.386995	1650.043304	2048.245322	2368.380824	2459.808297	1719.750046	2292.144814	-0.414248946	0.0064245	0.0365375	Hcfc1	X	73942792	73966357	-	8360	protein_coding	host cell factor C1 [Source:MGI Symbol;Acc:MGI:105942]	-
ENSMUSG0000011389	31.06517064	53.7166168	155.2850503	16.36529063	26.66650636	30.16378845	80.02227924	24.39852848	1.714132194	0.0064331	0.0365756	Gm19144	12	69970386	69972795	+	1859	processed_pseudog	predicted gene, 19144 [Source:MGI Symbol;Acc:MGI:5011329]	-
ENSMUSG0000002941	11.02312506	6.319601977	2.23431727	35.31457451	17.77767091	30.16378845	6.525681437	27.75201129	-2.078496339	0.0064599	0.0367154	Kntc1	5	123749716	123821593	+	7983	protein_coding	kinetochore associated 1 [Source:MGI Symbol;Acc:MGI:2673709]	-
ENSMUSG0000003041	491.0301165	414.9871965	503.8385445	360.0363938	316.0474828	298.7188082	469.9519525	324.9342182	0.531621565	0.0064616	0.0367154	Pdcd5	7	35641982	35647521	-	2486	protein_coding	programmed cell death 5 [Source:MGI Symbol;Acc:MGI:1913538]	-
ENSMUSG0000005506	9536.005283	10110.3099	10471.12789	7085.30951	8449.331923	7500.080044	10039.14769	7678.240492	0.386814942	0.0064693	0.0367476	Ddx17	15	79527736	79546741	-	7643	protein_coding	DEAD (Asp-Glu-Ala-Asp) box polypeptide 17 [Source:MGI Symbol;Acc:MGI:1914290]	-
ENSMUSG0000011597	11.02312506	24.22514091	37.9833936	6.029317599	4.938241919	5.838152603	24.41055319	5.60190404	2.118376872	0.0064746	0.0367572	8430426J06Rik	15	81242657	81312866	-	1712	lincRNA	RIKEN cDNA 8430426J06 gene [Source:MGI Symbol;Acc:MGI:1925352]	-
ENSMUSG0000002052	420.882957	424.4665994	456.9178818	320.4151638	311.1092409	304.5569608	434.0891461	312.0271218	0.475857715	0.0064749	0.0367572	Ppm1d	11	85311244	85347066	+	3173	protein_coding	protein phosphatase 1D magnesium-dependent, delta isoform [Source:MGI Symbol;Acc:MGI:1858214]	-
ENSMUSG0000005623	3881.142125	3290.406096	2538.184419	1462.540183	2303.196031	2340.126168	3236.577547	2035.287461	0.669498573	0.0064858	0.0368081	Ncoa4	14	32159865	32179855	+	9365	protein_coding	nuclear receptor coactivator 4 [Source:MGI Symbol;Acc:MGI:1350932]	-
ENSMUSG0000003393	477.0006846	531.899833	567.5165867	836.3524841	690.3662202	692.7941089	525.4723681	739.8376044	-0.494399234	0.0064894	0.0368171	Vhl	6	113623959	113631633	+	2997	protein_coding	von Hippel-Lindau tumor suppressor [Source:MGI Symbol;Acc:MGI:103223]	-
ENSMUSG0000003377	438.920798	517.1540951	483.7296891	295.4365624	357.5287149	351.2621816	479.9348607	334.7424863	0.520362885	0.0064955	0.0368303	Rpap2	5	107597373	107661838	+	4136	protein_coding	RNA polymerase II associated protein 2 [Source:MGI Symbol;Acc:MGI:2141142]	-
ENSMUSG0000003652	9.018920508	21.06533992	11.17158635	49.09587188	25.67885798	52.54337342	13.75194893	42.43936776	-1.627657005	0.0064956	0.0368303	Card11	5	140872990	141000582	-	4866	protein_coding	caspase recruitment domain family, member 11 [Source:MGI Symbol;Acc:MGI:1916978]	-
ENSMUSG0000004011	756.5872204	710.9552224	630.0774703	534.0252731	484.9353564	503.0541493	699.2066377	507.3382596	0.462825198	0.0065282	0.0370035	Mrps35	6	147042764	147073991	+	8631	protein_coding	mitochondrial ribosomal protein S35 [Source:MGI Symbol;Acc:MGI:2385255]	-
ENSMUSG0000008085	21.04414785	26.3316749	44.68634541	9.474641942	9.876483837	8.757228904	30.68738939	9.369451561	1.708706246	0.0065306	0.0370062	Rpl10-ps1	2	105554201	105554845	-	645	transcribed_process	ribosomal protein L10, pseudogene 1 [Source:MGI Symbol;Acc:MGI:3650909]	-
ENSMUSG0000002278	247.5192628	229.6122051	230.1346789	180.0181969	112.5919157	139.142637	235.7553823	143.9175832	0.710138209	0.0065568	0.0371434	Wdr53	16	32247227	32257083	+	1523	protein_coding	WD repeat domain 53 [Source:MGI Symbol;Acc:MGI:1916230]	-
ENSMUSG0000003053	209.4393762	290.7016909	296.0470383	386.7376574	440.4911792	384.3450463	265.3960352	403.857961	-0.606393892	0.0065704	0.0372006	Sema4b	7	80186841	80226527	+	5645	protein_coding	sema domain, immunoglobulin domain (Ig), transmembrane domain (TM) and short cytoplasmic domain, (semaphorin) 4B [Source	-
ENSMUSG0000003995	3879.137921	3826.518997	2407.476859	4598.646666	5215.771115	5479.106218	3371.044592	5097.841333	-0.596553824	0.0065709	0.0372006	Dag1	9	108204634	108263958	-	6357	protein_coding	dystroglycan 1 [Source:MGI Symbol;Acc:MGI:101864]	-
ENSMUSG0000009386	0	1.053266996	0	10.33597303	7.90118707	7.78420347	0.351088999	8.673787856	-4.519505162	0.0065835	0.037261	Igkv1-110	6	68270485	68271267	+	401	IG_V_gene	immunoglobulin kappa variable 1-110 [Source:MGI Symbol;Acc:MGI:4439558]	-
ENSMUSG0000002037	9217.336759	10642.20973	8681.439754	7965.58988	6371.319724	6426.83299	9513.662081	6921.247531	0.458917456	0.0065926	0.0373011	Rack1	11	48800332	48806434	+	5119	protein_coding	receptor for activated C kinase 1 [Source:MGI Symbol;Acc:MGI:101849]	-
ENSMUSG0000001531	6.012613672	6.319601977	3.351475906	20.67194605	24.69120959	18.48748324	5.227897185	21.2835463	-2.019236496	0.0066014	0.0373392	Slamf6	1	171917515	171953170	+	10832	protein_coding	SLAM family member 6 [Source:MGI Symbol;Acc:MGI:1353620]	-
ENSMUSG0000006308	14.0294319	17.90553893	14.52306226	37.03723668	43.45652888	37.94799192	15.48601103	39.48058583	-1.349675042	0.0066166	0.0374088	Gm10125	18	5491503	5593505	-	7466	processed_transcrip	predicted gene 10125 [Source:MGI Symbol;Acc:MGI:3642044]	-
ENSMUSG0000003222	9.018920508	8.426135969	2.23431727	105.9437235	7.90118707	15.56840694	6.559791249	43.13777251	-2.714575106	0.0066177	0.0374088	Mns1	9	72438011	72462025	+	5363	protein_coding	meiosis-specific nuclear structural protein 1 [Source:MGI Symbol;Acc:MGI:107933]	-
ENSMUSG0000000282	140.294319	152.7237144	160.8708435	101.6370681	94.81424484	68.11178036	151.2962923	88.18769777	0.776853721	0.0066215	0.0374193	Qtrt1	9	21411837	21420274	+	1973	protein_coding	queuine tRNA-ribosyltransferase catalytic subunit 1 [Source:MGI Symbol;Acc:MGI:1931441]	-
ENSMUSG0000003594	760.5956295	636.1732656	557.462159	460.8121308	465.1823887	435.9153943	651.4103514	453.9699713	0.521415016	0.0066277	0.0374431	Ttc38	15	85832306	85858822	+	3842	protein_coding	tetratricopeptide repeat domain 38 [Source:MGI Symbol;Acc:MGI:2146198]	-
ENSMUSG0000002986	2082.368535	1517.757741	1353.996266	2521.116088	2557.021666	2217.524964	1651.374181	2431.887572	-0.558195002	0.0066422	0.0375059	Zyx	6	42349630	42360213	+	5390	protein_coding	zyxin [Source:MGI Symbol;Acc:MGI:103072]	-
ENSMUSG0000003300	6.012613672	2.106533992	2.23431727	25.83993257	10.86413222	20.43353411	3.451154978	19.0458663	-2.45515175	0.0066444	0.0375059	Sox10	15	79154908	79165240	-	3051	protein_coding	SRY (sex determining region Y)-box 10 [Source:MGI Symbol;Acc:MGI:98358]	HMG
ENSMUSG0000007340	25.05255697	9.479402965	6.702951811	2.583993257	0.987648384	0.973025434	13.74497058	1.514889025	3.163954487	0.0066449	0.0375059	Trim10	17	36869574	36877833	+	2245	protein_coding	tripartite motif-containing 10 [Source:MGI Symbol;Acc:MGI:1338757]	-
ENSMUSG0000003821	151.3174441	195.9076613	184.3311748	298.8818867	262.7144701	252.0135873	177.1854267	271.2033147	-0.615419717	0.0066506	0.0375267	Bend3	10	43478831	43515396	+	6819	protein_coding	BEN domain containing 3 [Source:MGI Symbol;Acc:MGI:2677212]	-
ENSMUSG0000002685	660.3854016	599.3089208	914.9529222	512.4919959	428.6393985	487.4857423	724.8824149	476.2057123	0.605486356	0.0066576	0.0375553	BC005624	2	30972177	30982201	-	3280	protein_coding	cDNA sequence BC005624 [Source:MGI Symbol;Acc:MGI:2385132]	-
ENSMUSG0000001898	28.0588638	43.18394684	72.61531129	89.5784329	140.2460705	95.35649251	47.95270731	108.3936653	-1.179585093	0.0066852	0.0376992	E2f2	4	136172394	136196057	+	4882	protein_coding	E2F transcription factor 2 [Source:MGI Symbol;Acc:MGI:1096341]	E2F
ENSMUSG0000001984	549.1520487	765.7251062	421.1688055	785.5339501	1092.339112	951.6188742	578.6819868	943.1639789	-0.704266376	0.0067051	0.037803	Rev3l	10	39732118	39875211	+	13293	protein_coding	REV3 like, DNA directed polymerase zeta catalytic subunit [Source:MGI Symbol;Acc:MGI:1337131]	-
ENSMUSG0000005843	8.016818229	1.053266996	32.39760042	0.861331086	0.987648384	0	13.82256188	0.61632649	4.473448182	0.0067162	0.0378514	Btnl4	17	34469042	34475937	-	1761	protein_coding	butyrophilin-like 4 [Source:MGI Symbol;Acc:MGI:1932036]	-
ENSMUSG0000004229	374.7862522	376.0163176	408.8800605	278.2099406	281.4797894	264.662918	386.5608768	274.784216	0.492041781	0.0067223	0.0378745	Gm5617	9	48495442	48495972	+	531	protein_coding	predicted gene 5617 [Source:MGI Symbol;Acc:MGI:3643566]	-
ENSMUSG0000003008	1301.73086	2067.563113	1843.311748	1019.816005	1267.152876	1174.441699	1737.53524	1153.803527	0.590683723	0.0067366	0.0379438	Klf15	6	90462576	90475238	+	2791	protein_coding	Kruppel-like factor 15 [Source:MGI Symbol;Acc:MGI:1929988]	zf-C2
ENSMUSG0000002920	1398.934781	1526.183877	1299.255493	911.2882886	1126.906806	1050.867468	1408.124717	1029.687521	0.451977277	0.0067554	0.0380381	Ugdh	5	65413221	65435949	-	6752	protein_coding	UDP-glucose dehydrogenase [Source:MGI Symbol;Acc:MGI:1306785]	-
ENSMUSG0000000204	381.8009682	290.7016909	351.9049701	271.319292	164.9372801	180.0097052	341.4692097	205.4220924	0.73157305	0.0067592	0.0380477	Trappc6a	7	19508395	19516145	+	1589	protein_coding	trafficking protein particle complex 6A [Source:MGI Symbol;Acc:MGI:1914341]	-
ENSMUSG0000004273	32.06727292	20.01207293	43.56918677	194.6608253	72.09833201	36.97496648	31.88284421	101.2447079	-1.670150597	0.0067652	0.0380701	Ttc9	12	81631249	81667557	+	4778	protein_coding	tetratricopeptide repeat domain 9 [Source:MGI Symbol;Acc:MGI:1916730]	-
ENSMUSG0000010389	94.19761419	53.7166168	118.4188153	53.40252731	27.65415474	30.16378845	88.77768211	37.07349017	1.254888635	0.0067827	0.0381574	Gm30238	11	112690986	112693588	-	2603	TEC	predicted gene, 30238 [Source:MGI Symbol;Acc:MGI:5589397]	-
ENSMUSG0000001771	4.008409114	7.372868973	16.75737953	56.84785165	17.77767091	37.94799192	9.379552538	37.52450482	-2.011868821	0.0068289	0.0384057	Birc5	11	117849251	117855743	+	3402	protein_coding	baculoviral IAP repeat-containing 5 [Source:MGI Symbol;Acc:MGI:1203517]	-
ENSMUSG0000002099	2105.416887	1787.394092	3168.261889	1627.915752	1360.979473	1528.622956	2353.690956	1505.839394	0.644137599	0.0068773	0.0386441	Pnn	12	59066884	59073998	+	5762	protein_coding	pinin [Source:MGI Symbol;Acc:MGI:1100514]	-
ENSMUSG0000004863	16.03363646	13.69247095	136.2933535	11.19730411	13.82707737	5.838152603	55.3398203	10.28751136	2.424966259	0.0068776	0.0386441	A730049H05R	6	92816478	92847174	+	3739	protein_coding	RIKEN cDNA A730049H05 gene [Source:MGI Symbol;Acc:MGI:1921766]	-
ENSMUSG0000005331	69.14505722	130.6051075	153.050733	60.29317599	63.20949656	41.84009365	117.6002993	55.1142554	1.091331113	0.0068777	0.0386441	Nrgn	9	37544492	37552904	-	1453	protein_coding	neurogranin [Source:MGI Symbol;Acc:MGI:1927184]	-
ENSMUSG0000004706	3.006306836	3.159800988	15.64022089	0	0	0	7.268776239	0	5.391758301	0.0068796	0.0386441	Gm9817	13	45078692	45081222	+	2531	protein_coding	predicted gene 9817 [Source:MGI Symbol;Acc:MGI:3809102]	-
ENSMUSG0000002661	1065.234722	1005.869981	683.7010848	1268.740689	1512.089676	1346.6672	918.2685961	1375.832522	-0.582830513	0.0068858	0.0386563	Bpnt1	1	185332149	185357777	+	2308	protein_coding	bisphosphate 3'-nucleotidase 1 [Source:MGI Symbol;Acc:MGI:1338800]	-
ENSMUSG0000004299	160.3363646	172.7357874	158.6365262	228.2527377	254.813283	249.094511	163.9028927	244.0535106	-0.573777402	0.0068859	0.0386563	Borcs5	6	134639510	134711187	+	2506	protein_coding	BLOC-1 related complex subunit 5 [Source:MGI Symbol;Acc:MGI:1915024]	-
ENSMUSG0000006044	27.05676152	44.23721384	36.86623496	66.32249359	95.80189322	67.13875493	36.05340344	76.42104725	-1.084290082	0.0068883	0.0386563	Sycp2	2	178345293	178407685	-	5967	protein_coding	synaptonemal complex protein 2 [Source:MGI Symbol;Acc:MGI:1933281]	-
ENSMUSG0000004021	472.9922755	312.8202978	497.1355927	612.4064019	663.6997139	691.8210834	427.6493887	655.975733	-0.61720841	0.0069	0.0387122	Emp3	7	45918023	45921404	-	1071	protein_coding	epithelial membrane protein 3 [Source:MGI Symbol;Acc:MGI:1098729]	-
ENSMUSG0000002119	3.006306836	1.053266996	2.23431727	20.67194605	11.8517806	10.70327977	2.097963701	14.40900214	-2.778689122	0.0069092	0.0387525	Unc79	12	102948859	103184065	+	14265	protein_coding	unc-79 homolog [Source:MGI Symbol;Acc:MGI:2684729]	-
ENSMUSG0000003427	496.0406279	294.9147589	635.6632634	262.7059811	294.3192184	263.6898926	475.5395501	273.5716973	0.797486602	0.0069148	0.0387721	Jdp2	12	85599027	85639878	+	2148	protein_coding	Jun dimerization protein 2 [Source:MGI Symbol;Acc:MGI:1932093]	TF_b
ENSMUSG0000006161	698.4652882	622.4807947	1005.442772	568.4785165	444.4417727	470.9443099	775.4629515	494.621533	0.64794784	0.0069255	0.0388204	U2af1	17	31647082	31658754	-	974	protein_coding	U2 small nuclear ribonucleoprotein auxiliary factor (U2AF) 1 [Source:MGI Symbol;Acc:MGI:98884]	-
ENSMUSG0000003616	396.8325023	408.6675945	402.1771087	313.5245152	278.5168442	252.0135873	402.5590685	281.3516489	0.515962689	0.0069284	0.0388248	Surf6	2	26888628	26902879	-	4071	protein_coding	surfeit gene 6 [Source:MGI Symbol;Acc:MGI:98447]	-
ENSMUSG0000007527	206.4330694	153.7769814	211.1429821	364.3430492	245.9244476	315.2602405	190.451011	308.5092458	-0.696799937	0.0069441	0.0389015	Ttc30b	2	75935849	75938585	-	2737	protein_coding	tetratricopeptide repeat domain 30B [Source:MGI Symbol;Acc:MGI:1919671]	-
ENSMUSG0000004383	163.3426714	154.8302484	243.5405825	106.8050546	121.4807512	95.35649251	187.2378341	107.8807661	0.794555093	0.0069544	0.0389474	2900005J15Rik	5	25100983	25110109	+	2928	processed_transcrip	RIKEN cDNA 2900005J15 gene [Source:MGI Symbol;Acc:MGI:1914511]	-
ENSMUSG0000004147	492.0322188	658.2918726	191.0341266	235.1433864	221.233238	183.901807	447.119406	213.4261438	1.067108761	0.0069711	0.0390217	Syt15	14	34219595	34230420	+	5164	protein_coding	synaptotagmin XV [Source:MGI Symbol;Acc:MGI:2442166]	-
ENSMUSG0000007824	62.13034127	73.72868973	44.68634541	22.39460823	21.72826444	34.05589018	60.18179214	26.05958762	1.21018997	0.0069718	0.0390217	Hmga1b	11	120762794	120764419	+	1626	protein_coding	high mobility group AT-hook 1B [Source:MGI Symbol;Acc:MGI:96161]	HMG
ENSMUSG0000003416	1169.453359	1090.131341	769.7222997	1449.620217	1690.854033	1366.127709	1009.769	1502.200653	-0.572656409	0.0069764	0.0390354	Irf2bpl	12	86880701	86884798	-	4098	protein_coding	interferon regulatory factor 2 binding protein-like [Source:MGI Symbol;Acc:MGI:2442463]	-
ENSMUSG0000001535	314.6601155	193.8011273	180.9796989	345.3937653	447.4047178	368.7766394	229.8136472	387.1917075	-0.751065738	0.0069818	0.0390541	Pcolce2	9	95637601	95698096	+	4625	protein_coding	procollagen C-endopeptidase enhancer 2 [Source:MGI Symbol;Acc:MGI:1923727]	-
ENSMUSG0000002678	339.7126725	396.0283905	331.7961147	466.8414484	493.8241919	568.2468533	355.8457259	509.6374979	-0.517796887	0.0070063	0.0391798	Abi2	1	60409619	60481158	+	8543	protein_coding	abl-interactor 2 [Source:MGI Symbol;Acc:MGI:106913]	-
ENSMUSG0000002885	1580.315293	1527.237144	1900.286839	1411.721649	968.8830645	987.6208153	1669.279759	1122.741843	0.571690574	0.0070123	0.0392016	Nudc	4	133532542	133545996	-	1425	protein_coding	nudC nuclear distribution protein [Source:MGI Symbol;Acc:MGI:106014]	-
ENSMUSG0000002091	7.01471595	3.159800988	0	11.19730411	23.70356121	33.08286475	3.391505646	22.66124336	-2.719181181	0.0070147	0.0392034	Krt36	11	100102007	100105626	-	1671	protein_coding	keratin 36 [Source:MGI Symbol;Acc:MGI:109364]	-
ENSMUSG0000006604	72.15136406	92.68749566	129.5904017	45.65054754	57.28360626	43.78614452	98.14308713	48.9067661	1.004051179	0.0070172	0.0392052	Med18	4	132458731	132463921	-	1998	protein_coding	mediator complex subunit 18 [Source:MGI Symbol;Acc:MGI:1914469]	-
ENSMUSG0000003743	846.7764254	948.9935635	1084.761035	753.6646999	573.823711	680.1447782	960.1770079	669.211063	0.520138328	0.0070215	0.0392121	Slc30a1	1	191906767	191913247	+	5244	protein_coding	solute carrier family 30 (zinc transporter), member 1 [Source:MGI Symbol;Acc:MGI:1345281]	-
ENSMUSG0000003031	1576.306884	1401.898372	1224.405864	895.784329	1030.117264	1089.788486	1400.870373	1005.230026	0.479278758	0.0070226	0.0392121	Atg7	6	114643097	114860614	+	6968	protein_coding	autophagy related 7 [Source:MGI Symbol;Acc:MGI:1921494]	-
ENSMUSG0000005181	91.19130735	112.6995686	69.26383538	227.3914066	116.5425093	182.9287816	91.05157044	175.6208991	-0.948041623	0.0070272	0.039226	Sox12	2	152393611	152398063	-	4453	protein_coding	SRY (sex determining region Y)-box 12 [Source:MGI Symbol;Acc:MGI:98360]	HMG
ENSMUSG0000003576	865.8163687	1063.799666	678.1152916	560.7265367	600.4902173	591.5994637	869.2437755	584.2720726	0.573479052	0.0070475	0.0393276	Dym	18	75018772	75286966	+	2471	protein_coding	dymeclin [Source:MGI Symbol;Acc:MGI:1918480]	-
ENSMUSG0000004139	907.9046644	963.7393014	1061.300703	1225.674135	1299.745273	1406.021752	977.6482231	1310.480387	-0.422809721	0.0070561	0.039364	Mdfic	6	15720661	15802169	+	7427	protein_coding	MyoD family inhibitor domain containing [Source:MGI Symbol;Acc:MGI:104611]	-
ENSMUSG0000008662	86.18079596	73.72868973	86.02121491	50.81853405	40.49358373	36.00194105	81.9769002	42.43801961	0.94705717	0.0070798	0.0394845	2810403D21Ri	X	108833800	108974906	+	8345	processed_transcrip	RIKEN cDNA 2810403D21 gene [Source:MGI Symbol;Acc:MGI:1917214]	-
ENSMUSG0000002609	748.5704021	714.1150234	1109.338525	503.017354	601.4778657	574.0850059	857.3413168	559.5267419	0.615565171	0.0070858	0.0395064	Stk17b	1	53755506	53785224	-	5185	protein_coding	serine/threonine kinase 17b (apoptosis-inducing) [Source:MGI Symbol;Acc:MGI:2138162]	-
ENSMUSG0000002685	523.0973894	366.5369146	325.0931628	593.457118	613.3296463	664.5763713	404.9091556	623.7877118	-0.622515152	0.0070912	0.0395244	Dolpp1	2	30392254	30400529	+	3233	protein_coding	dolichyl pyrophosphate phosphatase 1 [Source:MGI Symbol;Acc:MGI:1914093]	-
ENSMUSG0000001829	3133.573825	3058.687357	3629.648406	5206.746412	4028.617757	4268.662578	3273.969863	4501.342249	-0.459475204	0.0071135	0.039637	Pfn1	11	70651850	70654644	-	2405	protein_coding	profilin 1 [Source:MGI Symbol;Acc:MGI:97549]	-
ENSMUSG0000001579	1241.604723	936.3543595	1083.643876	808.7898894	725.9215621	803.7190083	1087.200986	779.4768199	0.480046223	0.0071304	0.0397192	Surf1	2	26913381	26916530	-	1753	protein_coding	surfeit gene 1 [Source:MGI Symbol;Acc:MGI:98443]	-
ENSMUSG0000005371	58.12193216	57.92968479	49.15497995	79.24245987	147.1596092	100.2216197	55.06886563	108.8745629	-0.9807813	0.0071338	0.0397261	4732471J01Rik	7	25376819	25566417	+	4332	antisense	RIKEN cDNA 4732471J01 gene [Source:MGI Symbol;Acc:MGI:3603586]	-
ENSMUSG0000010747	194.4078421	141.1377775	165.339478	228.2527377	266.6650636	294.8267064	166.9616992	263.2481692	-0.655648166	0.007151	0.0398103	Zfp862-ps	6	48504337	48534832	+	6979	transcribed_unitary	zinc finger protein 862, pseudogene [Source:MGI Symbol;Acc:MGI:1889827]	-
ENSMUSG0000002901	1374.834326	1341.862153	1178.60236	1652.894353	1693.816978	1793.285874	1298.449613	1713.332402	-0.399742763	0.0071736	0.0399241	Pmpcb	5	21737141	21757152	+	5141	protein_coding	peptidase (mitochondrial processing) beta [Source:MGI Symbol;Acc:MGI:1920328]	-
ENSMUSG0000003817	83.17448913	53.7166168	29.04612452	180.0181969	92.83894807	114.8170012	55.31241015	129.2247154	-1.222318508	0.0071815	0.0399504	Enpp6	8	46986877	47096762	+	6412	protein_coding	ectonucleotide pyrophosphatase/phosphodiesterase 6 [Source:MGI Symbol;Acc:MGI:2445171]	-
ENSMUSG0000002271	1.002102279	0	0	11.19730411	5.925890302	8.757228904	0.334034093	8.626807773	-4.512411617	0.0071841	0.0399504	Tmem114	16	8409275	8425136	-	1407	protein_coding	transmembrane protein 114 [Source:MGI Symbol;Acc:MGI:1921970]	-
ENSMUSG0000000372	1925.038477	1149.114293	2028.760082	1005.173377	1174.313928	1076.16613	1700.970951	1085.217812	0.648435473	0.0071864	0.0399504	Insig2	1	121304353	121332589	-	3473	protein_coding	insulin induced gene 2 [Source:MGI Symbol;Acc:MGI:1920249]	-
ENSMUSG0000003355	1328.787621	1328.169682	1017.731517	1496.132096	1803.445949	1987.890961	1224.896273	1762.489669	-0.524567815	0.0071869	0.0399504	Fam20b	1	156678532	156719086	-	8770	protein_coding	family with sequence similarity 20, member B [Source:MGI Symbol;Acc:MGI:2443990]	-
ENSMUSG0000004113	2221.660752	2760.612797	2459.983315	1755.392752	1834.063049	1968.430453	2480.752288	1852.628751	0.421234611	0.0071958	0.0399868	Smc1a	X	152016428	152062694	+	5769	protein_coding	structural maintenance of chromosomes 1A [Source:MGI Symbol;Acc:MGI:1344345]	-
ENSMUSG0000009870	73.15346634	81.1015587	117.3016567	40.48256102	53.33301272	42.81311909	90.51889391	45.54289761	0.990908291	0.0071977	0.0399868	1500015A07Ri	18	61726390	61735917	+	2296	antisense	RIKEN cDNA 1500015A07 gene [Source:MGI Symbol;Acc:MGI:1916232]	-
ENSMUSG0000002448	673.4127312	657.2386056	739.5590165	979.3334443	855.3035003	953.5649251	690.0701178	929.4006232	-0.429978042	0.0072063	0.0400226	Yipf5	18	40204865	40219399	-	2329	protein_coding	Yip1 domain family, member 5 [Source:MGI Symbol;Acc:MGI:1914430]	-
ENSMUSG0000008576	8.016818229	4.213067984	4.468634541	18.0879528	22.71591283	25.29866128	5.566173585	22.03417563	-1.977015557	0.0072137	0.0400523	2810430I11Rik	2	27882925	27887052	-	3416	processed_transcrip	RIKEN cDNA 2810430I11 gene [Source:MGI Symbol;Acc:MGI:3590515]	-
ENSMUSG0000010065	3.006306836	5.26633498	2.23431727	13.78129737	12.83942899	29.19076301	3.502319696	18.60382979	-2.404847071	0.007216	0.040053	F730311O21Ri	1	132342023	132349626	+	5447	lincRNA	RIKEN cDNA F730311O21 gene [Source:MGI Symbol;Acc:MGI:3643355]	-
ENSMUSG0000004254	1079.264154	1131.208754	1051.246276	819.1258624	838.5134778	843.6130511	1087.239728	833.7507971	0.383114579	0.0072227	0.0400781	Asxl1	2	153345829	153404007	+	7444	protein_coding	additional sex combs like 1 [Source:MGI Symbol;Acc:MGI:2684063]	-
ENSMUSG0000003087	804.6881297	840.5070629	490.4326409	931.9602346	1328.387076	1180.279851	711.8759445	1146.875721	-0.687427394	0.0072678	0.0403164	Gga2	7	121986722	122021222	-	9769	protein_coding	golgi associated, gamma adaptin ear containing, ARF binding protein 2 [Source:MGI Symbol;Acc:MGI:1921355]	-
ENSMUSG0000002019	8515.865164	7461.3434	4674.19173	8664.12939	12850.29312	11758.03934	6883.800098	11090.82062	-0.688016808	0.0072976	0.0404682	Mknk2	10	80665327	80678112	-	3892	protein_coding	MAP kinase-interacting serine/threonine kinase 2 [Source:MGI Symbol;Acc:MGI:894279]	-
ENSMUSG0000002487	3024.344677	2532.053859	1869.006397	3570.21735	3569.361259	3723.768335	2475.134977	3621.115648	-0.548747807	0.007302	0.0404682	Rab1b	19	5099207	5106996	-	1835	protein_coding	RAB1B, member RAS oncogene family [Source:MGI Symbol;Acc:MGI:1923558]	-
ENSMUSG0000004282	243.5108537	136.9247095	189.916968	260.9833189	381.2322761	332.7746984	190.1175104	324.9967645	-0.772310431	0.0073038	0.0404682	Fgf11	11	69796068	69801857	-	3044	protein_coding	fibroblast growth factor 11 [Source:MGI Symbol;Acc:MGI:109167]	-
ENSMUSG0000006112	3.006306836	0	0	10.33597303	8.888835454	21.40655954	1.002102279	13.54378934	-3.714257567	0.0073047	0.0404682	Cyp4f39	17	32468462	32492479	+	1599	protein_coding	cytochrome P450, family 4, subfamily f, polypeptide 39 [Source:MGI Symbol;Acc:MGI:2445210]	-
ENSMUSG0000002087	447.9397185	510.8344931	704.9270988	876.8350451	809.8716747	807.61111	554.5671035	831.4392766	-0.585039158	0.0073059	0.0404682	Slc35b1	11	95384692	95391776	+	2223	protein_coding	solute carrier family 35, member B1 [Source:MGI Symbol;Acc:MGI:1343133]	-
ENSMUSG0000004252	2484.211549	2841.714355	2808.536809	1983.64549	2115.542838	2150.386209	2711.487571	2083.191512	0.380296851	0.0073183	0.0405188	Ubap2l	3	90000140	90052628	-	18659	protein_coding	ubiquitin-associated protein 2-like [Source:MGI Symbol;Acc:MGI:1921633]	-
ENSMUSG0000002620	213.4477853	267.529817	199.9713957	395.3509683	333.8251537	310.3951134	226.9829994	346.5237451	-0.610744664	0.0073194	0.0405188	Tuba4a	1	75214228	75219865	-	3701	protein_coding	tubulin, alpha 4A [Source:MGI Symbol;Acc:MGI:1095410]	-
ENSMUSG0000004072	265.5571038	278.062487	192.1512853	494.4040431	352.590473	341.5319273	245.2569587	396.1754811	-0.691881794	0.0073284	0.0405565	Scamp5	9	57441327	57468060	-	3707	protein_coding	secretory carrier membrane protein 5 [Source:MGI Symbol;Acc:MGI:1928948]	-
ENSMUSG0000003448	579.215117	391.8153225	475.9095786	708.0141524	668.6379558	743.3914314	482.3133394	706.6811799	-0.55075079	0.0073446	0.0406343	Kdelc2	9	53384025	53401867	+	3642	protein_coding	KDEL (Lys-Asp-Glu-Leu) containing 2 [Source:MGI Symbol;Acc:MGI:1923765]	-
ENSMUSG0000008692	4.008409114	1.053266996	0	9.474641942	18.76531929	16.54143237	1.68722537	14.9271312	-3.113333366	0.0073549	0.0406793	Gm11788	11	120489406	120492394	-	562	antisense	predicted gene 11788 [Source:MGI Symbol;Acc:MGI:3649943]	-
ENSMUSG0000004022	145.3048304	42.13067984	59.20940767	167.9595617	178.7643575	220.8767735	82.21497264	189.2002309	-1.199977352	0.0073594	0.040692	Gpr34	X	13632089	13640858	+	2090	protein_coding	G protein-coupled receptor 34 [Source:MGI Symbol;Acc:MGI:1346334]	-
ENSMUSG0000009577	0	3.159800988	0	35.31457451	21.72826444	0.973025434	1.053266996	19.33862146	-4.198255367	0.0073666	0.0406954	Igkv14-111	6	68256389	68256869	+	368	IG_V_gene	immunoglobulin kappa variable 14-111 [Source:MGI Symbol;Acc:MGI:4439863]	-
ENSMUSG0000006175	338.7105702	401.2947255	389.8883637	291.1299069	183.7025994	245.2024093	376.6312198	240.0116385	0.648543216	0.0073669	0.0406954	Kalrn	16	33969073	34573532	-	24050	protein_coding	kalirin, RhoGEF kinase [Source:MGI Symbol;Acc:MGI:2685385]	-
ENSMUSG0000003047	252.5297742	252.7840791	225.6660443	355.7297383	346.6645827	336.6668001	243.6599659	346.353707	-0.507059126	0.0073687	0.0406954	Zdhhc13	7	48789003	48827440	+	2410	protein_coding	zinc finger, DHHC domain containing 13 [Source:MGI Symbol;Acc:MGI:1919227]	-
ENSMUSG0000002203	4.008409114	2.106533992	4.468634541	18.94928388	15.80237414	16.54143237	3.527859216	17.0976968	-2.279878167	0.0073687	0.0406954	Esco2	14	65819038	65833994	-	6648	protein_coding	establishment of sister chromatid cohesion N-acetyltransferase 2 [Source:MGI Symbol;Acc:MGI:1919238]	-
ENSMUSG0000006869	324.6811383	170.6292534	160.8708435	343.6711032	475.0588726	369.7496648	218.7270784	396.1598802	-0.855428881	0.0074184	0.0409578	Flnc	6	29433256	29461883	+	9402	protein_coding	filamin C, gamma [Source:MGI Symbol;Acc:MGI:95557]	-
ENSMUSG0000004257	176.370001	245.4112101	207.7915062	114.5570344	146.1719608	129.4123827	209.8575724	130.047126	0.691149942	0.0074225	0.0409683	4632404H12Ri	3	89767511	89773240	-	5730	lincRNA	RIKEN cDNA 4632404H12 gene [Source:MGI Symbol;Acc:MGI:1921284]	-
ENSMUSG0000005344	5.010511393	3.159800988	2.23431727	17.22662171	24.69120959	11.67630521	3.468209884	17.86471217	-2.355104368	0.0074469	0.0410907	Adamts19	18	58836764	59053678	+	4666	protein_coding	a disintegrin-like and metallopeptidase (reprolysin type) with thrombospondin type 1 motif, 19 [Source:MGI Symbol;Acc:MGI:244	-
ENSMUSG0000005580	494.0364234	425.5198664	416.7001709	627.9103614	633.082614	582.8422348	445.4188202	614.6117367	-0.464111807	0.0074553	0.041125	Fmnl1	11	103171107	103198901	+	4886	protein_coding	formin-like 1 [Source:MGI Symbol;Acc:MGI:1888994]	-
ENSMUSG0000002854	0	0	0	23.25593931	0.987648384	2.919076301	0	9.054221332	-5.546648441	0.0074617	0.0411484	Slc5a9	4	111875375	111902918	-	5184	protein_coding	solute carrier family 5 (sodium/glucose cotransporter), member 9 [Source:MGI Symbol;Acc:MGI:2140201]	-
ENSMUSG0000002074	3265.851326	3191.398998	3295.617974	3857.901932	4267.628666	4342.612511	3250.956099	4156.047703	-0.354278444	0.0074873	0.041277	Pafah1b1	11	74673949	74724670	-	9620	protein_coding	platelet-activating factor acetylhydrolase, isoform 1b, subunit 1 [Source:MGI Symbol;Acc:MGI:109520]	-
ENSMUSG0000011538	51.10721621	215.9197342	194.3856025	74.93580445	59.25890302	42.81311909	153.8041843	59.00260885	1.380139231	0.0075028	0.0413504	Eppk1	15	76101481	76120195	-	12332	protein_coding	epiplakin 1 [Source:MGI Symbol;Acc:MGI:2386306]	-
ENSMUSG0000002508	3.006306836	0	1.117158635	65.4611625	2.962945151	1.946050868	1.37448849	23.45671951	-4.083855564	0.0075107	0.0413816	Gfra1	19	58235604	58455909	-	5476	protein_coding	glial cell line derived neurotrophic factor family receptor alpha 1 [Source:MGI Symbol;Acc:MGI:1100842]	-
ENSMUSG0000003607	1469.08194	1015.349384	1607.591276	1794.152651	2078.012199	2253.526905	1364.007534	2041.897252	-0.582039701	0.0075193	0.0414168	Sigmar1	4	41738493	41756157	-	3136	protein_coding	sigma non-opioid intracellular receptor 1 [Source:MGI Symbol;Acc:MGI:1195268]	-
ENSMUSG0000006814	69.14505722	85.31462668	72.61531129	46.51187862	35.55534181	32.10983931	75.69166507	38.05901992	0.988613431	0.0075392	0.0415146	Gm10232	17	3044014	3044733	-	720	processed_pseudog	predicted pseudogene 10232 [Source:MGI Symbol;Acc:MGI:3641637]	-
ENSMUSG0000002018	446.9376163	260.156948	463.6208336	417.7455765	898.7600292	885.4531447	390.238466	733.9862502	-0.911057907	0.0075425	0.0415204	Nav3	10	109681259	110456204	-	15207	protein_coding	neuron navigator 3 [Source:MGI Symbol;Acc:MGI:2183703]	-
ENSMUSG0000003887	730.5325611	426.5731334	697.1069884	455.6441443	324.9363183	347.3700799	618.0708943	375.9835141	0.716491685	0.0075665	0.04164	Bpgm	6	34476207	34505613	+	3588	protein_coding	2,3-bisphosphoglycerate mutase [Source:MGI Symbol;Acc:MGI:1098242]	-
ENSMUSG0000002880	3333.994281	3284.086494	4812.719401	2652.899744	2536.281049	2746.8508	3810.266725	2645.343864	0.526325783	0.0075843	0.0417257	Srrm1	4	135320484	135353321	-	7299	protein_coding	serine/arginine repetitive matrix 1 [Source:MGI Symbol;Acc:MGI:1858303]	-
ENSMUSG0000007657	1.002102279	0	0	4.306655428	4.938241919	19.46050868	0.334034093	9.568468674	-4.657710241	0.0075959	0.0417777	Igkv8-30	6	70117061	70117620	-	368	IG_V_gene	immunoglobulin kappa chain variable 8-30 [Source:MGI Symbol;Acc:MGI:3642250]	-
ENSMUSG0000010489	16.03363646	13.69247095	43.56918677	6.029317599	6.913538686	2.919076301	24.43176473	5.287310862	2.203134208	0.0076356	0.0419833	2510017J16Rik	5	3585112	3586374	+	1263	antisense	RIKEN cDNA 2510017J16 gene [Source:MGI Symbol;Acc:MGI:1913824]	-
ENSMUSG0000000242	674.4148335	766.7783732	697.1069884	487.5133944	553.0830949	533.2179377	712.7667317	524.604809	0.442551967	0.0076454	0.0420251	Hltf	3	20057811	20118490	+	5931	protein_coding	helicase-like transcription factor [Source:MGI Symbol;Acc:MGI:1196437]	-
ENSMUSG0000010630	6.012613672	23.17187391	10.05442772	2.583993257	0	0	13.07963843	0.861331086	3.860342832	0.0076492	0.0420333	Gm42765	5	30529457	30543830	+	3211	antisense	predicted gene 42765 [Source:MGI Symbol;Acc:MGI:5662902]	-
ENSMUSG0000010654	22.04625013	43.18394684	73.73246992	10.33597303	15.80237414	18.48748324	46.32088896	14.8752768	1.63999857	0.0076613	0.0420876	B230303O12R	5	146188620	146194809	-	3645	lincRNA	RIKEN cDNA B230303O12 gene [Source:MGI Symbol;Acc:MGI:2442644]	-
ENSMUSG0000000274	3703.770022	4117.220688	3978.2019	2665.81971	3203.931357	3094.220879	3933.064203	2987.990649	0.396581532	0.0076809	0.0421832	Baz1b	5	135187264	135246129	+	13667	protein_coding	bromodomain adjacent to zinc finger domain, 1B [Source:MGI Symbol;Acc:MGI:1353499]	-
ENSMUSG0000003947	1828.836658	1571.474358	1075.823766	2036.186686	2700.230681	2176.657895	1492.044927	2304.358421	-0.626756335	0.0076967	0.042243	Tnrc18	5	142724661	142817662	-	12266	protein_coding	trinucleotide repeat containing 18 [Source:MGI Symbol;Acc:MGI:3648294]	-
ENSMUSG0000002936	151.3174441	229.6122051	614.4372494	78.38112879	134.3201802	182.9287816	331.7889662	131.8766968	1.3313212	0.0076975	0.042243	Alb	5	90460897	90476602	+	2342	protein_coding	albumin [Source:MGI Symbol;Acc:MGI:87991]	-
ENSMUSG0000003083	161.3384669	181.1619233	121.7702912	243.7566972	248.8873927	237.4182058	154.7568938	243.3540986	-0.651954143	0.0076996	0.042243	Itgal	7	127296260	127335138	+	6569	protein_coding	integrin alpha L [Source:MGI Symbol;Acc:MGI:96606]	-
ENSMUSG0000002623	6126.853331	7002.11899	6505.214733	5709.763766	4078.987825	4054.596983	6544.729018	4614.449525	0.504052489	0.0077009	0.042243	Ptma	1	86526726	86530712	+	1462	protein_coding	prothymosin alpha [Source:MGI Symbol;Acc:MGI:97803]	-
ENSMUSG0000004767	562.1793783	689.8898824	570.8680626	472.0094349	418.7629147	396.0213515	607.6457744	428.9312337	0.501980203	0.0077115	0.0422887	Rpsa-ps10	3	150072658	150073542	-	885	processed_pseudog	ribosomal protein SA, pseudogene 10 [Source:MGI Symbol;Acc:MGI:3704228]	-
ENSMUSG0000004172	1066.236824	728.8607613	1426.611577	593.457118	706.1685944	691.8210834	1073.903054	663.8155986	0.694001352	0.007716	0.0422301	Coro2b	9	62419492	62537044	-	5919	protein_coding	coronin, actin binding protein, 2B [Source:MGI Symbol;Acc:MGI:2444283]	-
ENSMUSG0000002128	681.4295495	574.0305129	462.503675	885.448356	793.0816521	815.3953135	572.6545791	831.3084405	-0.537255606	0.0077319	0.0423663	Zfyve21	12	111814170	111832192	+	1919	protein_coding	zinc finger, FYVE domain containing 21 [Source:MGI Symbol;Acc:MGI:1915770]	-
ENSMUSG0000002899	841.765914	463.4374783	773.0737756	340.2257788	398.0222986	505.0002001	692.759056	414.4160925	0.741720068	0.0077353	0.0423663	Tomm7	5	23838944	23844161	-	1125	protein_coding	translocase of outer mitochondrial membrane 7 [Source:MGI Symbol;Acc:MGI:1913419]	-
ENSMUSG0000004431	537.1268213	382.3359196	625.6088357	316.1085084	356.5410665	324.9904949	515.0238589	332.5466899	0.631047834	0.0077362	0.0423663	Gpr4	7	19212538	19224174	+	2882	protein_coding	G protein-coupled receptor 4 [Source:MGI Symbol;Acc:MGI:2441992]	-
ENSMUSG0000008387	93.19551191	92.68749566	56.9750904	31.00791908	37.53063858	47.67824626	80.95269932	38.73893464	1.066991299	0.0077369	0.0423663	Gm14038	2	134762824	134763630	-	807	processed_pseudog	predicted gene 14038 [Source:MGI Symbol;Acc:MGI:3650657]	-
ENSMUSG0000005700	4.008409114	0	1.117158635	43.92788537	0.987648384	22.37958498	1.708522583	22.43170624	-3.70186558	0.0077512	0.0424272	Nxf3	X	136072099	136085255	-	3048	protein_coding	nuclear RNA export factor 3 [Source:MGI Symbol;Acc:MGI:2685230]	-
ENSMUSG0000000813	59.12403444	35.81107787	12.28874499	165.3755684	74.07362878	73.94993297	35.74128576	104.4663767	-1.544757211	0.0077548	0.0424272	Fhl2	1	43123074	43196984	-	1897	protein_coding	four and a half LIM domains 2 [Source:MGI Symbol;Acc:MGI:1338762]	-
ENSMUSG0000002883	8.016818229	16.85227194	10.05442772	487.5133944	8.888835454	28.21773758	11.64117263	174.8733225	-3.909574837	0.0077549	0.0424272	Slc30a2	4	134343181	134354484	+	3564	protein_coding	solute carrier family 30 (zinc transporter), member 2 [Source:MGI Symbol;Acc:MGI:106637]	-
ENSMUSG0000009742	3.006306836	6.319601977	1.117158635	13.78129737	24.69120959	17.51445781	3.481022483	18.66232159	-2.413923282	0.0077624	0.0424357	Gm26522	7	88278787	88283529	-	1555	lincRNA	predicted gene, 26522 [Source:MGI Symbol;Acc:MGI:5477016]	-
ENSMUSG0000002878	322.6769337	210.6533992	180.9796989	531.4412798	310.1215925	415.4818602	238.1033439	419.0149108	-0.815012434	0.0077637	0.0424357	Azin2	4	128930233	128962442	-	4024	protein_coding	antizyme inhibitor 2 [Source:MGI Symbol;Acc:MGI:2442093]	-
ENSMUSG0000010643	22.04625013	3.159800988	24.57748997	3.445324342	0	0	16.5945137	1.148441447	3.800128454	0.007764	0.0424357	Gm32051	5	105409367	105415670	+	2507	lincRNA	predicted gene, 32051 [Source:MGI Symbol;Acc:MGI:5591210]	-
ENSMUSG0000010021	382.8030704	201.1739963	605.4999803	186.9088456	224.1961831	189.7399596	396.492349	200.2816628	0.985044105	0.0077655	0.0424357	Gm8292	1	55335401	55335796	+	396	processed_pseudog	predicted gene 8292 [Source:MGI Symbol;Acc:MGI:3648460]	-
ENSMUSG0000003086	311.6538087	186.4282583	94.95848399	916.4562751	268.6403604	321.0983931	197.6801837	502.0650095	-1.344406006	0.0077774	0.0424887	Cpxm2	7	132032687	132154739	-	4622	protein_coding	carboxypeptidase X 2 (M14 family) [Source:MGI Symbol;Acc:MGI:1926006]	-
ENSMUSG0000004120	187.3931261	102.1668986	45.80350404	323.8604882	194.5667316	257.85174	111.7878429	258.7596532	-1.209207758	0.0077815	0.0424982	Pla2g2d	4	138775735	138782046	+	2243	protein_coding	phospholipase A2, group IID [Source:MGI Symbol;Acc:MGI:1341796]	-
ENSMUSG0000001362	473.9943778	483.4495512	461.3865163	962.1068226	612.3419979	612.0329978	472.9434818	728.8272728	-0.624508332	0.0077883	0.0425198	Cad	5	31054780	31078479	+	8183	protein_coding	carbamoyl-phosphate synthetase 2, aspartate transcarbamylase, and dihydroorotase [Source:MGI Symbol;Acc:MGI:1916969]	-
ENSMUSG0000002220	522.0952872	473.9701482	668.0608639	416.0229143	313.0845376	372.6687411	554.7087664	367.258731	0.593927512	0.00779	0.0425198	Ngdn	14	55015454	55024137	+	1483	protein_coding	neuroguidin, EIF4E binding protein [Source:MGI Symbol;Acc:MGI:1916216]	-
ENSMUSG0000005327	3749.866727	2834.341487	5927.643718	7420.367302	5904.162038	7227.632922	4170.617311	6850.720754	-0.71608123	0.007795	0.0425347	Aldh1a1	19	20492715	20643462	+	4934	protein_coding	aldehyde dehydrogenase family 1, subfamily A1 [Source:MGI Symbol;Acc:MGI:1353450]	-
ENSMUSG0000008495	27.05676152	14.74573795	50.27213858	54.26385839	95.80189322	89.51833991	30.69154602	79.86136384	-1.381932823	0.0078005	0.0425524	Gm5577	6	87980883	88005471	+	2100	antisense	predicted gene 5577 [Source:MGI Symbol;Acc:MGI:3648213]	-
ENSMUSG0000009797	28.0588638	18.95880593	40.21177087	10.33597303	2.962945151	9.730254338	29.0784602	7.676390839	1.912956115	0.0078358	0.0427324	Gm10605	7	99625936	99631711	+	3569	lincRNA	predicted gene 10605 [Source:MGI Symbol;Acc:MGI:3642596]	-
ENSMUSG0000004107	422.8871616	363.3771137	283.7582933	523.6893	551.1077981	509.8653273	356.6741895	528.2208085	-0.565574694	0.0078483	0.0427881	Fzd7	1	59482424	59486955	+	4532	protein_coding	frizzled class receptor 7 [Source:MGI Symbol;Acc:MGI:108570]	-
ENSMUSG0000001993	14.0294319	2.106533992	1.117158635	76.65846662	22.71591283	12.64933064	5.751041509	37.34123669	-2.688591469	0.007853	0.0428018	Epyc	10	97644068	97682454	+	2561	protein_coding	epiphycan [Source:MGI Symbol;Acc:MGI:107942]	-
ENSMUSG0000003627	2724.716096	2110.74706	2459.983315	3474.609599	3126.894783	3215.849059	2431.81549	3272.451147	-0.42834041	0.0078611	0.0428448	9530068E07Ri	11	52396428	52408728	+	3296	protein_coding	RIKEN cDNA 9530068E07 gene [Source:MGI Symbol;Acc:MGI:2654705]	-
ENSMUSG0000002391	99.20812558	136.9247095	155.2850503	85.27177747	67.16009009	63.2466532	130.4726285	71.89284025	0.856691377	0.0078668	0.0428448	Slc25a27	17	43641891	43667214	-	3935	protein_coding	solute carrier family 25, member 27 [Source:MGI Symbol;Acc:MGI:1921261]	-
ENSMUSG0000006221	626.3139241	633.0134647	621.1402012	783.8112879	831.5999391	875.7228904	626.82253	830.3780391	-0.405465869	0.007868	0.0428448	Tnfaip8	18	49979427	50107173	+	10328	protein_coding	tumor necrosis factor, alpha-induced protein 8 [Source:MGI Symbol;Acc:MGI:2147191]	-
ENSMUSG0000000102	5.010511393	4.213067984	1.117158635	23.25593931	10.86413222	21.40655954	3.446912671	18.50887703	-2.414170192	0.0078701	0.0428448	S100a4	3	90603771	90606045	+	584	protein_coding	S100 calcium binding protein A4 [Source:MGI Symbol;Acc:MGI:1330282]	-
ENSMUSG0000005869	2268.759559	2167.623478	1979.605102	2314.396627	3538.744159	3622.57369	2138.662713	3158.571492	-0.562359295	0.0078894	0.0429372	Ccser2	14	36874936	36968777	-	8715	protein_coding	coiled-coil serine rich 2 [Source:MGI Symbol;Acc:MGI:101859]	-

ENSMUSG0000004753	4.008409114	5.26633498	13.40590362	24.1172704	31.60474828	27.24471215	7.560215906	27.65557694	-1.881467767	0.0078932	0.0429433	Mis18bp1	12	65132734	65172604	-	7990	protein_coding	MIS18 binding protein 1 [Source:MGI Symbol;Acc:MGI:2145099]	-
ENSMUSG0000003878	286.6012517	408.6675945	437.926185	301.46588	150.1225543	173.1985272	377.7316771	208.2623205	0.856925113	0.0078951	0.0429433	Stap2	17	55997077	56005584	-	2569	protein_coding	signal transducing adaptor family member 2 [Source:MGI Symbol;Acc:MGI:2147039]	-
ENSMUSG0000002386	129.2711939	145.3508455	264.7665965	69.76781793	100.7401351	104.1137214	179.796212	91.54055816	0.974114846	0.0079186	0.0430482	Pde10a	17	8525372	8986648	+	12849	protein_coding	phosphodiesterase 10A [Source:MGI Symbol;Acc:MGI:1345143]	-
ENSMUSG0000002308	1312.753985	1417.697377	987.5682335	1573.651893	2028.62978	1795.231925	1239.339865	1799.1712	-0.537407138	0.007919	0.0430482	Abcc1	16	14361558	14475737	+	15092	protein_coding	ATP-binding cassette, sub-family C (CFTR/MRP), member 1 [Source:MGI Symbol;Acc:MGI:102676]	-
ENSMUSG0000002890	688.4442654	764.6718392	890.3754323	559.0038745	590.6137335	535.1639886	781.1638456	561.5938655	0.47574252	0.0079346	0.0431204	Utp11	4	124678160	124693600	-	4711	protein_coding	UTP11 small subunit processome component [Source:MGI Symbol;Acc:MGI:1914455]	-
ENSMUSG0000002756	362.7610249	341.2585067	481.4953718	257.5379946	280.492141	250.0675365	395.1716345	262.699224	0.588599291	0.0079432	0.0431551	Mrgbp	2	180581304	180586304	+	3499	protein_coding	MRG/MORF4L binding protein [Source:MGI Symbol;Acc:MGI:1920497]	-
ENSMUSG0000004308	10.02102279	89.52769467	30.16328315	13.78129737	0	0.973025434	43.23733354	4.918107601	3.116475715	0.0079482	0.0431696	Tmem82	4	141613376	141618633	-	3991	protein_coding	transmembrane protein 82 [Source:MGI Symbol;Acc:MGI:2384869]	-
ENSMUSG0000006145	196.4120466	218.0262682	220.0802511	299.7432178	287.4056797	333.7477238	211.5061886	306.9655404	-0.537748308	0.0079621	0.0432212	Nol10	12	17348458	17430095	+	3021	protein_coding	nucleolar protein 10 [Source:MGI Symbol;Acc:MGI:2684913]	-
ENSMUSG0000003890	5244.001224	6053.125427	5947.752574	4948.347087	3713.557923	3827.882056	5748.293075	4163.262355	0.465275618	0.0079623	0.0432212	Rpl12	2	32961559	32965345	+	3598	protein_coding	ribosomal protein L12 [Source:MGI Symbol;Acc:MGI:98002]	-
ENSMUSG0000006348	285.5991494	290.7016909	318.390211	179.1568658	237.0356121	167.3603746	298.2303505	194.5176175	0.617005255	0.0079658	0.0432275	Zkscan7	9	122885685	122898618	+	7253	protein_coding	zinc finger with KRAB and SCAN domains 7 [Source:MGI Symbol;Acc:MGI:3040678]	zf-C2
ENSMUSG0000002201	130.2732962	96.90056364	83.78689764	184.3248523	150.1225543	196.5511376	103.6535858	176.9995148	-0.770433331	0.0079697	0.0432359	Epsti1	14	77904239	78002657	+	2321	protein_coding	epithelial stromal interaction 1 (breast) [Source:MGI Symbol;Acc:MGI:1915168]	-
ENSMUSG0000002547	80876.6686	135373.2472	81343.67171	68613.63428	44377.01718	69595.64415	99197.8625	60862.09854	0.70476169	0.0079792	0.0432753	Cyp2e1	7	140763739	140774987	+	2158	protein_coding	cytochrome P450, family 2, subfamily e, polypeptide 1 [Source:MGI Symbol;Acc:MGI:88607]	-
ENSMUSG0000001776	90.18920508	91.63422866	69.26383538	167.0982306	115.5548609	153.7380185	83.69575637	145.4637033	-0.797225109	0.0080092	0.0434252	Zswim1	2	164822686	164826871	+	2781	protein_coding	zinc finger SWIM-type containing 1 [Source:MGI Symbol;Acc:MGI:1919221]	-
ENSMUSG0000011061	49.10301165	123.2322385	84.90405628	23.25593931	48.3947708	34.05589018	85.74643549	35.23553343	1.28569111	0.0080124	0.0434265	Lncbate1	8	108553252	108584595	-	812	lincRNA	brown adipose tissue enriched long non-coding RNA 1 [Source:MGI Symbol;Acc:MGI:1924437]	-
ENSMUSG0000007476	98.2060233	70.56888874	98.3099599	374.6790222	98.76483837	158.6031457	89.02829065	210.6823354	-1.244173033	0.0080141	0.0434265	Ism1	2	139678178	139758581	+	2965	protein_coding	isthmin 1, angiogenesis inhibitor [Source:MGI Symbol;Acc:MGI:2442963]	-
ENSMUSG0000002538	235.4940355	225.3991372	183.2140162	336.7804545	349.6275278	282.1773758	214.7023963	322.861786	-0.587857631	0.0080189	0.0434367	Faap100	11	120369055	120378764	-	5212	protein_coding	Fanconi anemia core complex associated protein 100 [Source:MGI Symbol;Acc:MGI:1919135]	-
ENSMUSG0000010318	130.2732962	124.2855055	206.6743475	99.91440593	81.97481585	65.19270406	153.7443831	82.36064195	0.89769716	0.0080243	0.0434367	Gm37090	4	156018556	156022660	-	4105	TEC	predicted gene, 37090 [Source:MGI Symbol;Acc:MGI:5610318]	-
ENSMUSG0000004774	79.16608001	52.6633498	46.92066268	104.2210614	116.5425093	111.8979249	59.58336417	110.8871652	-0.892853239	0.0080251	0.0434367	Zc3hav1l	6	38287396	38299259	-	6536	protein_coding	zinc finger CCCH-type, antiviral 1-like [Source:MGI Symbol;Acc:MGI:2443387]	-
ENSMUSG0000003466	59.12403444	62.14275277	91.60700809	39.62122994	34.56769343	24.32563584	70.95793177	32.8381864	1.107559633	0.0080253	0.0434367	Itga2b	11	102453297	102470122	-	5454	protein_coding	integrin alpha 2b [Source:MGI Symbol;Acc:MGI:96601]	-
ENSMUSG0000005987	568.191992	529.793299	815.5258037	435.8335293	436.5405856	388.2371481	637.8370316	420.2037543	0.60150976	0.0080278	0.0434375	Zfp422	6	116624016	116628999	-	3625	protein_coding	zinc finger protein 422 [Source:MGI Symbol;Acc:MGI:1914505]	zf-C2
ENSMUSG0000005426	2069.341205	2848.033957	2311.401216	1291.135297	1982.210306	1555.867669	2409.592126	1609.737757	0.582155148	0.0080301	0.0434375	Lifr	15	7090614	7197489	+	10776	protein_coding	leukemia inhibitory factor receptor [Source:MGI Symbol;Acc:MGI:96788]	-
ENSMUSG0000004822	458.9628436	577.1903139	536.2361449	347.1164275	415.7999696	305.5299862	524.1297675	356.1487944	0.557436728	0.0080468	0.043506	Mfap1b	2	121460235	121474067	-	3362	protein_coding	microfibrillar-associated protein 1B [Source:MGI Symbol;Acc:MGI:3694697]	-
ENSMUSG0000004414	864.8142664	831.0276599	776.4252515	623.603706	628.1443721	617.8711504	824.0890593	623.2064095	0.40331093	0.0080491	0.043506	1810030O07Ri	X	12654879	12673668	-	2699	protein_coding	RIKEN cDNA 1810030O07 gene [Source:MGI Symbol;Acc:MGI:1916405]	-
ENSMUSG0000007966	11.02312506	10.53266996	21.22601407	27.56259474	44.44417727	50.59732256	14.26060303	40.86803152	-1.522310439	0.0080519	0.043506	Gm17396	9	117252536	117254696	+	1655	processed_transcrip	predicted gene, 17396 [Source:MGI Symbol;Acc:MGI:4937030]	-
ENSMUSG0000006655	69.14505722	74.78195672	41.3348695	10.33597303	27.65415474	31.13681388	61.75396115	23.04231388	1.429476958	0.008052	0.043506	Gm6969	12	24638669	24639010	-	342	processed_pseudog	predicted pseudogene 6969 [Source:MGI Symbol;Acc:MGI:3645320]	-
ENSMUSG0000011671	56.1177276	58.98295178	111.7158635	36.17590559	37.53063858	25.29866128	75.6055143	33.00173515	1.193106053	0.0080652	0.0435644	AC154378.1	17	15421802	15434965	-	2109	lincRNA	novel transcript	-
ENSMUSG0000003274	399.8388092	482.3962842	459.1521991	548.6679015	639.0085043	777.4473216	447.1290975	655.0412425	-0.550711029	0.008077	0.0436156	Ccdc88a	11	29373658	29510808	+	10891	protein_coding	coiled coil domain containing 88A [Source:MGI Symbol;Acc:MGI:1925177]	-
ENSMUSG0000002780	823.728073	764.6718392	773.0737756	968.1361402	1292.831734	1031.40696	787.1578959	1097.458278	-0.479054554	0.0080914	0.0436808	Tsc22d2	3	58414715	58466783	+	11244	protein_coding	TSC22 domain family, member 2 [Source:MGI Symbol;Acc:MGI:1919283]	TSC2
ENSMUSG0000001364	532.1163099	482.3962842	513.8929722	727.8247673	664.6873623	663.6033458	509.4685221	685.3718251	-0.428088716	0.0080988	0.0437066	Sh3bp5l	11	58330724	58347728	+	6352	protein_coding	SH3 binding domain protein 5 like [Source:MGI Symbol;Acc:MGI:1933124]	-
ENSMUSG0000005058	226.475115	115.8593696	192.1512853	240.3113729	325.9239666	397.9674024	178.1619233	321.400914	-0.850282334	0.0081008	0.0437066	Lrrc4c	2	96318169	97631666	+	4626	protein_coding	leucine rich repeat containing 4C [Source:MGI Symbol;Acc:MGI:2442636]	-
ENSMUSG0000002922	81.17028457	85.31462668	189.916968	47.37320971	66.17244171	46.70522082	118.8006264	53.41695741	1.152538341	0.008108	0.0437288	Uchl1	5	66676091	66687234	+	1632	protein_coding	ubiquitin carboxy-terminal hydrolase L1 [Source:MGI Symbol;Acc:MGI:103149]	-
ENSMUSG0000003679	11.02312506	9.479402965	7.820110447	0	0.987648384	0.973025434	9.440879492	0.653557939	3.895563916	0.0081099	0.0437288	Slitrk2	X	66649318	66661393	+	8080	protein_coding	SLIT and NTRK-like family, member 2 [Source:MGI Symbol;Acc:MGI:2679449]	-
ENSMUSG0000003478	21.04414785	29.49147589	18.9916968	38.75989885	103.7030803	45.73219539	23.17577351	62.73172484	-1.433919686	0.008112	0.0437288	B3galt1	2	67565871	68122689	+	5753	protein_coding	UDP-Gal:betaGlcNAc beta 1,3-galactosyltransferase, polypeptide 1 [Source:MGI Symbol;Acc:MGI:1349403]	-
ENSMUSG0000000201	2635.528993	2489.923179	1948.32466	3093.901259	3168.376015	3471.754748	2357.92561	3244.677341	-0.460364353	0.0081238	0.0437802	Idh3g	X	73778963	73786897	-	2701	protein_coding	isocitrate dehydrogenase 3 (NAD+), gamma [Source:MGI Symbol;Acc:MGI:1099463]	-
ENSMUSG0000004883	0	0	0	15.50395954	1.975296767	4.865127169	0	7.448127826	-5.264727753	0.0081306	0.0438039	Vstm2a	11	16257724	16427310	+	4765	protein_coding	V-set and transmembrane domain containing 2A [Source:MGI Symbol;Acc:MGI:2384826]	-
ENSMUSG0000004879	1254.632053	1217.576647	1091.463987	741.6060647	907.6488647	941.8886199	1187.890896	863.7145164	0.460347137	0.0081437	0.0438623	Cep120	18	53681724	53744547	-	4553	protein_coding	centrosomal protein 120 [Source:MGI Symbol;Acc:MGI:2147298]	-
ENSMUSG0000002631	692.4526745	616.1611927	585.3911249	398.7962926	520.4906982	392.1292498	631.3349974	437.1387469	0.531041838	0.008165	0.0439639	Hdac4	1	91928779	92195699	-	15813	protein_coding	histone deacetylase 4 [Source:MGI Symbol;Acc:MGI:3036234]	-
ENSMUSG0000003953	2413.062287	2576.291072	2287.940885	1826.883233	1810.359487	1987.890961	2425.764748	1875.04456	0.3716041	0.0081945	0.044104	Stau1	2	166947549	166996299	-	6065	protein_coding	staufen double-stranded RNA binding protein 1 [Source:MGI Symbol;Acc:MGI:1338864]	-
ENSMUSG0000011460	64.13454583	107.4332336	33.51475906	120.586352	201.4802703	140.1156625	68.36084616	154.0607616	-1.170192053	0.0081971	0.044104	Gm36161	13	120010390	120018931	+	1509	lincRNA	predicted gene, 36161 [Source:MGI Symbol;Acc:MGI:5595320]	-
ENSMUSG0000002279	2491.226265	2790.104273	1837.725955	1551.257285	1775.791794	1613.276169	2373.018831	1646.775083	0.52727742	0.008198	0.044104	Arhgap31	16	38598340	38713274	-	11141	protein_coding	Rho GTPase activating protein 31 [Source:MGI Symbol;Acc:MGI:1333857]	-
ENSMUSG0000006356	114.2396598	73.72868973	60.3265663	347.1164275	85.92540939	177.0906289	82.76497193	203.3774886	-1.297084985	0.0082189	0.0442035	Col23a1	11	51289920	51583918	+	5736	protein_coding	collagen, type XXIII, alpha 1 [Source:MGI Symbol;Acc:MGI:2653243]	-
ENSMUSG0000003040	256.5381833	185.3749913	188.7998094	106.8050546	138.2707737	140.1156625	210.2376613	128.3971636	0.713597935	0.0082258	0.0442277	Gipr	7	19156061	19166127	-	3482	protein_coding	gastric inhibitory polypeptide receptor [Source:MGI Symbol;Acc:MGI:1352753]	-
ENSMUSG0000005135	114.2396598	71.62215573	44.68634541	298.0205556	123.456048	124.5472555	76.84938697	182.007953	-1.24321979	0.0082324	0.0442399	Ncald	15	37366175	37792570	-	8581	protein_coding	neurocalcin delta [Source:MGI Symbol;Acc:MGI:1196326]	-
ENSMUSG0000004379	35.07357975	17.90553893	31.28044179	49.95720296	82.96246423	62.27362776	28.08652016	65.06443165	-1.209794957	0.0082328	0.0442399	Prr33	7	142491074	142506771	-	2348	lincRNA	proline rich 33 [Source:MGI Symbol;Acc:MGI:3642289]	-
ENSMUSG0000002943	633.3286401	595.0958528	626.7259944	515.0759892	362.4669568	262.7168671	618.3834957	380.0866044	0.701179738	0.0082429	0.0442815	Bcl7a	5	123343834	123374992	+	9490	protein_coding	B cell CLL/lymphoma 7A [Source:MGI Symbol;Acc:MGI:1924295]	-
ENSMUSG0000004114	20.04204557	17.90553893	8.937269082	144.7036224	31.60474828	18.48748324	15.62828453	64.9319513	-2.054616616	0.0082525	0.0443207	Dnah7b	1	46066315	46373546	+	19980	protein_coding	dynein, axonemal, heavy chain 7B [Source:MGI Symbol;Acc:MGI:2684953]	-
ENSMUSG0000003975	2078.360126	2448.845766	2891.206548	1723.523502	1826.161862	1830.260841	2472.804147	1793.315402	0.463407576	0.008258	0.044337	Fbxl5	5	43744615	43821638	-	9740	protein_coding	F-box and leucine-rich repeat protein 5 [Source:MGI Symbol;Acc:MGI:2152883]	-
ENSMUSG0000003035	22.04625013	16.85227194	10.05442772	165.3755684	18.76531929	28.21773758	16.31764993	70.78620843	-2.116964184	0.0082754	0.0444181	Tspan11	6	127887589	127953977	+	5326	protein_coding	tetraspanin 11 [Source:MGI Symbol;Acc:MGI:1915748]	-
ENSMUSG0000002126	4.008409114	4.213067984	3.351475906	48.23454079	9.876483837	9.730254338	3.857651002	22.61375966	-2.554907342	0.0082864	0.0444644	Degs2	12	108678711	108702315	-	2583	protein_coding	delta(4)-desaturase, sphingolipid 2 [Source:MGI Symbol;Acc:MGI:1917309]	-
ENSMUSG0000000291	404.8493206	528.740032	883.6724805	459.9507997	211.3567541	252.0135873	605.7539444	307.7737137	0.975541915	0.0082985	0.0445163	Arrdc2	8	70835129	70839720	-	3232	protein_coding	arrestin domain containing 2 [Source:MGI Symbol;Acc:MGI:1918057]	-
ENSMUSG0000003417	431.9060821	461.3309443	412.2315364	318.6925017	317.0351312	311.3681388	435.1561876	315.6985906	0.463066518	0.0083189	0.0446033	Rhbdd3	11	5098926	5106093	+	2790	protein_coding	rhomboid domain containing 3 [Source:MGI Symbol;Acc:MGI:2444684]	-
ENSMUSG0000003225	44.09250026	51.61008281	44.68634541	149.0102778	64.19714494	87.57228904	46.79630949	100.2599039	-1.101690704	0.0083195	0.0446033	Lca5	9	83390293	83441127	-	6306	protein_coding	Leber congenital amaurosis 5 (human) [Source:MGI Symbol;Acc:MGI:1923032]	-
ENSMUSG0000002727	95.19971647	140.0845105	138.5276708	162.7915752	243.9491508	233.5261041	124.6039659	213.4222767	-0.776443944	0.0083258	0.0446246	Mkks	2	136873780	136891389	-	3227	protein_coding	McKusick-Kaufman syndrome [Source:MGI Symbol;Acc:MGI:1891836]	-
ENSMUSG0000004940	29.06096608	38.97087886	60.3265663	150.73294	51.35771595	112.8709503	42.78613708	104.9872021	-1.299165289	0.0083294	0.0446312	Rarres1	3	67478972	67515523	-	1325	protein_coding	retinoic acid receptor responder (tazarotene induced) 1 [Source:MGI Symbol;Acc:MGI:1924461]	-
ENSMUSG0000003787	1839.859784	2782.731404	3437.497121	1399.663014	1917.025513	1751.445781	2686.696103	1689.378103	0.669338593	0.0083348	0.0446472	Jmjd1c	10	67096125	67256326	+	17822	protein_coding	jumonji domain containing 1C [Source:MGI Symbol;Acc:MGI:1918614]	-
ENSMUSG0000004367	866.818471	455.0113423	1123.861587	289.4072448	467.1576855	534.1909631	815.2304668	430.2519645	0.922362765	0.0083393	0.0446583	Kcns3	12	11090202	11151056	-	3164	protein_coding	potassium voltage-gated channel, delayed-rectifier, subfamily S, member 3 [Source:MGI Symbol;Acc:MGI:1098804]	-
ENSMUSG0000008971	983.0623353	954.2598985	763.0193479	1813.963266	1101.227948	1245.472555	900.1138605	1386.887923	-0.623805732	0.0083509	0.0447006	Cbx6	15	79823896	79834688	-	10609	protein_coding	chromobox 6 [Source:MGI Symbol;Acc:MGI:3512628]	-
ENSMUSG0000004825	5.010511393	2.106533992	7.820110447	25.83993257	15.80237414	22.37958498	4.979051944	21.34063056	-2.107615615	0.008352	0.0447006	Bcl11b	12	107910403	108003602	-	8111	protein_coding	B cell leukemia/lymphoma 11B [Source:MGI Symbol;Acc:MGI:1929913]	zf-C2
ENSMUSG0000005529	4235.886332	3479.994155	3997.193597	1830.328557	3000.47579	2933.671683	3904.358028	2588.158677	0.593346822	0.0083603	0.0447214	Tmem245	4	56866923	56947437	-	17090	protein_coding	transmembrane protein 245 [Source:MGI Symbol;Acc:MGI:2445107]	-
ENSMUSG0000003530	152.3195463	171.6825204	118.4188153	237.7273796	209.3814574	252.9866128	147.4736273	233.3651499	-0.661316049	0.0083606	0.0447214	Ror1	4	100095791	100444765	+	5762	protein_coding	receptor tyrosine kinase-like orphan receptor 1 [Source:MGI Symbol;Acc:MGI:1347520]	-
ENSMUSG0000003861	5062.620712	5270.548048	5618.190777	4043.088116	4461.207749	3815.232726	5317.119846	4106.50953	0.372718337	0.0083648	0.0447311	Mcl1	3	95658788	95663176	+	3418	protein_coding	myeloid cell leukemia sequence 1 [Source:MGI Symbol;Acc:MGI:101769]	-
ENSMUSG0000010466	26.05465924	8.426135969	35.74907633	3.445324342	4.938241919	6.811178036	23.40995718	5.064914766	2.213143403	0.0083674	0.0447323	Gm43267	5	144223725	144225520	+	1796	TEC	predicted gene 43267 [Source:MGI Symbol;Acc:MGI:5663404]	-
ENSMUSG0000002932	2458.156889	2989.171735	3226.354139	2244.628809	2148.135235	2001.513317	2891.227588	2131.425787	0.439672422	0.0083764	0.0447672	Hnrnpdl	5	100033577	100039664	-	4818	protein_coding	heterogeneous nuclear ribonucleoprotein D-like [Source:MGI Symbol;Acc:MGI:1355299]	-
ENSMUSG0000002052	1054.211597	1050.107195	1231.108816	857.0244302	808.8840263	838.7479239	1111.809203	834.8854601	0.412914709	0.0084108	0.0449385	Fam114a2	11	57482993	57518617	-	3075	protein_coding	family with sequence similarity 114, member A2 [Source:MGI Symbol;Acc:MGI:1917629]	-
ENSMUSG0000002545	1121.35245	1229.162584	1391.979659	950.9095185	967.8954161	796.9078303	1247.498231	905.2375883	0.462339371	0.0084304	0.0450303	Paip1	13	119428601	119458218	+	3456	protein_coding	polyadenylate binding protein-interacting protein 1 [Source:MGI Symbol;Acc:MGI:2384993]	-
ENSMUSG0000003372	338.7105702	361.2705797	237.9547893	407.4096035	531.3548305	515.7034799	312.645313	484.8226379	-0.631750625	0.008434	0.0450357	Sfxn5	6	85213049	85333422	-	9635	protein_coding	sideroflexin 5 [Source:MGI Symbol;Acc:MGI:2137681]	-
ENSMUSG0000007343	88.18500052	67.40908775	51.38929722	31.86925017	34.56769343	33.08286475	68.99446183	33.17326945	1.058984585	0.0084362	0.0450357	D330041H03R	17	24404649	24414542	-	3749	processed_transcrip	RIKEN cDNA D330041H03 gene [Source:MGI Symbol;Acc:MGI:3603827]	-
ENSMUSG0000002180	1991.177228	2142.34507	2638.728696	1636.529063	1580.237414	1741.715526	2257.416998	1652.827334	0.449572026	0.0084405	0.0450458	Rtraf	14	19811351	19823824	-	1927	protein_coding	RNA transcription, translation and transport factor [Source:MGI Symbol;Acc:MGI:1915295]	-
ENSMUSG0000004033	1883.952284	1688.386995	3260.986056	2981.066887	4122.444354	4213.200128	2277.775112	3772.237123	-0.727877317	0.0084488	0.0450771	Fam102b	3	108970997	109027607	-	5702	protein_coding	family with sequence similarity 102, member B [Source:MGI Symbol;Acc:MGI:3036259]	-
ENSMUSG0000002912	0	0	0	39.62122994	0.987648384	0	0	13.53629277	-6.124932356	0.0084788	0.0452241	Ppp2r2c	5	36868513	36955078	+	4450	protein_coding	protein phosphatase 2, regulatory subunit B, gamma [Source:MGI Symbol;Acc:MGI:2442660]	-
ENSMUSG0000004429	16.03363646	27.3849419	24.57748997	7.75197977	5.925890302	4.865127169	22.66535611	6.180999081	1.867683548	0.0084876	0.0452489	Zfp879	11	50832031	50841552	-	2414	protein_coding	zinc finger protein 879 [Source:MGI Symbol;Acc:MGI:3053099]	zf-C2
ENSMUSG0000003511	15.03153418	29.49147589	54.74077313	211.026116	43.45652888	71.03085667	33.08792773	108.5045005	-1.718277946	0.0084903	0.0452489	Wnk4	11	101260567	101277409	+	4148	protein_coding	WNK lysine deficient protein kinase 4 [Source:MGI Symbol;Acc:MGI:1917097]	-
ENSMUSG0000003802	2096.397967	2252.938105	1606.474117	1168.826283	1504.188488	1462.457227	1985.270063	1378.490666	0.526593402	0.0084907	0.0452489	Phf2	13	48801750	48871119	-	5494	protein_coding	PHD finger protein 2 [Source:MGI Symbol;Acc:MGI:1338034]	-
ENSMUSG0000010882	51.10721621	47.39701482	60.3265663	19.81061497	26.66650636	26.27168671	52.94359911	24.24960268	1.128812753	0.0084951	0.0452596	Gm45838	7	83633371	83635817	+	2447	TEC	predicted gene 45838 [Source:MGI Symbol;Acc:MGI:5804953]	-
ENSMUSG0000010482	1.002102279	11.58593696	8.937269082	0	0	0	7.175102772	0	5.374651225	0.0085126	0.0453398	Gm43182	5	43782243	43784770	+	2528	TEC	predicted gene 43182 [Source:MGI Symbol;Acc:MGI:5663319]	-
ENSMUSG0000002048	703.4757996	876.3181408	769.7222997	490.9587188	613.3296463	569.2198788	783.17208	557.8360813	0.489895987	0.0085171	0.045351	Ccdc117	11	5528887	5542187	-	3372	protein_coding	coiled-coil domain containing 117 [Source:MGI Symbol;Acc:MGI:2144383]	-
ENSMUSG0000004929	428.8997752	360.2173127	454.6835645	568.4785165	604.4408109	560.4626499	414.6002175	577.7939924	-0.478954525	0.0085278	0.045395	Trappc1	11	69323980	69325793	+	1112	protein_coding	trafficking protein particle complex 1 [Source:MGI Symbol;Acc:MGI:1098727]	-
ENSMUSG0000002430	327.6874451	343.3650407	267.0009138	448.7534956	411.849376	499.1620475	312.6844666	453.254973	-0.535030555	0.0085312	0.0454004	Myo1f	17	33555719	33607764	+	4914	protein_coding	myosin IF [Source:MGI Symbol;Acc:MGI:107711]	-
ENSMUSG0000003382	122.256478	57.92968479	67.02951811	191.215501	142.2213673	146.9268405	82.40522696	160.1212363	-0.95695551	0.0085339	0.0454017	Tpsb2	17	25366550	25368094	+	1081	protein_coding	tryptase beta 2 [Source:MGI Symbol;Acc:MGI:96942]	-
ENSMUSG0000002835	52.10931849	57.92968479	44.68634541	106.8050546	80.98716747	95.35649251	51.57511623	94.38290486	-0.872021956	0.008543	0.045437	Zfp618	4	62965573	63139708	+	9352	protein_coding	zinc finger protein 618 [Source:MGI Symbol;Acc:MGI:1919950]	zf-C2
ENSMUSG0000005288	88.18500052	69.51562174	84.90405628	120.586352	134.3201802	152.7649931	80.86822618	135.8905084	-0.747850452	0.0085497	0.0454508	Prkcb	7	122288751	122634402	+	7651	protein_coding	protein kinase C, beta [Source:MGI Symbol;Acc:MGI:97596]	-
ENSMUSG0000000191	1234.590007	1188.085172	879.2038459	1499.57742	1512.089676	1635.655754	1100.626342	1549.107617	-0.492746082	0.0085504	0.0454508	Nacc1	8	84670483	84687862	-	4300	protein_coding	nucleus accumbens associated 1, BEN and BTB (POZ) domain containing [Source:MGI Symbol;Acc:MGI:1914080]	-
ENSMUSG0000005636	34.07147747	52.6633498	36.86623496	102.4983992	74.07362878	72.0038821	41.20035408	82.85863669	-1.009880216	0.0085571	0.0454733	Actr3b	5	25759997	25850688	+	2425	protein_coding	ARP3 actin-related protein 3B [Source:MGI Symbol;Acc:MGI:2661120]	-
ENSMUSG0000004132	21.04414785	30.54474289	37.9833936	203.2741362	40.49358373	46.70522082	29.85742811	96.82431358	-1.701199469	0.0085752	0.0455568	Inhba	13	16011851	16031621	+	6390	protein_coding	inhibin beta-A [Source:MGI Symbol;Acc:MGI:96570]	-
ENSMUSG0000002875	3717.799454	3758.056642	2999.570936	2435.84431	2701.21833	2683.604146	3491.80901	2606.888929	0.421814876	0.0085858	0.0456002	Hp1bp3	4	138216296	138244683	+	8433	protein_coding	heterochromatin protein 1, binding protein 3 [Source:MGI Symbol;Acc:MGI:109369]	-
ENSMUSG0000002405	20.04204557	14.74573795	36.86623496	134.3676494	45.43182565	39.89404278	23.88467283	73.2311726	-1.621850944	0.0086075	0.0457023	Clip4	17	71768473	71864273	+	8514	protein_coding	CAP-GLY domain containing linker protein family, member 4 [Source:MGI Symbol;Acc:MGI:1919100]	-
ENSMUSG0000003059	177.3721033	184.3217243	165.339478	127.4770007	107.6536738	97.30254338	175.6777685	110.8110726	0.663494832	0.0086171	0.0457396	Fam98c	7	29152510	29156234	-	2713	protein_coding	family with sequence similarity 98, member C [Source:MGI Symbol;Acc:MGI:1921083]	-
ENSMUSG0000003079	828.7385844	396.0283905	585.3911249	260.9833189	392.0964083	383.3720209	603.3860333	345.4839161	0.805310536	0.0086194	0.0457396	Adm	7	110627661	110629820	+	1619	protein_coding	adrenomedullin [Source:MGI Symbol;Acc:MGI:108058]	-
ENSMUSG0000002158	2911.107119	2816.435948	2751.561719	3266.167477	4112.56787	3825.936006	2826.368262	3734.890451	-0.401965485	0.0086244	0.0457532	Cast	13	74692368	74808810	-	11470	protein_coding	calpastatin [Source:MGI Symbol;Acc:MGI:1098236]	-
ENSMUSG0000000318	1123.356654	997.4438453	1196.476898	851.8564436	830.6122907	812.4762372	1105.759133	831.6483239	0.410792788	0.0086356	0.0457994	Irf3	7	44997648	45002848	+	3442	protein_coding	interferon regulatory factor 3 [Source:MGI Symbol;Acc:MGI:1859179]	IRF
ENSMUSG0000003260	1787.750465	1875.86852	1657.863415	1446.174893	1333.325318	1307.746183	1773.827467	1362.415465	0.380613806	0.0086458	0.0458404	Qars	9	108507706	108515941	+	6572	protein_coding	glutaminyl-tRNA synthetase [Source:MGI Symbol;Acc:MGI:1915851]	-
ENSMUSG0000002976	209.4393762	159.0433164	249.1263757	287.6845826	299.2574603	446.6186741	205.8696894	344.520239	-0.742943137	0.008654	0.045871	Akr1b8	6	34354119	34368463	+	1499	protein_coding	aldo-keto reductase family 1, member B8 [Source:MGI Symbol;Acc:MGI:107673]	-
ENSMUSG0000008763	20.04204557	40.02414585	39.10055223	12.91996628	7.90118707	11.67630521	33.05558122	10.83248619	1.603768582	0.0086568	0.0458728	Gm13414	2	34649514	34650240	-	727	processed_pseudog	predicted gene 13414 [Source:MGI Symbol;Acc:MGI:3652154]	-
ENSMUSG0000000035	201.422558	182.2151903	136.2933535	354.0070762	262.7144701	237.4182058	173.3103673	284.7132507	-0.715965206	0.0086649	0.045903	Rem1	2	152626951	152635198	+	2156	protein_coding	rad and gem related GTP binding protein 1 [Source:MGI Symbol;Acc:MGI:1097696]	-
ENSMUSG0000002082	1587.330009	2061.243511	1424.37726	2157.634369	2477.022146	2602.843035	1690.983594	2412.49985	-0.512506153	0.0086687	0.0459102	Sec14l1	11	117115168	117159268	+	7176	protein_coding	SEC14-like lipid binding 1 [Source:MGI Symbol;Acc:MGI:1921386]	-
ENSMUSG0000005408	78.16397773	102.1668986	107.247229	35.31457451	38.51828697	67.13875493	95.85936844	46.9905388	1.029999643	0.0086883	0.046001	Capn12	7	28881422	28893563	+	4770	protein_coding	calpain 12 [Source:MGI Symbol;Acc:MGI:1891369]	-
ENSMUSG0000002600	5412.354407	6661.91375	3625.179771	2813.968657	3635.533701	3613.816461	5233.149309	3354.439606	0.641716749	0.0086942	0.0460191	Acadl	1	66830839	66863277	-	2005	protein_coding	acyl-Coenzyme A dehydrogenase, long-chain [Source:MGI Symbol;Acc:MGI:87866]	-
ENSMUSG0000007434	8.016818229	14.74573795	7.820110447	47.37320971	28.64180313	22.37958498	10.19422221	32.79819927	-1.688266879	0.0087357	0.0462257	Tnfaip8l3	9	54023405	54068388	-	4585	protein_coding	tumor necrosis factor, alpha-induced protein 8-like 3 [Source:MGI Symbol;Acc:MGI:2685363]	-
ENSMUSG0000003170	11157.40677	14645.67758	4753.509993	4260.143549	6154.037079	5809.934865	10185.53145	5408.038498	0.913390184	0.0087519	0.0462982	Gpt2	8	85492576	85527560	+	5643	protein_coding	glutamic pyruvate transaminase (alanine aminotransferase) 2 [Source:MGI Symbol;Acc:MGI:1915391]	-
ENSMUSG0000003544	305.641195	308.6072299	328.4446388	492.681381	475.0588726	381.42597	314.2310212	449.7220745	-0.517820464	0.0087721	0.0463917	Thyn1	9	26999710	27007336	+	2813	protein_coding	thymocyte nuclear protein 1 [Source:MGI Symbol;Acc:MGI:1925112]	-
ENSMUSG0000010803	21.04414785	27.3849419	25.69464861	6.029317599	6.913538686	9.730254338	24.70791279	7.557703541	1.712199884	0.0087782	0.0464112	9530062K07Ri	6	116199408	116207993	-	787	bidirectional_prom	RIKEN cDNA 9530062K07 gene [Source:MGI Symbol;Acc:MGI:1925989]	-
ENSMUSG0000004267	328.6895474	218.0262682	468.0894682	163.6529063	204.4432154	207.2544174	338.2684279	191.783513	0.818771439	0.0088074	0.0464437	Dcst1	3	89350219	89365253	-	5241	protein_coding	DC-STAMP domain containing 1 [Source:MGI Symbol;Acc:MGI:1925022]	-
ENSMUSG0000006183	2114.435808	2248.725037	1603.122642	1334.201852	1535.793237	1378.77704	1988.761162	1416.257376	0.490039712	0.0088384	0.0467029	Suclg2	6	95473009	95718800	-	4623	protein_coding	succinate-Coenzyme A ligase, GDP-forming, beta subunit [Source:MGI Symbol;Acc:MGI:1306824]	-
ENSMUSG0000002057	205.4309671	125.3387725	118.4188153	243.7566972	233.0850186	287.042503	149.7295183	254.6280729	-0.764273132	0.0088566	0.0467859	Pik3cg	12	32173473	32208659	-	8426	protein_coding	phosphatidylinositol-4,5-bisphosphate 3-kinase catalytic subunit gamma [Source:MGI Symbol;Acc:MGI:1353576]	-
ENSMUSG0000009433	1.002102279	3.159800988	0	12.0586352	11.8517806	13.62235607	1.387301089	12.51092396	-3.160985891	0.0088811	0.0469022	Igkv1-117	6	68121046	68121827	+	404	IG_V_gene	immunoglobulin kappa variable 1-117 [Source:MGI Symbol;Acc:MGI:4439721]	-
ENSMUSG0000002007	2187.589274	2286.642649	2273.417823	1800.181969	1673.076362	1823.449663	2249.216582	1765.569331	0.349191509	0.0088899	0.0469356	Ddx50	10	62615895	62651218	-	10129	protein_coding	DEAD (Asp-Glu-Ala-Asp) box polypeptide 50 [Source:MGI Symbol;Acc:MGI:2182303]	-
ENSMUSG0000002532	24.05045469	12.63920395	7.820110447	35.31457451	48.3947708	43.78614452	14.8365897	42.49849661	-1.509601447	0.0088938	0.0469387	Itgb8	12	119158022	119238802	-	11060	protein_coding	integrin beta 8 [Source:MGI Symbol;Acc:MGI:1338035]	-
ENSMUSG0000003353	228.4793195	308.6072299	493.7841168	164.5142373	220.2455896	192.6590359	343.6235554	192.4729543	0.835921336	0.0088956	0.0469387	Casp4	9	5308828	5336783	+	3710	protein_coding	caspase 4, apoptosis-related cysteine peptidase [Source:MGI Symbol;Acc:MGI:107700]	-
ENSMUSG0000000319	747.5682998	624.5873287	768.7010848	897.5069912	887.895897	977.8905609	685.2855711	921.0978164	-0.426410275	0.0089054	0.0469675	Mpnd	17	56005711	56016790	+	2305	protein_coding	MPN domain containing [Source:MGI Symbol;Acc:MGI:1915297]	-
ENSMUSG0000008635	82.17238685	55.82315079	89.37269082	24.97860148	42.4688805	39.89404278	75.78940949	35.78050826	1.086242896	0.008906	0.0469675	B230369F24Ri	1	178313328	178318549	-	2560	antisense	RIKEN cDNA B230369F24 gene [Source:MGI Symbol;Acc:MGI:2443392]	-
ENSMUSG0000002532	213.4477853	269.636351	268.1180725	155.0395954	182.714951	155.6840694	250.4007363	164.4795386	0.606315807	0.0089202	0.0470281	Sp4	12	118234933	118301440	-	5770	protein_coding	trans-acting transcription factor 4 [Source:MGI Symbol;Acc:MGI:107595]	zf-C2
ENSMUSG0000004048	8932.739712	5583.368346	4887.569029	11314.44514	10294.2591	8796.149921	6467.892362	10134.95139	-0.647926697	0.0089225	0.0470281	Ltbp4	7	27305136	27337692	-	6463	protein_coding	latent transforming growth factor beta binding protein 4 [Source:MGI Symbol;Acc:MGI:1321395]	-
ENSMUSG0000002517	64.13454583	40.02414585	33.51475906	161.068913	58.27125464	105.0867468	45.89115025	108.1423048	-1.236081426	0.0089453	0.0471345	Hoga1	19	42045610	42070953	+	2259	protein_coding	4-hydroxy-2-oxoglutarate aldolase 1 [Source:MGI Symbol;Acc:MGI:1914682]	-
ENSMUSG0000002280	59.12403444	17.90553893	50.27213858	68.04515576	164.9372801	95.35649251	42.43390399	109.4463095	-1.365301222	0.0089492	0.0471419	Popdc2	16	38362209	38378216	+	2360	protein_coding	popeye domain containing 2 [Source:MGI Symbol;Acc:MGI:1930150]	-
ENSMUSG0000001840	636.3349469	758.3522372	599.9141871	867.3604032	986.6607354	890.3182719	664.8671237	914.7798035	-0.460064478	0.0089525	0.0471461	Mtmr4	11	87592162	87616302	+	6240	protein_coding	myotubularin related protein 4 [Source:MGI Symbol;Acc:MGI:2180699]	-
ENSMUSG0000003299	1044.190574	1339.755619	1379.690914	811.3738826	944.1918549	939.942569	1254.545703	898.5027688	0.481587265	0.0089656	0.047202	Foxj3	4	119537004	119629119	+	10973	protein_coding	forkhead box J3 [Source:MGI Symbol;Acc:MGI:2443432]	Fork
ENSMUSG0000002866	868.8226756	740.4466983	774.1909342	643.4143209	494.8118403	567.2738279	794.4867693	568.4999964	0.4824529	0.0089808	0.0472684	Pithd1	4	135975460	135987264	-	2477	protein_coding	PITH (C-terminal proteasome-interacting domain of thioredoxin-like) domain containing 1 [Source:MGI Symbol;Acc:MGI:191344	-
ENSMUSG0000003678	482.011196	232.7720061	268.1180725	457.3668064	828.636994	529.325836	327.6337582	605.1098788	-0.884166544	0.0089839	0.0472716	Klhl13	X	23219271	23365082	-	4253	protein_coding	kelch-like 13 [Source:MGI Symbol;Acc:MGI:1914705]	-
ENSMUSG0000007570	9250.406134	7165.375374	10207.47845	11286.02121	12435.4808	13359.63921	8874.419986	12360.38041	-0.477999459	0.0089983	0.0473088	Gpx4	10	80047166	80056439	+	1724	protein_coding	glutathione peroxidase 4 [Source:MGI Symbol;Acc:MGI:104767]	-
ENSMUSG0000002044	816.7133571	1132.262021	1505.92984	757.9713553	726.9092104	744.3644568	1151.635073	743.0816742	0.631707823	0.0089979	0.0473184	Rnf185	11	3415982	3452363	-	2906	protein_coding	ring finger protein 185 [Source:MGI Symbol;Acc:MGI:1922078]	-
ENSMUSG0000003840	1.002102279	0	0	30.146588	3.950593535	0.973025434	0.334034093	11.69006899	-4.953519619	0.0090017	0.0473255	1700018A04Ri	13	31565492	31625792	-	4216	lincRNA	RIKEN cDNA 1700018A04 gene [Source:MGI Symbol;Acc:MGI:1916631]	-
ENSMUSG0000003468	1088.283075	1142.794691	1062.417862	894.0616668	835.5505326	754.0947112	1097.831876	827.9023036	0.406873714	0.0090159	0.0473865	Rnps1	17	24414675	24425895	+	1920	protein_coding	ribonucleic acid binding protein S1 [Source:MGI Symbol;Acc:MGI:97960]	-
ENSMUSG0000004202	272.5718198	304.3941619	270.3523897	170.5435549	152.0978571	224.7688752	282.4394571	182.4700937	0.630623807	0.0090247	0.0474194	Ncapg2	12	116405402	116463731	+	9939	protein_coding	non-SMC condensin II complex, subunit G2 [Source:MGI Symbol;Acc:MGI:1923294]	-
ENSMUSG0000003049	547.1478441	528.740032	431.2232332	384.1536642	350.6151762	317.2062914	502.3703698	350.6583773	0.518635474	0.0090441	0.0474956	Kctd15	7	34639014	34656732	-	4238	protein_coding	potassium channel tetramerisation domain containing 15 [Source:MGI Symbol;Acc:MGI:2385276]	-
ENSMUSG0000003653	534.1205145	554.0184399	516.1272895	379.8470087	419.7505631	384.3450463	534.7554146	394.6475394	0.438714751	0.0090442	0.0474956	Cdc42ep3	17	79333727	79355091	-	2309	protein_coding	CDC42 effector protein (Rho GTPase binding) 3 [Source:MGI Symbol;Acc:MGI:2384718]	-
ENSMUSG0000002218	3529.404225	4088.782479	2668.89198	4183.485083	5351.078943	5590.031117	3429.026228	5041.531714	-0.555938257	0.0090693	0.0476139	Oxct1	15	4026383	4155344	+	3701	protein_coding	3-oxoacid CoA transferase 1 [Source:MGI Symbol;Acc:MGI:1914291]	-
ENSMUSG0000004510	519.0889803	532.9531	421.1688055	745.051389	614.3172947	721.0118464	491.0702953	693.4601767	-0.49771109	0.0090762	0.0476369	Dmd	X	82948870	85206141	+	23801	protein_coding	dystrophin, muscular dystrophy [Source:MGI Symbol;Acc:MGI:94909]	-
ENSMUSG0000002263	984.0644376	1009.029782	1323.832983	524.5506311	800.9828392	828.0446441	1105.642401	717.8593715	0.62343351	0.0091143	0.0478112	Cblb	16	52031225	52208048	+	10475	protein_coding	Casitas B-lineage lymphoma b [Source:MGI Symbol;Acc:MGI:2146430]	-
ENSMUSG0000003729	282.5928426	268.583084	309.452942	415.1615833	370.3681439	425.2121146	286.8762895	403.5806139	-0.493003394	0.0091146	0.0478112	Lsm1	8	25785288	25803975	+	3093	protein_coding	LSM1 homolog, mRNA degradation associated [Source:MGI Symbol;Acc:MGI:1914457]	-
ENSMUSG0000003488	27.05676152	12.63920395	12.28874499	65.4611625	47.40712242	32.10983931	17.32823682	48.32604141	-1.476362829	0.0091172	0.0478114	Tbxa2r	10	81328731	81335172	+	1919	protein_coding	thromboxane A2 receptor [Source:MGI Symbol;Acc:MGI:98496]	-
ENSMUSG0000006197	1.002102279	2.106533992	0	8.613310856	11.8517806	12.64933064	1.03621209	11.0381407	-3.395331819	0.0091358	0.0478958	Cldn24	8	47822143	47822805	+	663	protein_coding	claudin 24 [Source:MGI Symbol;Acc:MGI:3712484]	-
ENSMUSG0000003270	405.8514228	385.4957206	434.5747091	260.9833189	315.0598344	292.8806556	408.6406175	289.6412696	0.497141115	0.0091598	0.0479963	Exd2	12	80463095	80500227	+	8337	protein_coding	exonuclease 3'-5' domain containing 2 [Source:MGI Symbol;Acc:MGI:1922485]	-
ENSMUSG0000002341	283.5949448	290.7016909	306.1014661	236.8660485	170.8631704	138.1696116	293.4660339	181.9662768	0.687574425	0.0091601	0.0479963	Nfatc4	14	55823144	55833943	+	5808	protein_coding	nuclear factor of activated T cells, cytoplasmic, calcineurin dependent 4 [Source:MGI Symbol;Acc:MGI:1920431]	RHD
ENSMUSG0000003138	1.002102279	9.479402965	1.117158635	55.12518948	14.81472576	10.70327977	3.866221293	26.881065	-2.799884232	0.0091727	0.048049	Asb11	X	164436991	164459170	+	2847	protein_coding	ankyrin repeat and SOCS box-containing 11 [Source:MGI Symbol;Acc:MGI:1916104]	-
ENSMUSG0000001594	394.8282978	371.8032496	320.6245283	509.9080027	528.3918853	477.755488	362.4186919	505.351792	-0.479009406	0.0091891	0.0481214	Castor2	5	134099711	134144343	+	7560	protein_coding	cytosolic arginine sensor for mTORC1 subunit 2 [Source:MGI Symbol;Acc:MGI:1933384]	-
ENSMUSG0000005357	35.07357975	24.22514091	31.28044179	12.0586352	6.913538686	11.67630521	30.19305415	10.2161597	1.559685964	0.0092026	0.0481787	4930563E22Ri	11	72215138	72218450	+	3313	processed_transcrip	RIKEN cDNA 4930563E22 gene [Source:MGI Symbol;Acc:MGI:1922554]	-
ENSMUSG0000002620	10.02102279	13.69247095	21.22601407	4.306655428	0.987648384	1.946050868	14.97983593	2.41345156	2.609731844	0.0092182	0.0482466	Slc23a3	1	75125541	75133890	-	2167	protein_coding	solute carrier family 23 (nucleobase transporters), member 3 [Source:MGI Symbol;Acc:MGI:104516]	-
ENSMUSG0000002602	944.9824487	1149.114293	851.27488	1227.396797	1427.151915	1462.457227	981.7905405	1372.335313	-0.482840881	0.009223	0.0482581	Trak2	1	58900440	58973430	-	7088	protein_coding	trafficking protein, kinesin binding 2 [Source:MGI Symbol;Acc:MGI:1918077]	-
ENSMUSG0000010818	19.03994329	11.58593696	6.702951811	47.37320971	18.76531929	59.35455146	12.44294402	41.83102682	-1.744012568	0.0092288	0.0482749	4930511E03Ri	6	94943834	94951545	+	2232	lincRNA	RIKEN cDNA 4930511E03 gene [Source:MGI Symbol;Acc:MGI:1926023]	-
ENSMUSG0000004852	29.06096608	25.27840791	15.64022089	85.27177747	50.37006757	39.89404278	23.32653163	58.51196261	-1.325877693	0.0092336	0.0482866	Fbxl13	5	21483847	21645634	-	5439	protein_coding	F-box and leucine-rich repeat protein 13 [Source:MGI Symbol;Acc:MGI:2443416]	-
ENSMUSG0000006759	3.006306836	3.159800988	13.40590362	31.00791908	23.70356121	25.29866128	6.524003816	26.67004719	-2.046175148	0.0092441	0.0483227	Klra7	6	130218605	130233322	-	1265	protein_coding	killer cell lectin-like receptor, subfamily A, member 7 [Source:MGI Symbol;Acc:MGI:101901]	-
ENSMUSG0000007539	385.8093773	241.1981421	226.7832029	432.388205	468.1453339	458.2949793	284.5969074	452.9428394	-0.669196192	0.0092477	0.0483227	Hoxc4	15	103018934	103036852	+	2568	protein_coding	homeobox C4 [Source:MGI Symbol;Acc:MGI:96195]	Hom
ENSMUSG0000005691	947.9887556	1093.291142	974.1623299	1226.535466	1627.644536	1321.368539	1005.147409	1391.849514	-0.469381365	0.0092482	0.0483227	Sipa1	19	5651185	5663707	-	3868	protein_coding	signal-induced proliferation associated gene 1 [Source:MGI Symbol;Acc:MGI:107576]	-
ENSMUSG0000005523	36.07568203	28.43820889	14.52306226	77.5197977	42.4688805	75.89598383	26.34565106	65.29488735	-1.306103379	0.0092541	0.04834	Wdr86	5	24711738	24730727	-	5034	protein_coding	WD repeat domain 86 [Source:MGI Symbol;Acc:MGI:1915466]	-
ENSMUSG0000002627	210.4414785	338.0987057	234.6033134	509.9080027	381.2322761	370.7226903	261.0478326	420.6209897	-0.688911553	0.0092718	0.0484189	Bok	1	93685660	93695764	+	1840	protein_coding	BCL2-related ovarian killer [Source:MGI Symbol;Acc:MGI:1858494]	-
ENSMUSG0000002404	1853.889215	1801.086563	2253.308967	1521.110697	1522.953808	1297.042903	1969.428249	1447.035803	0.444455841	0.0092812	0.0484543	Akap8	17	32303676	32321153	-	3695	protein_coding	A kinase (PRKA) anchor protein 8 [Source:MGI Symbol;Acc:MGI:1928488]	-
ENSMUSG0000002606	46.09670482	48.45028182	101.6614358	83.5491153	214.3196993	152.7649931	65.40280748	150.2112692	-1.200707824	0.0092869	0.0484707	Ptp4a1	1	30940302	30949309	-	5541	protein_coding	protein tyrosine phosphatase 4a1 [Source:MGI Symbol;Acc:MGI:1277096]	-
ENSMUSG0000000103	40.08409114	43.18394684	58.09224903	141.258298	87.90070615	67.13875493	47.12009567	98.76591971	-1.071544549	0.0092963	0.048497	B9d1	11	61505144	61512931	+	1593	protein_coding	B9 protein domain 1 [Source:MGI Symbol;Acc:MGI:1351471]	-
ENSMUSG0000002840	273.5739221	400.2414585	403.2942673	460.8121308	590.6137335	595.4915655	359.0365493	548.9724766	-0.612841102	0.0092971	0.048497	Zdhhc21	4	82798738	82859958	-	12972	protein_coding	zinc finger, DHHC domain containing 21 [Source:MGI Symbol;Acc:MGI:1915518]	-
ENSMUSG0000002274	343.7210816	302.2876279	358.6079219	255.8153324	227.1591283	207.2544174	334.8722105	230.0762927	0.540507649	0.0093107	0.048554	Cpox	16	58670292	58717636	+	6661	protein_coding	coproporphyrinogen oxidase [Source:MGI Symbol;Acc:MGI:104841]	-
ENSMUSG0000006102	945.984551	762.5653052	1175.250884	633.939679	669.6256042	680.1447782	961.2669135	661.2366871	0.539638869	0.0093205	0.0485916	Rrs1	1	9545408	9547455	+	2048	protein_coding	ribosome biogenesis regulator 1 [Source:MGI Symbol;Acc:MGI:1929721]	-
ENSMUSG0000010825	26.05465924	55.82315079	54.74077313	16.36529063	19.75296767	16.54143237	45.53952772	17.55323023	1.374597507	0.0093416	0.0486882	Gm43923	6	93194941	93197851	+	2911	TEC	predicted gene, 43923 [Source:MGI Symbol;Acc:MGI:5690315]	-
ENSMUSG0000009777	51.10721621	43.18394684	45.80350404	8.613310856	23.70356121	21.40655954	46.69822236	17.90781054	1.390840087	0.0093566	0.048753	Gm10614	8	120589531	120593801	+	2538	bidirectional_prom	predicted gene 10614 [Source:MGI Symbol;Acc:MGI:3642104]	-
ENSMUSG0000001567	1216.552166	1156.487162	1286.966748	1710.603536	1492.336708	1575.328177	1220.002025	1592.75614	-0.384909122	0.0093637	0.048776	Psma2	13	14613240	14674236	+	3673	protein_coding	proteasome (prosome, macropain) subunit, alpha type 2 [Source:MGI Symbol;Acc:MGI:104885]	-
ENSMUSG0000002005	8.016818229	63.19601977	124.0046085	24.97860148	2.962945151	8.757228904	65.07248217	12.23292518	2.40225167	0.0093792	0.0488311	Pah	10	87521795	87584136	+	2201	protein_coding	phenylalanine hydroxylase [Source:MGI Symbol;Acc:MGI:97473]	-
ENSMUSG0000007694	2.004204557	0	0	10.33597303	2.962945151	24.32563584	0.668068186	12.54151801	-4.18596629	0.0093795	0.0488311	Iglv2	16	19260401	19260859	-	366	IG_V_gene	immunoglobulin lambda variable 2 [Source:MGI Symbol;Acc:MGI:99548]	-
ENSMUSG0000002496	1161.436541	1140.688157	1116.041477	1378.129737	1599.990382	1470.24143	1139.388725	1482.787183	-0.379796868	0.0093934	0.0488901	Mark2	19	7275396	7341860	-	7942	protein_coding	MAP/microtubule affinity regulating kinase 2 [Source:MGI Symbol;Acc:MGI:99638]	-
ENSMUSG0000002918	0	0	2.23431727	55.98652056	1.975296767	0.973025434	0.744772423	19.64494759	-4.762354427	0.0094104	0.048965	Slc34a2	5	53038081	53071664	+	5013	protein_coding	solute carrier family 34 (sodium phosphate), member 2 [Source:MGI Symbol;Acc:MGI:1342284]	-
ENSMUSG0000009774	47.0988071	49.50354882	41.3348695	19.81061497	19.75296767	22.37958498	45.97907514	20.64772254	1.156202585	0.0094257	0.0490308	Gm26534	11	60879118	60887218	+	1724	lincRNA	predicted gene, 26534 [Source:MGI Symbol;Acc:MGI:5477028]	-
ENSMUSG0000002917	509.0679575	507.6746921	417.8173296	888.0323492	569.8731174	696.6862106	478.1866597	718.1972258	-0.587042095	0.0094374	0.0490784	Tbc1d1	5	64156305	64351486	+	7405	protein_coding	TBC1 domain family, member 1 [Source:MGI Symbol;Acc:MGI:1889508]	-
ENSMUSG0000003062	97.20392103	63.19601977	65.91235948	38.75989885	43.45652888	25.29866128	75.43743342	35.838363	1.074380494	0.0094505	0.0491326	Me3	7	89632392	89854359	+	14624	protein_coding	malic enzyme 3, NADP(+)-dependent, mitochondrial [Source:MGI Symbol;Acc:MGI:1916679]	-
ENSMUSG0000000002	70.1471595	78.99502471	50.27213858	171.404886	88.88835454	127.4663318	66.47144093	129.2531908	-0.959728333	0.0094563	0.049149	Cdc45	16	18780447	18811987	-	3506	protein_coding	cell division cycle 45 [Source:MGI Symbol;Acc:MGI:1338073]	-
ENSMUSG0000002270	1297.722451	1945.384142	981.9824404	1397.079021	3671.089042	2938.53681	1408.363011	2668.901624	-0.922053576	0.0094693	0.0491917	Gbe1	16	70313949	70569716	+	5352	protein_coding	glucan (1,4-alpha-), branching enzyme 1 [Source:MGI Symbol;Acc:MGI:1921435]	-
ENSMUSG0000003031	71.14926178	25.27840791	20.10885543	154.1782643	79.0118707	79.78808557	38.84550837	104.3260735	-1.422532888	0.0094698	0.0491917	Cand2	6	115774538	115805557	+	5474	protein_coding	cullin-associated and neddylation-dissociated 2 (putative) [Source:MGI Symbol;Acc:MGI:1914338]	-
ENSMUSG0000006028	26.05465924	47.39701482	40.21771087	122.3090142	83.95011262	52.54337342	37.88979498	86.26750007	-1.190432081	0.0094743	0.0492019	Ppih	4	119300010	119320546	-	1894	protein_coding	peptidyl prolyl isomerase H [Source:MGI Symbol;Acc:MGI:106499]	-
ENSMUSG0000009757	68.14295495	64.24928676	65.91235948	27.56259474	42.4688805	27.24471215	66.10153373	32.4253958	1.030324906	0.0094864	0.0492506	6230400D17Ri	14	20687236	20703028	-	2472	antisense	RIKEN cDNA 6230400D17 gene [Source:MGI Symbol;Acc:MGI:1923383]	-
ENSMUSG0000002070	1891.969102	1822.151903	1946.090343	1496.132096	1407.398947	1528.622956	1886.737116	1477.384666	0.352760691	0.0094946	0.0492799	Ddx42	11	106216926	106249139	+	4228	protein_coding	DEAD (Asp-Glu-Ala-Asp) box polypeptide 42 [Source:MGI Symbol;Acc:MGI:1919297]	-
ENSMUSG0000004799	0	1.053266996	1.117158635	9.474641942	8.888835454	10.70327977	0.72347521	9.688919056	-3.769922919	0.0094984	0.049286	C2cd4a	9	67830489	67832330	-	1395	protein_coding	C2 calcium-dependent domain containing 4A [Source:MGI Symbol;Acc:MGI:3645763]	-
ENSMUSG0000011442	21.04414785	22.11860692	41.3348695	5.167986514	7.90118707	11.67630521	28.16587476	8.24849293	1.775813729	0.0095042	0.049302	Gm47414	14	29958071	29961381	-	2151	lincRNA	predicted gene, 47414 [Source:MGI Symbol;Acc:MGI:6096350]	-
ENSMUSG0000003370	586.229833	572.9772459	685.935402	500.4333607	394.0717051	404.7785805	615.0474936	433.0945488	0.504980614	0.0095413	0.049481	Acbd6	1	155558120	155691330	+	6453	protein_coding	acyl-Coenzyme A binding domain containing 6 [Source:MGI Symbol;Acc:MGI:1919732]	-
ENSMUSG0000003482	1271.667792	1469.30746	1562.904931	1132.650378	947.1548	1094.653613	1434.626727	1058.15293	0.438733009	0.0095454	0.0494887	Cpsf7	19	10525244	10547735	+	3604	protein_coding	cleavage and polyadenylation specific factor 7 [Source:MGI Symbol;Acc:MGI:1917826]	-
ENSMUSG0000006714	109.2291484	85.31462668	105.0129117	53.40252731	57.28360626	60.32757689	99.85222892	57.00457015	0.809658929	0.0095636	0.0495681	Rpl7a-ps11	X	166854211	166855011	+	801	processed_pseudog	ribosomal protein L7A, pseudogene 11 [Source:MGI Symbol;Acc:MGI:3644020]	-
ENSMUSG0000003166	233.4898309	120.0724376	137.4105121	357.4524005	306.170999	222.8228243	163.6575935	295.4820746	-0.851693775	0.0095661	0.0495681	Nkd1	8	88521354	88594884	+	4558	protein_coding	naked cuticle 1 [Source:MGI Symbol;Acc:MGI:2135954]	-
ENSMUSG0000003244	5.010511393	5.26633498	0	24.97860148	16.79002252	16.54143237	3.425615458	19.43668546	-2.490813334	0.0095741	0.0495844	Eomes	9	118478212	118486132	+	4155	protein_coding	eomesodermin [Source:MGI Symbol;Acc:MGI:1201683]	T-box
ENSMUSG0000003431	4.008409114	6.319601977	5.585793176	33.59191234	7.90118707	30.16378845	5.304601422	23.88562929	-2.175900806	0.0095745	0.0495844	Kif4	X	100625737	100727214	+	5514	protein_coding	kinesin family member 4 [Source:MGI Symbol;Acc:MGI:108389]	-
ENSMUSG0000010687	73.15346634	41.07741285	54.74077313	67.18382468	147.1596092	149.8459168	56.3238841	121.3964502	-1.105157707	0.009583	0.0496148	Gm3289	6	21852981	21858178	-	756	lincRNA	predicted gene 3289 [Source:MGI Symbol;Acc:MGI:3781467]	-
ENSMUSG0000002169	1074.253643	1280.772667	1046.777641	786.3952811	870.1182261	866.9656615	1133.93465	841.1597229	0.431154732	0.0095916	0.0496455	Jmy	13	93430101	93499808	-	8858	protein_coding	junction-mediating and regulatory protein [Source:MGI Symbol;Acc:MGI:1913096]	-
ENSMUSG0000004184	11.02312506	5.26633498	6.702951811	77.5197977	13.82707737	15.56840694	7.664137286	35.63842734	-2.217952205	0.0095976	0.0496637	Fhdc1	3	84442198	84480429	-	6270	protein_coding	FH2 domain containing 1 [Source:MGI Symbol;Acc:MGI:2684972]	-
ENSMUSG0000003547	2.004204557	0	2.23431727	62.01583616	0.987648384	3.892101735	1.412840609	22.29852943	-3.986068588	0.0096029	0.0496765	Slc25a21	12	56712634	57197472	-	4872	protein_coding	solute carrier family 25 (mitochondrial oxodicarboxylate carrier), member 21 [Source:MGI Symbol;Acc:MGI:2445059]	-
ENSMUSG0000002788	77.16187545	70.56888874	70.38099402	138.6743048	120.4931028	107.0327977	72.7039194	122.0667351	-0.748099236	0.0096105	0.0497019	Gpsm2	3	108678638	108722309	-	3909	protein_coding	G-protein signalling modulator 2 (AGS3-like, C. elegans) [Source:MGI Symbol;Acc:MGI:1923373]	-
ENSMUSG0000002440	1118.346143	1044.84086	1063.535021	843.2431328	826.6616972	829.990695	1075.574008	833.2985083	0.368229043	0.0096153	0.049713	Wdr33	18	31804057	31908987	+	6413	protein_coding	WD repeat domain 33 [Source:MGI Symbol;Acc:MGI:1921570]	-
ENSMUSG0000002977	6658.969641	5463.295909	5966.744271	3233.436895	4794.045255	4611.167531	6029.66994	4212.883227	0.517398417	0.0096313	0.0497819	Hibadh	6	52546228	52640389	-	10179	protein_coding	3-hydroxyisobutyrate dehydrogenase [Source:MGI Symbol;Acc:MGI:1889802]	-
ENSMUSG0000009720	10.02102279	18.95880593	11.17158635	3.445324342	0	0	13.38380502	1.148441447	3.485013333	0.0096616	0.0499252	Gm26698	12	86891404	86892567	-	971	lincRNA	predicted gene, 26698 [Source:MGI Symbol;Acc:MGI:5477192]	-
ENSMUSG0000002732	237.49824	250.6775451	304.9843074	217.9167647	105.6783771	95.35649251	264.3866975	139.6505447	0.918046756	0.0096649	0.0499283	Spef1	2	131170261	131187282	-	2747	protein_coding	sperm flagellar 1 [Source:MGI Symbol;Acc:MGI:3513546]	-
ENSMUSG0000006018	230.4835241	231.7187391	268.1180725	434.1108671	367.4051988	298.7188082	243.4401119	366.744958	-0.592409542	0.0096785	0.0499848	Slc35e3	10	117733679	117746365	-	3526	protein_coding	solute carrier family 35, member E3 [Source:MGI Symbol;Acc:MGI:2448489]	-
ENSMUSG0000006710	29.06096608	15.79900494	17.87453816	38.75989885	46.41947404	72.0038821	20.91150306	52.39441833	-1.320338525	0.0096831	0.0499945	Gm5529	1	79922536	79923426	-	891	processed_pseudog	predicted pseudogene 5529 [Source:MGI Symbol;Acc:MGI:3646377]	-

23 39
