# Supplementary material for: LCN2 secreted by tissue-infiltrating neutrophils induces the ferroptosis and wasting of adipose and muscle tissues in lung cancer cachexia
Source: J Hematol Oncol. 2023 Mar 27;16:30. doi: 10.1186/s13045-023-01429-1 (PMC10044814; doi:10.1186/s13045-023-01429-1)
Supplement: Supplementary file 6 — Additional file 6. Table S7: KEGG gene signatures. [file 13045_2023_1429_MOESM6_ESM.pdf]

Table S7. Gene lists of KEGG

KEGGID	KEGG description	Genes
mmu04975	Ferroptosis	Map1lc3b/Steap3/Slc39a8/Slc3a2/Pcbp2/Vdac3/Sat1/Tfrc/Acsl3/Map1lc3a/Alox15/Slc39a14/Acsl1/Ncoa4/Atg7/Gpx4
mmu04216	Fat digestion and absorption	Dgat2/Agpat2/Scarb1/Mogat2/Apoa1/Slc27a4/Plpp3/Acat2/Plpp1/Pla2g2e/Apob/Fabp1/Mttp/Pla2g2c
mmu04979	Cholesterol metabolism	Ldlr/Angptl8/Apoc3/Npc1/Scarb1/Soat2/Mylip/Apoa1/Angptl4/Vdac3/Lipg/Lrp1/Apob/Apoe/Lpl/Lrpap
mmu04668	TNF signaling pathway	Pik3r1/Nfkbia/Edn1/Sele/Lif/Ccl2/Creb3l1/Icam1/Pik3r3/Socs3/Traf3/Tab3/Irf1/Csf1/Mapk11/Gm5431/Map2k6/Il6/Mmp9/Cxcl2/Junb/Ccl5/Casp3/Mapk12/Il1b/Mlkl/Ripk3/Cebpb/Il18r1/Casp
mmu00071	Fatty acid degradation	Acox1/Eci2/Acat2/Acaa2/Cyp4a32/Adh5/Eci1/Acsl3/Cyp4a10/Acads/Aldh7a1/Acsl1/Cpt2/Acad
mmu04920	Adipocytokine signaling pathway	Prkag3/Nfkbia/Adipor2/Acacb/Pck1/Stk11/Stat3/Socs3/Lepr/Rxrb/Irs2/Pck2/Camkk2/Acsl3/Adipor1/Prkag1/Rxrg/Lep/Prkcq/Acsl
mmu04630	JAK-STAT signaling pathway	Il4ra/Pik3r1/Csf2rb2/Csf2rb/Il17d/Jak3/Csf3r/Lif/Egfr/Il6st/Il22ra2/Stat3/Pik3r3/Il2rb/Socs3/Pdgfa/Lepr/Il6ra/Ifngr1/Pim1/Thpo/Ifngr2/Il11ra1/Pdgfb/Il6/Pdgfra/Egf/Ccnd1/Lep/Osmr/Tslp/Lifr/[illegible]
